# Exploring the Prognostic Value, Immune Implication and Biological Function of *H2AFY* Gene in Hepatocellular Carcinoma

**DOI:** 10.3389/fimmu.2021.723293

**Published:** 2021-11-24

**Authors:** Yongbiao Huang, Shanshan Huang, Li Ma, Yali Wang, Xi Wang, Lingyan Xiao, Wan Qin, Long Li, Xianglin Yuan

**Affiliations:** Department of Oncology, Tongji Hospital, Tongji Medical College, Huazhong University of Science and Technology, Wuhan, China

**Keywords:** hepatocellular carcinoma, *H2AFY*, prognosis, immune infiltration, biomarker

## Abstract

**Background:**

Hepatocellular carcinoma (HCC) is an extremely malignant cancer with poor survival. *H2AFY* gene encodes for a variant of H2A histone, and it has been found to be dysregulated in various tumors. However, the clinical value, biological functions and correlations with immune infiltration of *H2AFY* in HCC remain unclear.

**Methods:**

We analyzed the expression and clinical significance of *H2AFY* in HCC using multiple databases, including Oncomine, HCCDB, TCGA, ICGC, and so on. The genetic alterations of *H2AFY* were analyzed by cBioPortal and COSMIC databases. Co-expression networks of *H2AFY* and its regulators were investigated by LinkedOmics. The correlations between *H2AFY* and tumor immune infiltration were explored using TIMER, TISIDB databases, and CIBERSORT method. Finally, *H2AFY* was knocked down with shRNA lentiviruses in HCC cell lines for functional assays *in vitro*.

**Results:**

*H2AFY* expression was upregulated in the HCC tissues and cells. Kaplan–Meier and Cox regression analyses revealed that high *H2AFY* expression was an independent prognostic factor for poor survival in HCC patients. Functional network analysis indicated that *H2AFY* and its co-expressed genes regulates cell cycle, mitosis, spliceosome and chromatin assembly through pathways involving many cancer-related kinases and E2F family. Furthermore, we observed significant correlations between *H2AFY* expression and immune infiltration in HCC. *H2AFY* knockdown suppressed the cell proliferation and migration, promoted cycle arrest, and apoptosis of HCC cells *in vitro*.

**Conclusion:**

Our study revealed that *H2AFY* is a potential biomarker for unfavorable prognosis and correlates with immune infiltration in HCC.

## Introduction

Hepatocellular carcinoma (HCC) is the major pathological type of primary liver cancer, which is an extremely malignant and aggressive cancer with poor clinical outcome and high mortality rate (1, 2). Due to the abuse of alcohol, hepatitis virus infection, and nonalcoholic fatty liver disease, the morbidity of HCC is increasing, and it has gradually become one of the leading causes of cancer-related death worldwide (3, 4). Nowadays, the common treatment methods for HCC include curative surgical resection, liver transplantation, radiation therapy, chemotherapy, immune and molecular-targeted therapy, curative resection is still considered the preferred treatment choice for early HCC (5, 6). On account of lacking the early specific symptoms and effective biomarkers, most HCC patients were usually at an advanced stage when they were first diagnosed, and lost the opportunity for curative resection. Therefore, it is urgent to identify a novel and reliable biomarker which could be helpful for early diagnosis and prognosis prediction of HCC and even serve as a therapeutic target.

In recent years, a growing body of studies suggest that epigenetics regulation mechanisms such as DNA methylation, m6A modification, and histone variants are involved in initiation and development of various human diseases, especially tumorigenesis (7–9). Histone variants can replace their corresponding canonical histones within the nucleosome and alter the composition and structure of chromatin, thereby regulating various fundamental cellular biological processes, and, their dysregulation may lead to cancer initiation and progression (10–12). There are plenty of histone variants, but most of the histone variants are from the H2A histone family. The *H2AFY* gene encodes for H2A variants family member macroH2A1, which has two splicing variant isoforms, macroH2A1.1 and macroH2A1.2 respectively (13). Currently, the role for *H2AFY* in the tumorigenesis and progression of various solid tumors has drawn considerable attention, such as lung cancer, melanoma, breast cancer, colorectal carcinoma, bladder cancer, and gastric cancer, and it has been found to be dysregulated in these tumors (14–19).

Although *H2AFY* has been reported to be highly expressed in HCC which may lead to a lower survival and a poorer prognosis (20, 21), the biological function of *H2AFY* and its relationship with clinicopathological characteristics and tumor immune infiltrates in HCC remain largely unclear. In this study, we comprehensively investigated the expression level, mutations, diagnostic and prognostic significance of *H2AFY* in patients with HCC in various public databases, including Oncomine, HCCDB, The Cancer Genome Atlas (TCGA), International Cancer Genome Consortium (ICGC) and others. Furthermore, through a range of bioinformatics analyses, we explored the potential biological functions and gene regulatory networks correlated with *H2AFY* in HCC, and analyzed the correlation between *H2AFY* and infiltrating immune cells in tumor microenvironment. Additionally, we performed a series of functional assays to further evaluated the effects of *H2AFY* knockdown on HCC cell proliferation, migration, apoptosis, and cell cycle *in vitro*, and our results revealed that *H2AFY* regulates HCC development may in part through the regulation of STAT3 signaling.

## Materials and Methods

### Data Acquisition and Processing

The RNA-seq data, corresponding clinical data, and survival information of HCC patients were obtained from the TCGA database (22), and the details were shown in **Table 1**.

**Table 1:** The clinical characteristics of patients in the TCGA-LIHC cohort.

Characteristic		Total (371)	Percentage (%)
**Status**			
	Dead	130	35.04%
	Live	241	64.96%
**Age at diagnosis**			
	≤65	232	62.53%
	>60	138	37.20%
	Unknown	1	0.27%
**Gender**			
	Female	121	32.61%
	Male	250	67.39%
**Tumor stage**			
	Stage I	171	46.09%
	Stage II	86	23.18%
	Stage III	85	22.91%
	Stage IV	5	1.35%
	Unknown	24	6.47%
**T classification**			
	T1	181	48.79%
	T2	94	25.34%
	T3	80	21.56%
	T4	13	3.50%
	Unknown	3	0.81%
**Grade**			
	G1	55	14.82%
	G2	177	47.72%
	G3	122	32.88%
	G4	12	3.23
	Unknown	5	1.35%

### Differential Expression Analysis of *H2AFY*

We used the Oncomine database to examine the expression of *H2AFY* in liver cancers and normal tissues, set the threshold as: *P*-value as 0.001, fold change (FC) as 1.5, and gene rank as top 10% (23). Besides, we also analyzed the *H2AFY* gene expression level in HCC *via* TIMER database based on TCGA data (24). The HCCDB database contains 15 public HCC datasets which were from the Gene Expression Omnibus (GEO), TCGA, and ICGC, and it was further used for verifying the differential expression of *H2AFY* between HCC and normal tissues (25).

### Genetic Alteration and Survival Analysis

The cBioPortal database and the Catalogue of Somatic Mutations in Cancer (COSMIC) database were utilized to evaluate the alteration frequency and types of *H2AFY* in HCC (26, 27). In the TCGA-LIHC cohort, patients with complete follow-up information were included in survival analyses, Kaplan–Meier curves, receiver operating characteristic (ROC) curves, and Cox regression models were applied to determine the prognostic significance of *H2AFY*. Additionally, the impacts of *H2AFY* expression on overall survival of HCC patients were further validated in the ICGC dataset (LIRI-JP project), Kaplan–Meier Plotter, and GEPIA2 database (28, 29). GeneMANIA was applied to visualize the interaction network of *H2AFY* and predict their function (30).

### Coexpression Analysis in LinkedOmics

LinkedOmics is an online analysis platform that contains multi-dimensional data of 32 TCGA cancer types (31). *H2AFY* co-expression statistical analysis was performed using Spearman correlation test in the “LinkFinder” module, the results were presented in volcano plot and heat maps. The survival heatmaps of top 50 co-expressed genes were plotted by GEPIA2 database. The GO annotation, KEGG pathways, kinase-target enrichment, miRNA-target enrichment, and transcription factor-target enrichment analyses were conducted by gene set enrichment analysis (GSEA) in the “LinkInterpreter” module. The simulations of 500 and the rank criterion was set as false discovery rate (FDR) <0.05.

### GSEA Between *H2AFY* High- and Low-Expression Groups

GSEA analysis was carried out to detect different functional phenotypes between *H2AFY* high- and low-expression groups by using GSEA software (v.4.0.3) based on the expression profile of the TCGA-LIHC dataset (32). KEGG gene set (c2.cp.kegg.v7.4.symbols.gmt) and GO_BP gene set (c5.go.bp.v7.4.symbols.gmt were) were used as the reference gene sets, and 1,000 random permutations were performed per analysis. Nominal *P*-value <0.05 and FDR <0.05 were regarded significant.

### Immune Infiltration Analysis

We used the TIMER database to investigate the correlations between *H2AFY* expression, copy number alterations and the abundance of six major tumor-infiltrating immune cells in HCC. Besides, the correlations between *H2AFY* and immune cell marker genes and several key immune checkpoint genes were also analyzed through the “Correlation” module of TIMER and GEPIA2. Then, we compared the expression of these immune checkpoint genes between patients with high- and low-*H2AFY* expression. The distribution of *H2AFY* expression across immune subtypes were further explored in TISIDB database (33). The relative fractions of 22 immune cell types of patients in TCGA-LIHC cohort were calculated through CIBERSORT algorithm, presenting in bar graphs, heatmap, and violin plot (34, 35).

### Cell Culture and Transfection

The human normal liver cell line L02 and HCC cell lines MHCC-97H, Hep3B, Huh7 and HepG2 were gifts from gastroenterology laboratory and hepatic surgery laboratory of the Tongji Hospital, Wuhan, China. Jurkat cell line was stored in oncology laboratory of the Tongji Hospital, Wuhan, China. L02 cells and Jurkat cells were cultured in RPMI 1640 medium (HyClone, USA) and other hepatoma cells was in DMEM medium (HyClone, USA), with 10% fetal bovine serum (FBS, Gibco, USA), at 37°C in 5% CO2 incubator. The lentiviral *H2AFY*-specific shRNA vectors and negative control (NC) were obtained from OBiO (Shanghai, China). Transfection was carried out with polybrene (OBiO, China). The sequences of H2AFY-shRNAs were listed: *H2AFY*-sh1, 5′-GGATGCTGCGGTACATCAA-3′; *H2AFY*-sh2, 5′-GCTGAAATCCATTGCATTT-3′; *H2AFY*-sh3, 5′-GCGAGAGTATAGGCATCTA-3′; and NC, 5′-TTCTCCGAACGTGTCACGT-3′.

### qRT-PCR

Total RNA from cells was extracted using TRIzol reagent (TaKaRa, Japan) and reverse transcribed by Hi Script II QRT SuperMix (Vazyme, China). The qRT-PCR was carried out using ChamQ Universal SYBR qPCR Master Mix (Vazyme, China). All primers were listed as follows: H2AFY, Forward: CGGATGCTGCGGTACATCAA, Reverse: CTCCGCTGTCAGGTATTCCAG. GAPDH, Forward: GACAGTCAGCCGCATCTTCT, and Reverse: GCGCCCAATACGACCAAATC. GAPDH was utilized as internal control.

### CCK8 Viability Assay

Cells (3,000 cells/well) were seeded in 96-well plates, after overnight attachment, the medium was changed to 100 μl FBS-free medium with 10% CCK8 (MCE, USA) in each well and incubated for 2 h at 37°C, then the OD values at 450 nm were detected through microplate reader (BioTek, USA). These steps were repeated at 0, 24, 48, and 72 h, and the relative absorbance was calculated based on the OD values at 0 h.

### Clone Formation Assay

Cells (2,000 cells/well) were seeded in 6-well plates and cultured until visible clones appeared. Then we used methanol to fix clones 15 min, 1% crystal violet to stain clones 20 min, and counted the number of clones (>50 cells).

### Cell Apoptosis and Cell Cycle Assays

For cell apoptosis assay, cells were collected by EDTA-free trypsin, washed with PBS for three times, and resuspended in binding buffer. After incubation with PI and Annexin V-APC (BD Biosciences, USA) in dark for 15 min, the cell apoptosis was examined through flow cytometer (BD, Biosciences, USA) and analyzed by FlowJo 10.6.2. For cell cycle assay, cells were collected and fixed in 70% ethanol at 4°C overnight, then stained as the protocol of the cell cycle staining kit (MultiSciences, China). The cell cycle was examined using flow cytometer and analyzed by Modfit LT software.

### Wound Healing Assay

Cells were seeded in 6-well plates with serum-free DMEM and cultured to 100% density, and then the scratch wounds were created using 10 μl pipette tips. Images of wounds were captured at 0, 24,  and 48 h, the area of wounds was quantified by ImageJ software (40×).

### Transwell For Migration Assay

For transwell migration assay, 4 × 10^4^ cells were seeded on the upper transwell chambers in 200 μl serum-free culture medium, and 600 μl medium containing 20% FBS was added to the lower chambers. After 40 h incubation, the cells that migrated through membranes were fixed with methanol, stained with 1% crystal violet and counted under light microscope (200×).

### Western Blot

Total cellular protein was extracted with RIPA lysis buffer (Servicebio, China), denatured by mixing 5× loading buffer and boiling for 5 min. Then the denatured protein was subjected to SDS/polyacrylamide-gel electrophoresis and transferred to 0.45 µm polyvinylidene fluoride membranes. The membranes were blocked in 5% nonfat milk for 1 h at room temperature, and subsequently incubated with the following primary antibodies: H2AFY (Abcam, CAT# ab183041, 1:10,000), Cyclin B1 (Proteintech, CAT# 28603-1-AP, 1:1,000), Cyclin D1 (Proteintech, CAT# 26939-1-AP, 1:1,000), E-Cadherin (Cell Signaling Technology, CAT# 3195, 1:1,000), Vimentin (Cell Signaling Technology, CAT# 5741, 1:1,000), Bcl-2 (Cell Signaling Technology, CAT# 4223, 1:1,000), STAT3 (Cell Signaling Technology, CAT# 9139, 1:1,000), p-STAT3 (Cell Signaling Technology, CAT# 9145, 1:1,000), and α-Tublin (Proteintech, CAT# 11224-1-AP, 1:5,000) at 4°C overnight. Next, the membranes were washed with TBST three times, each for 10 min and incubated with secondary antibodies at room temperature for 1 h. Finally, the indicated proteins were visualized by West Pico plus Chemiluminescent Substrate (Thermo Fisher Scientific, USA).

### Statistical Analysis

All data of this study were statistically analyzed by R software 3.6.1 and Prism 8.0. The Wilcoxon test or Kruskal–Wallis test were used to examine the mRNA expression levels of *H2AFY* in different clinical subgroups, logistic regression was conducted to analyze the association of the *H2AFY* expression and clinicopathological characteristics. The Kaplan–Meier method and log-rank test were applied for comparing overall survival. Correlation analyses were performed by Spearman correlation test. For experimental data, Student’s t-test was used to determine the differences between two groups. *P* <0.05 was regarded statistically significant.

## Results

### High *H2AFY* Expression in HCC

**Figure 1:**
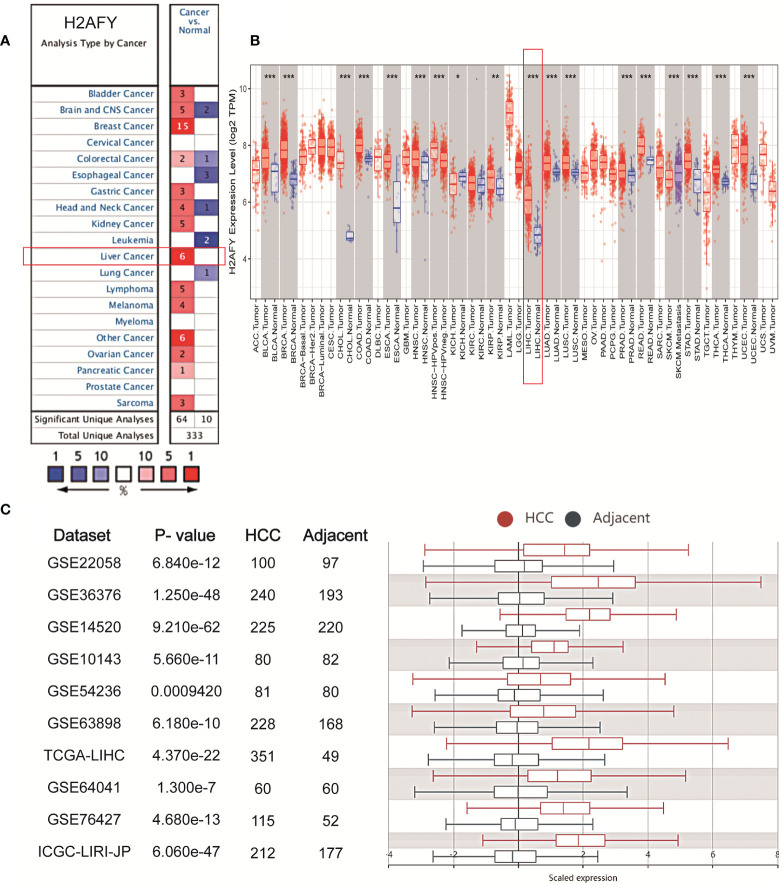
The elevated *H2AFY* expression in HCC. **(A)** Upregulated or downregulated *H2AFY* expression in different cancer types (Oncomine database, red color—upregulation, blue color—downregulation). **(B)** *H2AFY* expression levels in different tumor tissues and normal tissues (TIMER database). **(C)** Comparing the *H2AFY* expression between HCC and adjacent tissues in ten HCC cohorts (HCCDB database) *P < 0.05; **P < 0.01; ***P < 0.001.

We initially analyzed *H2AFY* mRNA expression levels in multiple public databases to examine *H2AFY* expression in HCC. Data from the Oncomine database revealed that *H2AFY* expression was dramatically higher in HCC tissues than normal tissues (FC >1.5, *P* <0.01), and ranked within the top 10% (**Figure 1A** and **Figure S1**). Meanwhile, the upregulation of *H2AFY* in HCC compared with normal tissues was also observed in TIMER database (**Figure 1B**). In the HCCDB database, analysis of ten HCC cohorts further verified that *H2AFY* was significantly upregulated in HCC (**Figure 1C**).

### Association With *H2AFY* Expression and Clinical Variables

**Figure 2:**
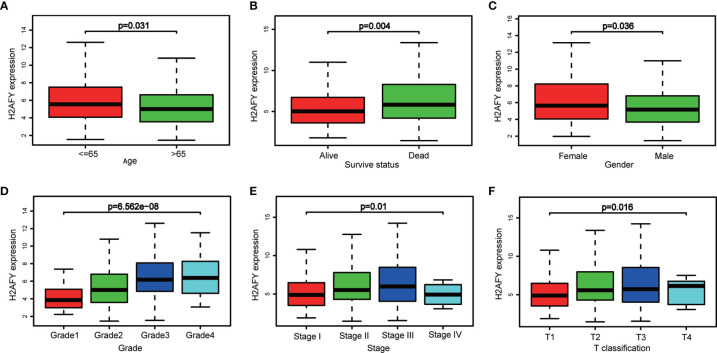
*H2AFY* expression in sub-groups of different clinical characteristics. Subgroup analyses of *H2AFY* expression based on **(A)** age, **(B)** survive status, **(C)** gender, **(D)** histological grade, **(E)** tumor stage, and **(F)** T classification.

Based on the *H2AFY* expression data and clinical information from TCGA, a total of 371 HCC patients were analyzed. The *H2AFY* expression in younger patients (≤65 years) was significantly higher than patients older than 65 years (*P* = 0.031, **Figure 2A**). Dead patients presented increased *H2AFY* expression compared to alive patients (*P* = 0.004). *H2AFY* expression was increased in dead patients compared to alive patients (*P* = 0.004, **Figure 2B**), increased in female compared to male (*P* = 0.004, **Figure 2C**).

Besides, *H2AFY* expression increased with the histological grade (*P* = 6.562e−08, **Figure 2D**) and T classification (*P* = 0.016, **Figure 2F**). As shown in **Figure 2E**, the *H2AFY* expression levels were significant different in the subgroups of clinical stage (*P* = 0.01). In logistic regression analysis, *H2AFY* expression as a dependent categorical variable (according to the median value), the results indicated that increased *H2AFY* expression in HCC was prominently associated with age (OR = 1.669 for ≤65 *vs.* >65, *P* = 0.018), survival status (OR = 1.624 for dead *vs.* alive, *P* = 0.027), histological grade (OR = 3.394 for G3–G4 *vs.* G1–G2, *P <*0.0001), T classification (OR = 1.590 for T2–T3 *vs.* T1, *P* = 0.030; OR = 4.304 for T4 *vs.* T1, *P* = 0.031), clinical stage (OR = 1.638 for stages II–III *vs.* stage I, *P* = 0.024; OR = 1.784 for stage III *vs.* stage I, *P <*0.031) (**Table 2**).

**Table 2:** Correlations between H2AFY expression and clinicopathological parameters by logistic regression.

Clinicopathological parameters	Total	Odds ratio in H2AFY expression	P-value
**Age**			
≤65 *vs* >65	370	1.669 (1.092–2.564)	**0.018**
**Gender**			
Female *vs* Male	371	1.198 (0.775–1.852)	0.417
**Survival status**			
Dead *vs* Alive	371	1.624 (1.058–2.505)	**0.027**
**Histological grade**			
G3–G4 *vs* G1–G2	366	3.394 (2.176–5.361)	**<0.001**
**T classification**			
T2 *vs* T1	275	1.531 (0.929–2.535)	0.095
T3 *vs* T1	261	1.578 (0.931–2.690)	0.091
T4 *vs* T1	194	4.304 (1.268–19.669)	**0.031**
T2–T3 *vs* T1	355	1.590 (1.047–2.423)	**0.030**
**TNM stage**			
II *vs* I	257	1.580 (0.939–2.670)	0.085
III *vs* I	256	1.784 (1.057–3.034)	**0.031**
IV *vs* I	176	1.966 (0.318–15.213)	0.465
II-III *vs* I	342	1.638 (1.070–2.517)	**0.023**

Bold values indicates P-value < 0.05.

### Genetic Alterations of *H2AFY* in HCC

**Figure 3:**
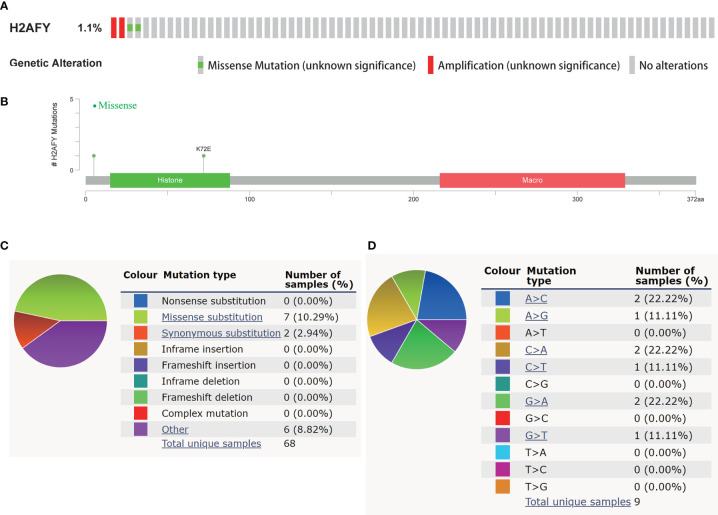
Genetic alterations of *H2AFY* in HCC. **(A)** OncoPrint of *H2AFY* alterations in TCGA-LIHC cohort (cBioPortal). **(B)** Schematic presentation of *H2AFY* mutations in TCGA-LIHC cohort (cBioPortal). **(C, D)** The mutation types of *H2AFY* in HCC (Catalogue of Somatic Mutations in Cancer (COSMIC) database).

In the cBioPortal database, we evaluated the alteration (copy-number alteration and mutation) types and frequency of *H2AFY* in HCC. The TCGA–Firehose Legacy dataset was selected for analysis, which included 360 samples with complete DNA sequencing data. The alteration frequency of *H2AFY* was 1.1% in HCC, which include amplification in two cases, missense mutation in two cases (**Figure 3A**). The detailed mutation landscapes were showed in **Figure 3B**. Since the alteration frequency was relatively low, we failed to explore the association between *H2AFY* genetic alteration and the survival of HCC patients. In addition, we further evaluated the mutation types of *H2AFY* in another database, COSMIC. The mutation types of *H2AFY* were clearly displayed in two pie charts (**Figures 3C, D**). Approximately seven (10.29%) of the 68 samples had missense substitutions, two (2.94%) of the 68 samples had synonymous substitutions, and seven (8.82%) of the 68 samples had other mutations (**Figure 3C**). The substitution mutations mainly included A > C (22.22%), C > A (22.22%), G > A (22.22%), followed by A > G (11.11%), C > T (11.11%), and G > T (11.11%) (**Figure 3D**).

### Prognostic Significance of *H2AFY* in HCC

**Figure 4:**
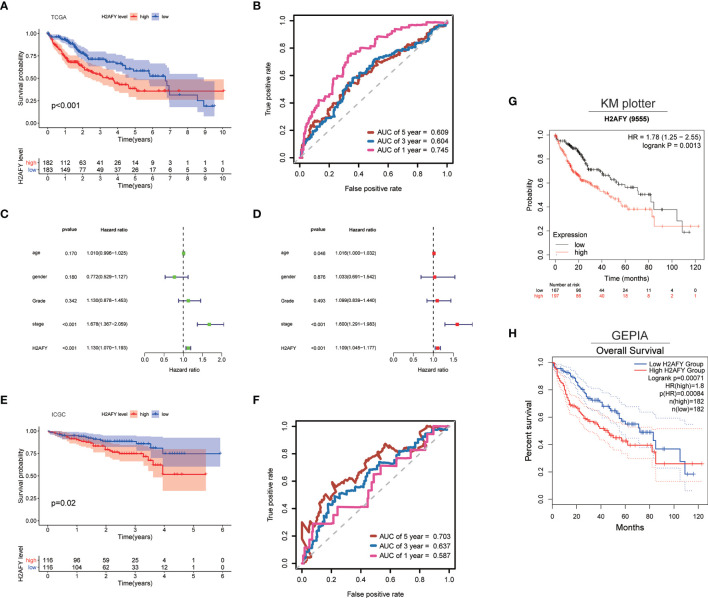
*H2AFY* is associated with overall survival of HCC patients. **(A)** Kaplan–Meier survival curves and **(B)** time-dependent ROC curves of *H2AFY* in TCGA-LIHC cohort. **(C)** univariate Cox analysis and **(D)** multivariate Cox analysis in TCGA-LIHC cohort. **(E)** Kaplan–Meier survival curves and **(F)** time-dependent ROC curves of *H2AFY* in ICGC cohort (LIRI-JP project). **(G)** Kaplan–Meier survival analyses of *H2AFY* in Kaplan–Meier Plotter and **(H)** GEPIA2.

Then, we explore the role of *H2AFY* in HCC patients’ survival outcomes in multiple databases. Based on the median *H2AFY* expression value, the HCC patients were split into high- and low-*H2AFY* expression groups. In the TCGA-LIHC cohort, Kaplan–Meier survival curves indicated that patients with high *H2AFY* expression tended to have poor overall survival (log-rank *P <*0.001, **Figure 4A**), time-dependent ROC curves indicated that *H2AFY* had moderate sensitivity and specificity for predicting survival (**Figure 4B**). Further univariate and multivariate Cox regression analyses revealed that *H2AFY* could function as a prognostic indicator independent of other clinical parameters for HCC patients (**Figures 4C, D**). In the ICGC cohort, the similar results were observed (**Figures 4E, F**). Besides, we verified the prognostic significance of *H2AFY* through K–M plotter and GEPIA online databases, the results also indicated that high *H2AFY* expression was associated poor survival (log-rank *P <*0.001, **Figures 4G, H**).

### *H2AFY* Co-Expression Networks in HCC

**Figure 5:**
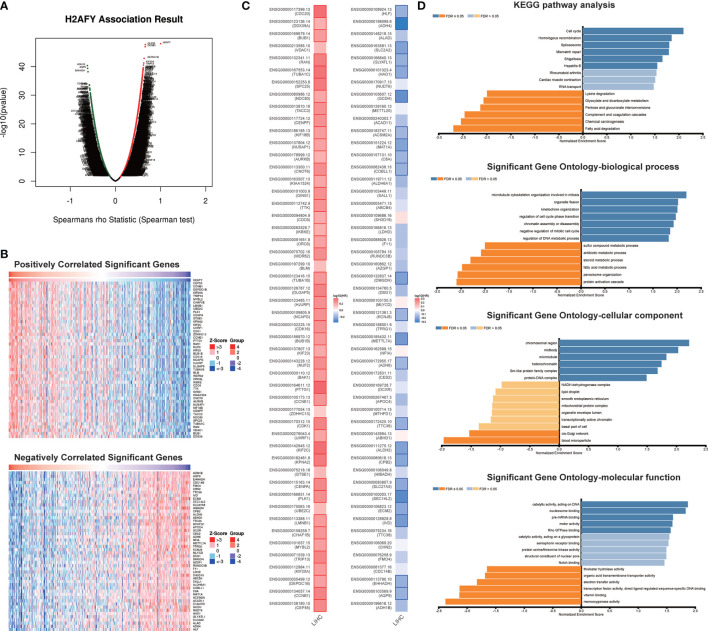
*H2AFY* co-expression networks in HCC (LinkedOmics). **(A)** Volcano plot of the global *H2AFY* highly correlated genes identified by Spearman test. **(B)** Heat maps of top 50 genes positively and negatively correlated with *H2AFY*. **(C)** Survival heatmaps of top 50 genes positively and negatively correlated with *H2AFY*. **(D)** Significantly enriched GO terms and KEGG pathways related to *H2AFY*.

The co-expression pattern of *H2AFY* was explored in TCGA-LIHC cohort through LinkedOmics (Table S1). As presented in **Figure 5A**, a total of 7,201 genes positively correlated with *H2AFY* and 2,928 genes negatively correlated with *H2AFY* were identified (FDR <0.01). The top 50 positively and negatively correlated genes were presented in heat maps (**Figure 5B**). *H2AFY* expression exhibited a strong positive correlation with the expression of *CEP55* (positive rank #1, r = 0.663, FDR = 2.14E−44), *CCNB1* (r = 0.659, FDR = 7.55E−44) and *DEPDC1B* (r = 0.637, FDR = 6.98E−40), etc. Remarkably, the top 50 positively correlated genes had high probability of being high-risk markers in HCC, of which 45/50 genes owned high hazard ratio (HR, *P <*0.05). Conversely, 23/50 genes were with low HR (*P <*0.05) in the top 50 negatively correlated genes (**Figure 5C**). The results of GO enrichment analysis by GSEA suggested that *H2AFY* co-expressed genes participate mainly in microtubule cytoskeleton organization involved in mitosis, organelle fission, kinetochore organization, chromosome segregation, cell cycle G2/M phase transition and regulation of cell cycle phase transition. (**Figure 5D** and **Table S2**). KEGG pathway analysis revealed enrichment in the cell cycle, homologous recombination, DNA replication, spliceosome, and mRNA surveillance pathway. (**Figure 5D** and **Table S3**). All these findings indicated the important roles of *H2AFY* and its co-expressed genes in cell cycle regulation for HCC progression.

### Regulators of *H2AFY* Networks in HCC

To determine the regulatory factors of *H2AFY* in HCC, we further analyzed the kinase, miRNA, and transcription factor targets’ enrichment of *H2AFY* co-expressed genes using GSEA. The top five most significant kinase-target networks were related mainly to *PLK1*, *CDK1*, *CHEK1*, *AURKB*, and *CDK2* (**Table 3** and **Table S4**). Interestingly, no significant miRNA targets were enriched for *H2AFY* co-expressed genes (**Table 3** and **Table S5**). The significantly enriched transcription factor-targets were associated primarily with E2F transcription factor family (**Table 3** and **Table S6**), including V$E2F_Q4, V$E2F_Q6, V$E2F_02, V$E2F1DP1_01, and V$E2F1DP2_01.

**Table 3:** The kinase, miRNA and transcription factor-target networks of H2AFY in HCC (LinkedOmics).

Enriched Category	Enriched Geneset	LeadingEdgeNum	FDR
**Kinase Target**	Kinase_PLK1	32	0.00E+00
	Kinase_CDK1	77	0.00E+00
	Kinase_CHEK1	34	0.00E+00
	Kinase_AURKB	24	2.65E−04
	Kinase_CDK2	90	5.31E−04
**miRNA Target**	GAGCCAG, MIR-149	43	2.56E−01
	TAGGTCA, MIR-192, MIR-215	7	3.79E−01
	GCAAGAC, MIR-431	15	4.35E−01
	ACACTCC, MIR-122A	22	4.73E−01
	GGGGCCC, MIR-296	12	4.75E−01
**Transcription Factor** **Target**	V$E2F_Q4	75	0.00E+00
	V$E2F_Q6	75	0.00E+00
	V$E2F_02	82	0.00E+00
	V$E2F1DP1_01	82	0.00E+00
	V$E2F1DP2_01	82	0.00E+00

### GSEA Between High- and Low-*H2AFY* Expression Groups

**Figure 6:**
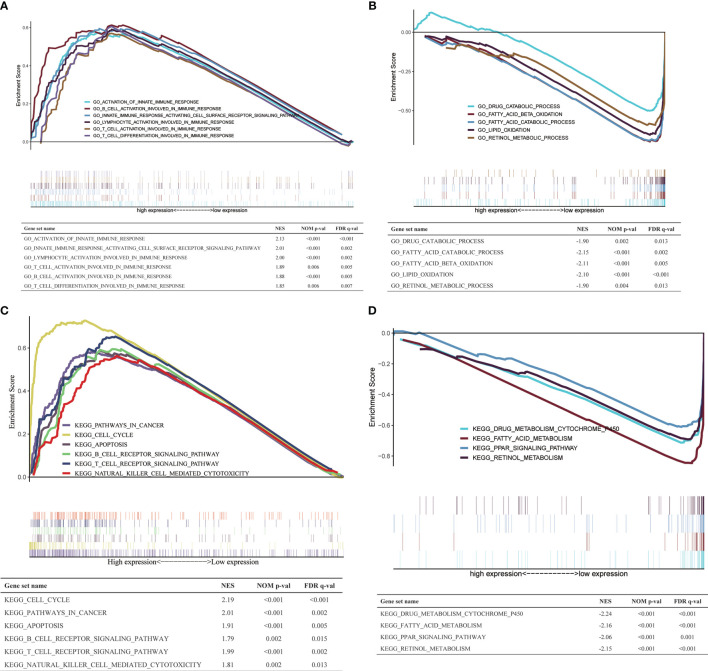
GSEA in TCGA-LIHC cohort. **(A, B)** The GO_BP annotations enriched in HCC patients with high/low *H2AFY* expression. **(C, D)** The KEGG pathways enriched in HCC patients with high/low *H2AFY* expression.

To explore the biological processes and signaling pathways that *H2AFY* may regulate, GSEA was performed between high- and low-*H2AFY* expression groups using TCGA-LIHC transcriptome data. We found some immune-related and cancer-related processes and pathways were significantly gathered in high-*H2AFY* expression group (**Figures 6A, C**), such as activation of innate immune response, innate immune response activating cell surface receptor signaling pathway, T-cell activation involved in immune response, B-cell activation involved in immune response, T-cell differentiation involved in immune response, lymphocyte activation of immune response, pathways in cancer, cell cycle, apoptosis and T-cell receptor signaling pathway, these results implied that *H2AFY* might be involved in immune response and impact immune infiltration. However, multiple metabolic processes like drug catabolic process, fatty acid catabolic process, lipid oxidation, drug metabolism cytochrome P450, and fatty acid metabolism were activated in low-*H2AFY* expression group (**Figures 6B, D**).

### Association Between *H2AFY* Expression and Immune Infiltration

**Figure 7:**
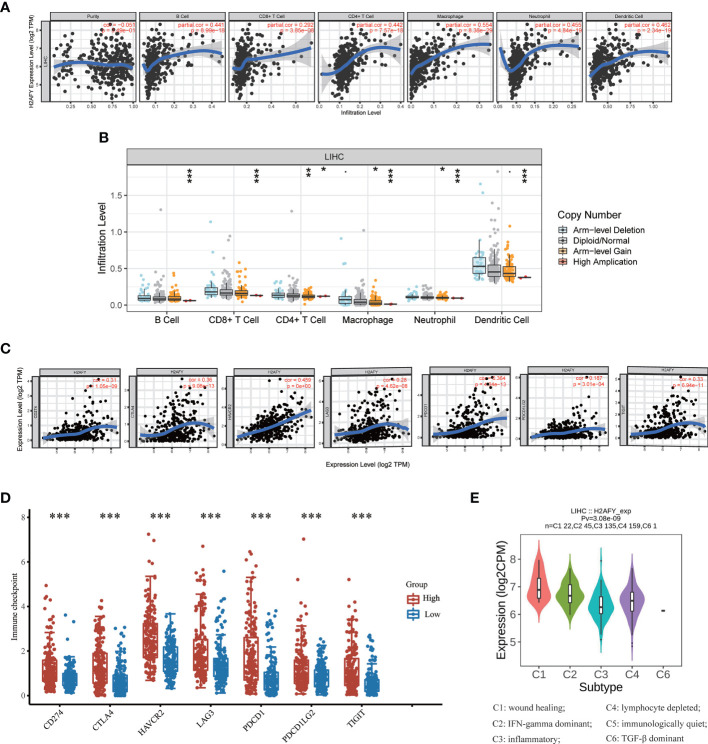
Correlations of *H2AFY* expression with immune infiltration in HCC. **(A)** Correlation analysis of *H2AFY* expression and abundance of immune cells in TIMER. **(B)** *H2AFY* copy number alterations affects the immune infiltration levels. **(C)** Correlations between the expression of *H2AFY* and several immune checkpoint genes. **(D)** The expression of several immune checkpoint genes between high- and low-*H2AFY* expression patients. **(E)** *H2AFY* expression in different immune subtypes of HCC (TISIDB database) *P < 0.05; **P < 0.01; ***P < 0.001.

Then, we investigate the correlation between *H2AFY* expression and immune infiltration levels in HCC through TIMER database. The results revealed that a significant positive correlation between *H2AFY* expression and infiltration level of B cells (r = 0.441, *P* = 8.99e−18), CD8+ T cells (r = 0.292, *P* = 3.85e−08), CD4+ T cells (r = 0.442, *P* = 7.57e−18), Macrophages (r = 0.554, *P* = 8.38e−29), Neutrophils (r = 0.455, *P* = 4.84e−19), and Dendritic cells (r = 0.462, *P* = 2.34e−19) in HCC (**Figure 7A**). Moreover, the copy number alterations of *H2AFY* could affect the infiltration level of six dominant immune cells, especially high amplification (**Figure 7B**). Next, we comprehensively explored the correlation between *H2AFY* expression and related marker genes of various tumor-infiltrating immune cells in HCC tissues. Correlation analysis was adjusted by tumor purity. In line with the above results, the *H2AFY* expression was significantly correlated with most selected immune cell marker genes (**Table 4**).

**Table 4:** Correlations between H2AFY and markers of immune infiltrates for HCC in TIMER.

Description	Gene markers	H2AFY			
		None		Purity	
		Cor	*P*	Cor	*P*
CD8+ T cell	CD8A	0.242	***	0.232	***
	CD8B	0.184	**	0.169	*
T cell (general)	CD3D	0.314	***	0.316	***
	CD3E	0.298	***	0.311	***
	CD2	0.305	***	0.32	***
B cell	CD19	0.323	***	0.311	***
	CD20 (MS4A1)	0.183	**	0.17	*
	CD79A	0.236	***	0.226	***
Monocyte	CD86	0.448	***	0.487	***
	CD16 (FCGR3A)	0.36	***	0.367	***
	CD115 (CSF1R)	0.339	***	0.366	***
TAM	CCL2	0.266	***	0.27	***
	CD68	0.37	***	0.369	***
	IL10	0.345	***	0.353	***
M1 Macrophage	NOS2	0.14	*	0.135	0.0121
	CXCL10	0.165	*	0.161	*
	IRF5	0.522	***	0.516	***
	COX2 (PTGS2)	0.326	***	0.356	***
M2 Macrophage	CD163	0.206	***	0.205	**
	ARG1	−0.16	*	-0.172	*
	MRC1	0.045	0.389	0.037	0.493
Neutrophils	CD11b (ITGAM)	0.441	***	0.468	***
	CD66b (CEACAM8)	0.076	0.147	0.081	0.132
	CCR7	0.216	***	0.223	***
	CD15(FUT4)	0.602	***	0.59	***
Natural killer cell	KIR2DL1	−0.008	0.872	−0.054	0.317
	KIR2DL3	0.15	*	0.144	*
	KIR2DL4	0.182	**	0.164	*
	KIR3DL1	0.037	0.476	0.02	0.708
	KIR3DL2	0.085	0.104	0.067	0.212
	KIR3DL3	0.033	0.539	0.061	0.241
	KIR2DS4	0.052	0.317	0.044	0.419
Dendritic cell	HLA-DPB1	0.33	***	0.337	***
	HLA-DQB1	0.264	***	0.255	***
	HLA-DRA	0.351	***	0.365	***
	HLA-DPA1	0.328	***	0.348	***
	BDCA-1 (CD1C)	0.285	***	0.284	***
	BDCA-4 (NRP1)	0.363	***	0.358	***
	CD11c (ITGAX)	0.492	***	0.529	***
Th1	T-bet (TBX21)	0.132	0.011	0.118	0.0279
	STAT4	0.377	***	0.377	***
	STAT1	0.468	***	0.458	***
	IFNG (IFN-γ)	0.234	***	0.241	***
	TNF(TNF-α)	0.343	***	0.37	***
Th2	GATA3	0.333	***	0.361	***
	STAT6	0.255	***	0.237	***
	STAT5A	0.339	***	0.339	***
	IL13	0.116	0.0253	0.103	0.0558
Tfh	BCL6	0.174	**	0.185	**
	IL21	0.115	0.0271	0.131	0.0152
	CD278 (ICOS)	0.339	***	0.35	***
	CXCL13	0.206	***	0.211	***
Th17	STAT3	0.319	***	0.318	***
	IL17A	0.09	0.0834	0.1	0.064
Treg	FOXP3	0.204	***	0.224	***
	CCR8	0.467	***	0.489	***
	STAT5B	0.281	***	0.303	***
	TGFB1	0.435	***	0.446	***
T cell exhaustion	PDCD1	0.364	***	0.358	***
	CTLA4	0.36	***	0.369	***
	LAG3	0.28	***	0.255	***
	HAVCR2 (TIM3)	0.459	***	0.503	***
	GZMB	0.091	0.0801	0.068	0.206

TAM, tumor-associated macrophage; Th, T helper cell; Tfh, Follicular helper T cell; Treg, regulatory T cell; None, correlation without adjustment. Purity, correlation adjusted by purity; Cor, R value of Spearman’s correlation. *P < 0.01; **P < 0.001; ***P < 0.0001.

Based on reported studies, immune checkpoint molecules expression level might be tightly linked to the efficacy of immune checkpoint inhibitors. Therefore, we further investigated the correlation of *H2AFY* and seven key immune checkpoint molecules to clarify the role of *H2AFY* in immune checkpoint blockade therapy for HCC patients. The results in TIMER database pointed out that *H2AFY* had a close correlation with CD274 (PD-L1), CTLA4, HAVCR2, LAG3, PDCD1, PDCD1LG2, and TIGIT (*P <*0.001, **Figure 7C**), and the correlation was validated in GEPIA2 database (**Figure S3**). Additionally, compared with low-*H2AFY* expression group, these immune checkpoint genes expression levels were also higher in high-*H2AFY* expression group (*P <*0.001, **Figure 7D**). We further explored the relationship of *H2AFY* expression and immune subtypes, as displayed in **Figure 7E**, *H2AFY* expression was significantly differently distributed between six immune subtypes.

**Figure 8:**
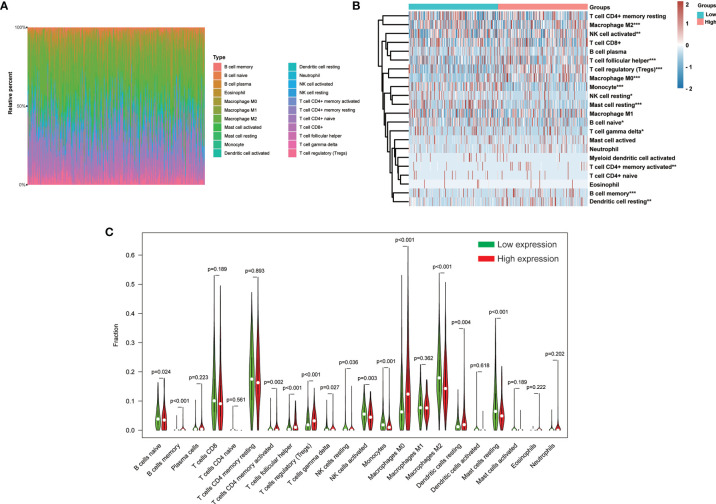
Correlation of *H2AFY* expression and 22 immune cell types in HCC based on CIBERSORT. **(A)** The relative fraction of 22 immune cell types in TCGA-LIHC cohort. **(B)** The heat map showing relative immune cell fraction of HCC patients **(C)** Violin plots showing the difference of 22 immune cell types between high- and low-*H2AFY* expression patients *P < 0.05; **P < 0.01; ***P < 0.001.

The CIBERSORT method was further employed to understand the association between *H2AFY* expression with 22 immune cell types in TCGA-LIHC cohort. **Figure 8A** summarized the relative fraction of these immune cells in each HCC patient. Within and between groups, the relative fraction of each immune cell type varied in HCC (**Figure 8B**). We found that high-*H2AFY* expression patients presented significantly higher B cell memory, T cells CD4 memory active, T cells regulatory (Tregs), T cells follicular helper, T cells gamma delta, macrophages M0 and Dendritic cells resting proportions (*P <*0.05), and lower B cell naive, NK cell resting, NK cell active, Monocytes, macrophages M2, Mast cells resting (*P <*0.05, **Figure 8C**). All these findings suggested that *H2AFY* was closely related to immune infiltration, and *H2AFY* might be able to predict the response of HCC patients to immune checkpoint blockade therapy.

### Effects of *H2AFY* Knockdown on Cell Proliferation and Apoptosis in HCC Cells *In Vitro*

**Figure 9:**
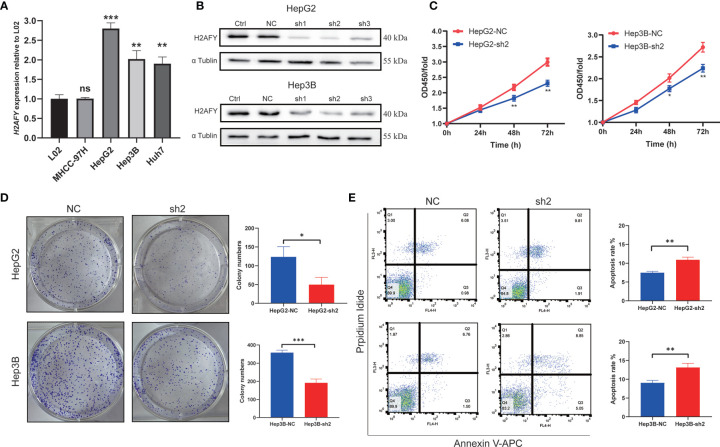
Effects of H2AFY knockdown on cell proliferation and apoptosis in HCC cells. **(A)** *H2AFY* mRNA expression in normal liver cell line (L02) and several HCC cell lines. **(B)** Evaluation of *H2AFY* expression in HepG2 and Hep3B cells after shRNA transfection. **(C)** The effect of *H2AFY* knockdown on cell proliferation in HepG2 and Hep3B cells examined by CCK8 assay and **(D)** colony formation assay. **(E)** The effect of *H2AFY* knockdown on cell apoptosis in HepG2 and Hep3B cells examined by flow cytometry ns, no significance; *P < 0.05; **P < 0.01; ***P < 0.001.

The qRT-PCR assay was applied to detect *H2AFY* mRNA expression in different HCC cell lines. We found that *H2AFY* was also significantly overexpressed in HCC cell lines than normal liver cell line (**Figure 9A**), and selected HepG2 and Hep3B cell lines with relative higher *H2AFY* expression levels for subsequent experiments *in vitro*. *H2AFY* was knockdown in HepG2 and Hep3B cells by lentivirus transfection with shRNAs. Western blot assay examined the knockdown efficiency of shRNAs, the results showed that both shRNAs effectively inhibited H2AFY protein expression compared with negative control (NC) shRNA (**Figure 9B**). ShRNA-2 targeting *H2AFY* was used for the subsequent investigation.

The CCK8 assays were performed to explore the effect of *H2AFY* knockdown HCC cell proliferation, and the results revealed that the proliferation of HepG2 and Hep3B cells was significantly decreased after *H2AFY* knockdown (**Figure 9C**). Further colony formation assays suggested that *H2AFY* downregulation dramatically suppressed colony formation in both HepG2 and Hep3B cell lines (*P <*0.05, **Figure 9D**). In addition, the cell apoptosis was detected by flow cytometry, and *H2AFY* knockdown markedly enhanced the cell apoptosis in HepG2 and Hep3B cells (**Figure 9E**).

### Effects of *H2AFY* Knockdown on Cell Cycle, Migration and anti-T-Cells Killing Ability in HCC Cells *In Vitro*

**Figure 10:**
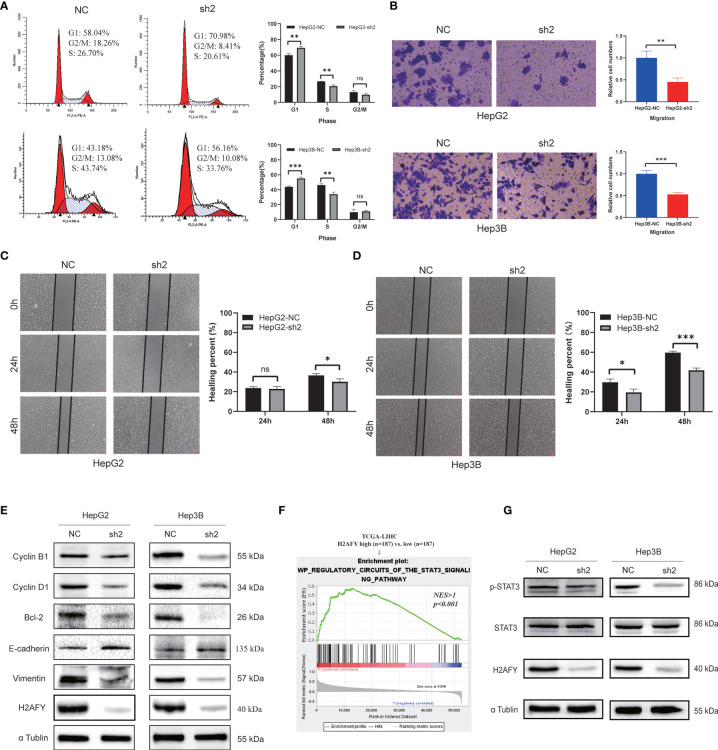
Effects of *H2AFY* knockdown on cell cycle and migration in HCC cells. **(A)** Cell cycle detected by flow cytometry in HepG2 and Hep3B cells after *H2AFY* knockdown. **(B–D)** Representative images of transwell (200×) and wound healing assays (40×) in HepG2 and Hep3B cells, and the quantitative result following *H2AFY* knockdown. **(E)** Western blot analysis of cell cycle, apoptosis, EMT related molecular markers in HepG2 and Hep3B cells transfected with *H2AFY*-shRNA or the negative control. **(F)** STAT3 signaling pathway was significantly enriched in high-*H2AFY* expression patients. **(G)** Evaluation of p-STAT3 and STAT3 expression in HepG2 and Hep3B cells after transfecting *H2AFY*-shRNA ns, no significance; *P < 0.05; **P < 0.01; ***P < 0.001.

The preceding results indicated that *H2AFY* may be involved in the cell cycle process, we therefore performed cell cycle analysis using flow cytometry. As showcased in **Figure 10A**, the *H2AFY* downregulation resulted in G1/S phase arrest, the percentage of cells in G1 phase significantly increased and the proportion of cells in S phase decreased in both HepG2 and Hep3B cells (*P <*0.05). Subsequently, to investigate the impacts of the *H2AFY* knockdown on HCC cell migration ability, wound-healing and transwell assays was performed to measure the migration ability following *H2AFY* knockdown. These assays revealed that *H2AFY* knockdown drastically decreased the migration ability of HepG2 and Hep3B cells, compared to NC group (**Figures 10B–D**). We next conducted T-cells-mediated cancer killing assay to detect the effect of *H2AFY* knockdown in HCC cells on anti-T-cells killing ability (36). We found that *H2AFY* knockdown significantly reduced the survival of HCC cells than those with NC after co-culturing with activated Jurkat cells (**Figure S4**). Besides, we also detected the expression of cell cycle, apoptosis and EMT related molecular markers in HCC cells with *H2AFY* knockdown. As expected, we observed that the expression levels of Cyclin B1, Cyclin D1, Bcl-2, and Vimentin showed significantly downward trends after suppressing *H2AFY* in HepG2 and Hep3B cells. Conversely, the expression of E-cadherin was significantly upregulated in HCC cells transfected with *H2AFY*-shRNA (**Figure 10E**).

Moreover, we noticed that *H2AFY* expression was positively correlated with STAT3 signaling pathway among the various pathways revealed by GSEA (**Figure 10F**). Some previous studies have demonstrated that the STAT3 signaling pathway was activated in HCC and associated with multiple malignant biological behaviors of HCC (37, 38). Therefore, we examined whether *H2AFY* might affect STAT3 signaling pathway activation in HCC cells. Western blot results indicated that *H2AFY* knockdown decreased the expression of phosphorylated STAT3 and inhibited STAT3 signaling pathway activation (**Figure 10G**). Overall, these results illustrated that *H2AFY* knockdown inhibited HCC progression at least partly *via* regulating STAT3 signaling.

## Discussion

The *H2AFY* gene encodes for macroH2A1, a histone variant of the histone H2A that have been reported to be dysregulated in various human cancers (39, 40). Several prior published studies indicate that the decreased expression level of *H2AFY* was inversely correlated with cell proliferation and function as a marker for poor prognosis in lung cancer and colon cancer (14, 41). By contrast, *H2AFY* promoted cancer cell proliferation by interacting with HER2 and higher expression of *H2AFY* was associated with worse prognosis in triple-negative breast cancer (18, 19). Additionally, the expression *H2AFY* was reduced in metastatic cutaneous melanomas compared to benign nevi, and the loss of *H2AFY* promoted proliferation and migration of cutaneous melanoma cells through regulation of *CDK8* (17, 42). Interestingly, however, contrary to cutaneous melanoma, the metastatic uveal melanoma has been reported to have a higher *H2AFY* expression level than non-metastatic uveal melanoma, and *H2AFY* silencing decreases the invasiveness of uveal melanoma cells by reducing mitochondrial metabolism (43). These proofs of evidence suggest that *H2AFY* exhibits either oncogenic function or tumor suppressor function in different tumor types, which seems to depend on the context and genetic background of the specific tumor studied. To understand more details about the potential functions and regulatory network of *H2AFY* in HCC, we conducted a series of bioinformatics analyses and experiments *in vitro* to provide new insights for HCC.

In this study, we first investigated the expression of *H2AFY* in HCC, and found that *H2AFY* mRNA expression was prominently upregulated in HCC compared to normal tissues across various public databases. Clinical association analyses demonstrated that increased *H2AFY* expression was correlated with higher histological grade, more advanced clinical stage and larger tumor size. Besides, we also found several genetic alterations of *H2AFY* in HCC, mainly amplification and missense mutation. Kaplan–Meier and Cox regression analyses further revealed that high *H2AFY* expression was an independent risk factor to predict poor OS for HCC patients. Therefore, our findings demonstrated that *H2AFY* could act as a potential diagnostic or prognostic biomarker for HCC and deserves further clinical verification.

Next, we explored the co-expression network of *H2AFY* and identified multiple genes co-expressed with *H2AFY*, which were further used for GO and KEGG enrichment analyses. The result displayed that the enrichment primarily associated with cell cycle, chromatin, mitosis, and spliceosome, and *H2AFY* may affect cell cycle and mitosis progression through these factors. The regulators responsible for *H2AFY* dysregulation were explored in HCC, and the kinase networks related to *H2AFY* were found, namely, *PLK1*, *CDK1*, *CHEK1*, *AURKB*, and *CDK2*. These kinases could regulate mitosis, cell cycle, and genome stability. All these kinase genes, except *CDK2*, were found to be significantly highly expressed in HCC and related to the poor OS of patients with HCC. *PKL1* is a key regulator for the cell cycle progression, the main function of *PLK1* is to control mitotic entry and maintain genomic stability in mitosis and DNA damage response (44). Studies have revealed the role of *PLK1* in most human cancers, and established a causal association between *PLK1* and hepatocarcinogenesis (45). The activity of *CDK1* is often enhanced in cancer cells, it therefore has been considered as an appealing specific anti-cancer target (46). Multiple inhibitors targeting *CDK1* have been developed and have entered early clinical trials for some malignancies (47). *AURKB* plays a crucial role for the cell cycle transition from G2 to M phase (48). In HCC, *H2AFY* may regulate cell cycle progression, mitosis and chromatin assembly *via* these interacted kinases. We also identified that the main transcription factor targets of *H2AFY* were E2F family members. E2F transcription factors are involved in cell cycle regulation and DNA synthesis, and the oncogenic role of the E2Fs has been reported in previous studies (49, 50). However, no miRNA targets significantly associated with *H2AFY* were identified, possibly because *H2AFY* participates in mRNA splicesome. Our results demonstrated that E2F1 is a pivotal regulator of *H2AFY*, and *H2AFY* might regulate the cell cycle and proliferation of HCC through this factor.

Furthermore, we observed many immune-related pathways significantly gathered in high-*H2AFY* expression phenotype, such as activation of innate immune response, lymphocyte activation of immune response, B-cell receptor signaling pathway, and T-cell receptor signaling pathway. Previous studies have manifested that infiltrating immune cells in tumor microenvironment play a major role in tumor development and metastasis, thus affecting the prognosis of cancer patients (51, 52). Recently, immunotherapeutic strategies especially immune checkpoint blockade therapy, have been considered as promising options for the treatment of various malignancies, including HCC (53, 54). Therefore, the exploration of novel immune biomarkers or immunotherapeutic targets for HCC is clinically significant. Here, we revealed a correlation between *H2AFY* expression and immune infiltration in HCC. *H2AFY* expression showed significantly positive correlations with the expression of various immune cell marker genes and immune checkpoint molecules such as PD-L1 and CTLA4. Additionally, the high-*H2AFY* expression patients have higher expression of these immune checkpoint genes than low-*H2AFY* expression patients. The upregulated PD-L1 expression is found to be associated with poor prognosis of patients with HCC, and it was an appealing immunotherapeutic target for HCC. Together, these results suggested that *H2AFY* may exert a vital role in modulating tumor immunity, and serve as a potential biomarker related to immune infiltration in HCC.

Furthermore, a series of functional assays *in vitro* verified the role of *H2AFY* in HCC by downregulating the *H2AFY* expression. The results showed that *H2AFY* knockdown suppressed the cell proliferation, migration and promoted apoptosis of HCC cells *in vitro*. In addition, we observed an increased proportion of HCC cells in G1 phase and a decreased proportion in S phase after *H2AFY* knockdown. The STAT3 signaling was activated in many cancers, its activation has been found to promote HCC progression (55–57). *H2AFY* knockdown also downregulated the phosphorylated STAT3 expression in HCC cells, and the result showed that *H2AFY* knockdown inhibited HCC malignant progression at least partly *via* regulating STAT3 signaling.

Nonetheless, several limitations in our study should be recognized. First, our finding is based on retrospective data from public databases, more prospective data and larger HCC cohorts were required to confirm its clinical suitability. Second, the role of *H2AFY* in tumor immune infiltration needs to be further validated *in vitro or in vivo*. Finally, we have demonstrated that *H2AFY* could regulate STAT3 signaling in HCC, but the detailed regulatory mechanism requires more functional studies to elucidate in future. Our findings should be taken with these limitations for interpretation.

In general, our study provided multi-level evidence for *H2AFY* as a potential biomarker and prognostic predictor for HCC. These results revealed that *H2AFY* was upregulated in HCC and its high expression was associated with poor prognosis of HCC patients. Moreover, *H2AFY* has a significantly positive correlation with immune infiltration in HCC.

## Data Availability Statement

The original contributions presented in the study are included in the article/**Supplementary Material**. Further inquiries can be directed to the corresponding author.

## Author Contributions

YH designed the study and wrote the manuscript. SH, LM, XW, LX, WQ, LL, and YW participated in data preparation and figure preparation. SH revised the manuscript. XY reviewed the manuscript. All authors contributed to the article and approved the submitted version.

## Funding

This study was supported by National Natural Science Foundation of China (2018, 81773360).

## Conflict of Interest

The authors declare that the research was conducted in the absence of any commercial or financial relationships that could be construed as a potential conflict of interest.

## Publisher’s Note

All claims expressed in this article are solely those of the authors and do not necessarily represent those of their affiliated organizations, or those of the publisher, the editors and the reviewers. Any product that may be evaluated in this article, or claim that may be made by its manufacturer, is not guaranteed or endorsed by the publisher.
