# Supplementary material for: Exploring the Prognostic Value, Immune Implication and Biological Function of H2AFY Gene in Hepatocellular Carcinoma
Source: Front Immunol. 2021 Nov 24;12:723293. doi: 10.3389/fimmu.2021.723293 (PMC8651705; doi:10.3389/fimmu.2021.723293)
Supplement: Supplementary file 1 [file DataSheet_1.pdf]

Supplementary figures

Figure S1.

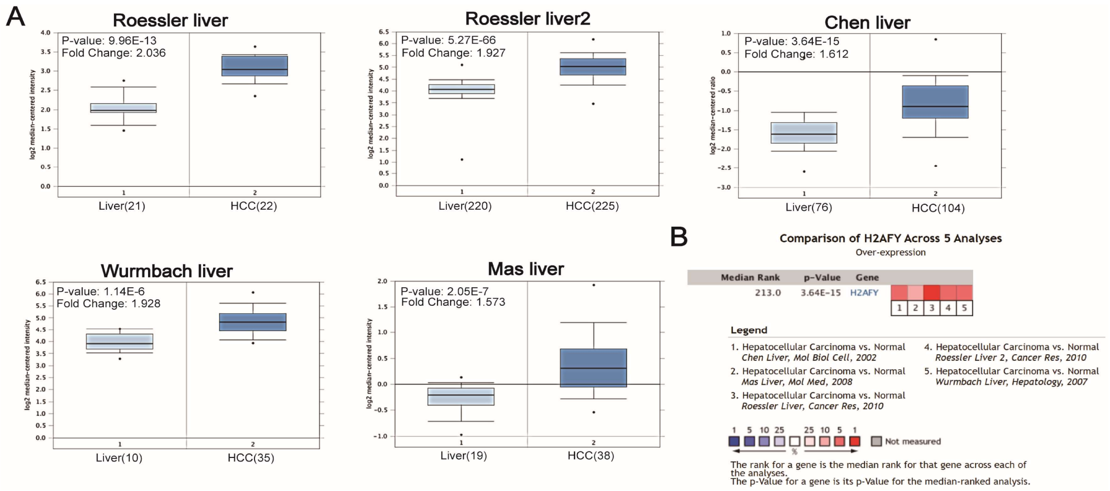

Supplementary Figure 1. H2AFY mRNA levels in the Roessler Liver, Roessler Liver 2, Chen liver, Wurmbach Liver and Mas liver datasets in Oncomine.

Figure S2.

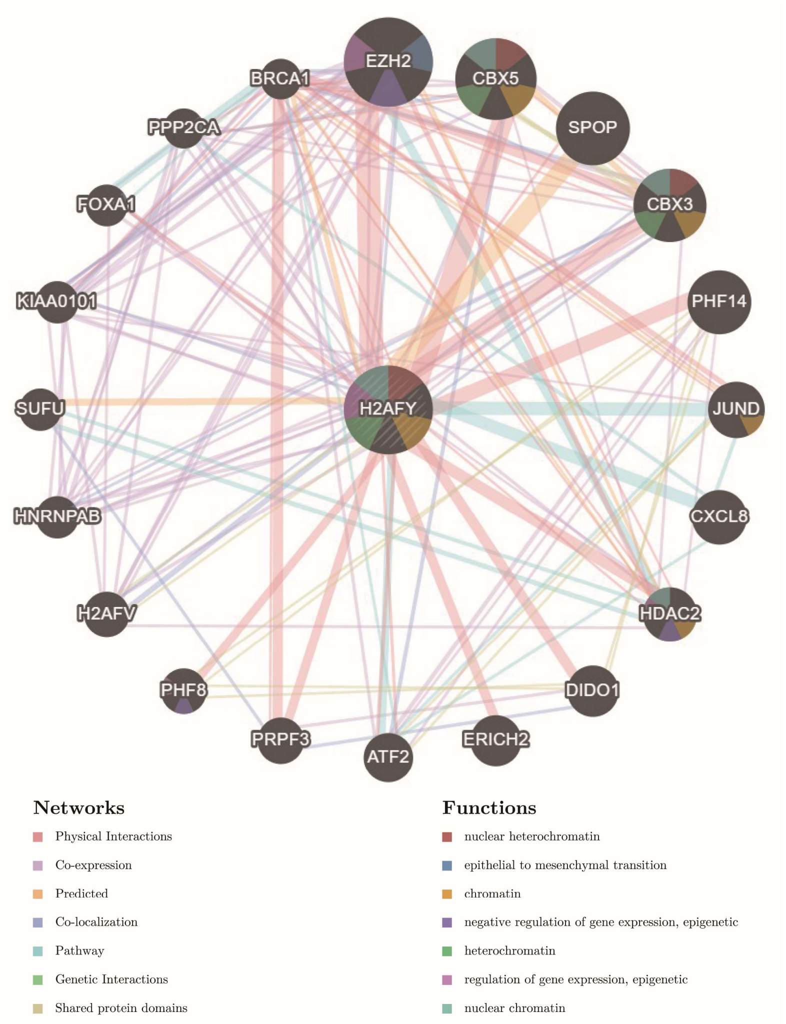

Supplementary Figure 2. Protein-Protein Interaction Network of H2AFY through GeneMANIA.

Figure S3.

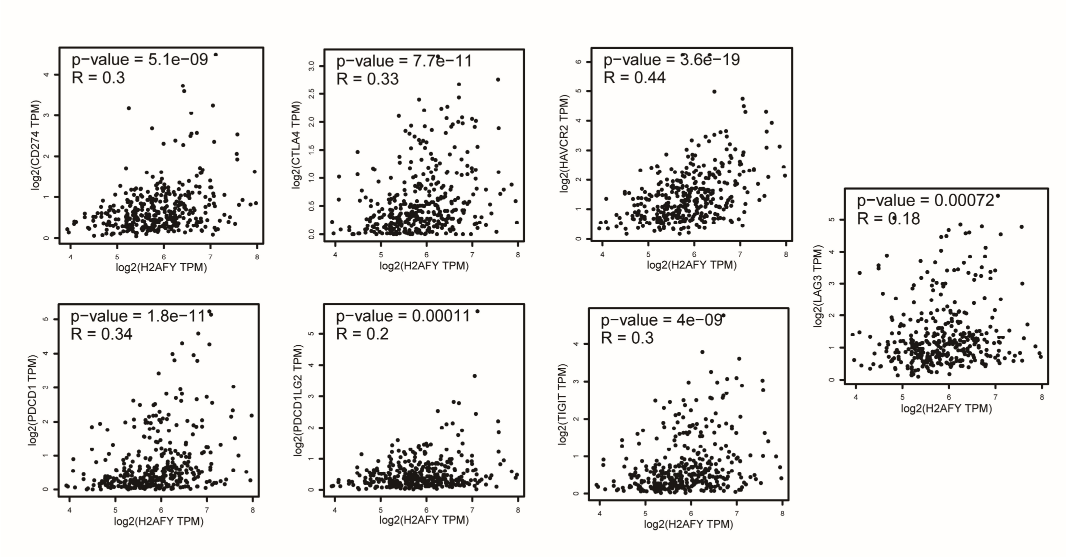

Supplementary Figure 3. Correlations between H2AFY expression and immune checkpoint molecules determined by GEPIA2.

Figure S4.

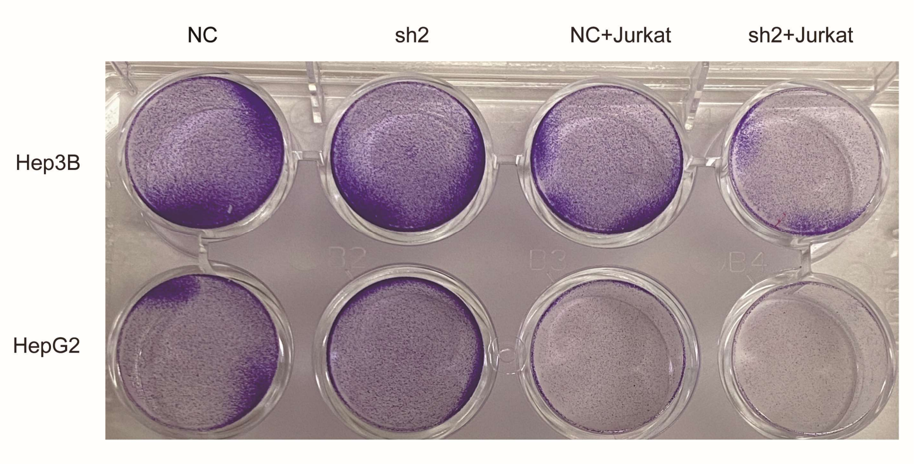

Supplementary Figure 4. Knockdown of H2AFY in HCC cells increased the cytotoxicity of T-cells.
