# Supplementary material for: Exploring the Prognostic Value, Immune Implication and Biological Function of H2AFY Gene in Hepatocellular Carcinoma
Source: Front Immunol. 2021 Nov 24;12:723293. doi: 10.3389/fimmu.2021.723293 (PMC8651705; doi:10.3389/fimmu.2021.723293)
Supplement: Supplementary file 2 [file Table_1.pdf]

Supplementary Table 1. H2AFY co-expressed genes.

Query	Correlation cofficient	P-value	FDR (BH)
A1BG	-0.375836855	6.83E-14	8.43E-13
A1CF	-0.235840196	4.39E-06	1.55E-05
A2BP1	0.134288601	0.00960897	0.017807433
A2LD1	-0.21704193	2.48E-05	7.60E-05
A2ML1	0.070879105	0.173092207	0.231354776
A2M	-0.002857096	0.956261179	0.965664802
A4GALT	0.080275642	0.122711391	0.171808021
A4GNT	0.33546332	3.29E-11	2.67E-10
AAA1	0.268664344	1.49E-07	6.73E-07
AAAS	0.115327407	0.026331782	0.0440307
AACSL	0.240217181	2.87E-06	1.04E-05
AACS	0.499308374	8.69E-25	5.44E-23
AADACL2	-0.04387417	0.399435233	0.475617041
AADACL3	0.095368274	0.066519292	0.100408951
AADACL4	0.000625756	0.990415854	0.992558209
AADAC	-0.484944816	2.79E-23	1.38E-21
AADAT	-0.365730702	3.48E-13	3.86E-12
AAGAB	0.248220909	1.29E-06	4.99E-06
AAK1	0.238201317	3.49E-06	1.25E-05
AAMP	0.028032714	0.590416937	0.658029999
AANAT	0.149381722	0.003929292	0.00792221
AARS2	0.050015989	0.336689719	0.410673641
AARSD1	0.109605901	0.0348242	0.056463586
AARS	-0.042286107	0.416732674	0.49269065
AASDHPPT	-0.136469011	0.008487555	0.015899302
AASDH	-0.234355058	5.06E-06	1.77E-05
AASS	-0.487227931	1.63E-23	8.33E-22
AATF	0.267608136	1.67E-07	7.49E-07
AATK	0.334342274	3.86E-11	3.10E-10
ABAT	-0.484030264	3.47E-23	1.68E-21
ABCA10	-0.11411758	0.027961441	0.046432261
ABCA11P	0.206309334	6.24E-05	0.000178975
ABCA12	0.216030246	2.71E-05	8.26E-05
ABCA13	-0.01963146	0.706256133	0.76103606
ABCA17P	0.083163084	0.10977617	0.155877467
ABCA1	-0.093518083	0.071994688	0.107613908
ABCA2	-0.039935317	0.443129786	0.518807687
ABCA3	0.152420724	0.003249031	0.006669468
ABCA4	0.013741583	0.791933364	0.833690348
ABCA5	-0.160771604	0.001893409	0.004094268
ABCA6	-0.518794462	5.98E-27	5.27E-25
ABCA7	0.118641754	0.02227954	0.037900521
ABCA8	-0.427403031	6.59E-18	1.50E-16
ABCA9	-0.386244367	1.20E-14	1.65E-13
ABCB10	-0.034650692	0.505815725	0.578930304
ABCB11	-0.431175236	3.14E-18	7.51E-17
ABCB1	-0.20976216	4.66E-05	0.000136701
ABCB4	-0.533844444	1.02E-28	1.18E-26
ABCB5	0.294714584	7.19E-09	4.07E-08
ABCB6	-0.102193605	0.049195001	0.076964254
ABCB7	-0.182792876	0.000401921	0.00098926
ABCB8	-0.160652117	0.001908444	0.004122788
ABCB9	0.347360338	5.84E-12	5.32E-11
ABCC10	0.379731883	3.59E-14	4.61E-13
ABCC11	-0.233157322	5.67E-06	1.97E-05
ABCC12	-0.12713538	0.014266262	0.025326365

ABCC13	0.137320098	0.008082482	0.015223523
ABCC1	0.426724216	7.53E-18	1.70E-16
ABCC2	-0.291521487	1.06E-08	5.83E-08
ABCC3	0.041641635	0.423876025	0.500234475
ABCC4	0.39058392	5.71E-15	8.33E-14
ABCC5	0.400810529	9.48E-16	1.56E-14
ABCC6P1	-0.258958893	4.25E-07	1.78E-06
ABCC6P2	-0.066056077	0.204290149	0.267385083
ABCC6	-0.432870334	2.25E-18	5.50E-17
ABCC8	0.204560641	7.22E-05	0.000204589
ABCC9	-0.475916223	2.28E-22	9.88E-21
ABCD1	0.193762045	0.000173221	0.000457195
ABCD2	0.104179287	0.044928524	0.070971573
ABCD3	-0.289093882	1.42E-08	7.66E-08
ABCD4	-0.189441727	0.000242667	0.00062267
ABCE1	0.030402183	0.55939102	0.629828637
ABCF1	0.110069075	0.034059269	0.055358469
ABCF2	0.150035511	0.003772918	0.007636297
ABCF3	-0.0167029	0.748470107	0.796685379
ABCG1	0.255802933	5.92E-07	2.42E-06
ABCG2	-0.50054161	6.40E-25	4.10E-23
ABCG4	0.166920274	0.00125126	0.002802744
ABCG5	-0.281610243	3.43E-08	1.73E-07
ABCG8	-0.355836539	1.63E-12	1.61E-11
ABHD10	-0.331853399	5.49E-11	4.30E-10
ABHD11	0.188631372	0.000258296	0.000659208
ABHD12B	-0.129472479	0.012563289	0.02263801
ABHD12	0.31252372	7.55E-10	4.96E-09
ABHD13	-0.079003847	0.128772175	0.179035472
ABHD14A	-0.033987652	0.513996214	0.586774215
ABHD14B	-0.419159092	3.23E-17	6.70E-16
ABHD15	-0.318683527	3.34E-10	2.31E-09
ABHD1	-0.555561894	1.97E-31	3.78E-29
ABHD2	-0.245556951	1.69E-06	6.38E-06
ABHD3	0.348592546	4.86E-12	4.48E-11
ABHD4	-0.000390448	0.994019767	0.995468848
ABHD5	-0.081805263	0.115718604	0.163210339
ABHD6	-0.437499703	8.88E-19	2.32E-17
ABHD8	0.007653978	0.88318673	0.907793109
ABI1	0.173159857	0.000809939	0.001884781
ABI2	0.410841633	1.53E-16	2.87E-15
ABI3BP	0.172212307	0.000866068	0.002002066
ABI3	0.19598405	0.00014523	0.000388881
ABL1	0.227481891	9.65E-06	3.20E-05
ABL2	0.15002837	0.003774595	0.00763814
ABLIM1	-0.202125103	8.83E-05	0.000246378
ABLIM2	0.170081363	0.001005661	0.002294338
ABLIM3	-0.09577418	0.065364941	0.098868754
ABO	-0.055137994	0.289483706	0.361075281
ABP1	-0.081169652	0.118585499	0.166746211
ABRA	0.149963146	0.003789945	0.007662974
ABR	0.381443257	2.70E-14	3.53E-13
ABT1	0.04722341	0.364394197	0.439994012
ABTB1	-0.042400666	0.415470346	0.491479143
ABTB2	-0.3342349	3.92E-11	3.14E-10
ACAA1	-0.500735512	6.10E-25	3.92E-23
ACAA2	-0.399556866	1.19E-15	1.93E-14
ACACA	0.110714723	0.033016879	0.053861961

ACACB	-0.240085013	2.91E-06	1.06E-05
ACAD10	-0.21106441	4.17E-05	0.000123529
ACAD11	-0.528511245	4.42E-28	4.71E-26
ACAD8	-0.30914826	1.17E-09	7.47E-09
ACAD9	-0.047719025	0.359376426	0.434831288
ACADL	-0.39122079	5.12E-15	7.53E-14
ACADM	-0.416123255	5.73E-17	1.14E-15
ACADSB	-0.388983084	7.52E-15	1.08E-13
ACADS	-0.244411326	1.90E-06	7.10E-06
ACADVL	-0.247290159	1.42E-06	5.43E-06
ACAN	0.37070086	1.57E-13	1.84E-12
ACAP1	0.230984642	6.96E-06	2.37E-05
ACAP2	0.0032036	0.950962909	0.96197233
ACAP3	0.063394322	0.223160889	0.288689041
ACAT1	-0.455455594	2.12E-20	6.81E-19
ACAT2	-0.042521929	0.414136591	0.490046874
ACBD3	0.125130299	0.015885662	0.027897933
ACBD4	-0.477972622	1.42E-22	6.31E-21
ACBD5	-0.210734709	4.29E-05	0.000126825
ACBD6	0.372775802	1.13E-13	1.35E-12
ACBD7	0.208878701	5.02E-05	0.000146511
ACCN1	0.033571175	0.51916947	0.591628048
ACCN2	0.132807237	0.010444088	0.019173159
ACCN3	0.08624024	0.097198393	0.1401655
ACCN4	0.233070309	5.71E-06	1.98E-05
ACCN5	-0.085802285	0.098915102	0.1423487
ACCSL	0.02268596	0.663167625	0.724321569
ACCS	-0.140801891	0.006599028	0.012670185
ACD	0.251651399	9.11E-07	3.61E-06
ACE2	-0.228606895	8.69E-06	2.90E-05
ACER1	-0.277154644	5.73E-08	2.78E-07
ACER2	-0.116359472	0.02500684	0.042012673
ACER3	0.296220057	5.98E-09	3.42E-08
ACE	0.112288134	0.030589927	0.050302313
ACHE	0.239360178	3.12E-06	1.13E-05
ACIN1	0.368118531	2.38E-13	2.71E-12
ACLY	0.257887859	4.76E-07	1.97E-06
ACMSD	-0.252483173	8.36E-07	3.33E-06
ACN9	-0.228584954	8.71E-06	2.91E-05
ACO1	-0.230324417	7.40E-06	2.51E-05
ACO2	0.010236833	0.844207006	0.876683276
ACOT11	0.389241798	7.19E-15	1.04E-13
ACOT12	-0.260692219	3.53E-07	1.50E-06
ACOT13	-0.391762689	4.66E-15	6.91E-14
ACOT1	-0.349587151	4.19E-12	3.92E-11
ACOT2	-0.382286874	2.34E-14	3.09E-13
ACOT4	-0.222098692	1.58E-05	5.02E-05
ACOT6	-0.360168136	8.34E-13	8.63E-12
ACOT7	0.238270289	3.47E-06	1.24E-05
ACOT8	0.056013001	0.281886613	0.352725651
ACOT9	0.294568562	7.32E-09	4.14E-08
ACOX1	-0.509835367	6.14E-26	4.72E-24
ACOX2	-0.4065971	3.34E-16	5.91E-15
ACOX3	-0.157284851	0.00237973	0.005047267
ACOXL	0.109370225	0.035218996	0.057020141
ACP1	0.115023764	0.026732916	0.044622803
ACP2	0.025626448	0.622708452	0.687709839
ACP5	0.338992399	1.99E-11	1.67E-10

ACP6	-0.054566723	0.294517099	0.366412893
ACPL2	0.081487044	0.117147062	0.165014761
ACPP	0.37222096	1.23E-13	1.47E-12
ACPT	0.240793446	2.71E-06	9.92E-06
ACRBP	0.047569782	0.360882841	0.436468429
ACRC	0.036209316	0.486857787	0.560970551
ACRV1	0.353879891	2.19E-12	2.14E-11
ACR	-0.376165489	6.47E-14	8.05E-13
ACSBG1	0.0242478	0.64154622	0.705230027
ACSBG2	0.098031095	0.059242912	0.090592362
ACSF2	-0.318841172	3.27E-10	2.27E-09
ACSF3	-0.314058917	6.17E-10	4.11E-09
ACSL1	-0.341832458	1.32E-11	1.14E-10
ACSL3	0.135394839	0.009024559	0.016821413
ACSL4	0.254245357	6.96E-07	2.81E-06
ACSL5	-0.403305259	6.06E-16	1.04E-14
ACSL6	-0.260954777	3.43E-07	1.46E-06
ACSM1	-0.138812505	0.007413615	0.014069078
ACSM2A	-0.528816327	4.06E-28	4.40E-26
ACSM2B	-0.502239641	4.19E-25	2.81E-23
ACSM3	-0.482383346	5.10E-23	2.41E-21
ACSM4	0.059087213	0.256269879	0.325185257
ACSM5	-0.476317861	2.08E-22	9.09E-21
ACSS1	0.457671211	1.32E-20	4.39E-19
ACSS2	-0.211873363	3.89E-05	0.000115799
ACSS3	-0.31085575	9.38E-10	6.09E-09
ACTA1	0.288470402	1.53E-08	8.18E-08
ACTA2	0.032525848	0.532270885	0.604176433
ACTBL2	0.190473368	0.000224047	0.000578993
ACTB	0.26206871	3.05E-07	1.31E-06
ACTC1	0.084421256	0.10448676	0.149388922
ACTG1	0.323499795	1.74E-10	1.27E-09
ACTG2	0.078116253	0.133137493	0.184307216
ACTL6A	0.430373723	3.68E-18	8.69E-17
ACTL6B	0.073330545	0.158664299	0.214589963
ACTL7A	0.012165705	0.815337512	0.852748525
ACTL7B	0.039576385	0.447242367	0.522977193
ACTL8	0.272004653	1.03E-07	4.75E-07
ACTL9	0.0259727	0.618015049	0.68309453
ACTN1	0.136763815	0.008345242	0.015659217
ACTN2	-0.113487922	0.028843348	0.047761381
ACTN3	0.141398062	0.00637104	0.012275034
ACTN4	0.19349839	0.000176859	0.000465994
ACTR10	0.091632594	0.077947281	0.115420375
ACTR1A	0.169584148	0.001041087	0.002367641
ACTR1B	-0.096430374	0.063533633	0.096428238
ACTR2	0.253614581	7.44E-07	2.99E-06
ACTR3B	-0.200357948	0.000102071	0.000281331
ACTR3C	-0.126904373	0.014445128	0.025616507
ACTR3	0.370042645	1.75E-13	2.03E-12
ACTR5	0.04440155	0.393787477	0.47012849
ACTR6	0.159616562	0.002043392	0.004391893
ACTR8	0.237401593	3.77E-06	1.34E-05
ACTRT1	0.091776429	0.077479552	0.114787509
ACVR1B	0.192567918	0.000190277	0.000498317
ACVR1C	-0.202430026	8.61E-05	0.000240848
ACVR1	0.091069776	0.079799532	0.117865233
ACVR2A	-0.062029289	0.233306431	0.299829057

ACVR2B	0.11907898	0.021787551	0.037136515
ACVRL1	0.081031995	0.119213639	0.167511222
ACY1	-0.415199902	6.81E-17	1.35E-15
ACY3	-0.324671434	1.49E-10	1.09E-09
ACYP1	0.430701116	3.45E-18	8.19E-17
ACYP2	-0.479534325	9.91E-23	4.56E-21
ADAD1	-0.021986171	0.672947988	0.732513923
ADAD2	-0.145278171	0.005051966	0.009940272
ADAL	-0.354669498	1.94E-12	1.91E-11
ADAM10	0.044019689	0.397872028	0.474180817
ADAM11	0.186938302	0.000294032	0.000742515
ADAM12	0.459720532	8.46E-21	2.90E-19
ADAM15	0.288237048	1.57E-08	8.40E-08
ADAM17	0.284175557	2.54E-08	1.31E-07
ADAM18	0.080900431	0.119816405	0.168246531
ADAM19	0.37214275	1.25E-13	1.48E-12
ADAM20	0.01879182	0.718274043	0.771482396
ADAM21P1	-0.051715155	0.320511289	0.394125419
ADAM21	0.007217473	0.889804936	0.913137055
ADAM22	0.270704892	1.19E-07	5.45E-07
ADAM23	0.284565379	2.43E-08	1.26E-07
ADAM28	0.387970271	8.95E-15	1.26E-13
ADAM29	0.124676771	0.016273532	0.028496204
ADAM2	0.063726331	0.220741511	0.285949177
ADAM30	0.061745646	0.235454837	0.302161202
ADAM32	0.159064745	0.002118811	0.004537348
ADAM33	0.057378234	0.270304417	0.34024165
ADAM3A	0.128077981	0.013556427	0.024221628
ADAM5P	0.021469357	0.680206702	0.738760176
ADAM6	0.196335348	0.000141214	0.000379044
ADAM7	0.104646111	0.043971922	0.06957419
ADAM8	0.381501228	2.67E-14	3.50E-13
ADAM9	0.204022965	7.55E-05	0.000213266
ADAMDEC1	0.344200875	9.31E-12	8.23E-11
ADAMTS10	0.129027801	0.012872779	0.023120402
ADAMTS12	0.104138889	0.045012122	0.071078907
ADAMTS13	0.05558731	0.285565612	0.356768978
ADAMTS14	0.419492166	3.03E-17	6.32E-16
ADAMTS15	0.039900905	0.443523135	0.519176679
ADAMTS16	0.133679155	0.009945085	0.018367107
ADAMTS17	0.013356892	0.797630269	0.838277601
ADAMTS18	-0.037038058	0.476935484	0.551451463
ADAMTS19	0.177574813	0.000590071	0.001410534
ADAMTS1	-0.008716399	0.867113278	0.895569015
ADAMTS20	0.117737653	0.023327475	0.039527936
ADAMTS2	0.121676618	0.019054358	0.032863035
ADAMTS3	0.296945979	5.47E-09	3.15E-08
ADAMTS4	-0.018996469	0.715338328	0.768826617
ADAMTS5	0.231718199	6.49E-06	2.22E-05
ADAMTS6	0.394609771	2.84E-15	4.35E-14
ADAMTS7	0.215637326	2.80E-05	8.53E-05
ADAMTS8	-0.045335675	0.383902773	0.460119783
ADAMTS9	0.191645084	0.000204522	0.000532751
ADAMTSL1	-0.011379624	0.827075042	0.862579258
ADAMTSL2	0.090800335	0.080698794	0.118964139
ADAMTSL3	-0.269418704	1.37E-07	6.22E-07
ADAMTSL4	-0.387592167	9.54E-15	1.34E-13
ADAMTSL5	0.128551499	0.013211711	0.023654508

ADAP1	0.408316834	2.44E-16	4.41E-15
ADAP2	0.259102631	4.18E-07	1.75E-06
ADARB1	-0.043131627	0.407468969	0.483737369
ADARB2	0.19775778	0.000125993	0.000341501
ADAR	0.087774409	0.091371097	0.132751968
ADAT1	-0.153558933	0.003023094	0.006253356
ADAT2	0.277499923	5.51E-08	2.68E-07
ADAT3	0.154457336	0.002854944	0.005938212
ADA	0.350957617	3.41E-12	3.22E-11
ADCK1	-0.034615682	0.506245966	0.579289611
ADCK2	0.052653956	0.31179334	0.384620015
ADCK4	0.020488354	0.694066044	0.750659284
ADCK5	0.032715606	0.529880256	0.601908681
ADCY10	-0.223713268	1.36E-05	4.39E-05
ADCY1	-0.121259035	0.019472497	0.033520602
ADCY2	0.187695831	0.000277508	0.000705077
ADCY3	0.265411735	2.12E-07	9.37E-07
ADCY4	-0.061851033	0.234654981	0.30132761
ADCY5	0.215669281	2.80E-05	8.51E-05
ADCY6	0.289642936	1.33E-08	7.22E-08
ADCY7	0.269276969	1.39E-07	6.31E-07
ADCY8	-0.136882995	0.008288315	0.015567061
ADCY9	-0.167845834	0.001174174	0.002646312
ADCYAP1R	-0.03442658	0.50857313	0.581484121
ADCYAP1	-0.196242697	0.000142263	0.000381654
ADC	0.061219129	0.239479737	0.306692937
ADD1	0.008016227	0.877700511	0.903739383
ADD2	0.253872983	7.24E-07	2.91E-06
ADD3	0.205288295	6.80E-05	0.000193541
ADH1A	-0.41154666	1.35E-16	2.55E-15
ADH1B	-0.621802444	4.53E-41	1.29E-37
ADH1C	-0.436854543	1.01E-18	2.61E-17
ADH4	-0.523610229	1.66E-27	1.61E-25
ADH5	-0.365656774	3.52E-13	3.89E-12
ADH6	-0.546878009	2.53E-30	3.80E-28
ADH7	-0.292734341	9.15E-09	5.10E-08
ADHFE1	-0.440390991	4.94E-19	1.33E-17
ADI1	-0.467192214	1.64E-21	6.18E-20
ADIG	-0.130366519	0.011960843	0.021662175
ADIPOQ	0.030200954	0.561995037	0.632331702
ADIPOR1	-0.092505967	0.075142158	0.111673411
ADIPOR2	-0.315585929	5.04E-10	3.41E-09
ADK	-0.314140634	6.11E-10	4.07E-09
ADM2	0.353810659	2.21E-12	2.16E-11
ADM	-0.075054356	0.149075033	0.203290629
ADNP2	0.115211969	0.026483669	0.044258674
ADNP	0.140190663	0.006840334	0.013083059
ADORA1	0.262116305	3.03E-07	1.30E-06
ADORA2A	0.148413669	0.004171611	0.008358326
ADORA2B	0.278866312	4.71E-08	2.32E-07
ADORA3	0.329628876	7.49E-11	5.78E-10
ADO	0.295403859	6.61E-09	3.77E-08
ADPGK	0.184619125	0.000350506	0.000872957
ADPRHL1	0.149344502	0.003938368	0.007938099
ADPRHL2	0.242018801	2.40E-06	8.86E-06
ADPRH	0.273385895	8.78E-08	4.12E-07
ADRA1A	-0.387184576	1.02E-14	1.42E-13
ADRA1B	-0.370401581	1.65E-13	1.92E-12

ADRA1D	0.108933223	0.035961117	0.058084756
ADRA2A	0.190968272	0.000215596	0.000558823
ADRA2B	-0.278622926	4.84E-08	2.38E-07
ADRA2C	0.005309293	0.918820302	0.936543262
ADRB1	-0.244707927	1.84E-06	6.91E-06
ADRB2	-0.464447302	3.01E-21	1.11E-19
ADRB3	0.266996133	1.79E-07	7.98E-07
ADRBK1	0.187184399	0.000288566	0.000730101
ADRBK2	-0.125581332	0.015507979	0.027292399
ADRM1	0.113402517	0.028964785	0.047926615
ADSL	0.231228009	6.80E-06	2.32E-05
ADSSL1	-0.134022807	0.009754297	0.018056598
ADSS	0.332500797	5.01E-11	3.95E-10
AEBP1	0.146830971	0.004596991	0.009131644
AEBP2	0.21517502	2.92E-05	8.86E-05
AEN	-0.088757403	0.08778673	0.128104837
AES	0.072611682	0.162797879	0.21940599
AFAP1L1	0.117411564	0.023715761	0.040100611
AFAP1L2	0.160550335	0.001921337	0.004146557
AFAP1	0.360960076	7.37E-13	7.71E-12
AFARP1	-0.321373252	2.33E-10	1.65E-09
AFF1	-0.055605515	0.285407611	0.356593944
AFF2	0.147718066	0.004353983	0.008695744
AFF3	0.127211867	0.014207469	0.025237734
AFF4	-0.093157721	0.07310278	0.109073816
AFG3L1	0.193758285	0.000173272	0.00045727
AFG3L2	-0.011046749	0.832057201	0.866731689
AFMID	-0.402035147	7.62E-16	1.28E-14
AFM	-0.368985819	2.07E-13	2.37E-12
AFP	0.306169642	1.72E-09	1.07E-08
AFTPH	0.225565411	1.15E-05	3.76E-05
AG2	0.059018729	0.256822406	0.325824109
AGAP11	-0.031148751	0.549781312	0.620939015
AGAP1	0.395255453	2.53E-15	3.90E-14
AGAP2	0.069700179	0.180369046	0.239626913
AGAP3	-0.101948386	0.049744642	0.07768384
AGAP4	0.189413469	0.000243197	0.000623949
AGAP5	0.063025083	0.225873696	0.291441436
AGAP6	0.171852439	0.00088831	0.002048729
AGAP7	0.156498522	0.002504069	0.00528566
AGAP8	0.185365744	0.000331308	0.000829289
AGA	0.118955143	0.021925933	0.037346823
AGBL1	0.116453941	0.024888486	0.041863257
AGBL2	-0.022124997	0.671003274	0.731116125
AGBL3	0.055321661	0.287877763	0.359387191
AGBL4	-0.006182955	0.905519863	0.925875934
AGBL5	0.072454708	0.163711171	0.220462497
AGER	0.193903491	0.000171298	0.0004526
AGFG1	0.27397075	8.22E-08	3.87E-07
AGFG2	-0.247953453	1.33E-06	5.11E-06
AGGF1	0.14808798	0.004256122	0.00851652
AGK	0.05993076	0.249531579	0.317646525
AGL	-0.382187325	2.38E-14	3.14E-13
AGMAT	-0.30191162	2.94E-09	1.77E-08
AGPAT1	-0.008766925	0.866350183	0.895012878
AGPAT2	0.213501189	3.38E-05	0.000101584
AGPAT3	-0.057409119	0.270046216	0.339981086
AGPAT4	0.245351902	1.73E-06	6.50E-06

AGPAT5	0.263955574	2.49E-07	1.08E-06
AGPAT6	-0.016831912	0.746593476	0.795253982
AGPAT9	-0.132063495	0.010887138	0.019902144
AGPHD1	-0.234992077	4.76E-06	1.67E-05
AGPS	0.096260742	0.064002948	0.097037042
AGR2	0.367905972	2.46E-13	2.80E-12
AGR3	0.247685958	1.36E-06	5.24E-06
AGRN	0.309305356	1.15E-09	7.33E-09
AGRP	0.069151642	0.183830997	0.243502734
AGTPBP1	0.288977944	1.44E-08	7.76E-08
AGTR1	-0.108342524	0.036985321	0.059594109
AGTR2	0.116105749	0.025327118	0.042504157
AGTRAP	0.259604601	3.96E-07	1.67E-06
AGT	0.014584633	0.77948763	0.822725963
AGXT2L1	-0.3476654	5.58E-12	5.09E-11
AGXT2L2	-0.150497615	0.003665813	0.007440685
AGXT2	-0.307418518	1.46E-09	9.21E-09
AGXT	-0.316307179	4.58E-10	3.12E-09
AHCTF1	0.056793668	0.275223283	0.345557291
AHCYL1	-0.125394179	0.01566373	0.027542174
AHCYL2	-0.199768727	0.000107085	0.000294011
AHCY	0.043042328	0.408441534	0.484660923
AHDC1	0.109818326	0.034471585	0.055960147
AHI1	0.060804826	0.242680625	0.310213886
AHNAK2	0.171058981	0.000939227	0.002154685
AHNAK	-0.041702846	0.4231945	0.499489356
AHRR	0.10315676	0.047085043	0.074029535
AHR	-0.315611037	5.03E-10	3.40E-09
AHSA1	0.193163966	0.000181576	0.000477665
AHSA2	0.100962096	0.052007394	0.080776744
AHSG	-0.287624494	1.69E-08	8.99E-08
AHSP	-0.049434673	0.342342697	0.416624997
AICDA	0.220521962	1.82E-05	5.73E-05
AIDA	0.178760899	0.00054125	0.001302427
AIF1L	-0.05180796	0.319642477	0.393154128
AIF1	0.223920639	1.34E-05	4.32E-05
AIFM1	-0.250639237	1.01E-06	3.98E-06
AIFM2	0.074427623	0.15250914	0.207345474
AIFM3	0.114563612	0.027350822	0.045543554
AIG1	-0.29428868	7.57E-09	4.27E-08
AIM1L	0.076090729	0.143525778	0.196621687
AIM1	-0.155851105	0.002610854	0.005488965
AIM2	0.20104179	9.65E-05	0.000267426
AIMP1	-0.050313031	0.333824361	0.407802852
AIMP2	-0.083281967	0.109267537	0.155299127
AIPL1	0.172250401	0.000863744	0.001997157
AIP	0.04397433	0.398358899	0.47460271
AIRE	0.211693221	3.95E-05	0.000117486
AJAP1	0.094509036	0.06901806	0.103732765
AK1	0.018161645	0.727339862	0.778826377
AK2	-0.041135206	0.42953901	0.505884306
AK3L1	-0.001862588	0.971477755	0.977611751
AK3	-0.405391988	4.16E-16	7.24E-15
AK5	0.159146459	0.002107485	0.004515035
AK7	-0.120409544	0.020347821	0.03489449
AKAP10	0.032886451	0.527732561	0.599776832
AKAP11	-0.172389378	0.000855314	0.001980538
AKAP12	-0.04074593	0.433921566	0.510005041

AKAP13	-0.0874333	0.09264195	0.134346359
AKAP14	0.042353817	0.4159863	0.491914232
AKAP1	-0.102561957	0.048378912	0.075884158
AKAP2	-0.129285155	0.012692855	0.022828117
AKAP3	-0.402143362	7.47E-16	1.26E-14
AKAP4	0.066137459	0.203731988	0.266759689
AKAP5	0.081893363	0.115325552	0.162759681
AKAP6	-0.38518716	1.44E-14	1.95E-13
AKAP7	-0.071269726	0.170729986	0.22871917
AKAP8L	0.305306191	1.92E-09	1.18E-08
AKAP8	0.273562742	8.61E-08	4.04E-07
AKAP9	-0.174531045	0.000734649	0.001723466
AKD1	0.067451252	0.194873727	0.256424992
AKIRIN1	0.16817062	0.001148176	0.002592538
AKIRIN2	-0.092821926	0.074147798	0.110401527
AKNAD1	0.148560306	0.004134059	0.008288086
AKNA	0.253664416	7.40E-07	2.97E-06
AKR1A1	-0.167090328	0.001236757	0.002773688
AKR1B10	0.145435605	0.005004057	0.009857691
AKR1B15	0.105939474	0.041410638	0.06599334
AKR1B1	0.239240564	3.16E-06	1.14E-05
AKR1C1	-0.117495444	0.023615352	0.039951176
AKR1C2	-0.135949526	0.008743598	0.016340522
AKR1C3	0.193105793	0.000182409	0.000479665
AKR1C4	-0.247021807	1.46E-06	5.57E-06
AKR1CL1	-0.138425218	0.007582262	0.014362824
AKR1D1	-0.305287174	1.92E-09	1.19E-08
AKR1E2	0.078340285	0.132025054	0.182932476
AKR7A2	-0.274033494	8.16E-08	3.85E-07
AKR7A3	-0.325849358	1.26E-10	9.35E-10
AKR7L	-0.220502734	1.82E-05	5.73E-05
AKT1S1	0.04693106	0.367374399	0.443189583
AKT1	0.019719325	0.705002649	0.760107739
AKT2	-0.257480713	4.96E-07	2.05E-06
AKT3	0.096454379	0.063467447	0.096335122
AKTIP	-0.155392381	0.002689008	0.005630693
ALAD	-0.524002236	1.50E-27	1.46E-25
ALAS1	-0.422249324	1.79E-17	3.86E-16
ALAS2	-0.081024257	0.119249028	0.167549131
ALB	-0.162314361	0.001708779	0.003722097
ALCAM	-0.111440505	0.031877705	0.052204491
ALDH16A1	0.159418246	0.00207021	0.004444258
ALDH18A1	0.230214127	7.48E-06	2.53E-05
ALDH1A1	-0.29623142	5.97E-09	3.42E-08
ALDH1A2	0.230280355	7.43E-06	2.52E-05
ALDH1A3	0.078888364	0.129333787	0.17971596
ALDH1B1	-0.352768467	2.59E-12	2.50E-11
ALDH1L1	-0.447503886	1.14E-19	3.32E-18
ALDH1L2	0.212261981	3.76E-05	0.000112377
ALDH2	-0.556351752	1.56E-31	3.04E-29
ALDH3A1	-0.177545831	0.000591313	0.001412995
ALDH3A2	-0.396591929	2.00E-15	3.13E-14
ALDH3B1	0.42555362	9.45E-18	2.11E-16
ALDH3B2	0.323200018	1.82E-10	1.31E-09
ALDH4A1	-0.421674218	2.00E-17	4.26E-16
ALDH5A1	-0.473192516	4.24E-22	1.75E-20
ALDH6A1	-0.533145712	1.24E-28	1.40E-26
ALDH7A1	-0.277955165	5.23E-08	2.55E-07

ALDH8A1	-0.277132573	5.74E-08	2.78E-07
ALDH9A1	-0.383626006	1.87E-14	2.50E-13
ALDOA	0.500850896	5.93E-25	3.82E-23
ALDOB	-0.200731922	9.90E-05	0.000273712
ALDOC	-0.01479096	0.776450103	0.820171737
ALG10B	-0.177476992	0.000594274	0.001419673
ALG10	-0.038524154	0.459422393	0.534427941
ALG11	0.180321228	0.000482725	0.001171501
ALG12	0.039132216	0.452361367	0.527964275
ALG13	-0.04893177	0.347281623	0.421656783
ALG14	-0.115214672	0.026480105	0.04425643
ALG1L2	0.144632805	0.005252701	0.010289509
ALG1L	0.192791059	0.000186975	0.000490637
ALG1	-0.048269845	0.353850742	0.428744342
ALG2	-0.079854302	0.12469422	0.174180217
ALG3	0.182396296	0.000413976	0.001016294
ALG5	-0.128614126	0.013166702	0.023580281
ALG6	0.141000572	0.006522249	0.012537268
ALG8	-0.031073218	0.550749862	0.621962403
ALG9	-0.137706146	0.007904493	0.01491931
ALKBH1	-0.070251479	0.176938499	0.235815412
ALKBH2	-0.069935999	0.178895646	0.237962147
ALKBH3	-0.158717023	0.002167632	0.004630449
ALKBH4	-0.041187613	0.428950964	0.505355476
ALKBH5	-0.162271925	0.001713629	0.003732253
ALKBH6	-0.010022631	0.84742663	0.879522444
ALKBH7	-0.216388272	2.62E-05	8.03E-05
ALKBH8	-0.039420325	0.44903719	0.524829504
ALK	0.128626396	0.0131579	0.023570873
ALLC	0.036457147	0.483878986	0.557990111
ALMS1P	0.054879231	0.291756442	0.363454967
ALMS1	0.122227708	0.018514515	0.032031799
ALOX12B	0.176174177	0.000652968	0.001549176
ALOX12P2	-0.040625734	0.435279952	0.511450735
ALOX12	0.031867179	0.540610971	0.612213038
ALOX15B	0.239754643	3.00E-06	1.09E-05
ALOX15	-0.002173281	0.966722603	0.97390886
ALOX5AP	0.296502807	5.77E-09	3.31E-08
ALOX5	0.426705719	7.56E-18	1.71E-16
ALOXE3	0.296765455	5.59E-09	3.21E-08
ALPI	0.209452947	4.78E-05	0.000140145
ALPK1	-0.032863539	0.528020335	0.599966983
ALPK2	-0.075612113	0.146068593	0.199669172
ALPK3	0.202131084	8.83E-05	0.000246291
ALPL	-0.255860155	5.88E-07	2.40E-06
ALPPL2	0.135752235	0.008842626	0.016508555
ALPP	0.226401065	1.07E-05	3.51E-05
ALS2CL	0.200806217	9.84E-05	0.000272283
ALS2CR11	0.046892412	0.3677695	0.443585638
ALS2CR12	0.073262875	0.159050003	0.214980268
ALS2CR4	0.420828395	2.35E-17	4.95E-16
ALS2CR8	-0.265596693	2.08E-07	9.20E-07
ALS2	0.083808596	0.10703667	0.152520173
ALX1	0.133027522	0.010315967	0.018974674
ALX3	0.106093974	0.041113266	0.06557189
ALX4	0.145989947	0.004838606	0.009569613
AMAC1L2	0.133435727	0.010082226	0.01859453
AMAC1L3	-0.01345309	0.796204648	0.837132626

AMAC1	-0.001016129	0.984437466	0.98814752
AMACR	-0.449244563	7.92E-20	2.36E-18
AMBN	0.057237393	0.271484009	0.34140288
AMBP	-0.310636814	9.65E-10	6.26E-09
AMBRA1	-0.00152901	0.976584296	0.98165351
AMD1	0.402122871	7.50E-16	1.26E-14
AMDHD1	-0.279118417	4.57E-08	2.26E-07
AMDHD2	-0.051116836	0.326149427	0.400292563
AMELX	-0.03435852	0.509412068	0.58231049
AMELY	0.021577605	0.678683876	0.737347449
AMFR	-0.358183156	1.13E-12	1.15E-11
AMHR2	0.101068986	0.051758096	0.080462333
AMH	0.183083663	0.00039329	0.000969814
AMICA1	0.214554799	3.08E-05	9.33E-05
AMIGO1	-0.149257191	0.003959734	0.007977934
AMIGO2	0.327076043	1.07E-10	8.01E-10
AMIGO3	0.272465764	9.74E-08	4.53E-07
AMMECR1I	0.055134117	0.289517664	0.361095029
AMMECR1	0.180198132	0.00048712	0.001181302
AMN1	-0.027715461	0.594630228	0.66202429
AMN	-0.003843032	0.941191409	0.954656854
AMOTL1	0.089352993	0.085670516	0.125420931
AMOTL2	-0.199330657	0.000110961	0.000303775
AMOT	-0.053399003	0.304986504	0.377606822
AMPD1	0.211419678	4.04E-05	0.000120121
AMPD2	-0.305075876	1.97E-09	1.22E-08
AMPD3	0.359073166	9.88E-13	1.01E-11
AMPH	0.183701025	0.000375535	0.000929367
AMTN	0.194289197	0.000166157	0.000440358
AMT	-0.327510865	1.00E-10	7.57E-10
AMY1A	-0.139832624	0.006985323	0.0133386
AMY2A	-0.069980255	0.178620131	0.237659136
AMY2B	-0.141188822	0.006450241	0.012407231
AMZ1	0.259909669	3.84E-07	1.62E-06
AMZ2P1	0.042528507	0.41406432	0.490046874
AMZ2	0.140915629	0.006554976	0.012595316
ANAPC10	-0.036480733	0.483595999	0.557728349
ANAPC11	0.082790118	0.111383987	0.15791288
ANAPC13	0.009991262	0.847898347	0.879782857
ANAPC16	-0.123615367	0.017213826	0.029979355
ANAPC1	0.110427667	0.03347692	0.05452761
ANAPC2	-0.199871609	0.000106193	0.000291763
ANAPC4	0.392424276	4.15E-15	6.21E-14
ANAPC5	0.209648731	4.71E-05	0.000137948
ANAPC7	0.508157224	9.42E-26	6.90E-24
ANGEL1	0.1621904	0.001722981	0.003750572
ANGEL2	-0.083379251	0.108852695	0.154808923
ANGPT1	-0.037201044	0.474997159	0.549720773
ANGPT2	0.060712358	0.243399097	0.31101256
ANGPT4	-0.099459022	0.055620205	0.085770239
ANGPTL1	-0.221151993	1.72E-05	5.43E-05
ANGPTL2	-0.013811962	0.790892314	0.832791631
ANGPTL3	-0.506078115	1.60E-25	1.14E-23
ANGPTL4	-0.159181965	0.002102581	0.004505982
ANGPTL5	0.143090947	0.005761632	0.01118091
ANGPTL6	-0.323321982	1.79E-10	1.29E-09
ANGPTL7	-0.183534839	0.00038024	0.000940076
ANG	-0.360253205	8.23E-13	8.54E-12

ANK1	0.288805555	1.47E-08	7.90E-08
ANK2	0.210937864	4.21E-05	0.000124766
ANK3	0.220249146	1.86E-05	5.85E-05
ANKAR	-0.080616614	0.12112481	0.169873176
ANKDD1A	0.288078643	1.60E-08	8.55E-08
ANKFN1	-0.133328546	0.010143139	0.018691298
ANKFY1	-0.144766395	0.00521057	0.010215014
ANKHD1-El	0.112921817	0.029656497	0.048939145
ANKHD1	-0.005964701	0.908840195	0.928391183
ANKH	0.027716047	0.594622435	0.66202429
ANKIB1	0.050321021	0.333747505	0.407758971
ANKK1	-0.063098102	0.225335372	0.290935274
ANKLE1	0.300764218	3.40E-09	2.02E-08
ANKLE2	0.417409136	4.49E-17	9.12E-16
ANKMY1	-0.205984357	6.41E-05	0.000183626
ANKMY2	-0.054025759	0.299337007	0.3715972
ANKRA2	-0.144826831	0.005191609	0.010184859
ANKRD10	0.219411394	2.01E-05	6.27E-05
ANKRD11	0.042944807	0.409505221	0.48574951
ANKRD12	-0.020489878	0.694044435	0.750659284
ANKRD13A	0.364217298	4.42E-13	4.80E-12
ANKRD13B	0.296115314	6.05E-09	3.46E-08
ANKRD13C	-0.00786101	0.880050554	0.905256333
ANKRD13D	0.541147313	1.31E-29	1.72E-27
ANKRD16	0.182822218	0.000401042	0.000987408
ANKRD17	-0.149374264	0.003931109	0.007925071
ANKRD19	0.128355951	0.013353121	0.023883572
ANKRD1	0.152774841	0.003177148	0.006545516
ANKRD20A	0.025992954	0.617740985	0.68282949
ANKRD20A	0.007008156	0.89298131	0.91549885
ANKRD20B	-0.096951443	0.062109687	0.094490202
ANKRD22	0.362111822	6.16E-13	6.52E-12
ANKRD23	0.23333686	5.57E-06	1.94E-05
ANKRD24	-0.433200924	2.10E-18	5.17E-17
ANKRD26P	0.172028444	0.000877367	0.00202607
ANKRD26	0.052389458	0.314233639	0.387098847
ANKRD27	0.307612553	1.43E-09	9.01E-09
ANKRD28	-0.076605239	0.14083013	0.1934977
ANKRD29	-0.087752549	0.091452118	0.13284062
ANKRD2	0.03001922	0.564351777	0.634513034
ANKRD30A	0.113797532	0.028406769	0.047108936
ANKRD30B	0.073938117	0.155232773	0.210477595
ANKRD31	-0.079037906	0.128606898	0.178868097
ANKRD32	0.387161679	1.03E-14	1.43E-13
ANKRD33	0.13727987	0.008101233	0.015248749
ANKRD34A	-0.002620806	0.959875325	0.968528982
ANKRD34B	0.26432826	2.39E-07	1.04E-06
ANKRD34C	0.013984087	0.788347775	0.830449681
ANKRD35	-0.109687964	0.034687618	0.056265
ANKRD36B	-0.026277494	0.613896412	0.679409162
ANKRD36B	0.229906431	7.70E-06	2.60E-05
ANKRD36	0.287205394	1.78E-08	9.41E-08
ANKRD37	-0.361610549	6.66E-13	7.01E-12
ANKRD39	0.190264284	0.000227709	0.00058737
ANKRD40	0.031540277	0.544774238	0.616087214
ANKRD42	-0.1542979	0.00288415	0.005990203
ANKRD43	0.064279968	0.216748874	0.281454247
ANKRD44	0.058027041	0.264915684	0.334494249

ANKRD45	0.157906155	0.002285496	0.004859195
ANKRD46	-0.222349473	1.54E-05	4.92E-05
ANKRD49	0.207441563	5.67E-05	0.000163946
ANKRD50	-0.231800082	6.44E-06	2.21E-05
ANKRD52	0.453397461	3.29E-20	1.03E-18
ANKRD53	0.074078925	0.154445571	0.209595685
ANKRD54	0.089881752	0.083826349	0.12307381
ANKRD55	-0.062283779	0.231390658	0.297730717
ANKRD56	-0.407421731	2.87E-16	5.14E-15
ANKRD57	-0.268666713	1.49E-07	6.73E-07
ANKRD58	0.387863495	9.11E-15	1.28E-13
ANKRD5	0.053216533	0.306644413	0.379274229
ANKRD6	0.194532813	0.000162984	0.000432586
ANKRD7	0.133547398	0.010019107	0.018484965
ANKRD9	0.003301006	0.949473877	0.960856374
ANKS1A	0.103016801	0.047386857	0.0744688
ANKS1B	0.083912523	0.106600696	0.152030153
ANKS3	0.232870377	5.82E-06	2.01E-05
ANKS4B	-0.432590297	2.37E-18	5.79E-17
ANKS6	0.353883135	2.19E-12	2.14E-11
ANKZF1	0.018140261	0.727648168	0.77911463
ANLN	0.534758036	7.91E-29	9.33E-27
ANO10	0.203648955	7.79E-05	0.000219434
ANO1	-0.205710221	6.56E-05	0.000187429
ANO2	-0.160415567	0.001938531	0.004182305
ANO3	-0.019667722	0.705738729	0.760643095
ANO4	0.296383041	5.86E-09	3.36E-08
ANO5	0.168319577	0.001136431	0.002568347
ANO6	-0.198809528	0.000115744	0.000315741
ANO7	0.105928763	0.041431322	0.06602102
ANO8	0.060456684	0.245393416	0.313199285
ANO9	0.509359744	6.93E-26	5.23E-24
ANP32A	0.30243117	2.76E-09	1.67E-08
ANP32B	0.246286705	1.57E-06	5.97E-06
ANP32C	0.150686774	0.00362277	0.007363072
ANP32D	0.047803055	0.358529985	0.433934444
ANP32E	0.238186907	3.50E-06	1.25E-05
ANPEP	-0.020485646	0.694104436	0.750659284
ANTXR1	0.192622168	0.000189469	0.000496332
ANTXR2	-0.06345454	0.222720678	0.288194424
ANTXRL	0.086891856	0.094688166	0.136932175
ANUBL1	-0.169031865	0.001081782	0.002453468
ANXA10	-0.420483604	2.51E-17	5.27E-16
ANXA11	0.249686337	1.11E-06	4.35E-06
ANXA13	0.372203429	1.23E-13	1.47E-12
ANXA1	0.15087691	0.003579967	0.00728202
ANXA2P1	0.351544581	3.12E-12	2.97E-11
ANXA2P2	0.386808455	1.09E-14	1.51E-13
ANXA2P3	0.312319016	7.75E-10	5.09E-09
ANXA2	0.390509191	5.78E-15	8.42E-14
ANXA3	0.069404536	0.182228883	0.241621551
ANXA4	0.341554829	1.37E-11	1.18E-10
ANXA5	0.422298185	1.77E-17	3.82E-16
ANXA6	-0.247684837	1.36E-06	5.24E-06
ANXA7	-0.213995451	3.24E-05	9.75E-05
ANXA8L1	0.065010453	0.211560433	0.275561095
ANXA8L2	0.168477397	0.001124108	0.002542227
ANXA8	0.243400413	2.10E-06	7.79E-06

ANXA9	0.157456949	0.002353279	0.004995421
AOAH	0.154466962	0.002853189	0.005935182
AOC2	-0.055042462	0.290321381	0.361961484
AOC3	-0.165068881	0.001419596	0.003150056
AOX1	-0.519547684	4.90E-27	4.38E-25
AOX2P	-0.146468643	0.004699716	0.009322687
AP1AR	-0.16969117	0.001033366	0.002351962
AP1B1	0.16412008	0.001513702	0.003335468
AP1G1	-0.265950614	2.00E-07	8.87E-07
AP1G2	0.47390811	3.61E-22	1.50E-20
AP1M1	-0.068733883	0.186500274	0.246628507
AP1M2	0.223781737	1.35E-05	4.37E-05
AP1S1	-0.083826113	0.106963087	0.152437129
AP1S2	0.153565748	0.003021785	0.006251947
AP1S3	0.352252586	2.80E-12	2.69E-11
AP2A1	-0.021016569	0.686590551	0.744151948
AP2A2	0.157481435	0.002349538	0.00498801
AP2B1	-0.019426869	0.709177903	0.76348242
AP2M1	0.061171428	0.239846754	0.307143217
AP2S1	0.190504961	0.000223498	0.000577896
AP3B1	0.248660927	1.24E-06	4.78E-06
AP3B2	0.292365165	9.57E-09	5.31E-08
AP3D1	0.155828112	0.002614722	0.005496516
AP3M1	0.067724675	0.193066085	0.25429835
AP3M2	0.335945327	3.07E-11	2.50E-10
AP3S1	-0.010312388	0.843071968	0.875894535
AP3S2	-0.241969644	2.41E-06	8.90E-06
AP4B1	0.054674929	0.293559247	0.365403769
AP4E1	0.178192793	0.000564146	0.001353435
AP4M1	0.343637649	1.01E-11	8.89E-11
AP4S1	-0.087707568	0.091619018	0.13305395
APAF1	0.278111839	5.14E-08	2.51E-07
APBA1	-0.258384025	4.51E-07	1.88E-06
APBA2	0.266323484	1.92E-07	8.54E-07
APBA3	-0.046853642	0.368166117	0.444037134
APBB1IP	0.206538222	6.12E-05	0.000175924
APBB1	0.178558898	0.000549291	0.001320339
APBB2	-0.283463549	2.76E-08	1.42E-07
APBB3	-0.122893455	0.017880173	0.031020535
APC2	-0.054981594	0.290855948	0.362559017
APCDD1L	0.335846274	3.12E-11	2.53E-10
APCDD1	0.266521391	1.88E-07	8.37E-07
APCS	-0.227683623	9.47E-06	3.14E-05
APC	0.028228462	0.587824148	0.655520582
APEH	-0.170279015	0.00099189	0.002266105
APEX1	0.190263065	0.000227731	0.00058737
APEX2	0.256773957	5.35E-07	2.20E-06
APH1A	0.046483943	0.371961431	0.447792294
APH1B	0.212238976	3.77E-05	0.000112526
API5	-0.089017304	0.086858156	0.126935752
APIP	0.074096991	0.154344793	0.209487462
APITD1	-0.029957091	0.565158545	0.635066192
APLF	0.015891924	0.760300348	0.806748524
APLNR	-0.012531696	0.809886522	0.848205198
APLN	-0.041428487	0.426254162	0.502653925
APLP1	0.324229614	1.58E-10	1.15E-09
APLP2	-0.155034517	0.00275145	0.005742131
APOA1BP	0.212183597	3.79E-05	0.000113014

APOA1	-0.170351002	0.000986918	0.00225578
APOA2	-0.069451439	0.181932883	0.241293316
APOA4	0.15271878	0.00318843	0.006561297
APOA5	-0.411199403	1.43E-16	2.70E-15
APOB48R	0.359783135	8.85E-13	9.13E-12
APOBEC1	0.228203598	9.02E-06	3.01E-05
APOBEC2	0.132048951	0.010895965	0.019914398
APOBEC3A	0.275649191	6.80E-08	3.25E-07
APOBEC3B	0.279267494	4.50E-08	2.22E-07
APOBEC3C	0.233883713	5.29E-06	1.84E-05
APOBEC3D	0.292142333	9.83E-09	5.44E-08
APOBEC3F	0.171617315	0.000903126	0.002077848
APOBEC3G	0.284575204	2.43E-08	1.26E-07
APOBEC3H	0.158176695	0.002245537	0.00478302
APOBEC4	0.096185418	0.064212255	0.097332158
APOB	-0.420804988	2.36E-17	4.97E-16
APOC1P1	-0.079654352	0.125643849	0.175334601
APOC1	-0.30826477	1.31E-09	8.32E-09
APOC2	-0.135143258	0.009154616	0.017044696
APOC3	-0.33004866	7.06E-11	5.47E-10
APOC4	-0.554097391	3.05E-31	5.29E-29
APOD	-0.005943902	0.909156691	0.928610105
APOE	-0.107502663	0.038484062	0.06171452
APOF	-0.322424273	2.02E-10	1.45E-09
APOH	-0.378278549	4.57E-14	5.78E-13
APOL1	-0.13040479	0.011935634	0.021618484
APOL2	0.067266901	0.19609946	0.257913993
APOL3	0.008241352	0.874293949	0.900930226
APOL4	0.219645674	1.97E-05	6.15E-05
APOL5	0.009613368	0.853585176	0.884853984
APOL6	-0.228777353	8.55E-06	2.86E-05
APOLD1	-0.234729205	4.88E-06	1.71E-05
APOM	-0.157365174	0.002367351	0.005022082
APOOL	-0.186244366	0.000309971	0.000780195
APOO	0.281359602	3.53E-08	1.78E-07
APPBP2	-0.039272042	0.450746343	0.526487374
APPL1	0.187589982	0.000279764	0.000710175
APPL2	0.145438429	0.005003202	0.00985698
APP	0.215491158	2.84E-05	8.63E-05
APRT	-0.065869952	0.205570871	0.268796461
APTX	-0.074418698	0.152558476	0.207345474
AQP10	0.303956519	2.27E-09	1.39E-08
AQP11	-0.396694294	1.97E-15	3.08E-14
AQP12A	0.064889474	0.212413477	0.276545634
AQP12B	0.126546398	0.014726196	0.026061587
AQP1	0.007311586	0.888377345	0.912046043
AQP2	0.04921122	0.344531642	0.418828373
AQP3	0.134569278	0.009457596	0.017553029
AQP4	-0.14817637	0.004233035	0.008472024
AQP5	0.048646768	0.350100526	0.424794609
AQP6	-0.199976424	0.000105292	0.000289407
AQP7P1	-0.153856801	0.002966372	0.006145596
AQP7P3	-0.110819058	0.032851012	0.053613325
AQP7	-0.285914453	2.07E-08	1.09E-07
AQP8	-0.102826641	0.047799522	0.07507585
AQP9	-0.615757339	4.29E-40	7.93E-37
AQPEP	0.125852384	0.015284813	0.026937725
AQR	0.056683216	0.27615948	0.346621166

ARAF	-0.108899611	0.036018743	0.058158973
ARAP1	0.19581599	0.000147189	0.000393756
ARAP2	0.278389792	4.97E-08	2.44E-07
ARAP3	0.117914555	0.023119139	0.039214941
ARCN1	0.038025625	0.465257048	0.540237274
ARC	-0.052694194	0.311423192	0.38425448
AREG	0.194568585	0.000162523	0.000431531
ARF1	0.174931598	0.000713909	0.001679161
ARF3	0.3497766	4.07E-12	3.81E-11
ARF4	0.261280851	3.32E-07	1.42E-06
ARF5	0.146590595	0.004664912	0.009255491
ARF6	0.094178677	0.069998999	0.105016948
ARFGAP1	0.266689297	1.85E-07	8.24E-07
ARFGAP2	-0.139385007	0.007170451	0.013655455
ARFGAP3	0.097341835	0.061060168	0.093021386
ARFGEF1	0.198212658	0.00012146	0.000330203
ARFGEF2	-0.035879378	0.490838741	0.564936703
ARFIP1	-0.034808619	0.503877368	0.577242377
ARFIP2	0.152113907	0.003312502	0.006785775
ARFRP1	0.087797909	0.091284061	0.132644863
ARG1	-0.12196543	0.018769755	0.032419894
ARG2	-0.100119044	0.054008905	0.083532583
ARGFXP2	0.086167946	0.097480127	0.140531086
ARGFX	0.090131905	0.082965109	0.121953139
ARGLU1	0.243430123	2.09E-06	7.77E-06
ARHGAP10	-0.023438623	0.65271179	0.715372409
ARHGAP11.	0.535632217	6.20E-29	7.53E-27
ARHGAP11]	0.405319436	4.21E-16	7.31E-15
ARHGAP12	-0.009690228	0.852427904	0.883884282
ARHGAP15	0.217582789	2.36E-05	7.28E-05
ARHGAP17	0.241852028	2.44E-06	9.00E-06
ARHGAP18	0.31194555	8.14E-10	5.33E-09
ARHGAP19	0.206451933	6.17E-05	0.000177076
ARHGAP1	0.20201939	8.91E-05	0.000248284
ARHGAP20	-0.026813683	0.606680379	0.672656195
ARHGAP21	0.026024068	0.617320077	0.68251571
ARHGAP22	0.410241837	1.71E-16	3.19E-15
ARHGAP23	0.098585446	0.05781399	0.088686403
ARHGAP24	-0.050975139	0.32749403	0.401610143
ARHGAP25	0.123953748	0.016909028	0.02950527
ARHGAP26	0.134173691	0.009671561	0.017915095
ARHGAP27	0.352255427	2.80E-12	2.69E-11
ARHGAP28	0.141355872	0.006386938	0.01229853
ARHGAP29	-0.086926064	0.09455783	0.136803274
ARHGAP30	0.15547651	0.002674517	0.005605552
ARHGAP31	0.028504929	0.584171141	0.652455988
ARHGAP32	-0.232594048	5.98E-06	2.06E-05
ARHGAP33	0.347058581	6.10E-12	5.55E-11
ARHGAP36	0.055067855	0.290098565	0.361706322
ARHGAP39	0.391940958	4.52E-15	6.71E-14
ARHGAP42	-0.216854964	2.52E-05	7.73E-05
ARHGAP4	0.378970851	4.07E-14	5.19E-13
ARHGAP5	-0.099545069	0.055407904	0.085456086
ARHGAP6	0.038761384	0.456660313	0.5318671
ARHGAP8	0.131759153	0.01107318	0.020205157
ARHGAP9	0.276387531	6.25E-08	3.01E-07
ARHGDIA	0.126024919	0.015144231	0.026723061
ARHGDIB	0.270035924	1.28E-07	5.84E-07

ARHGDIG	0.188354048	0.000263858	0.000672282
ARHGEF10l	-0.007045805	0.892409869	0.915148474
ARHGEF10	0.069383282	0.182363134	0.24177328
ARHGEF11	0.128826939	0.013014765	0.023354364
ARHGEF12	-0.210740114	4.29E-05	0.000126785
ARHGEF15	-0.151170539	0.003514765	0.007158894
ARHGEF16	0.277907829	5.26E-08	2.56E-07
ARHGEF17	0.160059083	0.001984692	0.004274028
ARHGEF18	0.190796486	0.000218495	0.000565895
ARHGEF19	0.198550819	0.00011819	0.000322148
ARHGEF1	0.329556751	7.56E-11	5.83E-10
ARHGEF2	0.536791051	4.48E-29	5.55E-27
ARHGEF33	0.030144044	0.562732538	0.63294702
ARHGEF35	0.225569614	1.15E-05	3.76E-05
ARHGEF37	-0.164619312	0.001463496	0.00323737
ARHGEF38	0.373959327	9.28E-14	1.12E-12
ARHGEF3	0.288429242	1.54E-08	8.22E-08
ARHGEF4	0.368032478	2.42E-13	2.74E-12
ARHGEF5	0.224226135	1.30E-05	4.21E-05
ARHGEF6	0.0739125	0.155376314	0.210629214
ARHGEF7	0.071257103	0.170805942	0.228790155
ARHGEF9	-0.151691554	0.003401714	0.006954227
ARID1A	-0.041072462	0.430243664	0.506608799
ARID1B	0.035861279	0.491057622	0.565155976
ARID2	0.095985459	0.064770628	0.09806661
ARID3A	0.411169347	1.44E-16	2.71E-15
ARID3B	0.223747903	1.36E-05	4.38E-05
ARID3C	-0.306768958	1.59E-09	9.95E-09
ARID4A	-0.222239599	1.56E-05	4.96E-05
ARID4B	-0.043126342	0.407526489	0.483748002
ARID5A	0.157601003	0.002331345	0.004950443
ARID5B	0.235167696	4.68E-06	1.64E-05
ARIH1	-0.230099317	7.56E-06	2.56E-05
ARIH2	0.251793793	8.97E-07	3.55E-06
ARL10	0.031936115	0.539735054	0.611499189
ARL11	0.261087816	3.38E-07	1.44E-06
ARL13A	0.087281871	0.093210635	0.135060169
ARL13B	0.06997665	0.178642566	0.237673114
ARL14	0.400100075	1.08E-15	1.76E-14
ARL15	-0.080960655	0.119540193	0.167899022
ARL16	0.230816135	7.07E-06	2.40E-05
ARL17A	0.226882896	1.02E-05	3.36E-05
ARL17B	-0.010513291	0.840055466	0.873328028
ARL1	0.242361972	2.32E-06	8.58E-06
ARL2BP	-0.002698474	0.958687277	0.967526203
ARL2	0.381390897	2.72E-14	3.56E-13
ARL3	-0.026502635	0.610861861	0.676689835
ARL4A	0.093797031	0.071146383	0.106449737
ARL4C	0.339406464	1.87E-11	1.57E-10
ARL4D	-0.10889478	0.036027031	0.05816764
ARL5A	-0.021323773	0.682256832	0.740462007
ARL5B	0.003144498	0.951866469	0.962690821
ARL5C	0.044204139	0.395895904	0.472249458
ARL6IP1	-0.051064781	0.326642974	0.400725495
ARL6IP4	0.063032372	0.22581992	0.291415391
ARL6IP5	-0.078295235	0.13224817	0.183168362
ARL6IP6	0.26392538	2.49E-07	1.09E-06
ARL6	-0.039630284	0.446623428	0.522345423

ARL8A	-0.000953854	0.985391127	0.988865708
ARL8B	0.167512285	0.001201435	0.002701466
ARL9	0.256295555	5.62E-07	2.30E-06
ARMC10	-0.087370577	0.092877168	0.134616147
ARMC1	0.022165412	0.670437553	0.730779501
ARMC2	0.250867757	9.87E-07	3.89E-06
ARMC3	0.270791919	1.17E-07	5.40E-07
ARMC4	0.20025937	0.000102894	0.000283247
ARMC5	-0.342680113	1.16E-11	1.01E-10
ARMC6	-0.053176114	0.307012467	0.379635179
ARMC7	0.246243598	1.58E-06	5.99E-06
ARMC8	0.120873654	0.019865453	0.034120144
ARMC9	0.304474599	2.13E-09	1.31E-08
ARMCX1	0.230474359	7.30E-06	2.47E-05
ARMCX2	0.222385258	1.54E-05	4.90E-05
ARMCX3	0.227012145	1.01E-05	3.33E-05
ARMCX5	-0.045062012	0.386782831	0.463097997
ARMCX6	0.289898428	1.29E-08	7.01E-08
ARMS2	-0.043888428	0.399281911	0.475514175
ARNT2	0.456743998	1.61E-20	5.29E-19
ARNTL2	0.40369026	5.65E-16	9.70E-15
ARNTL	-0.080419465	0.122040205	0.170988463
ARNT	-0.182694519	0.00040488	0.000996174
ARPC1A	0.1514303	0.003457983	0.007057596
ARPC1B	0.400897031	9.34E-16	1.54E-14
ARPC2	0.438876118	6.72E-19	1.78E-17
ARPC3	0.486042864	2.16E-23	1.08E-21
ARPC4	0.306195032	1.71E-09	1.07E-08
ARPC5L	0.320428715	2.64E-10	1.86E-09
ARPC5	0.198187313	0.000121709	0.000330743
ARPM1	0.022892497	0.660291833	0.721932599
ARPP19	0.076604285	0.140835089	0.1934977
ARPP21	-0.167397563	0.001210945	0.002719474
ARR3	0.12759314	0.013917563	0.024780202
ARRB1	0.300743666	3.41E-09	2.03E-08
ARRB2	0.337164963	2.58E-11	2.13E-10
ARRDC1	0.132589207	0.010572284	0.019385278
ARRDC2	0.237894016	3.60E-06	1.29E-05
ARRDC3	-0.041860295	0.421444433	0.497600663
ARRDC4	-0.129172104	0.012771617	0.022955265
ARRDC5	0.175115795	0.000704555	0.001659314
ARSA	-0.340787133	1.53E-11	1.31E-10
ARSB	-0.065908738	0.205303507	0.268499735
ARSD	-0.457974909	1.23E-20	4.13E-19
ARSE	-0.138016343	0.007764018	0.014680598
ARSF	0.087429443	0.092656401	0.134354817
ARSG	-0.136312722	0.008563875	0.01603021
ARSH	0.021796764	0.675604751	0.734683288
ARSI	0.322909141	1.89E-10	1.36E-09
ARSJ	0.191684423	0.000203895	0.000531396
ARSK	-0.105889871	0.041506496	0.066113592
ART1	0.043579516	0.402611739	0.478798416
ART3	0.159850167	0.00201221	0.004327674
ART4	-0.138849416	0.007397718	0.014042624
ART5	0.324854869	1.45E-10	1.06E-09
ARTN	0.306172353	1.72E-09	1.07E-08
ARV1	0.064042033	0.218458388	0.283415473
ARVCF	-0.065707341	0.206694532	0.2699999

ARX	0.152736743	0.003184811	0.006556558
AR	-0.485476179	2.46E-23	1.22E-21
AS3MT	-0.07034865	0.176338888	0.235126385
ASAH1	0.254455827	6.81E-07	2.76E-06
ASAH2B	-0.086086459	0.097798467	0.140918635
ASAH2	-0.203817013	7.68E-05	0.000216602
ASAM	0.179955651	0.000495885	0.001201242
ASAP1IT1	0.072516436	0.163351575	0.220033136
ASAP1	0.377231938	5.43E-14	6.82E-13
ASAP2	-0.034677014	0.505492386	0.57863364
ASAP3	-0.039329605	0.450082424	0.525835212
ASB10	-0.005229966	0.92002914	0.937487623
ASB11	0.061705505	0.235760006	0.302532099
ASB12	0.091757154	0.0775421	0.114863091
ASB13	-0.285363603	2.21E-08	1.16E-07
ASB14	0.178190863	0.000564226	0.001353462
ASB15	-0.072624124	0.162725651	0.219353165
ASB16	0.211364503	4.06E-05	0.000120638
ASB17	0.004805146	0.926505885	0.942640838
ASB18	0.118908261	0.021978521	0.037417202
ASB1	0.232886045	5.81E-06	2.01E-05
ASB2	0.283782944	2.66E-08	1.37E-07
ASB3	0.003052615	0.953271319	0.963734912
ASB4	-0.472460419	5.01E-22	2.05E-20
ASB5	0.119172171	0.021683914	0.036975685
ASB6	0.259059248	4.20E-07	1.76E-06
ASB7	-0.172387763	0.000855411	0.001980538
ASB8	-0.184286849	0.000359379	0.000893381
ASB9	0.040817374	0.43311531	0.50914753
ASCC1	0.011166247	0.830267888	0.865229736
ASCC2	-0.11243224	0.030375476	0.049995062
ASCC3	0.180179695	0.000487781	0.001182762
ASCL1	-0.099105732	0.056498947	0.086957047
ASCL2	0.288686339	1.49E-08	8.01E-08
ASCL3	0.152475112	0.003237895	0.00664935
ASCL4	0.081342887	0.117798685	0.165803689
ASF1A	0.083878625	0.106742744	0.15217754
ASF1B	0.56595082	8.42E-33	2.21E-30
ASFMR1	-0.113492186	0.028837297	0.047755331
ASGR1	-0.410050432	1.77E-16	3.29E-15
ASGR2	-0.234723109	4.88E-06	1.71E-05
ASH1L	0.003107133	0.952437737	0.963100615
ASH2L	-0.00675617	0.896807445	0.918759535
ASIP	-0.117297765	0.023852575	0.040297743
ASL	-0.236830917	3.99E-06	1.42E-05
ASMTL	-0.374741672	8.17E-14	9.96E-13
ASMT	0.189242931	0.000246417	0.000631561
ASNA1	0.027091894	0.602951146	0.669527492
ASNSD1	-0.246983428	1.46E-06	5.59E-06
ASNS	0.387170514	1.03E-14	1.43E-13
ASPA	-0.247271295	1.42E-06	5.44E-06
ASPDH	-0.483520796	3.91E-23	1.87E-21
ASPG	0.090801071	0.080696326	0.118964139
ASPHD1	0.233954541	5.25E-06	1.83E-05
ASPHD2	0.333091286	4.61E-11	3.66E-10
ASPH	0.110486763	0.033381769	0.054403763
ASPM	0.458871722	1.02E-20	3.43E-19
ASPN	0.130109493	0.012131371	0.021935122

ASPRV1	-0.315204961	5.31E-10	3.57E-09
ASPSCR1	-0.298048366	4.77E-09	2.77E-08
ASRGL1	0.476426313	2.03E-22	8.88E-21
ASS1	-0.222371158	1.54E-05	4.91E-05
ASTE1	-0.058336781	0.262369281	0.331973376
ASTL	0.165060808	0.001420374	0.00315143
ASTN1	-0.320242334	2.71E-10	1.91E-09
ASTN2	-0.298432486	4.55E-09	2.65E-08
ASXL1	0.216319326	2.64E-05	8.08E-05
ASXL2	-0.092119738	0.076372366	0.113315728
ASXL3	-0.138019896	0.007762422	0.014678973
ASZ1	0.024624161	0.636380113	0.70051744
ATAD1	-0.110277041	0.033720494	0.054861549
ATAD2B	0.070715959	0.174085975	0.232495863
ATAD2	0.377742263	4.99E-14	6.28E-13
ATAD3A	0.146939231	0.004566693	0.009076889
ATAD3B	0.304973905	2.00E-09	1.23E-08
ATAD3C	-0.004151696	0.936477607	0.951076108
ATAD5	0.442767543	3.04E-19	8.42E-18
ATCAY	0.08905882	0.086710562	0.126747951
ATE1	-0.152763693	0.003179388	0.006548777
ATF1	0.001013543	0.984477059	0.98814752
ATF2	-0.081188376	0.118500257	0.166638111
ATF3	-0.174919143	0.000714546	0.00168046
ATF4	0.294710072	7.19E-09	4.08E-08
ATF5	-0.026953004	0.604811583	0.671069694
ATF6B	0.075993523	0.144039466	0.197193117
ATF6	-0.151032939	0.003545184	0.007216426
ATF7IP2	-0.236082229	4.28E-06	1.52E-05
ATF7IP	-0.058453105	0.261417336	0.330958068
ATF7	0.012948348	0.803692181	0.843002982
ATG10	0.031768107	0.541871031	0.613366247
ATG12	0.135546538	0.008946934	0.01668609
ATG16L1	-0.070900914	0.172959677	0.231239694
ATG16L2	0.107363186	0.038737872	0.062081561
ATG2A	-0.261023121	3.41E-07	1.45E-06
ATG2B	-0.097500492	0.060637839	0.092476999
ATG3	0.273104769	9.06E-08	4.24E-07
ATG4A	-0.153686347	0.002998711	0.006207432
ATG4B	0.243134379	2.15E-06	7.98E-06
ATG4C	-0.292473378	9.44E-09	5.25E-08
ATG4D	0.332668624	4.89E-11	3.87E-10
ATG5	-0.021203864	0.683947148	0.741932757
ATG7	0.263112497	2.72E-07	1.18E-06
ATG9A	-0.072424989	0.16388451	0.220661476
ATG9B	0.383629262	1.87E-14	2.50E-13
ATHL1	-0.000323121	0.995050952	0.996301205
ATIC	0.332010965	5.37E-11	4.22E-10
ATL1	-0.020602912	0.692442217	0.74926198
ATL2	-0.102336472	0.048877123	0.076545087
ATL3	0.085782338	0.098993863	0.142436123
ATMIN	-0.312274013	7.80E-10	5.11E-09
ATM	0.032946965	0.526972904	0.598947638
ATN1	0.338411781	2.16E-11	1.80E-10
ATOH1	0.164254645	0.001500016	0.003308238
ATOH7	-0.09599798	0.064735548	0.098020946
ATOH8	-0.325095388	1.40E-10	1.03E-09
ATOX1	-0.199170836	0.000112408	0.00030744

ATP10A	0.227967761	9.22E-06	3.07E-05
ATP10B	0.325608792	1.31E-10	9.66E-10
ATP10D	0.165021735	0.001424143	0.003159088
ATP11A	0.224040396	1.32E-05	4.28E-05
ATP11B	0.115707761	0.025836625	0.043289927
ATP11C	-0.342379122	1.22E-11	1.06E-10
ATP12A	0.129489241	0.012551752	0.022619268
ATP13A1	0.365324958	3.71E-13	4.08E-12
ATP13A2	0.420985843	2.28E-17	4.83E-16
ATP13A3	-0.178827735	0.000538614	0.001296709
ATP13A4	0.104877061	0.043505033	0.068884698
ATP13A5	0.059325461	0.254354114	0.323124772
ATP1A1	0.498435976	1.08E-24	6.66E-23
ATP1A2	-0.154981059	0.00276089	0.005758816
ATP1A3	0.25347018	7.55E-07	3.03E-06
ATP1A4	0.223999391	1.33E-05	4.29E-05
ATP1B1	0.163936821	0.001532525	0.003372096
ATP1B2	0.085640624	0.099554843	0.143149158
ATP1B3	0.42469071	1.12E-17	2.46E-16
ATP1B4	0.055129057	0.289561996	0.361127713
ATP2A1	0.04863401	0.350227055	0.424922253
ATP2A2	0.171334495	0.00092125	0.002115635
ATP2A3	0.273366524	8.80E-08	4.13E-07
ATP2B1	0.26903153	1.43E-07	6.47E-07
ATP2B2	-0.050295756	0.333990573	0.407916999
ATP2B3	-0.044970272	0.387751247	0.464034381
ATP2B4	0.088023501	0.090451913	0.131512408
ATP2C1	0.080172366	0.123195126	0.172400485
ATP2C2	0.335272411	3.38E-11	2.74E-10
ATP4A	0.221005084	1.74E-05	5.50E-05
ATP4B	0.017340012	0.739217169	0.788873175
ATP5A1	0.016289775	0.754489461	0.80160287
ATP5B	0.069198116	0.183535803	0.243176395
ATP5C1	0.256421681	5.55E-07	2.28E-06
ATP5D	-0.040040711	0.441926303	0.517642039
ATP5EP2	0.184081568	0.000364965	0.000905911
ATP5E	0.202273868	8.73E-05	0.000243689
ATP5F1	-0.017107334	0.742592145	0.791838608
ATP5G1	0.182280998	0.000417543	0.001024169
ATP5G2	0.320091315	2.77E-10	1.95E-09
ATP5G3	0.127318667	0.014125731	0.025103731
ATP5H	0.059819012	0.250417075	0.318631217
ATP5I	0.096460019	0.063451905	0.09631887
ATP5J2	0.138705128	0.007460034	0.014147435
ATP5J	-0.020119406	0.699305226	0.755261775
ATP5L2	0.049126103	0.345367779	0.419793588
ATP5L	0.16941539	0.00105337	0.002393116
ATP5O	0.111167197	0.032302683	0.052835307
ATP5SL	0.260003789	3.80E-07	1.60E-06
ATP5S	-0.248389549	1.27E-06	4.91E-06
ATP6AP1L	-0.085047026	0.101932129	0.146229703
ATP6AP1	0.178819756	0.000538928	0.001297309
ATP6AP2	0.232688219	5.92E-06	2.05E-05
ATP6V0A1	-0.165661887	0.00136354	0.0030331
ATP6V0A2	0.166276561	0.001307587	0.002916779
ATP6V0A4	0.216255833	2.66E-05	8.12E-05
ATP6V0B	0.261072189	3.39E-07	1.45E-06
ATP6V0C	0.081150434	0.118673037	0.166833974

ATP6V0D1	-0.046822972	0.368480057	0.444308176
ATP6V0D2	0.40018539	1.06E-15	1.73E-14
ATP6V0E1	0.085913108	0.098478431	0.141785597
ATP6V0E2	-0.431327996	3.05E-18	7.30E-17
ATP6V1A	0.001545812	0.976327062	0.981450587
ATP6V1B1	0.231531401	6.61E-06	2.26E-05
ATP6V1B2	0.058315268	0.262545591	0.332112088
ATP6V1C1	0.253768725	7.32E-07	2.94E-06
ATP6V1C2	0.292533603	9.38E-09	5.21E-08
ATP6V1D	0.040037301	0.44196521	0.517657176
ATP6V1E1	0.197499628	0.000128636	0.000347812
ATP6V1E2	0.022719653	0.662698156	0.723927875
ATP6V1F	0.340944541	1.50E-11	1.28E-10
ATP6V1G1	-0.07808559	0.13329031	0.184467493
ATP6V1G2	0.163962819	0.001529841	0.003366564
ATP6V1G3	0.063513774	0.222288267	0.287728339
ATP6V1H	0.219068311	2.07E-05	6.45E-05
ATP7A	0.186974961	0.000293212	0.000740633
ATP7B	-0.248438855	1.26E-06	4.89E-06
ATP8A1	0.116980081	0.024238119	0.040872846
ATP8A2	0.277843627	5.30E-08	2.58E-07
ATP8B1	0.030120418	0.563038846	0.633209872
ATP8B2	0.232412485	6.08E-06	2.09E-05
ATP8B3	0.093983736	0.070583167	0.105757962
ATP8B4	0.011590181	0.82392717	0.860166479
ATP8B5P	0.092245825	0.075968971	0.11275919
ATP9A	0.209608732	4.72E-05	0.000138379
ATP9B	0.073022105	0.160428084	0.216578457
ATPAF1	-0.344552943	8.84E-12	7.84E-11
ATPAF2	-0.071229894	0.170969757	0.228932618
ATPBD4	0.100018823	0.054251043	0.08387314
ATPIF1	-0.117854748	0.023189392	0.039320714
ATRIP	0.201596501	9.22E-05	0.0002563
ATRNL1	0.301552916	3.08E-09	1.84E-08
ATRN	-0.126188359	0.015012105	0.026508701
ATRX	-0.01014354	0.84560896	0.877864601
ATR	0.200017883	0.000104937	0.000288472
ATXN10	0.043173574	0.407012594	0.483310777
ATXN1L	-0.254111908	7.06E-07	2.85E-06
ATXN1	-0.143988806	0.005460136	0.01065082
ATXN2L	0.330929546	6.24E-11	4.87E-10
ATXN2	0.127877519	0.01370473	0.024458087
ATXN3L	-0.014110234	0.78648436	0.828925056
ATXN3	-0.247178109	1.44E-06	5.49E-06
ATXN7L1	-0.432003378	2.67E-18	6.44E-17
ATXN7L2	0.210173665	4.50E-05	0.000132662
ATXN7L3B	-0.063201981	0.224571114	0.29021184
ATXN7L3	0.302203252	2.84E-09	1.71E-08
ATXN7	-0.093021433	0.073525461	0.109614175
AUH	-0.278020827	5.19E-08	2.54E-07
AUP1	0.166657492	0.001273979	0.002849552
AURKAIP1	-0.07294667	0.160861684	0.21711968
AURKAPS1	0.510713942	4.90E-26	3.87E-24
AURKA	0.493810026	3.34E-24	1.89E-22
AURKB	0.580458172	8.50E-35	4.03E-32
AURKC	0.025150458	0.629185408	0.693862812
AUTS2	-0.16820021	0.001145834	0.002587837
AVEN	-0.13186634	0.011007339	0.020092378

AVIL	-0.095317757	0.066664119	0.100589456
AVL9	-0.051766836	0.320027282	0.393603131
AVPI1	-0.236248331	4.22E-06	1.49E-05
AVPR1A	-0.152829927	0.003166096	0.006524097
AVPR1B	0.20994113	4.59E-05	0.000134849
AVPR2	-0.186764647	0.000297948	0.000751451
AVP	0.106282282	0.040753257	0.065049787
AWAT1	0.135858234	0.008789298	0.01641361
AWAT2	0.154872712	0.002780113	0.005795879
AXIN1	0.300634545	3.46E-09	2.05E-08
AXIN2	-0.205594473	6.63E-05	0.000189171
AXL	0.203478793	7.90E-05	0.000222219
AZGP1	-0.542438794	9.08E-30	1.23E-27
AZI1	0.386882972	1.08E-14	1.50E-13
AZI2	0.028864126	0.579440803	0.648335843
AZIN1	0.136937251	0.008262514	0.015521528
AZU1	0.117280909	0.023872898	0.04032182
B2M	-0.036504703	0.483308509	0.557493609
B3GALNT1	0.346828069	6.32E-12	5.74E-11
B3GALNT2	0.171862907	0.000887656	0.002047457
B3GALT1	-0.204071836	7.52E-05	0.000212506
B3GALT2	0.209312529	4.84E-05	0.000141663
B3GALT4	0.233237221	5.62E-06	1.95E-05
B3GALT5	0.309519817	1.12E-09	7.15E-09
B3GALT6	0.002828192	0.956703236	0.966013273
B3GALTL	0.053525564	0.303840054	0.37648349
B3GAT1	0.259874901	3.85E-07	1.62E-06
B3GAT2	0.135101699	0.00917626	0.01708008
B3GAT3	0.207072277	5.85E-05	0.000168735
B3GNT1	-0.240777813	2.72E-06	9.94E-06
B3GNT2	0.092951393	0.073743455	0.109889828
B3GNT3	0.380929233	2.94E-14	3.83E-13
B3GNT4	0.305830026	1.79E-09	1.11E-08
B3GNT5	0.313595836	6.56E-10	4.35E-09
B3GNT6	0.061404935	0.238053884	0.305161153
B3GNT7	0.35223745	2.81E-12	2.69E-11
B3GNT8	0.329249022	7.90E-11	6.06E-10
B3GNT9	0.269833325	1.31E-07	5.96E-07
B3GNTL1	0.318374017	3.48E-10	2.41E-09
B4GALNT1	0.160563721	0.001919636	0.004143337
B4GALNT2	0.385406112	1.38E-14	1.89E-13
B4GALNT3	0.114184643	0.027868892	0.046297871
B4GALNT4	0.368789482	2.14E-13	2.45E-12
B4GALT1	0.026312404	0.613425448	0.679012484
B4GALT2	0.170628221	0.000967984	0.002215554
B4GALT3	0.209416999	4.80E-05	0.000140512
B4GALT4	0.32137401	2.33E-10	1.65E-09
B4GALT5	0.330222244	6.89E-11	5.35E-10
B4GALT6	0.022982848	0.659035364	0.720853228
B4GALT7	0.11301782	0.029517232	0.048743559
B9D1	0.169459452	0.00105015	0.002387435
B9D2	0.140057067	0.006894117	0.013178333
BAALC	-0.044338685	0.394458159	0.470816335
BAAT	-0.448324405	9.60E-20	2.83E-18
BACE1	-0.325988788	1.24E-10	9.20E-10
BACE2	0.328836328	8.36E-11	6.39E-10
BACH1	0.018418026	0.723646847	0.775832809
BACH2	-0.116410808	0.024942465	0.041925733

BAD	0.093438083	0.072239492	0.107915054
BAG1	-0.068056151	0.190891141	0.251749855
BAG2	0.109983751	0.034199096	0.055554011
BAG3	0.186173386	0.000311646	0.000783915
BAG4	-0.076507457	0.141339426	0.194110302
BAG5	0.03528966	0.497997302	0.571887379
BAGE2	-0.045894517	0.378062216	0.454240122
BAGE	0.044805895	0.389490087	0.465947368
BAHCC1	0.02423879	0.641670111	0.705327299
BAHD1	0.003285614	0.949709167	0.960948043
BAI1	0.224889518	1.22E-05	3.98E-05
BAI2	0.441889065	3.64E-19	9.93E-18
BAI3	0.080215663	0.122992145	0.172140615
BAIAP2L1	0.27269104	9.49E-08	4.43E-07
BAIAP2L2	0.371567007	1.37E-13	1.61E-12
BAIAP2	-0.329761493	7.35E-11	5.69E-10
BAIAP3	-0.289122031	1.41E-08	7.65E-08
BAK1	0.594591962	7.71E-37	6.40E-34
BAMBI	0.100667163	0.052700458	0.081760597
BANF1	0.234085105	5.19E-06	1.81E-05
BANF2	0.017134254	0.742201417	0.791506698
BANK1	0.238474795	3.40E-06	1.22E-05
BANP	-0.165006519	0.001425613	0.003161702
BAP1	-0.140834328	0.006586438	0.012650889
BARD1	0.206454732	6.16E-05	0.00017706
BARHL1	0.096831005	0.062436455	0.094914526
BARHL2	0.173789804	0.000774505	0.001808662
BARX1	0.265383498	2.13E-07	9.40E-07
BARX2	0.220606018	1.80E-05	5.69E-05
BASE	0.305340301	1.91E-09	1.18E-08
BASP1	0.279729325	4.26E-08	2.12E-07
BAT1	0.171127913	0.000934699	0.002145038
BAT2L1	0.093171116	0.073061345	0.109028323
BAT2L2	0.222431096	1.53E-05	4.88E-05
BAT2	0.247455259	1.40E-06	5.35E-06
BAT3	0.107588475	0.038328608	0.061485025
BAT4	-0.111079798	0.0324396	0.053024426
BAT5	-0.038513577	0.45954576	0.534509028
BATF2	0.126599222	0.014684421	0.025999204
BATF3	0.278028282	5.18E-08	2.53E-07
BATF	0.21338651	3.41E-05	0.000102507
BAX	0.308616832	1.25E-09	7.97E-09
BAZ1A	0.192158754	0.000196474	0.0005134
BAZ1B	0.058444047	0.261491375	0.331030766
BAZ2A	0.336146682	2.99E-11	2.44E-10
BAZ2B	-0.153462702	0.003041628	0.006285821
BBC3	0.277965554	5.22E-08	2.55E-07
BBOX1	-0.188743096	0.000256087	0.000653905
BBS10	-0.192309398	0.000194171	0.000507847
BBS12	0.10598511	0.041322613	0.065868867
BBS1	-0.249985107	1.08E-06	4.23E-06
BBS2	-0.219947658	1.91E-05	6.00E-05
BBS4	-0.280386662	3.95E-08	1.98E-07
BBS5	0.057744339	0.267254561	0.336998884
BBS7	0.169221508	0.001067646	0.002423891
BBS9	-0.226658826	1.04E-05	3.43E-05
BBX	0.108021955	0.037551434	0.060384186
BCAM	0.100070505	0.054126065	0.083686415

BCAN	0.178481104	0.000552417	0.001327213
BCAP29	-0.058145829	0.263937121	0.333470023
BCAP31	-0.001813944	0.972222357	0.978114018
BCAR1	0.092731425	0.074431515	0.11074721
BCAR3	0.080801914	0.120269315	0.168792201
BCAR4	0.024461857	0.638605843	0.702695698
BCAS1	0.367886557	2.47E-13	2.80E-12
BCAS2	0.017198391	0.741270778	0.790725875
BCAS3	-0.471536636	6.17E-22	2.48E-20
BCAS4	0.390871485	5.43E-15	7.95E-14
BCAT1	0.399154386	1.27E-15	2.05E-14
BCAT2	0.202135455	8.83E-05	0.000246237
BCCIP	0.12143482	0.019295515	0.033235798
BCDIN3D	-0.124810563	0.016158257	0.028314257
BCHE	-0.225364905	1.17E-05	3.82E-05
BCKDHA	-0.421236133	2.17E-17	4.61E-16
BCKDHB	-0.433900771	1.83E-18	4.56E-17
BCKDK	-0.278033468	5.18E-08	2.53E-07
BCL10	0.23195415	6.35E-06	2.18E-05
BCL11A	0.350763323	3.51E-12	3.31E-11
BCL11B	0.268856692	1.45E-07	6.59E-07
BCL2A1	0.305383234	1.90E-09	1.17E-08
BCL2L10	-0.042556331	0.413758672	0.489861548
BCL2L11	0.214110939	3.20E-05	9.66E-05
BCL2L12	0.248713331	1.23E-06	4.76E-06
BCL2L13	-0.225503385	1.16E-05	3.78E-05
BCL2L14	0.291866339	1.02E-08	5.61E-08
BCL2L15	0.169448777	0.001050929	0.002388933
BCL2L1	-0.147141629	0.004510532	0.008974216
BCL2L2	0.150934537	0.003567085	0.00725878
BCL2	0.082269829	0.113657745	0.160713294
BCL3	0.201976115	8.94E-05	0.000249029
BCL6B	-0.012739081	0.806801829	0.845552424
BCL6	-0.201207909	9.52E-05	0.000264037
BCL7A	0.211320112	4.08E-05	0.000121063
BCL7B	-0.026780169	0.607130302	0.673005223
BCL7C	0.214250267	3.17E-05	9.56E-05
BCL8	0.036243947	0.486440942	0.560555093
BCL9L	0.138585858	0.007511898	0.014239014
BCL9	0.310330468	1.00E-09	6.49E-09
BCLAF1	0.080727674	0.1206115	0.169236675
BCMO1	0.091175711	0.079448199	0.117415951
BCO2	-0.325882492	1.26E-10	9.32E-10
BCORL1	0.241443706	2.54E-06	9.34E-06
BCORL2	0.086841854	0.09487894	0.137178187
BCOR	0.160800029	0.001889848	0.004087456
BCR	0.113203585	0.029249345	0.048341252
BCS1L	-0.117540101	0.023562046	0.039871062
BCYRN1	-0.050507716	0.331954892	0.406036754
BDH1	-0.460318636	7.43E-21	2.58E-19
BDH2	-0.177663804	0.000586271	0.001402965
BDKRB1	0.227853764	9.32E-06	3.10E-05
BDKRB2	0.084160724	0.105565167	0.150725239
BDNFOS	-0.362240459	6.04E-13	6.40E-12
BDNF	0.063105845	0.225278341	0.290880492
BDP1	0.089519622	0.08508587	0.124646295
BEAN	0.141028856	0.006511384	0.012517591
BECN1	0.076089315	0.143533239	0.196621687

BEGAIN	0.202264543	8.73E-05	0.000243842
BEND2	0.013183638	0.800199524	0.839914379
BEND3	0.497205735	1.46E-24	8.70E-23
BEND4	0.145453576	0.004998615	0.009849891
BEND5	0.067248198	0.196224122	0.258031482
BEND6	0.277370022	5.59E-08	2.72E-07
BEND7	0.017942392	0.730503079	0.78168177
BEST1	0.415315403	6.66E-17	1.32E-15
BEST2	0.122771664	0.017994781	0.0312085
BEST3	0.1546003	0.002828985	0.005887905
BEST4	0.096877395	0.062310424	0.09475941
BET1L	0.096111518	0.064418153	0.097592277
BET1	-0.184324203	0.000358372	0.00089121
BET3L	0.099552378	0.055389903	0.085434937
BEX1	0.162119628	0.001731138	0.003765858
BEX2	0.412029982	1.23E-16	2.34E-15
BEX4	0.326243388	1.20E-10	8.90E-10
BEX5	0.168482173	0.001123737	0.002541836
BEYLA	0.073019577	0.160442597	0.216583373
BFAR	0.166395215	0.001297033	0.002896154
BFSP1	0.282218383	3.20E-08	1.63E-07
BFSP2	0.338903033	2.01E-11	1.69E-10
BGLAP	0.142541433	0.00595345	0.011526203
BGN	0.208916065	5.01E-05	0.000146154
BHLHA15	0.215999895	2.72E-05	8.28E-05
BHLHB9	-0.203088146	8.16E-05	0.000228866
BHLHE22	0.081847208	0.115531342	0.162991065
BHLHE40	-0.13223751	0.010782012	0.019731696
BHLHE41	0.223562268	1.38E-05	4.44E-05
BHMT2	-0.34948857	4.25E-12	3.97E-11
BHMT	-0.343861581	9.78E-12	8.62E-11
BICC1	0.20821887	5.31E-05	0.00015424
BICD1	0.309998075	1.05E-09	6.75E-09
BICD2	0.197734533	0.000126229	0.000342094
BID	0.288206247	1.58E-08	8.43E-08
BIK	0.144801484	0.005199554	0.010196022
BIN1	0.259389006	4.06E-07	1.70E-06
BIN2	0.20657856	6.10E-05	0.000175355
BIN3	0.066321272	0.20247536	0.265306112
BIRC2	-0.040947692	0.431646876	0.507810857
BIRC3	0.301316742	3.17E-09	1.90E-08
BIRC5	0.536562892	4.78E-29	5.88E-27
BIRC6	-0.048138481	0.355163667	0.430099725
BIRC7	0.306046207	1.74E-09	1.09E-08
BIRC8	0.049071236	0.345907449	0.420346968
BIVM	-0.130238866	0.01204527	0.021795265
BLCAP	-0.401111701	8.98E-16	1.49E-14
BLID	-0.133806205	0.009874167	0.018254748
BLK	-0.004352307	0.933415126	0.948314353
BLMH	0.306496886	1.65E-09	1.03E-08
BLM	0.584909019	1.98E-35	1.20E-32
BLNK	0.073931305	0.155270938	0.210515015
BLOC1S1	-0.070963294	0.17258104	0.230826417
BLOC1S2	0.038961025	0.454343094	0.529739139
BLOC1S3	0.299429973	4.02E-09	2.36E-08
BLVRA	0.490287535	7.83E-24	4.14E-22
BLVRB	-0.307744522	1.40E-09	8.87E-09
BLZF1	0.182007493	0.00042612	0.00104328

BMF	0.304510572	2.12E-09	1.30E-08
BMI1	-0.120019916	0.020760603	0.035544236
BMP10	-0.109014665	0.035821813	0.057892606
BMP15	0.02172144	0.67666243	0.735512272
BMP1	-0.254548988	6.75E-07	2.73E-06
BMP2K	0.11759274	0.023499344	0.039778565
BMP2	0.137778525	0.007871514	0.014862697
BMP3	0.093671294	0.071527744	0.106988191
BMP4	-0.106306638	0.040706888	0.064986186
BMP5	0.11440148	0.027571444	0.045857263
BMP6	-0.011522384	0.824940437	0.86102915
BMP7	0.276012556	6.53E-08	3.13E-07
BMP8A	0.333789698	4.18E-11	3.34E-10
BMP8B	0.256392384	5.57E-07	2.28E-06
BMPER	-0.058284185	0.262800484	0.332376607
BMPR1A	0.187542843	0.000280774	0.000712285
BMPR1B	0.301116039	3.25E-09	1.94E-08
BMPR2	-0.127938251	0.01365965	0.024384189
BMS1P4	-0.056627777	0.276630201	0.347063849
BMS1P5	-0.014135907	0.786105277	0.828788597
BMS1	0.261759939	3.15E-07	1.35E-06
BMX	-0.097186488	0.061476031	0.093604823
BNC1	0.087523511	0.092304488	0.133922512
BNC2	0.188808868	0.000254795	0.000651022
BNIP1	0.319620127	2.95E-10	2.06E-09
BNIP2	-0.068722947	0.186570527	0.246705034
BNIP3L	0.034156262	0.511909446	0.584595011
BNIP3	-0.346703541	6.43E-12	5.84E-11
BNIPL	0.050578222	0.331279522	0.405290508
BOC	-0.092666219	0.07463648	0.111004701
BOD1L	-0.033383821	0.521505383	0.593749586
BOD1	0.25843931	4.49E-07	1.87E-06
BOK	-0.467013264	1.70E-21	6.42E-20
BOLA1	-0.081199524	0.118449529	0.166578535
BOLA2	0.432001733	2.67E-18	6.44E-17
BOLA3	0.304024145	2.26E-09	1.38E-08
BOLL	0.184667442	0.000349233	0.000870222
BOP1	0.16643303	0.001293685	0.002889327
BPESC1	0.107582801	0.03833887	0.061496535
BPGM	0.100961409	0.052009	0.080776744
BPHL	-0.44077914	4.57E-19	1.24E-17
BPIL1	0.214363687	3.13E-05	9.47E-05
BPIL2	0.066554303	0.200890352	0.263506557
BPIL3	0.148120451	0.004247628	0.008500376
BPI	-0.090620846	0.081302379	0.119736448
BPNT1	0.090963546	0.080153101	0.118326053
BPTF	0.063191219	0.224650208	0.290257568
BPY2	0.049626291	0.340472669	0.414729227
BRAF	0.056272555	0.279659217	0.350334585
BRAP	0.301300898	3.18E-09	1.90E-08
BRCA1	0.363302294	5.11E-13	5.49E-12
BRCA2	0.274782645	7.50E-08	3.56E-07
BRCC3	0.143951953	0.005472226	0.010670225
BRD1	0.105940896	0.041407893	0.06599334
BRD2	-0.042126554	0.418494548	0.494498718
BRD3	0.139152092	0.007268505	0.013826331
BRD4	0.058879618	0.257947285	0.327147047
BRD7P3	0.067959851	0.191521142	0.252513845

BRD7	-0.082227201	0.113845639	0.160921868
BRD8	0.236742129	4.02E-06	1.43E-05
BRD9	0.292941793	8.92E-09	4.98E-08
BRDT	0.133574701	0.010003728	0.018465141
BREA2	0.187538914	0.000280858	0.000712408
BRE	0.086908449	0.094624926	0.136870527
BRF1	0.076743516	0.140112307	0.192650796
BRF2	0.099337196	0.055921933	0.086188824
BRI3BP	0.41620219	5.64E-17	1.13E-15
BRI3	0.013461227	0.796084084	0.837050041
BRIP1	0.130436765	0.011914608	0.021586287
BRIX1	0.449286093	7.85E-20	2.34E-18
BRMS1L	-0.032553934	0.531916707	0.603808811
BRMS1	0.300062145	3.71E-09	2.20E-08
BRP44L	-0.092746353	0.074384656	0.110717736
BRP44	0.230833435	7.06E-06	2.40E-05
BRPF1	0.344850255	8.46E-12	7.54E-11
BRPF3	-0.07139017	0.17000649	0.227857192
BRS3	0.214278618	3.16E-05	9.54E-05
BRSK1	0.451239951	5.20E-20	1.57E-18
BRSK2	0.215501963	2.84E-05	8.63E-05
BRWD1	-0.18801822	0.000270742	0.000688941
BRWD3	-0.065797709	0.206069529	0.269360091
BSCL2	-0.096290855	0.063919427	0.096932543
BSDC1	-0.446828283	1.31E-19	3.80E-18
BSG	0.316019363	4.76E-10	3.23E-09
BSND	0.334983659	3.53E-11	2.85E-10
BSN	0.033890948	0.515195026	0.587859865
BSPRY	0.265582613	2.08E-07	9.21E-07
BST1	0.061386682	0.23819369	0.305320723
BST2	-0.020966219	0.687301813	0.744760768
BSX	0.008312229	0.873221926	0.900261043
BTAF1	-0.008285297	0.873629241	0.900454941
BTBD10	0.32642606	1.17E-10	8.70E-10
BTBD11	0.027246164	0.600887711	0.667645565
BTBD12	0.103723085	0.045880186	0.072289233
BTBD16	-0.024922424	0.632298481	0.696686161
BTBD17	0.021823048	0.675235827	0.734362275
BTBD18	0.028434081	0.585106273	0.653317295
BTBD19	-0.068157243	0.190231422	0.250978542
BTBD1	0.117045967	0.02415772	0.040754518
BTBD2	-0.034462812	0.508126812	0.581108056
BTBD3	0.297562544	5.06E-09	2.93E-08
BTBD6	-0.0023656	0.963779735	0.97143536
BTBD7	0.060531655	0.244807442	0.312531487
BTBD8	0.039372934	0.449583031	0.525375008
BTBD9	0.056047434	0.281590429	0.352421442
BTC	0.22433856	1.29E-05	4.16E-05
BTD	-0.396433306	2.06E-15	3.21E-14
BTF3L1	0.013236536	0.799414839	0.839533075
BTF3L4	0.232617336	5.96E-06	2.06E-05
BTF3	0.175514903	0.000684674	0.001616698
BTG1	0.295209887	6.77E-09	3.85E-08
BTG2	0.224229425	1.30E-05	4.21E-05
BTG3	0.221821748	1.62E-05	5.14E-05
BTG4	0.034536164	0.507223872	0.580208657
BTK	0.258226695	4.59E-07	1.91E-06
BTLA	0.120582068	0.020167335	0.034596886

BTN1A1	0.15230951	0.003271909	0.006710208
BTN2A1	0.232856418	5.83E-06	2.02E-05
BTN2A2	0.099220761	0.056211579	0.086568264
BTN2A3	0.092249943	0.075955826	0.11275919
BTN3A1	-0.087483707	0.092453266	0.134118835
BTN3A2	-0.078091573	0.13326048	0.18444794
BTN3A3	-0.101208244	0.0514348	0.080009689
BTNL2	0.12713018	0.014270267	0.025331217
BTNL3	0.029705983	0.568424937	0.638124738
BTNL8	0.212777707	3.60E-05	0.000107733
BTNL9	-0.459832807	8.25E-21	2.83E-19
BTRC	0.004050119	0.938028609	0.952193176
BUB1B	0.591770938	2.01E-36	1.48E-33
BUB1	0.574835242	5.19E-34	1.88E-31
BUB3	0.362807667	5.52E-13	5.90E-12
BUD13	0.113495321	0.028832848	0.047751932
BUD31	0.177116827	0.000609991	0.001453617
BVES	0.133726885	0.00991839	0.0183246
BYSL	0.148311571	0.004197939	0.00840516
BZRAP1	0.034943499	0.502224971	0.575614191
BZW1	-0.011215596	0.829529202	0.864686101
BZW2	0.234657897	4.91E-06	1.72E-05
C10orf105	-0.34523653	7.99E-12	7.15E-11
C10orf107	0.067024901	0.197717015	0.25972032
C10orf108	0.07906355	0.128482569	0.17872029
C10orf10	-0.053857133	0.300850099	0.373266219
C10orf110	0.022722515	0.662658274	0.723924003
C10orf111	0.142924313	0.005819203	0.011286037
C10orf113	0.127085448	0.014304757	0.025385656
C10orf114	-0.133949355	0.009794801	0.01812484
C10orf116	-0.081634504	0.11648342	0.164172812
C10orf118	-0.045584058	0.381300117	0.457523395
C10orf119	0.264722856	2.29E-07	1.00E-06
C10orf11	-0.078722076	0.1301458	0.180693053
C10orf120	0.03784185	0.467418209	0.542304209
C10orf122	0.003682723	0.943640405	0.956507792
C10orf125	-0.39291897	3.81E-15	5.72E-14
C10orf128	-0.051965833	0.318168051	0.391437282
C10orf129	-0.121168492	0.019564207	0.033660777
C10orf12	0.045897312	0.378033139	0.454232581
C10orf131	0.13649449	0.008475171	0.015879089
C10orf137	0.226091465	1.10E-05	3.60E-05
C10orf140	-0.099811773	0.054754146	0.084572189
C10orf18	0.186539019	0.000303107	0.000763981
C10orf25	-0.311292547	8.86E-10	5.78E-09
C10orf26	-0.355280181	1.77E-12	1.75E-11
C10orf27	0.02145	0.680479152	0.738975506
C10orf28	0.08934058	0.085714198	0.12547566
C10orf2	0.120458165	0.020296815	0.034810016
C10orf32	-0.201861002	9.03E-05	0.000251254
C10orf35	0.399122707	1.28E-15	2.06E-14
C10orf41	-0.202037826	8.90E-05	0.000247978
C10orf46	0.256428776	5.54E-07	2.27E-06
C10orf47	-0.177945534	0.000574389	0.00137602
C10orf4	-0.070822828	0.173434524	0.231750124
C10orf50	0.071816625	0.167463221	0.224841777
C10orf53	0.076549579	0.14111986	0.193848849
C10orf54	0.164369999	0.001488374	0.0032862

C10orf55	0.208093788	5.37E-05	0.000155746
C10orf57	-0.414046702	8.45E-17	1.65E-15
C10orf58	-0.322938993	1.88E-10	1.36E-09
C10orf62	-0.037263009	0.474261363	0.549060604
C10orf67	0.157692895	0.002317451	0.004922513
C10orf68	0.192485314	0.000191513	0.000501357
C10orf71	0.01849822	0.722492998	0.774927614
C10orf72	-0.360535244	7.88E-13	8.20E-12
C10orf75	-0.143657317	0.005569756	0.010845535
C10orf76	-0.071265752	0.170753897	0.228735821
C10orf78	0.409112043	2.11E-16	3.84E-15
C10orf79	-0.079779727	0.125047745	0.174625065
C10orf81	0.338713228	2.07E-11	1.73E-10
C10orf82	0.193837731	0.00017219	0.000454714
C10orf84	0.122335234	0.018410752	0.031874424
C10orf88	0.070946391	0.17268358	0.23093121
C10orf90	0.225902966	1.12E-05	3.65E-05
C10orf91	0.347695029	5.55E-12	5.07E-11
C10orf93	0.230231692	7.47E-06	2.53E-05
C10orf95	0.116505925	0.024823565	0.041768164
C10orf96	0.019305869	0.710907913	0.765055501
C10orf99	0.055766571	0.284012438	0.354984365
C11orf10	0.250281967	1.05E-06	4.11E-06
C11orf16	0.081753723	0.115949033	0.16348904
C11orf17	0.315284798	5.25E-10	3.53E-09
C11orf1	-0.332560232	4.97E-11	3.92E-10
C11orf20	0.170326168	0.000988631	0.002259176
C11orf21	0.082270437	0.113655068	0.160713294
C11orf24	-0.02148956	0.679922398	0.738555329
C11orf2	-0.148917999	0.004043734	0.008128269
C11orf30	0.024594224	0.636790404	0.700814187
C11orf31	-0.050079555	0.336075214	0.410049633
C11orf34	0.026997432	0.604216198	0.67055847
C11orf35	-0.242898648	2.20E-06	8.16E-06
C11orf36	0.180971304	0.000460123	0.001120472
C11orf41	0.354243662	2.07E-12	2.03E-11
C11orf42	-0.026467516	0.611334784	0.677176067
C11orf45	0.36564042	3.53E-13	3.90E-12
C11orf46	-0.030714137	0.55536574	0.626099264
C11orf48	0.295783332	6.31E-09	3.60E-08
C11orf49	0.198659729	0.000117154	0.000319501
C11orf51	0.110572419	0.03324426	0.054192958
C11orf52	-0.133845635	0.009852249	0.0182193
C11orf53	0.252500797	8.34E-07	3.32E-06
C11orf54	-0.389105622	7.36E-15	1.06E-13
C11orf57	-0.037738206	0.468639488	0.543499586
C11orf58	-0.063840762	0.219911999	0.284985809
C11orf59	0.146439466	0.004708078	0.00933394
C11orf61	0.205061394	6.93E-05	0.000196933
C11orf63	0.252449216	8.39E-07	3.34E-06
C11orf65	-0.278511645	4.91E-08	2.41E-07
C11orf66	-0.334777548	3.63E-11	2.92E-10
C11orf67	-0.145070624	0.005115755	0.010053869
C11orf68	-0.12650338	0.014760293	0.026103379
C11orf70	0.299088815	4.19E-09	2.46E-08
C11orf71	-0.520076372	4.26E-27	3.88E-25
C11orf73	0.121301466	0.019429648	0.033452376
C11orf74	-0.092838594	0.074095639	0.110332112

C11orf75	0.115480682	0.02613127	0.043717431
C11orf80	0.459546083	8.78E-21	3.00E-19
C11orf82	0.360009043	8.55E-13	8.83E-12
C11orf83	0.171078798	0.000937923	0.002151942
C11orf84	0.528535246	4.39E-28	4.70E-26
C11orf85	-0.035209179	0.498978539	0.572873181
C11orf86	0.16274652	0.001660102	0.003623198
C11orf87	-0.038916348	0.45486108	0.530236538
C11orf88	0.056003502	0.281968349	0.352783612
C11orf90	0.200136846	0.000103926	0.000285811
C11orf92	0.315259382	5.27E-10	3.54E-09
C11orf93	0.42549026	9.57E-18	2.13E-16
C11orf94	0.068230874	0.189751966	0.250380094
C11orf95	-0.165518441	0.00137691	0.003061474
C11orf9	0.221801081	1.62E-05	5.14E-05
C12orf10	0.13183897	0.011024118	0.020121162
C12orf11	0.183600764	0.000378367	0.00093591
C12orf12	-0.019341818	0.710393778	0.76462612
C12orf23	0.206966095	5.91E-05	0.000170102
C12orf24	0.152537888	0.003225085	0.006625093
C12orf26	0.096019892	0.064674193	0.097942933
C12orf27	0.186305189	0.000308542	0.000776795
C12orf29	-0.132827846	0.010432042	0.019159733
C12orf32	0.560034563	5.14E-32	1.11E-29
C12orf34	0.338797469	2.04E-11	1.71E-10
C12orf35	-0.271432908	1.09E-07	5.05E-07
C12orf36	0.240444111	2.81E-06	1.02E-05
C12orf39	0.010342478	0.842620031	0.875535856
C12orf40	0.077042407	0.138570301	0.190814458
C12orf41	0.345550389	7.63E-12	6.85E-11
C12orf42	0.082808164	0.111305766	0.157824446
C12orf43	0.140855973	0.006578049	0.012637212
C12orf44	0.179644596	0.000507343	0.00122691
C12orf45	0.132242116	0.010779242	0.019728438
C12orf47	0.341978844	1.29E-11	1.12E-10
C12orf48	0.517757624	7.86E-27	6.75E-25
C12orf49	0.46437383	3.05E-21	1.13E-19
C12orf4	0.362790277	5.54E-13	5.91E-12
C12orf50	-0.00901844	0.862553363	0.891922978
C12orf51	-0.029661588	0.569003357	0.638666115
C12orf52	0.101469696	0.05083237	0.079183867
C12orf53	0.221734242	1.63E-05	5.17E-05
C12orf54	0.026113499	0.616110958	0.681519295
C12orf56	0.164643349	0.001461117	0.003233187
C12orf57	0.123595855	0.017231547	0.030007594
C12orf59	0.231314339	6.74E-06	2.30E-05
C12orf5	0.228378993	8.88E-06	2.96E-05
C12orf60	-0.206253287	6.27E-05	0.000179792
C12orf61	-0.17153906	0.000908108	0.002088344
C12orf62	0.076750697	0.140075107	0.19261294
C12orf63	0.06525003	0.209878411	0.273620686
C12orf65	0.369156653	2.02E-13	2.31E-12
C12orf66	-0.319142068	3.14E-10	2.19E-09
C12orf68	-0.091729525	0.077631826	0.114978904
C12orf69	-0.165368366	0.001391027	0.003090792
C12orf70	0.425347837	9.84E-18	2.18E-16
C12orf71	-0.076010119	0.143951664	0.197100003
C12orf72	-0.526727208	7.17E-28	7.44E-26

C12orf73	0.318124196	3.60E-10	2.48E-09
C12orf74	0.125981302	0.015179662	0.02678321
C12orf75	0.405383128	4.16E-16	7.25E-15
C12orf76	0.296786031	5.57E-09	3.21E-08
C12orf77	0.00811461	0.876211511	0.902392769
C13orf15	-0.218974665	2.09E-05	6.50E-05
C13orf16	-0.046824669	0.368462683	0.444308176
C13orf18	0.320079838	2.77E-10	1.95E-09
C13orf1	-0.345882031	7.27E-12	6.53E-11
C13orf23	0.352799663	2.58E-12	2.49E-11
C13orf26	0.031890546	0.540313978	0.611946281
C13orf27	-0.152731734	0.00318582	0.006557957
C13orf29	0.178638808	0.000546097	0.001313137
C13orf30	0.226926823	1.02E-05	3.35E-05
C13orf31	-0.010666408	0.837758036	0.871536341
C13orf33	0.244623689	1.86E-06	6.96E-06
C13orf34	0.443648313	2.54E-19	7.07E-18
C13orf35	0.199657954	0.000108053	0.000296464
C13orf36	0.024603209	0.636667254	0.70075608
C13orf37	0.333022217	4.65E-11	3.69E-10
C13orf38	0.170134668	0.00100193	0.002286944
C13orf39	0.088697208	0.088002928	0.128335724
C14orf101	0.104052774	0.045190761	0.071332726
C14orf102	-0.147145532	0.004509455	0.008972969
C14orf104	-0.180865652	0.000463729	0.001128837
C14orf105	0.08694749	0.094476268	0.136695201
C14orf106	0.289073722	1.42E-08	7.68E-08
C14orf109	0.218687133	2.14E-05	6.66E-05
C14orf115	0.153538883	0.003026947	0.006259377
C14orf118	0.036601639	0.48214681	0.55631465
C14orf119	0.242847081	2.21E-06	8.20E-06
C14orf126	-0.166071879	0.00132598	0.002955822
C14orf128	-0.117699076	0.023373121	0.039601914
C14orf129	0.234344321	5.06E-06	1.77E-05
C14orf132	0.326500919	1.16E-10	8.61E-10
C14orf135	0.053567969	0.303456575	0.376055106
C14orf138	0.03063696	0.556360275	0.627054163
C14orf139	0.148911049	0.004045472	0.008130941
C14orf142	0.023778784	0.648008386	0.711006967
C14orf143	0.256172216	5.70E-07	2.33E-06
C14orf145	0.175902586	0.000665861	0.001576011
C14orf147	0.093081475	0.073339003	0.10936075
C14orf148	0.040444287	0.437335169	0.513199696
C14orf149	-0.167436331	0.001207724	0.002712851
C14orf153	0.112200493	0.030720981	0.050476156
C14orf156	0.099069317	0.056590173	0.087068206
C14orf159	-0.120865671	0.019873665	0.034128364
C14orf162	-0.087275816	0.09323343	0.135073551
C14orf165	-0.012383114	0.812098373	0.849985488
C14orf166B	0.184522623	0.000353062	0.000878883
C14orf166	0.103195399	0.047002005	0.073910643
C14orf167	-0.354227662	2.08E-12	2.03E-11
C14orf169	0.268150627	1.57E-07	7.09E-07
C14orf174	0.119291247	0.021552112	0.036772969
C14orf176	0.016883964	0.74583674	0.794532888
C14orf178	-0.029579049	0.570079487	0.63965776
C14orf179	-0.104167459	0.044952987	0.070996782
C14orf180	0.021832733	0.67509992	0.73425455

C14orf181	0.020852949	0.68890293	0.746089925
C14orf182	-0.19236917	0.000193264	0.000505542
C14orf183	0.029968156	0.565014822	0.634976324
C14orf184	0.188386994	0.000263191	0.000670841
C14orf19	0.050817179	0.328997174	0.403067566
C14orf1	-0.358976486	1.00E-12	1.03E-11
C14orf21	-0.235934665	4.35E-06	1.54E-05
C14orf23	0.125361023	0.015691465	0.027583637
C14orf28	-0.3150529	5.41E-10	3.63E-09
C14orf2	0.064935974	0.212085305	0.276148401
C14orf33	0.006384639	0.902453073	0.923199468
C14orf34	0.215651979	2.80E-05	8.52E-05
C14orf37	0.132541497	0.010600521	0.0194299
C14orf39	0.150025142	0.003775353	0.007638336
C14orf43	0.144737037	0.005219802	0.0102311
C14orf45	-0.104117404	0.045056634	0.071143558
C14orf48	0.040979793	0.43128561	0.507535704
C14orf49	0.014121805	0.786313493	0.828867475
C14orf4	-0.107716628	0.03809744	0.061178237
C14orf50	0.216195203	2.67E-05	8.15E-05
C14orf53	-0.452326625	4.13E-20	1.27E-18
C14orf64	0.164822073	0.001443544	0.003197142
C14orf68	-0.290121709	1.25E-08	6.84E-08
C14orf70	0.06793864	0.191660109	0.252676823
C14orf72	0.361015796	7.31E-13	7.65E-12
C14orf73	-0.036840394	0.479291994	0.55356572
C14orf79	-0.083047444	0.110272714	0.156515603
C14orf80	0.350045375	3.91E-12	3.67E-11
C14orf86	-0.01181715	0.820537096	0.857331517
C14orf93	0.19565671	0.000149069	0.000398463
C15orf17	0.171979308	0.00088041	0.002032389
C15orf21	0.159436693	0.002067702	0.004439831
C15orf23	0.389084684	7.39E-15	1.06E-13
C15orf24	-0.007759962	0.881581023	0.906563627
C15orf26	-0.038984513	0.45407091	0.529563322
C15orf27	0.276607825	6.10E-08	2.94E-07
C15orf28	-0.056313127	0.279312121	0.349965791
C15orf29	0.224878148	1.23E-05	3.98E-05
C15orf2	-0.074422186	0.152539193	0.207345474
C15orf32	-0.025343889	0.62654987	0.691415638
C15orf33	-0.344933025	8.36E-12	7.47E-11
C15orf34	-0.19385051	0.000172016	0.000454316
C15orf37	-0.164014693	0.0015245	0.003356293
C15orf38	0.122951368	0.017825899	0.030937152
C15orf39	0.526034906	8.65E-28	8.76E-26
C15orf40	-0.003959351	0.939414768	0.953388743
C15orf41	0.050638608	0.330701801	0.404707985
C15orf42	0.570123498	2.30E-33	6.83E-31
C15orf43	-0.429547524	4.33E-18	1.02E-16
C15orf44	-0.038880419	0.455277888	0.530536154
C15orf48	0.350593007	3.60E-12	3.39E-11
C15orf50	-0.012689939	0.807532503	0.846184648
C15orf51	0.129120838	0.012807476	0.023009337
C15orf52	0.193927002	0.000170981	0.000451821
C15orf53	0.167576899	0.001196109	0.002690705
C15orf54	0.137994715	0.00777374	0.014694796
C15orf55	0.171638856	0.000901759	0.002075662
C15orf56	0.324203509	1.58E-10	1.16E-09

C15orf57	0.194726944	0.000160496	0.000426378
C15orf58	-0.149759407	0.003838257	0.007755933
C15orf59	0.214874701	3.00E-05	9.09E-05
C15orf5	0.076455044	0.141613003	0.194405612
C15orf60	0.065236824	0.209970877	0.273718873
C15orf61	-0.078451481	0.131475574	0.182297911
C15orf62	-0.013415874	0.796756098	0.83762401
C15orf63	-0.01754783	0.736207025	0.786124048
C16orf11	0.098850792	0.057140186	0.087794755
C16orf13	0.007681238	0.882773675	0.907555844
C16orf3	0.052887932	0.309645046	0.382439468
C16orf42	-0.051587545	0.321708443	0.395475479
C16orf45	-0.280201033	4.04E-08	2.02E-07
C16orf46	-0.270387715	1.23E-07	5.63E-07
C16orf48	-0.043974095	0.398361423	0.47460271
C16orf52	-0.354792754	1.91E-12	1.88E-11
C16orf53	0.226370485	1.07E-05	3.51E-05
C16orf54	0.132786471	0.010456238	0.019190159
C16orf55	-0.092386818	0.075519939	0.112176276
C16orf57	0.170371722	0.000985491	0.002252777
C16orf58	-0.36407473	4.53E-13	4.90E-12
C16orf59	0.497516608	1.35E-24	8.16E-23
C16orf5	-0.181024393	0.000458322	0.001116494
C16orf61	-0.050613825	0.33093883	0.404948306
C16orf62	-0.210248382	4.47E-05	0.000131862
C16orf63	-0.124453617	0.016467405	0.028807836
C16orf68	0.108824288	0.036148166	0.058349033
C16orf70	-0.467654951	1.48E-21	5.63E-20
C16orf71	-0.235492442	4.53E-06	1.60E-05
C16orf72	-0.215627024	2.81E-05	8.54E-05
C16orf73	0.113244438	0.029190714	0.048252357
C16orf74	0.316262598	4.61E-10	3.13E-09
C16orf75	0.439818655	5.55E-19	1.49E-17
C16orf78	-0.002971639	0.954509524	0.964580437
C16orf79	0.232020745	6.31E-06	2.17E-05
C16orf7	-0.149351199	0.003936734	0.007935607
C16orf80	-0.334626445	3.71E-11	2.99E-10
C16orf81	0.011797655	0.820828156	0.857545677
C16orf82	0.120869184	0.019870051	0.0341251
C16orf86	-0.362288815	5.99E-13	6.36E-12
C16orf87	-0.110026997	0.034128164	0.055465923
C16orf88	0.428719418	5.10E-18	1.18E-16
C16orf89	0.072924551	0.160988997	0.217247361
C16orf90	0.064190149	0.217393078	0.282180407
C16orf91	0.096266393	0.063987267	0.097020652
C16orf92	0.022981699	0.659051335	0.720853228
C16orf93	0.336616382	2.79E-11	2.29E-10
C17orf100	-0.045157418	0.38577728	0.462032883
C17orf101	-0.10135221	0.051102347	0.079560875
C17orf102	0.029143961	0.575768056	0.644878349
C17orf103	-0.228903194	8.45E-06	2.83E-05
C17orf104	0.035999017	0.489393196	0.563510008
C17orf105	0.018689202	0.719747669	0.772815191
C17orf106	-0.140524476	0.006707587	0.012852607
C17orf107	-0.185268529	0.000333751	0.000835089
C17orf108	-0.276966094	5.85E-08	2.83E-07
C17orf28	0.478510296	1.26E-22	5.67E-21
C17orf37	0.135914746	0.008760984	0.016365337

C17orf39	-0.288613565	1.50E-08	8.07E-08
C17orf42	0.21130587	4.08E-05	0.000121175
C17orf44	-0.288445809	1.53E-08	8.20E-08
C17orf46	0.163847416	0.001541785	0.003390975
C17orf47	-0.160643335	0.001909553	0.0041247
C17orf48	-0.213646337	3.34E-05	0.000100403
C17orf49	0.15255695	0.003221205	0.006619169
C17orf50	0.14405937	0.005437053	0.01060891
C17orf51	0.0188496	0.717444757	0.770716336
C17orf53	0.470642616	7.55E-22	3.02E-20
C17orf54	-0.16541095	0.001387008	0.003082205
C17orf55	0.027657174	0.595405802	0.662589341
C17orf56	0.156625647	0.002483574	0.005245734
C17orf57	-0.06984502	0.179463025	0.238605338
C17orf58	-0.077546732	0.135998	0.187693256
C17orf59	0.057039828	0.273144592	0.343296105
C17orf60	0.287462397	1.72E-08	9.15E-08
C17orf61	-0.20198904	8.93E-05	0.000248834
C17orf62	0.146439039	0.0047082	0.00933394
C17orf63	0.317397875	3.97E-10	2.72E-09
C17orf64	0.147258548	0.004478375	0.008916468
C17orf65	0.087512643	0.092345093	0.133971668
C17orf66	0.063062542	0.225597419	0.291215757
C17orf67	0.178490963	0.00055202	0.001326419
C17orf68	0.03564404	0.493688871	0.567659569
C17orf69	0.368307694	2.31E-13	2.64E-12
C17orf70	-0.0265695	0.609961912	0.675843226
C17orf71	0.019818962	0.7035822	0.759026935
C17orf72	0.138228553	0.007669205	0.014519235
C17orf73	0.179159322	0.000525711	0.001268096
C17orf74	-0.058012177	0.26503831	0.334627874
C17orf75	0.185219212	0.000334996	0.000837891
C17orf76	0.388239422	8.54E-15	1.21E-13
C17orf77	0.106758852	0.03985402	0.063721653
C17orf78	-0.010455355	0.840925123	0.87395849
C17orf79	0.145424983	0.005007277	0.009861108
C17orf80	0.185200379	0.000335473	0.000838662
C17orf81	-0.021772729	0.67594218	0.734937312
C17orf82	0.017147982	0.742002186	0.791378958
C17orf85	0.045792263	0.379126795	0.455244652
C17orf86	0.21830081	2.22E-05	6.87E-05
C17orf87	0.132963557	0.010353025	0.01903405
C17orf88	0.129998168	0.012205895	0.022053858
C17orf89	0.006491798	0.900824216	0.9219738
C17orf90	0.064604213	0.214434701	0.27890371
C17orf91	-0.022714126	0.662775152	0.723972287
C17orf93	0.266206131	1.95E-07	8.64E-07
C17orf95	0.026378631	0.612532422	0.678351821
C17orf96	-0.081968905	0.11498936	0.162354219
C17orf97	-0.122431084	0.018318684	0.03172054
C17orf98	0.053868417	0.300748684	0.373185886
C17orf99	0.249108402	1.18E-06	4.59E-06
C18orf10	0.456928882	1.55E-20	5.10E-19
C18orf16	0.076540855	0.141165314	0.193884549
C18orf18	-0.383430063	1.93E-14	2.58E-13
C18orf19	0.022066482	0.671822707	0.731728827
C18orf1	-0.125916328	0.015232578	0.026865852
C18orf20	0.01085386	0.834947283	0.869242254

C18orf21	0.270483508	1.21E-07	5.57E-07
C18orf22	-0.054426763	0.295759126	0.367728473
C18orf25	0.03969127	0.445923685	0.52161891
C18orf26	-0.021834463	0.675075633	0.73425455
C18orf2	0.031532485	0.544873668	0.616164683
C18orf32	-0.123379415	0.01742919	0.030317325
C18orf34	-0.030009295	0.564480615	0.634590757
C18orf45	0.067287228	0.195964031	0.257757521
C18orf54	0.41618715	5.66E-17	1.13E-15
C18orf55	0.129431211	0.012591732	0.022676549
C18orf56	-0.02198775	0.672925855	0.732513923
C18orf62	0.005154537	0.921178748	0.93837107
C18orf8	0.075102648	0.148812882	0.202960926
C19orf10	0.111152634	0.032325463	0.052863887
C19orf12	-0.161307966	0.001827245	0.003961948
C19orf18	0.075127677	0.148677151	0.202803574
C19orf20	-0.142693492	0.005899805	0.011430119
C19orf21	0.528304092	4.67E-28	4.95E-26
C19orf22	0.242078081	2.39E-06	8.81E-06
C19orf23	0.167444606	0.001207037	0.002711615
C19orf24	0.021866846	0.674621253	0.733854133
C19orf25	0.156893652	0.002440867	0.00516429
C19orf26	0.341174339	1.45E-11	1.24E-10
C19orf28	0.172666097	0.000838754	0.001945472
C19orf29	0.027074982	0.603177544	0.669704232
C19orf2	0.000659755	0.989895145	0.992136192
C19orf30	0.064386743	0.215984843	0.280641405
C19orf33	0.262836268	2.81E-07	1.21E-06
C19orf34	0.082271517	0.11365031	0.160713294
C19orf35	0.198460574	0.000119054	0.000324194
C19orf36	-0.305757802	1.81E-09	1.12E-08
C19orf38	-0.032485313	0.532782262	0.604653539
C19orf39	-0.099582798	0.05531503	0.085332664
C19orf40	0.360520322	7.89E-13	8.21E-12
C19orf41	0.01909033	0.713993277	0.767629469
C19orf42	-0.048947109	0.347130314	0.421540032
C19orf43	0.013624084	0.793672258	0.835042975
C19orf44	0.058044548	0.264771304	0.334375526
C19orf45	0.017851421	0.731816896	0.782778856
C19orf46	0.172175893	0.000868295	0.002006982
C19orf47	0.342113085	1.26E-11	1.09E-10
C19orf48	0.355188545	1.80E-12	1.77E-11
C19orf50	0.249327497	1.16E-06	4.50E-06
C19orf51	0.07663183	0.14069187	0.193340928
C19orf52	0.14792736	0.004298368	0.008597599
C19orf53	0.143296034	0.005691477	0.011059852
C19orf54	0.10420225	0.044881062	0.070916919
C19orf55	0.193633384	0.000174987	0.000461551
C19orf56	-0.053624142	0.302949068	0.375542986
C19orf57	0.062963506	0.226328371	0.291896308
C19orf59	0.302272073	2.81E-09	1.70E-08
C19orf60	-0.051390249	0.323565053	0.397537032
C19orf61	0.475158732	2.71E-22	1.16E-20
C19orf62	0.10105821	0.051783183	0.080482787
C19orf63	0.043870579	0.399473865	0.475634613
C19orf66	-0.094019707	0.070475076	0.105603946
C19orf69	0.182384586	0.000414337	0.001017055
C19orf6	-0.083134638	0.10989815	0.156039551

C19orf70	-0.091375379	0.078789411	0.116502831
C19orf71	-0.034240123	0.510873201	0.583645826
C19orf73	-0.240481965	2.80E-06	1.02E-05
C19orf75	-0.028242687	0.587635929	0.655443871
C19orf76	0.163614689	0.001566133	0.003438071
C19orf77	0.32218332	2.08E-10	1.50E-09
C1D	-0.171706818	0.00089746	0.002066959
C1GALT1C1	-0.060175272	0.247601673	0.315572934
C1GALT1	0.043836159	0.399844167	0.476018614
C1QA	0.142403162	0.006002609	0.011613597
C1QBP	0.060985542	0.241280755	0.30866212
C1QB	0.169424055	0.001052736	0.002391949
C1QC	0.194362205	0.0001652	0.00043788
C1QL1	0.187020285	0.000292201	0.000738265
C1QL2	-0.205903478	6.46E-05	0.000184635
C1QL3	0.014236287	0.784623549	0.827445363
C1QL4	0.291565116	1.05E-08	5.81E-08
C1QTNF1	0.174155842	0.00075458	0.001765443
C1QTNF2	0.222515206	1.52E-05	4.85E-05
C1QTNF3	-0.290542002	1.19E-08	6.53E-08
C1QTNF4	0.041410674	0.426453254	0.502769661
C1QTNF6	0.227432071	9.69E-06	3.21E-05
C1QTNF7	0.09741983	0.060852251	0.092759876
C1QTNF8	0.287664473	1.68E-08	8.95E-08
C1QTNF9B	-0.18459473	0.000351151	0.000874344
C1QTNF9	-0.303023163	2.56E-09	1.55E-08
C1RL	-0.353594344	2.29E-12	2.23E-11
C1R	-0.338030948	2.28E-11	1.90E-10
C1S	-0.409308169	2.03E-16	3.72E-15
C1orf100	5.54E-05	0.999151216	0.999301698
C1orf101	-0.315404944	5.17E-10	3.48E-09
C1orf103	-0.096832607	0.062432097	0.094914526
C1orf104	0.139640878	0.007064097	0.013469653
C1orf105	-0.203943282	7.60E-05	0.000214498
C1orf106	0.525117544	1.11E-27	1.10E-25
C1orf107	-0.038979224	0.454132194	0.529572791
C1orf109	-0.018652789	0.720270829	0.773251897
C1orf110	0.103799248	0.045720139	0.072065559
C1orf111	-0.217275143	2.43E-05	7.46E-05
C1orf112	0.083198308	0.109625273	0.155685393
C1orf113	0.427789903	6.11E-18	1.40E-16
C1orf114	0.232627203	5.96E-06	2.06E-05
C1orf115	-0.292860384	9.01E-09	5.03E-08
C1orf116	0.331681977	5.62E-11	4.40E-10
C1orf122	-0.105858962	0.041566321	0.066193784
C1orf123	-0.01720204	0.741217849	0.790711753
C1orf124	0.105959436	0.041372115	0.065942497
C1orf125	0.199385365	0.00011047	0.000302763
C1orf126	0.344715221	8.63E-12	7.67E-11
C1orf127	0.269241972	1.39E-07	6.34E-07
C1orf128	-0.135735686	0.008850977	0.01652105
C1orf129	0.01359007	0.79417583	0.835474352
C1orf130	-0.238327858	3.45E-06	1.24E-05
C1orf131	0.205549612	6.65E-05	0.000189818
C1orf133	-0.040966307	0.431437361	0.507624327
C1orf135	0.45572294	2.00E-20	6.46E-19
C1orf141	-0.126633676	0.01465723	0.025955674
C1orf144	0.147725941	0.004351878	0.008693284

C1orf146	0.019167256	0.712891576	0.766629861
C1orf14	0.042897863	0.410017833	0.486212813
C1orf150	-0.039122243	0.452476682	0.528013148
C1orf151	0.067265487	0.196108883	0.257913993
C1orf152	-0.068751382	0.186387893	0.246512621
C1orf156	0.180980801	0.000459801	0.001119822
C1orf157	0.021003235	0.68677888	0.744275083
C1orf158	0.283438262	2.77E-08	1.42E-07
C1orf159	0.27469577	7.58E-08	3.59E-07
C1orf161	-0.19922705	0.000111897	0.000306168
C1orf162	0.180573615	0.000473831	0.001151318
C1orf163	0.040940158	0.431731695	0.507823039
C1orf168	-0.253511436	7.52E-07	3.02E-06
C1orf170	0.259863305	3.86E-07	1.62E-06
C1orf172	0.221096817	1.73E-05	5.46E-05
C1orf173	-0.305642727	1.84E-09	1.14E-08
C1orf174	0.150404827	0.003687096	0.007477027
C1orf175	0.002024209	0.969004015	0.975565898
C1orf177	0.005065433	0.922536981	0.939418408
C1orf180	0.147436143	0.004429924	0.008828427
C1orf182	-0.0163101	0.754192964	0.801420537
C1orf183	0.200291432	0.000102626	0.000282665
C1orf185	-0.074192929	0.153810436	0.208819102
C1orf186	0.249545103	1.13E-06	4.41E-06
C1orf187	0.255085856	6.38E-07	2.59E-06
C1orf189	0.057269273	0.271216693	0.341131319
C1orf190	0.005332732	0.91846316	0.936370865
C1orf192	-0.114605074	0.027294647	0.045469015
C1orf194	0.103427769	0.046505203	0.073181665
C1orf198	0.24146565	2.54E-06	9.32E-06
C1orf200	0.232277526	6.16E-06	2.12E-05
C1orf201	0.127198502	0.014217726	0.025253704
C1orf203	-0.165988514	0.00133354	0.00297101
C1orf204	0.291440859	1.07E-08	5.88E-08
C1orf210	0.131260336	0.011384191	0.020708168
C1orf212	-0.186110748	0.000313131	0.000787154
C1orf213	0.078525392	0.131111318	0.181865763
C1orf216	0.046005886	0.376904821	0.453150141
C1orf21	-0.154717948	0.002807783	0.005848056
C1orf220	0.054459821	0.295465449	0.367409192
C1orf223	-0.07151591	0.169253632	0.226985585
C1orf226	-0.126907921	0.014442366	0.02561617
C1orf227	-0.068333973	0.189082115	0.249611947
C1orf228	-0.084066672	0.105956626	0.151229969
C1orf229	0.136085604	0.008675872	0.016220038
C1orf230	0.133778039	0.009889851	0.018276958
C1orf25	-0.240690906	2.74E-06	1.00E-05
C1orf26	-0.174424985	0.000740233	0.001735544
C1orf27	0.04309215	0.407898748	0.484074513
C1orf31	0.185783995	0.000320985	0.000805374
C1orf35	0.264073793	2.45E-07	1.07E-06
C1orf38	0.185999616	0.000315782	0.000793218
C1orf43	-0.030773018	0.554607551	0.625559365
C1orf49	-0.018269444	0.725786302	0.777443687
C1orf50	-0.166767059	0.001264461	0.002830403
C1orf51	-0.098982961	0.056806998	0.087363673
C1orf52	0.209960547	4.58E-05	0.000134685
C1orf53	-0.36932868	1.96E-13	2.26E-12

C1orf54	0.275427685	6.97E-08	3.33E-07
C1orf55	-0.097537258	0.060540318	0.092370699
C1orf56	-0.10571938	0.041837395	0.066566935
C1orf57	-0.030512397	0.557967272	0.628466077
C1orf58	-0.021356315	0.681798369	0.740085387
C1orf59	0.207968297	5.43E-05	0.000157224
C1orf61	0.152721559	0.00318787	0.006560822
C1orf63	0.101930767	0.049784329	0.077739725
C1orf64	-0.181465409	0.000443606	0.001083602
C1orf65	0.402322884	7.23E-16	1.22E-14
C1orf66	-0.243510316	2.07E-06	7.71E-06
C1orf68	0.02735817	0.599391561	0.666317654
C1orf69	-0.12014329	0.020629118	0.035331266
C1orf70	0.197185089	0.000131926	0.000355887
C1orf74	-0.07804659	0.133484876	0.1847111
C1orf77	0.431964143	2.69E-18	6.48E-17
C1orf83	0.129146145	0.012789764	0.022979588
C1orf84	0.085666494	0.099452248	0.143032608
C1orf85	0.050927422	0.327947629	0.402028962
C1orf86	0.063275003	0.224034983	0.289669323
C1orf87	-0.109095343	0.035684267	0.057703082
C1orf88	0.286480705	1.94E-08	1.02E-07
C1orf89	-0.249646975	1.12E-06	4.37E-06
C1orf91	0.203650729	7.79E-05	0.000219434
C1orf92	0.050210195	0.334814571	0.408736803
C1orf93	0.43354	1.97E-18	4.86E-17
C1orf94	0.151979536	0.003340651	0.006837816
C1orf95	-0.044879851	0.388707169	0.465038688
C1orf96	0.322163737	2.09E-10	1.50E-09
C1orf97	-0.198911634	0.000114792	0.000313358
C1orf9	0.293966416	7.88E-09	4.43E-08
C20orf103	0.385808249	1.29E-14	1.77E-13
C20orf106	0.092183585	0.076167882	0.113029165
C20orf107	0.178373915	0.000556751	0.001336659
C20orf108	-0.413955406	8.60E-17	1.68E-15
C20orf111	-0.103856241	0.045600682	0.071905714
C20orf112	0.281226208	3.59E-08	1.81E-07
C20orf114	0.235097463	4.71E-06	1.65E-05
C20orf117	0.420523059	2.49E-17	5.24E-16
C20orf118	0.486513775	1.93E-23	9.72E-22
C20orf11	0.07156795	0.168942775	0.226583948
C20orf123	-0.019353661	0.710224423	0.764497703
C20orf12	0.178593667	0.000547899	0.001317312
C20orf132	-0.418577189	3.60E-17	7.42E-16
C20orf134	0.129627669	0.012456834	0.022468541
C20orf135	-0.096468009	0.063429893	0.096292793
C20orf141	0.105655502	0.041961945	0.066727799
C20orf144	0.020690204	0.69120583	0.748055331
C20orf151	0.105476555	0.042312523	0.067220899
C20orf152	0.079699049	0.125431083	0.17506273
C20orf160	-0.229927742	7.68E-06	2.59E-05
C20orf165	-0.074083243	0.154421478	0.209577266
C20orf166	0.05251601	0.313064496	0.385892787
C20orf173	-0.038852773	0.455598747	0.530847949
C20orf177	0.083992133	0.106267681	0.151630478
C20orf186	0.279609383	4.32E-08	2.15E-07
C20orf191	0.01565857	0.763714938	0.809638623
C20orf194	0.218210076	2.23E-05	6.92E-05

C20orf195	0.266931695	1.80E-07	8.04E-07
C20orf196	0.011828821	0.820362864	0.857194429
C20orf197	0.225280672	1.18E-05	3.85E-05
C20orf199	0.263554935	2.60E-07	1.13E-06
C20orf200	0.158553837	0.002190896	0.004675634
C20orf201	0.199899822	0.00010595	0.000291135
C20orf202	0.026855497	0.606119228	0.67222108
C20orf203	0.093668329	0.071536757	0.106993639
C20orf20	0.388820028	7.73E-15	1.10E-13
C20orf24	0.270640027	1.19E-07	5.49E-07
C20orf26	0.194222208	0.000167039	0.000442403
C20orf27	0.095668207	0.065664716	0.099276958
C20orf29	0.038714029	0.457210917	0.532228345
C20orf30	-0.177748649	0.000582669	0.001394848
C20orf3	-0.135927797	0.008754457	0.016354678
C20orf43	0.101966052	0.049704872	0.077639993
C20orf46	0.222277055	1.55E-05	4.95E-05
C20orf4	0.112111325	0.030854812	0.050670973
C20orf54	0.142757747	0.005877268	0.011390887
C20orf56	-0.495425867	2.26E-24	1.31E-22
C20orf70	0.380652655	3.08E-14	3.99E-13
C20orf72	0.353881822	2.19E-12	2.14E-11
C20orf7	0.227380399	9.74E-06	3.22E-05
C20orf85	0.101335754	0.051140255	0.079607452
C20orf94	-0.021917738	0.673907415	0.733317868
C20orf96	0.240569384	2.77E-06	1.01E-05
C21orf119	-0.073205398	0.159378167	0.215379992
C21orf121	-0.084268675	0.105117271	0.150171837
C21orf122	-0.108581542	0.03656795	0.058969297
C21orf125	-0.011400325	0.826765435	0.862345789
C21orf128	0.090060071	0.08321169	0.122270489
C21orf129	0.284419286	2.47E-08	1.28E-07
C21orf130	0.119208349	0.021643796	0.036916755
C21orf131	0.119807485	0.020988705	0.035891586
C21orf15	0.05866385	0.259698743	0.329016799
C21orf29	-0.020901306	0.688219223	0.745552114
C21orf2	-0.062754597	0.227875777	0.293701316
C21orf33	-0.230037305	7.60E-06	2.57E-05
C21orf34	-0.384370364	1.65E-14	2.23E-13
C21orf45	0.467690229	1.46E-21	5.60E-20
C21orf49	-0.108324024	0.037017793	0.059636785
C21orf54	-0.058036041	0.264841457	0.334438286
C21orf56	0.409729613	1.88E-16	3.46E-15
C21orf57	0.023797581	0.647748886	0.710871663
C21orf58	0.374081873	9.10E-14	1.10E-12
C21orf59	0.31719848	4.07E-10	2.79E-09
C21orf62	0.178779263	0.000540525	0.001300838
C21orf63	0.001735337	0.973425639	0.978731481
C21orf67	-0.159068922	0.002118231	0.004536593
C21orf70	0.101304599	0.051212094	0.07969437
C21orf71	0.009883109	0.849525111	0.881287194
C21orf7	-0.187944666	0.000272272	0.000692657
C21orf81	0.173094114	0.000813723	0.00189248
C21orf82	0.087283998	0.093202629	0.135058392
C21orf84	0.098441133	0.058183205	0.089204695
C21orf88	0.151820799	0.003374185	0.00690149
C21orf90	-0.081402572	0.117528554	0.165470237
C21orf91	0.008074505	0.876818431	0.902971144

C21orf94	0.008216849	0.874664614	0.901172344
C21orf96	0.096924637	0.062182295	0.094586216
C21orf99	0.024970366	0.631643447	0.696149633
C22orf13	-0.363575879	4.89E-13	5.27E-12
C22orf15	0.074422726	0.152536206	0.207345474
C22orf23	0.238414971	3.42E-06	1.23E-05
C22orf24	-0.043450282	0.404009688	0.480174263
C22orf25	0.016990887	0.744283038	0.793089788
C22orf26	0.092566175	0.074951844	0.111432137
C22orf27	0.063980582	0.218901471	0.28384243
C22orf28	-0.075814825	0.144987455	0.198313771
C22orf29	0.200477902	0.000101078	0.000278903
C22orf30	0.062472494	0.229977232	0.296122442
C22orf31	0.000805428	0.987664173	0.990697631
C22orf32	-0.190041154	0.000231679	0.000596936
C22orf33	-0.173906456	0.000768103	0.001795184
C22orf34	0.145261715	0.005056997	0.009948207
C22orf36	-0.097407058	0.060886258	0.092784826
C22orf39	0.018338012	0.724798709	0.776722459
C22orf40	0.057683952	0.267755993	0.337502998
C22orf41	0.022589027	0.664519016	0.725519145
C22orf42	-0.019581512	0.706969047	0.761680675
C22orf43	0.13615731	0.008640372	0.016158218
C22orf45	-0.344629394	8.74E-12	7.76E-11
C22orf46	0.01869497	0.719664819	0.772809536
C22orf9	0.324689567	1.48E-10	1.09E-09
C2CD2L	0.014007952	0.787995158	0.830189852
C2CD2	0.225210891	1.19E-05	3.87E-05
C2CD3	0.258629158	4.40E-07	1.84E-06
C2CD4A	0.280806972	3.76E-08	1.89E-07
C2CD4B	0.041984102	0.420071262	0.496126375
C2CD4C	0.101292752	0.051239431	0.079730684
C2CD4D	0.078533511	0.131071355	0.181826024
C2orf14	0.081698088	0.116198171	0.163817138
C2orf15	0.319051613	3.18E-10	2.21E-09
C2orf16	-0.264361062	2.38E-07	1.04E-06
C2orf18	0.345019198	8.25E-12	7.38E-11
C2orf24	-0.061540506	0.237017303	0.303969274
C2orf27A	0.364190338	4.44E-13	4.82E-12
C2orf27B	-0.023850619	0.647016903	0.710185716
C2orf28	0.028858604	0.579513394	0.648380649
C2orf29	0.468902227	1.12E-21	4.34E-20
C2orf34	0.148948901	0.004036015	0.008115209
C2orf39	0.164264071	0.001499061	0.003306499
C2orf3	-0.147730449	0.004350674	0.00869175
C2orf40	-0.100910638	0.052127767	0.080954894
C2orf42	-0.340543793	1.59E-11	1.35E-10
C2orf43	0.223083683	1.44E-05	4.63E-05
C2orf44	0.170475463	0.000978376	0.002237795
C2orf47	-0.334315446	3.88E-11	3.11E-10
C2orf48	0.364377174	4.31E-13	4.69E-12
C2orf49	0.170214488	0.000996367	0.002274767
C2orf50	0.298714129	4.39E-09	2.57E-08
C2orf51	-0.040451607	0.437252154	0.513162734
C2orf52	0.099592275	0.055291721	0.085303312
C2orf53	0.045944835	0.377539018	0.45374833
C2orf54	0.102137142	0.049321106	0.077119149
C2orf55	0.141758665	0.006236588	0.012033444

C2orf56	-0.022728644	0.662572894	0.723870425
C2orf57	0.088727753	0.087893166	0.128214892
C2orf58	-0.243794239	2.02E-06	7.51E-06
C2orf60	0.048977659	0.346829092	0.421287066
C2orf61	0.111732047	0.031429646	0.051525791
C2orf62	-0.097936317	0.059490115	0.0909285
C2orf63	0.031421012	0.546297076	0.617458888
C2orf64	-0.123453578	0.017361245	0.030214967
C2orf65	0.181470033	0.000443454	0.001083456
C2orf66	0.044634125	0.391312183	0.467721257
C2orf67	-0.110353963	0.033595917	0.054687727
C2orf68	0.250217226	1.05E-06	4.14E-06
C2orf69	-0.188556492	0.000259787	0.000662673
C2orf70	0.286880961	1.85E-08	9.77E-08
C2orf71	-0.119603256	0.021210048	0.036232771
C2orf72	-0.175363768	0.000692141	0.001632393
C2orf73	0.100085906	0.054088868	0.083638366
C2orf74	-0.139723832	0.00702992	0.013411181
C2orf76	0.116212373	0.025192097	0.042291814
C2orf77	0.158810317	0.002154434	0.004605712
C2orf78	-0.015977948	0.759042751	0.80561598
C2orf79	0.229555624	7.95E-06	2.68E-05
C2orf7	-0.132846513	0.010421142	0.019143382
C2orf80	-0.008391785	0.872018901	0.899237048
C2orf81	0.357787186	1.21E-12	1.22E-11
C2orf82	0.043892316	0.399240111	0.475498385
C2orf83	0.013826037	0.790684154	0.832698453
C2orf84	-0.000292219	0.995524259	0.996474616
C2orf85	0.067224489	0.196382246	0.258154214
C2orf86	-0.191152037	0.000212535	0.000551822
C2orf88	0.212195112	3.78E-05	0.000112919
C2orf89	0.174449716	0.000738927	0.001732891
C2	-0.195044778	0.000156501	0.000416707
C3AR1	0.203353786	7.98E-05	0.000224275
C3P1	-0.389045028	7.44E-15	1.07E-13
C3orf10	0.064706727	0.213706754	0.27801136
C3orf14	0.259009607	4.22E-07	1.77E-06
C3orf15	-0.072594433	0.162898048	0.219511291
C3orf16	0.113869448	0.028306176	0.046953842
C3orf17	0.165505506	0.001378122	0.003063825
C3orf18	-0.160691343	0.001903496	0.004113847
C3orf19	0.030967113	0.552111849	0.623359153
C3orf1	0.130353701	0.011969297	0.021673314
C3orf20	-0.031212991	0.548958218	0.620079697
C3orf21	0.389181927	7.27E-15	1.05E-13
C3orf22	0.029423382	0.572111694	0.641576649
C3orf23	-0.358072141	1.15E-12	1.17E-11
C3orf24	0.003633171	0.944397501	0.957031742
C3orf26	0.276117183	6.45E-08	3.10E-07
C3orf27	0.120920903	0.019816907	0.034039699
C3orf30	0.007660815	0.883083128	0.907733454
C3orf31	0.098068928	0.059144473	0.09046961
C3orf32	0.244647563	1.85E-06	6.94E-06
C3orf33	0.100898882	0.052155298	0.080991336
C3orf34	0.332486207	5.02E-11	3.96E-10
C3orf35	0.256853923	5.30E-07	2.18E-06
C3orf36	-0.040636921	0.435153417	0.511332215
C3orf37	0.197263645	0.000131097	0.000353891

C3orf38	0.172328671	0.000858987	0.001988003
C3orf39	0.098780382	0.057318344	0.088054908
C3orf42	0.116736756	0.02453705	0.041324466
C3orf43	0.139924695	0.006947779	0.013275815
C3orf45	-0.014978725	0.773688791	0.81781865
C3orf47	-0.01029617	0.843315585	0.875894535
C3orf48	0.039496735	0.448157903	0.523955502
C3orf49	-0.098782135	0.057313902	0.088054875
C3orf50	0.283457495	2.76E-08	1.42E-07
C3orf51	0.084359933	0.104739803	0.14969699
C3orf52	0.472578529	4.88E-22	2.00E-20
C3orf54	0.167042271	0.00124084	0.002781726
C3orf55	0.353229201	2.42E-12	2.35E-11
C3orf57	0.316685125	4.36E-10	2.97E-09
C3orf58	-0.16481247	0.001444484	0.003198867
C3orf59	0.210810366	4.26E-05	0.000126081
C3orf62	0.212384977	3.72E-05	0.000111284
C3orf63	0.244998862	1.79E-06	6.72E-06
C3orf64	0.207528629	5.63E-05	0.000162864
C3orf65	0.094032687	0.070436105	0.105566485
C3orf66	-0.087674214	0.091742934	0.133224194
C3orf67	-0.088448838	0.088899504	0.129519959
C3orf70	-0.002861091	0.956200083	0.965652053
C3orf71	0.071002878	0.172341086	0.230536434
C3orf72	0.171383988	0.000918054	0.002108783
C3orf74	0.035698543	0.493028016	0.566997871
C3orf75	0.04097153	0.431378581	0.507615126
C3orf77	-0.026523098	0.610586382	0.676422282
C3orf79	0.077214004	0.13769092	0.189727383
C3	-0.316087395	4.72E-10	3.20E-09
C4A	-0.286032136	2.04E-08	1.07E-07
C4BPA	-0.467959625	1.38E-21	5.28E-20
C4BPB	-0.340261756	1.65E-11	1.40E-10
C4orf10	0.000254267	0.996105539	0.996918794
C4orf12	-0.102496568	0.048522952	0.07606218
C4orf14	0.116730556	0.024544708	0.041333869
C4orf17	-0.143525684	0.005613831	0.010922819
C4orf19	0.032080996	0.537896492	0.609693554
C4orf21	0.307734833	1.40E-09	8.88E-09
C4orf22	-0.026212206	0.614777628	0.680233264
C4orf23	-0.012493749	0.810451273	0.84861822
C4orf26	0.074419261	0.152555361	0.207345474
C4orf27	0.025114864	0.629670909	0.694282923
C4orf29	-0.229603812	7.92E-06	2.67E-05
C4orf31	-0.097659635	0.060216644	0.091961364
C4orf32	0.006841359	0.895513678	0.917623537
C4orf33	-0.11215959	0.03078231	0.05056441
C4orf34	-0.319043604	3.18E-10	2.21E-09
C4orf35	-0.0801181	0.123449896	0.172702248
C4orf36	-0.152211835	0.003292122	0.006746802
C4orf37	-0.05734338	0.27059601	0.340522627
C4orf38	-0.25022501	1.05E-06	4.13E-06
C4orf39	0.185261506	0.000333928	0.000835427
C4orf3	-0.38237436	2.31E-14	3.05E-13
C4orf40	-0.20499591	6.96E-05	0.000197813
C4orf41	-0.210282834	4.46E-05	0.000131494
C4orf42	0.061016565	0.241041012	0.308414839
C4orf43	0.093864631	0.070942038	0.106213823

C4orf44	0.15601798	0.00258294	0.005439464
C4orf45	-0.084757063	0.103109644	0.147674358
C4orf46	0.43123226	3.11E-18	7.43E-17
C4orf47	0.155868521	0.002607928	0.005483392
C4orf48	0.366029218	3.32E-13	3.69E-12
C4orf49	0.061362154	0.23838165	0.305502685
C4orf50	0.072395897	0.164054327	0.220860272
C4orf51	0.023365165	0.653729308	0.71613303
C4orf52	0.170094777	0.001004721	0.002292527
C4orf6	0.253320417	7.67E-07	3.07E-06
C4orf7	0.203873088	7.65E-05	0.000215658
C5AR1	0.19097801	0.000215433	0.000558472
C5orf13	0.424417291	1.18E-17	2.59E-16
C5orf15	0.182277035	0.000417666	0.001024345
C5orf20	0.224511928	1.27E-05	4.10E-05
C5orf22	0.256847981	5.31E-07	2.18E-06
C5orf23	-0.22039461	1.84E-05	5.78E-05
C5orf24	-0.002595662	0.960259968	0.968818955
C5orf25	0.132873663	0.010405306	0.019116055
C5orf27	-0.284490437	2.45E-08	1.27E-07
C5orf28	0.220335912	1.85E-05	5.81E-05
C5orf30	0.473177594	4.26E-22	1.76E-20
C5orf32	0.232986652	5.76E-06	1.99E-05
C5orf33	-0.485509867	2.45E-23	1.21E-21
C5orf34	0.50724658	1.19E-25	8.57E-24
C5orf35	-0.248624958	1.24E-06	4.80E-06
C5orf36	-0.225116266	1.20E-05	3.90E-05
C5orf38	0.122712897	0.018050311	0.031293908
C5orf39	0.144308863	0.005356141	0.010468462
C5orf40	0.052958476	0.308999251	0.381712847
C5orf41	-0.221163769	1.72E-05	5.43E-05
C5orf42	0.190468419	0.000224133	0.000579065
C5orf43	0.24790497	1.33E-06	5.13E-06
C5orf44	0.098469134	0.058111413	0.089108342
C5orf45	-0.162157942	0.001726718	0.003758295
C5orf46	0.197294547	0.000130772	0.000353062
C5orf47	0.122774982	0.01799165	0.031205786
C5orf48	-0.011309401	0.828125501	0.863448801
C5orf49	0.14415204	0.005406872	0.010554155
C5orf4	-0.316123528	4.70E-10	3.19E-09
C5orf51	0.07310406	0.159957997	0.216017029
C5orf52	0.116216398	0.025187013	0.042286842
C5orf53	-0.260750595	3.51E-07	1.49E-06
C5orf54	0.300327739	3.59E-09	2.13E-08
C5orf55	-0.28962187	1.33E-08	7.23E-08
C5orf56	0.146583276	0.004666995	0.0092587
C5orf58	0.138208487	0.007678126	0.014534742
C5orf60	0.030688289	0.555698723	0.626379425
C5orf62	0.171713878	0.000897014	0.002066171
C5	-0.155645971	0.002645544	0.005552521
C6orf103	0.20118609	9.54E-05	0.000264435
C6orf105	0.192780362	0.000187132	0.000490984
C6orf106	-0.231564159	6.59E-06	2.25E-05
C6orf108	-0.065760568	0.206326239	0.269607197
C6orf10	0.00146543	0.977557711	0.982489392
C6orf114	-0.088867115	0.087393783	0.127618508
C6orf115	0.054794419	0.292503938	0.36422673
C6orf118	0.126675356	0.014624395	0.025902134

C6orf120	-0.134737231	0.009368031	0.017401391
C6orf122	-0.165167599	0.00141012	0.003130772
C6orf123	-0.130548981	0.011841074	0.02146087
C6orf124	-0.001269102	0.980563731	0.984882734
C6orf125	0.297198938	5.30E-09	3.06E-08
C6orf126	0.224155711	1.31E-05	4.23E-05
C6orf127	0.126030104	0.015140023	0.026718004
C6orf129	0.218959939	2.09E-05	6.51E-05
C6orf130	-0.230523564	7.26E-06	2.47E-05
C6orf132	0.440953415	4.41E-19	1.20E-17
C6orf134	0.262327881	2.96E-07	1.28E-06
C6orf136	0.180099893	0.000490653	0.001189425
C6orf138	-0.358235429	1.12E-12	1.15E-11
C6orf141	0.158492985	0.00219963	0.004692763
C6orf142	-0.243067565	2.17E-06	8.03E-06
C6orf145	-0.348803946	4.71E-12	4.36E-11
C6orf146	0.040460767	0.437148269	0.513161736
C6orf147	0.40702392	3.09E-16	5.49E-15
C6orf150	0.321439805	2.31E-10	1.64E-09
C6orf153	0.266905643	1.80E-07	8.06E-07
C6orf154	0.009025566	0.862445825	0.891904367
C6orf155	-0.010968014	0.833236629	0.8676427
C6orf15	0.106987511	0.039428553	0.063092019
C6orf162	0.039154893	0.452099218	0.527728907
C6orf163	0.245975484	1.62E-06	6.13E-06
C6orf164	0.072232988	0.165007706	0.221933805
C6orf165	0.127258599	0.014171652	0.025176356
C6orf167	0.408126627	2.53E-16	4.55E-15
C6orf168	0.374018763	9.19E-14	1.11E-12
C6orf170	0.079521738	0.126276766	0.176106806
C6orf174	0.126900216	0.014448365	0.025617686
C6orf176	0.099448073	0.055647265	0.085805326
C6orf182	0.19488894	0.000158448	0.000421555
C6orf186	0.205237228	6.83E-05	0.000194315
C6orf191	0.116429667	0.024918851	0.041896645
C6orf192	0.38515459	1.44E-14	1.96E-13
C6orf195	0.327433663	1.02E-10	7.64E-10
C6orf1	0.055370117	0.287455083	0.35894949
C6orf201	-0.262914982	2.78E-07	1.20E-06
C6orf203	0.050591617	0.331151314	0.405158539
C6orf204	0.046911638	0.367572915	0.443402217
C6orf208	-0.338743266	2.06E-11	1.72E-10
C6orf211	-0.202356048	8.67E-05	0.000242149
C6orf217	-0.112243053	0.030657279	0.05039227
C6orf218	0.107053378	0.039306709	0.062927374
C6orf221	0.061782244	0.235176856	0.301939377
C6orf222	0.38240365	2.30E-14	3.04E-13
C6orf223	0.291724979	1.03E-08	5.70E-08
C6orf225	-0.106336007	0.040651035	0.064902878
C6orf226	-0.211961327	3.86E-05	0.000115011
C6orf227	0.267127627	1.76E-07	7.88E-07
C6orf25	0.124744735	0.016214885	0.028400997
C6orf26	0.195550033	0.00015034	0.000401538
C6orf27	0.184352296	0.000357615	0.00088944
C6orf35	-0.016573649	0.750351717	0.798147627
C6orf41	-0.039130449	0.452381798	0.527964275
C6orf47	0.081092493	0.118937264	0.167146457
C6orf48	0.189090748	0.000249325	0.000638438

C6orf52	0.043586047	0.402541176	0.478771661
C6orf57	-0.045823173	0.378804791	0.454944977
C6orf58	-0.048438213	0.352172434	0.42689256
C6orf59	0.125423147	0.015639533	0.027502054
C6orf62	0.022060722	0.67190339	0.731754638
C6orf64	0.230384382	7.36E-06	2.49E-05
C6orf70	-0.161866602	0.00176059	0.003826166
C6orf72	-0.148746956	0.004086701	0.008204701
C6orf81	0.015069813	0.772350266	0.816663765
C6orf89	-0.252565671	8.29E-07	3.30E-06
C6orf94	0.140316962	0.006789835	0.012995206
C6orf97	-0.324398015	1.54E-10	1.13E-09
C6	-0.438096203	7.88E-19	2.07E-17
C7orf10	-0.375777121	6.90E-14	8.51E-13
C7orf11	-0.292448116	9.47E-09	5.26E-08
C7orf13	0.13925383	0.007225528	0.013749831
C7orf16	0.128385871	0.013331399	0.023849508
C7orf23	0.306104847	1.73E-09	1.08E-08
C7orf25	0.012634156	0.808362133	0.846920348
C7orf26	0.059214952	0.255241489	0.324066149
C7orf27	0.025864621	0.619478395	0.684446154
C7orf28A	0.301224623	3.21E-09	1.92E-08
C7orf28B	0.157236061	0.002387278	0.005060914
C7orf29	0.1478095	0.004329607	0.008653133
C7orf30	-0.101602581	0.050528444	0.078753533
C7orf31	0.347913833	5.38E-12	4.92E-11
C7orf33	-0.079319383	0.127247291	0.177199814
C7orf34	0.118925674	0.021958976	0.037390318
C7orf36	0.340896641	1.51E-11	1.29E-10
C7orf40	0.192772802	0.000187243	0.000491211
C7orf41	-0.252191408	8.61E-07	3.42E-06
C7orf42	-0.208758159	5.08E-05	0.000147845
C7orf43	0.067884477	0.192015307	0.253031415
C7orf44	0.403995517	5.35E-16	9.20E-15
C7orf45	-0.058961274	0.257286584	0.326329874
C7orf46	0.019657755	0.705880929	0.7607552
C7orf47	0.215008264	2.96E-05	8.99E-05
C7orf49	0.286480444	1.94E-08	1.02E-07
C7orf4	-0.047469564	0.36189663	0.43751872
C7orf50	0.156876506	0.002443579	0.005169479
C7orf51	0.072299369	0.16461873	0.221515322
C7orf52	-0.066334959	0.202382015	0.265201243
C7orf53	0.0365107	0.4832366	0.557442938
C7orf54	0.154618132	0.002825762	0.005881812
C7orf55	-0.35054502	3.63E-12	3.42E-11
C7orf57	0.183058492	0.00039403	0.000971278
C7orf58	-0.480342631	8.21E-23	3.81E-21
C7orf59	0.08557812	0.099803071	0.143475016
C7orf60	0.221040303	1.74E-05	5.49E-05
C7orf61	0.21012808	4.52E-05	0.000133126
C7orf63	-0.183980156	0.000367755	0.000912266
C7orf64	-0.206318904	6.24E-05	0.000178857
C7orf65	0.032216836	0.53617551	0.60801961
C7orf66	0.066526459	0.201079264	0.263702265
C7orf68	0.355763113	1.64E-12	1.63E-11
C7orf69	0.10100797	0.051900282	0.080645614
C7orf70	0.183068506	0.000393736	0.000970792
C7orf71	0.0525143	0.313080269	0.385892787

C7orf72	0.022050888	0.672041137	0.731806698
C7	-0.10272363	0.048024319	0.07538138
C8A	-0.531953163	1.72E-28	1.92E-26
C8B	-0.366924606	2.88E-13	3.23E-12
C8G	-0.173865503	0.000770345	0.001800002
C8ORFK29	0.197523627	0.000128388	0.000347236
C8orf12	0.062058456	0.233086299	0.29964156
C8orf22	0.026973179	0.60454119	0.670844404
C8orf30A	0.184679732	0.00034891	0.000869525
C8orf31	0.217886369	2.30E-05	7.11E-05
C8orf33	0.22059095	1.81E-05	5.69E-05
C8orf34	0.140738242	0.006623796	0.012710389
C8orf37	-0.02633468	0.613124999	0.678808609
C8orf38	0.347330279	5.86E-12	5.34E-11
C8orf39	0.171425899	0.000915356	0.00210307
C8orf40	-0.332462884	5.04E-11	3.97E-10
C8orf41	0.082691757	0.11181108	0.158439458
C8orf42	0.067090175	0.197279764	0.259248513
C8orf44	0.206519798	6.13E-05	0.000176146
C8orf45	-0.070427378	0.17585419	0.234527191
C8orf46	-0.344325348	9.14E-12	8.09E-11
C8orf47	0.350381046	3.72E-12	3.50E-11
C8orf48	0.327979149	9.42E-11	7.13E-10
C8orf4	0.009021779	0.862502978	0.891917173
C8orf51	0.196274025	0.000141907	0.000380803
C8orf55	-0.074589184	0.151618206	0.206222291
C8orf56	-8.04E-05	0.998768712	0.999019445
C8orf58	-0.001001793	0.984656997	0.988278336
C8orf59	0.219719867	1.95E-05	6.11E-05
C8orf71	0.039127707	0.452413506	0.527970351
C8orf73	0.172886857	0.000825756	0.001917332
C8orf74	0.117656089	0.023424076	0.03967138
C8orf75	-0.007699859	0.882491544	0.907406273
C8orf76	0.314318354	5.96E-10	3.98E-09
C8orf77	0.219555569	1.98E-05	6.19E-05
C8orf79	0.1776294	0.000587737	0.001405967
C8orf80	-0.43695195	9.92E-19	2.57E-17
C8orf83	-0.173747554	0.000776836	0.001813468
C8orf84	0.076686638	0.140407232	0.192993709
C8orf85	0.121305443	0.019425636	0.03344836
C8orf86	0.015740361	0.762517598	0.808670513
C9orf100	0.460247134	7.54E-21	2.61E-19
C9orf102	-0.174263598	0.000748805	0.001753785
C9orf103	-0.399314466	1.24E-15	2.00E-14
C9orf106	0.132261547	0.010767561	0.019712494
C9orf109	0.423370617	1.44E-17	3.15E-16
C9orf110	0.410445304	1.65E-16	3.08E-15
C9orf114	-0.011873353	0.819698157	0.856663959
C9orf116	-0.045845968	0.378567436	0.454737442
C9orf117	0.190938342	0.000216099	0.000559939
C9orf119	-0.069785131	0.179837239	0.239023115
C9orf11	0.174402578	0.000741418	0.001738117
C9orf122	0.290495554	1.20E-08	6.56E-08
C9orf123	-0.132534636	0.010604587	0.019435565
C9orf125	0.272831889	9.35E-08	4.36E-07
C9orf128	-0.088377849	0.089157107	0.129807676
C9orf129	-0.122150783	0.01858906	0.032144019
C9orf130	-0.200554499	0.000100449	0.000277358

C9orf131	-0.121878385	0.018855139	0.032553261
C9orf135	0.111542195	0.031720807	0.051968908
C9orf139	0.201530868	9.27E-05	0.000257611
C9orf140	0.563949349	1.56E-32	3.93E-30
C9orf142	0.249551772	1.13E-06	4.40E-06
C9orf144B	-0.052377015	0.314348745	0.387216701
C9orf144	-0.030413974	0.559238627	0.629692643
C9orf150	-0.007540592	0.884905103	0.909090319
C9orf152	-0.1585127	0.002196797	0.004687221
C9orf153	0.002808476	0.957004773	0.966125579
C9orf156	-0.073280958	0.158946863	0.214884596
C9orf163	0.141440017	0.006355264	0.012245823
C9orf167	0.380871743	2.97E-14	3.86E-13
C9orf169	-0.213975673	3.24E-05	9.77E-05
C9orf16	0.030277277	0.561006689	0.631397958
C9orf170	0.137288818	0.008097059	0.015243073
C9orf171	0.079921144	0.124378008	0.17384822
C9orf172	0.319160586	3.13E-10	2.18E-09
C9orf173	-0.298111075	4.73E-09	2.75E-08
C9orf21	-0.057765022	0.267082965	0.336825146
C9orf23	-0.204251958	7.41E-05	0.000209667
C9orf24	-0.155048673	0.002748955	0.005738728
C9orf25	0.270268736	1.24E-07	5.70E-07
C9orf30	0.375318282	7.44E-14	9.14E-13
C9orf37	0.021761216	0.676103822	0.735065507
C9orf3	-0.159914541	0.002003694	0.004312152
C9orf40	0.228949606	8.42E-06	2.82E-05
C9orf41	-0.095845871	0.06516278	0.098600402
C9orf43	-0.205509792	6.67E-05	0.000190378
C9orf44	0.018566198	0.721515425	0.774170857
C9orf45	0.290520247	1.20E-08	6.55E-08
C9orf46	-0.013587892	0.794208076	0.835474352
C9orf47	0.089031802	0.086806593	0.126869705
C9orf4	0.064385165	0.215996118	0.280641405
C9orf50	-0.026332876	0.613149328	0.678808609
C9orf53	0.096037689	0.064624397	0.097889845
C9orf57	0.175590611	0.000680962	0.001609077
C9orf5	-0.312636559	7.44E-10	4.90E-09
C9orf64	-0.082287806	0.113578578	0.160658367
C9orf66	0.260722527	3.52E-07	1.50E-06
C9orf68	0.024878336	0.632901118	0.697150064
C9orf69	0.12935237	0.01264623	0.022755596
C9orf6	0.095465522	0.066241216	0.100027098
C9orf70	0.123118384	0.017670183	0.030682941
C9orf71	-0.273973311	8.22E-08	3.87E-07
C9orf72	-0.228243973	8.99E-06	3.00E-05
C9orf78	-0.226100852	1.10E-05	3.59E-05
C9orf79	0.21473486	3.03E-05	9.19E-05
C9orf7	0.034567976	0.506832535	0.579890627
C9orf80	0.114867034	0.026942016	0.044926663
C9orf82	-0.281451989	3.49E-08	1.77E-07
C9orf84	-0.060448591	0.245456735	0.313239963
C9orf85	0.064873575	0.212525772	0.276673752
C9orf86	0.425940525	8.77E-18	1.96E-16
C9orf89	0.293391309	8.45E-09	4.74E-08
C9orf91	-0.133734912	0.009913907	0.018318016
C9orf93	-0.032115345	0.537461054	0.609303996
C9orf95	-0.371574431	1.37E-13	1.61E-12

C9orf96	-0.184146534	0.000363189	0.000902063
C9orf98	0.172638645	0.000840383	0.001948344
C9orf9	-0.116262759	0.025128507	0.042199286
C9	-0.056223008	0.280083488	0.350789585
CA10	0.080462363	0.121840564	0.170744775
CA11	0.236262707	4.21E-06	1.49E-05
CA12	0.268936356	1.44E-07	6.53E-07
CA13	-0.054532051	0.294824456	0.366749474
CA14	-0.330710647	6.44E-11	5.02E-10
CA1	0.025190229	0.628643143	0.693375259
CA2	-0.134698048	0.009388859	0.017436827
CA3	-0.170310045	0.000989744	0.002261461
CA4	-0.422943009	1.57E-17	3.40E-16
CA5A	-0.398848083	1.34E-15	2.15E-14
CA5BP	0.367745525	2.53E-13	2.86E-12
CA5B	-0.030815419	0.554061888	0.625135693
CA6	-0.004018061	0.938518168	0.952575858
CA7	0.203520546	7.87E-05	0.000221491
CA8	-0.017461303	0.737459845	0.787166472
CA9	0.403059264	6.34E-16	1.08E-14
CAB39L	0.068350955	0.188971951	0.249499583
CAB39	0.045242137	0.384885702	0.461075944
CABC1	-0.323514229	1.74E-10	1.27E-09
CABIN1	0.268302733	1.55E-07	6.98E-07
CABLES1	-0.152170443	0.003300722	0.006762339
CABLES2	0.234584606	4.95E-06	1.73E-05
CABP1	-0.003824479	0.941474809	0.95489569
CABP2	-0.032821713	0.528545862	0.600495618
CABP4	0.145484908	0.004989138	0.009833163
CABP5	0.024115491	0.643366475	0.706801969
CABP7	0.145811898	0.004891202	0.009661166
CABYR	0.233816296	5.32E-06	1.85E-05
CACHD1	0.218705437	2.14E-05	6.65E-05
CACNA1A	0.161567168	0.001796036	0.00389937
CACNA1B	0.149107605	0.003996582	0.008043227
CACNA1C	0.112016255	0.030998049	0.050868462
CACNA1D	0.324780568	1.46E-10	1.07E-09
CACNA1E	0.212164798	3.79E-05	0.000113164
CACNA1F	0.300632852	3.46E-09	2.05E-08
CACNA1G	0.329113754	8.05E-11	6.17E-10
CACNA1H	0.189069822	0.000249727	0.000639303
CACNA1I	-0.011512016	0.825095428	0.86110941
CACNA1S	0.027455597	0.598091526	0.665095138
CACNA2D1	0.076095403	0.143501114	0.196604717
CACNA2D2	0.187689514	0.000277642	0.000705328
CACNA2D3	0.046141031	0.375503267	0.451574073
CACNA2D4	0.144838547	0.005187941	0.01018067
CACNB1	0.47481849	2.93E-22	1.25E-20
CACNB2	0.031328188	0.547483755	0.618694842
CACNB3	0.362249741	6.03E-13	6.39E-12
CACNB4	-0.004569771	0.930096494	0.94566614
CACNG1	0.07228313	0.164713827	0.221598383
CACNG2	-0.048306861	0.353481326	0.428348843
CACNG3	0.076867093	0.139473174	0.1918912
CACNG4	0.171872529	0.000887055	0.002046307
CACNG5	0.057691739	0.267691296	0.337464152
CACNG6	0.102059084	0.049495889	0.077349945
CACNG7	0.137796867	0.007863177	0.014848361

CACNG8	0.08258436	0.112278874	0.159000549
CACYBP	0.44296579	2.92E-19	8.10E-18
CADM1	-0.418382661	3.74E-17	7.67E-16
CADM2	0.141063512	0.006498094	0.012493247
CADM3	0.178120954	0.000567105	0.001359878
CADM4	0.064020061	0.21861674	0.283583975
CADPS2	-0.000348736	0.994658633	0.996008509
CADPS	0.24809395	1.31E-06	5.04E-06
CAD	0.247683485	1.36E-06	5.24E-06
CAGE1	0.213901512	3.26E-05	9.83E-05
CALB1	0.136947853	0.008257481	0.015514999
CALB2	0.258670964	4.38E-07	1.83E-06
CALCA	0.217442609	2.39E-05	7.36E-05
CALCB	0.125908793	0.015238725	0.026870762
CALCOCO1	-0.297188011	5.30E-09	3.06E-08
CALCOCO2	-0.259737126	3.91E-07	1.64E-06
CALCRL	-0.082823717	0.111238388	0.157740136
CALCR	0.260217764	3.71E-07	1.57E-06
CALD1	-0.339895298	1.74E-11	1.47E-10
CALHM1	0.224659298	1.25E-05	4.06E-05
CALHM2	0.166009458	0.001331637	0.002967435
CALHM3	0.321294392	2.35E-10	1.67E-09
CALM1	0.053887813	0.300574431	0.372993884
CALM2	0.296134601	6.04E-09	3.46E-08
CALM3	0.099419698	0.055717451	0.085900252
CALML3	-0.137888042	0.007821848	0.014780124
CALML4	-0.239270202	3.15E-06	1.14E-05
CALML5	0.09906818	0.056593023	0.087068206
CALML6	-0.035143959	0.499774446	0.573465414
CALN1	0.090448344	0.081885899	0.120500139
CALR3	-0.132808485	0.010443358	0.019173159
CALR	0.131797266	0.011049729	0.020166059
CALU	0.251022414	9.71E-07	3.84E-06
CALY	0.140194432	0.006838822	0.013081423
CAMK1D	-0.170469205	0.000978803	0.002238517
CAMK1G	0.192432036	0.000192314	0.00050319
CAMK1	0.066870586	0.198753546	0.260995856
CAMK2A	0.023418688	0.652987857	0.71547817
CAMK2B	-0.021949267	0.673465307	0.732916849
CAMK2D	-0.015175533	0.77079756	0.81541148
CAMK2G	-0.073749421	0.156292455	0.211763072
CAMK2N1	-0.207011401	5.88E-05	0.000169529
CAMK2N2	0.028650154	0.582256487	0.650828353
CAMK4	0.171631569	0.000902222	0.002076486
CAMKK1	0.182156375	0.000421431	0.001032561
CAMKK2	0.089895372	0.083779275	0.123013761
CAMKV	0.221848944	1.61E-05	5.12E-05
CAMLG	0.092634879	0.074735154	0.111134862
CAMP	0.175353914	0.00069263	0.001633161
CAMSAP1L	0.206715527	6.03E-05	0.000173499
CAMSAP1	0.122752325	0.018013038	0.031237444
CAMTA1	0.069241473	0.183260722	0.242844227
CAMTA2	-0.08900251	0.0869108	0.126994056
CAND1	0.226230453	1.08E-05	3.56E-05
CAND2	0.206821875	5.98E-05	0.000172079
CANT1	0.276893747	5.90E-08	2.85E-07
CANX	0.283605152	2.72E-08	1.40E-07
CAP1	0.283236072	2.84E-08	1.46E-07

CAP2	-0.170519162	0.000975392	0.002231228
CAPG	0.50598062	1.64E-25	1.16E-23
CAPN10	0.427105759	6.99E-18	1.58E-16
CAPN11	-0.064484219	0.215289039	0.279868726
CAPN12	0.181745215	0.000434499	0.001062488
CAPN13	0.292805158	9.07E-09	5.06E-08
CAPN14	0.119182735	0.021672193	0.036962026
CAPN1	0.216810223	2.53E-05	7.76E-05
CAPN2	0.175089184	0.000705899	0.001662087
CAPN3	-0.145142542	0.005093569	0.01001424
CAPN5	-0.005125289	0.921624553	0.938737753
CAPN6	0.269790015	1.31E-07	5.99E-07
CAPN7	-0.146154666	0.004790402	0.009480865
CAPN8	0.181287337	0.000449494	0.001097002
CAPN9	0.308895565	1.21E-09	7.70E-09
CAPNS1	0.220250307	1.86E-05	5.85E-05
CAPNS2	-0.037053861	0.47674735	0.551265929
CAPRIN1	0.32064291	2.57E-10	1.81E-09
CAPRIN2	0.07003518	0.178278634	0.23734743
CAPS2	-0.120056184	0.020721875	0.03548098
CAPSL	0.215676083	2.79E-05	8.51E-05
CAPS	0.093554156	0.071884527	0.107457308
CAPZA1	0.327208951	1.05E-10	7.87E-10
CAPZA2	0.039790439	0.444787177	0.520472842
CAPZA3	-0.014875482	0.775206755	0.81907468
CAPZB	0.106450692	0.040433549	0.06457569
CARD10	0.239250185	3.15E-06	1.14E-05
CARD11	0.358625732	1.06E-12	1.08E-11
CARD14	0.338925056	2.01E-11	1.68E-10
CARD16	0.191227225	0.000211294	0.000549101
CARD17	0.108968161	0.0359013	0.058002247
CARD18	0.104656876	0.043950064	0.069550653
CARD6	0.184509829	0.000353402	0.00087951
CARD8	0.073209993	0.159351911	0.215359119
CARD9	0.411517143	1.35E-16	2.56E-15
CARHSP1	0.098054722	0.05918142	0.090512226
CARKD	-0.227086783	1.00E-05	3.30E-05
CARM1	0.222522183	1.52E-05	4.85E-05
CARNS1	0.141991539	0.006151118	0.011877733
CARS2	0.124100041	0.016778722	0.029298423
CARS	0.120981297	0.019755006	0.03394215
CARTPT	-0.052818066	0.310285513	0.383110289
CASC1	-0.027687131	0.595007125	0.662256659
CASC2	-0.102612518	0.048267783	0.075727735
CASC3	0.088393525	0.089100169	0.129755377
CASC4	-0.142645318	0.005916753	0.011460725
CASC5	0.094962969	0.067688562	0.102027051
CASD1	-0.063837946	0.219932387	0.284993691
CASKIN1	0.149241109	0.00396368	0.007984271
CASKIN2	0.101705293	0.050294568	0.078456533
CASK	-0.029680742	0.568753767	0.638421937
CASP10	0.114877125	0.02692851	0.044907901
CASP12	-0.186211462	0.000310746	0.000781949
CASP14	0.017814946	0.732343885	0.783132307
CASP1	0.182111811	0.00042283	0.001035606
CASP2	0.460808036	6.68E-21	2.33E-19
CASP3	0.308621396	1.25E-09	7.97E-09
CASP4	0.334885334	3.57E-11	2.88E-10

CASP5	0.249497257	1.14E-06	4.43E-06
CASP6	0.277806124	5.32E-08	2.59E-07
CASP7	0.272039174	1.02E-07	4.73E-07
CASP8AP2	0.228823477	8.52E-06	2.85E-05
CASP8	0.256984984	5.23E-07	2.16E-06
CASP9	-0.131946967	0.010958041	0.020020736
CASQ1	0.111392958	0.031951294	0.052320703
CASQ2	-0.280097057	4.09E-08	2.04E-07
CASR	0.090926888	0.080275406	0.118462714
CASS4	-0.246260911	1.58E-06	5.98E-06
CAST	0.000601475	0.990787717	0.992832791
CASZ1	0.276581387	6.12E-08	2.95E-07
CATSPER1	0.310706611	9.57E-10	6.21E-09
CATSPER2I	-0.140967421	0.006535004	0.012560574
CATSPER2	0.100362389	0.053424705	0.082752527
CATSPER3	0.047453109	0.362063252	0.437577293
CATSPER4	0.075569746	0.146295327	0.19993795
CATSPERB	0.280839221	3.75E-08	1.88E-07
CATSPERG	-0.14136432	0.006383752	0.012294771
CAT	-0.491532401	5.80E-24	3.16E-22
CAV1	0.030625961	0.55650208	0.62707377
CAV2	0.097868934	0.059666384	0.091176935
CAV3	0.063955234	0.219084419	0.284024194
CBARA1	-0.387844156	9.14E-15	1.28E-13
CBFA2T2	0.40540287	4.15E-16	7.24E-15
CBFA2T3	-0.085430184	0.100392538	0.14427037
CBFB	0.290310329	1.23E-08	6.70E-08
CBLB	0.063398376	0.223131236	0.288669426
CBLC	0.067342934	0.195593241	0.257303787
CBLL1	0.016154297	0.756466671	0.803279624
CBLN1	-0.313523884	6.62E-10	4.38E-09
CBLN2	0.127191172	0.014223355	0.02526145
CBLN3	-0.150501628	0.003664895	0.007440671
CBLN4	-0.238605751	3.36E-06	1.21E-05
CBL	0.303533688	2.40E-09	1.46E-08
CBR1	-0.341294922	1.42E-11	1.22E-10
CBR3	0.301607869	3.06E-09	1.83E-08
CBR4	-0.437110925	9.61E-19	2.49E-17
CBS	-0.195198748	0.000154599	0.000412139
CBWD1	0.10565193	0.04196892	0.066733564
CBWD2	0.072570335	0.163038067	0.219670253
CBWD3	-0.014117575	0.786375953	0.828867475
CBWD5	0.057404902	0.270081462	0.340003974
CBWD6	0.091826349	0.077317753	0.114581884
CBX1	0.433589845	1.95E-18	4.82E-17
CBX2	0.281146561	3.62E-08	1.82E-07
CBX3	0.412237648	1.18E-16	2.26E-15
CBX4	0.126316433	0.014909279	0.02633413
CBX5	0.277249266	5.67E-08	2.75E-07
CBX6	0.150387302	0.003691128	0.007484442
CBX7	-0.388020169	8.87E-15	1.25E-13
CBX8	0.024150431	0.642885584	0.706429488
CBY1	0.092249104	0.075958503	0.11275919
CC2D1A	-0.110207148	0.033834026	0.055037272
CC2D1B	0.201362479	9.40E-05	0.000260936
CC2D2A	0.19736547	0.00013003	0.000351247
CC2D2B	0.094248253	0.069791464	0.104745031
CCAR1	0.331211347	6.00E-11	4.69E-10

CCBE1	0.06760846	0.193832898	0.255222411
CCBL1	-0.129985378	0.012214482	0.022067372
CCBL2	-0.186849003	0.00029604	0.000746923
CCBP2	-0.478712953	1.20E-22	5.45E-21
CCDC101	-0.064468792	0.215399049	0.279975197
CCDC102A	0.198039488	0.000123167	0.000334388
CCDC102B	0.225949501	1.11E-05	3.64E-05
CCDC103	0.065634614	0.207198527	0.27056955
CCDC104	0.132904785	0.01038718	0.019088036
CCDC106	-0.278124294	5.13E-08	2.51E-07
CCDC107	-0.00540341	0.917386334	0.935416652
CCDC108	0.198881362	0.000115074	0.000314083
CCDC109A	0.442579311	3.16E-19	8.68E-18
CCDC109B	0.490836078	6.86E-24	3.67E-22
CCDC110	0.011507193	0.825167529	0.861130828
CCDC111	-0.14320624	0.005722098	0.011111769
CCDC112	0.449491166	7.52E-20	2.25E-18
CCDC113	-0.147625262	0.004378851	0.008738401
CCDC114	0.118106674	0.022894707	0.038850796
CCDC115	-0.065709582	0.206679022	0.269997343
CCDC116	-0.095059073	0.067409798	0.101629937
CCDC117	-0.15382438	0.002972498	0.006157649
CCDC11	0.010183641	0.845006285	0.877421829
CCDC120	0.341978569	1.29E-11	1.12E-10
CCDC121	-0.219802077	1.94E-05	6.07E-05
CCDC122	-0.003724823	0.942997191	0.956019927
CCDC123	0.285818507	2.09E-08	1.10E-07
CCDC124	0.185680703	0.000323506	0.000810882
CCDC125	-0.023141825	0.656826874	0.718933299
CCDC126	-0.257811	4.79E-07	1.99E-06
CCDC127	0.265781064	2.04E-07	9.02E-07
CCDC129	0.089715933	0.084401205	0.123753647
CCDC12	0.127923336	0.013670709	0.024401744
CCDC130	0.155648203	0.002645164	0.005552308
CCDC132	-0.107909697	0.037751405	0.060671465
CCDC134	0.166888955	0.001253948	0.002808133
CCDC135	-0.105748112	0.041781476	0.066493881
CCDC136	0.394951188	2.67E-15	4.11E-14
CCDC137	0.357778705	1.21E-12	1.22E-11
CCDC138	0.228572906	8.72E-06	2.91E-05
CCDC13	0.003353647	0.948669242	0.960334788
CCDC140	0.142346826	0.006022743	0.011647906
CCDC141	0.123511864	0.01730801	0.030135481
CCDC142	0.01701662	0.74390928	0.792818738
CCDC144A	-0.066234412	0.203068472	0.265960824
CCDC144B	-0.038870064	0.455398051	0.53064514
CCDC144C	0.157598544	0.002331718	0.004950707
CCDC144NI	0.129702407	0.01240585	0.022384689
CCDC146	-0.223910742	1.34E-05	4.32E-05
CCDC147	-0.201327753	9.43E-05	0.000261605
CCDC148	0.044010758	0.397967867	0.474266665
CCDC149	0.348794388	4.71E-12	4.36E-11
CCDC14	0.246951924	1.47E-06	5.61E-06
CCDC150	0.178448546	0.00055373	0.001329887
CCDC151	0.147508067	0.004410437	0.008795269
CCDC152	-0.326799941	1.11E-10	8.30E-10
CCDC153	0.099250606	0.056137217	0.086460428
CCDC154	0.23959616	3.05E-06	1.11E-05

CCDC155	0.038656062	0.457885434	0.532857855
CCDC157	0.027157927	0.602067535	0.668732685
CCDC158	-0.354942991	1.86E-12	1.84E-11
CCDC159	-0.154083582	0.002923837	0.00606378
CCDC15	-0.094925851	0.067796482	0.102174258
CCDC160	0.23156149	6.59E-06	2.25E-05
CCDC163P	0.205154798	6.87E-05	0.000195544
CCDC17	-0.033410618	0.521170945	0.593429867
CCDC18	0.245487862	1.70E-06	6.42E-06
CCDC19	0.292969244	8.89E-09	4.97E-08
CCDC21	0.185710217	0.000322784	0.000809582
CCDC22	0.112259575	0.03063258	0.050362194
CCDC23	-0.009094278	0.861409146	0.891109824
CCDC24	0.138461545	0.007566299	0.014335312
CCDC25	-0.203198477	8.08E-05	0.000226949
CCDC27	0.121016054	0.019719458	0.033892765
CCDC28A	-0.262810275	2.81E-07	1.22E-06
CCDC28B	0.363655516	4.83E-13	5.21E-12
CCDC30	0.081879635	0.11538673	0.162834486
CCDC33	0.148178604	0.004232453	0.00847171
CCDC34	0.192650967	0.000189042	0.000495473
CCDC36	0.145523806	0.004977396	0.009817237
CCDC37	0.06781119	0.192496685	0.253632206
CCDC38	-0.119000951	0.021874657	0.03727223
CCDC39	0.018290981	0.725476061	0.777248405
CCDC3	0.00613331	0.906274974	0.92645862
CCDC40	0.076106556	0.143442272	0.196564654
CCDC41	0.22407528	1.32E-05	4.26E-05
CCDC42B	0.243808085	2.01E-06	7.51E-06
CCDC42	-0.089206812	0.086186077	0.126073796
CCDC43	0.448369577	9.51E-20	2.81E-18
CCDC45	0.283197666	2.85E-08	1.46E-07
CCDC46	0.23132753	6.74E-06	2.30E-05
CCDC47	-0.052419488	0.313955949	0.386828525
CCDC48	-0.044901741	0.388475615	0.46481749
CCDC50	0.036794461	0.479840508	0.554134984
CCDC51	0.055771188	0.283972507	0.354956725
CCDC52	0.243807749	2.01E-06	7.51E-06
CCDC53	-0.283235912	2.84E-08	1.46E-07
CCDC54	-0.0634856	0.222493866	0.28793834
CCDC55	0.07298863	0.160620387	0.216808683
CCDC56	0.073086381	0.160059316	0.216139205
CCDC57	-0.070896785	0.172984762	0.23124792
CCDC58	0.208903481	5.01E-05	0.000146275
CCDC59	0.338982433	1.99E-11	1.67E-10
CCDC60	-0.030017074	0.564379628	0.634513034
CCDC61	0.030802736	0.554225075	0.625240877
CCDC62	0.092224956	0.076035617	0.112849703
CCDC63	0.144485592	0.005299484	0.010368917
CCDC64B	0.40543011	4.13E-16	7.21E-15
CCDC64	0.393718548	3.32E-15	5.03E-14
CCDC65	0.235548656	4.51E-06	1.59E-05
CCDC66	0.295966578	6.17E-09	3.52E-08
CCDC67	0.151458995	0.003451761	0.007045695
CCDC68	0.054758112	0.292824326	0.364557319
CCDC69	-0.000304086	0.995342493	0.996442872
CCDC6	0.370477368	1.63E-13	1.90E-12
CCDC70	-0.071884308	0.167062208	0.224378973

CCDC71	-0.009030004	0.862378871	0.891881424
CCDC72	0.160255266	0.001959165	0.004222251
CCDC73	0.006082551	0.907047131	0.926937967
CCDC74A	0.423824747	1.32E-17	2.89E-16
CCDC74B	0.444558129	2.10E-19	5.91E-18
CCDC75	-0.012314913	0.813114155	0.85086985
CCDC76	0.09728721	0.061206135	0.093233761
CCDC77	0.129651631	0.012440468	0.022441054
CCDC78	0.217456456	2.39E-05	7.35E-05
CCDC79	0.070972472	0.172525382	0.230767467
CCDC7	-0.049948776	0.337340246	0.411416736
CCDC80	0.21168834	3.95E-05	0.000117518
CCDC81	0.220481249	1.83E-05	5.74E-05
CCDC82	0.061053104	0.240758863	0.308113193
CCDC83	0.123486062	0.017331558	0.030168572
CCDC84	0.180551889	0.00047459	0.001153023
CCDC85A	-0.162765076	0.001658041	0.003619889
CCDC85B	-0.032384381	0.534056651	0.605961761
CCDC85C	0.037530588	0.471091206	0.545905995
CCDC86	0.192215752	0.0001956	0.000511249
CCDC87	0.018521663	0.722155828	0.774636697
CCDC88A	0.160934457	0.001873092	0.004053855
CCDC88B	0.340208667	1.67E-11	1.41E-10
CCDC88C	0.337702637	2.39E-11	1.98E-10
CCDC89	0.162813282	0.001652696	0.003609012
CCDC8	0.252746162	8.13E-07	3.24E-06
CCDC90A	-0.019815908	0.70362572	0.759026935
CCDC90B	-0.140699087	0.006639073	0.012734798
CCDC91	0.1152907	0.026379998	0.044100219
CCDC92	0.059946156	0.249409753	0.317511732
CCDC93	0.277816784	5.31E-08	2.59E-07
CCDC94	0.032971289	0.526667708	0.598677956
CCDC96	0.302930604	2.59E-09	1.57E-08
CCDC97	0.307984527	1.36E-09	8.61E-09
CCDC99	0.570454928	2.07E-33	6.35E-31
CCDC9	0.226467891	1.06E-05	3.49E-05
CCHCR1	0.123502091	0.017316926	0.03014837
CCIN	0.25104111	9.70E-07	3.83E-06
CCKAR	0.070555464	0.175067735	0.233603443
CCKBR	0.303249618	2.49E-09	1.51E-08
CCK	0.128601517	0.013175753	0.023594369
CCL11	0.188322062	0.000264506	0.000673848
CCL13	0.156775293	0.002459645	0.005199601
CCL14-CCL	-0.246471938	1.54E-06	5.86E-06
CCL14	-0.384065003	1.74E-14	2.33E-13
CCL15	-0.111264133	0.032151403	0.052605145
CCL16	-0.407413137	2.88E-16	5.14E-15
CCL17	0.139280111	0.007214463	0.013730086
CCL18	0.052703293	0.31133953	0.384175046
CCL19	0.191238205	0.000211114	0.000548703
CCL1	0.238688676	3.33E-06	1.20E-05
CCL20	0.413297583	9.72E-17	1.88E-15
CCL21	0.109984599	0.034197703	0.055554011
CCL22	0.225355433	1.17E-05	3.82E-05
CCL23	-0.135440034	0.00900137	0.016784471
CCL24	0.028495563	0.584294732	0.652557442
CCL25	0.102657479	0.048169142	0.075590835
CCL26	0.384403387	1.64E-14	2.22E-13

CCL27	0.094589946	0.068779535	0.103436695
CCL28	0.186163888	0.000311871	0.000784381
CCL2	0.134545535	0.009470318	0.017570094
CCL3L1	0.224590195	1.26E-05	4.08E-05
CCL3L3	0.073351285	0.158546222	0.214444826
CCL3	0.094213328	0.069895577	0.104877584
CCL4L2	0.209821015	4.64E-05	0.000136098
CCL4	0.122818249	0.017950868	0.031137762
CCL5	0.132595528	0.010568548	0.019380211
CCL7	0.193471451	0.000177234	0.000466922
CCL8	0.010537629	0.8396902	0.873130548
CCM2	-0.186081053	0.000313837	0.000788759
CCNA1	0.145869284	0.004874194	0.009632347
CCNA2	0.502526485	3.90E-25	2.64E-23
CCNB1IP1	-0.168815785	0.001098099	0.002488209
CCNB1	0.659517385	1.14E-47	7.55E-44
CCNB2	0.571858012	1.33E-33	4.23E-31
CCNB3	-0.076043229	0.143776618	0.196887392
CCNC	0.041269396	0.428034216	0.50439475
CCND1	-0.240698675	2.74E-06	1.00E-05
CCND2	0.214011183	3.23E-05	9.74E-05
CCND3	0.232538602	6.01E-06	2.07E-05
CCNDBP1	-0.131210478	0.011415698	0.020759793
CCNE1	0.598279973	2.17E-37	1.97E-34
CCNE2	0.544095023	5.65E-30	8.16E-28
CCNF	0.527436572	5.92E-28	6.20E-26
CCNG1	-0.022138032	0.670820796	0.730997259
CCNG2	0.140744199	0.006621474	0.012708313
CCNH	0.008512424	0.870195209	0.897895178
CCNI2	0.470282079	8.19E-22	3.26E-20
CCNI	-0.041152953	0.429339821	0.505723876
CCNJL	0.353317566	2.39E-12	2.32E-11
CCNJ	0.275906459	6.60E-08	3.17E-07
CCNK	0.190984988	0.000215316	0.000558314
CCNL1	0.056217804	0.280128075	0.350789585
CCNL2	-0.01230281	0.813294438	0.850969107
CCNO	0.246225182	1.58E-06	6.00E-06
CCNT1	-0.072301599	0.164605677	0.221512719
CCNT2	0.061006225	0.241120899	0.308497241
CCNYL1	0.110599111	0.033201509	0.054132127
CCNY	-0.201673322	9.17E-05	0.000254797
CCPG1	-0.180098376	0.000490708	0.001189425
CCR10	0.242513221	2.29E-06	8.46E-06
CCR1	0.151548884	0.003432338	0.007011078
CCR2	0.179830458	0.000500467	0.001211459
CCR3	0.21064506	4.32E-05	0.000127708
CCR4	0.128439543	0.01329251	0.023788482
CCR5	0.245312995	1.73E-06	6.52E-06
CCR6	0.356600526	1.45E-12	1.44E-11
CCR7	0.111056063	0.032476867	0.053080986
CCR8	0.280371545	3.96E-08	1.98E-07
CCR9	-0.069496783	0.181647059	0.24102085
CCRL1	0.110440285	0.033456584	0.054507856
CCRL2	0.255027472	6.42E-07	2.61E-06
CCRN4L	0.079215095	0.127749724	0.177800057
CCS	-0.365026938	3.89E-13	4.26E-12
CCT2	0.453451275	3.26E-20	1.02E-18
CCT3	0.380782092	3.01E-14	3.91E-13

CCT4	0.300865095	3.36E-09	2.00E-08
CCT5	0.531673164	1.86E-28	2.04E-26
CCT6A	0.53365781	1.07E-28	1.22E-26
CCT6B	-0.452007452	4.42E-20	1.35E-18
CCT6P1	0.232556642	6.00E-06	2.07E-05
CCT7	0.261049527	3.40E-07	1.45E-06
CCT8L2	0.010296306	0.843313541	0.875894535
CCT8	0.438881303	6.72E-19	1.78E-17
CD101	0.349099573	4.50E-12	4.19E-11
CD109	0.306529785	1.64E-09	1.02E-08
CD14	-0.324145863	1.60E-10	1.16E-09
CD151	0.198955026	0.00011439	0.000312309
CD160	0.067937113	0.191670121	0.252676823
CD163L1	0.056037316	0.281677438	0.352501083
CD163	0.019554235	0.707358481	0.761935423
CD164L2	0.228917309	8.44E-06	2.83E-05
CD164	0.093006981	0.073570399	0.109664758
CD177	0.333193641	4.54E-11	3.61E-10
CD180	0.185087452	0.000338346	0.000845102
CD19	0.267980986	1.60E-07	7.21E-07
CD1A	0.391165367	5.16E-15	7.60E-14
CD1B	0.23991159	2.96E-06	1.07E-05
CD1C	0.186208913	0.000310806	0.000782001
CD1D	-0.053516625	0.303920936	0.376560289
CD1E	0.247301496	1.42E-06	5.43E-06
CD200R1L	0.023671399	0.649491702	0.712313443
CD200R1	0.14403067	0.005446431	0.010625127
CD200	0.176460929	0.000639607	0.00152001
CD207	0.279765062	4.25E-08	2.11E-07
CD209	0.072451762	0.163728348	0.220466113
CD226	0.068058303	0.190877081	0.251747978
CD22	0.278593114	4.86E-08	2.39E-07
CD244	0.076486081	0.141450949	0.194236684
CD247	0.09273237	0.074428546	0.11074721
CD248	0.246632152	1.52E-06	5.78E-06
CD24	0.44950494	7.50E-20	2.25E-18
CD274	0.043945095	0.398672888	0.474879597
CD276	0.393800021	3.27E-15	4.96E-14
CD27	0.211440511	4.04E-05	0.000119924
CD28	0.095639756	0.065745392	0.099368874
CD2AP	0.264107445	2.45E-07	1.07E-06
CD2BP2	0.309949167	1.06E-09	6.79E-09
CD2	0.246383173	1.56E-06	5.91E-06
CD300A	0.227935331	9.25E-06	3.08E-05
CD300C	0.224572865	1.26E-05	4.08E-05
CD300E	0.065229934	0.210019132	0.273750403
CD300LB	0.310991784	9.22E-10	5.99E-09
CD300LD	0.080135941	0.12336609	0.172603359
CD300LF	0.325952404	1.25E-10	9.23E-10
CD300LG	-0.497224799	1.45E-24	8.69E-23
CD302	-0.461642394	5.56E-21	1.98E-19
CD320	0.208549235	5.17E-05	0.00015029
CD33	0.181801241	0.000432697	0.00105834
CD34	-0.102534333	0.048439719	0.075967573
CD36	-0.329667866	7.45E-11	5.75E-10
CD37	0.28532038	2.22E-08	1.16E-07
CD38	0.187700728	0.000277404	0.000704903
CD3D	0.284309506	2.50E-08	1.30E-07

CD3EAP	0.302427175	2.76E-09	1.67E-08
CD3E	0.223755762	1.36E-05	4.37E-05
CD3G	0.170696309	0.000963385	0.002206295
CD40LG	0.163188371	0.00161165	0.003525948
CD40	0.03017576	0.562321471	0.632556089
CD44	0.105793163	0.041693921	0.066381411
CD46	-0.031701841	0.542714651	0.614176396
CD47	0.291886573	1.01E-08	5.60E-08
CD48	0.213490639	3.38E-05	0.000101662
CD4	0.124325169	0.016579912	0.028984293
CD52	0.268334395	1.54E-07	6.96E-07
CD53	0.253946485	7.18E-07	2.89E-06
CD55	-0.092969947	0.073685657	0.109820128
CD58	0.370586781	1.60E-13	1.87E-12
CD59	-0.002399792	0.96325658	0.971055439
CD5L	-0.227296465	9.81E-06	3.24E-05
CD5	0.224576738	1.26E-05	4.08E-05
CD63	0.326149056	1.21E-10	9.00E-10
CD68	0.223187604	1.43E-05	4.59E-05
CD69	0.119175667	0.021680034	0.036972234
CD6	0.24045409	2.80E-06	1.02E-05
CD70	0.240349949	2.83E-06	1.03E-05
CD72	0.141535527	0.006319482	0.012182768
CD74	0.209998277	4.57E-05	0.000134351
CD79A	0.208336533	5.26E-05	0.000152823
CD79B	0.063310843	0.22377217	0.289423436
CD7	0.28456426	2.43E-08	1.26E-07
CD80	0.289148921	1.41E-08	7.63E-08
CD81	-0.321005732	2.44E-10	1.73E-09
CD82	-0.083735154	0.107345603	0.152916633
CD83	0.323657539	1.71E-10	1.24E-09
CD84	0.163758527	0.001551043	0.003409079
CD86	0.293834983	8.00E-09	4.50E-08
CD8A	0.12657761	0.014701501	0.026024817
CD8B	0.131757465	0.01107422	0.020205203
CD93	0.070539464	0.175165834	0.233718688
CD96	0.170375579	0.000985226	0.002252429
CD97	0.412523191	1.12E-16	2.16E-15
CD99L2	-0.208793996	5.06E-05	0.000147439
CD99	0.000300326	0.99540008	0.99645045
CD9	0.170527387	0.000974832	0.002230202
CDADC1	-0.43224682	2.54E-18	6.17E-17
CDAN1	0.091438251	0.078582887	0.116275124
CDA	-0.035570117	0.494585963	0.568503735
CDC123	0.313349596	6.77E-10	4.48E-09
CDC14A	0.227167478	9.93E-06	3.28E-05
CDC14B	-0.577193204	2.44E-34	1.03E-31
CDC14C	-0.440433895	4.90E-19	1.32E-17
CDC16	0.221114014	1.72E-05	5.45E-05
CDC20B	0.286772422	1.87E-08	9.88E-08
CDC20	0.574286238	6.18E-34	2.14E-31
CDC23	0.27751335	5.50E-08	2.67E-07
CDC25A	0.534830994	7.75E-29	9.19E-27
CDC25B	0.512954106	2.75E-26	2.25E-24
CDC25C	0.553017579	4.20E-31	7.15E-29
CDC26	0.058853761	0.258156741	0.327391851
CDC27	0.25120113	9.54E-07	3.77E-06
CDC34	-0.071152223	0.171438022	0.229497936

CDC37L1	-0.459395829	9.08E-21	3.08E-19
CDC37	0.06422463	0.217145609	0.281896911
CDC40	-0.074011199	0.154823823	0.210008865
CDC42BPA	-0.274301343	7.92E-08	3.75E-07
CDC42BPB	-0.061914725	0.23417251	0.300766214
CDC42BPG	0.401146999	8.93E-16	1.48E-14
CDC42EP1	0.315362781	5.20E-10	3.50E-09
CDC42EP2	0.170219178	0.000996041	0.002274284
CDC42EP3	-0.018869339	0.717161535	0.770495179
CDC42EP4	-0.074965053	0.149560736	0.203883193
CDC42EP5	0.097772708	0.059918849	0.091534646
CDC42SE1	0.392382658	4.18E-15	6.24E-14
CDC42SE2	0.433276317	2.07E-18	5.10E-17
CDC42	0.172287772	0.00086147	0.001992824
CDC45	0.507007236	1.26E-25	9.05E-24
CDC5L	-0.049363049	0.343043359	0.417375705
CDC6	0.583674495	2.97E-35	1.60E-32
CDC73	0.010485344	0.840474949	0.873627357
CDC7	0.534714647	8.01E-29	9.38E-27
CDCA2	0.554757414	2.51E-31	4.54E-29
CDCA3	0.554786168	2.49E-31	4.54E-29
CDCA4	0.539984867	1.82E-29	2.36E-27
CDCA5	0.542046082	1.02E-29	1.37E-27
CDCA7L	0.386710837	1.11E-14	1.54E-13
CDCA7	0.528555981	4.36E-28	4.70E-26
CDCA8	0.558025455	9.43E-32	1.94E-29
CDCP1	0.499652645	7.98E-25	5.01E-23
CDCP2	-0.032804386	0.528763654	0.600708799
CDH10	0.211144594	4.14E-05	0.000122737
CDH11	0.30566204	1.83E-09	1.14E-08
CDH12	0.224519111	1.27E-05	4.10E-05
CDH13	-0.161145456	0.001847065	0.004002309
CDH15	0.11343063	0.028924763	0.047868344
CDH16	0.227569782	9.57E-06	3.17E-05
CDH17	0.309821709	1.07E-09	6.90E-09
CDH18	0.14289666	0.005828808	0.011302463
CDH19	-0.045996674	0.377000476	0.453237794
CDH1	0.098114858	0.059025146	0.090314821
CDH20	0.109100078	0.03567621	0.057694736
CDH22	-0.127616429	0.013900024	0.024753399
CDH23	-0.44644619	1.42E-19	4.09E-18
CDH24	0.344891339	8.41E-12	7.50E-11
CDH26	0.055299928	0.288067484	0.359556445
CDH2	-0.131571653	0.011189193	0.020392562
CDH3	0.278472336	4.93E-08	2.42E-07
CDH4	0.013171758	0.800375778	0.840055121
CDH5	-0.187232272	0.000287514	0.000727808
CDH6	0.280001889	4.13E-08	2.06E-07
CDH7	-0.013751991	0.791779383	0.833579711
CDH8	0.128738405	0.013077785	0.02345204
CDH9	0.109848477	0.034421783	0.055892962
CDHR1	0.053101489	0.307692752	0.380381942
CDHR2	0.271655264	1.07E-07	4.93E-07
CDHR3	-0.251802576	8.97E-07	3.55E-06
CDHR4	0.21006655	4.54E-05	0.000133734
CDHR5	-0.345683474	7.48E-12	6.72E-11
CDIPT	-0.11457174	0.027339803	0.045529011
CDK10	-0.176264599	0.000648727	0.001540215

CDK11A	0.052502027	0.31319353	0.386008507
CDK11B	0.073925786	0.155301855	0.210542604
CDK12	0.108225397	0.037191318	0.059877601
CDK13	-0.108807831	0.036176494	0.058385298
CDK14	-0.161955962	0.001750136	0.003806355
CDK15	-0.012353689	0.812536585	0.850354794
CDK16	0.590586856	2.99E-36	2.13E-33
CDK17	0.17698761	0.000615723	0.001466224
CDK18	0.078590825	0.130789496	0.181472931
CDK19	0.266781314	1.83E-07	8.16E-07
CDK1	0.601172554	7.96E-38	7.93E-35
CDK20	0.184223742	0.000361088	0.000897405
CDK2AP1	0.405152972	4.34E-16	7.51E-15
CDK2AP2	0.112996187	0.029548564	0.048775085
CDK2	0.329623452	7.49E-11	5.78E-10
CDK3	-0.076178692	0.143062148	0.196124698
CDK4	0.422189567	1.81E-17	3.90E-16
CDK5R1	0.263099479	2.73E-07	1.18E-06
CDK5R2	0.27562397	6.82E-08	3.26E-07
CDK5RAP1	0.19971469	0.000107556	0.000295264
CDK5RAP2	-0.062702913	0.228259766	0.294139137
CDK5RAP3	-0.094054235	0.070371449	0.1054804
CDK5	-0.086420135	0.096500143	0.139279566
CDK6	-0.232603994	5.97E-06	2.06E-05
CDK7	0.36136582	6.92E-13	7.27E-12
CDK8	0.080899344	0.119821393	0.168246531
CDK9	-0.048939237	0.347207963	0.421593042
CDKAL1	0.239775267	3.00E-06	1.09E-05
CDKL1	0.158847753	0.002149158	0.004596407
CDKL2	0.203542407	7.86E-05	0.000221177
CDKL3	0.099260135	0.056113493	0.086430571
CDKL4	0.239618078	3.04E-06	1.10E-05
CDKL5	-0.100254763	0.053682432	0.083098677
CDKN1A	0.036700571	0.480962759	0.555237879
CDKN1B	-0.138896752	0.007377376	0.014013938
CDKN1C	0.24991521	1.09E-06	4.26E-06
CDKN2AIPN	0.28297029	2.93E-08	1.50E-07
CDKN2AIP	-0.068412751	0.188571457	0.249020321
CDKN2A	0.207332889	5.73E-05	0.000165287
CDKN2BAS	0.230673243	7.16E-06	2.43E-05
CDKN2B	0.080709085	0.120697296	0.169345133
CDKN2C	0.155309211	0.002703404	0.0056543
CDKN2D	0.386267185	1.20E-14	1.64E-13
CDKN3	0.558217598	8.90E-32	1.87E-29
CDNF	-0.41115419	1.45E-16	2.71E-15
CDO1	-0.469502092	9.76E-22	3.84E-20
CDON	-0.006498966	0.900715283	0.9219738
CDR1	0.266733592	1.84E-07	8.20E-07
CDR2L	0.286122191	2.02E-08	1.06E-07
CDR2	0.094031563	0.070439478	0.105566485
CDRT15P	0.117874517	0.023166149	0.039284646
CDRT15	0.078747191	0.130022907	0.180585357
CDRT1	0.013825538	0.790691529	0.832698453
CDRT4	0.237656316	3.68E-06	1.31E-05
CDS1	0.296281457	5.93E-09	3.40E-08
CDS2	-0.086691894	0.095452917	0.13789865
CDSN	0.179772045	0.000502619	0.001216371
CDT1	0.52011532	4.22E-27	3.85E-25

CDV3	0.238398096	3.43E-06	1.23E-05
CDX1	0.174292489	0.000747264	0.001750793
CDX2	0.288879605	1.46E-08	7.84E-08
CDY2B	0.034298789	0.510148938	0.582985553
CDYL2	0.037776293	0.468190488	0.543042087
CDYL	0.238126761	3.52E-06	1.26E-05
CEACAM16	0.211497378	4.02E-05	0.000119393
CEACAM18	0.031482181	0.545515771	0.616715763
CEACAM19	0.138340404	0.007619649	0.014428157
CEACAM1	-0.097609849	0.060348149	0.092148067
CEACAM20	0.122191926	0.018549157	0.032086161
CEACAM21	0.138896038	0.007377682	0.014013938
CEACAM22	-0.182342377	0.000415641	0.001019753
CEACAM3	0.182786614	0.000402109	0.0009896
CEACAM4	0.180860027	0.000463921	0.001129168
CEACAM5	0.26370492	2.55E-07	1.11E-06
CEACAM6	0.233685076	5.39E-06	1.88E-05
CEACAM7	0.348014519	5.30E-12	4.85E-11
CEACAM8	0.062330513	0.231040065	0.297337221
CEBPA	0.124850725	0.016123792	0.028258836
CEBPB	-0.178450084	0.000553668	0.001329887
CEBPD	-0.063126477	0.22512643	0.290740874
CEBPE	0.156202437	0.002552401	0.00538084
CEBPG	0.145710556	0.004921368	0.009712084
CEBPZ	-0.048207097	0.354477492	0.42934707
CECR1	0.094107697	0.070211242	0.10527987
CECR2	-0.267989948	1.60E-07	7.20E-07
CECR4	-0.220674296	1.79E-05	5.66E-05
CECR5	-0.154702348	0.002810586	0.00585267
CECR6	0.336942638	2.67E-11	2.19E-10
CECR7	0.129727913	0.012388493	0.022355396
CELA1	0.164147912	0.001510862	0.003329947
CELA2A	0.10523019	0.042799214	0.067896636
CELA2B	0.094338292	0.06952364	0.104390259
CELA3A	0.1448183	0.005194282	0.010189098
CELA3B	0.091058713	0.079836293	0.117910789
CELF1	-0.114885924	0.026916739	0.04489921
CELF2	0.185093445	0.000338193	0.000844825
CELF3	0.149997818	0.003781778	0.00764879
CELF4	0.279734001	4.26E-08	2.12E-07
CELF5	0.079020796	0.128689905	0.178948646
CELF6	-0.272305511	9.91E-08	4.60E-07
CELP	0.08199895	0.114855862	0.162210018
CELSR1	0.047204476	0.364586751	0.440173157
CELSR2	0.076973574	0.138924257	0.191241643
CELSR3	0.090391109	0.082080255	0.120771295
CEL	0.0643882	0.215974433	0.280641405
CEMP1	-0.008262973	0.873966901	0.90068639
CEND1	0.318767932	3.30E-10	2.29E-09
CENPA	0.612953036	1.20E-39	1.70E-36
CENPBD1	-0.207418712	5.68E-05	0.000164214
CENPB	0.136826778	0.008315124	0.015612997
CENPC1	-0.213744331	3.31E-05	9.96E-05
CENPE	0.55505415	2.30E-31	4.27E-29
CENPF	0.57825566	1.73E-34	7.68E-32
CENPH	0.514898238	1.66E-26	1.38E-24
CENPI	0.457180796	1.46E-20	4.84E-19
CENPJ	0.428003466	5.86E-18	1.35E-16

CENPK	0.563110743	2.02E-32	4.62E-30
CENPL	0.496391823	1.78E-24	1.05E-22
CENPM	0.509716929	6.33E-26	4.85E-24
CENPN	0.018865341	0.717218902	0.770515259
CENPO	0.518410629	6.62E-27	5.81E-25
CENPP	0.277662492	5.41E-08	2.63E-07
CENPQ	0.346416022	6.71E-12	6.09E-11
CENPT	0.136568431	0.008439322	0.015814897
CENPV	0.016492693	0.751531002	0.799103364
CENPW	0.548076057	1.79E-30	2.72E-28
CEP110	0.237366752	3.79E-06	1.35E-05
CEP120	-0.119935413	0.02085108	0.035680744
CEP135	0.371943846	1.29E-13	1.52E-12
CEP152	0.323138331	1.83E-10	1.32E-09
CEP164	0.188878119	0.000253441	0.000647811
CEP170L	0.071353901	0.170224114	0.228118176
CEP170	0.234318105	5.07E-06	1.77E-05
CEP192	0.187667028	0.00027812	0.000706272
CEP250	0.315253723	5.27E-10	3.55E-09
CEP290	0.26948979	1.36E-07	6.18E-07
CEP350	-0.027037611	0.603677962	0.670110457
CEP55	0.663348623	2.14E-48	2.14E-44
CEP57	-0.009996782	0.847815325	0.879742534
CEP63	-0.037938195	0.46628452	0.54120952
CEP68	0.04906531	0.345965765	0.420392191
CEP70	-0.112201544	0.030719407	0.050476156
CEP72	0.508417807	8.82E-26	6.48E-24
CEP76	-0.081423261	0.117435028	0.165373621
CEP78	0.376803868	5.83E-14	7.27E-13
CEP97	0.099078588	0.056566937	0.08704824
CEPT1	-0.323649409	1.71E-10	1.24E-09
CER1	0.150748058	0.003608924	0.007337924
CERCAM	0.339142582	1.94E-11	1.63E-10
CERKL	0.250811284	9.93E-07	3.91E-06
CERK	-0.208361577	5.25E-05	0.000152521
CES1	-0.370787521	1.55E-13	1.82E-12
CES2	-0.550254214	9.48E-31	1.50E-28
CES3	-0.418394454	3.73E-17	7.66E-16
CES4	-0.267736343	1.65E-07	7.39E-07
CES7	-0.423948791	1.29E-17	2.82E-16
CES8	-0.398849007	1.34E-15	2.15E-14
CETN1	-0.016388465	0.753050149	0.800547762
CETN2	0.008610651	0.868710821	0.89658982
CETN3	0.018033453	0.729188767	0.780470565
CETN4P	-0.062475276	0.229956446	0.296114816
CETP	-0.278953198	4.66E-08	2.30E-07
CFB	-0.281190891	3.60E-08	1.82E-07
CFC1B	0.064289954	0.216677335	0.281379693
CFDP1	-0.110858424	0.032788614	0.053529031
CFD	0.186053537	0.000314493	0.000790179
CFHR1	-0.258055249	4.67E-07	1.94E-06
CFHR2	-0.411507533	1.35E-16	2.56E-15
CFHR3	-0.297218626	5.28E-09	3.05E-08
CFHR4	-0.486305646	2.03E-23	1.02E-21
CFHR5	-0.377272615	5.39E-14	6.77E-13
CFH	-0.227565197	9.57E-06	3.17E-05
CFI	-0.42573262	9.13E-18	2.04E-16
CFL1	0.420928757	2.30E-17	4.87E-16

CFL2	-0.455658631	2.03E-20	6.54E-19
CFLAR	0.025949452	0.618329693	0.683328571
CFLP1	-0.009304257	0.858242557	0.888757055
CFP	-0.078090281	0.133266923	0.18444794
CFTR	0.272190741	1.00E-07	4.66E-07
CG030	0.139307794	0.007202825	0.013711865
CGA	0.074802324	0.150448878	0.204927122
CGB1	0.131744448	0.01108224	0.020217984
CGB2	0.101205518	0.051441113	0.080013262
CGB5	0.157266848	0.002382513	0.005052631
CGB7	0.211370272	4.06E-05	0.000120596
CGB8	-0.020546589	0.693240406	0.749972054
CGB	0.07219621	0.165223512	0.222209061
CGGBP1	0.055004755	0.290652467	0.362351593
CGNL1	-0.346294252	6.84E-12	6.18E-11
CGN	-0.097985851	0.059360814	0.090758721
CGREF1	-0.083854058	0.106845784	0.152299415
CGRRF1	0.013321994	0.798147609	0.838600109
CH25H	-0.123350628	0.017455626	0.030355357
CHAC1	0.040517046	0.436510379	0.512650945
CHAC2	0.044332578	0.394523353	0.470855562
CHADL	-0.274665589	7.60E-08	3.60E-07
CHAD	-0.441386373	4.03E-19	1.10E-17
CHAF1A	0.407432422	2.87E-16	5.14E-15
CHAF1B	0.616783301	2.94E-40	6.50E-37
CHAT	0.025092757	0.629972522	0.694538605
CHCHD10	-0.033271496	0.522908415	0.595006937
CHCHD1	0.102118195	0.049363484	0.077173297
CHCHD2	0.119488594	0.021335203	0.036434075
CHCHD3	0.199237152	0.000111805	0.000305987
CHCHD4	0.089361004	0.085642335	0.125388888
CHCHD5	-0.120292616	0.020470942	0.035081471
CHCHD6	0.109077739	0.035714243	0.05774218
CHCHD7	0.100744254	0.052518564	0.081504621
CHCHD8	0.008408436	0.871767159	0.899065931
CHD1L	0.150129149	0.003750989	0.007598089
CHD1	0.074814957	0.150379785	0.204859551
CHD2	-0.076904133	0.139282047	0.191667952
CHD3	0.407399404	2.88E-16	5.15E-15
CHD4	0.284503311	2.45E-08	1.27E-07
CHD5	0.252566013	8.29E-07	3.30E-06
CHD6	0.049970293	0.337131902	0.411187814
CHD7	0.114236848	0.027797028	0.046186187
CHD8	0.188982061	0.000251421	0.000643143
CHD9	-0.29111939	1.11E-08	6.11E-08
CHDH	-0.175579475	0.000681507	0.001609982
CHEK1	0.355497898	1.71E-12	1.70E-11
CHEK2	0.241059898	2.64E-06	9.68E-06
CHERP	-0.136941623	0.008260439	0.015519092
CHFR	0.307264269	1.49E-09	9.39E-09
CHGA	0.212638865	3.64E-05	0.000109001
CHGB	0.182625112	0.00040698	0.001000353
CHI3L1	-0.141591011	0.006298778	0.012145205
CHI3L2	0.219347736	2.02E-05	6.30E-05
CHIA	0.177475887	0.000594322	0.001419673
CHIC1	0.037668409	0.469462914	0.544264442
CHIC2	0.131572593	0.011188608	0.020392562
CHID1	-0.077315143	0.137174622	0.189081355

CHIT1	0.147696029	0.004359876	0.008704932
CHKA	0.184212932	0.000361382	0.00089791
CHKB-CPT1	0.159001939	0.002127554	0.004555582
CHKB	0.118396659	0.02255952	0.038344063
CHL1	0.147948725	0.004292727	0.008587177
CHML	0.437240578	9.36E-19	2.43E-17
CHMP1A	0.006117095	0.906521624	0.926519793
CHMP1B	-0.096327252	0.063818599	0.096801746
CHMP2A	-0.020400348	0.695314437	0.751766755
CHMP2B	0.282946941	2.93E-08	1.50E-07
CHMP4A	0.158299179	0.002227657	0.004747473
CHMP4B	0.419049465	3.29E-17	6.84E-16
CHMP4C	0.364371531	4.32E-13	4.69E-12
CHMP5	-0.033569002	0.519196525	0.591628048
CHMP6	-0.120854039	0.019885637	0.034145979
CHMP7	-0.021370066	0.681604674	0.739915449
CHM	-0.063703762	0.220905376	0.286124237
CHN1	0.033208987	0.523690045	0.595726207
CHN2	-0.576155524	3.40E-34	1.36E-31
CHODL	-0.024367876	0.639896114	0.703687921
CHORDC1	0.395769439	2.32E-15	3.58E-14
CHP2	0.210172384	4.50E-05	0.000132662
CHPF2	0.176102258	0.00065636	0.001556296
CHPF	-0.00884177	0.865220003	0.894169887
CHPT1	-0.260838132	3.48E-07	1.48E-06
CHP	-0.234469222	5.00E-06	1.75E-05
CHRAC1	0.316073295	4.73E-10	3.21E-09
CHRDL1	0.255704668	5.98E-07	2.44E-06
CHRDL2	0.313956959	6.25E-10	4.16E-09
CHRD	-0.058432304	0.261587385	0.331131267
CHRFAM7A	0.274297784	7.92E-08	3.75E-07
CHRM1	0.235446997	4.55E-06	1.60E-05
CHRM2	-0.112405851	0.03041465	0.050055403
CHRM3	-0.043504392	0.403424019	0.479621252
CHRM4	0.045932138	0.377671	0.453879572
CHRM5	-0.152438288	0.003245431	0.006662765
CHRNA10	0.218445086	2.19E-05	6.79E-05
CHRNA1	0.237708091	3.66E-06	1.31E-05
CHRNA2	-0.018219661	0.726503616	0.778056394
CHRNA3	0.140807317	0.006596921	0.012667359
CHRNA4	-0.16453808	0.001471559	0.003253763
CHRNA5	0.333058342	4.63E-11	3.67E-10
CHRNA6	0.235329828	4.61E-06	1.62E-05
CHRNA7	0.262891755	2.79E-07	1.21E-06
CHRNA9	0.049945886	0.337368238	0.411421999
CHRNB1	-0.084092007	0.105851064	0.151090127
CHRNB2	0.126369303	0.014867011	0.026268789
CHRNB3	0.069376987	0.182402909	0.241775907
CHRNB4	-0.223091052	1.44E-05	4.63E-05
CHRND	0.169695273	0.001033071	0.002351559
CHRNE	-0.15138332	0.00346819	0.00707633
CHRNG	0.077357275	0.136959982	0.188837758
CHST10	0.388439522	8.26E-15	1.17E-13
CHST11	0.478025206	1.40E-22	6.26E-21
CHST12	0.309401099	1.13E-09	7.25E-09
CHST13	0.002257736	0.965430213	0.972902054
CHST14	0.046683725	0.369907486	0.445732592
CHST15	-0.164027834	0.00152315	0.003353691

CHST1	0.358110853	1.15E-12	1.17E-11
CHST2	0.224665606	1.25E-05	4.05E-05
CHST3	0.197484625	0.000128791	0.000348185
CHST4	0.301310142	3.18E-09	1.90E-08
CHST5	0.053823562	0.301151932	0.373570909
CHST6	0.282112343	3.23E-08	1.65E-07
CHST7	0.06720709	0.196498341	0.258289783
CHST8	0.083619801	0.107832259	0.153522065
CHST9	-0.074712115	0.150942943	0.20550026
CHSY1	0.131023185	0.011534744	0.02095142
CHSY3	-0.05732734	0.270730268	0.340667146
CHTF18	0.386686772	1.11E-14	1.54E-13
CHTF8	-0.304549852	2.11E-09	1.30E-08
CHUK	-0.076351546	0.142154409	0.195054765
CHURC1	-0.198294824	0.000120658	0.000328291
CIAO1	-0.028838983	0.579771334	0.648487143
CIAPIN1	0.115160878	0.026551133	0.044349096
CIB1	-0.072557397	0.163113278	0.219727008
CIB2	0.434895581	1.50E-18	3.81E-17
CIB3	0.165298375	0.001397656	0.003104483
CIB4	0.120739999	0.020003336	0.034336245
CIC	0.163512142	0.001576972	0.003459443
CIDEA	-0.027261295	0.600685495	0.667491074
CIDEB	-0.495540047	2.19E-24	1.28E-22
CIDECP	0.301963419	2.93E-09	1.76E-08
CIDEC	0.107068578	0.039278636	0.062887487
CIITA	0.202922554	8.27E-05	0.000231787
CILP2	0.344724022	8.62E-12	7.66E-11
CILP	0.174171253	0.000753752	0.001763919
CINP	0.003048502	0.953334199	0.963734912
CIR1	0.069073802	0.184326207	0.244093772
CIRBP	-0.219087438	2.07E-05	6.44E-05
CIRH1A	0.094502968	0.069035976	0.103751864
CISD1	0.068474673	0.188170775	0.248572022
CISD2	-0.12432591	0.01657926	0.028984293
CISD3	-0.19057748	0.000222244	0.000574973
CISH	-0.232861447	5.83E-06	2.02E-05
CITED1	0.061829433	0.234818765	0.301518494
CITED2	-0.105116835	0.043024726	0.068200078
CITED4	0.079655475	0.125638497	0.175334601
CIT	0.474676613	3.03E-22	1.28E-20
CIZ1	0.269713962	1.32E-07	6.04E-07
CKAP2L	0.493295999	3.79E-24	2.12E-22
CKAP2	0.349071332	4.52E-12	4.20E-11
CKAP4	0.475328316	2.61E-22	1.12E-20
CKAP5	0.401123522	8.97E-16	1.48E-14
CKB	0.061549881	0.236945742	0.303916628
CKLF	0.349069335	4.53E-12	4.20E-11
CKMT1A	0.351263577	3.26E-12	3.09E-11
CKMT1B	0.388661166	7.95E-15	1.13E-13
CKMT2	0.11528272	0.02639049	0.044114058
CKM	0.282049522	3.26E-08	1.66E-07
CKS1B	0.266581684	1.87E-07	8.32E-07
CKS2	0.541755775	1.10E-29	1.47E-27
CLASP1	-0.123006693	0.017774185	0.030855465
CLASP2	0.042415358	0.415308614	0.491316996
CLCA1	0.098194974	0.058817483	0.090045484
CLCA2	0.049944066	0.337385865	0.411421999

CLCA3P	0.169660448	0.001035577	0.002355928
CLCA4	0.108239212	0.03716697	0.059848075
CLCC1	-0.014496508	0.780786022	0.823965417
CLCF1	0.328983163	8.19E-11	6.27E-10
CLCN1	0.035387657	0.496803902	0.570747208
CLCN2	0.284672375	2.40E-08	1.25E-07
CLCN3	-0.238677642	3.33E-06	1.20E-05
CLCN4	0.003923632	0.939960306	0.953796629
CLCN5	0.000788181	0.987928302	0.990862792
CLCN6	0.01634312	0.75371136	0.800951497
CLCN7	0.057311471	0.270863144	0.340751172
CLCNKA	0.289600803	1.34E-08	7.24E-08
CLCNKB	0.226384245	1.07E-05	3.51E-05
CLC	0.050488629	0.332137881	0.406166035
CLDN10	0.161068269	0.001856547	0.004021544
CLDN11	0.214263767	3.16E-05	9.55E-05
CLDN12	-0.276556291	6.13E-08	2.96E-07
CLDN14	-0.34222878	1.24E-11	1.08E-10
CLDN15	-0.195399706	0.000152149	0.000406042
CLDN16	-0.21005734	4.54E-05	0.000133814
CLDN18	0.393246844	3.60E-15	5.42E-14
CLDN19	0.045755105	0.379514107	0.455654796
CLDN1	0.056005128	0.281954357	0.352783612
CLDN20	0.116281147	0.025105336	0.042167482
CLDN22	-0.026650786	0.608868647	0.674744461
CLDN23	-0.14229999	0.006039527	0.011675834
CLDN25	0.045887916	0.378130885	0.454288734
CLDN2	-0.284442968	2.46E-08	1.28E-07
CLDN3	0.003404875	0.94788626	0.959688473
CLDN4	0.321473964	2.29E-10	1.63E-09
CLDN5	-0.040299673	0.438977164	0.514730694
CLDN6	-0.028043917	0.590268412	0.65790127
CLDN7	0.192127052	0.000196962	0.000514337
CLDN8	-0.088277584	0.089521963	0.130283918
CLDN9	-0.121116032	0.019617514	0.033735012
CLDND1	0.082418122	0.113005999	0.159939295
CLDND2	-0.159370382	0.00207673	0.004456815
CLEC10A	0.127797833	0.013764077	0.024542016
CLEC11A	0.222622305	1.50E-05	4.81E-05
CLEC12A	0.174437675	0.000739563	0.001734177
CLEC12B	0.083804413	0.107054245	0.152534306
CLEC14A	-0.118279505	0.02269442	0.038543754
CLEC16A	0.005704558	0.912799853	0.93174149
CLEC17A	0.163992119	0.001526822	0.003360663
CLEC18A	0.105677137	0.041919727	0.066665986
CLEC18B	0.131650401	0.011140339	0.020314675
CLEC18C	0.023788116	0.647879548	0.710975893
CLEC1A	-0.163502742	0.001577969	0.003460624
CLEC1B	-0.072838117	0.161487201	0.217831134
CLEC2A	0.117109843	0.024079994	0.040636437
CLEC2B	0.150114823	0.003754336	0.007603323
CLEC2D	0.461347888	5.93E-21	2.10E-19
CLEC2L	0.216454695	2.61E-05	7.99E-05
CLEC3A	0.106833442	0.039714806	0.063519458
CLEC3B	-0.300034245	3.73E-09	2.20E-08
CLEC4A	0.308895385	1.21E-09	7.70E-09
CLEC4C	0.084621866	0.103662339	0.148327308
CLEC4D	0.157782231	0.002304015	0.004895541

CLEC4E	0.155770954	0.002624359	0.005515029
CLEC4F	0.159698741	0.002032372	0.004369623
CLEC4GP1	0.007098353	0.891612361	0.914613123
CLEC4G	-0.131349332	0.011328143	0.020619394
CLEC4M	-0.180620413	0.000472198	0.001147771
CLEC5A	0.386627141	1.13E-14	1.56E-13
CLEC6A	0.252586355	8.27E-07	3.29E-06
CLEC7A	0.252224528	8.58E-07	3.41E-06
CLEC9A	0.028843421	0.579712989	0.648469887
CLECL1	0.334557219	3.75E-11	3.01E-10
CLGN	0.297654099	5.01E-09	2.90E-08
CLIC1	0.460471556	7.18E-21	2.50E-19
CLIC2	0.123295646	0.017506214	0.030438016
CLIC3	0.322215221	2.08E-10	1.49E-09
CLIC4	-0.078718677	0.130162435	0.180703556
CLIC5	0.081908394	0.115258601	0.162676716
CLIC6	0.258933086	4.26E-07	1.78E-06
CLINT1	0.147578841	0.004391337	0.008761565
CLIP1	-0.023952563	0.645610919	0.708853409
CLIP2	0.249306817	1.16E-06	4.51E-06
CLIP3	0.293060685	8.79E-09	4.92E-08
CLIP4	0.06810031	0.190602754	0.251419457
CLK1	-0.115557298	0.026031536	0.043579854
CLK2P	0.270842146	1.17E-07	5.37E-07
CLK2	0.298429114	4.55E-09	2.65E-08
CLK3	0.110959473	0.032628907	0.053294505
CLK4	-0.020922159	0.687924464	0.745354391
CLLU1OS	0.266529415	1.88E-07	8.37E-07
CLLU1	0.045917684	0.377821271	0.45400539
CLMN	-0.366560808	3.05E-13	3.40E-12
CLN3	0.178829711	0.000538536	0.001296679
CLN5	-0.211836766	3.90E-05	0.00011613
CLN6	0.302942136	2.59E-09	1.57E-08
CLN8	0.152334832	0.003266687	0.006701569
CLNK	0.053423959	0.304760216	0.377388924
CLNS1A	0.049105254	0.345572789	0.419991526
CLOCK	-0.040895036	0.432239864	0.50831698
CLP1	-0.091345321	0.078888302	0.116623089
CLPB	-0.145034678	0.005126876	0.010072744
CLPP	-0.091516004	0.078328093	0.115941174
CLPS	0.044534061	0.392376005	0.468724277
CLPTM1L	0.155505551	0.002669531	0.005597586
CLPTM1	-0.016819346	0.74677619	0.795363553
CLPX	-0.35729365	1.30E-12	1.31E-11
CLRN1OS	-0.174965211	0.000712193	0.001676313
CLRN1	-0.141504275	0.00633117	0.01220412
CLRN2	-0.190885549	0.000216988	0.000562137
CLRN3	-0.03286952	0.527945197	0.599915823
CLSPN	0.463952717	3.35E-21	1.23E-19
CLSTN1	0.383803216	1.82E-14	2.43E-13
CLSTN2	-0.003878933	0.940643031	0.954294977
CLSTN3	0.104404806	0.044464236	0.070308478
CLTA	0.318784439	3.30E-10	2.28E-09
CLTB	0.231146202	6.85E-06	2.33E-05
CLTCL1	-0.255240922	6.28E-07	2.55E-06
CLTC	0.095669164	0.065662004	0.099276958
CLUAP1	-0.124184192	0.016704166	0.029175906
CLUL1	0.126373318	0.014863806	0.026265454

CLU	-0.254929331	6.49E-07	2.63E-06
CLVS1	-0.018637575	0.720489448	0.773403243
CLVS2	-0.015643137	0.763940925	0.809718795
CLYBL	-0.329541574	7.58E-11	5.84E-10
CMA1	-0.15947171	0.002062948	0.004430102
CMAH	0.059166428	0.255631802	0.324478927
CMAS	0.088902284	0.087268122	0.127460082
CMBL	-0.329501802	7.62E-11	5.87E-10
CMC1	-0.00713769	0.891015434	0.914142006
CMIP	-0.09303141	0.073494452	0.109576147
CMKLR1	0.124134336	0.016748302	0.029247868
CMPK1	-0.123443004	0.017370919	0.030226522
CMPK2	0.073980654	0.154994649	0.210211954
CMTM1	0.242413717	2.31E-06	8.54E-06
CMTM2	0.097557611	0.060486387	0.092302551
CMTM3	0.388032579	8.85E-15	1.25E-13
CMTM4	0.255760164	5.95E-07	2.43E-06
CMTM5	-0.074706778	0.150972212	0.205526063
CMTM6	0.111105628	0.032399084	0.052966892
CMTM7	0.421942795	1.90E-17	4.07E-16
CMTM8	-0.094354914	0.069474287	0.104331883
CMYA5	0.096897355	0.062256262	0.09468427
CN5H6.4	0.204934122	7.00E-05	0.000198806
CNBD1	0.047215064	0.364479069	0.440069819
CNBP	-0.161291554	0.001829238	0.003965838
CNDP1	-0.188792713	0.000255112	0.000651665
CNDP2	-0.207128571	5.82E-05	0.000167986
CNFN	0.28030723	3.99E-08	1.99E-07
CNGA1	-0.114984962	0.026784553	0.04469776
CNGA2	0.083884881	0.106716515	0.152151035
CNGA3	0.232472714	6.05E-06	2.08E-05
CNGA4	0.023785145	0.647920566	0.710981742
CNGB1	0.298598425	4.45E-09	2.60E-08
CNGB3	0.245807544	1.65E-06	6.23E-06
CNIH2	0.237761279	3.64E-06	1.30E-05
CNIH3	0.106259658	0.040796369	0.065113385
CNIH4	0.398943311	1.32E-15	2.12E-14
CNIH	-0.175944339	0.000663864	0.001572592
CNKSR1	0.233235938	5.62E-06	1.95E-05
CNKSR2	-0.104998401	0.043261408	0.068537079
CNKSR3	-0.107901212	0.037766557	0.06069092
CNN1	0.089132565	0.086448888	0.126411828
CNN2	0.137721656	0.007897416	0.014907364
CNN3	-0.125595938	0.01549588	0.027275926
CNNM1	-0.005453594	0.916621846	0.934780672
CNNM2	-0.199337449	0.0001109	0.000303649
CNNM3	-0.283336282	2.80E-08	1.44E-07
CNNM4	0.222595515	1.51E-05	4.82E-05
CNOT10	0.298269468	4.64E-09	2.70E-08
CNOT1	-0.129542225	0.012515347	0.022559785
CNOT2	0.407944248	2.61E-16	4.70E-15
CNOT3	0.360458538	7.97E-13	8.29E-12
CNOT4	-0.19942112	0.00011015	0.00030201
CNOT6L	-0.244214765	1.93E-06	7.23E-06
CNOT6	0.581192826	6.70E-35	3.25E-32
CNOT7	0.082661226	0.111943906	0.158593835
CNOT8	0.087006219	0.094252992	0.136401867
CNO	0.283389977	2.79E-08	1.43E-07

CNPY1	0.084700343	0.103341233	0.147952859
CNPY2	0.112514476	0.030253675	0.049827522
CNPY3	0.155029085	0.002752408	0.005742927
CNPY4	0.352655254	2.64E-12	2.54E-11
CNP	0.099605833	0.055258389	0.085265093
CNR1	0.024654031	0.635970864	0.700183009
CNR2	0.079506496	0.126349669	0.176171479
CNRIP1	0.045521309	0.381956612	0.458200736
CNST	-0.371251427	1.44E-13	1.69E-12
CNTD1	0.28061018	3.85E-08	1.93E-07
CNTD2	0.188531201	0.000260292	0.000663877
CNTFR	0.16312803	0.001618189	0.003539478
CNTF	0.01832052	0.725050605	0.77691793
CNTLN	0.000362718	0.994444478	0.995844118
CNTN1	0.183503252	0.00038114	0.000942068
CNTN2	0.15835135	0.00222008	0.004733352
CNTN3	0.056646555	0.276470697	0.346885579
CNTN4	0.007545182	0.884835531	0.909065724
CNTN5	0.117446475	0.023673926	0.040036669
CNTN6	0.020436362	0.694803469	0.751255072
CNTNAP1	0.37588743	6.77E-14	8.39E-13
CNTNAP2	-0.019793184	0.703949595	0.759130185
CNTNAP3	-0.172957334	0.000821646	0.001908456
CNTNAP4	-0.044331178	0.394538294	0.470855562
CNTNAP5	0.178939888	0.000534217	0.001286902
CNTROB	0.155338585	0.002698311	0.005646613
COASY	0.014607312	0.779153587	0.822460539
COBLL1	-0.531996959	1.70E-28	1.91E-26
COBL	-0.23223988	6.18E-06	2.13E-05
COBRA1	0.203767146	7.71E-05	0.000217437
COCH	0.244373554	1.90E-06	7.13E-06
COG1	0.158228717	0.002237927	0.00476783
COG2	0.281791258	3.36E-08	1.70E-07
COG3	-0.312142441	7.93E-10	5.20E-09
COG4	-0.058686101	0.259517748	0.328866648
COG5	-0.01979758	0.703886931	0.759130185
COG6	-0.07517961	0.148395825	0.202475284
COG7	-0.06177232	0.235252211	0.302016661
COG8	-0.330026784	7.08E-11	5.49E-10
COIL	0.238417397	3.42E-06	1.23E-05
COL10A1	0.297102766	5.36E-09	3.09E-08
COL11A1	0.420733763	2.39E-17	5.03E-16
COL11A2	0.069339305	0.182641138	0.242055398
COL12A1	0.154210228	0.002900325	0.006020033
COL13A1	0.199365498	0.000110648	0.000303128
COL14A1	0.111795469	0.031332892	0.051384086
COL15A1	0.054671166	0.293592524	0.365422358
COL16A1	0.353936875	2.17E-12	2.12E-11
COL17A1	0.140817551	0.006592947	0.01266095
COL18A1	-0.430527478	3.57E-18	8.45E-17
COL19A1	0.064180951	0.217459123	0.282247746
COL1A1	0.342183819	1.25E-11	1.08E-10
COL1A2	0.307221852	1.50E-09	9.43E-09
COL20A1	0.075719372	0.145495781	0.198940765
COL21A1	-0.029347161	0.573107985	0.642433922
COL22A1	0.494375059	2.92E-24	1.66E-22
COL23A1	0.068140389	0.190341291	0.251091194
COL24A1	0.231050588	6.91E-06	2.35E-05

COL25A1	-0.326889574	1.09E-10	8.20E-10
COL27A1	0.026831276	0.60644425	0.672486465
COL28A1	0.161356561	0.001821357	0.003950328
COL29A1	0.101179639	0.05150107	0.080094014
COL2A1	0.211938863	3.87E-05	0.0001152
COL3A1	0.26171016	3.17E-07	1.36E-06
COL4A1	0.181849073	0.000431163	0.001055108
COL4A2	0.232812907	5.85E-06	2.02E-05
COL4A3BP	-0.179883792	0.00049851	0.001207162
COL4A3	0.126507702	0.014756865	0.026099632
COL4A4	0.058213743	0.263378767	0.332827897
COL4A5	0.230847354	7.05E-06	2.40E-05
COL4A6	0.055830307	0.283461582	0.354362552
COL5A1	0.310033142	1.04E-09	6.72E-09
COL5A2	0.303380705	2.45E-09	1.49E-08
COL5A3	-0.186947231	0.000293832	0.000742105
COL6A1	0.187683221	0.000277776	0.000705488
COL6A2	0.24424295	1.93E-06	7.22E-06
COL6A3	0.248984136	1.20E-06	4.64E-06
COL6A4P2	0.256654682	5.41E-07	2.22E-06
COL6A6	0.059214946	0.25524154	0.324066149
COL7A1	0.003443884	0.947290053	0.959279847
COL8A1	0.039210896	0.451452188	0.527188188
COL8A2	0.336593567	2.80E-11	2.29E-10
COL9A1	0.075411288	0.147145718	0.200962295
COL9A2	0.52686841	6.90E-28	7.20E-26
COL9A3	0.092044008	0.076615486	0.113642597
COLEC10	-0.13003072	0.012184062	0.022021728
COLEC11	0.027540736	0.596956502	0.664055359
COLEC12	0.171514591	0.000909671	0.002091455
COLQ	0.012933425	0.803913821	0.843146678
COMMD10	0.165579011	0.00137125	0.003049229
COMMD1	-0.107972057	0.037640208	0.060507401
COMMD2	0.303090644	2.54E-09	1.54E-08
COMMD3	0.062453577	0.230118641	0.296266225
COMMD4	0.070803613	0.173551522	0.231875355
COMMD5	0.266842515	1.82E-07	8.11E-07
COMMD6	-0.042420886	0.415247778	0.491274199
COMMD7	0.298155612	4.71E-09	2.74E-08
COMMD8	0.319307776	3.07E-10	2.14E-09
COMMD9	0.174958388	0.000712541	0.001676934
COMP	0.332907401	4.73E-11	3.74E-10
COMTD1	0.188819913	0.000254578	0.000650553
COMT	-0.101615853	0.050498174	0.078718671
COPA	0.16388427	0.001537962	0.003383312
COPB1	0.176103709	0.000656291	0.001556296
COPB2	0.076066376	0.143654339	0.196746992
COPE	0.255248457	6.27E-07	2.55E-06
COPG2	0.155702578	0.002635929	0.005534091
COPG	0.237726312	3.66E-06	1.31E-05
COPS2	-0.017681429	0.734274026	0.784522533
COPS3	0.150345725	0.00370071	0.007502345
COPS4	-0.170683544	0.000964246	0.002207652
COPS5	0.176497191	0.000637935	0.001516218
COPS6	0.078121188	0.133112911	0.184285991
COPS7A	-0.084307525	0.104956447	0.149952835
COPS7B	0.298741543	4.38E-09	2.56E-08
COPS8	0.188884402	0.000253318	0.000647581

COPZ1	0.283469558	2.76E-08	1.42E-07
COPZ2	-0.277313319	5.63E-08	2.73E-07
COQ10A	-0.21473333	3.03E-05	9.19E-05
COQ10B	-0.045252712	0.3847745	0.461025898
COQ2	0.116898074	0.024338513	0.041024779
COQ3	0.052749124	0.310918362	0.383726651
COQ4	-0.271743414	1.06E-07	4.88E-07
COQ5	-0.205953103	6.43E-05	0.000183952
COQ6	-0.298000949	4.80E-09	2.78E-08
COQ7	-0.133393148	0.010106386	0.018632188
COQ9	-0.254073193	7.09E-07	2.86E-06
CORIN	0.074463474	0.152311098	0.207122299
CORO1A	0.29662467	5.69E-09	3.27E-08
CORO1B	0.227805549	9.36E-06	3.11E-05
CORO1C	0.398094102	1.54E-15	2.45E-14
CORO2A	0.154004388	0.002938627	0.006092551
CORO2B	0.252342071	8.48E-07	3.37E-06
CORO6	0.409074465	2.12E-16	3.86E-15
CORO7	0.357137675	1.33E-12	1.34E-11
CORT	-0.125889492	0.015254481	0.026891405
COTL1	0.310528322	9.79E-10	6.34E-09
COX10	0.075441645	0.146982512	0.200766925
COX11	-0.020690568	0.691200687	0.748055331
COX15	-0.233864511	5.30E-06	1.85E-05
COX16	-0.059784831	0.250688367	0.318874578
COX17	0.0417992	0.422123006	0.498283731
COX18	-0.395407496	2.47E-15	3.81E-14
COX19	0.28199542	3.28E-08	1.66E-07
COX4I1	-0.027689259	0.594978818	0.662256659
COX4I2	-0.134045931	0.009741577	0.018034727
COX4NB	0.075563374	0.146329454	0.199970873
COX5A	0.144498976	0.005295215	0.010361582
COX5B	-0.029361964	0.572914427	0.64225993
COX6A1	0.136607996	0.008420195	0.015780538
COX6A2	-0.087142954	0.093734781	0.135740295
COX6B1	0.225349291	1.17E-05	3.83E-05
COX6B2	0.311035881	9.17E-10	5.96E-09
COX6C	0.130279866	0.012018097	0.021754
COX7A1	0.022038277	0.672217818	0.731919078
COX7A2L	0.061586092	0.236669466	0.303596895
COX7A2	0.158215304	0.002239887	0.004771495
COX7B2	0.009107777	0.861205503	0.891084243
COX7B	0.092680816	0.074590557	0.110952966
COX7C	0.188764124	0.000255673	0.000652932
COX8A	0.155502026	0.002670136	0.005598187
COX8C	-0.102432197	0.048665097	0.076260998
CP110	-0.007439191	0.886442308	0.910481731
CPA1	0.014618924	0.778982566	0.82236716
CPA2	0.221711484	1.63E-05	5.18E-05
CPA3	-0.043959812	0.398514798	0.47474806
CPA4	0.173591195	0.000785519	0.001832449
CPA5	-0.000248077	0.996200349	0.99695099
CPA6	0.238881258	3.27E-06	1.18E-05
CPAMD8	0.182347396	0.000415486	0.001019498
CPB1	-0.147239313	0.004483651	0.008924298
CPB2	-0.563421545	1.83E-32	4.40E-30
CPD	0.181336918	0.000447848	0.001093251
CPEB1	0.258618158	4.40E-07	1.84E-06

CPEB2	-0.261870679	3.11E-07	1.33E-06
CPEB3	-0.522336309	2.34E-27	2.22E-25
CPEB4	-0.207379054	5.70E-05	0.000164693
CPE	0.228351381	8.90E-06	2.97E-05
CPLX1	0.011180815	0.830049804	0.865047719
CPLX2	-0.043729675	0.400991081	0.477155915
CPLX3	0.243196635	2.14E-06	7.93E-06
CPLX4	-0.050220603	0.334714268	0.408640006
CPM	0.188896041	0.000253091	0.000647084
CPN1	-0.158055407	0.002263372	0.00481586
CPN2	-0.506871142	1.31E-25	9.33E-24
CPNE1	0.2422381	2.35E-06	8.68E-06
CPNE2	-0.013695993	0.79260794	0.834143444
CPNE3	-0.028288144	0.587034662	0.654919893
CPNE4	0.156200783	0.002552674	0.005380845
CPNE5	0.195502317	0.000150912	0.000403011
CPNE6	0.08389813	0.106660993	0.152082758
CPNE7	0.327129024	1.06E-10	7.95E-10
CPNE8	0.058422545	0.261667195	0.33121125
CPNE9	0.236396871	4.16E-06	1.47E-05
CPOX	-0.093868005	0.07093185	0.106208517
CPO	-0.109480796	0.035033301	0.056751782
CPPED1	-0.376539709	6.08E-14	7.59E-13
CPS1	-0.24124885	2.59E-06	9.51E-06
CPSF1	0.144545464	0.005280412	0.010335662
CPSF2	0.013069727	0.801889931	0.841420514
CPSF3L	0.032106853	0.537568689	0.60939134
CPSF3	0.249956041	1.08E-06	4.24E-06
CPSF4L	0.134816489	0.009326028	0.017335909
CPSF4	0.170347164	0.000987182	0.002256126
CPSF6	0.551087312	7.42E-31	1.23E-28
CPSF7	0.145839418	0.004883039	0.009647912
CPT1A	-0.135840386	0.008798257	0.016427261
CPT1B	0.162750281	0.001659684	0.003622683
CPT1C	0.073374004	0.158416959	0.21431364
CPT2	-0.434272353	1.70E-18	4.25E-17
CPVL	0.22747522	9.65E-06	3.20E-05
CPXCR1	0.114702307	0.0271633	0.045261564
CPXM1	0.344710724	8.64E-12	7.67E-11
CPXM2	0.049814473	0.338642543	0.412727073
CPZ	0.29692495	5.48E-09	3.15E-08
CP	-0.17493287	0.000713844	0.001679161
CR1L	0.2829296	2.94E-08	1.50E-07
CR1	0.063223454	0.224413361	0.290067993
CR2	0.333940923	4.09E-11	3.27E-10
CRABP1	0.190420291	0.000224971	0.000581156
CRABP2	0.331771483	5.55E-11	4.35E-10
CRADD	-0.446402363	1.43E-19	4.12E-18
CRAMP1L	0.123817802	0.01703091	0.029702337
CRAT	-0.503898458	2.77E-25	1.93E-23
CRB1	-0.088564965	0.088479395	0.128983353
CRB2	0.233596381	5.43E-06	1.89E-05
CRB3	0.066012599	0.204588803	0.267702835
CRBN	-0.32042605	2.64E-10	1.86E-09
CRCP	0.092860605	0.07402681	0.110254344
CRCT1	0.143228281	0.005714568	0.011099723
CREB1	0.221936093	1.60E-05	5.09E-05
CREB3L1	0.396440873	2.06E-15	3.20E-14

CREB3L2	-0.051240095	0.3249827	0.399032687
CREB3L3	-0.052816098	0.310303565	0.383110289
CREB3L4	0.056945825	0.273937127	0.344205326
CREB3	0.400762049	9.56E-16	1.57E-14
CREB5	0.074203553	0.153751347	0.208781564
CREBBP	-0.156040188	0.002579246	0.005432259
CREBL2	-0.331670786	5.63E-11	4.41E-10
CREBZF	0.149153928	0.003985138	0.008024249
CREG1	-0.060951695	0.241542507	0.308937457
CREG2	0.249080802	1.18E-06	4.60E-06
CRELD1	-0.13593686	0.008749926	0.016350585
CRELD2	0.129575822	0.01249231	0.022520297
CREM	-0.085525716	0.100011567	0.143764372
CRHBP	-0.224539609	1.26E-05	4.09E-05
CRHR1	0.191923122	0.000200128	0.000521921
CRHR2	-0.147337548	0.004456764	0.008876989
CRH	0.01524588	0.769764883	0.814578562
CRIM1	-0.149268941	0.003956852	0.007972935
CRIP1	0.350177678	3.83E-12	3.60E-11
CRIP2	-0.010015347	0.847536155	0.879590293
CRIP3	-0.030960417	0.55219785	0.623420921
CRIPAK	0.11019297	0.033857096	0.055070303
CRIPT	0.085961411	0.09828859	0.141532728
CRISP1	0.117089282	0.02410499	0.040672449
CRISP2	0.177196785	0.000606469	0.001445916
CRISP3	0.179854365	0.000499589	0.001209627
CRISPLD1	0.119841189	0.020952371	0.035832529
CRISPLD2	0.108133977	0.037352778	0.06008899
CRKL	-0.05490287	0.291548317	0.363218408
CRK	-0.21780434	2.32E-05	7.16E-05
CRLF1	0.206808093	5.98E-05	0.000172229
CRLF2	0.258644348	4.39E-07	1.83E-06
CRLF3	0.284411327	2.47E-08	1.28E-07
CRLS1	-0.409304457	2.03E-16	3.72E-15
CRMP1	0.401759354	8.00E-16	1.34E-14
CRNKL1	0.102631899	0.048225242	0.07566695
CRNN	0.038661436	0.457822873	0.532816174
CROCCL1	-0.259105819	4.18E-07	1.75E-06
CROCCL2	0.250861206	9.88E-07	3.89E-06
CROCC	0.300029663	3.73E-09	2.20E-08
CROT	-0.011862308	0.819863009	0.856717066
CRP	0.193407893	0.000178124	0.000469203
CRTAC1	-0.028270463	0.587268502	0.655070722
CRTAM	0.189041032	0.000250281	0.000640394
CRTAP	-0.114517344	0.027413626	0.045629063
CRTC1	0.131496587	0.011235939	0.020466525
CRTC2	0.113367973	0.029014028	0.04799215
CRTC3	0.219356509	2.02E-05	6.30E-05
CRX	-0.002934635	0.955075379	0.964956471
CRY1	0.120987621	0.019748534	0.033933958
CRY2	-0.447983072	1.03E-19	3.03E-18
CRYAA	-0.200825215	9.83E-05	0.000271899
CRYAB	0.139086411	0.007296372	0.013875365
CRYBA1	0.114136748	0.027934962	0.046399893
CRYBA2	0.293083013	8.77E-09	4.91E-08
CRYBA4	-0.062963449	0.226328795	0.291896308
CRYBB1	0.186237481	0.000310133	0.000780504
CRYBB2	0.163556852	0.001572238	0.003450713

CRYBB3	0.103828794	0.045658178	0.071979285
CRYBG3	-0.033151925	0.524404074	0.596470338
CRYGA	0.106516763	0.0403087	0.064407276
CRYGC	0.081249125	0.118224035	0.166296633
CRYGD	-0.054472686	0.295351215	0.367312997
CRYGN	0.144391588	0.005329553	0.010421609
CRYGS	0.180035493	0.000492983	0.001194793
CRYL1	-0.507523437	1.11E-25	8.05E-24
CRYM	-0.257070728	5.18E-07	2.14E-06
CRYZL1	0.067932664	0.19169928	0.252698542
CRYZ	-0.282763342	3.00E-08	1.53E-07
CSAD	-0.333779103	4.18E-11	3.34E-10
CSAG1	-0.020164303	0.698666906	0.75477697
CSAG2	0.103083643	0.047242514	0.074265397
CSAG3	0.124029249	0.016841669	0.029400606
CSDAP1	0.33778898	2.36E-11	1.96E-10
CSDA	0.36684838	2.92E-13	3.26E-12
CSDC2	0.150266151	0.003719112	0.00753735
CSDE1	-0.176142688	0.000654451	0.001552325
CSE1L	0.317190707	4.08E-10	2.79E-09
CSF1R	0.16080283	0.001889498	0.004087141
CSF1	0.223631096	1.37E-05	4.42E-05
CSF2RA	0.365477428	3.63E-13	4.00E-12
CSF2RB	0.213282641	3.44E-05	0.000103326
CSF2	0.18014816	0.000488914	0.001185366
CSF3R	0.31728559	4.03E-10	2.76E-09
CSF3	-0.031763164	0.541933928	0.613397404
CSGALNAC	0.086077852	0.097832139	0.140946765
CSGALNAC	0.094380887	0.069397231	0.104231879
CSH1	0.089127637	0.086466357	0.126428093
CSH2	0.148564102	0.00413309	0.008286979
CSK	0.088727916	0.087892583	0.128214892
CSMD1	-0.01987389	0.702799567	0.758295764
CSMD2	0.189066462	0.000249792	0.000639387
CSMD3	0.10056749	0.052936409	0.08207636
CSN1S1	-0.090457174	0.081855949	0.120464963
CSN1S2A	-0.010595251	0.838825532	0.872504686
CSN2	-0.244948044	1.80E-06	6.75E-06
CSN3	-0.155738122	0.002629909	0.005523196
CSNK1A1L	-0.061653126	0.236158625	0.303024097
CSNK1A1P	-0.128353042	0.013355235	0.023883572
CSNK1A1	0.038532867	0.459320782	0.534364913
CSNK1D	0.301049839	3.28E-09	1.96E-08
CSNK1E	0.360111661	8.41E-13	8.70E-12
CSNK1G1	0.182190866	0.000420352	0.00103017
CSNK1G2	0.201300075	9.45E-05	0.000262162
CSNK1G3	-0.00067362	0.989682805	0.99202353
CSNK2A1P	0.087448805	0.09258388	0.134278978
CSNK2A1	0.271277884	1.11E-07	5.13E-07
CSNK2A2	-0.098022186	0.059266112	0.090620883
CSNK2B	0.15041843	0.003683968	0.007472207
CSPG4PY2	0.109382257	0.035198748	0.056996623
CSPG4	0.167586191	0.001195345	0.00268929
CSPG5	0.194827262	0.000159225	0.000423283
CSPP1	0.253522418	7.51E-07	3.01E-06
CSRNP1	-0.149130998	0.003990799	0.008033212
CSRNP2	0.425589019	9.39E-18	2.09E-16
CSRNP3	0.132270279	0.010762316	0.019706513

CSRP1	0.092009888	0.076725232	0.113788437
CSRP2BP	-0.056847489	0.274767876	0.34514033
CSRP2	-0.05830563	0.262624611	0.332190952
CSRP3	0.085323584	0.100819011	0.144820559
CST11	-0.062189811	0.232096742	0.298504279
CST1	0.082731233	0.111639517	0.158263891
CST2	0.284786987	2.37E-08	1.23E-07
CST3	0.012583044	0.809122489	0.847672393
CST4	0.080311424	0.122544141	0.171597974
CST5	0.225652525	1.14E-05	3.73E-05
CST6	0.265203128	2.17E-07	9.58E-07
CST7	0.058802663	0.258571014	0.327875485
CST8	0.152248582	0.003284504	0.006733268
CST9L	0.171389125	0.000917723	0.002108265
CST9	0.138810712	0.007414388	0.014069078
CSTA	0.07752288	0.136118825	0.187840386
CSTB	0.298235306	4.66E-09	2.71E-08
CSTF1	-0.054805124	0.292409515	0.364131914
CSTF2T	-0.07891537	0.129202281	0.179583329
CSTF2	0.263378115	2.65E-07	1.15E-06
CSTF3	0.323195455	1.82E-10	1.31E-09
CSTL1	-0.058235405	0.26320084	0.332666359
CSTT	0.089858152	0.083907973	0.123181283
CS	0.261919237	3.10E-07	1.33E-06
CT45A1	0.244361314	1.90E-06	7.13E-06
CT45A2	0.184176055	0.000362384	0.000900177
CT45A3	0.230230085	7.47E-06	2.53E-05
CT45A4	0.11687153	0.024371086	0.041076208
CT45A5	0.214225869	3.17E-05	9.58E-05
CT45A6	0.164155939	0.001510044	0.003328512
CT47A10	0.026105669	0.616216773	0.681598498
CT47A11	0.090859458	0.080500773	0.118724933
CT47A1	0.016746975	0.747828815	0.796305062
CT47A2	0.05818732	0.263595903	0.33308116
CT47A6	-0.003602482	0.944866424	0.957409536
CT47A7	-0.007970447	0.878393529	0.904284973
CT47A9	-0.077755793	0.13494252	0.186443226
CT47B1	0.081494852	0.117111847	0.164976822
CT62	0.184994159	0.000340737	0.000850539
CTAG1B	0.118851365	0.022042487	0.037513278
CTAG2	0.102021946	0.049579226	0.077468027
CTAGE1	-0.26468912	2.30E-07	1.01E-06
CTAGE4	0.268323959	1.54E-07	6.96E-07
CTAGE5	-0.402346424	7.20E-16	1.21E-14
CTAGE6	0.216207016	2.67E-05	8.15E-05
CTAGE9	0.260591574	3.57E-07	1.51E-06
CTBP1	0.210959614	4.21E-05	0.000124589
CTBP2	0.227263736	9.84E-06	3.25E-05
CTBS	-0.348713217	4.77E-12	4.41E-11
CTCFL	0.056870921	0.274569763	0.344913224
CTCF	-0.024422443	0.639146824	0.703174454
CTDP1	0.140454103	0.006735379	0.012900897
CTDSP1	-0.324670199	1.49E-10	1.09E-09
CTDSP2	0.019929293	0.702010484	0.75773164
CTDSPL2	0.164035882	0.001522324	0.003352286
CTDSPL	0.267640816	1.66E-07	7.47E-07
CTF1	0.09023311	0.082618709	0.121470842
CTGF	0.052422122	0.313931596	0.386822442

CTHRC1	0.421478768	2.07E-17	4.42E-16
CTH	-0.447905758	1.05E-19	3.08E-18
CTLA4	0.314444555	5.87E-10	3.92E-09
CTNNA1	0.308870295	1.21E-09	7.73E-09
CTNNA2	-0.208925109	5.00E-05	0.000146063
CTNNA3	-0.260692577	3.53E-07	1.50E-06
CTNNAL1	0.021338221	0.682053262	0.740281399
CTNNB1	-0.15531072	0.002703142	0.0056543
CTNNBIP1	-0.168971471	0.00108632	0.00246348
CTNNBL1	-0.066873746	0.198732276	0.260985129
CTNND1	-0.062993942	0.226103555	0.291700235
CTNND2	0.443347987	2.70E-19	7.50E-18
CTNS	-0.090488189	0.081750816	0.120336911
CTPS2	0.19768684	0.000126714	0.000343208
CTPS	-0.087765474	0.091404207	0.132790389
CTR9	-0.254855734	6.54E-07	2.65E-06
CTRB1	0.045057121	0.386834422	0.463131932
CTRB2	0.156004714	0.002585149	0.00544239
CTRC	0.102383494	0.048772875	0.076405851
CTRL	0.280823803	3.76E-08	1.89E-07
CTSA	0.219916273	1.92E-05	6.01E-05
CTSB	0.008636384	0.868322021	0.896445629
CTSC	0.324831604	1.45E-10	1.07E-09
CTSD	0.098701435	0.057518651	0.088301346
CTSE	0.322872646	1.90E-10	1.37E-09
CTSF	-0.458180424	1.18E-20	3.96E-19
CTSG	-0.182918921	0.000398158	0.000980649
CTSH	0.037578769	0.470521618	0.545301436
CTSK	0.345505954	7.68E-12	6.89E-11
CTSL1	0.140317905	0.006789459	0.012995206
CTSL2	0.503264742	3.24E-25	2.23E-23
CTSO	-0.467474113	1.54E-21	5.83E-20
CTSS	0.283255325	2.83E-08	1.45E-07
CTSW	0.050184949	0.335057954	0.409008858
CTSZ	0.021021681	0.686518345	0.744114172
CTTNBP2NI	0.2832941	2.82E-08	1.45E-07
CTTNBP2	-0.015503979	0.76597955	0.811478043
CTTN	0.218064768	2.26E-05	7.00E-05
CTU1	0.100267439	0.053652025	0.083063074
CTU2	-0.132450433	0.010654604	0.019521844
CTXN1	0.270770244	1.18E-07	5.41E-07
CTXN2	0.048503976	0.351518274	0.426203339
CTXN3	0.087278232	0.093224334	0.135070196
CUBN	0.210323569	4.44E-05	0.000131095
CUEDC1	0.263442121	2.63E-07	1.14E-06
CUEDC2	-0.099844103	0.054675335	0.084457007
CUL1	-0.155956764	0.002593148	0.0054575
CUL2	0.078330824	0.132071887	0.18298464
CUL3	-0.012760117	0.806489101	0.845402571
CUL4A	0.0313437	0.547285363	0.618505729
CUL4B	0.098612371	0.057745321	0.088587886
CUL5	-0.192961622	0.000184487	0.000484747
CUL7	0.097277122	0.061233122	0.093256346
CUL9	-0.053979109	0.299755094	0.372057194
CUTA	-0.029542212	0.570560073	0.640059762
CUTC	-0.064412275	0.21580243	0.280444619
CUX1	-0.222505276	1.52E-05	4.86E-05
CUX2	-0.405289553	4.23E-16	7.34E-15

CUZD1	0.27226109	9.96E-08	4.63E-07
CWC15	0.087628588	0.091912656	0.133441477
CWC22	0.147548544	0.004399505	0.008776102
CWC25	-0.031767747	0.541875612	0.613366247
CWC27	0.413176272	9.94E-17	1.92E-15
CWF19L1	0.087904496	0.090890131	0.132091712
CWF19L2	-0.194148722	0.000168012	0.000444625
CWH43	-0.214329201	3.14E-05	9.50E-05
CX3CL1	-0.11488439	0.026918791	0.04489921
CX3CR1	-0.015849152	0.760925862	0.807154306
CXADRP2	-0.136619925	0.008414435	0.015772712
CXADRP3	-0.019695947	0.705336079	0.76025025
CXADR	-0.1553891	0.002689574	0.005630696
CXCL10	0.000987341	0.984878314	0.988450668
CXCL11	-0.009270407	0.858752875	0.889100654
CXCL12	0.044271714	0.395173416	0.471528798
CXCL13	0.146213655	0.004773245	0.009451604
CXCL14	0.190048261	0.000231552	0.000596685
CXCL16	0.226605613	1.05E-05	3.44E-05
CXCL17	0.257393458	5.01E-07	2.07E-06
CXCL1	0.319590394	2.96E-10	2.07E-09
CXCL2	-0.019952675	0.701677557	0.75749541
CXCL3	0.298123794	4.72E-09	2.74E-08
CXCL5	0.369700821	1.85E-13	2.13E-12
CXCL6	0.252860673	8.04E-07	3.21E-06
CXCL9	0.038661978	0.457816571	0.532816174
CXCR1	-0.017707149	0.733902088	0.784251322
CXCR2P1	0.058537697	0.260726564	0.330209447
CXCR2	0.078894827	0.129302305	0.179697281
CXCR3	0.270189479	1.26E-07	5.74E-07
CXCR4	0.297855973	4.88E-09	2.83E-08
CXCR5	0.201363115	9.40E-05	0.000260936
CXCR6	0.192128579	0.000196939	0.000514337
CXCR7	0.04228528	0.4167418	0.49269065
CXXC1	0.168966683	0.001086681	0.002464017
CXXC4	0.113910789	0.028248488	0.046862052
CXXC5	0.246578146	1.53E-06	5.81E-06
CXorf1	0.017192269	0.74135959	0.79077827
CXorf21	0.284244495	2.52E-08	1.30E-07
CXorf22	-0.077409727	0.136693134	0.188495924
CXorf23	0.138840486	0.007401561	0.014048581
CXorf26	0.17174102	0.000895303	0.002062946
CXorf27	0.019880371	0.702707241	0.758278283
CXorf30	-0.031115975	0.55020149	0.62137835
CXorf36	-0.145160275	0.005088112	0.010005465
CXorf38	0.090090302	0.083107846	0.122126909
CXorf40A	-0.057779243	0.266965028	0.336697727
CXorf40B	0.094680284	0.068514012	0.10316198
CXorf41	-0.062494858	0.229810142	0.295945549
CXorf42	-0.024396828	0.639498506	0.703405987
CXorf48	0.164293114	0.001496124	0.003301117
CXorf49B	0.090929775	0.080265769	0.118457268
CXorf50B	0.179411474	0.000516091	0.001246401
CXorf51	0.046816534	0.368545976	0.444360765
CXorf56	-0.048681798	0.349753278	0.424450829
CXorf57	0.058325847	0.262458884	0.332044573
CXorf58	-0.082102812	0.114395311	0.161595645
CXorf59	0.003796711	0.941898986	0.95522865

CXorf61	0.219985955	1.91E-05	5.98E-05
CXorf64	0.05530364	0.288035069	0.359538511
CXorf65	0.28255205	3.07E-08	1.57E-07
CXorf66	-0.458340148	1.14E-20	3.83E-19
CYB561D1	0.292741458	9.14E-09	5.09E-08
CYB561D2	0.134560826	0.009462123	0.017559796
CYB561	0.187393256	0.000284001	0.000720106
CYB5A	-0.5022256	4.21E-25	2.81E-23
CYB5B	-0.15173878	0.003391631	0.006935039
CYB5D1	-0.294512615	7.37E-09	4.17E-08
CYB5D2	-0.44609434	1.53E-19	4.39E-18
CYB5R1	-0.087956057	0.090700057	0.13183471
CYB5R2	0.210028202	4.56E-05	0.000134068
CYB5R3	-0.04028958	0.439091895	0.51477601
CYB5R4	0.164096933	0.001516068	0.003340312
CYB5RL	0.057688773	0.267715939	0.337473863
CYBASC3	-0.153523191	0.003029966	0.006264969
CYBA	0.37373993	9.62E-14	1.16E-12
CYBB	0.230072997	7.58E-06	2.56E-05
CYBRD1	-0.028454058	0.584842528	0.653096011
CYC1	0.182480393	0.000411392	0.001010449
CYCSP52	-0.007163554	0.890622993	0.913833501
CYCS	0.307105294	1.52E-09	9.55E-09
CYFIP1	0.121999305	0.018736617	0.03236827
CYFIP2	0.090923244	0.080287572	0.118471891
CYGB	-0.167248001	0.001223448	0.002746004
CYHR1	0.054979603	0.290873445	0.362559017
CYLC1	0.063223078	0.224416122	0.290067993
CYLC2	0.125286356	0.015754082	0.027685215
CYLD	-0.162150441	0.001727582	0.003759765
CYMP	0.143177729	0.005731852	0.011128579
CYP11A1	-0.207423535	5.68E-05	0.000164171
CYP11B1	0.008675965	0.86772405	0.896060467
CYP11B2	-0.076740026	0.140130392	0.192660229
CYP17A1	-0.188157127	0.000267874	0.00068208
CYP19A1	0.418598315	3.59E-17	7.40E-16
CYP1A1	-0.452087457	4.35E-20	1.33E-18
CYP1A2	-0.445514051	1.72E-19	4.90E-18
CYP1B1	0.035520184	0.495192408	0.569092769
CYP20A1	-0.112904551	0.029681602	0.048974398
CYP21A2	0.135034823	0.009211184	0.017138807
CYP24A1	0.223210334	1.43E-05	4.58E-05
CYP26A1	-0.190076238	0.000231051	0.000595471
CYP26B1	0.175637495	0.000678673	0.001603857
CYP26C1	0.067025179	0.197715151	0.25972032
CYP27A1	-0.446640063	1.36E-19	3.94E-18
CYP27B1	0.239649149	3.03E-06	1.10E-05
CYP27C1	0.015230138	0.769995939	0.814779789
CYP2A13	-0.238766782	3.31E-06	1.19E-05
CYP2A6	-0.347113805	6.05E-12	5.51E-11
CYP2A7	-0.269307457	1.38E-07	6.29E-07
CYP2B6	-0.407642979	2.76E-16	4.95E-15
CYP2B7P1	-0.162840971	0.001649634	0.003603114
CYP2C18	-0.157624991	0.002327711	0.004943779
CYP2C19	-0.283734595	2.68E-08	1.38E-07
CYP2C8	-0.418153558	3.90E-17	7.98E-16
CYP2C9	-0.395049713	2.63E-15	4.04E-14
CYP2D6	-0.172647512	0.000839857	0.001947576

CYP2D7P1	-0.175457844	0.000687484	0.001622756
CYP2E1	-0.386313809	1.19E-14	1.63E-13
CYP2F1	0.031510932	0.545148728	0.61644074
CYP2J2	-0.474967775	2.83E-22	1.21E-20
CYP2R1	0.0775055	0.136206919	0.18794253
CYP2S1	0.404532062	4.86E-16	8.37E-15
CYP2U1	-0.388939315	7.58E-15	1.08E-13
CYP2W1	0.213126859	3.49E-05	0.000104667
CYP39A1	-0.409027537	2.14E-16	3.89E-15
CYP3A43	-0.366758864	2.96E-13	3.30E-12
CYP3A4	-0.454872955	2.40E-20	7.67E-19
CYP3A5	-0.221351074	1.69E-05	5.34E-05
CYP3A7	0.110402118	0.03351813	0.054581345
CYP46A1	-0.20922451	4.88E-05	0.000142642
CYP4A11	-0.405375021	4.17E-16	7.25E-15
CYP4A22	-0.389690498	6.66E-15	9.64E-14
CYP4B1	-0.017682778	0.734254515	0.784522533
CYP4F11	-0.324715299	1.48E-10	1.08E-09
CYP4F12	-0.309560662	1.11E-09	7.12E-09
CYP4F22	0.092863991	0.074016227	0.110246825
CYP4F2	-0.430896827	3.32E-18	7.91E-17
CYP4F3	-0.375839021	6.83E-14	8.43E-13
CYP4F8	0.111654189	0.031548774	0.05170407
CYP4V2	-0.388938868	7.58E-15	1.08E-13
CYP4X1	-0.128054112	0.013574011	0.02425087
CYP4Z1	-0.126214789	0.014990835	0.026473488
CYP4Z2P	-0.038096943	0.464419852	0.53929659
CYP51A1	-0.214108434	3.20E-05	9.66E-05
CYP7A1	-0.261508512	3.24E-07	1.38E-06
CYP7B1	-0.289003928	1.43E-08	7.73E-08
CYP8B1	-0.519589544	4.85E-27	4.37E-25
CYR61	-0.131306334	0.011355192	0.020664852
CYS1	0.241734914	2.47E-06	9.10E-06
CYSLTR1	-0.013363838	0.797527312	0.838257841
CYSLTR2	0.147562552	0.004395727	0.008769444
CYTH1	-0.154401633	0.002865117	0.005956884
CYTH2	0.307698522	1.41E-09	8.91E-09
CYTH3	0.065743535	0.206444047	0.269725754
CYTH4	0.281095566	3.64E-08	1.83E-07
CYTIP	0.23486737	4.81E-06	1.69E-05
CYTL1	0.058651375	0.259800262	0.329119981
CYTSA	-0.263885357	2.50E-07	1.09E-06
CYTSB	0.324966883	1.43E-10	1.05E-09
CYYR1	-0.209745123	4.67E-05	0.00013688
CYorf15A	-0.105630174	0.042011416	0.066790475
CYorf15B	-0.061767456	0.235289148	0.302044617
D2HGDH	-0.329935254	7.18E-11	5.55E-10
D4S234E	0.130557415	0.011835564	0.021452834
DAAM1	-0.295254719	6.73E-09	3.83E-08
DAAM2	-0.287905786	1.64E-08	8.71E-08
DAB1	-0.198214246	0.000121444	0.000330203
DAB2IP	0.124433084	0.016485344	0.028836687
DAB2	0.349635305	4.16E-12	3.89E-11
DACH1	0.063527615	0.222187312	0.287616351
DACH2	0.051060158	0.326686829	0.400754619
DACT1	0.174210499	0.000751646	0.001759327
DACT2	0.031905135	0.5401286	0.611805888
DACT3	0.224457594	1.27E-05	4.12E-05

DAD1L	0.14240299	0.006002671	0.011613597
DAD1	0.135100184	0.00917705	0.01708008
DAG1	0.017063503	0.743228462	0.792220301
DAGLA	0.463190094	3.96E-21	1.43E-19
DAGLB	-0.021209912	0.683861861	0.741880636
DAK	-0.395447893	2.45E-15	3.78E-14
DALRD3	0.146442403	0.004707235	0.009333885
DAND5	0.429608289	4.28E-18	1.00E-16
DAO	-0.40867396	2.28E-16	4.14E-15
DAP3	0.116831577	0.024420184	0.041148515
DAPK1	0.076186212	0.143022565	0.196083927
DAPK2	-0.155568864	0.002658691	0.005577764
DAPK3	0.155906239	0.002601601	0.005471822
DAPL1	0.152345145	0.003264562	0.00669928
DAPP1	0.212422589	3.71E-05	0.00011094
DAP	0.050858815	0.32860053	0.402680677
DARC	0.011001823	0.832730131	0.86723274
DARS2	0.217580165	2.36E-05	7.28E-05
DARS	0.146315304	0.00474381	0.009398924
DAXX	0.130570361	0.01182711	0.021443495
DAZ1	0.014271286	0.784107109	0.826944511
DAZ2	0.019301219	0.710974431	0.765085756
DAZ3	-0.006496892	0.9007468	0.9219738
DAZAP1	0.432832474	2.26E-18	5.53E-17
DAZAP2	0.038839068	0.455757848	0.531002272
DAZL	0.042586354	0.413429024	0.489587649
DBC1	0.179559509	0.00051052	0.001233994
DBF4B	0.418107288	3.94E-17	8.04E-16
DBF4	0.49267261	4.41E-24	2.45E-22
DBH	-0.164084898	0.001517299	0.003342655
DBI	-0.022653146	0.663624984	0.724741636
DBN1	0.445611924	1.69E-19	4.82E-18
DBNDD1	-0.166495305	0.00128819	0.0028777
DBNDD2	0.495485233	2.22E-24	1.29E-22
DBNL	0.185306128	0.000332804	0.000832825
DBP	-0.199501829	0.000109431	0.000300163
DBR1	0.28113476	3.62E-08	1.83E-07
DBT	-0.459193316	9.48E-21	3.20E-19
DBX1	0.050832896	0.328847405	0.402908856
DBX2	0.063000579	0.226054556	0.291655907
DCAF10	-0.171881462	0.000886497	0.002045257
DCAF11	-0.434361928	1.67E-18	4.18E-17
DCAF12L1	0.126111139	0.015074404	0.026609275
DCAF12L2	-0.045709676	0.379987965	0.45611377
DCAF12	0.124846407	0.016127495	0.028262839
DCAF13	0.308411173	1.29E-09	8.17E-09
DCAF15	0.2998277	3.82E-09	2.25E-08
DCAF16	0.356452402	1.48E-12	1.47E-11
DCAF17	0.027100819	0.602831681	0.669432149
DCAF4L1	0.166303516	0.001305183	0.002911741
DCAF4L2	-0.033677097	0.517851226	0.59043279
DCAF4	0.127145025	0.014258836	0.025315437
DCAF5	-0.180308746	0.000483169	0.001172436
DCAF6	-0.24929492	1.16E-06	4.51E-06
DCAF7	0.202407018	8.63E-05	0.000241203
DCAF8L1	0.018475968	0.722813102	0.775187479
DCAF8L2	0.038483663	0.459894761	0.534790068
DCAF8	-0.352108906	2.87E-12	2.74E-11

DCAKD	-0.248221247	1.29E-06	4.99E-06
DCBLD1	-0.099667044	0.055108113	0.085052977
DCBLD2	-0.036234104	0.486559405	0.560659174
DCC	-0.037744573	0.468564413	0.543444154
DCDC1	0.13282633	0.010432928	0.019159733
DCDC2B	-0.021775967	0.675896719	0.734937312
DCDC2	0.189302858	0.000245281	0.000628811
DCHS1	0.088551193	0.088529135	0.129036979
DCHS2	0.217560415	2.37E-05	7.29E-05
DCI	-0.301587541	3.07E-09	1.84E-08
DCK	0.268530735	1.51E-07	6.82E-07
DCLK1	0.237338469	3.80E-06	1.35E-05
DCLK2	0.196312622	0.000141471	0.000379682
DCLK3	0.124582958	0.016354791	0.028623398
DCLRE1A	0.01993035	0.701995426	0.75773164
DCLRE1B	0.344172308	9.35E-12	8.26E-11
DCLRE1C	0.459339484	9.19E-21	3.11E-19
DCN	0.073297996	0.158849735	0.214797015
DCP1A	0.099115382	0.056474792	0.086927572
DCP1B	-0.074744056	0.150767867	0.205289963
DCP2	0.367242461	2.74E-13	3.08E-12
DCPS	0.133322257	0.010146724	0.018696174
DCST1	0.042681507	0.412385273	0.488538325
DCST2	-0.05744884	0.269714387	0.339627712
DCTD	0.017330811	0.739350541	0.788973243
DCTN1	0.128731287	0.013082863	0.023456199
DCTN2	0.327527785	1.00E-10	7.55E-10
DCTN3	-0.049714719	0.339611893	0.413807226
DCTN4	0.027238763	0.600986629	0.667718232
DCTN5	0.349088845	4.51E-12	4.19E-11
DCTN6	0.09138409	0.078760771	0.116469127
DCTPP1	0.347663249	5.58E-12	5.09E-11
DCT	-0.082711528	0.111725126	0.158373983
DCUN1D1	-0.133228423	0.010200336	0.018789746
DCUN1D2	0.216229532	2.66E-05	8.13E-05
DCUN1D3	-0.135551347	0.008944482	0.01668308
DCUN1D4	-0.183038987	0.000394605	0.000972574
DCUN1D5	0.336644954	2.78E-11	2.28E-10
DCXR	-0.552436868	4.99E-31	8.35E-29
DCX	0.107479182	0.038526692	0.061777911
DDA1	0.294380246	7.49E-09	4.23E-08
DDAH1	-0.119849176	0.020943767	0.035827042
DDAH2	0.15170269	0.003399334	0.006950075
DDB1	0.023542494	0.65127409	0.714032383
DDB2	-0.095749975	0.065433312	0.098957143
DDC	-0.066037473	0.204417907	0.267517147
DDHD1	0.313248492	6.86E-10	4.53E-09
DDHD2	0.114577144	0.027332477	0.045520616
DDI1	0.156421569	0.00251655	0.005310317
DDI2	-0.304334822	2.17E-09	1.33E-08
DDIT3	0.261916151	3.10E-07	1.33E-06
DDIT4L	0.109257792	0.035408675	0.05730395
DDIT4	0.155230251	0.002717136	0.005679522
DDN	0.341106304	1.46E-11	1.25E-10
DDOST	0.223935496	1.34E-05	4.31E-05
DDO	0.027788072	0.593664709	0.661205811
DDR1	0.439285707	6.19E-19	1.65E-17
DDR2	0.091715293	0.077678079	0.115038857

DDRGK1	-0.060886145	0.242050002	0.309487204
DDTL	-0.260176466	3.73E-07	1.57E-06
DDT	-0.249474135	1.14E-06	4.44E-06
DDX10	0.112265006	0.030624464	0.05035495
DDX11L2	-0.079433666	0.126698463	0.176608366
DDX11	0.402597612	6.88E-16	1.17E-14
DDX12	0.439550131	5.86E-19	1.57E-17
DDX17	0.107707241	0.038114333	0.061195499
DDX18	0.038565046	0.458945624	0.534029246
DDX19A	-0.085033784	0.101985672	0.146288642
DDX19B	-0.173700683	0.00077943	0.001819309
DDX1	0.137508655	0.007995105	0.015074625
DDX20	0.167782757	0.001179285	0.002657059
DDX21	0.0280703	0.589918678	0.657585044
DDX23	0.305927125	1.77E-09	1.10E-08
DDX24	-0.168198477	0.001145971	0.002587853
DDX25	-0.065499191	0.208139357	0.271691277
DDX26B	0.286012044	2.05E-08	1.07E-07
DDX27	0.377856884	4.90E-14	6.18E-13
DDX28	-0.232350446	6.12E-06	2.10E-05
DDX31	0.178087857	0.000568473	0.001362829
DDX39	0.574776291	5.29E-34	1.88E-31
DDX3X	0.070286149	0.176724387	0.235577333
DDX3Y	-0.07000678	0.178455153	0.237518944
DDX41	0.179560115	0.000510497	0.001233994
DDX42	0.139114987	0.007284236	0.013854931
DDX43	0.110152317	0.033923321	0.055173319
DDX46	0.341333854	1.42E-11	1.22E-10
DDX47	0.152009858	0.00333428	0.006828283
DDX49	0.266262542	1.93E-07	8.59E-07
DDX4	0.072691758	0.162333462	0.218913371
DDX50	0.160915549	0.00187544	0.004058497
DDX51	0.11714507	0.024037221	0.040574748
DDX52	0.313692376	6.48E-10	4.30E-09
DDX53	-0.035543792	0.494905637	0.568796013
DDX54	0.263401191	2.64E-07	1.15E-06
DDX55	0.402291928	7.27E-16	1.22E-14
DDX56	0.091279869	0.079103985	0.116924588
DDX58	-0.018062123	0.728775115	0.780195488
DDX59	-0.036609982	0.482046904	0.556242867
DDX5	0.033889662	0.515210985	0.587859865
DDX60L	-0.150119649	0.003753208	0.007601811
DDX60	-0.140401439	0.006756245	0.012938374
DDX6	-0.021223893	0.683664702	0.741707139
DEAF1	0.284258075	2.52E-08	1.30E-07
1-Dec	-0.009098943	0.861338761	0.891109824
DECR1	-0.252577846	8.28E-07	3.30E-06
DECR2	-0.36813905	2.37E-13	2.71E-12
DEDD2	0.071797891	0.167574349	0.22497582
DEDD	0.150081823	0.003762058	0.007615864
DEF6	0.335850036	3.12E-11	2.53E-10
DEF8	-0.04330259	0.405610831	0.481818555
DEFA1B	-0.037817177	0.467708785	0.54257814
DEFA4	0.035186381	0.49925667	0.573108828
DEFA5	0.074860034	0.150133454	0.204593931
DEFA6	0.081022346	0.119257765	0.16754959
DEFB103B	0.05565816	0.284951057	0.356068177
DEFB109P1	0.070227663	0.177085692	0.235969854

DEFB118	-8.41E-05	0.998712218	0.999013096
DEFB123	0.040378054	0.43808677	0.513930316
DEFB124	0.036151018	0.487559928	0.561649641
DEFB125	-0.074700004	0.151009365	0.20554855
DEFB126	0.133533794	0.010026778	0.018497404
DEFB131	0.13204751	0.01089684	0.019914398
DEFB132	-0.329749463	7.36E-11	5.69E-10
DEFB1	0.075924099	0.144407196	0.197655799
DEFB4A	0.013021992	0.802598572	0.841988876
DEGS1	0.432218988	2.56E-18	6.19E-17
DEGS2	0.173129326	0.000811694	0.001887983
DEK	0.166971948	0.001246836	0.002794191
DEM1	0.196405529	0.000140424	0.000377127
DENND1A	-0.138412908	0.007587678	0.014371202
DENND1B	0.059242799	0.255017678	0.323876418
DENND1C	0.110080583	0.034040446	0.055336905
DENND2A	0.118474111	0.022470716	0.038206163
DENND2C	0.009385083	0.857024265	0.887633866
DENND2D	0.276405306	6.24E-08	3.01E-07
DENND3	0.336884396	2.69E-11	2.21E-10
DENND4A	-0.205855332	6.48E-05	0.000185301
DENND4B	0.3441999	9.31E-12	8.23E-11
DENND4C	-0.162686959	0.001666734	0.003636478
DENND5A	0.159588872	0.002047117	0.004398002
DENND5B	-0.280276232	4.00E-08	2.00E-07
DENR	0.444563471	2.10E-19	5.91E-18
DEPDC1B	0.636772045	1.40E-43	6.98E-40
DEPDC1	0.474548892	3.12E-22	1.31E-20
DEPDC4	0.058256523	0.263027471	0.332531621
DEPDC5	-0.32346245	1.75E-10	1.27E-09
DEPDC6	-0.047473554	0.361856234	0.43751289
DEPDC7	-0.470409617	7.96E-22	3.17E-20
DERA	-0.246964247	1.47E-06	5.60E-06
DERL1	-0.014144483	0.785978647	0.828698947
DERL2	0.028654624	0.582197601	0.650799047
DERL3	0.105133402	0.042991704	0.068158581
DES	0.217623919	2.35E-05	7.25E-05
DET1	-0.229554503	7.95E-06	2.68E-05
DEXI	-0.374692939	8.24E-14	1.00E-12
DFFA	0.035263565	0.498315342	0.572219624
DFFB	0.243412922	2.09E-06	7.78E-06
DFNA5	0.230499974	7.28E-06	2.47E-05
DFNB31	0.130096466	0.012140071	0.02194886
DFNB59	-0.14109792	0.006484923	0.012471535
DGAT1	0.106168336	0.04097078	0.06536557
DGAT2L6	0.017877186	0.73144472	0.782506804
DGAT2	-0.128327077	0.013374114	0.023913041
DGCR10	-0.01557069	0.765002044	0.8106149
DGCR11	0.15288638	0.003154807	0.006502179
DGCR14	0.182249455	0.000418524	0.00102607
DGCR2	0.004666576	0.928619571	0.944405487
DGCR5	-0.080563139	0.121372574	0.170184715
DGCR6L	-0.269407189	1.37E-07	6.23E-07
DGCR6	-0.176557769	0.000635152	0.001509963
DGCR8	0.183561146	0.000379491	0.000938459
DGCR9	0.002125422	0.967455002	0.974428422
DGKA	0.279204057	4.53E-08	2.24E-07
DGKB	-0.092418564	0.07541913	0.112059958

DGKD	0.230500078	7.28E-06	2.47E-05
DGKE	0.064854188	0.212662757	0.276833994
DGKG	0.329112196	8.05E-11	6.17E-10
DGKH	0.027267786	0.600598747	0.667491074
DGKI	0.034740543	0.504712441	0.57803284
DGKK	0.099184454	0.05630215	0.086680945
DGKQ	0.121535641	0.01919464	0.033073484
DGKZ	0.468462312	1.23E-21	4.75E-20
DGUOK	0.241499137	2.53E-06	9.30E-06
DHCR24	-0.206143122	6.33E-05	0.000181356
DHCR7	-0.127663672	0.013864506	0.024694564
DHDDS	-0.087044876	0.094106255	0.136219197
DHDH	0.389900523	6.42E-15	9.31E-14
DHDPSL	-0.354510523	1.99E-12	1.95E-11
DHFRL1	-0.275906155	6.61E-08	3.17E-07
DHFR	0.151245033	0.003498395	0.007130655
DHH	-0.003745787	0.942676916	0.955785992
DHODH	-0.34639311	6.74E-12	6.10E-11
DHPS	-0.104820627	0.043618732	0.069048263
DHRS11	-0.079875691	0.124592966	0.174087604
DHRS12	-0.478196955	1.35E-22	6.03E-21
DHRS13	0.275336907	7.05E-08	3.36E-07
DHRS1	-0.328273961	9.04E-11	6.87E-10
DHRS2	-0.159597685	0.002045931	0.004396401
DHRS3	-0.364535442	4.21E-13	4.58E-12
DHRS4L1	-0.462869441	4.25E-21	1.53E-19
DHRS4L2	-0.341094607	1.47E-11	1.25E-10
DHRS4	-0.389012377	7.48E-15	1.07E-13
DHRS7B	0.042960792	0.409330758	0.485571476
DHRS7C	0.046958384	0.367095215	0.442906424
DHRS7	0.113974141	0.028160281	0.046731289
DHRS9	-0.015146863	0.771218539	0.815726878
DHRSX	-0.075220739	0.14817331	0.202213227
DHTKD1	-0.45008721	6.63E-20	2.00E-18
DHX15	0.289557011	1.34E-08	7.28E-08
DHX16	-0.053405592	0.304926745	0.377571671
DHX29	-0.000601358	0.990789515	0.992832791
DHX30	0.122074857	0.018662894	0.032260493
DHX32	0.018626937	0.720642328	0.773483996
DHX33	-0.052681321	0.31154158	0.384376749
DHX34	0.401641291	8.17E-16	1.36E-14
DHX35	0.157918267	0.002283693	0.004856505
DHX36	-0.160609221	0.001913868	0.004133572
DHX37	0.332738085	4.84E-11	3.83E-10
DHX38	-0.0619636	0.233802746	0.300388127
DHX40P1	-0.014910344	0.774694096	0.818664108
DHX40	-0.09324796	0.072824005	0.108714862
DHX57	0.192436896	0.000192241	0.000503131
DHX58	-0.30540959	1.89E-09	1.17E-08
DHX8	0.046826609	0.36844282	0.444308176
DHX9	0.282063939	3.25E-08	1.65E-07
DIABLO	0.13226314	0.010766604	0.019712494
DIAPH1	-0.184005558	0.000367054	0.000910642
DIAPH2	-0.049920233	0.337616752	0.411653157
DIAPH3	0.397799674	1.62E-15	2.57E-14
DICER1	0.013300375	0.798468141	0.838792872
DIDO1	0.143890468	0.005492451	0.010707565
DIMT1L	0.15618575	0.00255515	0.005385496

DIO1	-0.167736785	0.001183023	0.002664556
DIO2	0.057514501	0.269166479	0.339087808
DIO3OS	0.029372926	0.572771113	0.642135403
DIO3	0.179444701	0.000514836	0.001243822
DIP2A	0.273362518	8.81E-08	4.13E-07
DIP2B	0.217365912	2.41E-05	7.40E-05
DIP2C	-0.167882014	0.001171251	0.002640749
DIRAS1	0.374701909	8.22E-14	1.00E-12
DIRAS2	0.235496462	4.53E-06	1.60E-05
DIRAS3	-0.227685359	9.47E-06	3.14E-05
DIRC1	0.053367904	0.305268648	0.3777837
DIRC2	0.007275163	0.888929803	0.912396311
DIRC3	0.112837522	0.029779242	0.049111097
DIS3L2	-0.017387799	0.73852465	0.788176358
DIS3L	-0.137059709	0.008204544	0.015422808
DIS3	-0.166504949	0.001287341	0.002876125
DISC1	-0.042742822	0.411713531	0.487911694
DISC2	0.070116398	0.177774548	0.236787092
DISP1	-0.311895356	8.19E-10	5.36E-09
DISP2	0.046331227	0.373536242	0.449479888
DIXDC1	-0.320777766	2.52E-10	1.78E-09
DKC1	0.302315804	2.80E-09	1.69E-08
DKFZP434K	-0.045682563	0.38027094	0.456425935
DKFZP434L	0.247555609	1.38E-06	5.30E-06
DKFZP586I1	-0.084342744	0.104810818	0.149766254
DKFZP686I1	0.233101994	5.70E-06	1.98E-05
DKFZp434J(	0.198467478	0.000118988	0.000324058
DKFZp434L	-0.10526484	0.042730482	0.067820001
DKFZp566F	0.04312083	0.407586486	0.483770912
DKFZp686A	0.088015923	0.090479769	0.131537216
DKFZp686O	0.140418185	0.006749604	0.012926899
DKFZp761E	0.161836161	0.001764164	0.003833097
DKFZp779M	-0.196210117	0.000142633	0.000382597
DKK1	0.254224906	6.98E-07	2.82E-06
DKK2	0.084258929	0.105157643	0.150218741
DKK3	-0.019972765	0.701391553	0.757268726
DKK4	-0.174908869	0.000715071	0.001681299
DKKL1	0.1839578	0.000368372	0.000913571
DLAT	0.116256062	0.025136952	0.042209909
DLC1	-0.289722449	1.32E-08	7.15E-08
DLD	-0.154207878	0.00290076	0.006020308
DLEC1	-0.11961265	0.021199823	0.036218409
DLEU1	-0.11360718	0.028674507	0.04750549
DLEU2L	0.218335303	2.21E-05	6.85E-05
DLEU2	0.24865138	1.24E-06	4.79E-06
DLEU7	0.179111108	0.000527569	0.001271962
DLG1	-0.108519156	0.036676499	0.059129985
DLG2	-0.268396059	1.53E-07	6.92E-07
DLG3	0.459629181	8.63E-21	2.95E-19
DLG4	0.129877699	0.012286995	0.022190328
DLG5	0.119336993	0.021501662	0.036693174
DLGAP1	0.184018052	0.00036671	0.000909901
DLGAP2	0.04731761	0.363437144	0.438971431
DLGAP3	0.114458186	0.027494108	0.045755377
DLGAP4	0.406393523	3.46E-16	6.11E-15
DLGAP5	0.588600507	5.82E-36	3.74E-33
DLK1	0.054769319	0.292725405	0.364456942
DLK2	0.306641523	1.62E-09	1.01E-08

DLL1	0.024139618	0.64303439	0.70655403
DLL3	0.356983253	1.36E-12	1.37E-11
DLL4	0.004532509	0.930665041	0.946195915
DLST	-0.116616091	0.024686465	0.041558539
DLX1	0.069012645	0.184715974	0.244496155
DLX2	0.15347586	0.003039088	0.006281223
DLX3	0.140314566	0.00679079	0.012995784
DLX4	0.362820073	5.51E-13	5.89E-12
DLX5	0.187789293	0.00027553	0.000700321
DLX6AS	0.218982857	2.09E-05	6.50E-05
DLX6	0.232500786	6.03E-06	2.08E-05
DMAP1	0.062089089	0.232855263	0.299383191
DMBT1	0.375163526	7.63E-14	9.34E-13
DMBX1	0.509134462	7.34E-26	5.50E-24
DMC1	0.243152331	2.15E-06	7.97E-06
DMD	-0.42660561	7.70E-18	1.74E-16
DMGDH	-0.542524066	8.87E-30	1.21E-27
DMKN	0.36998291	1.77E-13	2.05E-12
DMP1	0.133850681	0.009849448	0.018215809
DMPK	0.294724147	7.18E-09	4.07E-08
DMRT1	0.24019213	2.88E-06	1.05E-05
DMRT2	0.311148506	9.03E-10	5.88E-09
DMRT3	0.168728473	0.001104756	0.002502192
DMRTA1	-0.29965691	3.90E-09	2.30E-08
DMRTA2	0.123748839	0.017093032	0.029792422
DMRTB1	0.102263349	0.049039605	0.076733193
DMRTC1B	0.180985027	0.000459657	0.00111961
DMRTC1	0.082499497	0.112649605	0.159457541
DMRTC2	0.094659834	0.068574047	0.103197776
DMTF1	0.215349832	2.88E-05	8.73E-05
DMWD	0.121787323	0.018944825	0.032693937
DMXL1	0.005123057	0.921658578	0.938737753
DMXL2	0.167723645	0.001184094	0.002665783
DNA2	0.432669008	2.34E-18	5.71E-17
DNAH10	0.20623126	6.28E-05	0.000180073
DNAH11	-0.05521841	0.288779826	0.36031012
DNAH12	0.114545212	0.027375783	0.045577499
DNAH14	0.388464595	8.22E-15	1.17E-13
DNAH17	0.320327802	2.68E-10	1.89E-09
DNAH1	-0.098577456	0.057834381	0.088704022
DNAH2	0.196862262	0.000135385	0.000364577
DNAH3	0.187832352	0.000274624	0.000698284
DNAH5	0.053089346	0.307803548	0.380471694
DNAH6	-0.047638195	0.360191817	0.435685572
DNAH7	0.186709907	0.000299192	0.000754303
DNAH8	0.120375837	0.020383247	0.034946218
DNAH9	0.065226618	0.210042356	0.273762762
DNAI1	0.160728237	0.001898853	0.004105149
DNAI2	0.127817131	0.013749684	0.024527328
DNAJA1	0.022951238	0.659474838	0.721237248
DNAJA2	-0.27603636	6.51E-08	3.13E-07
DNAJA3	-0.16588206	0.00134325	0.002990303
DNAJA4	0.31645971	4.49E-10	3.06E-09
DNAJB11	0.25648942	5.51E-07	2.26E-06
DNAJB12	-0.045444606	0.382760022	0.45905395
DNAJB13	0.250779542	9.96E-07	3.92E-06
DNAJB14	-0.0677718	0.192755779	0.25395679
DNAJB1	-0.039802424	0.444649938	0.520373381

DNAJB2	0.02142335	0.680854321	0.739302327
DNAJB3	-0.166657063	0.001274017	0.002849552
DNAJB4	-0.140225442	0.006826395	0.013058905
DNAJB5	0.059136833	0.255870067	0.324751716
DNAJB6	0.193264721	0.000180143	0.000474458
DNAJB7	0.035236023	0.498651134	0.572539213
DNAJB8	-0.077445587	0.136510922	0.188309832
DNAJB9	-0.452726021	3.80E-20	1.17E-18
DNAJC10	0.274171726	8.04E-08	3.79E-07
DNAJC11	-0.017661101	0.734568042	0.78479458
DNAJC12	-0.222746149	1.49E-05	4.76E-05
DNAJC13	0.101058692	0.051782061	0.080482787
DNAJC14	0.14287413	0.005836643	0.011315453
DNAJC15	-0.094385923	0.069382299	0.104217308
DNAJC16	-0.315492579	5.11E-10	3.44E-09
DNAJC17	0.006159277	0.905879996	0.926149096
DNAJC18	0.432459768	2.44E-18	5.93E-17
DNAJC19	-0.326324208	1.18E-10	8.81E-10
DNAJC1	0.169978326	0.00101291	0.00230936
DNAJC21	0.116122265	0.025306162	0.042476145
DNAJC22	-0.076132872	0.143303512	0.196415037
DNAJC24	-0.077788549	0.134777717	0.186267199
DNAJC25-G	-0.118175008	0.022815336	0.038739207
DNAJC25	-0.461207188	6.12E-21	2.16E-19
DNAJC27	-0.126527218	0.014741391	0.02607921
DNAJC28	-0.237415486	3.77E-06	1.34E-05
DNAJC2	0.141316736	0.006401719	0.012324608
DNAJC30	-0.422798704	1.61E-17	3.49E-16
DNAJC3	-0.07731785	0.137160824	0.189075418
DNAJC4	0.090761566	0.080828859	0.119120619
DNAJC5B	0.206240333	6.28E-05	0.000179962
DNAJC5G	-0.095310254	0.066685651	0.100614325
DNAJC5	0.399252568	1.25E-15	2.02E-14
DNAJC6	0.211034788	4.18E-05	0.000123825
DNAJC7	0.198385252	0.000119781	0.000326127
DNAJC8	0.13600306	0.008716899	0.016292153
DNAJC9	0.496308987	1.82E-24	1.07E-22
DNAL1	0.034399833	0.508902743	0.581795044
DNAL4	0.051151974	0.325816546	0.399908646
DNALI1	-0.117992104	0.023028319	0.039070871
DNASE1L1	0.028360057	0.58608405	0.654216893
DNASE1L2	-0.075866761	0.144711448	0.197977026
DNASE1L3	-0.417463488	4.45E-17	9.03E-16
DNASE1	0.310668729	9.61E-10	6.23E-09
DNASE2B	0.100452207	0.053210413	0.082454012
DNASE2	-0.016374606	0.753252221	0.80063441
DND1	0.239225698	3.16E-06	1.14E-05
DNER	0.262857285	2.80E-07	1.21E-06
DNHD1	-0.044620799	0.39145375	0.467818933
DNLZ	0.174801624	0.000720579	0.001693189
DNM1L	0.452491637	3.99E-20	1.23E-18
DNM1P35	0.078923111	0.129164606	0.179543489
DNM1	0.227779384	9.38E-06	3.12E-05
DNM2	0.199354145	0.00011075	0.000303322
DNM3	-0.021488028	0.679943946	0.738555329
DNMBP	-0.193936436	0.000170853	0.000451544
DNMT1	0.566836239	6.40E-33	1.70E-30
DNMT3A	0.326226176	1.20E-10	8.92E-10

DNMT3B	0.375901714	6.76E-14	8.38E-13
DNMT3L	-0.28131635	3.55E-08	1.79E-07
DNPEP	-0.147715048	0.004354789	0.008696483
DNTTIP1	0.286885948	1.85E-08	9.76E-08
DNTTIP2	0.09490163	0.067866981	0.102272768
DNTT	-0.084579557	0.103835781	0.148522145
DOC2A	0.202649731	8.46E-05	0.000236795
DOC2B	0.05599436	0.282047042	0.352859908
DOCK10	0.316616942	4.40E-10	3.00E-09
DOCK11	0.108312092	0.03703875	0.059660897
DOCK1	-0.051574487	0.321831104	0.395601854
DOCK2	0.225575358	1.15E-05	3.76E-05
DOCK3	0.338301276	2.19E-11	1.83E-10
DOCK4	-0.231112494	6.87E-06	2.34E-05
DOCK5	-0.005705615	0.912783757	0.93174149
DOCK6	0.13381424	0.009869698	0.018248178
DOCK7	-0.028550517	0.583569795	0.651930547
DOCK8	0.29218183	9.78E-09	5.42E-08
DOCK9	-0.030745039	0.554967763	0.62576793
DOHH	0.078940849	0.12907831	0.179436054
DOK1	0.397983512	1.57E-15	2.49E-14
DOK2	0.214384896	3.13E-05	9.46E-05
DOK3	0.3939707	3.17E-15	4.83E-14
DOK4	-0.075157886	0.148513458	0.202608033
DOK5	0.016390939	0.753014091	0.800547762
DOK6	0.202570958	8.51E-05	0.000238236
DOK7	0.053283564	0.306034686	0.378590599
DOLK	0.009109316	0.861182288	0.891084243
DOLPP1	-0.195893823	0.000146279	0.000391478
DOM3Z	0.240163191	2.88E-06	1.05E-05
DONSON	0.421311827	2.14E-17	4.55E-16
DOPEY1	0.11706034	0.024140212	0.04072843
DOPEY2	0.162804546	0.001653664	0.003610729
DOT1L	0.391487531	4.88E-15	7.22E-14
DPAGT1	0.209162923	4.90E-05	0.000143267
DPCD	0.066098612	0.203998285	0.267038165
DPCR1	0.051929007	0.318511577	0.391811524
DPEP1	0.310970722	9.24E-10	6.01E-09
DPEP2	0.126822939	0.014508649	0.025715419
DPEP3	0.07873336	0.13009057	0.180654143
DPF1	0.351866988	2.97E-12	2.84E-11
DPF2	0.260358415	3.66E-07	1.55E-06
DPF3	-0.279446739	4.41E-08	2.18E-07
DPH1	-0.168572563	0.001116737	0.002527565
DPH2	0.224954287	1.22E-05	3.96E-05
DPH3B	0.116739878	0.024533194	0.041321465
DPH3	0.142719846	0.005890552	0.011414413
DPH5	-0.111135864	0.032351711	0.05290247
DPM1	0.110552353	0.03327643	0.054240962
DPM2	0.278195936	5.09E-08	2.49E-07
DPM3	-0.122678999	0.01808241	0.031344103
DPP10	0.206725277	6.03E-05	0.000173382
DPP3	0.254882114	6.52E-07	2.65E-06
DPP4	-0.361169106	7.14E-13	7.48E-12
DPP6	0.113984478	0.028145912	0.046711334
DPP7	0.015428745	0.767082374	0.812278533
DPP8	-0.029042838	0.577093995	0.646181799
DPP9	0.145791361	0.004897302	0.009670338

DPPA2	0.040365171	0.438233046	0.514071645
DPPA3	0.091391119	0.078737665	0.116443602
DPPA4	0.01538284	0.767755514	0.812840801
DPPA5	0.146599564	0.004662362	0.009251352
DPRXP4	0.087576077	0.092108303	0.133696206
DPRX	-0.053631041	0.302886776	0.375489132
DPT	0.035183204	0.499295445	0.573113842
DPY19L1	-0.062016596	0.233402279	0.299931639
DPY19L2P1	0.138591593	0.007509398	0.014235628
DPY19L2P2	0.182284325	0.00041744	0.001024041
DPY19L2P4	0.093850255	0.070985453	0.106256834
DPY19L2	0.076201515	0.142942038	0.196000502
DPY19L3	0.019484206	0.70835864	0.762682853
DPY19L4	-0.026758433	0.607422181	0.673291309
DPY30	0.097601771	0.060369511	0.09217362
DPYD	0.016503974	0.751366639	0.79897124
DPYSL2	-0.0626357	0.228759807	0.294688191
DPYSL3	0.149223831	0.003967925	0.007992013
DPYSL4	0.299193779	4.14E-09	2.43E-08
DPYSL5	0.252448129	8.39E-07	3.34E-06
DPYS	-0.327496727	1.01E-10	7.58E-10
DQX1	0.387859395	9.12E-15	1.28E-13
DR1	0.311251015	8.91E-10	5.81E-09
DRAM1	0.416453074	5.38E-17	1.08E-15
DRAM2	0.264385384	2.37E-07	1.04E-06
DRAP1	0.226213266	1.08E-05	3.56E-05
DRD1	-0.433665046	1.92E-18	4.76E-17
DRD2	0.181136813	0.000454528	0.001108167
DRD3	0.053338753	0.305533276	0.378087703
DRD4	-0.011000106	0.832755864	0.86723274
DRD5	-0.033704164	0.51751464	0.590082799
DRG1	0.124895536	0.016085415	0.028196536
DRG2	0.016576236	0.750314042	0.798147627
DRGX	-0.008413302	0.871693576	0.899041179
DRP2	0.440860741	4.49E-19	1.22E-17
DSC1	-0.183387471	0.000384457	0.000949914
DSC2	0.261374513	3.28E-07	1.40E-06
DSC3	0.170752747	0.000959589	0.002197853
DSCAML1	0.312083825	8.00E-10	5.24E-09
DSCAM	-0.068808039	0.186024383	0.246064521
DSCC1	0.504403114	2.44E-25	1.70E-23
DSCR10	0.061375533	0.238279117	0.305410575
DSCR3	-0.123779652	0.017065252	0.029751323
DSCR4	0.055140669	0.289460274	0.36106866
DSCR6	0.26658211	1.87E-07	8.32E-07
DSCR8	-0.094127271	0.070152659	0.105215785
DSCR9	0.210294649	4.45E-05	0.00013138
DSEL	0.202081981	8.86E-05	0.000247114
DSE	0.148936546	0.0040391	0.008120591
DSG1	-0.542672873	8.50E-30	1.18E-27
DSG2	0.439260732	6.22E-19	1.66E-17
DSG3	0.09592919	0.064928473	0.098275721
DSG4	-0.15506954	0.002745281	0.005731659
DSN1	0.400574333	9.89E-16	1.62E-14
DSPP	0.076423811	0.141776218	0.194589446
DSP	-0.07009427	0.177911785	0.236938203
DSTN	0.167062931	0.001239083	0.002778279
DSTYK	0.24366465	2.04E-06	7.60E-06

DST	-0.155029672	0.002752304	0.005742927
DTD1	0.109499657	0.035001708	0.056709827
DTHD1	0.040929492	0.431851791	0.507931951
DTL	0.490851265	6.84E-24	3.66E-22
DTNA	0.028755435	0.580870292	0.649570472
DTNBP1	0.454494871	2.61E-20	8.29E-19
DTNB	0.206103148	6.35E-05	0.000181938
DTWD1	-0.150406481	0.003686715	0.007477017
DTWD2	-0.020254415	0.697386377	0.753638807
DTX1	-0.085989125	0.098179795	0.141406729
DTX2	0.293671494	8.16E-09	4.59E-08
DTX3L	-0.066562257	0.200836408	0.263453146
DTX3	0.387873224	9.10E-15	1.28E-13
DTX4	0.1724109	0.000854015	0.001977876
DTYMK	0.422950705	1.56E-17	3.40E-16
DULLARD	0.227135382	9.96E-06	3.29E-05
DUOX1	0.177335482	0.000600404	0.001433515
DUOX2	0.403433768	5.92E-16	1.01E-14
DUOXA1	0.280944531	3.71E-08	1.86E-07
DUOXA2	0.390819853	5.48E-15	8.01E-14
DUPD1	0.065529325	0.207929734	0.271435434
DUS1L	-0.06357628	0.221832616	0.287175865
DUS2L	-0.082112258	0.114353493	0.161559018
DUS3L	0.065257329	0.209827317	0.273571977
DUS4L	-0.057114328	0.272517584	0.342616137
DUSP10	-0.18366432	0.000376569	0.000931696
DUSP11	0.178023008	0.000571162	0.001368782
DUSP12	0.427188268	6.88E-18	1.56E-16
DUSP13	0.337045709	2.63E-11	2.16E-10
DUSP14	0.037178562	0.475264267	0.549966004
DUSP15	0.294401291	7.47E-09	4.22E-08
DUSP16	-0.333069196	4.62E-11	3.67E-10
DUSP18	0.169747255	0.001029341	0.002344408
DUSP19	-0.268194282	1.56E-07	7.06E-07
DUSP1	-0.154294944	0.002884694	0.005990708
DUSP21	0.113370583	0.029010304	0.04799215
DUSP22	0.170763912	0.000958839	0.002196389
DUSP23	0.072106681	0.165749737	0.222781423
DUSP26	0.305645549	1.84E-09	1.14E-08
DUSP27	0.128807666	0.013028461	0.02337473
DUSP28	0.144056023	0.005438146	0.010610003
DUSP2	0.214534706	3.09E-05	9.34E-05
DUSP3	-0.127274509	0.014159477	0.025156969
DUSP4	0.31260037	7.47E-10	4.91E-09
DUSP5P	0.281348557	3.54E-08	1.79E-07
DUSP5	0.229240375	8.19E-06	2.75E-05
DUSP6	-0.046283995	0.374024126	0.449985424
DUSP7	0.23575109	4.42E-06	1.56E-05
DUSP8	-0.013203555	0.799904058	0.839747144
DUSP9	0.274253083	7.96E-08	3.76E-07
DUT	-0.094746531	0.068319833	0.102885155
DUXA	0.079026085	0.128664243	0.178935361
DVL1	-0.119970677	0.020813282	0.035625242
DVL2	0.2142196	3.17E-05	9.58E-05
DVL3	0.326999002	1.08E-10	8.09E-10
DVWA	0.241695708	2.48E-06	9.12E-06
DYDC1	0.172372776	0.000856317	0.001982054
DYDC2	0.174678573	0.000726946	0.001706601

DYM	0.186128512	0.000312709	0.000786192
DYNC1H1	0.389556438	6.82E-15	9.84E-14
DYNC1I1	-0.03335153	0.521908522	0.594158395
DYNC1I2	0.1577908	0.00230273	0.004894377
DYNC1LI1	0.49185332	5.37E-24	2.95E-22
DYNC1LI2	-0.036356894	0.485082783	0.559189018
DYNC2H1	0.036363367	0.485005008	0.559189018
DYNC2LI1	0.08982029	0.084039054	0.123295238
DYNLL1	0.327962686	9.44E-11	7.14E-10
DYNLL2	-0.269770495	1.31E-07	6.00E-07
DYNLRB1	0.264837503	2.26E-07	9.93E-07
DYNLRB2	-0.111338604	0.032035593	0.052441503
DYNLT1	0.382021227	2.45E-14	3.22E-13
DYNLT3	-0.106471013	0.040395115	0.064524654
DYRK1A	0.039142204	0.452245902	0.527867521
DYRK1B	0.064792846	0.213096601	0.277290038
DYRK2	0.41023138	1.72E-16	3.19E-15
DYRK3	0.050781019	0.329341892	0.403415468
DYRK4	0.072697813	0.162298389	0.21888089
DYSFIP1	-0.192770458	0.000187278	0.000491237
DYSF	-0.167000079	0.001244434	0.002789336
DYX1C1	0.342802237	1.14E-11	9.98E-11
DZIP1L	0.494030482	3.17E-24	1.79E-22
DZIP1	0.187050384	0.000291531	0.00073704
DZIP3	-0.072118183	0.16568206	0.222705486
E2F1	0.471653284	6.01E-22	2.42E-20
E2F2	0.552914063	4.33E-31	7.31E-29
E2F3	0.519702726	4.70E-27	4.26E-25
E2F4	0.253175504	7.78E-07	3.11E-06
E2F5	0.280655903	3.83E-08	1.92E-07
E2F6	0.349178981	4.45E-12	4.14E-11
E2F7	0.431986576	2.68E-18	6.46E-17
E2F8	0.449044933	8.26E-20	2.46E-18
E4F1	-0.137081078	0.008194465	0.015411134
EAF1	0.007624369	0.883635408	0.908066884
EAF2	0.265765523	2.04E-07	9.03E-07
EAPP	-0.218802828	2.12E-05	6.59E-05
EARS2	-0.151968002	0.003343078	0.006842079
EBAG9	-0.060543747	0.244713025	0.312453055
EBF1	0.073943986	0.155199906	0.210447354
EBF2	-0.055191711	0.289013391	0.360578956
EBF3	-0.142231563	0.006064125	0.011719974
EBF4	0.225947829	1.11E-05	3.64E-05
EBI3	0.20766481	5.57E-05	0.000161073
EBNA1BP2	0.034313591	0.509966282	0.582843685
EBPL	-0.385952826	1.26E-14	1.73E-13
EBP	-0.052293344	0.315123488	0.388123038
ECD	0.199774356	0.000107036	0.000293957
ECE1	0.036086419	0.488338601	0.562449072
ECE2	0.090390511	0.082082288	0.120771295
ECEL1	0.26355055	2.60E-07	1.13E-06
ECH1	-0.37459358	8.37E-14	1.02E-12
ECHDC1	-0.079337416	0.127160569	0.177128573
ECHDC2	-0.466343404	1.98E-21	7.41E-20
ECHDC3	-0.344262349	9.22E-12	8.16E-11
ECHS1	-0.484437717	3.15E-23	1.53E-21
ECM1	0.208850242	5.04E-05	0.000146844
ECM2	-0.5670497	5.99E-33	1.61E-30

ECSCR	-0.086122266	0.097658478	0.140757647
ECSIT	-0.223307178	1.41E-05	4.54E-05
ECT2L	-0.011716158	0.822045139	0.858636987
ECT2	0.55353563	3.60E-31	6.19E-29
EDA2R	-0.098056001	0.059178094	0.090512226
EDARADD	0.191619776	0.000204926	0.000533735
EDAR	-0.123138875	0.01765116	0.030655253
EDA	0.348678182	4.80E-12	4.43E-11
EDC3	0.252616533	8.24E-07	3.28E-06
EDC4	0.066550049	0.200919204	0.263527051
EDDM3A	-0.082213161	0.113907577	0.160997996
EDDM3B	-0.042857174	0.410462453	0.486653157
EDEM1	-0.363078502	5.29E-13	5.68E-12
EDEM2	0.064149674	0.217683824	0.282520987
EDEM3	-0.020460035	0.694467663	0.750932739
EDF1	0.040526511	0.43640314	0.512589078
EDIL3	0.061037447	0.240879733	0.308248075
EDN1	-0.054238883	0.297431888	0.369577625
EDN2	0.017859436	0.731701106	0.782697027
EDN3	-0.027148487	0.602193819	0.668835661
EDNRA	0.210089406	4.53E-05	0.000133546
EDNRB	-0.211217158	4.11E-05	0.000122011
EEA1	-0.006394862	0.902297665	0.923199468
EED	0.486685145	1.85E-23	9.36E-22
EEF1A1P9	0.077693145	0.135258146	0.186788631
EEF1A1	0.03327749	0.522833492	0.594955665
EEF1A2	-0.0185711	0.721444939	0.774136921
EEF1B2	0.012540627	0.809753624	0.848110599
EEF1DP3	0.158634921	0.002179309	0.004652399
EEF1D	0.13859175	0.007509329	0.014235628
EEF1E1	0.46753824	1.51E-21	5.76E-20
EEF1G	0.099655271	0.055136991	0.085090954
EEF2K	-0.007301484	0.888530569	0.912156343
EEF2	-0.16988329	0.001019638	0.002324169
EEFSEC	-0.243130854	2.15E-06	7.98E-06
EEPD1	0.020494697	0.693976088	0.750645652
EFCAB10	0.093711177	0.071406598	0.106823027
EFCAB1	-0.095969556	0.064815207	0.098111736
EFCAB2	0.089304129	0.085842575	0.12564077
EFCAB3	0.253186738	7.77E-07	3.11E-06
EFCAB4A	0.328271474	9.04E-11	6.87E-10
EFCAB4B	0.173171299	0.000809283	0.001883473
EFCAB5	0.068416395	0.188547858	0.249005664
EFCAB6	-0.302227543	2.83E-09	1.71E-08
EFCAB7	0.235423663	4.56E-06	1.61E-05
EFEMP1	0.123990311	0.016876378	0.029453457
EFEMP2	0.236479361	4.12E-06	1.46E-05
EFHA1	-0.010422835	0.841413361	0.874420291
EFHA2	-0.330984419	6.20E-11	4.84E-10
EFHB	-0.019196602	0.712471445	0.76628279
EFHC1	0.319872161	2.85E-10	2.00E-09
EFHC2	0.288473953	1.53E-08	8.18E-08
EFHD1	-0.214780043	3.02E-05	9.16E-05
EFHD2	0.232580314	5.98E-06	2.06E-05
EFNA1	0.143954488	0.005471394	0.010669646
EFNA2	-0.002252791	0.965505871	0.972929083
EFNA3	0.28009238	4.09E-08	2.04E-07
EFNA4	0.169862718	0.0010211	0.002327235

EFNA5	0.36465436	4.13E-13	4.50E-12
EFNB1	0.28906679	1.42E-08	7.68E-08
EFNB2	-0.005048092	0.922801337	0.939639572
EFNB3	0.066636434	0.200333877	0.26286317
EFR3A	-0.168481266	0.001123807	0.002541836
EFR3B	0.234436046	5.02E-06	1.75E-05
EFS	0.251931298	8.85E-07	3.51E-06
EFTUD1	0.12425749	0.016639462	0.029080722
EFTUD2	0.326059366	1.23E-10	9.11E-10
EGFL6	0.33706556	2.62E-11	2.15E-10
EGFL7	0.077432058	0.136579643	0.188378541
EGFL8	0.187310409	0.000285804	0.000724074
EGFLAM	-0.064290888	0.216670648	0.281379693
EGFR	-0.099216772	0.056221524	0.086576888
EGF	0.22592406	1.11E-05	3.65E-05
EGLN1	-0.086855228	0.094827884	0.13711432
EGLN2	0.248862657	1.21E-06	4.70E-06
EGLN3	0.474710363	3.00E-22	1.28E-20
EGOT	0.044198388	0.395957431	0.472294572
EGR1	-0.008229132	0.874478799	0.9010741
EGR2	0.199303879	0.000111202	0.000304393
EGR3	0.092730138	0.074435556	0.11074721
EGR4	0.150068044	0.003765286	0.007621625
EHBP1L1	0.315995257	4.78E-10	3.24E-09
EHBP1	-0.3854078	1.38E-14	1.89E-13
EHD1	0.212537754	3.67E-05	0.000109892
EHD2	0.138976533	0.007343204	0.013955099
EHD3	0.171372202	0.000918814	0.002110285
EHD4	0.086384068	0.096639813	0.139460945
EHF	0.262454618	2.92E-07	1.26E-06
EHHADH	-0.608221295	6.59E-39	7.72E-36
EHMT1	-0.007002565	0.89306617	0.915538735
EHMT2	0.313813679	6.37E-10	4.23E-09
EI24	-0.272748262	9.43E-08	4.40E-07
EID1	-0.102293374	0.048972835	0.076658821
EID2B	0.276770661	5.99E-08	2.89E-07
EID2	0.090742446	0.080893067	0.119188794
EID3	0.333128083	4.58E-11	3.64E-10
EIF1AD	0.16191371	0.001755072	0.00381584
EIF1AX	0.011397485	0.826807896	0.862345789
EIF1AY	-0.04947742	0.341924956	0.416218316
EIF1B	0.017714561	0.733794902	0.784184051
EIF1	0.002368537	0.963734786	0.97143536
EIF2AK1	-0.020333493	0.696263362	0.752629338
EIF2AK2	0.261198853	3.34E-07	1.43E-06
EIF2AK3	-0.015084472	0.772134906	0.816479385
EIF2AK4	-0.332032682	5.35E-11	4.20E-10
EIF2A	-0.008873849	0.864735702	0.893715743
EIF2B1	0.170975812	0.000944717	0.002165534
EIF2B2	0.127675261	0.013855806	0.024685688
EIF2B3	-0.110478081	0.033395733	0.054417618
EIF2B4	-0.028831106	0.579874909	0.648530171
EIF2B5	0.097240239	0.061331878	0.093399608
EIF2C1	-0.095489246	0.066173525	0.099940032
EIF2C2	0.290542572	1.19E-08	6.53E-08
EIF2C3	-0.000590313	0.990958674	0.992856086
EIF2C4	-0.12329121	0.017510301	0.030442466
EIF2S1	0.114969375	0.026805319	0.044728668

EIF2S2	0.421500066	2.06E-17	4.40E-16
EIF2S3	0.412836373	1.06E-16	2.04E-15
EIF3A	-0.048410605	0.352447292	0.427173289
EIF3B	0.205057329	6.93E-05	0.000196971
EIF3CL	0.112018377	0.030994846	0.050867397
EIF3C	0.191669556	0.000204131	0.000531911
EIF3D	0.179026297	0.000530852	0.00127926
EIF3E	0.072055326	0.166052154	0.223127682
EIF3F	0.084593134	0.103780097	0.148453155
EIF3G	0.055291327	0.288142583	0.35962765
EIF3H	0.150513983	0.00366207	0.007436877
EIF3IP1	0.060483828	0.24518115	0.312968467
EIF3I	0.148642582	0.004113122	0.008251925
EIF3J	0.175868916	0.000667476	0.00157927
EIF3K	0.192276085	0.000194678	0.00050904
EIF3L	-0.107039725	0.03933194	0.062952591
EIF3M	0.187367792	0.000284554	0.000721416
EIF4A1	0.205301925	6.79E-05	0.000193352
EIF4A2	0.022464561	0.66625586	0.726738351
EIF4A3	0.246614894	1.52E-06	5.79E-06
EIF4B	-0.097527858	0.060565239	0.092401646
EIF4E1B	0.156005638	0.002584995	0.00544239
EIF4E2	0.142544185	0.005952475	0.011525435
EIF4E3	0.197462524	0.00012902	0.000348709
EIF4EBP1	0.254331308	6.90E-07	2.79E-06
EIF4EBP2	-0.328660179	8.57E-11	6.54E-10
EIF4EBP3	-0.228609841	8.69E-06	2.90E-05
EIF4ENIF1	0.135011414	0.009223437	0.017155197
EIF4E	0.060598516	0.244285689	0.312025357
EIF4G1	-0.156674915	0.002475673	0.005230154
EIF4G2	0.144815107	0.005195283	0.010189382
EIF4G3	0.029149644	0.575693571	0.644831177
EIF4H	-0.039849785	0.444107837	0.519800032
EIF5A2	0.320724106	2.54E-10	1.80E-09
EIF5AL1	0.067922437	0.191766317	0.252753461
EIF5A	0.102134262	0.049327545	0.077123164
EIF5B	0.108529712	0.036658113	0.059105126
EIF5	-0.202912332	8.28E-05	0.00023195
EIF6	0.037207763	0.474917344	0.549660334
ELAC1	-0.230483564	7.29E-06	2.47E-05
ELAC2	-0.007588063	0.884185612	0.908535658
ELANE	0.033408708	0.521194778	0.593429867
ELAVL1	0.24039722	2.82E-06	1.03E-05
ELAVL2	-0.30120445	3.22E-09	1.92E-08
ELAVL3	0.112760633	0.029891581	0.049284125
ELAVL4	0.21228583	3.75E-05	0.000112173
ELF1	0.082596362	0.112226519	0.158937706
ELF2	-0.028876579	0.579277127	0.648235812
ELF3	0.343820339	9.84E-12	8.67E-11
ELF4	0.406243218	3.56E-16	6.28E-15
ELF5	-0.060254145	0.246981356	0.314983841
ELFN1	-0.380054541	3.40E-14	4.38E-13
ELFN2	0.074110402	0.154270012	0.209414499
ELK1	0.038148048	0.463820458	0.538726164
ELK3	0.02765976	0.595371375	0.662588042
ELK4	-0.118288553	0.022683977	0.038530238
ELL2	-0.222077216	1.58E-05	5.03E-05
ELL3	0.140598249	0.006678563	0.012800687

ELL	0.18732185	0.000285554	0.000723583
ELMO1	-0.104314338	0.044650001	0.070579807
ELMO2	0.267917252	1.61E-07	7.26E-07
ELMO3	0.129436108	0.012588354	0.022672921
ELMOD1	0.13246357	0.010646786	0.019509316
ELMOD2	-0.234566525	4.95E-06	1.73E-05
ELMOD3	-0.064508742	0.215114241	0.279696247
ELN	-0.030752322	0.554873987	0.625697604
ELOF1	0.092931653	0.073804989	0.109973298
ELOVL1	0.385285249	1.41E-14	1.92E-13
ELOVL2	-0.124655927	0.016291556	0.028525257
ELOVL3	0.16444371	0.001480978	0.003272086
ELOVL4	0.295110961	6.85E-09	3.89E-08
ELOVL5	-0.139331278	0.007192965	0.013695713
ELOVL6	-0.12069433	0.02005064	0.034411514
ELOVL7	0.526173455	8.33E-28	8.51E-26
ELP2P	0.1362722	0.008583763	0.016064418
ELP2	-0.209499316	4.77E-05	0.000139654
ELP3	-0.128971166	0.012912674	0.023185785
ELP4	-0.007858897	0.880082555	0.905256333
ELSPBP1	0.011202683	0.829722479	0.864797072
ELTD1	-0.193214009	0.000180863	0.000476166
EMB	0.280280831	4.00E-08	2.00E-07
EMCN	-0.306549643	1.64E-09	1.02E-08
EMD	0.02013421	0.699094716	0.755157237
EME1	0.466318522	1.99E-21	7.44E-20
EME2	0.093120933	0.073216677	0.109202863
EMG1	0.165640277	0.001365546	0.003037224
EMID1	0.20183393	9.05E-05	0.000251778
EMID2	-0.045340863	0.383848306	0.460082182
EMILIN1	0.193002265	0.000183899	0.000483329
EMILIN2	0.463197587	3.96E-21	1.43E-19
EMILIN3	0.139760092	0.007015028	0.013385332
EML1	0.006676271	0.898021123	0.919624592
EML2	0.161045385	0.001859367	0.004026776
EML3	-0.016277435	0.754669493	0.801713093
EML4	0.001973272	0.969783611	0.976202774
EML5	-0.039387419	0.449416163	0.525210817
EML6	-0.138888636	0.00738086	0.014018638
EMP1	-0.054997222	0.290718639	0.362411409
EMP2	-0.236672307	4.05E-06	1.44E-05
EMP3	0.307172855	1.51E-09	9.48E-09
EMR1	0.063275112	0.224034182	0.289669323
EMR2	0.292799907	9.08E-09	5.06E-08
EMR3	0.232718555	5.91E-06	2.04E-05
EMR4P	0.17869194	0.000543983	0.001308824
EMX1	0.027859798	0.592711662	0.660291994
EMX2OS	-0.048627209	0.350294511	0.424978214
EMX2	-0.118769699	0.022134584	0.037657146
EN1	0.146308735	0.004745707	0.009401748
EN2	0.254201914	7.00E-07	2.83E-06
ENAH	0.402923342	6.49E-16	1.10E-14
ENAM	0.018046387	0.729002145	0.780354669
ENC1	0.069699114	0.180375725	0.239626913
ENDOD1	0.143419055	0.005649764	0.010984151
ENDOG	-0.163491345	0.001579179	0.003462515
ENDOU	-0.074282589	0.153312316	0.208284776
ENGASE	0.02875849	0.580830091	0.649561978

ENG	-0.142470614	0.005978583	0.011572612
ENHO	0.120962586	0.019774166	0.03397214
ENKUR	-0.012396371	0.811900969	0.849823523
ENO1	0.336394799	2.88E-11	2.36E-10
ENO2	0.426433827	7.97E-18	1.79E-16
ENO3	-0.115894401	0.025596598	0.042913021
ENOPH1	0.314253241	6.02E-10	4.01E-09
ENOSF1	-0.11622234	0.025179507	0.042277804
ENOX1	0.113775308	0.028437917	0.047152741
ENOX2	-0.020881389	0.688500794	0.745739142
ENPEP	-0.301860173	2.96E-09	1.78E-08
ENPP1	-0.357076277	1.34E-12	1.35E-11
ENPP2	0.157927884	0.002282263	0.004853981
ENPP3	-0.038791615	0.456309	0.531551157
ENPP4	0.093830518	0.071045097	0.106322147
ENPP5	0.338478642	2.14E-11	1.78E-10
ENPP6	0.137271094	0.008105329	0.015255017
ENPP7	-0.223115539	1.44E-05	4.62E-05
ENSA	0.156381611	0.002523053	0.005322912
ENTHD1	0.218984458	2.09E-05	6.50E-05
ENTPD1	0.319151171	3.14E-10	2.18E-09
ENTPD2	0.289732319	1.31E-08	7.15E-08
ENTPD3	0.202988033	8.23E-05	0.000230632
ENTPD4	0.05869444	0.259449938	0.328822551
ENTPD5	-0.268432021	1.52E-07	6.89E-07
ENTPD6	0.371589867	1.36E-13	1.61E-12
ENTPD7	-0.031462608	0.545765713	0.616930032
ENTPD8	-0.019911078	0.702269872	0.757888429
ENY2	0.145397841	0.005015512	0.009876349
EOMES	0.023674631	0.649447034	0.712303668
EP300	-0.064070828	0.218250992	0.283183292
EP400NL	0.042727674	0.41187942	0.488071723
EP400	0.303049259	2.55E-09	1.55E-08
EPAS1	-0.19272921	0.000187885	0.000492635
EPB41L1	0.377830387	4.92E-14	6.20E-13
EPB41L2	0.37799305	4.79E-14	6.05E-13
EPB41L3	0.225316848	1.18E-05	3.84E-05
EPB41L4A	0.140734681	0.006625184	0.012711828
EPB41L4B	-0.352943872	2.53E-12	2.44E-11
EPB41L5	-0.300987529	3.31E-09	1.97E-08
EPB41	-0.037645617	0.469731982	0.544544693
EPB42	-0.102459223	0.048605374	0.0761734
EPB49	0.14833132	0.004192835	0.008395784
EPC1	-0.074681777	0.151109377	0.20567063
EPC2	-0.05337387	0.305214502	0.377740157
EPCAM	0.414537704	7.71E-17	1.51E-15
EPDR1	0.079765689	0.125114381	0.174705874
EPGN	0.056164196	0.280587656	0.351298848
EPHA10	0.32593878	1.25E-10	9.25E-10
EPHA1	0.002119675	0.967542959	0.974428422
EPHA2	0.063662157	0.22120769	0.286459932
EPHA3	0.128799156	0.013034512	0.023383481
EPHA4	-0.071833598	0.167362596	0.224736966
EPHA5	0.148517763	0.004144922	0.008307358
EPHA6	0.188578632	0.000259346	0.000661632
EPHA7	0.007075738	0.891955574	0.914823875
EPHA8	0.183834928	0.000371784	0.000921114
EPHB1	0.105902261	0.041482535	0.066081486

EPHB2	0.033474768	0.520370782	0.592627149
EPHB3	0.392207507	4.31E-15	6.43E-14
EPHB4	0.092103988	0.076422879	0.11338223
EPHB6	0.292654827	9.24E-09	5.14E-08
EPHX1	-0.370359238	1.66E-13	1.93E-12
EPHX2	-0.466676133	1.83E-21	6.91E-20
EPHX3	0.321676008	2.23E-10	1.59E-09
EPHX4	0.342641444	1.17E-11	1.02E-10
EPM2AIP1	-0.198728254	0.000116507	0.000317779
EPM2A	-0.344694335	8.66E-12	7.69E-11
EPN1	-0.185948706	0.000317003	0.000796085
EPN2	0.051048693	0.326795609	0.400859645
EPN3	0.352851447	2.56E-12	2.47E-11
EPOR	0.195612178	0.000149598	0.000399771
EPO	0.318126489	3.60E-10	2.48E-09
EPPK1	0.417233661	4.65E-17	9.41E-16
EPR1	0.538462564	2.80E-29	3.58E-27
EPRS	0.299985131	3.75E-09	2.21E-08
EPS15L1	0.410187131	1.73E-16	3.21E-15
EPS15	-0.134170738	0.009673175	0.017916417
EPS8L1	0.424186413	1.23E-17	2.70E-16
EPS8L2	0.198208511	0.000121501	0.000330268
EPS8L3	0.351167528	3.30E-12	3.13E-11
EPS8	-0.044544727	0.392262528	0.468616819
EPSTI1	0.053866421	0.300766619	0.373185886
EPT1	-0.128578575	0.013192236	0.023621761
EPX	-0.065330411	0.209316238	0.273030714
EPYC	0.212201938	3.78E-05	0.00011287
ERAL1	0.066477083	0.20141458	0.264089855
ERAP1	-0.012844125	0.805240522	0.844404762
ERAP2	0.150201419	0.003734143	0.007566272
ERAS	-0.081917371	0.115218624	0.162643338
ERBB2IP	0.026801671	0.606841618	0.672760084
ERBB2	-0.056465834	0.278008334	0.348573355
ERBB3	0.025861796	0.619516659	0.68445047
ERBB4	0.05924163	0.255027074	0.323876418
ERC1	-0.068558571	0.187628888	0.247956398
ERC2	0.24575098	1.66E-06	6.26E-06
ERCC1	0.066764238	0.199470179	0.261838564
ERCC2	-0.125880285	0.015262001	0.026902282
ERCC3	0.191587638	0.000205441	0.000535005
ERCC4	-0.26067863	3.54E-07	1.50E-06
ERCC5	-0.227837183	9.33E-06	3.10E-05
ERCC6L	0.502349825	4.08E-25	2.74E-23
ERCC6	-0.089840254	0.083969916	0.123230104
ERCC8	0.151348115	0.003475857	0.007089795
EREG	0.25097967	9.76E-07	3.85E-06
ERF	-0.055658168	0.284950993	0.356068177
ERGIC1	-0.001838031	0.971853648	0.977891225
ERGIC2	0.17770055	0.000584708	0.001399394
ERGIC3	0.057951379	0.26554028	0.33515544
ERG	-0.156041805	0.002578977	0.005432259
ERH	0.31184865	8.24E-10	5.40E-09
ERI1	0.282987529	2.92E-08	1.49E-07
ERI2	-0.292272908	9.68E-09	5.36E-08
ERI3	0.054366492	0.296295049	0.368279883
ERICH1	0.083585813	0.107975978	0.153704711
ERLEC1	-0.036928875	0.478236359	0.552654244

ERLIN1	-0.285551936	2.16E-08	1.13E-07
ERLIN2	-0.118555517	0.022377706	0.038057766
ERMAP	-0.47971383	9.50E-23	4.39E-21
ERMN	0.21252807	3.68E-05	0.000109967
ERMP1	0.237636577	3.69E-06	1.32E-05
ERN1	-0.231351355	6.72E-06	2.30E-05
ERN2	0.222127358	1.57E-05	5.01E-05
ERO1LB	-0.140881664	0.006568104	0.012619323
ERO1L	0.229170581	8.25E-06	2.77E-05
ERP27	0.32846732	8.80E-11	6.70E-10
ERP29	0.116332325	0.02504094	0.042066414
ERP44	-0.011886629	0.819500015	0.856562397
ERRFI1	-0.223070627	1.44E-05	4.63E-05
ERVFRDE1	-0.172773199	0.000832425	0.001931915
ESAM	-0.071409432	0.169891002	0.227763698
ESCO1	0.248198335	1.30E-06	4.99E-06
ESCO2	0.474289103	3.31E-22	1.38E-20
ESD	-0.245317151	1.73E-06	6.52E-06
ESF1	0.070216112	0.177157113	0.236027821
ESM1	-0.084780529	0.103013951	0.147569132
ESPL1	0.393431898	3.48E-15	5.26E-14
ESPNL	-0.023057691	0.657995289	0.719974853
ESPNP	-0.19588485	0.000146383	0.000391653
ESPN	-0.344773018	8.56E-12	7.62E-11
ESR1	-0.483619108	3.82E-23	1.83E-21
ESR2	0.100844792	0.052282132	0.081181967
ESRP1	0.409482122	1.97E-16	3.62E-15
ESRP2	-0.155372533	0.002692437	0.005635678
ESRRA	0.102219678	0.04913686	0.076879332
ESRRB	0.018935392	0.716214055	0.769601748
ESRRG	-0.009539207	0.854702097	0.885689216
ESX1	0.10788831	0.037789606	0.060718165
ESYT1	-0.064195373	0.217355572	0.282150108
ESYT2	-0.13389522	0.009824748	0.018175192
ESYT3	0.40290666	6.51E-16	1.10E-14
ETAA1	0.152561713	0.003220236	0.006618543
ETF1	0.293074601	8.78E-09	4.91E-08
ETFA	-0.442672356	3.10E-19	8.55E-18
ETFB	-0.215737565	2.78E-05	8.47E-05
ETFDH	-0.484159954	3.36E-23	1.63E-21
ETHE1	-0.177386625	0.000598182	0.00142838
ETNK1	0.135095805	0.009179333	0.017082734
ETNK2	-0.382300409	2.34E-14	3.08E-13
ETS1	-0.00947544	0.855662716	0.886638543
ETS2	-0.033041197	0.525791073	0.597843146
ETV1	0.003220402	0.950706046	0.961810169
ETV2	-0.079005482	0.128764234	0.179035472
ETV3L	0.115363106	0.026284963	0.043963482
ETV3	-0.00091649	0.985963315	0.989340242
ETV4	0.347568489	5.66E-12	5.16E-11
ETV5	0.275662933	6.79E-08	3.25E-07
ETV6	0.386323747	1.18E-14	1.63E-13
ETV7	0.222583844	1.51E-05	4.83E-05
EVC2	0.429270777	4.57E-18	1.07E-16
EVC	0.328072216	9.30E-11	7.04E-10
EVI2A	0.258414355	4.50E-07	1.87E-06
EVI2B	0.200912036	9.76E-05	0.000270127
EVI5L	0.159185206	0.002102133	0.004505509

EVI5	-0.173001479	0.000819081	0.001903386
EVL	0.071019571	0.17223997	0.230416645
EVPLL	-0.029963208	0.565079097	0.635012736
EVPL	0.217252775	2.43E-05	7.47E-05
EVX1	0.114266043	0.027756908	0.046127221
EWSR1	0.34783821	5.44E-12	4.97E-11
EXD1	0.105926191	0.041436289	0.066023655
EXD2	0.108811839	0.036169593	0.05837889
EXD3	0.081410801	0.117491345	0.165441234
EXO1	0.554533011	2.68E-31	4.81E-29
EXOC1	0.082948383	0.110699475	0.15705419
EXOC2	0.123071327	0.017713936	0.030756234
EXOC3L2	-0.256358819	5.58E-07	2.29E-06
EXOC3L	-0.070025772	0.178337094	0.237409395
EXOC3	0.162509663	0.001686621	0.003677853
EXOC4	-0.20593724	6.44E-05	0.000184166
EXOC5	-0.053493611	0.304129231	0.37672465
EXOC6B	-0.010163866	0.845303471	0.877638927
EXOC6	0.265774883	2.04E-07	9.03E-07
EXOC7	0.014275614	0.78404326	0.82692095
EXOC8	-0.081408104	0.117503541	0.165446713
EXOG	0.282457946	3.11E-08	1.58E-07
EXOSC10	0.052732677	0.311069458	0.383889348
EXOSC1	0.208137204	5.35E-05	0.00015522
EXOSC2	0.183236187	0.000388832	0.000960129
EXOSC3	0.346240852	6.89E-12	6.22E-11
EXOSC4	0.173684917	0.000780304	0.001821136
EXOSC5	0.086594713	0.095826361	0.138387297
EXOSC6	-0.065167204	0.210458822	0.274233806
EXOSC7	0.165916164	0.001340132	0.002984363
EXOSC8	0.152054598	0.0033249	0.006810472
EXOSC9	0.299839802	3.82E-09	2.25E-08
EXPH5	-0.410792418	1.55E-16	2.89E-15
EXT1	0.290271385	1.23E-08	6.73E-08
EXT2	0.071589209	0.16881591	0.226429042
EXTL1	0.093458023	0.072178412	0.107839982
EXTL2	-0.070575779	0.174943238	0.233452957
EXTL3	0.133272798	0.010174951	0.018746451
EYA1	0.207407771	5.69E-05	0.000164342
EYA2	0.245213042	1.75E-06	6.58E-06
EYA3	0.030777467	0.554550288	0.625545033
EYA4	0.143268136	0.005700975	0.011076148
EYS	-0.012769396	0.80635116	0.845304249
EZH1	-0.029851232	0.566534453	0.636253418
EZH2	0.55037309	9.15E-31	1.46E-28
EZR	0.381531006	2.66E-14	3.49E-13
F10	-0.246069451	1.61E-06	6.09E-06
F11R	0.123806295	0.017041262	0.029715189
F11	-0.538924474	2.46E-29	3.16E-27
F12	-0.416967057	4.89E-17	9.86E-16
F13A1	0.081667879	0.116333629	0.163984897
F13B	-0.323437927	1.76E-10	1.28E-09
F2RL1	0.329039601	8.13E-11	6.22E-10
F2RL2	0.135161189	0.009145292	0.017032112
F2RL3	0.076738459	0.140138509	0.192660229
F2R	0.133550557	0.010017326	0.018483392
F2	-0.25520748	6.30E-07	2.56E-06
F3	0.350450275	3.68E-12	3.46E-11

F5	-0.472063617	5.48E-22	2.22E-20
F7	-0.310454375	9.89E-10	6.39E-09
F8A1	-0.163829976	0.001543598	0.003393837
F8	-0.412145643	1.20E-16	2.29E-15
F9	-0.398392407	1.46E-15	2.33E-14
FA2H	0.484523197	3.09E-23	1.51E-21
FAAH2	0.073703349	0.156552017	0.212064274
FAAH	-0.434697085	1.56E-18	3.94E-17
FABP12	0.008116701	0.876179869	0.902392769
FABP1	-0.070870226	0.173146177	0.231380342
FABP2	-0.084379122	0.104660569	0.14959448
FABP3	0.325510609	1.32E-10	9.77E-10
FABP4	-0.382233905	2.36E-14	3.11E-13
FABP5L3	0.082371997	0.113208402	0.160191618
FABP5	0.383815246	1.81E-14	2.43E-13
FABP6	0.364051156	4.54E-13	4.92E-12
FABP7	0.018203468	0.726736983	0.778264483
FABP9	-0.029974712	0.56492967	0.634916444
FADD	0.15371348	0.002993542	0.006198019
FADS1	0.155540312	0.002663575	0.005586833
FADS2	0.037682394	0.469297867	0.544199774
FADS3	0.126778407	0.01454349	0.025770293
FADS6	-0.065013051	0.211542138	0.275555282
FAF1	0.016635106	0.749456861	0.797493835
FAF2	0.323234817	1.81E-10	1.31E-09
FAHD1	-0.222718393	1.49E-05	4.77E-05
FAHD2A	-0.27711646	5.75E-08	2.79E-07
FAHD2B	0.310805207	9.44E-10	6.13E-09
FAH	-0.289114998	1.42E-08	7.65E-08
FAIM2	0.197542645	0.000128192	0.0003468
FAIM3	0.145128525	0.005097886	0.010021718
FAIM	0.392645809	4.00E-15	5.98E-14
FAM100A	-0.211685415	3.95E-05	0.00011753
FAM100B	0.218327234	2.21E-05	6.85E-05
FAM101A	0.328521012	8.74E-11	6.66E-10
FAM101B	-0.089011542	0.086878659	0.126956402
FAM102A	0.154155699	0.002910427	0.006037856
FAM102B	0.367230881	2.75E-13	3.08E-12
FAM103A1	0.15891256	0.002140053	0.004580485
FAM104A	0.257483664	4.96E-07	2.05E-06
FAM104B	0.105888368	0.041509403	0.066113592
FAM105A	0.383460378	1.92E-14	2.56E-13
FAM105B	0.038704517	0.457321571	0.53232605
FAM106A	0.026056006	0.61688815	0.682151739
FAM106C	-0.013384367	0.797223028	0.838026444
FAM107A	-0.365131021	3.83E-13	4.20E-12
FAM107B	-0.267849243	1.63E-07	7.31E-07
FAM108A1	0.162918755	0.001641059	0.003584777
FAM108B1	-0.241140296	2.62E-06	9.60E-06
FAM108C1	0.380567187	3.12E-14	4.04E-13
FAM109A	0.411539281	1.35E-16	2.55E-15
FAM109B	0.294417659	7.45E-09	4.21E-08
FAM10A4	-0.224775911	1.24E-05	4.02E-05
FAM110A	0.45348339	3.23E-20	1.02E-18
FAM110B	-0.019592425	0.706813254	0.7615952
FAM110C	0.039301805	0.450402995	0.526148028
FAM111A	0.235786762	4.41E-06	1.56E-05
FAM111B	0.352378313	2.75E-12	2.64E-11

FAM113A	0.087303945	0.093127565	0.134959434
FAM113B	0.361362552	6.92E-13	7.27E-12
FAM114A1	-0.220400837	1.84E-05	5.78E-05
FAM114A2	-0.078523982	0.131118259	0.181865763
FAM115A	-0.105582377	0.042104909	0.06693377
FAM115C	0.268281873	1.55E-07	6.99E-07
FAM116A	0.343193625	1.08E-11	9.46E-11
FAM116B	0.148720575	0.004093364	0.008216424
FAM117A	-0.195584237	0.000149931	0.000400607
FAM117B	0.390441862	5.85E-15	8.51E-14
FAM118A	0.369064917	2.05E-13	2.35E-12
FAM118B	0.186136865	0.000312511	0.000785793
FAM119A	0.30585904	1.79E-09	1.11E-08
FAM119B	-0.008524879	0.87000696	0.897812932
FAM120AOS	0.08157177	0.116765395	0.164535309
FAM120A	-0.158762885	0.002161135	0.004618055
FAM120B	-0.095262073	0.066824057	0.100815515
FAM120C	-0.18391471	0.000369565	0.000915959
FAM122A	-0.312613647	7.46E-10	4.91E-09
FAM122B	0.237165802	3.86E-06	1.37E-05
FAM122C	-0.052943813	0.309133411	0.381854899
FAM123A	0.073457767	0.157941064	0.213713365
FAM123B	-0.220664269	1.80E-05	5.66E-05
FAM123C	0.177170299	0.000607633	0.001448346
FAM124A	0.300629637	3.46E-09	2.05E-08
FAM124B	-0.033752886	0.516909067	0.589493527
FAM125A	0.170277018	0.000992028	0.002266161
FAM125B	0.156224417	0.002548785	0.005373784
FAM126A	0.008219734	0.874620975	0.901172344
FAM126B	-0.09972577	0.054964257	0.084844098
FAM127A	0.264984321	2.22E-07	9.78E-07
FAM127B	0.348159681	5.18E-12	4.76E-11
FAM127C	0.280251087	4.01E-08	2.00E-07
FAM128A	0.255359993	6.20E-07	2.52E-06
FAM128B	0.182208956	0.000419787	0.001028912
FAM129A	0.171964405	0.000881334	0.002034288
FAM129B	0.339736088	1.78E-11	1.51E-10
FAM129C	0.090373492	0.082140151	0.12082966
FAM131A	0.233362931	5.56E-06	1.93E-05
FAM131B	0.115521353	0.026078286	0.043646798
FAM131C	0.315149498	5.34E-10	3.59E-09
FAM132A	0.114561348	0.027353893	0.04554486
FAM133A	0.068503942	0.187981596	0.248389557
FAM133B	0.070317055	0.176533683	0.235354616
FAM134A	-0.151843701	0.003369328	0.006892972
FAM134B	-0.191058956	0.000214081	0.000555544
FAM134C	-0.174542111	0.000734068	0.001722307
FAM135A	0.210449057	4.39E-05	0.000129753
FAM135B	-0.093176826	0.073043686	0.109010137
FAM136A	0.321736493	2.21E-10	1.58E-09
FAM136B	0.073031504	0.160374117	0.216520274
FAM138B	-0.098997687	0.056769975	0.087313474
FAM138D	0.017085104	0.742914837	0.792097906
FAM138E	-0.095616984	0.06581002	0.099451355
FAM138F	-0.118403595	0.022551555	0.038333795
FAM13AOS	0.02494989	0.631923184	0.696380887
FAM13A	-0.364835437	4.01E-13	4.38E-12
FAM13B	0.205354565	6.76E-05	0.000192642

FAM13C	-0.258927	4.26E-07	1.78E-06
FAM149A	-0.297508712	5.10E-09	2.95E-08
FAM149B1	-0.240294202	2.85E-06	1.04E-05
FAM150A	0.332970708	4.69E-11	3.71E-10
FAM150B	0.110002215	0.034168797	0.055518905
FAM151A	-0.111037461	0.032506101	0.053115694
FAM151B	-0.008628041	0.868448069	0.8965293
FAM153A	0.23131374	6.75E-06	2.30E-05
FAM153B	0.28704428	1.81E-08	9.59E-08
FAM153C	0.171514889	0.000909652	0.002091455
FAM154A	-0.239003725	3.23E-06	1.17E-05
FAM154B	0.06414291	0.217732438	0.282565672
FAM155A	0.155633337	0.002647694	0.005556448
FAM155B	0.370358725	1.66E-13	1.93E-12
FAM156A	0.279777937	4.24E-08	2.11E-07
FAM157A	0.185471681	0.000328664	0.000822879
FAM157B	0.112677094	0.030014047	0.049469666
FAM158A	0.077295059	0.137277025	0.189183238
FAM159A	0.419776455	2.87E-17	5.99E-16
FAM160A1	0.205955201	6.43E-05	0.000183952
FAM160A2	-0.133606079	0.009986079	0.01843769
FAM160B1	-0.355139942	1.81E-12	1.79E-11
FAM160B2	0.123148677	0.017642067	0.030642133
FAM161A	0.04920771	0.344566098	0.418844701
FAM161B	-0.069456748	0.181899403	0.241281038
FAM162A	-0.057546475	0.268899941	0.338773453
FAM162B	-0.071719167	0.168041917	0.225512365
FAM163A	0.184190731	0.000361985	0.000899297
FAM163B	0.006564714	0.899716106	0.921265638
FAM164A	0.411770142	1.29E-16	2.45E-15
FAM164C	-0.108129382	0.03736091	0.060097218
FAM165B	0.027161218	0.602023505	0.668721063
FAM166A	-0.109998183	0.034175411	0.055524592
FAM166B	-0.204611818	7.19E-05	0.000203884
FAM167A	0.222881511	1.47E-05	4.71E-05
FAM167B	-0.192097795	0.000197413	0.000515381
FAM168A	-0.036927467	0.478253146	0.552654244
FAM168B	0.105155059	0.04294857	0.068106457
FAM169A	-0.341962881	1.29E-11	1.12E-10
FAM169B	0.234661023	4.91E-06	1.72E-05
FAM170A	0.007627322	0.883590654	0.908066884
FAM170B	0.040297526	0.439001567	0.514730694
FAM171A1	-0.013067021	0.801930092	0.841420514
FAM171A2	0.34802184	5.29E-12	4.85E-11
FAM171B	0.223457211	1.40E-05	4.48E-05
FAM172A	-0.033532819	0.519647252	0.592073923
FAM173A	-0.070536186	0.175185934	0.233729853
FAM173B	0.068155421	0.190243297	0.250978542
FAM174A	-0.159293246	0.002087278	0.00447704
FAM174B	0.026312054	0.613430161	0.679012484
FAM175A	-0.152200902	0.003294392	0.006750064
FAM175B	-0.081872928	0.115416629	0.162865143
FAM176A	-0.414787012	7.36E-17	1.45E-15
FAM176B	0.272428548	9.78E-08	4.55E-07
FAM177A1	-0.230613843	7.20E-06	2.45E-05
FAM177B	0.208799851	5.06E-05	0.000147409
FAM178A	0.107191768	0.039051745	0.062544325
FAM178B	0.250375427	1.04E-06	4.08E-06

FAM179A	0.0168233	0.746718706	0.795344849
FAM179B	-0.205038635	6.94E-05	0.000197222
FAM180A	-0.16343088	0.001585611	0.003475469
FAM180B	0.148661111	0.004108421	0.008243323
FAM181A	0.107924897	0.037724275	0.060632756
FAM181B	0.207918894	5.45E-05	0.000157836
FAM182A	-0.01858048	0.721310091	0.774043108
FAM182B	0.181421292	0.000445058	0.001086975
FAM183A	0.216740874	2.54E-05	7.80E-05
FAM183B	0.040074048	0.441546016	0.517257422
FAM184A	-0.239434437	3.10E-06	1.12E-05
FAM184B	0.129245371	0.012720523	0.02286962
FAM185A	-0.402873593	6.55E-16	1.11E-14
FAM186A	0.10754013	0.038416122	0.061610529
FAM186B	-0.503382051	3.15E-25	2.17E-23
FAM187B	-0.045166779	0.385678706	0.461942595
FAM188A	-0.190763715	0.000219052	0.00056719
FAM188B	0.249243456	1.16E-06	4.53E-06
FAM189A1	-0.00336025	0.94856832	0.96028142
FAM189A2	0.103591023	0.046158811	0.072685349
FAM189B	0.257530178	4.94E-07	2.05E-06
FAM18A	0.185692037	0.000323229	0.000810289
FAM18B2	-0.043007426	0.408822031	0.485083532
FAM18B	-0.14596431	0.004846148	0.009582625
FAM190A	0.134647631	0.009415718	0.01748182
FAM190B	-0.092899769	0.073904469	0.110088592
FAM192A	0.059283581	0.254690151	0.323510404
FAM193A	0.237438267	3.76E-06	1.34E-05
FAM193B	0.236065662	4.29E-06	1.52E-05
FAM194A	-0.131953927	0.010953795	0.020014812
FAM195A	-0.01753385	0.736409397	0.786255856
FAM195B	0.137516864	0.007991321	0.015068915
FAM196A	0.157617523	0.002328841	0.004945654
FAM196B	0.028916466	0.578753022	0.647784577
FAM197Y2	0.087452959	0.092568325	0.134266194
FAM198A	-0.006382528	0.902485174	0.923199468
FAM198B	-0.13541667	0.009013351	0.016803667
FAM199X	0.15198118	0.003340306	0.006837816
FAM19A1	-0.080534051	0.121507511	0.170325966
FAM19A2	-0.197039414	0.000133476	0.000359875
FAM19A3	0.222860271	1.47E-05	4.72E-05
FAM19A4	0.202456693	8.59E-05	0.000240354
FAM19A5	0.372466732	1.18E-13	1.41E-12
FAM200A	0.018182562	0.727038329	0.778545345
FAM200B	-0.125702524	0.015407846	0.027137752
FAM20A	-0.327475361	1.01E-10	7.60E-10
FAM20B	0.018469613	0.722904533	0.775211237
FAM20C	-0.319552429	2.97E-10	2.08E-09
FAM21A	0.086539441	0.096039276	0.138654573
FAM21B	0.097952128	0.059448816	0.090879321
FAM21C	0.084967715	0.102253142	0.146584666
FAM22A	-0.221228393	1.71E-05	5.40E-05
FAM22D	-0.25777973	4.81E-07	2.00E-06
FAM22F	-0.006333111	0.903236478	0.923873138
FAM22G	0.195704989	0.000148497	0.000397147
FAM23A	0.124154699	0.016730264	0.029218928
FAM24A	0.016779854	0.747350542	0.795847632
FAM24B	0.289088873	1.42E-08	7.67E-08

FAM25A	0.010069091	0.846728077	0.878889009
FAM25B	0.023292094	0.654742099	0.716927124
FAM26D	-0.005008965	0.923397864	0.940102833
FAM26E	-0.023626973	0.65010576	0.712947647
FAM26F	0.218116064	2.25E-05	6.97E-05
FAM27A	0.340794737	1.53E-11	1.31E-10
FAM27B	0.179037176	0.00053043	0.001278397
FAM27C	0.292293656	9.65E-09	5.35E-08
FAM27L	0.07552653	0.14652688	0.200185743
FAM32A	0.091129123	0.079602552	0.117626617
FAM35A	-0.221821395	1.62E-05	5.14E-05
FAM35B2	-0.332213532	5.22E-11	4.10E-10
FAM35B	-0.307457338	1.46E-09	9.17E-09
FAM36A	-0.046549307	0.37128866	0.447074902
FAM38A	0.018519782	0.722182882	0.774636697
FAM38B	-0.020867991	0.688690238	0.745900127
FAM3A	-0.200927029	9.74E-05	0.000269834
FAM3B	0.054345463	0.296482192	0.368466514
FAM3C	0.062718878	0.228141105	0.294005246
FAM3D	0.175147422	0.00070296	0.001655754
FAM40A	-0.083264695	0.10934132	0.155331476
FAM40B	0.222123719	1.57E-05	5.01E-05
FAM41C	0.082537138	0.112485048	0.159281194
FAM43A	-0.156926678	0.002435651	0.005153802
FAM43B	0.181365744	0.000446893	0.001091054
FAM45A	0.02637386	0.612596737	0.678385336
FAM45B	-0.070482599	0.175514812	0.2341216
FAM46A	-0.048561711	0.350944602	0.425637304
FAM46B	0.275317758	7.06E-08	3.37E-07
FAM46C	-0.076250262	0.142685763	0.195689506
FAM46D	0.115304302	0.026362122	0.044074034
FAM47A	0.096367751	0.063706558	0.096653876
FAM47B	0.122239779	0.018502841	0.032014382
FAM47C	-0.201114155	9.60E-05	0.000265922
FAM47E	-0.322791476	1.92E-10	1.38E-09
FAM48A	0.288184913	1.58E-08	8.45E-08
FAM48B1	0.07094358	0.172700635	0.23093121
FAM48B2	0.015497667	0.766072055	0.811532887
FAM49A	0.287526165	1.71E-08	9.09E-08
FAM49B	0.404327991	5.04E-16	8.68E-15
FAM50A	0.23350769	5.48E-06	1.91E-05
FAM50B	-0.079063527	0.128482682	0.17872029
FAM53A	-0.192894166	0.000185467	0.000487066
FAM53B	0.33048172	6.65E-11	5.17E-10
FAM53C	0.203836742	7.67E-05	0.000216278
FAM54A	0.570347802	2.14E-33	6.47E-31
FAM54B	-0.266183763	1.95E-07	8.66E-07
FAM55A	0.13978772	0.0070037	0.013367285
FAM55B	0.054924504	0.29135794	0.363094763
FAM55C	0.264210444	2.42E-07	1.06E-06
FAM55D	0.112008595	0.031009615	0.050883252
FAM57A	0.453375371	3.31E-20	1.03E-18
FAM57B	0.260761843	3.50E-07	1.49E-06
FAM58A	0.124772368	0.016191092	0.028369299
FAM58B	-0.018362498	0.724446149	0.776479055
FAM59A	0.022621067	0.664072207	0.725110798
FAM5B	-0.000725161	0.988893448	0.991531293
FAM5C	0.086709681	0.095384694	0.137829395

FAM60A	0.348758178	4.74E-12	4.39E-11
FAM63A	-0.30538421	1.90E-09	1.17E-08
FAM63B	-0.051504458	0.322489468	0.396337766
FAM64A	0.561576648	3.22E-32	7.21E-30
FAM65A	0.142796337	0.005863769	0.011365831
FAM65B	-0.05442265	0.295795674	0.367750962
FAM65C	-0.163511094	0.001577084	0.003459443
FAM66A	0.079038271	0.128605131	0.178868097
FAM66C	0.155393429	0.002688827	0.005630693
FAM66D	0.052494274	0.313265093	0.386072824
FAM66E	0.098962328	0.056858907	0.087430006
FAM69A	-0.082615079	0.112144915	0.158844721
FAM69B	0.156311935	0.002534429	0.00534578
FAM69C	0.070691616	0.174234617	0.232663183
FAM70A	0.144575413	0.005270895	0.010320485
FAM70B	0.052053409	0.317352067	0.390602241
FAM71A	0.085653341	0.099504398	0.14309728
FAM71C	0.143112877	0.005754093	0.011168457
FAM71D	0.429003454	4.82E-18	1.12E-16
FAM71E1	0.16507508	0.001419	0.003149082
FAM71E2	0.214900128	2.99E-05	9.07E-05
FAM71F1	0.081366366	0.117692363	0.165665743
FAM71F2	0.110966712	0.03261749	0.053280226
FAM72A	0.523956346	1.51E-27	1.47E-25
FAM72B	0.567805334	4.74E-33	1.37E-30
FAM72D	0.567311017	5.53E-33	1.51E-30
FAM73A	-0.082697514	0.111786045	0.158433318
FAM73B	-0.022526482	0.665391562	0.726113644
FAM74A1	0.068645208	0.187070513	0.247300515
FAM74A3	-0.079914754	0.124408213	0.173853845
FAM74A4	0.038886675	0.4552053	0.530491326
FAM75A2	0.1150685	0.026673488	0.0445348
FAM75A3	0.079333409	0.127179836	0.177138822
FAM75A5	0.057251486	0.27136582	0.341275795
FAM75A6	0.016744829	0.74786004	0.796305062
FAM75C1	0.214665452	3.05E-05	9.24E-05
FAM76A	-0.193016528	0.000183693	0.000482851
FAM76B	0.042214196	0.417526221	0.493500883
FAM78A	0.216941678	2.50E-05	7.67E-05
FAM78B	-0.072363294	0.164244797	0.221086813
FAM7A2	0.242308525	2.34E-06	8.62E-06
FAM7A3	0.313101242	7.00E-10	4.62E-09
FAM81A	0.41489251	7.21E-17	1.43E-15
FAM81B	0.225476807	1.16E-05	3.79E-05
FAM82A1	-0.441021893	4.35E-19	1.18E-17
FAM82A2	-0.288668268	1.49E-08	8.02E-08
FAM82B	-0.117826217	0.023222972	0.039370951
FAM83A	0.123702746	0.017134663	0.029854535
FAM83B	0.25233138	8.49E-07	3.37E-06
FAM83C	-0.091546683	0.078227739	0.115801235
FAM83D	0.21616714	2.68E-05	8.17E-05
FAM83E	0.257655015	4.87E-07	2.02E-06
FAM83F	0.220263625	1.86E-05	5.85E-05
FAM83G	0.155718205	0.002633281	0.005529696
FAM83H	0.172128541	0.000871199	0.002012993
FAM84A	0.073106808	0.159942251	0.216010408
FAM84B	0.02835817	0.58610898	0.654216893
FAM86A	0.03269446	0.530146388	0.602107996

FAM86B1	0.0268509	0.606180915	0.67225207
FAM86B2	0.02734684	0.599542821	0.666411431
FAM86C	-0.002236586	0.965753846	0.973129742
FAM86D	0.169321764	0.001060242	0.00240763
FAM89A	0.119936243	0.02085019	0.035680744
FAM89B	0.145046872	0.005123101	0.01006632
FAM8A1	-0.344734165	8.61E-12	7.65E-11
FAM90A1	0.1862953	0.000308774	0.000777281
FAM90A7	0.142416986	0.005997678	0.011606192
FAM91A1	0.168473991	0.001124373	0.002542249
FAM92A1	-0.084593599	0.10377819	0.148453155
FAM92A3	0.03352029	0.519803366	0.592150198
FAM92B	0.194767084	0.000159987	0.000425137
FAM95B1	-0.058248652	0.263092074	0.332571085
FAM96A	-0.027620286	0.595896873	0.662987686
FAM96B	-0.058405625	0.261805603	0.331344338
FAM98A	0.027575052	0.596499296	0.663583816
FAM98B	0.295626402	6.43E-09	3.67E-08
FAM98C	-0.08601499	0.098078348	0.14127083
FAM99A	-0.151190139	0.003510451	0.00715157
FAM99B	-0.097415389	0.060864075	0.092759876
FAM9A	0.063112568	0.225228833	0.290835417
FAM9B	0.015574019	0.764953276	0.810606338
FAM9C	0.021131151	0.684972936	0.742802832
FANCA	0.213417555	3.40E-05	0.000102262
FANCB	0.523455944	1.73E-27	1.67E-25
FANCC	0.084812846	0.10288228	0.14739111
FANCD2	0.535527503	6.38E-29	7.71E-27
FANCE	0.405097489	4.38E-16	7.58E-15
FANCF	0.329290147	7.85E-11	6.03E-10
FANCG	0.438683343	6.99E-19	1.85E-17
FANCI	0.56327541	1.92E-32	4.55E-30
FANCL	0.244726044	1.84E-06	6.90E-06
FANCM	0.1240024	0.016865595	0.029437217
FANK1	0.220100352	1.89E-05	5.92E-05
FAP	0.361415105	6.87E-13	7.22E-12
FAR1	0.23784869	3.61E-06	1.29E-05
FAR2	0.29813216	4.72E-09	2.74E-08
FARP1	0.094309442	0.069609363	0.104495345
FARP2	-0.365230093	3.77E-13	4.14E-12
FARS2	-0.286668459	1.89E-08	9.99E-08
FARSA	0.175267516	0.000696935	0.001642922
FARSB	0.316071767	4.73E-10	3.21E-09
FASLG	0.124709925	0.0162449	0.028448566
FASN	-0.105471811	0.042321852	0.067224998
FASTKD1	0.20571693	6.56E-05	0.000187351
FASTKD2	-0.26411809	2.44E-07	1.07E-06
FASTKD3	0.162680722	0.00166743	0.0036372
FASTKD5	0.086095471	0.097763216	0.14087999
FASTK	-0.04904134	0.346201721	0.42065325
FAS	-0.325110114	1.40E-10	1.03E-09
FAT1	-0.150448275	0.003677116	0.007459828
FAT2	0.249368889	1.15E-06	4.48E-06
FAT3	0.177000089	0.000615167	0.001465076
FAT4	-0.261431766	3.26E-07	1.39E-06
FATE1	-0.028706021	0.581520718	0.650224827
FAU	0.020250889	0.697436469	0.753652057
FBF1	0.278384067	4.98E-08	2.44E-07

FBLIM1	0.461107956	6.25E-21	2.20E-19
FBLL1	0.274749389	7.53E-08	3.57E-07
FBLN1	0.337914996	2.32E-11	1.92E-10
FBLN2	0.023039479	0.658248322	0.72017261
FBLN5	-0.085085908	0.101775047	0.146025385
FBLN7	-0.280175334	4.05E-08	2.02E-07
FBL	0.257059566	5.19E-07	2.14E-06
FBN1	0.136147864	0.008645042	0.016165433
FBN2	0.288462985	1.53E-08	8.19E-08
FBN3	0.060234504	0.247135726	0.315120195
FBP1	-0.208738278	5.08E-05	0.000148051
FBP2	0.060390011	0.245915353	0.313764933
FBRSL1	0.074822536	0.150338348	0.204831116
FBRS	0.187309641	0.000285821	0.000724074
FBXL12	0.07667755	0.140454399	0.193041224
FBXL13	0.131205448	0.011418881	0.020763686
FBXL14	0.050409964	0.332892713	0.406989176
FBXL15	-0.074908776	0.149867435	0.204259359
FBXL16	-0.013448904	0.796266672	0.837153657
FBXL17	-0.174988084	0.000711028	0.001673769
FBXL18	0.282936401	2.94E-08	1.50E-07
FBXL19	0.469425801	9.93E-22	3.89E-20
FBXL20	-0.016221388	0.755487326	0.802325204
FBXL21	0.033664913	0.518002771	0.590571775
FBXL22	-0.028835695	0.579814568	0.648499091
FBXL2	0.348615782	4.84E-12	4.47E-11
FBXL3	-0.194957806	0.000157585	0.00041937
FBXL4	-0.116404241	0.024950691	0.041928947
FBXL5	-0.332189484	5.23E-11	4.12E-10
FBXL6	0.237326512	3.80E-06	1.35E-05
FBXL7	0.227730414	9.43E-06	3.13E-05
FBXL8	-0.273516719	8.65E-08	4.06E-07
FBXO10	0.146687388	0.004637454	0.009206513
FBXO11	0.147610105	0.004382924	0.008745655
FBXO15	-0.102304921	0.048947176	0.076636721
FBXO16	0.190360536	0.000226016	0.000583542
FBXO17	0.170010491	0.001010642	0.002304717
FBXO18	0.273206731	8.96E-08	4.20E-07
FBXO21	0.144124399	0.005415858	0.010569625
FBXO22OS	0.19748063	0.000128832	0.000348249
FBXO22	0.177791781	0.000580846	0.001390724
FBXO24	-0.264665133	2.30E-07	1.01E-06
FBXO25	-0.086903494	0.094643808	0.1368879
FBXO27	0.116438302	0.024908045	0.041882768
FBXO28	-0.059774249	0.250772394	0.318961095
FBXO2	-0.12738824	0.014072708	0.025020659
FBXO30	0.17728108	0.000602776	0.001438316
FBXO31	-0.497383511	1.40E-24	8.41E-23
FBXO32	0.020598211	0.692508827	0.74926198
FBXO33	-0.276016486	6.52E-08	3.13E-07
FBXO34	0.0938633	0.070946057	0.106213823
FBXO36	-0.209098893	4.93E-05	0.000144028
FBXO38	-0.089200613	0.086207995	0.126096599
FBXO39	-0.052147792	0.316474188	0.389690264
FBXO3	-0.251980381	8.80E-07	3.49E-06
FBXO40	-0.056727118	0.275787102	0.346180496
FBXO41	0.391418112	4.94E-15	7.30E-14
FBXO42	-0.076306901	0.142388438	0.195335523

FBXO43	0.391904909	4.54E-15	6.75E-14
FBXO44	-0.056047545	0.281589475	0.352421442
FBXO45	0.29597465	6.16E-09	3.52E-08
FBXO46	0.339969557	1.72E-11	1.46E-10
FBXO47	0.07696014	0.138993418	0.191323628
FBXO48	-0.084204801	0.105382106	0.150528596
FBXO4	-0.057798865	0.266802353	0.336556479
FBXO5	0.464709469	2.84E-21	1.05E-19
FBXO6	0.084479498	0.104246878	0.149099454
FBXO7	-0.460840881	6.63E-21	2.32E-19
FBXO8	-0.320164869	2.74E-10	1.93E-09
FBXO9	-0.079263049	0.127518506	0.177490651
FBXW10	-0.073307073	0.158798002	0.214741637
FBXW11	0.113955563	0.028186123	0.046770277
FBXW12	0.063119288	0.225179356	0.290790376
FBXW2	0.001225041	0.981238406	0.985442936
FBXW4	-0.056576883	0.277062804	0.347524212
FBXW5	-0.078322702	0.132112099	0.183027624
FBXW7	-0.042680547	0.412395794	0.488538325
FBXW8	0.139917252	0.006950807	0.013280328
FBXW9	0.19946121	0.000109792	0.000301071
FCAMR	-0.140835232	0.006586088	0.012650889
FCAR	0.092392944	0.075500476	0.112155729
FCER1A	0.166335439	0.00130234	0.002907353
FCER1G	0.315970329	4.79E-10	3.25E-09
FCER2	0.033810235	0.516196718	0.588815975
FCF1	-0.037325555	0.473519299	0.54837357
FCGBP	0.319774854	2.89E-10	2.02E-09
FCGR1A	0.348417182	4.99E-12	4.59E-11
FCGR1B	0.338173473	2.23E-11	1.86E-10
FCGR1C	0.318282008	3.52E-10	2.43E-09
FCGR2A	0.359699786	8.97E-13	9.23E-12
FCGR2B	0.254930286	6.48E-07	2.63E-06
FCGR2C	0.265972382	2.00E-07	8.85E-07
FCGR3A	0.184084484	0.000364886	0.000905826
FCGR3B	0.071303796	0.1705251	0.228490789
FCGRT	-0.361887162	6.38E-13	6.72E-12
FCHO1	0.43084945	3.35E-18	7.98E-17
FCHO2	0.051678242	0.320857287	0.394477838
FCHSD1	0.336506638	2.84E-11	2.32E-10
FCHSD2	0.158774696	0.002159464	0.004614981
FCN1	0.020904529	0.688173661	0.745546819
FCN2	-0.267704135	1.65E-07	7.42E-07
FCN3	-0.074930757	0.149747587	0.204123943
FCRL1	0.130931643	0.011593328	0.02105591
FCRL2	0.205491009	6.68E-05	0.00019065
FCRL3	0.014625586	0.778884456	0.822307162
FCRL4	0.230214336	7.48E-06	2.53E-05
FCRL5	0.194912392	0.000158154	0.000420827
FCRL6	-0.103226266	0.046935757	0.073812294
FCRLA	0.249980952	1.08E-06	4.23E-06
FCRLB	0.101888943	0.049878647	0.077856491
FDFT1	-0.045760661	0.379456177	0.455612702
FDPSL2A	0.184484386	0.00035408	0.000880976
FDPS	0.026887563	0.60568907	0.671856217
FDX1L	0.160140077	0.001974117	0.004252174
FDX1	-0.302988367	2.57E-09	1.56E-08
FDXACB1	0.013043639	0.802277197	0.841740379

FDXR	-0.320745002	2.53E-10	1.79E-09
FECH	-0.341380011	1.41E-11	1.21E-10
FEM1A	-0.116400764	0.024955048	0.041932732
FEM1B	0.010964735	0.833285753	0.867648501
FEM1C	-0.065385047	0.208934747	0.272568793
FEN1	0.497941336	1.22E-24	7.42E-23
FER1L4	0.369399789	1.94E-13	2.23E-12
FER1L5	0.306906554	1.56E-09	9.79E-09
FER1L6	0.240303399	2.84E-06	1.04E-05
FERMT1	0.138144914	0.00770645	0.014582817
FERMT2	-0.317600032	3.86E-10	2.65E-09
FERMT3	0.336613287	2.79E-11	2.29E-10
FER	0.231805722	6.44E-06	2.21E-05
FES	0.232769342	5.88E-06	2.03E-05
FETUB	-0.282857693	2.97E-08	1.52E-07
FEV	0.152596944	0.003213077	0.006605192
FEZ1	0.190737491	0.000219499	0.000568273
FEZ2	-0.011418633	0.826491632	0.862141351
FEZF1	0.102504994	0.048504371	0.076046319
FEZF2	0.128745855	0.013072471	0.023445244
FFAR1	0.146929263	0.004569475	0.009080607
FFAR2	0.299092382	4.19E-09	2.46E-08
FFAR3	0.136842979	0.00830739	0.015600129
FGA	-0.259190536	4.14E-07	1.74E-06
FGB	-0.186827959	0.000296515	0.000748026
FGD1	0.261075096	3.39E-07	1.45E-06
FGD2	0.164042981	0.001521595	0.003351378
FGD3	0.376240396	6.39E-14	7.96E-13
FGD4	-0.258222097	4.59E-07	1.91E-06
FGD5	-0.102893761	0.047653522	0.074852437
FGD6	0.360266012	8.21E-13	8.53E-12
FGF10	0.019227891	0.712023604	0.765965948
FGF11	0.198124969	0.000122322	0.000332183
FGF12	-0.143170411	0.005734357	0.01113232
FGF13	-0.003864647	0.940861241	0.954467749
FGF14	-0.052314274	0.314929571	0.387908181
FGF16	-0.081428892	0.117409582	0.165349476
FGF17	-0.045744128	0.379628569	0.455709831
FGF18	0.084638874	0.103592678	0.148248928
FGF19	0.077304478	0.137228995	0.18913013
FGF1	0.106491099	0.040357156	0.06446919
FGF20	0.127994655	0.0136179	0.024318376
FGF21	-0.070013514	0.178413288	0.237494957
FGF22	0.058069033	0.264569469	0.334142216
FGF23	0.162454197	0.001692887	0.003690305
FGF2	-0.231802432	6.44E-06	2.21E-05
FGF3	0.022827441	0.661197126	0.722724083
FGF4	0.105467209	0.042330901	0.067234013
FGF5	0.218001886	2.28E-05	7.04E-05
FGF7	-0.002066096	0.968362936	0.975068304
FGF8	0.118978742	0.021899504	0.037308186
FGF9	0.23074011	7.12E-06	2.42E-05
FGFBP1	0.078353517	0.131959574	0.182854465
FGFBP2	-0.283915619	2.62E-08	1.35E-07
FGFBP3	-0.136753929	0.008349979	0.015665156
FGFR1OP2	0.040347035	0.438439019	0.51428298
FGFR1OP	0.028122241	0.589230446	0.656854622
FGFR1	0.231293532	6.76E-06	2.31E-05

FGFR2	0.289977927	1.28E-08	6.95E-08
FGFR3	0.25269204	8.18E-07	3.26E-06
FGFR4	0.186876978	0.000295409	0.000745522
FGFRL1	-0.335180588	3.43E-11	2.77E-10
FGGY	-0.239300033	3.14E-06	1.14E-05
FGG	-0.215985004	2.72E-05	8.29E-05
FGL1	-0.013396603	0.797041679	0.837880023
FGL2	0.063447252	0.222773927	0.288244604
FGR	0.286323583	1.97E-08	1.04E-07
FHAD1	0.28167202	3.41E-08	1.72E-07
FHDC1	0.468640412	1.18E-21	4.58E-20
FHIT	0.037056949	0.476710602	0.551255434
FHL1	-0.08219933	0.11396862	0.161061421
FHL2	0.034325106	0.509824214	0.582714744
FHL3	0.36026942	8.21E-13	8.53E-12
FHL5	-0.213898313	3.26E-05	9.83E-05
FHOD1	0.284718535	2.38E-08	1.24E-07
FHOD3	0.501518395	5.02E-25	3.31E-23
FH	-0.196596805	0.000138293	0.000371904
FIBCD1	0.296011533	6.13E-09	3.51E-08
FIBIN	0.261488406	3.24E-07	1.39E-06
FIBP	0.227468993	9.66E-06	3.20E-05
FICD	-0.0940594	0.07035596	0.105465119
FIG4	0.224650684	1.25E-05	4.06E-05
FIGF	-0.198954756	0.000114392	0.000312309
FIGLA	0.088121799	0.090091226	0.131035803
FIGNL1	0.222862342	1.47E-05	4.72E-05
FIGNL2	0.273058341	9.11E-08	4.26E-07
FIGN	0.180339675	0.00048207	0.001170339
FILIP1L	-0.008125969	0.876039619	0.902309031
FILIP1	-0.067989525	0.19132685	0.252291072
FIP1L1	0.315887938	4.85E-10	3.28E-09
FIS1	-0.282302243	3.16E-08	1.61E-07
FITM1	-0.381420859	2.71E-14	3.54E-13
FITM2	-0.280962441	3.70E-08	1.86E-07
FIZ1	0.253937438	7.19E-07	2.90E-06
FJX1	0.201406623	9.37E-05	0.000260138
FKBP10	0.335288412	3.38E-11	2.74E-10
FKBP11	0.199148487	0.000112612	0.000307954
FKBP14	-0.055888919	0.282955658	0.353853288
FKBP15	0.145945947	0.004851556	0.009588561
FKBP1AP1	0.323469194	1.75E-10	1.27E-09
FKBP1A	0.380337125	3.25E-14	4.19E-13
FKBP1B	0.084708268	0.103308852	0.147917131
FKBP2	-0.01662864	0.749550986	0.797551393
FKBP3	0.145972905	0.004843618	0.009578574
FKBP4	0.152481281	0.003236634	0.006647446
FKBP5	-0.078731236	0.130100966	0.180655987
FKBP6	0.173631961	0.000783247	0.001827362
FKBP7	-0.189376515	0.000243891	0.000625489
FKBP8	0.066247475	0.202979191	0.265861371
FKBP9L	0.114292375	0.027720767	0.046074847
FKBP9	0.227143639	9.95E-06	3.29E-05
FKBPL	0.071294866	0.170578781	0.228547345
FKRP	0.006885655	0.894841056	0.917075133
FKSG29	0.021955398	0.673379353	0.73290338
FKSG83	0.054836514	0.292132765	0.363878263
FKTN	0.054707007	0.293275699	0.365096449

FLAD1	0.163314636	0.001598044	0.003500025
FLCN	0.092380767	0.075539166	0.112196471
FLG2	-0.100521554	0.053045449	0.082219827
FLG	-0.013704631	0.792480115	0.834097044
FLI1	0.043350527	0.405090728	0.481268689
FLII	0.149441456	0.003914765	0.007898517
FLJ10038	-0.275653853	6.80E-08	3.25E-07
FLJ10213	-0.07848404	0.131315019	0.18210064
FLJ10357	-0.056325426	0.279206962	0.349877221
FLJ10661	0.070296093	0.17666301	0.235511275
FLJ11235	-0.016070421	0.757691584	0.804365968
FLJ12825	0.096787558	0.06255468	0.095065553
FLJ13197	-0.141770286	0.006232298	0.012026331
FLJ13224	-0.089857012	0.083911916	0.123181283
FLJ14107	0.084684009	0.103408004	0.148037817
FLJ16779	0.260083187	3.77E-07	1.59E-06
FLJ22536	0.295543478	6.49E-09	3.71E-08
FLJ23867	0.280145809	4.06E-08	2.03E-07
FLJ25328	0.082609409	0.112169632	0.158868435
FLJ25363	0.18569462	0.000323166	0.000810289
FLJ25758	0.044221941	0.39570549	0.472107125
FLJ26850	0.032878856	0.527827942	0.599816797
FLJ30679	0.017523437	0.736560143	0.786374661
FLJ32063	0.058490061	0.261115401	0.330659866
FLJ33360	-0.016609565	0.749828713	0.797761688
FLJ33630	0.01749143	0.737023563	0.786785094
FLJ34503	-0.021883296	0.674390487	0.733683229
FLJ35024	0.085003746	0.102107207	0.146407066
FLJ35220	-0.177027753	0.000613937	0.00146267
FLJ35390	0.079830567	0.124806652	0.174312824
FLJ35776	-0.115594434	0.025983312	0.04351009
FLJ36000	0.080102333	0.123523996	0.172787884
FLJ36031	-0.1089399	0.035949679	0.058070989
FLJ36777	-0.235143588	4.69E-06	1.65E-05
FLJ37201	0.033340622	0.522044739	0.594261773
FLJ37307	-0.15493431	0.00276917	0.005774272
FLJ37453	-0.271934654	1.03E-07	4.78E-07
FLJ37543	0.260493089	3.61E-07	1.53E-06
FLJ39582	-0.344514833	8.89E-12	7.88E-11
FLJ39609	0.079651069	0.125659486	0.17534414
FLJ39653	0.039975379	0.442672098	0.518302294
FLJ39739	0.186932356	0.000294166	0.000742665
FLJ40292	0.132647533	0.010537854	0.019332823
FLJ40330	0.208000937	5.41E-05	0.000156836
FLJ40504	0.074298003	0.153226801	0.208196994
FLJ40852	-0.245028133	1.78E-06	6.70E-06
FLJ41941	0.064496406	0.215202153	0.279774034
FLJ42289	-0.05635327	0.278968987	0.349623815
FLJ42393	0.027005752	0.604104729	0.670472112
FLJ42627	0.187003994	0.000292564	0.000739089
FLJ42709	-0.059490483	0.253032999	0.321631048
FLJ42875	0.147694329	0.004360331	0.008704932
FLJ43390	0.111359285	0.032003496	0.052397571
FLJ43663	-0.040943681	0.431692038	0.507823039
FLJ43859	0.040096067	0.441294942	0.517024102
FLJ43860	0.074825534	0.150321959	0.204822794
FLJ43950	-0.109427528	0.035122655	0.056887279
FLJ44054	0.174749628	0.000723263	0.001698555

FLJ44606	0.337166949	2.58E-11	2.13E-10
FLJ44635	-0.081778215	0.11583949	0.163369271
FLJ45079	0.2297339	7.82E-06	2.64E-05
FLJ45244	-0.265829548	2.03E-07	8.98E-07
FLJ45340	-0.005870349	0.91027608	0.929546367
FLJ45445	0.170913742	0.000948834	0.00217447
FLJ45983	0.16272267	0.001662755	0.003628193
FLJ46111	0.077947555	0.133979925	0.185324741
FLJ46321	0.017573507	0.735835382	0.785853569
FLJ46361	0.018459748	0.723046466	0.775270812
FLJ90757	0.0707935	0.173613123	0.231942102
FLNA	0.236899522	3.96E-06	1.41E-05
FLNB	-0.018306522	0.725252215	0.777092166
FLNC	0.256094095	5.74E-07	2.35E-06
FLOT1	0.217704009	2.34E-05	7.21E-05
FLOT2	0.203454295	7.92E-05	0.000222576
FLRT1	0.228309349	8.93E-06	2.98E-05
FLRT2	0.142374356	0.006012896	0.011631122
FLRT3	-0.121161857	0.019570942	0.033663656
FLT1	-0.169664937	0.001035254	0.00235572
FLT3LG	0.108285563	0.03708538	0.05972635
FLT3	0.036888619	0.478716489	0.552965147
FLT4	-0.116823573	0.024430029	0.041161624
FLVCR1	0.340285981	1.65E-11	1.40E-10
FLVCR2	-0.193585081	0.000175655	0.000463128
FLYWCH1	-0.007966759	0.878449357	0.904284973
FLYWCH2	0.193940738	0.000170795	0.000451451
FMN1	0.344521949	8.88E-12	7.87E-11
FMN2	-0.027212061	0.601343573	0.668040298
FMNL1	0.391917318	4.53E-15	6.74E-14
FMNL2	0.276970895	5.85E-08	2.83E-07
FMNL3	0.297542587	5.08E-09	2.94E-08
FMO1	0.299951153	3.76E-09	2.22E-08
FMO2	-0.217362819	2.41E-05	7.40E-05
FMO3	-0.50370808	2.90E-25	2.01E-23
FMO4	-0.576924636	2.66E-34	1.10E-31
FMO5	-0.411208803	1.43E-16	2.69E-15
FMO6P	-0.050617796	0.33090084	0.404926691
FMO9P	0.155761049	0.002626032	0.005517381
FMOD	0.298051891	4.77E-09	2.77E-08
FMR1NB	0.070226979	0.177089915	0.235969854
FMR1	-0.178988121	0.000532337	0.001282526
FN1	-0.049526724	0.341443539	0.4157085
FN3KRP	0.094398036	0.069346391	0.104171226
FN3K	-0.03082153	0.553983256	0.625117781
FNBP1L	0.12547302	0.015597951	0.027433776
FNBP1	0.317406431	3.96E-10	2.72E-09
FNBP4	0.350821868	3.48E-12	3.29E-11
FNDC1	0.282992492	2.92E-08	1.49E-07
FNDC3A	-0.257490463	4.96E-07	2.05E-06
FNDC3B	0.233296296	5.59E-06	1.94E-05
FNDC4	-0.151535733	0.003435174	0.007015286
FNDC5	-0.37713926	5.51E-14	6.92E-13
FNDC7	0.077521965	0.136123464	0.187840386
FNDC8	0.217845019	2.31E-05	7.13E-05
FNIP1	-0.121802591	0.018929762	0.032670772
FNIP2	-0.023977001	0.64527406	0.708545986
FNTA	0.219513572	1.99E-05	6.22E-05

FNTB	0.14940776	0.003922954	0.007911662
FOLH1B	-0.334265653	3.90E-11	3.13E-10
FOLH1	-0.357435492	1.27E-12	1.28E-11
FOLR1	0.300589702	3.48E-09	2.06E-08
FOLR2	0.029610121	0.569674255	0.639239074
FOLR3	0.075398387	0.147215119	0.201043296
FOLR4	0.194205338	0.000167262	0.000442875
FOSB	-0.135334781	0.009055456	0.016875846
FOSL1	0.121754644	0.018977101	0.032743963
FOSL2	-0.0609477	0.241573415	0.308957154
FOS	0.015652694	0.763800983	0.809643673
FOXA1	0.093515274	0.072003272	0.107618665
FOXA2	-0.315172055	5.33E-10	3.58E-09
FOXA3	0.144292313	0.005361474	0.010476831
FOXB1	0.115192346	0.026509564	0.044294517
FOXC1	0.01998428	0.701227644	0.757132791
FOXC2	0.196748113	0.000136628	0.000367726
FOXD1	0.206374574	6.21E-05	0.000178126
FOXD2	0.307490789	1.45E-09	9.14E-09
FOXD3	0.20539845	6.73E-05	0.000192015
FOXD4L1	0.145508928	0.004981884	0.009823725
FOXD4L2	0.088782925	0.087695194	0.127999389
FOXD4L3	0.120188035	0.02058161	0.035252931
FOXD4L5	-0.001781978	0.972711675	0.978359266
FOXD4L6	0.153866812	0.002964482	0.006142321
FOXD4	0.156628929	0.002483047	0.005245177
FOXE1	0.286516717	1.93E-08	1.02E-07
FOXE3	0.259732648	3.91E-07	1.65E-06
FOXF1	0.094061884	0.070348511	0.105461888
FOXF2	0.319179416	3.13E-10	2.18E-09
FOXG1	0.129242976	0.012722191	0.022870555
FOXH1	0.134632328	0.009423884	0.017495352
FOXI1	0.189490025	0.000241764	0.000620593
FOXI2	-0.082333124	0.113379205	0.160399129
FOXI3	0.286566227	1.92E-08	1.01E-07
FOXJ1	0.391587985	4.80E-15	7.10E-14
FOXJ2	-0.063998798	0.218770062	0.283727437
FOXJ3	0.108150681	0.037323233	0.060051158
FOXK1	0.374632802	8.32E-14	1.01E-12
FOXK2	0.428808835	5.01E-18	1.17E-16
FOXL1	0.338150001	2.24E-11	1.87E-10
FOXL2	0.158406952	0.002212031	0.004717706
FOXM1	0.558905115	7.24E-32	1.55E-29
FOXN1	0.262261412	2.98E-07	1.28E-06
FOXN2	0.118297013	0.022674216	0.038519292
FOXN3	-0.365996323	3.34E-13	3.71E-12
FOXN4	0.133981221	0.009777211	0.018095651
FOXO1	-0.347221786	5.96E-12	5.42E-11
FOXO3B	0.014956632	0.77401355	0.81807512
FOXO3	0.011732589	0.821799735	0.858425667
FOXO4	-0.34918431	4.45E-12	4.14E-11
FOXP1	0.00300162	0.954051069	0.964166053
FOXP2	-0.167669417	0.001188521	0.002674843
FOXP3	0.045090209	0.386485478	0.462769786
FOXP4	0.072715185	0.162197781	0.218760016
FOXQ1	0.25932691	4.08E-07	1.71E-06
FOXR1	-0.047403149	0.362569438	0.438029615
FOXR2	0.066471199	0.201454565	0.264124899

FOXRED1	-0.177289581	0.000602405	0.001437603
FOXRED2	-0.093590821	0.071772696	0.107319921
FOXS1	0.300834646	3.37E-09	2.01E-08
FPGS	-0.135276737	0.009085407	0.016926913
FPGT	-0.139926878	0.006946891	0.013275391
FPR1	0.190492506	0.000223714	0.000578359
FPR2	0.111213531	0.032230298	0.052729901
FPR3	0.207404727	5.69E-05	0.00016436
FRAS1	0.316803395	4.29E-10	2.93E-09
FRAT1	-0.059648518	0.25177228	0.320212421
FRAT2	0.304909063	2.02E-09	1.24E-08
FREM1	0.266245811	1.94E-07	8.61E-07
FREM2	0.163544235	0.001573573	0.003452942
FRG1B	-0.03708997	0.476317643	0.550857008
FRG1	-0.005528075	0.915487348	0.933862721
FRG2B	0.016263883	0.754867217	0.801880382
FRG2C	0.113051299	0.029468798	0.048675682
FRG2	0.060043641	0.248639324	0.316692834
FRK	-0.055318241	0.287907616	0.359401938
FRMD1	0.197035971	0.000133513	0.000359926
FRMD3	-0.241759576	2.47E-06	9.08E-06
FRMD4A	0.210620945	4.33E-05	0.000127934
FRMD4B	-0.081507742	0.117053729	0.164906612
FRMD5	0.286861695	1.85E-08	9.78E-08
FRMD6	0.085213047	0.101262749	0.145374107
FRMD7	-0.310518823	9.80E-10	6.35E-09
FRMD8	0.404240408	5.12E-16	8.81E-15
FRMPD1	0.115888547	0.025604098	0.042921982
FRMPD2L1	0.066742674	0.199615713	0.262007131
FRMPD2	0.125933172	0.015218844	0.026847588
FRMPD4	0.051951932	0.318297694	0.391572598
FRRS1	-0.256115817	5.73E-07	2.34E-06
FRS2	0.07927896	0.127441856	0.177433549
FRS3	0.073289288	0.158899372	0.21483497
FRYL	0.022924218	0.659850601	0.721568979
FRY	-0.10953725	0.034938811	0.05662522
FRZB	0.062299173	0.231275133	0.297601292
FSCB	0.07845471	0.131459644	0.182288511
FSCN1	0.255813699	5.91E-07	2.41E-06
FSCN2	0.232025269	6.31E-06	2.17E-05
FSCN3	-0.018526376	0.722088036	0.774618376
FSD1L	0.429905701	4.04E-18	9.50E-17
FSD1	0.070737591	0.173953967	0.232350716
FSD2	-0.085086246	0.101773682	0.146025385
FSHR	0.000858792	0.986846921	0.990077271
FSIP1	0.2977357	4.96E-09	2.87E-08
FSTL1	0.122328045	0.018417673	0.031883636
FSTL3	0.223901238	1.34E-05	4.32E-05
FSTL4	0.173377531	0.000797529	0.001857856
FSTL5	-0.008283798	0.87365192	0.900454941
FST	-0.130896506	0.011615884	0.021094953
FTCD	-0.38091919	2.95E-14	3.83E-13
FTH1	0.237009971	3.92E-06	1.39E-05
FTHL17	0.077596449	0.135746421	0.187384991
FTHL3	0.21197392	3.86E-05	0.000114904
FTL	0.073748477	0.156297772	0.211763072
FTMT	0.121854252	0.018878872	0.032588587
FTO	-0.24839538	1.27E-06	4.91E-06

FTSJ1	0.398953722	1.32E-15	2.12E-14
FTSJ2	0.116422329	0.024928036	0.041907796
FTSJ3	0.074623633	0.151428746	0.205992727
FTSJD1	-0.137009606	0.008228218	0.015464393
FTSJD2	-0.109175831	0.035547495	0.057505253
FUBP1	0.334111057	3.99E-11	3.20E-10
FUBP3	-0.123692211	0.01714419	0.029868523
FUCA1	-0.343716256	9.99E-12	8.80E-11
FUCA2	0.281396967	3.52E-08	1.78E-07
FUK	-0.119643098	0.021166709	0.03616804
FUNDC1	0.459971925	8.01E-21	2.75E-19
FUNDC2P2	0.025966286	0.618101856	0.683152575
FUNDC2	0.251992542	8.79E-07	3.49E-06
FURIN	0.077946711	0.133984151	0.185324741
FUS	0.487593378	1.49E-23	7.68E-22
FUT10	0.069736377	0.180142301	0.239364699
FUT11	0.063297228	0.22387198	0.289527414
FUT1	0.289595668	1.34E-08	7.25E-08
FUT2	0.338986508	1.99E-11	1.67E-10
FUT3	0.198638225	0.000117358	0.000319969
FUT4	0.437311286	9.23E-19	2.40E-17
FUT5	-0.170620073	0.000968536	0.002216562
FUT6	0.144726842	0.005223012	0.010236384
FUT7	0.324892427	1.44E-10	1.06E-09
FUT8	0.318126113	3.60E-10	2.48E-09
FUT9	0.099115126	0.056475432	0.086927572
FUZ	-0.048231305	0.354235612	0.429080189
FXC1	-0.005729587	0.91241878	0.93144796
FXN	-0.364481595	4.24E-13	4.62E-12
FXR1	-0.022043449	0.672145351	0.731880175
FXR2	-0.125915513	0.015233243	0.026865852
FXYD1	-0.137582404	0.00796116	0.015016307
FXYD2	0.45681611	1.58E-20	5.22E-19
FXYD3	0.419969931	2.77E-17	5.78E-16
FXYD4	0.160720068	0.00189988	0.004106479
FXYD5	0.289925873	1.28E-08	6.99E-08
FXYD6	-0.10394867	0.04540751	0.071629458
FXYD7	0.046673101	0.370016535	0.445810065
FYB	0.227912745	9.27E-06	3.08E-05
FYCO1	-0.248124517	1.31E-06	5.03E-06
FYN	-0.208032593	5.40E-05	0.00015644
FYTTD1	-0.078675551	0.130373692	0.180959012
FZD10	0.240657157	2.75E-06	1.00E-05
FZD1	0.403265192	6.10E-16	1.04E-14
FZD2	0.396349036	2.09E-15	3.25E-14
FZD3	0.115466574	0.026149671	0.043744542
FZD4	-0.370173047	1.71E-13	1.99E-12
FZD5	0.136341509	0.008549772	0.016005315
FZD6	0.17670336	0.000628508	0.001495419
FZD7	0.380683393	3.06E-14	3.97E-13
FZD8	0.172170135	0.000868648	0.002007564
FZD9	0.174234781	0.000750346	0.001756773
FZR1	0.042041962	0.419430423	0.495487007
G0S2	-0.139058798	0.007308116	0.013895045
G2E3	0.234669426	4.91E-06	1.72E-05
G3BP1	0.012307746	0.813220913	0.850936868
G3BP2	-0.108013893	0.037565764	0.060402354
G6PC2	-0.270729927	1.18E-07	5.43E-07

G6PC3	0.358459064	1.09E-12	1.11E-11
G6PC	-0.33449845	3.78E-11	3.03E-10
G6PD	0.502947129	3.51E-25	2.40E-23
GAA	-0.166587884	0.001280061	0.002861464
GAB1	-0.152625668	0.003207251	0.006593895
GAB2	0.065647891	0.20710645	0.27046704
GAB3	0.19119503	0.000211825	0.000550121
GAB4	0.056844099	0.274796545	0.345154579
GABARAPL	-0.424893797	1.07E-17	2.38E-16
GABARAPL	-0.225457443	1.16E-05	3.79E-05
GABARAPL	-0.277984227	5.21E-08	2.55E-07
GABARAP	0.01898014	0.715572416	0.768995235
GABBR1	0.289217339	1.40E-08	7.57E-08
GABBR2	-0.008814838	0.865626653	0.894450943
GABPA	-0.091292078	0.079063717	0.116873738
GABPB1	0.149009657	0.004020878	0.008088854
GABPB2	0.236894131	3.96E-06	1.41E-05
GABRA1	0.008773307	0.866253797	0.894959713
GABRA2	0.1978363	0.000125199	0.000339581
GABRA3	0.085033233	0.1019879	0.146288642
GABRA4	0.17477352	0.000722028	0.001695856
GABRA5	0.119466327	0.021359582	0.036469455
GABRA6	0.137601795	0.007952257	0.015002354
GABRB1	0.205773999	6.53E-05	0.000186486
GABRB2	-0.081396221	0.117557272	0.165498974
GABRB3	0.108429118	0.036833647	0.059368925
GABRD	0.247641818	1.37E-06	5.26E-06
GABRE	0.103637546	0.046060495	0.072555034
GABRG1	0.081575074	0.116750528	0.164525997
GABRG2	0.090505587	0.081691891	0.120267946
GABRG3	0.207367872	5.71E-05	0.000164824
GABRP	0.194461561	0.000163906	0.000434856
GABRQ	0.080760547	0.120459889	0.169047754
GABRR1	0.182916652	0.000398226	0.000980649
GABRR2	0.105686454	0.041901556	0.066642407
GABRR3	0.218074489	2.26E-05	7.00E-05
GAD1	0.238071483	3.54E-06	1.27E-05
GAD2	0.193661797	0.000174596	0.00046058
GADD45A	-0.466180724	2.05E-21	7.65E-20
GADD45B	-0.215355212	2.87E-05	8.73E-05
GADD45GII	0.146156781	0.004789786	0.009480865
GADD45G	-0.286827065	1.86E-08	9.82E-08
GADL1	-0.20775383	5.53E-05	0.00015996
GAGE10	0.096935657	0.062152437	0.094548019
GAGE12D	0.128079389	0.013555391	0.024221628
GAGE12F	-0.032698173	0.53009965	0.602089237
GAGE12J	0.194057563	0.000169227	0.000447721
GAGE13	0.086165636	0.097489137	0.140533907
GAGE1	0.138863415	0.007391697	0.014033869
GAGE2A	0.126947612	0.014411499	0.025563698
GAGE2B	0.108248177	0.037151178	0.059827482
GAGE2C	0.121161849	0.01957095	0.033663656
GAGE2D	0.092049428	0.076598066	0.113625217
GAGE2E	0.123496393	0.017322127	0.030154789
GAGE4	0.129597524	0.01247745	0.022499615
GAGE8	0.164000776	0.001525932	0.003359073
GAK	0.120190869	0.020578604	0.035250812
GAL3ST1	0.386740094	1.10E-14	1.53E-13

GAL3ST2	0.11686613	0.024377716	0.041083907
GAL3ST3	0.164823336	0.001443421	0.003197142
GAL3ST4	0.376128067	6.51E-14	8.09E-13
GALC	0.02088115	0.688504169	0.745739142
GALE	-0.098335538	0.058454594	0.089565638
GALK1	-0.09383909	0.071019189	0.106291359
GALK2	-0.072175292	0.165346352	0.22232924
GALM	-0.006732378	0.89716883	0.918940742
GALNS	0.011197382	0.829801824	0.864834525
GALNT10	0.156613934	0.002485456	0.005249153
GALNT11	-0.126866084	0.014474965	0.025660282
GALNT12	0.310376157	9.99E-10	6.46E-09
GALNT13	0.030625845	0.556503577	0.62707377
GALNT14	-0.000129719	0.998013169	0.998414098
GALNT1	-0.093489917	0.072080802	0.107718381
GALNT2	0.055245859	0.288539837	0.360033235
GALNT3	0.174251903	0.00074943	0.001754836
GALNT4	0.267318212	1.72E-07	7.72E-07
GALNT5	0.272131569	1.01E-07	4.69E-07
GALNT6	0.284604763	2.42E-08	1.26E-07
GALNT7	0.331492111	5.77E-11	4.51E-10
GALNT8	0.026204927	0.614875907	0.680304222
GALNT9	0.003642727	0.944251486	0.95693245
GALNTL1	-0.060091965	0.248258033	0.316308297
GALNTL2	-0.455102078	2.29E-20	7.32E-19
GALNTL4	0.303366876	2.45E-09	1.49E-08
GALNTL5	0.109142954	0.035603309	0.057586191
GALNTL6	0.075207019	0.14824751	0.202300629
GALP	-0.029928484	0.565530206	0.635340481
GALR1	-0.140078871	0.006885313	0.013162768
GALR2	0.155035537	0.00275127	0.005742131
GALR3	-0.366835539	2.92E-13	3.27E-12
GALT	-0.136842774	0.008307488	0.015600129
GAL	0.302158201	2.85E-09	1.72E-08
GAMT	-0.233093977	5.70E-06	1.98E-05
GANAB	-0.041845487	0.421608838	0.497765276
GANC	-0.095007619	0.067558932	0.101847071
GAN	-0.129695898	0.012410283	0.02239066
GAP43	0.289704427	1.32E-08	7.17E-08
GAPDHS	0.103636146	0.046063451	0.072555034
GAPDH	0.303991915	2.26E-09	1.38E-08
GAPT	0.247709201	1.36E-06	5.23E-06
GAPVD1	0.058795727	0.258627281	0.327925961
GAR1	0.133222399	0.010203787	0.018793629
GARNL3	-0.241698179	2.48E-06	9.12E-06
GARS	0.320817518	2.51E-10	1.78E-09
GART	0.201730579	9.12E-05	0.000253674
GAS1	0.108707774	0.036349142	0.058640182
GAS2L1	0.110290089	0.033699333	0.054833821
GAS2L2	0.084355676	0.104757387	0.149706898
GAS2L3	0.260480161	3.61E-07	1.53E-06
GAS2	-0.31511954	5.37E-10	3.60E-09
GAS5	0.233855838	5.30E-06	1.85E-05
GAS6	0.009464338	0.855829992	0.886719633
GAS7	0.356636501	1.44E-12	1.44E-11
GAS8	-0.15348198	0.003037907	0.006279434
GAST	0.231213794	6.81E-06	2.32E-05
GATA1	-0.037670845	0.469434161	0.544262781

GATA2	-0.012042198	0.817179016	0.854495085
GATA3	0.220167732	1.88E-05	5.89E-05
GATA4	0.21341061	3.40E-05	0.000102308
GATA5	0.155505475	0.002669544	0.005597586
GATA6	-0.013086643	0.801638833	0.841204969
GATAD1	-0.133428844	0.010086128	0.018598283
GATAD2A	0.237339126	3.80E-06	1.35E-05
GATAD2B	0.149209748	0.003971387	0.007998178
GATC	0.037801777	0.467890198	0.542756988
GATM	-0.406659681	3.30E-16	5.85E-15
GATSL1	0.07376811	0.156187258	0.211642127
GATSL2	0.062412318	0.230427269	0.296625229
GATSL3	-0.162140825	0.001728691	0.003760946
GATS	0.053504046	0.304034771	0.376677905
GBA2	0.046789488	0.368822995	0.444640951
GBA3	-0.274233002	7.98E-08	3.77E-07
GBAP1	0.08914831	0.086393102	0.126339527
GBAS	0.46822623	1.30E-21	4.99E-20
GBA	0.012505262	0.810279912	0.848527986
GBE1	-0.222576572	1.51E-05	4.83E-05
GBF1	-0.058617831	0.260073356	0.329424047
GBGT1	0.382329336	2.33E-14	3.07E-13
GBP1	-0.100363905	0.053421083	0.082752527
GBP2	0.234518191	4.98E-06	1.74E-05
GBP3	0.038007632	0.465468397	0.540388193
GBP4	0.020202361	0.698125981	0.754233503
GBP5	0.18799001	0.000271328	0.000690344
GBP6	-0.049268453	0.343970145	0.418273407
GBP7	-0.155340836	0.002697921	0.005646391
GBX1	0.042537182	0.413969007	0.490046874
GBX2	0.226401552	1.07E-05	3.51E-05
GCAT	-0.37559046	7.11E-14	8.75E-13
GCA	0.130743891	0.011714306	0.021264
GCC1	-0.301733745	3.01E-09	1.81E-08
GCC2	0.132599414	0.010566252	0.019377784
GCDH	-0.526534065	7.56E-28	7.80E-26
GCET2	0.303625676	2.37E-09	1.45E-08
GCFC1	0.243583915	2.06E-06	7.66E-06
GCGR	-0.271821978	1.05E-07	4.84E-07
GCG	0.191208965	0.000211595	0.000549744
GCH1	-0.009854374	0.84995744	0.881597965
GCHFR	-0.270860733	1.16E-07	5.36E-07
GCKR	-0.362934448	5.41E-13	5.79E-12
GCK	-0.139342926	0.007188078	0.013687717
GCLC	-0.37502936	7.80E-14	9.55E-13
GCLM	-0.071969548	0.166558199	0.223777478
GCM1	0.067237846	0.19629315	0.258101909
GCM2	0.056824797	0.274959823	0.345294351
GCN1L1	0.318561483	3.40E-10	2.35E-09
GCNT1	0.286383608	1.96E-08	1.03E-07
GCNT2	-0.148671687	0.004105739	0.008239602
GCNT3	0.420990266	2.28E-17	4.83E-16
GCNT4	-0.068013487	0.191170065	0.252101015
GCNT7	-0.009095431	0.861391743	0.891109824
GCOM1	0.19487464	0.000158628	0.000421864
GCSH	-0.264289858	2.40E-07	1.05E-06
GC	-0.349270513	4.39E-12	4.09E-11
GDAP1L1	0.027032731	0.603743324	0.670112738

GDAP1	0.35595391	1.60E-12	1.59E-11
GDAP2	0.038268011	0.462415109	0.537329836
GDA	0.056473474	0.277943207	0.348513631
GDE1	-0.151932291	0.0033506	0.006856066
GDEP	-0.022694991	0.663041783	0.724223828
GDF10	0.343311431	1.06E-11	9.30E-11
GDF11	0.152328732	0.003267944	0.006703458
GDF15	0.156986612	0.002426211	0.005136554
GDF1	0.34198259	1.29E-11	1.12E-10
GDF2	-0.073634132	0.15694258	0.212506632
GDF3	0.074038174	0.154673087	0.20982002
GDF5	0.0418651	0.421391091	0.497567171
GDF6	-0.008461131	0.870970517	0.898388456
GDF7	-0.2042282	7.42E-05	0.000210021
GDF9	-0.070952261	0.172647961	0.230900421
GDI1	0.122152861	0.018587043	0.032143321
GDI2	-0.000181477	0.997220422	0.997721236
GDNF	-0.16785235	0.001173647	0.002645552
GDPD1	0.113720979	0.028514185	0.047263466
GDPD2	0.239160339	3.18E-06	1.15E-05
GDPD3	0.35069575	3.55E-12	3.35E-11
GDPD4	-0.115689549	0.02586015	0.043322057
GDPD5	0.182964862	0.000396795	0.000977489
GEFT	0.228230357	9.00E-06	3.00E-05
GEMIN4	-0.028380968	0.585807755	0.654027241
GEMIN5	0.183520149	0.000380658	0.000940994
GEMIN6	0.06956506	0.181217306	0.24055245
GEMIN7	0.274910755	7.39E-08	3.52E-07
GEMIN8P4	-0.081145837	0.118693983	0.166851645
GEMIN8	0.06158455	0.236681226	0.303596895
GEM	0.197682716	0.000126756	0.000343208
GEN1	0.131511494	0.011226643	0.020453331
GET4	0.175011411	0.000709842	0.001671173
GFAP	0.013507902	0.795392659	0.836411325
GFER	0.118119009	0.022880362	0.038833069
GFI1B	0.068249594	0.189630213	0.250236014
GFI1	0.19859004	0.000117816	0.000321173
GFM1	-0.18851572	0.000260602	0.000664497
GFM2	-0.231873366	6.40E-06	2.19E-05
GFOD1	-0.420929252	2.30E-17	4.87E-16
GFOD2	-0.381703068	2.58E-14	3.39E-13
GFPT1	0.237916148	3.59E-06	1.28E-05
GFPT2	0.358406403	1.10E-12	1.12E-11
GFRA1	-0.396192834	2.15E-15	3.33E-14
GFRA2	-0.179114283	0.000527446	0.001271821
GFRA3	0.334525177	3.76E-11	3.02E-10
GFRA4	0.082953773	0.110676223	0.157032385
GFRAL	0.089177743	0.086288896	0.126205666
GGA1	0.1967034	0.000137118	0.000368866
GGA2	0.199416561	0.000110191	0.000302047
GGA3	0.239048806	3.22E-06	1.16E-05
GGCT	0.365453149	3.64E-13	4.01E-12
GGCX	-0.25738056	5.02E-07	2.07E-06
GGH	-0.150499278	0.003665432	0.007440671
GGNBP1	0.093326028	0.072583528	0.108380232
GGNBP2	0.171510528	0.00090993	0.002091811
GGN	0.313603873	6.55E-10	4.34E-09
GGPS1	0.254083841	7.08E-07	2.86E-06

GGT1	0.265909459	2.01E-07	8.91E-07
GGT3P	0.267606217	1.67E-07	7.49E-07
GGT5	0.058663413	0.259702297	0.329016799
GGT6	0.343945513	9.66E-12	8.53E-11
GGT7	-0.117255401	0.023903679	0.040366966
GGT8P	0.219825309	1.94E-05	6.06E-05
GGTA1	0.016287305	0.7545255	0.80160287
GGTLC1	0.278354465	4.99E-08	2.45E-07
GGTLC2	0.262563788	2.89E-07	1.25E-06
GH1	0.084758044	0.103105642	0.147674358
GH2	0.22150891	1.66E-05	5.27E-05
GHDC	-0.141080186	0.006491708	0.012482175
GHITM	-0.228908599	8.45E-06	2.83E-05
GHRHR	0.29131851	1.09E-08	5.96E-08
GHRH	0.03724283	0.474500897	0.549274077
GHRLOS	0.10958723	0.034855339	0.056509445
GHRL	0.193112396	0.000182314	0.000479479
GHR	-0.328884973	8.31E-11	6.35E-10
GHSR	0.11606465	0.025379329	0.042584604
GIF	0.127748138	0.013801202	0.024603808
GIGYF1	-0.032300544	0.535116369	0.606891959
GIGYF2	0.042523454	0.414119834	0.490046874
GIMAP1	-0.039922975	0.44327084	0.518942333
GIMAP2	0.010121683	0.845937486	0.878159897
GIMAP4	0.073175784	0.159547446	0.215535649
GIMAP5	-0.025128373	0.629486626	0.694148214
GIMAP6	-0.066731933	0.199688235	0.262085053
GIMAP7	-0.00612438	0.906410809	0.926479639
GIMAP8	-0.147088293	0.004525271	0.008999047
GIN1	-0.094495577	0.069057804	0.10377684
GINS1	0.582639499	4.18E-35	2.13E-32
GINS2	0.397786843	1.62E-15	2.57E-14
GINS3	0.451645765	4.77E-20	1.45E-18
GINS4	0.464085669	3.25E-21	1.20E-19
GIPC1	0.293102162	8.75E-09	4.90E-08
GIPC2	-0.161486123	0.001805741	0.003919161
GIPC3	-0.050107047	0.335809672	0.409826041
GIPR	0.355152538	1.81E-12	1.78E-11
GIP	0.017080181	0.742986314	0.792098379
GIT1	0.487751886	1.44E-23	7.41E-22
GIT2	0.388955174	7.56E-15	1.08E-13
GIYD2	-0.028865415	0.579423859	0.648335843
GJA10	0.093609636	0.071715361	0.107252716
GJA1	-0.0565764	0.277066914	0.347524212
GJA3	0.374466783	8.55E-14	1.04E-12
GJA4	-0.074471228	0.152268288	0.207078219
GJA5	0.023171902	0.656409383	0.718590953
GJA8	-0.132613167	0.010558129	0.019366452
GJA9	0.206224045	6.28E-05	0.000180156
GJB1	-0.270990162	1.15E-07	5.29E-07
GJB2	-0.039154769	0.452100655	0.527728907
GJB3	0.299569659	3.95E-09	2.32E-08
GJB4	0.047463273	0.361960319	0.437537532
GJB5	-0.061313324	0.238756153	0.30588425
GJB6	0.203132163	8.13E-05	0.000228132
GJB7	0.119344022	0.021493919	0.036683103
GJC1	0.275874414	6.63E-08	3.18E-07
GJC2	0.25437562	6.87E-07	2.78E-06

GJC3	-0.352287807	2.79E-12	2.67E-11
GJD2	0.089743444	0.084305611	0.12363168
GJD3	-0.039219036	0.45135819	0.527140219
GJD4	0.122420052	0.01832926	0.031736095
GK2	0.064137096	0.217774227	0.282601495
GK3P	-0.035844013	0.491266478	0.565331029
GK5	0.175096556	0.000705526	0.001661406
GKAP1	0.058768464	0.258848536	0.328164728
GKN1	0.063913155	0.219388375	0.284344233
GKN2	0.114436293	0.027523943	0.045797377
GK	-0.0367605	0.480246279	0.55453929
GLA	0.329341827	7.79E-11	5.99E-10
GLB1L2	0.289728611	1.31E-08	7.15E-08
GLB1L3	0.160548425	0.001921579	0.004146632
GLB1L	0.148606863	0.0041222	0.008266807
GLB1	0.068982506	0.18490828	0.244701923
GLCCI1	-0.108964816	0.035907023	0.058006789
GLCE	0.01167443	0.822668426	0.859062815
GLDC	0.072248964	0.164914032	0.22182279
GLDN	0.337192947	2.57E-11	2.12E-10
GLE1	-0.058556749	0.26057116	0.33003361
GLG1	0.047419037	0.362408418	0.437861635
GLI1	0.410144865	1.74E-16	3.24E-15
GLI2	0.296411339	5.84E-09	3.35E-08
GLI3	0.184367773	0.000357199	0.000888516
GLI4	0.110834206	0.032826988	0.053582897
GLIPR1L1	0.017510773	0.73674348	0.786528246
GLIPR1L2	-0.301281485	3.19E-09	1.90E-08
GLIPR1	0.19920363	0.00011211	0.000306708
GLIPR2	0.357147461	1.33E-12	1.34E-11
GLIS1	0.252349727	8.47E-07	3.37E-06
GLIS2	0.360185408	8.32E-13	8.62E-12
GLIS3	0.235666595	4.46E-06	1.57E-05
GLMN	0.237919555	3.59E-06	1.28E-05
GLO1	0.037308285	0.473724132	0.548534273
GLOD4	-0.174512128	0.000735642	0.001725389
GLOD5	-0.248135928	1.30E-06	5.03E-06
GLP1R	0.226592938	1.05E-05	3.45E-05
GLP2R	0.119102575	0.021761271	0.037103615
GLRA1	-0.019026361	0.714909879	0.768531977
GLRA2	0.031644584	0.543444115	0.614897124
GLRA3	0.170877017	0.000951277	0.002179568
GLRA4	0.046778632	0.368934219	0.444721215
GLRB	0.306565489	1.63E-09	1.02E-08
GLRX2	0.003723649	0.943015138	0.956019927
GLRX3	0.409162167	2.09E-16	3.81E-15
GLRX5	-0.212547586	3.67E-05	0.000109815
GLRX	-0.022888494	0.660347523	0.721953866
GLS2	-0.05922694	0.255145121	0.323985027
GLS	0.432306055	2.51E-18	6.10E-17
GLT1D1	-0.027733316	0.594392742	0.661979663
GLT25D1	0.417254179	4.63E-17	9.38E-16
GLT25D2	0.078029128	0.133572059	0.184818903
GLT6D1	0.024083215	0.64381085	0.707095195
GLT8D1	-0.299600675	3.93E-09	2.32E-08
GLT8D2	0.107783361	0.037977533	0.060995519
GLTPD1	-0.175920809	0.000664989	0.001574508
GLTPD2	-0.112902671	0.029684338	0.048974856

GLTP	0.435165603	1.42E-18	3.62E-17
GLTSCR1	0.157441458	0.002355649	0.004999386
GLTSCR2	-0.077318672	0.137156632	0.189075418
GLUD1	-0.310883814	9.35E-10	6.07E-09
GLUD2	-0.217718004	2.33E-05	7.20E-05
GLUL	-0.328368195	8.92E-11	6.79E-10
GLYATL1	-0.525404377	1.03E-27	1.02E-25
GLYATL2	0.271920896	1.03E-07	4.79E-07
GLYATL3	-0.034256096	0.510675951	0.5834874
GLYAT	-0.51956115	4.88E-27	4.38E-25
GLYCTK	-0.429700926	4.20E-18	9.88E-17
GLYR1	-0.158893191	0.002142771	0.004584715
GM2A	0.230088977	7.57E-06	2.56E-05
GMCL1L	0.158902424	0.002141475	0.004582435
GMCL1	0.021609993	0.678228494	0.736933082
GMDS	0.034620613	0.506185359	0.579274818
GMEB1	0.292662551	9.23E-09	5.14E-08
GMEB2	0.238270538	3.47E-06	1.24E-05
GMFB	0.193961429	0.000170517	0.000450813
GMFG	0.149908143	0.003802933	0.007688455
GMIP	0.40258128	6.90E-16	1.17E-14
GML	0.058088861	0.264406106	0.333977836
GMNN	0.426621002	7.68E-18	1.73E-16
GMPPA	0.030892856	0.553066011	0.624188821
GMPPB	0.042924595	0.409725886	0.485924461
GMPR2	-0.177223312	0.000605304	0.001443658
GMPR	-0.040418109	0.437632139	0.513427211
GMPS	0.419330522	3.12E-17	6.51E-16
GNA11	-0.141220404	0.00643823	0.01238652
GNA12	0.113482175	0.028851506	0.04777092
GNA13	0.167844894	0.00117425	0.002646312
GNA14	-0.35972366	8.93E-13	9.21E-12
GNA15	0.312773309	7.31E-10	4.81E-09
GNAI1	-0.247724815	1.36E-06	5.22E-06
GNAI2	-0.085344675	0.100734516	0.144720057
GNAI3	0.162144947	0.001728216	0.003760733
GNAL	0.008160395	0.875518709	0.90195903
GNAO1	-0.213595788	3.35E-05	0.000100814
GNAQ	-0.095975684	0.064798026	0.098100636
GNASAS	0.19826944	0.000120905	0.000328874
GNAS	0.332799322	4.80E-11	3.80E-10
GNAT1	-0.015783549	0.761885582	0.808129303
GNAT2	-0.339687307	1.80E-11	1.52E-10
GNAT3	-0.059421248	0.253586689	0.322252601
GNAZ	0.457204833	1.46E-20	4.82E-19
GNB1L	0.072275162	0.164760501	0.221637713
GNB1	0.148986953	0.004026528	0.008099404
GNB2L1	0.209874594	4.62E-05	0.000135576
GNB2	0.114799784	0.027032167	0.045061905
GNB3	0.415015625	7.05E-17	1.40E-15
GNB4	0.236938898	3.95E-06	1.40E-05
GNB5	0.13725391	0.008113354	0.015267237
GNE	-0.471986655	5.58E-22	2.25E-20
GNG10	-0.112810324	0.029818938	0.049168422
GNG11	-0.119101326	0.021762661	0.037103615
GNG12	-0.083993069	0.106263774	0.151630478
GNG13	0.276090421	6.47E-08	3.11E-07
GNG2	0.161466599	0.001808087	0.003923396

GNG3	-0.026192297	0.615046447	0.680455118
GNG4	0.280740128	3.79E-08	1.90E-07
GNG5	0.30472704	2.06E-09	1.27E-08
GNG7	-0.37586501	6.80E-14	8.41E-13
GNG8	0.099078865	0.056566242	0.08704824
GNGT1	0.150987952	0.003555182	0.007235298
GNGT2	0.240318511	2.84E-06	1.04E-05
GNL1	0.109833281	0.034446876	0.055924592
GNL2	0.187106442	0.000290287	0.000734269
GNL3L	0.220754416	1.78E-05	5.62E-05
GNL3	0.333056996	4.63E-11	3.67E-10
GNLY	0.050777847	0.329372147	0.403427722
GNMT	-0.276493717	6.18E-08	2.98E-07
GNPAT	0.027056052	0.603431005	0.669910977
GNPDA1	0.54073422	1.48E-29	1.92E-27
GNPDA2	0.264070738	2.46E-07	1.07E-06
GNPNAT1	-0.199129124	0.000112788	0.000308396
GNPTAB	0.214404778	3.12E-05	9.44E-05
GNPTG	-0.250448301	1.03E-06	4.05E-06
GNRH1	0.263308086	2.67E-07	1.16E-06
GNRH2	-0.089038479	0.086782851	0.126844311
GNRHR2	-0.003071062	0.952989262	0.963532891
GNRHR	-0.013633565	0.793531907	0.834939402
GNS	0.114198799	0.027849389	0.04626933
GOLGA1	-0.213335578	3.43E-05	0.000102914
GOLGA2B	0.051102848	0.326282007	0.400356624
GOLGA2P3	0.040301796	0.438953034	0.514730694
GOLGA2	-0.076052855	0.143725758	0.196831274
GOLGA3	0.21130587	4.08E-05	0.000121175
GOLGA4	-0.196520654	0.000139138	0.000373924
GOLGA5	-0.250474753	1.03E-06	4.04E-06
GOLGA6A	-0.34588404	7.26E-12	6.53E-11
GOLGA6B	-0.381649279	2.61E-14	3.42E-13
GOLGA6C	-0.307574256	1.43E-09	9.05E-09
GOLGA6D	-0.261589236	3.21E-07	1.37E-06
GOLGA6L1(	0.082278754	0.113618436	0.160703336
GOLGA6L1	0.108916636	0.035989545	0.058121248
GOLGA6L5	0.004410551	0.932526169	0.94758965
GOLGA6L6	0.075183767	0.148373323	0.202458448
GOLGA6L9	0.220110498	1.89E-05	5.92E-05
GOLGA7B	0.228352817	8.90E-06	2.97E-05
GOLGA7	0.028692692	0.581696219	0.650384559
GOLGA8A	0.089382715	0.085565995	0.125286327
GOLGA8B	0.156387947	0.002522021	0.005321298
GOLGA8C	0.204892043	7.03E-05	0.000199476
GOLGA8DP	0.181298151	0.000449135	0.001096259
GOLGA8E	0.073051275	0.160260644	0.216381739
GOLGA8F	0.055440373	0.286842971	0.358252502
GOLGA8G	0.200614124	1.00E-04	0.000276127
GOLGA9P	-0.144276505	0.005366573	0.010485766
GOLGB1	-0.194258947	0.000166555	0.000441237
GOLIM4	-0.219652136	1.97E-05	6.15E-05
GOLM1	0.372177926	1.24E-13	1.48E-12
GOLPH3L	0.114826581	0.026996214	0.045013272
GOLPH3	0.167686966	0.001187086	0.002671917
GOLT1A	-0.110011043	0.034154317	0.055503899
GOLT1B	0.195459394	0.000151428	0.000404282
GON4L	0.167516515	0.001201086	0.002701079

GOPC	-0.050298476	0.333964397	0.407916999
GORAB	0.315580679	5.05E-10	3.41E-09
GORASP1	-0.039687863	0.445962765	0.521633995
GORASP2	0.149251334	0.003961171	0.007980022
GOSR1	-0.078436803	0.131548005	0.182360359
GOSR2	0.133079419	0.010285986	0.018928267
GOT1L1	0.033620768	0.518552049	0.590995076
GOT1	-0.065040885	0.211346237	0.275318103
GOT2	-0.391245154	5.09E-15	7.51E-14
GP1BA	0.132906663	0.010386087	0.019087788
GP2	0.270281882	1.24E-07	5.69E-07
GP5	0.002126332	0.967441079	0.974428422
GP6	-0.074053804	0.154585794	0.209748913
GP9	-0.140822408	0.006591062	0.01265855
GPA33	0.136530305	0.00845779	0.015848014
GPAA1	0.150368855	0.003695376	0.007492295
GPAM	-0.358920792	1.01E-12	1.04E-11
GPAT2	0.034770896	0.50434002	0.577731069
GPATCH1	0.18194664	0.000428051	0.00104762
GPATCH2	0.164869946	0.00143887	0.003187853
GPATCH3	-0.048592378	0.350640124	0.425345707
GPATCH4	0.177855337	0.000578169	0.001384908
GPATCH8	0.083322328	0.109095278	0.15509856
GPBAR1	0.274858404	7.44E-08	3.54E-07
GPBP1L1	-0.28398103	2.60E-08	1.34E-07
GPBP1	0.247109944	1.45E-06	5.52E-06
GPC1	0.177824981	0.000579446	0.0013878
GPC2	0.398054357	1.55E-15	2.46E-14
GPC3	0.251611978	9.14E-07	3.62E-06
GPC4	0.367140877	2.78E-13	3.13E-12
GPC5	0.290055566	1.26E-08	6.89E-08
GPC6	0.063969294	0.218982925	0.283911092
GPCPD1	-0.07103062	0.172173066	0.230342611
GPD1L	0.427687302	6.24E-18	1.43E-16
GPD1	-0.32208858	2.11E-10	1.51E-09
GPD2	0.400751286	9.58E-16	1.58E-14
GPER	-0.284520097	2.44E-08	1.27E-07
GPHA2	-0.003186771	0.951220182	0.962183725
GPHN	-0.442716432	3.07E-19	8.50E-18
GPIHBP1	-0.386381583	1.17E-14	1.62E-13
GPI	0.220643334	1.80E-05	5.67E-05
GPKOW	0.130570251	0.011827182	0.021443495
GPLD1	-0.361755051	6.51E-13	6.86E-12
GPM6A	-0.253064424	7.87E-07	3.15E-06
GPM6B	0.151428837	0.0034583	0.007057596
GPN1	0.15001568	0.003777577	0.007641845
GPN2	0.116441267	0.024904336	0.041882768
GPN3	0.071830068	0.167383516	0.224749909
GPNMB	0.245994135	1.62E-06	6.13E-06
GPR101	0.019779504	0.704144591	0.759294644
GPR107	0.232568799	5.99E-06	2.07E-05
GPR108	0.076197387	0.142963755	0.196016787
GPR109A	0.202689954	8.43E-05	0.000236044
GPR109B	0.318653337	3.35E-10	2.32E-09
GPR110	0.015118509	0.771634954	0.816123994
GPR111	0.124646725	0.016299519	0.028536691
GPR112	-0.098367985	0.05837109	0.08945145
GPR113	0.060039228	0.248674167	0.316716964

GPR114	0.311071877	9.12E-10	5.94E-09
GPR115	0.287494205	1.72E-08	9.12E-08
GPR116	-0.164692876	0.001456228	0.003223084
GPR119	0.200557147	0.000100427	0.000277336
GPR120	0.376067029	6.58E-14	8.17E-13
GPR123	0.064104784	0.218006602	0.282884617
GPR124	0.210888372	4.23E-05	0.000125278
GPR125	-0.228296405	8.94E-06	2.98E-05
GPR126	0.065745403	0.206431122	0.269725754
GPR128	-0.212335044	3.74E-05	0.000111731
GPR12	0.113077076	0.029431553	0.048618193
GPR132	0.284444613	2.46E-08	1.28E-07
GPR133	0.257386287	5.01E-07	2.07E-06
GPR135	-0.140752291	0.006618322	0.012703556
GPR137B	0.234181765	5.14E-06	1.79E-05
GPR137C	0.228351616	8.90E-06	2.97E-05
GPR137	0.002117208	0.967580717	0.974428422
GPR141	0.155566956	0.002659018	0.005577861
GPR142	0.098158387	0.058912243	0.090155915
GPR143	0.125243967	0.015789728	0.027744131
GPR144	0.093672344	0.071524552	0.106988191
GPR146	-0.478267483	1.33E-22	5.96E-21
GPR148	0.076030849	0.143842049	0.196963455
GPR149	-0.197718179	0.000126395	0.000342451
GPR150	-0.05076695	0.329476079	0.403530214
GPR151	0.069049056	0.184483846	0.244237585
GPR152	0.118961502	0.021918809	0.037337881
GPR153	-0.018480064	0.722754182	0.77516602
GPR155	-0.399360314	1.23E-15	1.99E-14
GPR156	0.136690102	0.008380625	0.015712292
GPR157	-0.08582678	0.098818455	0.142223758
GPR158	-0.070337739	0.17640614	0.235200316
GPR15	0.065180203	0.210367656	0.274150876
GPR160	0.442254284	3.38E-19	9.25E-18
GPR161	0.321931541	2.16E-10	1.54E-09
GPR162	0.210952904	4.21E-05	0.000124642
GPR171	0.166240645	0.001310798	0.002923286
GPR172A	0.505889001	1.68E-25	1.18E-23
GPR172B	0.327782006	9.68E-11	7.31E-10
GPR173	0.151682935	0.003403557	0.006956104
GPR174	0.100540311	0.053000903	0.082163566
GPR176	-0.041130473	0.429592139	0.505901436
GPR179	0.011556629	0.824428598	0.860630189
GPR17	-0.175734725	0.000673947	0.001593446
GPR180	0.127663877	0.013864352	0.024694564
GPR182	-0.137468328	0.008013722	0.015105437
GPR183	0.232665943	5.94E-06	2.05E-05
GPR18	0.146219994	0.004771404	0.009448898
GPR19	0.397079783	1.84E-15	2.89E-14
GPR1	0.250735367	1.00E-06	3.94E-06
GPR20	0.037415175	0.472457131	0.547227495
GPR21	0.065600761	0.207433421	0.270840779
GPR22	0.062319659	0.231121456	0.297422754
GPR25	0.238750411	3.31E-06	1.19E-05
GPR26	0.038662843	0.457806503	0.532816174
GPR27	0.244169905	1.94E-06	7.26E-06
GPR31	0.073844254	0.155759213	0.211133908
GPR32	0.03090106	0.552960549	0.624140514

GPR34	0.17382011	0.000772837	0.001805402
GPR35	0.341665062	1.35E-11	1.16E-10
GPR37L1	0.219626883	1.97E-05	6.16E-05
GPR37	-0.111664446	0.031533058	0.051682566
GPR39	-0.192069102	0.000197857	0.000516336
GPR3	0.100746839	0.052512474	0.081504621
GPR44	-0.042568926	0.41362036	0.489726899
GPR45	-0.067490114	0.194616051	0.256153606
GPR4	-0.042367886	0.415831317	0.491813802
GPR50	0.149651992	0.003863951	0.007805479
GPR52	0.008152147	0.875643521	0.902040965
GPR55	0.218722194	2.14E-05	6.64E-05
GPR56	0.233330253	5.57E-06	1.94E-05
GPR61	-0.09270396	0.074517791	0.110861282
GPR62	-0.224001265	1.33E-05	4.29E-05
GPR63	0.250798528	9.94E-07	3.92E-06
GPR64	0.1666914	0.001271027	0.00284478
GPR65	0.177743309	0.000582895	0.001395222
GPR68	0.321706884	2.22E-10	1.59E-09
GPR6	0.039905613	0.443469311	0.519144178
GPR75	-0.077905518	0.134190488	0.185571491
GPR77	0.104392017	0.044490457	0.070344357
GPR78	0.055853376	0.283262385	0.354180207
GPR81	0.131104001	0.011483242	0.02086929
GPR82	0.245786902	1.65E-06	6.24E-06
GPR83	0.109288546	0.035356705	0.057224493
GPR84	0.436908474	1.00E-18	2.59E-17
GPR85	0.224944171	1.22E-05	3.96E-05
GPR87	0.147840805	0.004321289	0.008638634
GPR88	-0.056765719	0.275459979	0.345791663
GPR89A	0.074775406	0.150596177	0.205112261
GPR89B	0.041119454	0.429715845	0.506017204
GPR89C	0.117660875	0.023418398	0.039665136
GPR97	0.144221671	0.005384294	0.010516265
GPR98	-0.047849549	0.358062183	0.433425375
GPRASP1	0.069382577	0.182367588	0.24177328
GPRASP2	-0.331779199	5.54E-11	4.35E-10
GPRC5A	0.177312975	0.000601384	0.001435683
GPRC5B	0.160991787	0.001865987	0.004039795
GPRC5C	-0.13256067	0.010589166	0.019414445
GPRC5D	0.356997625	1.36E-12	1.36E-11
GPRC6A	0.178516974	0.000550973	0.001324064
GPRIN1	0.454258582	2.74E-20	8.68E-19
GPRIN2	0.366557741	3.06E-13	3.40E-12
GPRIN3	0.196082385	0.000144095	0.000386153
GPS1	-0.014292595	0.783792729	0.82671609
GPS2	0.158448059	0.002206098	0.004705555
GPSM1	0.272080235	1.02E-07	4.71E-07
GPSM2	0.492939274	4.13E-24	2.31E-22
GPSM3	0.279560682	4.35E-08	2.16E-07
GPT2	-0.387231189	1.01E-14	1.41E-13
GPT	-0.341121259	1.46E-11	1.25E-10
GPX1	0.041453164	0.425978437	0.502358516
GPX2	0.060122155	0.24802003	0.31604548
GPX3	-0.086652047	0.095605899	0.138088938
GPX4	-0.155390501	0.002689332	0.005630696
GPX5	-0.041000927	0.43104785	0.507345816
GPX6	0.01254562	0.809679318	0.848077359

GPX7	0.333289643	4.48E-11	3.57E-10
GPX8	0.306195068	1.71E-09	1.07E-08
GRAMD1A	0.514588011	1.80E-26	1.49E-24
GRAMD1B	0.349428641	4.29E-12	4.00E-11
GRAMD1C	-0.174393189	0.000741915	0.001738873
GRAMD2	0.145736606	0.004913598	0.009698671
GRAMD3	0.012747075	0.806682989	0.845540106
GRAMD4	0.047422789	0.362370402	0.437853709
GRAP2	0.095802615	0.065284695	0.098762373
GRAPL	0.03165083	0.543364509	0.614841972
GRAP	0.02272964	0.662559018	0.723870425
GRASP	0.044448899	0.393282775	0.469666673
GRB10	-0.271012658	1.15E-07	5.28E-07
GRB14	-0.014018054	0.787845901	0.830140479
GRB2	0.243936468	1.99E-06	7.42E-06
GRB7	0.150024692	0.003775459	0.007638336
GREB1L	-0.033543574	0.519513254	0.591955104
GREB1	-0.265789375	2.04E-07	9.02E-07
GREM1	0.240545347	2.78E-06	1.01E-05
GREM2	-0.371954259	1.29E-13	1.52E-12
GRHL1	0.264924938	2.24E-07	9.84E-07
GRHL2	0.275330898	7.05E-08	3.36E-07
GRHL3	-0.158821907	0.002152799	0.004602711
GRHPR	-0.516285923	1.16E-26	9.84E-25
GRIA1	0.049936771	0.337456524	0.411482976
GRIA2	0.138040502	0.007753172	0.014662872
GRIA3	-0.12497491	0.016017633	0.028099972
GRIA4	0.118947328	0.021934692	0.037358547
GRID1	0.147469801	0.004420795	0.00881416
GRID2IP	0.283509434	2.75E-08	1.41E-07
GRID2	0.110146329	0.033933086	0.055180388
GRIK1	0.063295912	0.223881629	0.289527414
GRIK2	0.074415304	0.152577238	0.207356827
GRIK3	0.104253485	0.04477532	0.0707574
GRIK4	0.300307953	3.60E-09	2.13E-08
GRIK5	0.318441187	3.45E-10	2.39E-09
GRIN1	0.356477741	1.47E-12	1.47E-11
GRIN2A	0.276510511	6.17E-08	2.97E-07
GRIN2B	0.047228579	0.364341646	0.439957223
GRIN2C	-0.139490876	0.007126273	0.013580411
GRIN2D	0.382954031	2.09E-14	2.78E-13
GRIN3A	0.048526106	0.351298314	0.426014427
GRIN3B	0.246827793	1.49E-06	5.67E-06
GRINA	-0.079933712	0.124318624	0.173777409
GRINL1A	0.000880182	0.986519343	0.989848318
GRIP1	0.225287714	1.18E-05	3.85E-05
GRIP2	0.208796647	5.06E-05	0.000147427
GRIPAP1	-0.046803932	0.368675038	0.444489477
GRK1	0.058169585	0.263741715	0.333244272
GRK4	0.088748381	0.087819104	0.128142694
GRK5	-0.029836427	0.566727007	0.63643379
GRK6	0.393565164	3.40E-15	5.15E-14
GRK7	0.168728261	0.001104773	0.002502192
GRLF1	-0.267581965	1.67E-07	7.51E-07
GRM1	0.102001862	0.049624343	0.077520282
GRM2	0.07378174	0.156110572	0.211552603
GRM3	0.130690041	0.011749209	0.021319587
GRM4	0.407852717	2.65E-16	4.77E-15

GRM5	0.147847638	0.004319476	0.008636351
GRM6	0.147342074	0.004455528	0.008875416
GRM7	0.142296289	0.006040856	0.011677268
GRM8	0.163036341	0.001628173	0.00355897
GRN	0.223055288	1.45E-05	4.64E-05
GRPEL1	-0.221012339	1.74E-05	5.50E-05
GRPEL2	0.444727198	2.03E-19	5.73E-18
GRPR	-0.223603822	1.38E-05	4.43E-05
GRP	0.188636519	0.000258194	0.000659032
GRRP1	-0.172149113	0.000869936	0.002010309
GRSF1	-0.026933266	0.605076185	0.671325894
GRTP1	-0.124205481	0.01668535	0.029150709
GRWD1	0.053569509	0.303442653	0.376055106
GRXCR2	0.04046681	0.437079757	0.513111545
GSC	0.147495891	0.004413731	0.008800955
GSDMA	0.264572359	2.33E-07	1.02E-06
GSDMB	0.099964639	0.054382331	0.084050023
GSDMC	0.319130758	3.15E-10	2.19E-09
GSDMD	0.157240508	0.002386589	0.005060199
GSG1L	-0.040233438	0.439730395	0.515433569
GSG1	0.045399401	0.383233994	0.459511745
GSG2	0.537765115	3.41E-29	4.25E-27
GSK3A	0.412501149	1.13E-16	2.17E-15
GSK3B	-0.014750895	0.777039677	0.820750991
GSN	-0.006384522	0.902454859	0.923199468
GSPT1	-0.019547896	0.707448997	0.761956522
GSPT2	0.164504281	0.001474926	0.003260123
GSR	0.138107639	0.007723101	0.014612937
GSS	0.017941458	0.730516574	0.78168177
GSTA1	-0.220574748	1.81E-05	5.70E-05
GSTA2	-0.18070234	0.000469353	0.00114183
GSTA3	-0.055497377	0.286346967	0.357677865
GSTA4	-0.042310072	0.416468419	0.492425891
GSTA5	-0.071100799	0.171748575	0.229821004
GSTCD	0.141394789	0.006372272	0.012276221
GSTK1	-0.362708776	5.61E-13	5.98E-12
GSTM1	-0.074559955	0.151779098	0.206427033
GSTM2P1	-0.15275963	0.003180205	0.006549106
GSTM2	-0.082854492	0.111105161	0.157573647
GSTM3	0.06305398	0.225660545	0.291241862
GSTM4	-0.177421854	0.000596656	0.001425078
GSTM5	-0.014090411	0.786777107	0.829156506
GSTO1	-0.239257366	3.15E-06	1.14E-05
GSTO2	-0.027111747	0.602685431	0.669307049
GSTP1	0.278477697	4.92E-08	2.42E-07
GSTT1	-0.244825765	1.82E-06	6.83E-06
GSTT2	-0.017760859	0.733125572	0.783673749
GSTTP1	-0.011029828	0.832310652	0.866905046
GSTTP2	-0.096154887	0.064297256	0.097453586
GSTZ1	-0.430569047	3.54E-18	8.39E-17
GSX1	0.067575026	0.194053909	0.255447468
GSX2	0.077752693	0.134958126	0.186451857
GTDC1	0.261963784	3.08E-07	1.32E-06
GTF2A1L	0.038973837	0.454194612	0.529614578
GTF2A1	-0.185208252	0.000335274	0.000838374
GTF2A2	0.201661243	9.18E-05	0.000255014
GTF2B	-0.103184818	0.047024732	0.073940546
GTF2E1	0.117169759	0.024007283	0.040535052

GTF2E2	0.25991449	3.84E-07	1.62E-06
GTF2F1	0.128526449	0.013229752	0.023682552
GTF2F2	0.113022416	0.029510579	0.048736613
GTF2H1	0.274895315	7.41E-08	3.52E-07
GTF2H2B	0.199078623	0.000113251	0.00030949
GTF2H2C	0.319894702	2.84E-10	1.99E-09
GTF2H2	0.203604751	7.82E-05	0.000220163
GTF2H3	0.005121647	0.921680074	0.938737753
GTF2H4	-0.052139782	0.316548623	0.389757829
GTF2H5	-0.028562265	0.583414871	0.651794026
GTF2IP1	-0.065319369	0.209393404	0.273083154
GTF2IRD1	0.247642434	1.37E-06	5.26E-06
GTF2IRD2B	-0.448366992	9.52E-20	2.81E-18
GTF2IRD2P	-0.488543649	1.19E-23	6.20E-22
GTF2IRD2	-0.47603422	2.22E-22	9.65E-21
GTF2I	-0.160315221	0.001951424	0.004207389
GTF3A	0.089283321	0.085915928	0.125715542
GTF3C1	0.145165588	0.005086478	0.01000324
GTF3C2	0.29877775	4.36E-09	2.55E-08
GTF3C3	0.129323943	0.01266593	0.022785865
GTF3C4	0.022570781	0.664773522	0.725637943
GTF3C5	0.070268298	0.176834608	0.235708488
GTF3C6	0.36018545	8.32E-13	8.62E-12
GTPBP10	-0.355962122	1.60E-12	1.59E-11
GTPBP1	0.197259051	0.000131145	0.000353973
GTPBP2	0.367299009	2.72E-13	3.06E-12
GTPBP3	0.264127552	2.44E-07	1.07E-06
GTPBP4	0.418936339	3.37E-17	6.98E-16
GTPBP5	-0.003027939	0.953648621	0.964003848
GTPBP8	0.110769386	0.032929889	0.053733251
GTSE1	0.612003928	1.69E-39	2.24E-36
GTSF1L	0.179466207	0.000514025	0.001242164
GTSF1	0.144261765	0.005371332	0.010493075
GUCA1A	0.245757309	1.66E-06	6.25E-06
GUCA1B	-0.060487348	0.245153632	0.312953393
GUCA1C	0.033525786	0.519734879	0.592106036
GUCA2A	0.247115342	1.45E-06	5.52E-06
GUCA2B	0.127807752	0.013756678	0.024533214
GUCY1A2	-0.111824087	0.031289317	0.05131685
GUCY1A3	0.184049811	0.000365837	0.000907848
GUCY1B2	0.217445774	2.39E-05	7.36E-05
GUCY1B3	0.212080878	3.82E-05	0.000113917
GUCY2C	0.285023108	2.30E-08	1.20E-07
GUCY2D	0.284440737	2.46E-08	1.28E-07
GUCY2E	-0.061917343	0.23415269	0.300763012
GUCY2F	0.083025971	0.110365113	0.156624432
GUCY2GP	0.020706411	0.690976372	0.747888258
GUF1	0.053380339	0.305155807	0.377714516
GUK1	0.032643229	0.530791443	0.602737524
GULP1	0.233212636	5.64E-06	1.96E-05
GUSBL1	0.138766822	0.007433332	0.014102165
GUSBL2	0.172281269	0.000861865	0.001993507
GUSBP1	0.126719915	0.014589363	0.025842384
GUSBP3	0.390137191	6.17E-15	8.95E-14
GUSB	-0.088923776	0.087191401	0.127366703
GVIN1	-0.010486047	0.840464404	0.873627357
GXYLT1	0.048582506	0.350738127	0.425438685
GXYLT2	0.183917752	0.000369481	0.000915959

GYG1	0.403781106	5.56E-16	9.55E-15
GYG2	-0.094535403	0.068940255	0.103631461
GYLTL1B	0.123267727	0.017531951	0.030469469
GYPA	-0.066307548	0.202568987	0.265411335
GYPB	-0.039590003	0.447085938	0.522824962
GYPC	0.177587527	0.000589526	0.00140974
GYPE	-0.01320006	0.799955893	0.839747144
GYS1	0.19674275	0.000136687	0.000367784
GYS2	-0.487043957	1.70E-23	8.64E-22
GZF1	0.018335895	0.724829195	0.776722459
GZMA	0.112226205	0.030682482	0.050429536
GZMB	0.035168509	0.499474776	0.573187586
GZMH	0.014872741	0.775247072	0.81907468
GZMK	0.061147573	0.240030451	0.307299444
GZMM	0.147256904	0.004478825	0.008916475
H19	0.228695064	8.62E-06	2.88E-05
H1F0	-0.190387697	0.000225541	0.000582552
H1FNT	0.019961205	0.701556116	0.757405352
H1FOO	0.040495607	0.436753311	0.512818969
H1FX	0.087464085	0.09252668	0.134215561
H2AFB1	0.192146761	0.000196659	0.000513747
H2AFJ	-0.05725766	0.27131405	0.341232229
H2AFV	0.080827548	0.120151341	0.168650392
H2AFX	0.476034337	2.22E-22	9.65E-21
H2AFY2	0.372030891	1.27E-13	1.51E-12
H2AFY	1	1.00E-48	1.00E-44
H2AFZ	0.508951808	7.69E-26	5.74E-24
H2BFM	0.027995404	0.590911719	0.658507762
H2BFWT	0.064574043	0.214649275	0.279164568
H2BFXP	0.219601381	1.97E-05	6.17E-05
H3F3A	0.160526502	0.001924367	0.004152198
H3F3B	0.080377577	0.122235391	0.171213771
H3F3C	0.050736731	0.329764418	0.403811765
H6PD	-0.383386919	1.95E-14	2.59E-13
HAAO	-0.44006531	5.28E-19	1.42E-17
HABP2	0.100819224	0.052342176	0.081268866
HABP4	-0.247730309	1.36E-06	5.22E-06
HACE1	0.212821262	3.58E-05	0.000107344
HACL1	-0.309445432	1.13E-09	7.21E-09
HADHA	-0.224969562	1.22E-05	3.95E-05
HADHB	-0.294296722	7.56E-09	4.27E-08
HADH	-0.459972738	8.01E-21	2.75E-19
HAGHL	0.350023188	3.92E-12	3.68E-11
HAGH	-0.422171447	1.82E-17	3.91E-16
HAL	0.141249418	0.006427214	0.012367715
HAMP	0.07636124	0.14210363	0.19499852
HAND1	0.14530641	0.005043342	0.009925265
HAND2	0.144185592	0.005395982	0.010535995
HAO1	-0.525847199	9.10E-28	9.15E-26
HAO2	-0.182090363	0.000423504	0.00103713
HAP1	0.365514527	3.61E-13	3.97E-12
HAPLN1	0.08027131	0.122731654	0.171824316
HAPLN2	-0.068472926	0.188182072	0.248572022
HAPLN3	0.379765986	3.57E-14	4.58E-13
HAPLN4	-0.436783508	1.03E-18	2.64E-17
HAR1A	0.144479403	0.005301459	0.010371763
HAR1B	0.13263182	0.01054712	0.01934804
HARBI1	-0.042158389	0.418142663	0.494141543

HARS2	0.177791055	0.000580876	0.001390724
HARS	0.196934543	0.000134603	0.000362618
HAS1	0.277627193	5.43E-08	2.64E-07
HAS2AS	0.206318906	6.24E-05	0.000178857
HAS2	0.235960543	4.34E-06	1.53E-05
HAS3	-0.02248653	0.66594916	0.726461374
HAT1	0.270823031	1.17E-07	5.38E-07
HAUS1	0.41360547	9.18E-17	1.79E-15
HAUS2	0.2706237	1.20E-07	5.49E-07
HAUS3	0.369190802	2.01E-13	2.30E-12
HAUS4	-0.200167847	0.000103664	0.000285169
HAUS5	0.343414009	1.04E-11	9.17E-11
HAUS6	0.333487296	4.36E-11	3.48E-10
HAUS7	0.168435432	0.001127373	0.002548743
HAUS8	0.365019146	3.90E-13	4.27E-12
HAVCR1	0.418484377	3.67E-17	7.54E-16
HAVCR2	0.328225101	9.10E-11	6.91E-10
HAX1	0.067154454	0.19684986	0.258700634
HBA1	-0.092916598	0.073851947	0.110026809
HBA2	-0.167914326	0.001168646	0.002635474
HBBP1	0.070876556	0.1731077	0.231359963
HBB	-0.217963446	2.28E-05	7.06E-05
HBD	-0.007076926	0.891937537	0.914823875
HBE1	0.018392715	0.724011143	0.77612819
HBEGF	0.094580735	0.068806654	0.10345405
HBG1	-0.071711839	0.168085488	0.225540452
HBG2	-0.016722725	0.748181632	0.796562338
HBM	-0.106507197	0.040326755	0.064425791
HBP1	-0.482359255	5.13E-23	2.42E-21
HBQ1	0.112191099	0.030735057	0.050495118
HBS1L	-0.098726697	0.057454492	0.088223246
HBXIP	0.125127339	0.015888166	0.027899872
HBZ	0.080858206	0.12001036	0.168488117
HCCS	0.200516547	0.00010076	0.000278179
HCFC1R1	0.057439192	0.269794964	0.339707702
HCFC1	0.26186015	3.12E-07	1.34E-06
HCFC2	-0.113363259	0.029020752	0.047995303
HCG11	0.053078009	0.307907008	0.380552355
HCG18	0.33942458	1.87E-11	1.57E-10
HCG22	0.005583433	0.91464424	0.933193821
HCG26	0.09435257	0.069481246	0.104334468
HCG27	0.049899906	0.337813755	0.411842949
HCG2P7	-0.011081013	0.831544052	0.866242449
HCG4P6	-0.16312135	0.001618915	0.003540676
HCG4	0.080827612	0.120151043	0.168650392
HCG9	0.037167631	0.47539417	0.550084373
HCK	0.271238256	1.12E-07	5.15E-07
HCLS1	0.207180388	5.80E-05	0.000167303
HCN1	0.088015267	0.090482181	0.131537216
HCN2	0.048521225	0.351346819	0.426047315
HCN3	-0.032340558	0.534610453	0.606486501
HCN4	0.373370747	1.02E-13	1.23E-12
HCP5	0.103830238	0.045655154	0.071979285
HCRTR1	0.097181747	0.061488761	0.09361705
HCRTR2	0.058751933	0.258982759	0.328314001
HCRT	0.20242426	8.62E-05	0.000240929
HCST	0.172636947	0.000840484	0.001948351
HDAC10	0.06005883	0.248519432	0.316600852

HDAC11	0.336228891	2.95E-11	2.41E-10
HDAC1	0.314829256	5.58E-10	3.73E-09
HDAC2	0.409390912	2.00E-16	3.67E-15
HDAC3	0.109859607	0.034403415	0.055867691
HDAC4	0.127748433	0.013800982	0.024603808
HDAC5	-0.067075397	0.197378691	0.259344301
HDAC6	-0.463223084	3.93E-21	1.43E-19
HDAC7	0.44436572	2.19E-19	6.14E-18
HDAC8	-0.05792143	0.265787786	0.335404084
HDAC9	0.233568803	5.45E-06	1.90E-05
HDC	-0.220464007	1.83E-05	5.75E-05
HDDC2	0.280589869	3.86E-08	1.93E-07
HDDC3	-0.061926941	0.234080051	0.300725013
HDGFL1	-0.034761626	0.504453735	0.577769766
HDGFRP2	0.136125822	0.008655946	0.016184303
HDGFRP3	0.107028086	0.039353458	0.062981972
HDGF	-0.000791236	0.987881516	0.990862792
HDHD1A	0.363684656	4.81E-13	5.19E-12
HDHD2	-0.213628805	3.34E-05	0.000100541
HDHD3	-0.371760636	1.33E-13	1.57E-12
HDLBP	-0.071702826	0.168139092	0.225593243
HDX	-0.0337749	0.516635568	0.58924255
HEATR1	0.222758708	1.49E-05	4.76E-05
HEATR2	0.287644889	1.69E-08	8.97E-08
HEATR3	0.004441565	0.932052853	0.947316817
HEATR4	-0.169043482	0.001080911	0.002452331
HEATR5A	-0.036973527	0.477704116	0.552115879
HEATR5B	-0.048945808	0.347143146	0.421540032
HEATR6	0.301758284	3.00E-09	1.80E-08
HEATR7A	0.233613244	5.43E-06	1.89E-05
HEATR7B2	0.038957737	0.454381207	0.529739139
HEBP1	-0.048672042	0.34984996	0.424542298
HEBP2	0.074976215	0.149499961	0.203816906
HECA	0.021340096	0.682026846	0.740281399
HECTD1	-0.244715896	1.84E-06	6.90E-06
HECTD2	0.245990051	1.62E-06	6.13E-06
HECTD3	0.011872061	0.819717437	0.856663959
HECW1	-0.155415936	0.002684943	0.005624547
HECW2	-0.022491465	0.665880274	0.726447995
HEG1	0.180274827	0.000484378	0.001174938
HELB	0.080508421	0.121626502	0.17048077
HELLS	0.557453119	1.12E-31	2.28E-29
HELQ	-0.312412482	7.66E-10	5.03E-09
HELT	0.160303401	0.001952948	0.004210219
HELZ	0.03010961	0.563178998	0.633306164
HEMGN	-0.096493669	0.063359243	0.096200202
HEMK1	-0.125524587	0.015555058	0.027370418
HEPACAM2	0.083341004	0.10901564	0.155007465
HEPACAM	-0.368070932	2.40E-13	2.73E-12
HEPHL1	0.056446376	0.278174228	0.348759406
HEPH	0.093896082	0.070847129	0.106089636
HEPN1	-0.279667056	4.29E-08	2.13E-07
HERC1	-0.140116521	0.006870135	0.013136274
HERC2P2	0.165053939	0.001421036	0.003152547
HERC2P4	0.086790004	0.095077084	0.137424775
HERC2	-0.007352828	0.887751864	0.911638796
HERC3	-0.264662563	2.30E-07	1.01E-06
HERC4	-0.000637548	0.990235258	0.992427146

HERC5	-0.196059238	0.000144362	0.000386815
HERC6	-0.086923719	0.094566759	0.136806258
HERPUD1	-0.293209203	8.64E-09	4.84E-08
HERPUD2	-0.196961344	0.000134314	0.000361938
HES1	0.032014143	0.538744479	0.610481059
HES2	0.317366705	3.98E-10	2.73E-09
HES3	0.074643953	0.151317076	0.205897055
HES4	0.325601239	1.31E-10	9.66E-10
HES5	-0.122702444	0.018060205	0.031308336
HES6	0.118442725	0.022506666	0.038260755
HES7	0.204787881	7.09E-05	0.000201099
HESRG	0.044119373	0.396803324	0.47304864
HESX1	-0.084667736	0.103474557	0.148111807
HEXA	-0.223071241	1.44E-05	4.63E-05
HEXB	0.121595669	0.019134799	0.032987492
HEXDC	-0.102650975	0.048183402	0.075607257
HEXIM1	0.123260535	0.017538585	0.030478341
HEXIM2	-0.125795304	0.015331573	0.027012965
HEY1	0.226198507	1.09E-05	3.56E-05
HEY2	-0.292204002	9.76E-09	5.41E-08
HEYL	0.241746354	2.47E-06	9.09E-06
HFE2	-0.407456132	2.85E-16	5.12E-15
HFE	-0.17729143	0.000602324	0.001437582
HFM1	0.160118644	0.00197691	0.004257731
HGC6.3	0.094253452	0.069775976	0.104729676
HGD	-0.380778192	3.02E-14	3.91E-13
HGFAC	-0.155987236	0.002588062	0.005447372
HGF	0.119883141	0.02090722	0.035770666
HGSNAT	0.001795849	0.97249934	0.97826166
HGS	0.24272375	2.24E-06	8.29E-06
HHATL	0.004271959	0.934641589	0.949415141
HHAT	-0.22449663	1.27E-05	4.11E-05
HHEX	0.091023241	0.07995426	0.11806751
HHIPL1	0.191416644	0.000208199	0.000541904
HHIPL2	0.0120745	0.81669729	0.854036188
HHIP	0.048656907	0.349999997	0.424698498
HHLA1	0.010027846	0.847348208	0.87948687
HHLA2	0.261228645	3.33E-07	1.42E-06
HHLA3	-0.249127602	1.18E-06	4.58E-06
HIAT1	-0.036978457	0.477645364	0.552091331
HIATL1	0.348414127	4.99E-12	4.59E-11
HIATL2	0.14633762	0.00473737	0.009388031
HIBADH	-0.563449694	1.82E-32	4.40E-30
HIBCH	-0.406236921	3.56E-16	6.28E-15
HIC1	0.046455316	0.372256323	0.448097029
HIC2	0.27357087	8.60E-08	4.04E-07
HIF1AN	0.105769447	0.041739992	0.066443798
HIF1A	0.264858214	2.25E-07	9.91E-07
HIF3A	-0.197557684	0.000128037	0.000346428
HIGD1A	-0.238550786	3.38E-06	1.21E-05
HIGD1B	0.051315785	0.324267585	0.39825281
HIGD1C	-0.061750102	0.235420982	0.302155444
HIGD2A	0.203995211	7.57E-05	0.000213697
HIGD2B	-0.144827623	0.005191361	0.010184859
HILS1	0.211693896	3.95E-05	0.000117486
HINFP	0.191711448	0.000203465	0.000530345
HINT1	0.075763691	0.145259595	0.198645089
HINT2	-0.333838695	4.15E-11	3.32E-10

HINT3	-0.14537612	0.00502211	0.009888366
HIP1R	0.202099383	8.85E-05	0.00024683
HIP1	-0.083940889	0.106481944	0.151881813
HIPK1	-0.008136426	0.875881395	0.902239356
HIPK2	-0.311485154	8.64E-10	5.65E-09
HIPK3	-0.101328686	0.051156546	0.079626589
HIPK4	-0.039161252	0.452025727	0.527703284
HIRA	-0.159904229	0.002005056	0.00431415
HIRIP3	-0.229040275	8.35E-06	2.80E-05
HIST1H1A	-0.012744927	0.806714914	0.845540106
HIST1H1B	0.159702567	0.002031861	0.004368994
HIST1H1C	-0.21741239	2.40E-05	7.37E-05
HIST1H1D	0.219522373	1.99E-05	6.21E-05
HIST1H1E	0.237977969	3.57E-06	1.28E-05
HIST1H1T	0.000707007	0.989171467	0.991760139
HIST1H2AA	-0.009959893	0.848370111	0.880180677
HIST1H2AB	0.13138559	0.011305378	0.020581718
HIST1H2AC	-0.260743969	3.51E-07	1.49E-06
HIST1H2AD	0.125682153	0.015424638	0.027164926
HIST1H2AE	-0.148977632	0.00402885	0.008101621
HIST1H2AG	-0.138184575	0.007688769	0.014552124
HIST1H2AH	0.279845248	4.21E-08	2.09E-07
HIST1H2AJ	0.174808172	0.000720241	0.001692656
HIST1H2AK	-0.229684279	7.86E-06	2.65E-05
HIST1H2AL	0.144702772	0.005230597	0.010249232
HIST1H2AM	-0.035569434	0.494594255	0.568503735
HIST1H2BA	0.010296872	0.843305042	0.875894535
HIST1H2BB	0.073361937	0.158485606	0.214377393
HIST1H2BC	-0.185820985	0.000320087	0.000803323
HIST1H2BD	-0.238829775	3.28E-06	1.19E-05
HIST1H2BE	-0.12938668	0.012622488	0.022717969
HIST1H2BF	0.044561192	0.392087386	0.468435676
HIST1H2BG	0.111299616	0.03209618	0.052527733
HIST1H2BH	0.263141818	2.71E-07	1.18E-06
HIST1H2BI	0.187298403	0.000286066	0.00072451
HIST1H2BJ	-0.045209801	0.385225862	0.461427948
HIST1H2BK	-0.204025432	7.55E-05	0.000213253
HIST1H2BL	-0.096732077	0.062705919	0.095266324
HIST1H2BM	0.208981732	4.98E-05	0.000145405
HIST1H2BN	-0.129438321	0.012586828	0.022672222
HIST1H2BO	0.099774296	0.054845623	0.084695027
HIST1H3A	-0.130158872	0.012098444	0.021883531
HIST1H3B	0.116449269	0.024894328	0.041869548
HIST1H3C	0.219305111	2.03E-05	6.32E-05
HIST1H3D	0.046176511	0.375135853	0.451268429
HIST1H3E	-0.151682461	0.003403658	0.006956104
HIST1H3F	0.127734813	0.013811172	0.024619379
HIST1H3G	0.172457242	0.000851225	0.001972102
HIST1H3H	-0.203054512	8.18E-05	0.00022947
HIST1H3I	0.131157607	0.011449193	0.020813105
HIST1H3J	0.044775153	0.389815819	0.466223344
HIST1H4A	0.226935421	1.01E-05	3.35E-05
HIST1H4B	0.080217793	0.122982169	0.172138746
HIST1H4C	0.079109968	0.128257757	0.178432335
HIST1H4D	0.214670083	3.05E-05	9.24E-05
HIST1H4E	0.205707999	6.56E-05	0.000187437
HIST1H4F	0.079854878	0.124691489	0.174180217
HIST1H4H	0.074265471	0.153407323	0.208391631

HIST1H4I	-0.025289894	0.627285092	0.692073633
HIST1H4J	-0.219823789	1.94E-05	6.06E-05
HIST1H4K	-0.101123815	0.051630606	0.080282933
HIST1H4L	0.036557466	0.482676003	0.556828498
HIST2H2AA	-0.191668656	0.000204146	0.000531911
HIST2H2AB	0.053542407	0.303687701	0.376318118
HIST2H2AC	0.125001961	0.01599459	0.028064491
HIST2H2BA	-0.078782841	0.129848618	0.18036844
HIST2H2BE	-0.164160353	0.001509594	0.003327889
HIST2H2BF	0.00359111	0.94504019	0.957536907
HIST2H3C	0.121360749	0.019369919	0.033361072
HIST2H3D	0.001136418	0.982595455	0.986606857
HIST2H4A	-0.08280174	0.111333606	0.157852686
HIST3H2A	0.342512397	1.19E-11	1.04E-10
HIST3H2BB	0.218743988	2.13E-05	6.63E-05
HIST3H3	0.100254375	0.053683364	0.083098677
HIST4H4	0.051728751	0.320383912	0.393993104
HIVEP1	-0.128685667	0.013115452	0.023507515
HIVEP2	-0.06203974	0.233227544	0.299783344
HIVEP3	0.206497121	6.14E-05	0.000176456
HJURP	0.588740766	5.55E-36	3.69E-33
HK1	0.146254279	0.004761461	0.009430359
HK2	0.375834556	6.83E-14	8.43E-13
HK3	0.212848509	3.57E-05	0.000107123
HKDC1	0.444112054	2.30E-19	6.46E-18
HKR1	0.141599714	0.006295536	0.012140129
HLA-A	0.13056809	0.011828593	0.021444101
HLA-B	0.06592659	0.205180532	0.268368806
HLA-C	0.018579884	0.721318657	0.774043108
HLA-DMA	0.217700849	2.34E-05	7.21E-05
HLA-DMB	0.24032377	2.84E-06	1.04E-05
HLA-DOA	0.181046195	0.000457584	0.001114969
HLA-DOB	0.249780894	1.10E-06	4.31E-06
HLA-DPA1	0.177575082	0.000590059	0.001410534
HLA-DPB1	0.202193264	8.78E-05	0.000245138
HLA-DPB2	0.276089929	6.47E-08	3.11E-07
HLA-DQA1	0.215379521	2.87E-05	8.71E-05
HLA-DQA2	0.193211189	0.000180903	0.000476176
HLA-DQB1	0.191891316	0.000200626	0.000523152
HLA-DQB2	0.298390227	4.57E-09	2.67E-08
HLA-DRA	0.220171702	1.88E-05	5.89E-05
HLA-DRB1	0.177618781	0.00058819	0.001406883
HLA-DRB5	0.092815917	0.074166608	0.110421281
HLA-DRB6	0.148770826	0.00408068	0.008193882
HLA-E	-0.075670628	0.145755885	0.199255387
HLA-F	0.084841751	0.102764625	0.147233143
HLA-G	0.073596895	0.157152998	0.212748168
HLA-H	0.131272047	0.011376801	0.020696616
HLA-J	0.095329291	0.066631028	0.10054714
HLA-L	0.111295401	0.032102735	0.052534145
HLCS	-0.121710237	0.019021037	0.032808407
HLF	-0.523167025	1.87E-27	1.79E-25
HLTF	0.079020414	0.128691761	0.178948646
HLX	-0.305350569	1.91E-09	1.18E-08
HM13	0.289141989	1.41E-08	7.63E-08
HMBOX1	-0.021400024	0.68118276	0.739578339
HMBS	0.010526232	0.839861241	0.873217246
HMCN1	0.054118215	0.29850955	0.370731703

HMG20A	-0.147695652	0.004359977	0.008704932
HMG20B	-0.03249988	0.53259845	0.604501184
HMGA1	0.493670584	3.46E-24	1.94E-22
HMGA2	0.321034641	2.43E-10	1.73E-09
HMGB1	0.07434819	0.152948633	0.207833208
HMGB2	0.477300855	1.66E-22	7.31E-21
HMGB3L1	0.002167478	0.966811404	0.973949069
HMGB3	0.211125769	4.15E-05	0.000122917
HMGB4	-0.015719995	0.762815683	0.80887892
HMGCLL1	-0.088904132	0.087261523	0.127459789
HMGCL	-0.51134702	4.16E-26	3.32E-24
HMGCR	-0.037135316	0.475778313	0.550432959
HMGCS1	-0.142913097	0.005823097	0.011292489
HMGCS2	-0.33612839	2.99E-11	2.44E-10
HMGN1	0.494870099	2.58E-24	1.48E-22
HMGN2	0.181080173	0.000456436	0.001112307
HMGN3	0.127073226	0.014314194	0.02540014
HMGN4	0.394197894	3.05E-15	4.66E-14
HMGN5	-0.184748116	0.000347117	0.00086549
HMGXB3	0.499120288	9.10E-25	5.68E-23
HMGXB4	0.19377591	0.000173032	0.000456877
HMHA1	0.229699546	7.85E-06	2.65E-05
HMHB1	0.015918375	0.759913592	0.806381089
HMMR	0.494242097	3.01E-24	1.71E-22
HMOX1	0.14853246	0.004141166	0.0083015
HMOX2	-0.136770841	0.008341876	0.015657326
HMP19	0.17623128	0.000650287	0.00154355
HMSD	0.258006852	4.70E-07	1.95E-06
HMX1	0.026285409	0.613789621	0.679328712
HMX2	0.225416923	1.17E-05	3.81E-05
HMX3	0.168700395	0.001106905	0.002506737
HN1L	0.164934032	0.001432635	0.003175099
HN1	0.550612905	8.53E-31	1.39E-28
HNF1A	-0.164568239	0.001468561	0.003247854
HNF1B	0.264561834	2.33E-07	1.02E-06
HNF4A	-0.502605326	3.83E-25	2.59E-23
HNF4G	0.184516644	0.000353221	0.000879169
HNMT	-0.413255653	9.79E-17	1.90E-15
HNRNPA0	0.485895167	2.23E-23	1.11E-21
HNRNPA1L	0.296560668	5.73E-09	3.29E-08
HNRNPA1	0.260284478	3.69E-07	1.56E-06
HNRNPA2B	0.357255866	1.31E-12	1.32E-11
HNRNPA3P	0.416966611	4.89E-17	9.86E-16
HNRNPA3	0.447782738	1.08E-19	3.15E-18
HNRNPAB	0.460606038	6.98E-21	2.43E-19
HNRNPCL1	-0.004904068	0.924997287	0.941394429
HNRNPC	0.454311328	2.71E-20	8.60E-19
HNRNPD	0.349846042	4.03E-12	3.78E-11
HNRNPF	0.148983916	0.004027285	0.008100108
HNRNPH1	0.416478502	5.36E-17	1.08E-15
HNRNPH2	-0.09055245	0.081533336	0.120049559
HNRNPH3	0.326636068	1.13E-10	8.47E-10
HNRNPK	0.181464906	0.000443623	0.001083602
HNRNPL	0.460109949	7.77E-21	2.68E-19
HNRNPM	0.366938492	2.88E-13	3.22E-12
HNRNPR	0.21449046	3.10E-05	9.37E-05
HNRNPUL1	0.331987191	5.38E-11	4.23E-10
HNRNPUL2	0.239071951	3.21E-06	1.16E-05

HNRNPU	0.430487109	3.60E-18	8.51E-17
HNRPDL	0.190554612	0.000222639	0.000575802
HNRPLL	0.246252132	1.58E-06	5.98E-06
HOMER1	0.352036616	2.90E-12	2.77E-11
HOMER2	-0.276882403	5.91E-08	2.86E-07
HOMER3	0.396209072	2.14E-15	3.33E-14
HOMEZ	-0.047325525	0.363356803	0.438954231
HOOK1	0.077613133	0.13566208	0.187294522
HOOK2	0.250728065	1.00E-06	3.94E-06
HOOK3	0.195014205	0.000156881	0.000417664
HOPX	0.360911315	7.43E-13	7.77E-12
HORMAD1	0.195045283	0.000156494	0.000416707
HORMAD2	-0.304290853	2.18E-09	1.33E-08
HOTAIR	0.102777152	0.047907408	0.075221578
HOXA10	0.12867715	0.013121545	0.023516118
HOXA11AS	0.066579721	0.200718016	0.26333252
HOXA11	0.095553456	0.065990596	0.099686431
HOXA13	-0.001120748	0.982835406	0.986748323
HOXA1	0.180325354	0.000482579	0.001171288
HOXA2	0.085182255	0.101386634	0.145530984
HOXA3	-0.121161892	0.019570906	0.033663656
HOXA4	-0.028450183	0.584893671	0.653116514
HOXA5	0.091695386	0.077742809	0.115126161
HOXA6	0.192433058	0.000192299	0.00050319
HOXA7	-0.029113476	0.576167629	0.645289606
HOXA9	0.02491938	0.632340084	0.696686161
HOXB13	0.288703256	1.49E-08	7.99E-08
HOXB1	0.024092488	0.643683162	0.707032913
HOXB2	0.163401449	0.00158875	0.003481968
HOXB3	0.186080573	0.000313849	0.000788759
HOXB4	0.160651981	0.001908461	0.004122788
HOXB5	0.125112512	0.015900719	0.027919455
HOXB6	0.112077831	0.030905211	0.05073701
HOXB7	0.173543469	0.000788187	0.001838243
HOXB8	0.135749257	0.008844128	0.016509813
HOXB9	0.209197702	4.89E-05	0.000142886
HOXC10	0.214187308	3.18E-05	9.60E-05
HOXC11	0.189668853	0.000238447	0.00061248
HOXC12	0.065847782	0.205723806	0.268961128
HOXC13	0.254496618	6.78E-07	2.75E-06
HOXC4	0.274211869	8.00E-08	3.78E-07
HOXC5	0.170059139	0.001007221	0.002297441
HOXC6	0.282830667	2.98E-08	1.52E-07
HOXC8	0.129447074	0.012580792	0.022667325
HOXC9	0.220911631	1.76E-05	5.55E-05
HOXD10	-0.02749099	0.597619558	0.664644495
HOXD11	0.272584444	9.61E-08	4.47E-07
HOXD12	0.070066514	0.178084034	0.237120766
HOXD13	0.110381932	0.033550719	0.054625484
HOXD1	0.273399717	8.77E-08	4.11E-07
HOXD3	0.051516247	0.322378578	0.396225926
HOXD4	0.134604522	0.009438738	0.017520201
HOXD8	-0.056330056	0.279167378	0.349850444
HOXD9	-0.053287158	0.306002014	0.378573689
HP1BP3	0.079572061	0.126036303	0.175783761
HPCAL1	0.145450506	0.004999544	0.009850748
HPCAL4	0.27933899	4.46E-08	2.21E-07
HPCA	0.201178853	9.55E-05	0.000264555

HPDL	0.295264333	6.72E-09	3.83E-08
HPD	-0.440641498	4.70E-19	1.27E-17
HPGDS	0.208931524	5.00E-05	0.000146005
HPGD	0.054826026	0.292225214	0.363947907
HPN	-0.322248257	2.07E-10	1.49E-09
HPRT1	0.009656624	0.852933837	0.884357166
HPR	-0.474659956	3.04E-22	1.29E-20
HPS1	0.046595134	0.37081742	0.44661294
HPS3	0.196954824	0.000134385	0.000362079
HPS4	0.027430765	0.59842275	0.665426324
HPS5	-0.045976269	0.377212407	0.453410497
HPS6	-0.058505381	0.260990301	0.330522457
HPSE2	0.125075975	0.01593169	0.02796644
HPSE	0.35149342	3.15E-12	2.99E-11
HPVC1	0.040574951	0.435854604	0.512035348
HPX	-0.387490345	9.71E-15	1.36E-13
HPYR1	0.06012617	0.24798839	0.316025377
HP	-0.345413033	7.79E-12	6.98E-11
HRASLS2	-0.098659603	0.057625021	0.088451011
HRASLS5	0.273282079	8.88E-08	4.16E-07
HRASLS	0.113442604	0.028907731	0.047844131
HRAS	0.333411959	4.40E-11	3.51E-10
HRCT1	-0.027291578	0.60028087	0.667194571
HRC	-0.01839065	0.72404088	0.77612819
HRG	-0.227659092	9.49E-06	3.15E-05
HRH1	0.160875463	0.001880429	0.004068408
HRH2	0.159930584	0.002001577	0.004308061
HRH3	0.012291058	0.813469515	0.851107593
HRH4	-0.172686217	0.000837562	0.001942932
HRK	0.309444584	1.13E-09	7.21E-09
HRNBP3	0.121021641	0.019713749	0.033885876
HRNR	0.059135684	0.255879314	0.324751716
HRSP12	-0.320205566	2.72E-10	1.92E-09
HR	0.166474144	0.001290055	0.002881542
HS1BP3	-0.062958821	0.226362993	0.291921513
HS2ST1	0.096125277	0.064379778	0.097548976
HS3ST1	0.218217621	2.23E-05	6.92E-05
HS3ST2	-0.073681733	0.156673905	0.212186101
HS3ST3A1	-0.245089894	1.77E-06	6.66E-06
HS3ST3B1	-0.35592218	1.61E-12	1.59E-11
HS3ST4	-0.034224817	0.51106226	0.583794859
HS3ST5	0.18571511	0.000322665	0.000809383
HS3ST6	0.315687654	4.98E-10	3.36E-09
HS6ST1	-0.128486132	0.013258834	0.023732479
HS6ST2	0.336318353	2.91E-11	2.38E-10
HS6ST3	0.160212692	0.001964679	0.004232761
HSBP1L1	0.071599549	0.168754231	0.226361554
HSBP1	0.1167237	0.024553179	0.04134464
HSCB	-0.154860456	0.002782295	0.005799821
HSD11B1L	-0.03551372	0.49527095	0.5691502
HSD11B1	-0.461948721	5.20E-21	1.85E-19
HSD11B2	0.049679943	0.339950251	0.414168841
HSD17B10	-0.296983446	5.44E-09	3.13E-08
HSD17B11	-0.27948924	4.38E-08	2.17E-07
HSD17B12	-0.033350283	0.5219241	0.594158395
HSD17B13	-0.247866416	1.34E-06	5.15E-06
HSD17B14	-0.050294807	0.333999703	0.407916999
HSD17B1	0.151272771	0.003492317	0.007118996

HSD17B2	-0.087564894	0.092150014	0.133731906
HSD17B3	-0.085156982	0.101488404	0.145656076
HSD17B4	-0.422761575	1.62E-17	3.51E-16
HSD17B6	-0.460242551	7.55E-21	2.61E-19
HSD17B7P2	-0.014493102	0.780836226	0.823974749
HSD17B7	-0.077416264	0.136659905	0.188463147
HSD17B8	-0.451399097	5.03E-20	1.53E-18
HSD3B1	-0.104999846	0.043258512	0.068537079
HSD3B2	-0.087396273	0.092780746	0.134495963
HSD3B7	-0.05636738	0.278848447	0.349516153
HSDL1	-0.060329551	0.246389328	0.314289166
HSDL2	-0.462589668	4.52E-21	1.62E-19
HSF1	0.250880931	9.86E-07	3.89E-06
HSF2BP	0.44008689	5.26E-19	1.41E-17
HSF2	0.306867748	1.57E-09	9.84E-09
HSF4	-0.020296944	0.69678233	0.753067736
HSF5	-0.044773079	0.38983779	0.466223344
HSFX2	-0.02214697	0.670695683	0.730940886
HSFY2	-0.022602922	0.664325238	0.725347331
HSFYL1	-0.048973117	0.346873868	0.421304339
HSH2D	0.345959969	7.18E-12	6.46E-11
HSN2	0.20763745	5.58E-05	0.000161422
HSP90AA1	0.231875688	6.40E-06	2.19E-05
HSP90AB1	0.257245831	5.09E-07	2.10E-06
HSP90AB2P	0.144394411	0.005328648	0.010420861
HSP90AB4P	0.17394008	0.000766267	0.001791523
HSP90B1	0.203208265	8.08E-05	0.000226798
HSP90B3P	0.122736166	0.018028306	0.031258478
HSPA12A	0.322502546	2.00E-10	1.44E-09
HSPA12B	-0.127678645	0.013853266	0.02468337
HSPA13	0.179884048	0.000498501	0.001207162
HSPA14	0.473567745	3.90E-22	1.62E-20
HSPA1A	-0.000969716	0.985148215	0.988671741
HSPA1B	0.089208469	0.086180216	0.126073796
HSPA1L	-0.050718049	0.32994276	0.403952782
HSPA2	0.2314805	6.64E-06	2.27E-05
HSPA4L	0.110289248	0.033700698	0.054833821
HSPA4	0.41784623	4.14E-17	8.42E-16
HSPA5	0.146770929	0.004613872	0.009161524
HSPA6	0.393598393	3.39E-15	5.13E-14
HSPA7	0.366555339	3.06E-13	3.40E-12
HSPA8	0.152627061	0.003206968	0.006593895
HSPA9	0.110647266	0.0331245	0.054015412
HSPB11	0.189999549	0.000232426	0.000598707
HSPB1	0.260256953	3.70E-07	1.56E-06
HSPB2	0.155372054	0.00269252	0.005635678
HSPB3	0.189464559	0.00024224	0.000621654
HSPB6	0.071420594	0.169824101	0.227704654
HSPB7	0.236231589	4.22E-06	1.49E-05
HSPB8	0.184795244	0.000345886	0.000862637
HSPB9	-0.194411695	0.000164554	0.000436285
HSPBAP1	0.200726001	9.91E-05	0.000273802
HSPBP1	0.207703516	5.55E-05	0.000160618
HSPC072	0.122922668	0.017852778	0.030981102
HSPC157	-0.251911277	8.87E-07	3.51E-06
HSPC159	0.246657208	1.51E-06	5.77E-06
HSPD1	0.207886474	5.46E-05	0.000158223
HSPE1	0.262489927	2.91E-07	1.26E-06

HSPG2	0.084329922	0.104863818	0.149831239
HSPH1	0.290162968	1.25E-08	6.81E-08
HTATIP2	0.065565991	0.20767489	0.271138289
HTATSF1	0.130412772	0.011930382	0.021612901
HTA	0.059179995	0.255522633	0.324361023
HTN1	0.110077643	0.034045253	0.055340204
HTN3	0.101897664	0.049858968	0.077831873
HTR1A	0.074098485	0.154336458	0.209487462
HTR1B	0.052626654	0.312044656	0.384878259
HTR1D	0.197426742	0.000129391	0.000349665
HTR1E	-0.021734855	0.676474006	0.735427839
HTR1F	0.173500594	0.000790591	0.001843202
HTR2A	0.007348132	0.887823085	0.91166494
HTR2B	-0.201194644	9.53E-05	0.000264287
HTR2C	0.054813563	0.292335097	0.364061999
HTR3A	0.383742502	1.83E-14	2.45E-13
HTR3B	0.204119836	7.49E-05	0.000211829
HTR3C	-0.056206952	0.280221065	0.350883975
HTR3D	0.060785939	0.242827253	0.310381402
HTR3E	0.11468941	0.027180689	0.045286752
HTR4	0.101497318	0.050769071	0.079091448
HTR5A	0.081236016	0.118283597	0.166368669
HTR6	0.125611063	0.015483362	0.027258707
HTR7P1	-0.096418766	0.063565657	0.096469492
HTR7	0.044665598	0.390977935	0.467418532
HTRA1	-0.057297257	0.270982199	0.34087942
HTRA2	0.165006254	0.001425638	0.003161702
HTRA3	0.389644	6.71E-15	9.71E-14
HTRA4	0.274666735	7.60E-08	3.60E-07
HTT	0.077848022	0.134478895	0.185938005
HULC	-0.403182427	6.20E-16	1.06E-14
HUNK	0.351470946	3.16E-12	3.00E-11
HUS1B	0.21583008	2.76E-05	8.40E-05
HUS1	0.150476736	0.003670592	0.007449627
HUWE1	0.119944247	0.020841606	0.035670659
HVCN1	0.260985608	3.42E-07	1.46E-06
HYAL1	-0.462639848	4.47E-21	1.60E-19
HYAL2	0.107599587	0.038308517	0.061462695
HYAL3	-0.112615837	0.030104121	0.049601712
HYAL4	0.315794516	4.91E-10	3.32E-09
HYDIN	0.119646196	0.021163342	0.036165389
HYI	-0.232986272	5.76E-06	1.99E-05
HYLS1	0.133441307	0.010079064	0.018590419
HYMAI	0.209698656	4.69E-05	0.000137403
HYOU1	0.152685358	0.003195174	0.006570424
IAH1	0.07776801	0.134881036	0.186371203
IAPP	-0.217454836	2.39E-05	7.35E-05
IARS2	-0.136190876	0.008623799	0.016133283
IARS	0.347290211	5.90E-12	5.37E-11
IBSP	0.253607608	7.44E-07	2.99E-06
IBTK	-0.038957846	0.454379944	0.529739139
ICA1L	0.282495327	3.09E-08	1.58E-07
ICA1	0.160602279	0.001914747	0.004135023
ICAM1	0.236605409	4.07E-06	1.44E-05
ICAM2	0.113345034	0.029046767	0.048034339
ICAM3	-0.22184842	1.61E-05	5.12E-05
ICAM4	0.263926196	2.49E-07	1.09E-06
ICAM5	0.179765721	0.000502852	0.001216789

ICK	-0.245002329	1.79E-06	6.72E-06
ICMT	0.233974083	5.24E-06	1.83E-05
ICOSLG	-0.136809154	0.008323545	0.015625863
ICOS	0.251153254	9.58E-07	3.79E-06
ICT1	0.0246854	0.635541176	0.699825962
ID1	0.124603156	0.016337266	0.028597752
ID2B	0.02409416	0.643660143	0.707032913
ID2	-0.017046698	0.743472465	0.792395595
ID3	0.103128183	0.047146536	0.074120366
ID4	0.172068023	0.000874923	0.002020895
IDE	-0.034168247	0.511761285	0.584492824
IDH1	-0.318976686	3.21E-10	2.23E-09
IDH2	-0.115672806	0.025881794	0.043354671
IDH3A	0.131165147	0.011444411	0.02080821
IDH3B	0.031624988	0.543693879	0.61514479
IDH3G	0.097279096	0.061227839	0.093255429
IDI1	-0.085262059	0.101065805	0.145122745
IDI2	0.317789461	3.76E-10	2.59E-09
IDO1	0.008251341	0.874142845	0.900821113
IDO2	-0.132754379	0.01047504	0.019221124
IDS	-0.090056687	0.083223321	0.122278561
IDUA	0.023123497	0.657081338	0.719172312
IER2	-0.017203091	0.741202596	0.790711753
IER3IP1	0.205569223	6.64E-05	0.000189543
IER3	0.343715296	1.00E-11	8.80E-11
IER5L	0.237130289	3.87E-06	1.38E-05
IER5	0.333747516	4.20E-11	3.36E-10
IFFO1	-0.13440048	0.009548376	0.017706669
IFFO2	0.283034277	2.91E-08	1.49E-07
IFI16	0.229540832	7.97E-06	2.68E-05
IFI27L1	0.005593992	0.914483444	0.93307755
IFI27L2	0.376815434	5.81E-14	7.27E-13
IFI27	-0.041042262	0.43058306	0.506918551
IFI30	0.474326025	3.28E-22	1.38E-20
IFI35	0.137645164	0.007932374	0.01496768
IFI44L	0.010800002	0.83575465	0.869991856
IFI44	-0.075076555	0.148954486	0.203140147
IFI6	0.067837713	0.192322372	0.253419293
IFIH1	0.01956058	0.707267887	0.761879034
IFIT1B	-0.188938624	0.000252263	0.000645132
IFIT1	-0.331334193	5.90E-11	4.61E-10
IFIT2	-0.165777143	0.001352884	0.003011077
IFIT3	-0.071414841	0.16985858	0.227735557
IFIT5	-0.179632013	0.000507812	0.001227894
IFITM1	0.083419191	0.108682742	0.154600328
IFITM2	-0.006165036	0.905792414	0.926107081
IFITM3	0.050041841	0.336439713	0.410407717
IFITM4P	0.181673769	0.000436808	0.001068003
IFITM5	0.024764257	0.634461574	0.698753026
IFLTD1	0.134751563	0.009360424	0.017390503
IFNA14	0.03027148	0.561081728	0.631446739
IFNA1	0.040721425	0.434198313	0.510300206
IFNA21	0.053170215	0.307066203	0.37967806
IFNA4	0.077237687	0.137569885	0.189573719
IFNA5	0.01308655	0.801640214	0.841204969
IFNA7	-0.07613765	0.143278327	0.196407543
IFNAR1	-0.170442348	0.000980642	0.002242206
IFNAR2	0.216604822	2.58E-05	7.89E-05

IFNB1	0.159901886	0.002005365	0.004314351
IFNE	0.233056855	5.72E-06	1.98E-05
IFNGR1	-0.231361722	6.71E-06	2.29E-05
IFNGR2	0.426122338	8.46E-18	1.89E-16
IFNG	0.163518056	0.001576345	0.003458585
IFNK	-0.000207851	0.996816459	0.997417252
IFNW1	0.090960583	0.080162981	0.11833187
IFRD1	0.256977715	5.23E-07	2.16E-06
IFRD2	-0.119700313	0.021104607	0.036077396
IFT122	-0.128813608	0.013024237	0.023369255
IFT140	0.043268161	0.405984629	0.482233829
IFT172	0.252468705	8.37E-07	3.33E-06
IFT20	0.158236679	0.002236764	0.004765863
IFT27	-0.066797376	0.199246671	0.261591682
IFT46	-0.233716422	5.37E-06	1.87E-05
IFT52	0.463226871	3.93E-21	1.43E-19
IFT57	0.340158475	1.68E-11	1.42E-10
IFT74	-0.050040663	0.336451101	0.410407717
IFT80	0.313594037	6.56E-10	4.35E-09
IFT81	0.11535567	0.02629471	0.043976092
IFT88	-0.326312086	1.19E-10	8.82E-10
IGBP1	0.052006539	0.317788603	0.3910429
IGDCC3	0.09381959	0.071078136	0.106363602
IGDCC4	0.433296396	2.06E-18	5.09E-17
IGF1R	0.220977625	1.75E-05	5.51E-05
IGF1	-0.257866106	4.77E-07	1.98E-06
IGF2AS	-0.040667927	0.434802837	0.510950399
IGF2BP1	0.306706276	1.60E-09	1.00E-08
IGF2BP2	0.4217387	1.97E-17	4.22E-16
IGF2BP3	0.358314499	1.11E-12	1.13E-11
IGF2R	0.106192906	0.040923793	0.065306297
IGF2	-0.034424271	0.508601587	0.581484121
IGFALS	-0.048970363	0.34690102	0.421304339
IGFBP1	-0.289623028	1.33E-08	7.23E-08
IGFBP2	-0.258565796	4.43E-07	1.85E-06
IGFBP3	0.162236783	0.001717655	0.003739794
IGFBP4	-0.311419861	8.72E-10	5.69E-09
IGFBP5	0.011480202	0.825571032	0.861472674
IGFBP6	0.26998424	1.28E-07	5.87E-07
IGFBP7	0.003669951	0.943835532	0.956608235
IGFBPL1	0.250245584	1.05E-06	4.13E-06
IGFL1	0.109545	0.034925858	0.056609953
IGFL2	0.249055368	1.19E-06	4.61E-06
IGFL3	0.046617549	0.370587058	0.446443452
IGFL4	0.061975389	0.233713623	0.300292989
IGFN1	0.166049267	0.001328027	0.002960053
IGHMBP2	-0.022099734	0.671357008	0.731381545
IGJ	0.169070747	0.00107887	0.002448536
IGLL1	0.12765253	0.013872876	0.024707262
IGLL3	0.174917398	0.000714635	0.001680471
IGLON5	0.280957151	3.70E-08	1.86E-07
IGSF10	-0.236507564	4.11E-06	1.46E-05
IGSF11	0.212605317	3.65E-05	0.000109301
IGSF1	0.359984643	8.58E-13	8.86E-12
IGSF21	0.047655615	0.360015989	0.435533668
IGSF22	0.300278828	3.61E-09	2.14E-08
IGSF3	0.449029632	8.28E-20	2.46E-18
IGSF5	0.175806488	0.000670479	0.001586188

IGSF6	0.238313134	3.45E-06	1.24E-05
IGSF8	-0.292945201	8.92E-09	4.98E-08
IGSF9B	0.030627337	0.556484343	0.62707377
IGSF9	0.013298261	0.798499489	0.838792872
IHH	-0.036176064	0.48725821	0.56139946
IKBIP	0.359851125	8.76E-13	9.04E-12
IKBKAP	-0.038447784	0.460313547	0.535152106
IKBKB	0.126402427	0.014840584	0.026226746
IKBKE	0.583795284	2.86E-35	1.58E-32
IKBKG	-0.136418009	0.008512394	0.015939836
IKZF1	0.20075071	9.89E-05	0.000273338
IKZF2	-0.112542988	0.030211542	0.049766358
IKZF3	0.060353979	0.246197749	0.314064901
IKZF4	0.282064947	3.25E-08	1.65E-07
IKZF5	-0.294794494	7.12E-09	4.04E-08
IK	0.24441573	1.89E-06	7.10E-06
IL10RA	0.118342478	0.022621822	0.038446676
IL10RB	0.122160506	0.018579623	0.032133281
IL10	0.200764796	9.87E-05	0.00027313
IL11RA	-0.036915478	0.478396113	0.552787389
IL11	0.315056488	5.41E-10	3.63E-09
IL12A	0.361888883	6.38E-13	6.72E-12
IL12B	0.114453981	0.027499835	0.045761085
IL12RB1	0.271598741	1.07E-07	4.96E-07
IL12RB2	-0.103959423	0.045385081	0.071605415
IL13RA1	-0.365359706	3.69E-13	4.07E-12
IL13RA2	-0.12498557	0.016008549	0.02808651
IL13	0.070665776	0.174392507	0.232842807
IL15RA	0.353226536	2.42E-12	2.35E-11
IL15	0.208581782	5.15E-05	0.000149901
IL16	0.071247279	0.170865072	0.228827625
IL17A	0.03038125	0.559661631	0.629990904
IL17B	0.203545083	7.86E-05	0.00022116
IL17C	0.154028883	0.002934045	0.006084318
IL17D	0.320313117	2.68E-10	1.89E-09
IL17F	0.17451823	0.000735321	0.001724841
IL17RA	0.038621798	0.458284395	0.533290989
IL17RB	0.250235981	1.05E-06	4.13E-06
IL17RC	-0.275549077	6.88E-08	3.29E-07
IL17RD	0.309773549	1.08E-09	6.93E-09
IL17REL	0.25603387	5.78E-07	2.36E-06
IL17RE	0.101669314	0.050376388	0.078559562
IL18BP	0.269881531	1.30E-07	5.94E-07
IL18R1	0.034682774	0.505421651	0.57863364
IL18RAP	0.115981481	0.025485269	0.04275516
IL18	0.260982859	3.42E-07	1.46E-06
IL19	0.120705609	0.020038948	0.034394411
IL1A	0.273839366	8.34E-08	3.92E-07
IL1B	0.334543962	3.75E-11	3.02E-10
IL1F10	0.071130507	0.171569114	0.229627134
IL1F5	0.106183626	0.040941536	0.065329377
IL1F6	-0.049323038	0.343435163	0.417775863
IL1F7	0.156829342	0.002451054	0.00518309
IL1F8	0.105492699	0.042280794	0.067181208
IL1F9	-0.053702382	0.302243143	0.374737858
IL1R1	-0.211270292	4.10E-05	0.000121528
IL1R2	0.076116182	0.143391503	0.196517811
IL1RAPL1	0.008483867	0.870626832	0.898173464

IL1RAPL2	-0.151133338	0.003522966	0.007173397
IL1RAP	0.038188933	0.463341234	0.538263692
IL1RL1	-0.148334763	0.004191946	0.008394847
IL1RL2	0.050321431	0.333743563	0.407758971
IL1RN	-0.05698929	0.273570483	0.343788
IL20RA	0.298142612	4.71E-09	2.74E-08
IL20RB	0.310125595	1.03E-09	6.65E-09
IL20	0.086163221	0.097498562	0.140537324
IL21R	0.302223242	2.83E-09	1.71E-08
IL21	0.075882429	0.144628261	0.197903991
IL22RA1	0.040439729	0.437386868	0.513199905
IL22RA2	0.218239811	2.23E-05	6.90E-05
IL22	0.160585002	0.001916936	0.004138854
IL23A	0.057967003	0.265411221	0.335013771
IL23R	0.082845677	0.111143309	0.157616529
IL24	0.068481572	0.188126169	0.248547619
IL25	-0.011767284	0.821281629	0.857974442
IL26	0.204543479	7.23E-05	0.000204852
IL27RA	0.380933687	2.94E-14	3.83E-13
IL27	-0.434960124	1.48E-18	3.76E-17
IL28A	0.108755306	0.036267039	0.05852022
IL28B	0.049595282	0.340774854	0.414995821
IL28RA	-0.274612479	7.65E-08	3.62E-07
IL29	0.030712461	0.55538732	0.626099264
IL2RA	0.240950327	2.67E-06	9.78E-06
IL2RB	0.148894887	0.004049516	0.008136758
IL2RG	0.333920748	4.10E-11	3.28E-10
IL2	0.114012803	0.028106568	0.046649925
IL31RA	0.153001329	0.003131932	0.006461719
IL31	0.030952847	0.552295092	0.623495372
IL32	0.04370302	0.401278477	0.477412351
IL33	-0.073716278	0.156479144	0.211979975
IL34	0.215450268	2.85E-05	8.66E-05
IL3RA	0.093964696	0.070640437	0.105831031
IL3	-0.029938483	0.565400292	0.635230352
IL4I1	0.412256209	1.18E-16	2.25E-15
IL4R	0.279780287	4.24E-08	2.11E-07
IL4	0.02938586	0.572602045	0.641981988
IL5RA	0.131380453	0.011308601	0.020585704
IL5	0.081101087	0.118898044	0.167103127
IL6R	-0.273772332	8.41E-08	3.95E-07
IL6ST	-0.155021321	0.002753777	0.005745183
IL6	0.049855384	0.338245503	0.412293619
IL7R	0.093285601	0.072707976	0.108557917
IL7	0.064006898	0.218711648	0.283670146
IL8	0.279134649	4.57E-08	2.25E-07
IL9R	0.340397461	1.62E-11	1.38E-10
IL9	0.086715552	0.095362181	0.13780686
ILDR1	0.362275942	6.00E-13	6.37E-12
ILDR2	0.117557933	0.023540789	0.03984195
ILF2	0.391092595	5.23E-15	7.68E-14
ILF3	0.437364498	9.13E-19	2.38E-17
ILKAP	0.253252083	7.72E-07	3.09E-06
ILK	0.119440516	0.021387872	0.036514627
ILVBL	-0.088417372	0.089013611	0.12964828
IMMP1L	0.199094814	0.000113102	0.000309169
IMMP2L	-0.250063934	1.07E-06	4.20E-06
IMMT	0.100270294	0.053645177	0.083058927

IMP3	-0.155225904	0.002717893	0.005680432
IMP4	0.006590264	0.899327855	0.920915422
IMP5	0.017598661	0.735471377	0.785549044
IMPA1	-0.094585334	0.068793112	0.103449304
IMPA2	0.121735727	0.018995806	0.032770563
IMPACT	-0.272434306	9.77E-08	4.55E-07
IMPAD1	-0.228061877	9.14E-06	3.04E-05
IMPDH1	0.433593395	1.94E-18	4.82E-17
IMPDH2	0.14239001	0.006007304	0.011621433
IMPG1	0.172847633	0.000828052	0.001922215
IMPG2	0.096897693	0.062255343	0.09468427
INADL	0.057899206	0.265971557	0.335593474
INA	-0.077308268	0.137209671	0.189116582
INCA1	-0.183126518	0.000392033	0.00096712
INCENP	0.397440927	1.72E-15	2.72E-14
INE1	0.008880786	0.864630968	0.893653859
INE2	0.076976505	0.138909172	0.191241643
INF2	0.199236048	0.000111815	0.000305987
ING1	-0.250886894	9.85E-07	3.89E-06
ING2	0.102517296	0.048477253	0.07601447
ING3	0.00301713	0.953813903	0.964073088
ING4	-0.168068866	0.001156263	0.00260932
ING5	0.129319573	0.012668961	0.02278926
INGX	0.056791861	0.275238585	0.345557291
INHA	0.217462379	2.39E-05	7.35E-05
INHBA	-0.162956472	0.001636915	0.003576903
INHBB	0.000306084	0.995311898	0.996442872
INHBC	-0.269486923	1.36E-07	6.18E-07
INHBE	0.105370403	0.042521651	0.067509456
INMT	-0.089824627	0.084024031	0.123291357
INO80B	-0.231783687	6.45E-06	2.21E-05
INO80C	0.159630232	0.002041555	0.004388418
INO80D	0.103018693	0.047382767	0.074468245
INO80E	0.140647607	0.006659208	0.012769731
INO80	-0.021308728	0.682468828	0.74065174
INPP1	-0.040447501	0.437298722	0.513187154
INPP4A	0.361979344	6.29E-13	6.64E-12
INPP4B	0.05026849	0.334253013	0.408151305
INPP5A	-0.101908195	0.049835213	0.077800886
INPP5B	-0.202259311	8.74E-05	0.000243913
INPP5D	0.229042742	8.34E-06	2.80E-05
INPP5E	0.117293948	0.023857175	0.040302099
INPP5F	0.304615146	2.09E-09	1.29E-08
INPP5J	0.435338291	1.37E-18	3.50E-17
INPP5K	-0.00883108	0.865381419	0.894285832
INPPL1	0.28662401	1.90E-08	1.00E-07
INS-IGF2	-0.256791359	5.34E-07	2.20E-06
INSC	0.089301394	0.085852214	0.12564077
INSIG1	-0.279320029	4.47E-08	2.21E-07
INSIG2	-0.272307744	9.91E-08	4.60E-07
INSL3	0.301927596	2.94E-09	1.77E-08
INSL4	0.17258332	0.000843676	0.001955523
INSL5	0.024598704	0.636729004	0.700785328
INSL6	0.007492172	0.885639086	0.909750535
INSM1	0.272520765	9.68E-08	4.50E-07
INSM2	0.052790723	0.310536401	0.383373996
INSRR	0.179620416	0.000508244	0.00122879
INSR	-0.286128519	2.02E-08	1.06E-07

INS	0.078950384	0.129031939	0.179384109
INTS10	0.008964793	0.863362941	0.892528489
INTS12	-0.077453344	0.136471536	0.188268537
INTS1	0.009914417	0.849054134	0.880844476
INTS2	0.00668197	0.897934551	0.919583207
INTS3	0.183746558	0.000374255	0.000926834
INTS4L1	0.14013046	0.006864524	0.013126804
INTS4L2	0.042671684	0.412492957	0.488624373
INTS4	0.309334025	1.14E-09	7.31E-09
INTS5	-0.212575585	3.66E-05	0.000109566
INTS6	0.040469931	0.437044368	0.513100235
INTS7	0.249968951	1.08E-06	4.24E-06
INTS8	0.461081636	6.29E-21	2.21E-19
INTS9	0.165898304	0.001341764	0.002987329
INTU	0.08198231	0.114929785	0.162293109
INVS	-0.091137102	0.079576099	0.117596249
IP6K1	0.188317427	0.0002646	0.000674002
IP6K2	0.293127424	8.72E-09	4.89E-08
IP6K3	0.194444935	0.000164122	0.000435371
IPCEF1	0.187296768	0.000286102	0.00072451
IPMK	0.022120644	0.671064226	0.731142549
IPO11	-0.045645988	0.380652876	0.456829313
IPO13	-0.002533622	0.961209052	0.969452772
IPO4	0.067667449	0.193443395	0.254744798
IPO5	0.116407891	0.024946119	0.041928337
IPO7	0.125285492	0.015754808	0.027685215
IPO8	-0.074236213	0.153569808	0.208563482
IPO9	0.268357119	1.54E-07	6.94E-07
IPPK	0.101912079	0.049826455	0.077793311
IPP	-0.171908221	0.000884829	0.00204188
IPW	0.302800397	2.63E-09	1.59E-08
IQCA1	0.218403797	2.20E-05	6.81E-05
IQCB1	0.327883532	9.54E-11	7.22E-10
IQCC	0.232446912	6.06E-06	2.09E-05
IQCD	0.408221708	2.48E-16	4.48E-15
IQCE	0.218474565	2.18E-05	6.77E-05
IQCF1	0.1265435	0.014728491	0.0260628
IQCF2	0.048702505	0.349548112	0.424227687
IQCF3	0.092533237	0.07505591	0.111578527
IQCF5	0.101680476	0.050350993	0.078532254
IQCF6	3.72E-05	0.99942979	0.99947996
IQCG	-0.098664652	0.057612175	0.088438107
IQCH	-0.313564427	6.59E-10	4.36E-09
IQCJ	0.045748751	0.379580363	0.455706881
IQCK	0.042810159	0.410976555	0.487146721
IQGAP1	0.330626498	6.52E-11	5.08E-10
IQGAP2	-0.413356795	9.61E-17	1.86E-15
IQGAP3	0.56757695	5.09E-33	1.41E-30
IQSEC1	-0.164531433	0.001472221	0.003254504
IQSEC2	0.231070063	6.90E-06	2.35E-05
IQSEC3	-0.138736615	0.007446396	0.014124259
IQUB	0.077000974	0.138783276	0.191087181
IRAK1BP1	0.158857996	0.002147717	0.00459431
IRAK1	0.195681033	0.00014878	0.000397852
IRAK2	0.06222404	0.231839367	0.298227779
IRAK3	0.056503205	0.277689893	0.348239837
IRAK4	0.198369189	0.000119936	0.000326505
IREB2	-0.14799775	0.004279808	0.008563054

IRF1	0.292282643	9.66E-09	5.36E-08
IRF2BP1	-0.058283665	0.262804754	0.332376607
IRF2BP2	0.10599831	0.041297182	0.065833596
IRF2	-0.153662576	0.003003246	0.006215528
IRF3	0.27568734	6.77E-08	3.24E-07
IRF4	0.200507231	0.000100837	0.000278352
IRF5	0.256141007	5.71E-07	2.34E-06
IRF6	-0.200176318	0.000103593	0.000285012
IRF7	0.093218227	0.072915762	0.108843685
IRF8	0.053180696	0.306970725	0.379615351
IRF9	-0.065701588	0.206734372	0.270034235
IRGC	0.160913316	0.001875718	0.004058657
IRGM	-0.021729714	0.67654621	0.735436705
IRGQ	0.179187174	0.00052464	0.00126582
IRS1	-0.511425741	4.08E-26	3.26E-24
IRS2	-0.427110056	6.98E-18	1.58E-16
IRS4	0.049027149	0.346341465	0.420746062
IRX1	-0.06366337	0.221198872	0.286459932
IRX2	0.050641863	0.330670672	0.404707985
IRX3	-0.121494104	0.019236144	0.033142131
IRX4	0.028666831	0.582036803	0.650692323
IRX5	0.030909082	0.552857448	0.624059498
IRX6	-0.00551391	0.915703097	0.934034975
ISCA1P1	-0.205113717	6.90E-05	0.000196187
ISCA1	-0.189163513	0.00024793	0.000635276
ISCA2	-0.044978123	0.387668306	0.463962994
ISCU	-0.199771735	0.000107059	0.000293979
ISG15	0.072834211	0.161509743	0.21784679
ISG20L2	0.230469099	7.30E-06	2.48E-05
ISG20	0.192636761	0.000189253	0.000495895
ISL1	0.109780378	0.034534352	0.056034645
ISL2	0.266085232	1.97E-07	8.75E-07
ISLR2	-0.003518412	0.946151078	0.958435659
ISLR	0.25880514	4.32E-07	1.81E-06
ISM1	-0.065074141	0.211112347	0.275049387
ISM2	0.119587367	0.021227353	0.036259224
ISOC1	-0.07558582	0.146209273	0.199847762
ISOC2	-0.19213228	0.000196882	0.000514262
ISPD	-0.269422543	1.37E-07	6.22E-07
ISX	0.097514643	0.060600288	0.092440961
ISY1	0.272089495	1.02E-07	4.71E-07
ISYNA1	0.274065641	8.13E-08	3.83E-07
ITCH	-0.2939877	7.86E-09	4.42E-08
ITFG1	-0.34251683	1.19E-11	1.04E-10
ITFG2	0.194790969	0.000159684	0.000424446
ITFG3	-0.040836649	0.432897941	0.508922033
ITGA10	0.02164007	0.67780571	0.736513873
ITGA11	0.210754424	4.28E-05	0.000126649
ITGA1	-0.153542592	0.003026234	0.006258803
ITGA2B	0.153569623	0.003021041	0.006251057
ITGA2	0.169260145	0.001064787	0.002417675
ITGA3	0.344743664	8.59E-12	7.65E-11
ITGA4	0.196359094	0.000140946	0.000378478
ITGA5	0.3720213	1.27E-13	1.51E-12
ITGA6	-0.084722035	0.103252616	0.147857865
ITGA7	-0.158719119	0.002167335	0.004630311
ITGA8	-0.01067121	0.837685993	0.871536341
ITGA9	0.048771796	0.348862124	0.423472535

ITGAD	0.012216203	0.814584872	0.852050815
ITGAE	0.234009436	5.23E-06	1.82E-05
ITGAL	0.154378377	0.002869374	0.005963245
ITGAM	0.2972387	5.27E-09	3.04E-08
ITGAV	0.211526557	4.01E-05	0.000119112
ITGAX	0.336144155	2.99E-11	2.44E-10
ITGB1BP1	0.308585677	1.26E-09	8.00E-09
ITGB1BP2	-0.034853569	0.503326375	0.576644318
ITGB1BP3	0.146855653	0.004590067	0.0091188
ITGB1	0.163728212	0.001554212	0.00341529
ITGB2	0.271389006	1.10E-07	5.07E-07
ITGB3BP	0.245105066	1.77E-06	6.65E-06
ITGB3	-0.093113301	0.073240326	0.109229957
ITGB4	0.348103711	5.23E-12	4.80E-11
ITGB5	0.223790463	1.35E-05	4.37E-05
ITGB6	0.360619071	7.77E-13	8.10E-12
ITGB7	-0.064014653	0.218655732	0.283616088
ITGB8	0.349437137	4.28E-12	4.00E-11
ITGBL1	0.103447563	0.046463089	0.073126692
ITIH1	-0.385501071	1.36E-14	1.86E-13
ITIH2	-0.158825249	0.002152328	0.004602198
ITIH3	-0.327068542	1.07E-10	8.02E-10
ITIH4	-0.468075859	1.34E-21	5.16E-20
ITIH5L	0.241843749	2.45E-06	9.00E-06
ITIH5	0.164409225	0.001484434	0.003278955
ITK	0.117691777	0.023381766	0.039613193
ITLN1	0.23193295	6.36E-06	2.18E-05
ITLN2	0.025306642	0.627056993	0.691860291
ITM2A	0.045964986	0.37732962	0.453524023
ITM2B	-0.112444061	0.030357942	0.04997446
ITM2C	0.350970268	3.40E-12	3.22E-11
ITPA	0.238283359	3.46E-06	1.24E-05
ITPK1	-0.069854568	0.179403413	0.238542001
ITPKA	0.342798239	1.14E-11	9.98E-11
ITPKB	0.03377298	0.516659415	0.58924255
ITPKC	0.251710251	9.05E-07	3.58E-06
ITPR1	0.035174501	0.499401643	0.573136674
ITPR2	-0.484741543	2.93E-23	1.43E-21
ITPR3	0.487152262	1.66E-23	8.46E-22
ITPRIPL1	0.246481737	1.54E-06	5.86E-06
ITPRIPL2	0.280085565	4.09E-08	2.04E-07
ITPRIP	-0.185360648	0.000331435	0.000829504
ITSN1	-0.04460753	0.391594754	0.467959372
ITSN2	-0.040441609	0.437365544	0.513199905
IVD	-0.567702758	4.89E-33	1.39E-30
IVL	0.102475343	0.048569783	0.0761296
IVNS1ABP	0.21484609	3.00E-05	9.11E-05
IWS1	0.239476351	3.08E-06	1.12E-05
IYD	-0.316789894	4.30E-10	2.94E-09
IZUMO1	0.046969735	0.366979276	0.442793359
JAG1	0.220848964	1.77E-05	5.58E-05
JAG2	0.243894463	2.00E-06	7.45E-06
JAGN1	0.003288669	0.949662465	0.960948043
JAK1	-0.190978245	0.000215429	0.000558472
JAK2	0.123811237	0.017036816	0.029710035
JAK3	0.339794444	1.77E-11	1.49E-10
JAKMIP1	0.226941918	1.01E-05	3.35E-05
JAKMIP2	-0.218884516	2.10E-05	6.55E-05

JAKMIP3	0.150083877	0.003761577	0.007615664
JAM2	-0.079118746	0.128215274	0.178385689
JAM3	0.106857726	0.039669572	0.0634573
JARID2	0.145953039	0.004849467	0.009585383
JAZF1	-0.061757398	0.235365554	0.302123232
JDP2	-0.15595041	0.00259421	0.005458581
JHDM1D	-0.007227928	0.889646334	0.913066522
JKAMP	-0.111702084	0.031475447	0.051592385
JMJD1C	-0.16969633	0.001032995	0.002351559
JMJD4	0.08816545	0.089931427	0.130832035
JMJD5	-0.246122928	1.60E-06	6.06E-06
JMJD6	0.362472251	5.82E-13	6.19E-12
JMJD7-PLA:	0.002590022	0.960346245	0.968856935
JMJD8	-0.228949163	8.42E-06	2.82E-05
JMY	-0.15807511	0.002260466	0.00481019
JOSD1	0.140480524	0.006724933	0.012883365
JOSD2	0.121489325	0.019240923	0.033147499
JPH1	0.375712251	6.97E-14	8.59E-13
JPH2	0.18113889	0.000454458	0.001108166
JPH3	-0.008668963	0.867829831	0.896123251
JPH4	0.065169323	0.21044396	0.274232377
JRKL	0.26906866	1.42E-07	6.45E-07
JRK	0.348145836	5.19E-12	4.77E-11
JSRP1	0.226168796	1.09E-05	3.57E-05
JTB	0.065257796	0.209824054	0.273571977
JUB	-0.052874959	0.309763908	0.382562555
JUNB	0.140481346	0.006724608	0.012883365
JUND	-0.106169759	0.040968058	0.06536557
JUN	-0.12218762	0.018553331	0.032090593
JUP	0.071720291	0.16803523	0.225512365
KAAG1	0.226347037	1.07E-05	3.52E-05
KAL1	0.183555752	0.000379645	0.000938721
KALRN	-0.124280637	0.016619074	0.029047656
KANK1	-0.373075689	1.07E-13	1.28E-12
KANK2	-0.064955695	0.211946237	0.275991433
KANK3	-0.217701437	2.34E-05	7.21E-05
KANK4	0.013780823	0.79135288	0.833218755
KARS	0.035641558	0.493718979	0.567661424
KAT2A	0.21231739	3.74E-05	0.000111885
KAT2B	-0.484194174	3.34E-23	1.62E-21
KAT5	0.10833597	0.036996823	0.059607821
KATNA1	0.3313432	5.89E-11	4.61E-10
KATNAL1	-0.060543751	0.244712997	0.312453055
KATNAL2	-0.000725789	0.988883826	0.991531293
KATNB1	-0.091959481	0.076887593	0.113986801
KAZALD1	-0.019092584	0.71396099	0.767629469
KAZ	0.002229066	0.965868922	0.973196473
KBTBD10	0.041046697	0.430533205	0.506889812
KBTBD11	-0.200434768	0.000101434	0.000279808
KBTBD12	0.13848872	0.007554377	0.014314521
KBTBD13	-0.007690661	0.882630908	0.907455899
KBTBD2	0.240303043	2.84E-06	1.04E-05
KBTBD3	-0.148665242	0.004107373	0.008242051
KBTBD4	-0.059622911	0.251976267	0.320430946
KBTBD5	0.000403448	0.993820659	0.995319484
KBTBD6	-0.18431976	0.000358491	0.000891396
KBTBD7	-0.189772827	0.000236539	0.000608199
KBTBD8	0.072188796	0.165267045	0.222237601

KC6	0.15272307	0.003187566	0.006560822
KCMF1	0.248062301	1.31E-06	5.06E-06
KCNA10	0.159858504	0.002011105	0.004325765
KCNA1	0.1444324	0.005316479	0.010399106
KCNA2	0.15959954	0.002045681	0.004396339
KCNA3	0.027259075	0.600715162	0.667491074
KCNA4	0.106219597	0.040872802	0.06523015
KCNA5	0.052581039	0.312464839	0.385301097
KCNA6	0.152993593	0.003133467	0.006464216
KCNA7	0.238596895	3.36E-06	1.21E-05
KCNAB1	-0.038501479	0.459686886	0.53461075
KCNAB2	0.131084379	0.011495728	0.02088817
KCNAB3	0.18374291	0.000374358	0.000926915
KCNB1	-0.308592557	1.26E-09	7.99E-09
KCNB2	0.115046438	0.026702781	0.044579972
KCNC1	0.191242137	0.000211049	0.000548678
KCNC2	0.223226535	1.42E-05	4.57E-05
KCNC3	-0.006386755	0.902420915	0.923199468
KCNC4	0.204480975	7.27E-05	0.000205831
KCND1	0.230174371	7.51E-06	2.54E-05
KCND2	0.313407938	6.72E-10	4.45E-09
KCND3	-0.221357341	1.69E-05	5.34E-05
KCNE1L	0.274147661	8.06E-08	3.80E-07
KCNE1	-0.121725739	0.019005689	0.032784773
KCNE2	-0.337497974	2.46E-11	2.03E-10
KCNE3	0.110430122	0.033472963	0.054525623
KCNE4	0.200116829	0.000104096	0.000286238
KCNF1	0.445540066	1.71E-19	4.88E-18
KCNG1	0.374281987	8.81E-14	1.07E-12
KCNG2	0.06988333	0.179223948	0.238335191
KCNG3	0.367175397	2.77E-13	3.11E-12
KCNG4	0.085317878	0.100841879	0.144842964
KCNH1	-0.148521897	0.004143865	0.008306075
KCNH2	0.352791424	2.59E-12	2.49E-11
KCNH3	0.472026787	5.53E-22	2.24E-20
KCNH4	0.291010483	1.13E-08	6.18E-08
KCNH5	0.109462286	0.035064328	0.056797426
KCNH6	0.085838339	0.098772873	0.14217953
KCNH7	0.101280986	0.051266596	0.079760494
KCNH8	0.114313521	0.027691771	0.046038176
KCNIP1	0.06653416	0.201027005	0.263651085
KCNIP2	0.136396539	0.00852287	0.015957952
KCNIP3	0.226233965	1.08E-05	3.56E-05
KCNIP4	0.047320008	0.363412803	0.438971431
KCNJ10	0.14910773	0.003996551	0.008043227
KCNJ11	0.40257289	6.91E-16	1.17E-14
KCNJ12	0.167141126	0.001232455	0.00276435
KCNJ13	0.125132852	0.015883501	0.027896598
KCNJ14	0.044585552	0.391828359	0.468154286
KCNJ15	-0.015548014	0.765334281	0.810910013
KCNJ16	0.201684622	9.16E-05	0.000254597
KCNJ1	0.179438196	0.000515081	0.001244264
KCNJ2	0.098886477	0.057050067	0.087669811
KCNJ3	0.01660347	0.749917457	0.797813499
KCNJ4	-0.060115963	0.248068835	0.316087453
KCNJ5	-0.018766409	0.718638857	0.771791014
KCNJ6	0.052853428	0.309961242	0.382758808
KCNJ8	-0.543621621	6.48E-30	9.15E-28

KCNJ9	-0.073377021	0.158399799	0.214304978
KCNK10	0.146669412	0.004642543	0.009215697
KCNK12	0.369658898	1.86E-13	2.15E-12
KCNK13	0.351628787	3.08E-12	2.93E-11
KCNK15	0.249133752	1.18E-06	4.58E-06
KCNK16	0.02842023	0.585289166	0.653484882
KCNK17	-0.072641312	0.162625916	0.219263231
KCNK1	-0.091770762	0.077497939	0.114806212
KCNK2	0.187223258	0.000287712	0.000728216
KCNK3	0.204095556	7.51E-05	0.000212141
KCNK4	0.068548357	0.187694796	0.248027044
KCNK5	0.098309224	0.058522386	0.089641933
KCNK6	0.20263292	8.47E-05	0.00023709
KCNK7	0.122302329	0.018442451	0.031915438
KCNK9	0.28098535	3.69E-08	1.86E-07
KCNMA1	-0.211251103	4.10E-05	0.000121692
KCNMB1	0.269207911	1.40E-07	6.36E-07
KCNMB2	0.10034936	0.053455851	0.082785095
KCNMB3	0.441991752	3.56E-19	9.74E-18
KCNMB4	0.294020061	7.82E-09	4.41E-08
KCNN1	0.160355802	0.001946201	0.004197489
KCNN2	0.095646774	0.065725485	0.099361293
KCNN3	0.010339643	0.842662609	0.875535856
KCNN4	0.353274417	2.40E-12	2.33E-11
KCNQ1DN	0.048487511	0.351681989	0.426349941
KCNQ1OT1	0.151345603	0.003476405	0.007090186
KCNQ1	0.247932641	1.33E-06	5.12E-06
KCNQ2	0.120212487	0.020555689	0.035214587
KCNQ3	0.190135993	0.000229984	0.000592875
KCNQ4	0.151138949	0.003521728	0.00717161
KCNQ5	0.230189816	7.50E-06	2.54E-05
KCNRG	-0.130407553	0.011933816	0.021617156
KCNS1	0.25985669	3.86E-07	1.63E-06
KCNS2	0.086385003	0.096636192	0.139460945
KCNS3	0.171626694	0.000902531	0.002076718
KCNT1	-0.007791811	0.881098584	0.906114288
KCNT2	0.121919253	0.018815009	0.032492424
KCNU1	-0.232223767	6.19E-06	2.13E-05
KCNV1	0.204000875	7.57E-05	0.000213627
KCNV2	0.039998963	0.442402797	0.518088241
KCP	0.390989875	5.32E-15	7.80E-14
KCTD10	0.388130076	8.70E-15	1.23E-13
KCTD11	0.010305335	0.843177908	0.875894535
KCTD12	0.129041196	0.012863359	0.023105566
KCTD13	0.040422462	0.437582758	0.513399511
KCTD14	0.005202722	0.920444349	0.937862741
KCTD15	0.12533247	0.015715384	0.027620811
KCTD16	-0.075551274	0.14639427	0.200032006
KCTD17	0.465622316	2.32E-21	8.65E-20
KCTD18	-0.303458813	2.42E-09	1.47E-08
KCTD19	0.138428576	0.007580785	0.014361392
KCTD1	0.278048945	5.17E-08	2.53E-07
KCTD20	-0.050973135	0.327513067	0.401610143
KCTD21	-0.39744444	1.72E-15	2.72E-14
KCTD2	0.106101687	0.041098467	0.065553535
KCTD3	0.040303435	0.438934401	0.514730694
KCTD4	0.034306757	0.510050604	0.582906616
KCTD5	0.269238038	1.39E-07	6.34E-07

KCTD6	0.181592691	0.000439442	0.001074179
KCTD7	0.291436049	1.07E-08	5.88E-08
KCTD8	0.118334156	0.022631404	0.038459681
KCTD9	0.236427647	4.14E-06	1.47E-05
KDELC1	0.226355824	1.07E-05	3.52E-05
KDELC2	0.165155703	0.001411259	0.003132951
KDELR1	0.172102634	0.000872792	0.002016439
KDELR2	-0.129780073	0.012353065	0.022293484
KDELR3	0.230036677	7.60E-06	2.57E-05
KDM1A	0.181559781	0.000440515	0.001076538
KDM1B	-0.000208795	0.996802003	0.997417252
KDM2A	0.031562601	0.544489417	0.615838302
KDM2B	0.058719581	0.259245572	0.328584444
KDM3A	0.084166154	0.105542603	0.150703823
KDM3B	0.230279261	7.43E-06	2.52E-05
KDM4A	0.001335137	0.979552632	0.984196466
KDM4B	-0.199341315	0.000110865	0.000303596
KDM4C	-0.102793541	0.047871657	0.075172937
KDM4DL	-0.014671825	0.77820359	0.821718976
KDM4D	-0.103364714	0.046639576	0.073387065
KDM5A	-0.108732058	0.036307176	0.058577224
KDM5B	0.241406722	2.55E-06	9.37E-06
KDM5C	0.2739659	8.23E-08	3.87E-07
KDM5D	-0.061495259	0.237362909	0.30433414
KDM6A	0.154277545	0.002887898	0.005996737
KDM6B	0.101308859	0.051202267	0.079685302
KDR	-0.291405734	1.07E-08	5.90E-08
KDSR	-0.217519822	2.38E-05	7.31E-05
KEAP1	-0.21806123	2.26E-05	7.00E-05
KEL	0.228085298	9.12E-06	3.04E-05
KERA	0.204564342	7.22E-05	0.000204555
KGFLP1	-0.167860627	0.001172978	0.002644343
KGFLP2	-0.20810791	5.36E-05	0.000155582
KHDC1L	0.210031985	4.55E-05	0.000134044
KHDC1	0.35731397	1.30E-12	1.31E-11
KHDRBS1	0.409756782	1.87E-16	3.45E-15
KHDRBS2	0.214322856	3.15E-05	9.50E-05
KHDRBS3	0.056672762	0.276248201	0.346671915
KHK	-0.406506933	3.39E-16	6.00E-15
KHNYN	0.047434192	0.362254869	0.437755763
KHSRP	0.224899741	1.22E-05	3.98E-05
KIAA0020	0.17026325	0.000992982	0.00226782
KIAA0040	0.27509644	7.24E-08	3.45E-07
KIAA0087	0.120315748	0.020446533	0.035042657
KIAA0090	0.002491676	0.961850775	0.969981834
KIAA0100	0.256193911	5.68E-07	2.33E-06
KIAA0101	0.573601222	7.68E-34	2.59E-31
KIAA0114	0.113650876	0.028612856	0.047419126
KIAA0125	0.199192603	0.00011221	0.00030694
KIAA0141	-0.094848296	0.068022424	0.102483758
KIAA0146	0.215175922	2.92E-05	8.86E-05
KIAA0174	0.051383558	0.323628142	0.397590025
KIAA0182	0.020245834	0.697508279	0.753688774
KIAA0195	0.028170532	0.588590919	0.656215137
KIAA0196	0.221774748	1.62E-05	5.15E-05
KIAA0226	0.341768521	1.33E-11	1.15E-10
KIAA0232	-0.096026679	0.0646552	0.097921613
KIAA0240	-0.208393561	5.23E-05	0.000152153

KIAA0247	0.036338919	0.485298793	0.559400715
KIAA0284	0.18836967	0.000263541	0.000671648
KIAA0317	0.059408483	0.253688873	0.32234133
KIAA0319L	0.027087078	0.603015612	0.669561756
KIAA0319	0.151201146	0.003508031	0.007147371
KIAA0355	-0.07245412	0.163714596	0.220462497
KIAA0368	-0.022021826	0.672448316	0.732130033
KIAA0391	-0.200780602	9.86E-05	0.000272816
KIAA0406	0.167362817	0.001213839	0.002725666
KIAA0408	-0.101058129	0.051783373	0.080482787
KIAA0415	0.264163711	2.43E-07	1.06E-06
KIAA0427	-0.24186867	2.44E-06	8.98E-06
KIAA0430	-0.206407482	6.19E-05	0.000177661
KIAA0467	-0.130353921	0.011969152	0.021673314
KIAA0494	-0.19674449	0.000136668	0.000367783
KIAA0495	0.015056511	0.772545689	0.816827047
KIAA0513	0.040798222	0.433331361	0.509371453
KIAA0528	0.36772005	2.54E-13	2.87E-12
KIAA0556	0.094805062	0.068148646	0.102666162
KIAA0562	-0.155407937	0.002686323	0.005626846
KIAA0564	-0.373665201	9.74E-14	1.17E-12
KIAA0586	-0.022567374	0.664821035	0.725650047
KIAA0649	-0.031542269	0.544748822	0.616087214
KIAA0652	0.042609836	0.413171303	0.489340628
KIAA0664P3	-0.049597702	0.340751258	0.414992454
KIAA0664	-0.34993861	3.97E-12	3.72E-11
KIAA0748	0.162724727	0.001662526	0.003628091
KIAA0753	0.171109123	0.000935931	0.002147619
KIAA0754	-0.140676855	0.006647761	0.012750236
KIAA0776	-0.096122681	0.064387018	0.097552527
KIAA0802	0.409519197	1.96E-16	3.59E-15
KIAA0831	-0.153333236	0.003066727	0.006333748
KIAA0892	0.243188311	2.14E-06	7.94E-06
KIAA0895L	0.125901905	0.015244346	0.026878296
KIAA0895	0.105251723	0.042756489	0.067845064
KIAA0907	0.369865949	1.80E-13	2.08E-12
KIAA0913	-0.15653436	0.002498276	0.005274515
KIAA0922	-0.351183743	3.30E-12	3.12E-11
KIAA0947	0.173408806	0.000795761	0.00185417
KIAA1009	0.292395425	9.53E-09	5.29E-08
KIAA1012	-0.244770665	1.83E-06	6.87E-06
KIAA1024	0.294717859	7.19E-09	4.07E-08
KIAA1033	-0.047139097	0.365252136	0.440896332
KIAA1045	0.079384877	0.12693254	0.176897521
KIAA1107	-0.023272131	0.655018901	0.717190796
KIAA1109	-0.240362925	2.83E-06	1.03E-05
KIAA1143	-0.075892492	0.144574851	0.197844497
KIAA1147	-0.083266452	0.10933381	0.155331476
KIAA1161	-0.363047599	5.32E-13	5.70E-12
KIAA1191	-0.152318735	0.003270005	0.006706996
KIAA1199	0.136796155	0.00832976	0.015636058
KIAA1210	0.183688324	0.000375893	0.000930137
KIAA1211	0.315337581	5.21E-10	3.51E-09
KIAA1217	0.068458336	0.188276423	0.248663676
KIAA1239	0.120622004	0.020125755	0.034528529
KIAA1244	0.401901162	7.80E-16	1.31E-14
KIAA1257	0.18791974	0.000272792	0.000693892
KIAA1267	0.121112977	0.019620623	0.033737445

KIAA1274	0.262586276	2.88E-07	1.24E-06
KIAA1279	0.1331733	0.010231948	0.018837525
KIAA1310	0.283109357	2.88E-08	1.48E-07
KIAA1324L	0.173195663	0.000807886	0.001880442
KIAA1324	0.498430159	1.08E-24	6.66E-23
KIAA1328	0.146855864	0.004590008	0.0091188
KIAA1370	-0.306556331	1.63E-09	1.02E-08
KIAA1377	0.354367058	2.04E-12	1.99E-11
KIAA1383	-0.059869544	0.250016392	0.318182354
KIAA1407	0.072287151	0.164690276	0.221581663
KIAA1409	-0.11309797	0.029401393	0.048572398
KIAA1429	0.138534755	0.007534219	0.014278607
KIAA1430	-0.095634709	0.065759711	0.099382867
KIAA1432	-0.174013219	0.000762287	0.001783264
KIAA1462	0.03690843	0.478480167	0.552808282
KIAA1467	-0.092026316	0.076672377	0.113718514
KIAA1468	0.056840194	0.274829572	0.345174299
KIAA1486	0.069376501	0.182405977	0.241775907
KIAA1522	0.418766597	3.48E-17	7.18E-16
KIAA1524	0.582330145	4.62E-35	2.30E-32
KIAA1529	0.062029161	0.233307401	0.299829057
KIAA1530	0.145605478	0.004952823	0.009770288
KIAA1539	0.050701009	0.330105482	0.404102335
KIAA1543	0.030756203	0.554824018	0.625676673
KIAA1549	0.128943437	0.012932247	0.023216746
KIAA1586	0.162411405	0.001697736	0.00370047
KIAA1598	0.074411639	0.152597501	0.20737022
KIAA1609	0.329494047	7.63E-11	5.87E-10
KIAA1614	0.344923664	8.37E-12	7.47E-11
KIAA1632	-0.090473123	0.081801873	0.12040317
KIAA1644	0.129264004	0.012707558	0.022850436
KIAA1671	-0.071701416	0.168147479	0.225593243
KIAA1683	-0.195936357	0.000145784	0.000390205
KIAA1704	-0.159105817	0.002113111	0.004526602
KIAA1712	-0.097862136	0.05968419	0.091197149
KIAA1715	-0.243412556	2.09E-06	7.78E-06
KIAA1731	0.320705579	2.55E-10	1.80E-09
KIAA1737	-0.333448521	4.38E-11	3.50E-10
KIAA1751	0.260690484	3.53E-07	1.50E-06
KIAA1755	0.098245426	0.05868702	0.089873381
KIAA1797	0.32697844	1.08E-10	8.11E-10
KIAA1804	0.208181476	5.33E-05	0.000154684
KIAA1826	0.085387425	0.100563432	0.144505531
KIAA1841	0.483352649	4.06E-23	1.94E-21
KIAA1875	0.071845817	0.167290174	0.22465486
KIAA1908	-0.152517082	0.003229326	0.00663312
KIAA1919	0.045242145	0.384885618	0.461075944
KIAA1949	0.44396864	2.37E-19	6.64E-18
KIAA1958	-0.138781125	0.007427154	0.014091787
KIAA1967	0.151355517	0.003474244	0.00708723
KIAA1984	0.22403413	1.32E-05	4.28E-05
KIAA2013	-0.269704108	1.32E-07	6.04E-07
KIAA2018	-0.292395425	9.53E-09	5.29E-08
KIAA2022	0.147991757	0.004281385	0.00856535
KIAA2026	-0.011140868	0.830647832	0.865580401
KIDINS220	0.029613415	0.569631312	0.639226891
KIF11	0.562361891	2.54E-32	5.74E-30
KIF12	0.297874085	4.87E-09	2.82E-08

KIF13A	-0.190781939	0.000218742	0.000566461
KIF13B	-0.012769281	0.806352873	0.845304249
KIF14	0.518365622	6.69E-27	5.85E-25
KIF15	0.531489365	1.95E-28	2.14E-26
KIF16B	-0.125849309	0.015287328	0.026939775
KIF17	-0.047801257	0.358548082	0.433934444
KIF18A	0.555267377	2.15E-31	4.09E-29
KIF18B	0.578346453	1.68E-34	7.62E-32
KIF19	0.022659425	0.663537462	0.72468578
KIF1A	-0.02350479	0.651795806	0.714565047
KIF1B	-0.180827209	0.000465047	0.001131769
KIF1C	-0.132053985	0.010892909	0.019910867
KIF20A	0.627374155	5.47E-42	2.18E-38
KIF20B	0.418905975	3.39E-17	7.01E-16
KIF21A	-0.035034913	0.501106698	0.574630038
KIF21B	0.218497125	2.18E-05	6.76E-05
KIF22	0.093396595	0.072366715	0.108097
KIF23	0.592147908	1.77E-36	1.35E-33
KIF24	0.337532499	2.45E-11	2.02E-10
KIF25	0.020460609	0.694459522	0.750932739
KIF26A	-0.00795536	0.878621927	0.904360942
KIF26B	0.304731446	2.06E-09	1.27E-08
KIF27	-0.091847857	0.077248128	0.114487219
KIF2A	0.383859051	1.80E-14	2.41E-13
KIF2B	-0.11718952	0.023983343	0.040498064
KIF2C	0.606906981	1.05E-38	1.17E-35
KIF3A	0.275775833	6.70E-08	3.21E-07
KIF3B	-0.197958168	0.000123977	0.000336427
KIF3C	0.388196137	8.61E-15	1.22E-13
KIF4A	0.558437875	8.33E-32	1.77E-29
KIF4B	0.370029337	1.75E-13	2.03E-12
KIF5A	0.257057859	5.19E-07	2.14E-06
KIF5B	0.225344527	1.17E-05	3.83E-05
KIF5C	0.195556496	0.000150263	0.000401385
KIF6	0.164396164	0.001485745	0.003280759
KIF7	0.211349202	4.07E-05	0.000120779
KIF9	-0.013305425	0.798393263	0.83876974
KIFAP3	-0.059831478	0.250318191	0.318546087
KIFC1	0.548571431	1.55E-30	2.37E-28
KIFC2	0.269632065	1.34E-07	6.09E-07
KIFC3	0.123449209	0.017365242	0.030219282
KILLIN	-0.279947403	4.16E-08	2.07E-07
KIN	0.369495315	1.91E-13	2.20E-12
KIR2DL1	-0.036307403	0.485677644	0.559772637
KIR2DL3	0.070008475	0.178444612	0.237518944
KIR2DL4	0.116108617	0.025323478	0.042501629
KIR2DS4	-0.038497857	0.459729137	0.534628677
KIR3DL1	-0.057172454	0.272029075	0.342045142
KIR3DL2	0.022527173	0.66538193	0.726113644
KIR3DL3	0.06696422	0.198124137	0.260237954
KIR3DP1	0.013381106	0.79727136	0.838033031
KIR3DX1	0.015396512	0.767555017	0.812671718
KIRREL2	0.262902384	2.79E-07	1.20E-06
KIRREL3	0.253107049	7.84E-07	3.13E-06
KIRREL	0.091410246	0.078674825	0.116385228
KISS1R	0.424465604	1.17E-17	2.57E-16
KISS1	-0.028533604	0.583792861	0.652070048
KITLG	0.13016109	0.012096967	0.021882846

KIT	0.065695239	0.206778339	0.270073957
KLB	0.003658789	0.944006073	0.95673241
KLC1	0.161575763	0.001795009	0.003897567
KLC2	0.501542727	4.99E-25	3.30E-23
KLC3	0.360172269	8.33E-13	8.63E-12
KLC4	-0.3930485	3.73E-15	5.61E-14
KLF10	-0.11545168	0.026169109	0.043773383
KLF11	-0.21972851	1.95E-05	6.11E-05
KLF12	-0.445822481	1.62E-19	4.62E-18
KLF13	-0.065904159	0.205335055	0.268523367
KLF14	0.12346448	0.017351278	0.030200258
KLF15	-0.359963911	8.61E-13	8.88E-12
KLF16	0.217840072	2.31E-05	7.13E-05
KLF17	0.153052232	0.003121851	0.006442254
KLF1	0.069679361	0.180499548	0.239743449
KLF2	0.049420385	0.342482391	0.416718635
KLF3	-0.126785707	0.014537773	0.025762455
KLF4	0.152286188	0.003276725	0.006719394
KLF5	0.332821725	4.79E-11	3.79E-10
KLF6	-0.124919837	0.016064637	0.028167549
KLF7	0.077809979	0.134669985	0.186131222
KLF8	0.050071568	0.336152385	0.410118673
KLF9	-0.458712014	1.05E-20	3.54E-19
KLHDC10	-0.323294252	1.79E-10	1.30E-09
KLHDC1	-0.379177877	3.94E-14	5.04E-13
KLHDC2	-0.509583423	6.55E-26	5.00E-24
KLHDC3	-0.029942178	0.565352287	0.635212242
KLHDC4	-0.000296332	0.995461268	0.996461631
KLHDC5	0.346063469	7.07E-12	6.37E-11
KLHDC7A	-0.169629325	0.001037821	0.002360484
KLHDC7B	0.201745935	9.11E-05	0.00025339
KLHDC8A	0.491750589	5.51E-24	3.01E-22
KLHDC8B	0.103956555	0.045391064	0.071609184
KLHDC9	-0.147435249	0.004430167	0.008828427
KLHL10	-0.210657998	4.32E-05	0.000127586
KLHL11	-0.129578717	0.012490327	0.02251876
KLHL12	0.029782494	0.567428739	0.637078187
KLHL13	-0.146270494	0.004756765	0.009422719
KLHL14	0.092526916	0.075075895	0.111599909
KLHL15	-0.339000351	1.98E-11	1.67E-10
KLHL17	0.287941774	1.63E-08	8.68E-08
KLHL18	0.26141104	3.27E-07	1.40E-06
KLHL1	0.054918186	0.29141353	0.363118611
KLHL20	-0.233227117	5.63E-06	1.95E-05
KLHL21	-0.186434831	0.000305518	0.000769862
KLHL22	0.131923812	0.010972179	0.020041052
KLHL23	0.061919397	0.234137145	0.300763012
KLHL24	-0.210131341	4.52E-05	0.000133108
KLHL25	-0.009144213	0.860655887	0.890654334
KLHL26	-0.25667279	5.40E-07	2.22E-06
KLHL28	-0.185887214	0.000318485	0.000799402
KLHL29	0.382801636	2.15E-14	2.85E-13
KLHL2	-0.442652982	3.11E-19	8.56E-18
KLHL30	0.20460635	7.19E-05	0.000203928
KLHL31	-0.101873657	0.049913157	0.077892051
KLHL32	-0.231231503	6.80E-06	2.32E-05
KLHL33	0.052457228	0.313607194	0.386470526
KLHL34	-0.019316442	0.710756684	0.764934075

KLHL35	0.256820229	5.32E-07	2.19E-06
KLHL36	-0.161843995	0.001763243	0.003831515
KLHL38	0.321594462	2.26E-10	1.61E-09
KLHL3	0.1449061	0.005166835	0.01014125
KLHL4	-0.00862178	0.868542663	0.896534038
KLHL5	0.184631451	0.000350181	0.000872257
KLHL6	0.234339566	5.06E-06	1.77E-05
KLHL7	-0.057031015	0.273218823	0.343367739
KLHL8	-0.285587169	2.15E-08	1.13E-07
KLHL9	0.016089791	0.757408664	0.804151322
KLK10	0.145083516	0.005111771	0.010047031
KLK11	0.145432054	0.005005133	0.009857994
KLK12	0.136392192	0.008524992	0.015960426
KLK13	0.227522056	9.61E-06	3.19E-05
KLK14	0.064972651	0.211826719	0.275873636
KLK15	-0.013247954	0.799245499	0.839443739
KLK1	0.273434097	8.73E-08	4.10E-07
KLK2	-0.026006464	0.617558209	0.682701901
KLK3	-0.018340442	0.724763727	0.776722459
KLK4	-0.141289988	0.006411838	0.012341704
KLK5	0.021705105	0.676891878	0.735681396
KLK6	0.247511677	1.39E-06	5.32E-06
KLK7	0.115571422	0.026013185	0.04355608
KLK8	0.141278574	0.006416161	0.012347638
KLK9	0.059234684	0.255082883	0.323926644
KLKB1	-0.501790297	4.69E-25	3.11E-23
KLKP1	-0.178639196	0.000546081	0.001313137
KLRA1	0.253401683	7.60E-07	3.05E-06
KLRAQ1	0.158117672	0.0022542	0.00479942
KLRB1	0.07264916	0.162580396	0.219216694
KLRC1	0.162753677	0.001659307	0.003622256
KLRC2	0.171789584	0.000892249	0.002056622
KLRC3	0.071381754	0.170056973	0.227909521
KLRC4	0.107942058	0.037693667	0.060588448
KLRD1	-0.095906718	0.064991599	0.098356323
KLRF1	-0.144551367	0.005278535	0.010334018
KLRG1	0.101758149	0.050174566	0.078281596
KLRG2	0.14463896	0.005250753	0.010286705
KLRK1	0.030971459	0.552056035	0.623343337
KL	-0.202097481	8.85E-05	0.000246834
KMO	-0.102069051	0.049473542	0.077321088
KNCN	0.083721059	0.107404977	0.152979335
KNDC1	0.21348848	3.38E-05	0.000101666
KNG1	-0.392381437	4.18E-15	6.24E-14
KNTC1	0.52544403	1.01E-27	1.02E-25
KPNA1	-0.044698244	0.390631419	0.467032295
KPNA2	0.609851849	3.67E-39	4.57E-36
KPNA3	-0.035617708	0.494008325	0.56792855
KPNA4	0.244205055	1.93E-06	7.24E-06
KPNA5	0.137820975	0.00785223	0.01482913
KPNA6	-0.017001697	0.744126018	0.792964885
KPNA7	0.246942577	1.47E-06	5.61E-06
KPNB1	0.345421023	7.78E-12	6.97E-11
KPRP	0.17188175	0.000886479	0.002045257
KPTN	0.225564065	1.15E-05	3.76E-05
KRAS	0.168228537	0.001143596	0.002583368
KRBA1	0.380136672	3.36E-14	4.32E-13
KRBA2	-0.035059713	0.500803539	0.574414653

KRCC1	-0.295244431	6.74E-09	3.83E-08
KREMEN1	0.093138792	0.073161365	0.109144879
KREMEN2	0.396655156	1.98E-15	3.10E-14
KRI1	0.158099234	0.002256912	0.004804168
KRIT1	-0.077738504	0.135029567	0.186524686
KRR1	0.071110767	0.171688346	0.229755843
KRT10	0.268078883	1.58E-07	7.14E-07
KRT12	-0.013655875	0.79320167	0.834636015
KRT13	0.108159776	0.037307153	0.060037678
KRT14	0.075577687	0.146252809	0.199893556
KRT15	0.279112321	4.58E-08	2.26E-07
KRT16	0.156551203	0.002495557	0.005269368
KRT17	0.22289788	1.47E-05	4.70E-05
KRT18	0.180677858	0.000470201	0.001143336
KRT19	0.37917108	3.94E-14	5.04E-13
KRT1	-0.061860877	0.23458037	0.301270638
KRT20	0.077210736	0.137707627	0.18973728
KRT222	0.083033795	0.110331439	0.156587798
KRT23	0.284877221	2.34E-08	1.22E-07
KRT24	0.040879321	0.43241693	0.508446561
KRT25	0.217413346	2.40E-05	7.37E-05
KRT27	-0.079597035	0.125917099	0.175654397
KRT28	0.093083119	0.073333904	0.10936075
KRT2	0.066943995	0.198259964	0.260399196
KRT31	0.037145658	0.475655359	0.550322671
KRT32	0.105911643	0.041464399	0.066057878
KRT33B	0.047305756	0.363557494	0.439090172
KRT34	-0.028322525	0.586580098	0.654486066
KRT35	-0.079366654	0.127020059	0.176982348
KRT36	0.220500976	1.82E-05	5.73E-05
KRT37	0.055900237	0.282858029	0.353763429
KRT38	0.189476757	0.000242012	0.000621149
KRT39	0.200390139	0.000101804	0.00028075
KRT3	0.133908754	0.009817254	0.018163013
KRT40	0.200306015	0.000102504	0.000282407
KRT4	0.145959557	0.004847547	0.00958349
KRT5	0.10505162	0.043154917	0.068395566
KRT6A	0.064318005	0.216476479	0.281210512
KRT6B	0.233012944	5.74E-06	1.99E-05
KRT6C	0.260489293	3.61E-07	1.53E-06
KRT71	0.096828855	0.062442299	0.094914526
KRT72	-0.003856243	0.940989604	0.954549361
KRT73	0.017886708	0.731307188	0.782401686
KRT74	0.041421628	0.426330822	0.502684815
KRT75	0.006833266	0.895636563	0.91770157
KRT76	0.124768815	0.016194149	0.028372161
KRT77	0.17392865	0.000766891	0.001792561
KRT78	0.120947186	0.019789948	0.033996322
KRT79	0.236070528	4.29E-06	1.52E-05
KRT7	0.201556707	9.25E-05	0.000257102
KRT80	0.344834458	8.48E-12	7.56E-11
KRT81	0.091238581	0.079240287	0.117117367
KRT82	0.009283661	0.858553061	0.888959502
KRT83	0.11112001	0.032376544	0.052934387
KRT84	-0.04232241	0.416332411	0.492294295
KRT85	0.157149505	0.002400722	0.005086917
KRT86	0.074702409	0.150996175	0.205544639
KRT8	0.193623139	0.000175129	0.000461863

KRT9	0.179981199	0.000494954	0.00119928
KRTAP1-1	0.109786237	0.034524656	0.056028038
KRTAP1-3	0.054588567	0.294323562	0.366217851
KRTAP1-5	0.030815776	0.554057291	0.625135693
KRTAP10-1(	0.043178408	0.406960022	0.483277155
KRTAP10-1:	0.013029297	0.802490124	0.84191944
KRTAP10-1	0.023577671	0.650787489	0.713616708
KRTAP10-2	0.05087151	0.328479659	0.402558744
KRTAP10-3	-0.006448823	0.901477402	0.922547532
KRTAP10-4	0.038198969	0.463223639	0.538158463
KRTAP10-5	-0.002539104	0.961125187	0.969446406
KRTAP10-6	-0.240180706	2.88E-06	1.05E-05
KRTAP10-7	0.0153604	0.768084628	0.813118049
KRTAP11-1	0.029829223	0.566820711	0.636467264
KRTAP12-1	0.041172272	0.429123052	0.505528321
KRTAP12-2	-0.09558904	0.0658894	0.099563761
KRTAP12-3	0.048248057	0.35406829	0.428922847
KRTAP13-2	0.098404255	0.058277865	0.089329201
KRTAP17-1	0.034769108	0.504361949	0.577731069
KRTAP19-1	0.204807566	7.07E-05	0.000200798
KRTAP19-3	0.132604041	0.010563518	0.019374554
KRTAP19-4	0.089771329	0.084208806	0.123507901
KRTAP19-5	0.176095142	0.000656696	0.001556908
KRTAP19-8	-0.010003991	0.847706915	0.87967586
KRTAP2-1	0.065987616	0.204760552	0.267861509
KRTAP2-2	0.060207658	0.247346831	0.315349006
KRTAP20-2	0.03322699	0.523464862	0.595538061
KRTAP20-4	0.126510369	0.014754749	0.026098207
KRTAP26-1	0.035088988	0.50044581	0.574136564
KRTAP3-1	0.128426233	0.013302145	0.023803586
KRTAP3-2	0.133068716	0.010292163	0.018934387
KRTAP3-3	0.085199023	0.101319158	0.145444608
KRTAP4-11	0.078723594	0.130138367	0.180693053
KRTAP4-12	0.081822124	0.115643299	0.163115676
KRTAP4-1	0.238202661	3.49E-06	1.25E-05
KRTAP4-2	-0.006278276	0.904070241	0.924631004
KRTAP4-4	0.023486113	0.652054307	0.714730449
KRTAP4-7	0.059189735	0.255444277	0.324282221
KRTAP4-8	0.114890225	0.026910988	0.044893711
KRTAP5-10	0.133074417	0.010288872	0.018930081
KRTAP5-11	0.098745732	0.057406188	0.088162664
KRTAP5-1	0.161935163	0.001752564	0.00381122
KRTAP5-2	0.11189596	0.031180107	0.051146159
KRTAP5-3	-0.000553631	0.991520481	0.993315449
KRTAP5-4	0.158310039	0.002226078	0.004744615
KRTAP5-5	0.366299324	3.18E-13	3.54E-12
KRTAP5-6	-0.119547937	0.02127035	0.036329553
KRTAP5-7	0.153025626	0.003127117	0.006452451
KRTAP5-8	-0.044069526	0.397337512	0.473628787
KRTAP5-9	-0.003781973	0.942124123	0.955408337
KRTAP6-3	0.109740758	0.034599991	0.056136576
KRTAP7-1	0.026809055	0.606742492	0.672687624
KRTAP8-1	0.034543106	0.507138461	0.580144268
KRTCAP2	0.182591675	0.000407996	0.001002601
KRTCAP3	0.129617611	0.012463709	0.022478908
KRTDAP	0.10571243	0.041850932	0.066577839
KSR1	0.266293695	1.93E-07	8.57E-07
KSR2	0.241375186	2.56E-06	9.40E-06

KTELC1	-0.118943394	0.021939103	0.037360372
KTI12	0.253072795	7.86E-07	3.14E-06
KTN1	-0.091872649	0.077167933	0.114376874
KYNU	0.156995922	0.002424748	0.005134002
KY	0.226185518	1.09E-05	3.57E-05
L1CAM	0.233437989	5.52E-06	1.92E-05
L1TD1	0.245823966	1.65E-06	6.22E-06
L2HGDH	-0.348577332	4.87E-12	4.49E-11
L3MBTL2	-0.194842055	0.000159038	0.000422843
L3MBTL3	0.188617357	0.000258575	0.00065975
L3MBTL4	-0.063139993	0.22502695	0.290631242
L3MBTL	0.314428332	5.88E-10	3.93E-09
LACE1	-0.014853705	0.775527047	0.819327031
LACTB2	-0.151280879	0.003490543	0.007116106
LACTB	-0.275313241	7.06E-08	3.37E-07
LAD1	0.307944957	1.37E-09	8.65E-09
LAG3	0.194208517	0.00016722	0.000442823
LAGE3	0.140729688	0.006627131	0.012713115
LAIR1	0.355396891	1.74E-12	1.72E-11
LAIR2	0.287476268	1.72E-08	9.13E-08
LALBA	-0.004408393	0.932559114	0.94758965
LAMA1	0.27072082	1.18E-07	5.44E-07
LAMA2	0.060207887	0.24734503	0.315349006
LAMA3	-0.068922217	0.185293402	0.245179008
LAMA4	0.184683984	0.000348798	0.000869356
LAMA5	0.166958112	0.001248019	0.002796114
LAMB1	0.355579089	1.69E-12	1.68E-11
LAMB2L	0.072074952	0.16593653	0.223002399
LAMB2	-0.214714387	3.04E-05	9.20E-05
LAMB3	0.005942845	0.909172778	0.928610105
LAMB4	0.04375191	0.400751425	0.476927711
LAMC1	0.21124563	4.10E-05	0.000121731
LAMC2	0.315531211	5.08E-10	3.43E-09
LAMC3	0.01110397	0.831200282	0.866017028
LAMP1	-0.171435907	0.000914713	0.002101835
LAMP2	-0.2003865	0.000101834	0.000280794
LAMP3	0.358089233	1.15E-12	1.17E-11
LANCL1	-0.036736882	0.480528567	0.554800933
LANCL2	-0.152970888	0.003137975	0.006472846
LANCL3	0.205017793	6.95E-05	0.000197481
LAP3	-0.249464574	1.14E-06	4.44E-06
LAPTM4A	-0.30197064	2.92E-09	1.76E-08
LAPTM4B	0.414768447	7.38E-17	1.45E-15
LAPTM5	0.336634947	2.79E-11	2.28E-10
LARGE	-0.189888009	0.000234441	0.000603117
LARP1B	-0.333288684	4.48E-11	3.57E-10
LARP1	0.288595117	1.51E-08	8.09E-08
LARP4B	0.492478796	4.62E-24	2.56E-22
LARP4	-0.400510156	1.00E-15	1.64E-14
LARP6	0.302755748	2.65E-09	1.60E-08
LARP7	-0.222047528	1.59E-05	5.04E-05
LARS2	-0.069950283	0.178806685	0.237875578
LARS	0.366489935	3.09E-13	3.44E-12
LAS1L	0.094181503	0.069990561	0.105012197
LASP1	0.398153204	1.52E-15	2.42E-14
LASS1	0.408710926	2.27E-16	4.12E-15
LASS2	-0.160023481	0.001989357	0.004282686
LASS3	0.162345361	0.001705244	0.003716023

LASS4	-0.20995688	4.58E-05	0.000134707
LASS5	0.50934399	6.96E-26	5.23E-24
LASS6	0.210637773	4.32E-05	0.000127769
LAT2	0.337389758	2.50E-11	2.06E-10
LATS1	0.015177765	0.770764794	0.81541148
LATS2	-0.197577202	0.000127836	0.000345932
LAT	0.246970328	1.47E-06	5.60E-06
LAX1	0.191656101	0.000204346	0.000532363
LAYN	0.093123283	0.073209399	0.109200183
LBH	0.302412417	2.76E-09	1.67E-08
LBP	0.028937558	0.578475976	0.647547249
LBR	0.315221547	5.29E-10	3.56E-09
LBX1	0.11650786	0.024821151	0.041767629
LBX2	-0.323895883	1.65E-10	1.20E-09
LBXCOR1	-0.153381554	0.003057338	0.006315666
LCA5L	-0.112735327	0.029928634	0.049341132
LCA5	0.09466323	0.068564073	0.103190562
LCAT	-0.298267888	4.64E-09	2.70E-08
LCE1B	0.131893316	0.010990823	0.020067747
LCE1C	0.208168044	5.34E-05	0.000154838
LCE1D	0.004487674	0.931349209	0.946746553
LCE1E	0.240433905	2.81E-06	1.02E-05
LCE1F	0.173134845	0.000811377	0.001887465
LCE2A	0.180898472	0.000462606	0.001126241
LCE2B	-0.034511069	0.50753269	0.580495249
LCE2C	0.00430105	0.934197515	0.949060831
LCE2D	-0.007695863	0.8825521	0.907421704
LCE3A	0.074000552	0.154883351	0.210075307
LCE3C	-0.032018135	0.538693823	0.610481059
LCE3D	0.037117095	0.475994993	0.550587717
LCE3E	-0.0307149	0.555355905	0.626099264
LCE5A	-0.02342272	0.652932008	0.715461008
LCE6A	0.161064558	0.001857004	0.004022096
LCK	0.207603488	5.60E-05	0.000161862
LCLAT1	0.100353125	0.05344685	0.08277759
LCMT1	0.407218104	2.98E-16	5.31E-15
LCMT2	-0.097380503	0.060957014	0.092878442
LCN10	0.10818922	0.03725514	0.059975507
LCN12	-0.150546413	0.003654665	0.007422595
LCN15	0.077835651	0.134541008	0.186004577
LCN1	0.198229707	0.000121293	0.000329884
LCN2	0.106665937	0.040028014	0.063989576
LCN6	-0.100746378	0.052513559	0.081504621
LCN8	0.015277561	0.769299944	0.814216303
LCNL1	0.237457677	3.75E-06	1.34E-05
LCORL	0.079722811	0.125318082	0.174953527
LCOR	-0.057349547	0.270544396	0.340500692
LCP1	0.182593074	0.000407953	0.001002601
LCP2	0.227995111	9.20E-06	3.06E-05
LCTL	0.174663358	0.000727737	0.001708257
LCT	0.126846339	0.014490372	0.025685309
LDB1	0.219362972	2.02E-05	6.29E-05
LDB2	-0.22934277	8.11E-06	2.73E-05
LDB3	-0.031160886	0.549625772	0.620798539
LDHAL6A	-0.005297202	0.919004542	0.936648179
LDHAL6B	0.090779978	0.080767067	0.119047167
LDHA	0.187558224	0.000280444	0.000711629
LDHB	0.16943981	0.001051584	0.00239015

LDHC	-0.060870902	0.242168127	0.3095985
LDHD	-0.537986505	3.21E-29	4.04E-27
LDLRAD1	0.103728839	0.045868079	0.072275874
LDLRAD2	0.082337263	0.113361006	0.160384772
LDLRAD3	0.130545346	0.01184345	0.021463223
LDLRAP1	-0.171225329	0.000928335	0.002131168
LDLR	-0.265318691	2.14E-07	9.46E-07
LDOC1L	0.241650343	2.49E-06	9.16E-06
LDOC1	0.182364892	0.000414945	0.001018296
LEAP2	-0.304118344	2.23E-09	1.36E-08
LECT1	0.022873782	0.660552212	0.722138022
LECT2	-0.328362588	8.93E-11	6.79E-10
LEF1	0.125622691	0.015473743	0.027246588
LEFTY1	0.39992494	1.11E-15	1.81E-14
LEFTY2	0.174934482	0.000713762	0.001679161
LEKR1	-0.209866134	4.62E-05	0.000135649
LELP1	0.023162152	0.656544711	0.718663941
LEMD1	0.158105244	0.002256028	0.004802799
LEMD2	0.293933852	7.91E-09	4.45E-08
LEMD3	0.070781532	0.173686044	0.232023963
LENEP	0.08116254	0.118617888	0.166779983
LENG1	0.039009658	0.453779618	0.529347555
LENG8	0.237283916	3.82E-06	1.36E-05
LENG9	0.046453545	0.372274567	0.448097029
LEO1	0.125202186	0.015824931	0.027798631
LEPRE1	0.252345801	8.48E-07	3.37E-06
LEPREL1	0.091488877	0.07841691	0.116055396
LEPREL2	0.266100206	1.97E-07	8.73E-07
LEPROTL1	0.338843568	2.03E-11	1.70E-10
LEPROT	-0.218616986	2.16E-05	6.69E-05
LEPR	-0.32491726	1.44E-10	1.05E-09
LEP	0.103457304	0.046442376	0.073105642
LETM1	0.196103323	0.000143855	0.000385664
LETM2	0.27462792	7.63E-08	3.62E-07
LETMD1	-0.08131644	0.11791854	0.165948937
LEUTX	0.109653499	0.034744924	0.056353365
LFNG	0.274473478	7.77E-08	3.68E-07
LGALS12	0.111120594	0.032375628	0.052934387
LGALS13	0.067236281	0.196303591	0.258101909
LGALS14	0.256793044	5.34E-07	2.20E-06
LGALS1	0.275799734	6.69E-08	3.21E-07
LGALS2	0.327767639	9.70E-11	7.32E-10
LGALS3BP	0.398416401	1.45E-15	2.32E-14
LGALS3	0.376807114	5.82E-14	7.27E-13
LGALS4	0.170641351	0.000967096	0.002213775
LGALS7B	0.20943741	4.79E-05	0.000140309
LGALS7	0.206726839	6.03E-05	0.000173382
LGALS8	-0.101467587	0.050837206	0.079185209
LGALS9B	0.193751954	0.000173359	0.000457438
LGALS9C	0.292400325	9.53E-09	5.29E-08
LGALS9	0.407428168	2.87E-16	5.14E-15
LGI1	-0.061751664	0.235409112	0.302155444
LGI2	0.3477369	5.52E-12	5.04E-11
LGI3	-0.141784163	0.006227178	0.012017615
LGI4	0.122567023	0.018188797	0.031506583
LGMN	-0.007031941	0.892620295	0.915222929
LGR4	0.026911412	0.605369221	0.671613612
LGR5	-0.12978779	0.012347831	0.022286056

LGR6	0.141810944	0.006217308	0.01199973
LGSN	-0.409399002	2.00E-16	3.67E-15
LGTN	0.016656839	0.749140487	0.79724235
LHB	0.185194274	0.000335628	0.000838944
LHCGR	0.055353343	0.287601353	0.359087124
LHFPL1	0.029952995	0.565211759	0.635090167
LHFPL2	0.434601273	1.59E-18	4.01E-17
LHFPL3	0.312298684	7.77E-10	5.10E-09
LHFPL4	0.181135572	0.00045457	0.001108167
LHFPL5	0.298143322	4.71E-09	2.74E-08
LHFP	0.105126952	0.043004558	0.068173534
LHPP	-0.409561849	1.94E-16	3.57E-15
LHX1	0.105455347	0.042354235	0.06726571
LHX2	0.167713239	0.001184942	0.002667391
LHX3	-0.299576815	3.94E-09	2.32E-08
LHX4	0.326784284	1.11E-10	8.31E-10
LHX5	0.094652374	0.068595958	0.103215157
LHX6	0.018735981	0.719075778	0.772218622
LHX8	0.133063779	0.010295013	0.018937881
LHX9	-0.113757645	0.028462694	0.047187847
LIAS	-0.260733762	3.51E-07	1.49E-06
LIFR	-0.082687174	0.111831009	0.158456426
LIF	0.360906358	7.43E-13	7.77E-12
LIG1	0.448725951	8.83E-20	2.62E-18
LIG3	0.106836007	0.039710026	0.063516912
LIG4	-0.108983028	0.035875872	0.057970567
LILRA1	0.060976168	0.241353224	0.30871518
LILRA2	0.084400079	0.104574091	0.149481599
LILRA3	0.060876766	0.242122681	0.309560265
LILRA4	0.119656739	0.021151888	0.036148916
LILRA5	0.052398159	0.314153163	0.387023642
LILRA6	0.172296191	0.000860958	0.001991919
LILRB1	0.168335333	0.001135195	0.002565845
LILRB2	0.143222354	0.005716592	0.011102159
LILRB3	0.254719743	6.63E-07	2.69E-06
LILRB4	0.278994473	4.64E-08	2.29E-07
LILRB5	-0.205967332	6.42E-05	0.000183835
LILRP2	0.192522224	0.00019096	0.000499974
LIM2	-0.005475908	0.916281936	0.934529705
LIMA1	0.201776626	9.09E-05	0.000252859
LIMCH1	0.175405	0.000690096	0.001628343
LIMD1	-0.054605723	0.294171629	0.366051667
LIMD2	0.38836101	8.37E-15	1.19E-13
LIME1	-0.414877016	7.24E-17	1.43E-15
LIMK1	0.478323384	1.31E-22	5.90E-21
LIMK2	0.294800352	7.11E-09	4.04E-08
LIMS1	0.014578404	0.779579387	0.82277922
LIMS2	-0.21774636	2.33E-05	7.19E-05
LIMS3-LOC	-0.03565108	0.49360349	0.567594154
LIMS3	0.29199671	1.00E-08	5.53E-08
LIN28A	0.153938115	0.002951056	0.006115774
LIN28B	0.117421266	0.023704128	0.040084343
LIN37	0.096151703	0.064306125	0.097459613
LIN52	-0.135908879	0.008763919	0.016367751
LIN54	-0.060916353	0.241816036	0.309227747
LIN7A	-0.202358585	8.66E-05	0.000242133
LIN7B	-0.064268567	0.216830569	0.281523632
LIN7C	-0.257493564	4.96E-07	2.05E-06

LIN9	0.379040528	4.03E-14	5.14E-13
LINGO1	0.346607953	6.53E-12	5.92E-11
LINGO2	0.017223415	0.740907774	0.790465628
LINGO3	0.007923734	0.879100756	0.90465678
LINGO4	-0.256271284	5.64E-07	2.31E-06
LINS1	-0.005657206	0.913520841	0.932381894
LIPA	-0.065319717	0.209390967	0.273083154
LIPC	-0.357283646	1.30E-12	1.31E-11
LIPE	-0.217659515	2.35E-05	7.23E-05
LIPF	0.063992747	0.218813704	0.283765566
LIPG	-0.188994986	0.00025117	0.000642586
LIPH	0.335188942	3.42E-11	2.77E-10
LIPI	0.04447677	0.392985881	0.46939653
LIPJ	-0.19373929	0.000173532	0.000457834
LIPK	0.004177737	0.93608001	0.950779339
LIPM	-0.004713634	0.927901713	0.94381997
LIPN	-0.069559083	0.181254894	0.240586315
LIPT1	0.01052118	0.839937061	0.873250502
LIPT2	0.116437943	0.024908495	0.041882768
LITAF	0.094994929	0.067595754	0.10189487
LIX1L	0.140547162	0.00669865	0.012837952
LIX1	0.019571491	0.707112102	0.761752409
LLGL1	0.287604629	1.69E-08	9.01E-08
LLGL2	0.125456791	0.015611472	0.027455132
LLPH	0.307882149	1.38E-09	8.71E-09
LMAN1L	0.04923414	0.34430671	0.418591669
LMAN1	-0.219440455	2.00E-05	6.25E-05
LMAN2L	0.066008381	0.204617796	0.267708395
LMAN2	0.031751064	0.54208794	0.613536868
LMBR1L	0.173091805	0.000813856	0.001892568
LMBR1	-0.147095478	0.004523283	0.008995991
LMBRD1	-0.03197719	0.539213494	0.610943026
LMBRD2	-0.150671784	0.003626164	0.007369218
LMCD1	0.211098812	4.16E-05	0.000123183
LMF1	-0.262160226	3.02E-07	1.30E-06
LMF2	0.083168816	0.109751605	0.155853694
LMLN	-0.177194045	0.000606589	0.00144603
LMNA	0.228590792	8.70E-06	2.91E-05
LMNB1	0.61569765	4.38E-40	7.93E-37
LMNB2	0.564161148	1.46E-32	3.73E-30
LMO1	0.164914685	0.001434515	0.003178911
LMO2	-0.082248092	0.113753527	0.160825901
LMO3	0.119986891	0.020795922	0.035598587
LMO4	0.042868198	0.410341964	0.486568216
LMO7	-0.363393868	5.04E-13	5.42E-12
LMOD1	-0.324322581	1.56E-10	1.14E-09
LMOD2	-0.158909514	0.00214048	0.004580798
LMOD3	-0.00591394	0.909612663	0.92891652
LMTK2	-0.025263965	0.627638278	0.692424952
LMTK3	0.349336404	4.35E-12	4.06E-11
LMX1A	0.184220982	0.000361163	0.000897479
LMX1B	0.290088265	1.26E-08	6.87E-08
LNP1	-0.33481834	3.61E-11	2.91E-10
LNPEP	0.051196612	0.325393991	0.39948845
LNX1	-0.037874509	0.467033747	0.541921272
LNX2	-0.139048803	0.007312371	0.013901809
LOC1000096	0.103860224	0.045592345	0.071898258
LOC1001012	0.001776884	0.972789641	0.978388289

LOC1001019	0.079698971	0.125431453	0.17506273
LOC1001246	0.267887607	1.62E-07	7.28E-07
LOC1001255	0.124396483	0.016517365	0.02888509
LOC1001267	-0.058976001	0.25716755	0.326199665
LOC1001278	0.296187643	6.00E-09	3.43E-08
LOC1001280	0.103070876	0.047270056	0.074296967
LOC1001280	0.121543712	0.019186585	0.033065323
LOC1001281	0.088926606	0.0871813	0.127361286
LOC1001281	0.2885598	1.51E-08	8.11E-08
LOC1001282	0.142886701	0.00583227	0.011308076
LOC1001282	0.100585384	0.052893986	0.08202335
LOC1001282	0.029652423	0.569122801	0.638764193
LOC1001285	-0.247670546	1.37E-06	5.25E-06
LOC1001285	0.164765607	0.001449075	0.003207965
LOC1001285	-0.083910674	0.106608442	0.152030153
LOC1001286	-0.184062096	0.000365499	0.000907124
LOC1001286	-0.102297268	0.04896418	0.07665732
LOC1001287	-0.034181286	0.511600127	0.584375766
LOC1001288	0.110423399	0.033483801	0.054529902
LOC1001288	-0.119299165	0.021543372	0.036761204
LOC1001288	0.095201877	0.066997312	0.101038642
LOC1001289	0.009170482	0.860259686	0.890336821
LOC1001290	-0.053079879	0.307889935	0.380552355
LOC1001290	0.180966774	0.000460277	0.00112071
LOC1001290	-0.036379005	0.484817146	0.559007245
LOC1001293	-0.499687512	7.91E-25	4.99E-23
LOC1001295	-0.041969566	0.42023235	0.496287206
LOC1001295	0.099804617	0.054771602	0.084592593
LOC1001296	0.148475599	0.004155714	0.008328149
LOC1001297	0.256829936	5.32E-07	2.19E-06
LOC1001297	-0.072910459	0.161070141	0.217341377
LOC1001299	0.124485026	0.016439995	0.028764938
LOC1001300	-0.299860917	3.81E-09	2.25E-08
LOC1001300	-0.263064112	2.74E-07	1.19E-06
LOC1001301	-0.109507278	0.034988949	0.056693766
LOC1001302	0.063279773	0.223999991	0.289661678
LOC1001302	-0.0180432	0.729048134	0.780361966
LOC1001302	0.179937459	0.000496548	0.001202703
LOC1001303	0.17349091	0.000791135	0.001844255
LOC1001303	0.093589586	0.07177646	0.107319921
LOC1001305	-0.149543312	0.003890107	0.007855925
LOC1001305	0.111030572	0.032516932	0.053129035
LOC1001305	-0.27505367	7.28E-08	3.46E-07
LOC1001306	0.104961403	0.04333557	0.068643653
LOC1001307	0.250467248	1.03E-06	4.04E-06
LOC1001308	-0.150030341	0.003774132	0.007637979
LOC1001309	0.352960771	2.52E-12	2.44E-11
LOC1001309	0.13994043	0.00694138	0.013267407
LOC1001309	0.018373769	0.724283882	0.776346901
LOC1001311	-0.132404844	0.010681771	0.019569037
LOC1001314	-0.02377831	0.648014933	0.711006967
LOC1001314	-0.275742678	6.73E-08	3.22E-07
LOC1001315	0.034567744	0.506835384	0.579890627
LOC1001316	-0.176405404	0.000642174	0.001525747
LOC1001317	-0.060254843	0.246975873	0.314983841
LOC1001321	-0.061053792	0.240753549	0.308113193
LOC1001322	-0.057732253	0.267354871	0.337061369
LOC1001322	0.137060609	0.008204119	0.015422808

LOC1001322	0.024972566	0.631613405	0.696149633
LOC1001322	-0.017144456	0.742053355	0.791391164
LOC1001323	-0.197731191	0.000126263	0.000342139
LOC1001327	-0.231531715	6.61E-06	2.26E-05
LOC1001327	0.207917075	5.45E-05	0.000157837
LOC1001328	0.067915934	0.191808955	0.252792935
LOC1001328	-0.196002924	0.000145012	0.0003884
LOC1001330	-0.076398267	0.141909809	0.194759384
LOC1001331	0.127439662	0.01403363	0.024955635
LOC1001333	0.029855319	0.5664813	0.636229589
LOC1001333	0.003325035	0.949106586	0.96067988
LOC1001334	0.051103885	0.326272179	0.400356624
LOC1001335	0.066183995	0.20341331	0.266377438
LOC1001336	0.078601875	0.130735211	0.181410244
LOC1001336	0.117651009	0.023430105	0.039678217
LOC1001338	0.17797848	0.000573015	0.001372892
LOC1001339	0.077994086	0.133747153	0.185048322
LOC1001339	-0.151187769	0.003510972	0.007151901
LOC1001339	-0.290492031	1.20E-08	6.56E-08
LOC1001339	0.136030629	0.008703177	0.016268033
LOC1001342	0.002296099	0.964843179	0.97245805
LOC1001342	0.191207172	0.000211625	0.000549744
LOC1001343	0.147718103	0.004353973	0.008695744
LOC1001347	0.209771325	4.66E-05	0.000136615
LOC1001348	0.123856756	0.016995908	0.029649079
LOC1001446	0.005534067	0.915396084	0.93381744
LOC1001446	0.024878708	0.632896027	0.697150064
LOC1001709	0.145499071	0.00498486	0.009826675
LOC1001889	0.001316384	0.979839767	0.98438567
LOC1001889	0.033337571	0.522082842	0.594271191
LOC1001895	0.094593739	0.068768371	0.103427713
LOC1001909	-0.016291544	0.754463652	0.80160287
LOC1001909	0.094102773	0.070225984	0.105286127
LOC1001909	0.063058501	0.225627214	0.291217711
LOC1001909	-0.010716579	0.837005531	0.870975408
LOC1001923	-0.154345357	0.002875428	0.005974581
LOC1001923	0.140378798	0.006765233	0.012954341
LOC1001924	0.091455008	0.078527919	0.116202421
LOC1002160	0.156254007	0.002543923	0.005364101
LOC1002165	-0.175745853	0.000673409	0.00159255
LOC1002332	0.196961764	0.00013431	0.000361938
LOC1002407	0.034500765	0.507659514	0.580606972
LOC1002407	0.117165886	0.024011976	0.040539542
LOC1002407	0.157128215	0.00240404	0.005093404
LOC1002681	0.022115502	0.671136217	0.731180997
LOC1002707	0.32224397	2.07E-10	1.49E-09
LOC1002707	-0.033098888	0.525068185	0.597157526
LOC1002708	0.169710899	0.001031949	0.002350078
LOC1002717	-0.107668794	0.038183588	0.061291873
LOC1002718	0.179994405	0.000494474	0.001198262
LOC1002718	0.121258894	0.01947264	0.033520602
LOC1002718	0.024441592	0.638883961	0.702924077
LOC1002721	0.357658387	1.23E-12	1.24E-11
LOC1002722	0.108865234	0.036077762	0.058240107
LOC1002722	-0.117971203	0.023052766	0.039109019
LOC1002722	-0.000818378	0.987465843	0.990548567
LOC1002867	0.40886896	2.20E-16	4.01E-15
LOC1002868	-0.027057469	0.603412039	0.669910977

LOC1002872	-0.258636443	4.39E-07	1.83E-06
LOC1002877	-0.02682326	0.606551837	0.672551104
LOC1002877	0.117108515	0.024081607	0.040636437
LOC1002887	0.168398312	0.001130268	0.002554998
LOC1002893	-0.150100144	0.003757769	0.007608728
LOC1003024	-0.044023204	0.397834318	0.474164241
LOC1003026	0.014838004	0.775758	0.819527568
LOC1003026	-0.057152505	0.272196663	0.342234264
LOC1003037	-0.381204354	2.81E-14	3.67E-13
LOC113230	0.120397176	0.020360814	0.034910761
LOC115110	0.243390953	2.10E-06	7.79E-06
LOC116437	0.027843268	0.592931242	0.660499676
LOC121838	-0.055754705	0.284115071	0.355090366
LOC121952	-0.160042017	0.001986927	0.004277916
LOC126536	0.37959731	3.67E-14	4.71E-13
LOC127841	0.357241492	1.31E-12	1.32E-11
LOC134466	0.150507705	0.003663505	0.007439033
LOC143188	-0.078521266	0.131131633	0.181871651
LOC143666	-0.130272134	0.012023217	0.021757338
LOC144438	0.088427931	0.088975306	0.129601963
LOC144486	0.082513573	0.112588045	0.159393053
LOC144571	-0.05032971	0.333663937	0.407731887
LOC144742	-0.121620596	0.019109997	0.032950437
LOC144776	-0.034679685	0.505459584	0.57863364
LOC145474	0.085975064	0.098234982	0.141465759
LOC145783	0.133113117	0.01026656	0.018894264
LOC145820	-0.066433965	0.201707728	0.264428441
LOC145837	-0.008752834	0.866562991	0.895139889
LOC145845	0.086589065	0.0958481	0.138408658
LOC146336	0.307194563	1.51E-09	9.46E-09
LOC146481	0.060024659	0.248789213	0.316814716
LOC146880	0.331810943	5.52E-11	4.33E-10
LOC147727	-0.186344346	0.000307626	0.000774782
LOC147804	0.366122025	3.27E-13	3.64E-12
LOC148145	-0.028715421	0.581396958	0.650122934
LOC148189	-0.076469162	0.141539273	0.194331178
LOC148413	-0.24060359	2.76E-06	1.01E-05
LOC148696	-0.114703988	0.027161034	0.045261564
LOC148709	-0.198021911	0.000123342	0.000334816
LOC148824	0.102387204	0.048764657	0.076404993
LOC149134	0.083683215	0.107564512	0.15317371
LOC149620	0.079610165	0.12585446	0.175602757
LOC149837	0.246386744	1.56E-06	5.91E-06
LOC150185	0.031918591	0.539957652	0.611716596
LOC150197	0.214111839	3.20E-05	9.66E-05
LOC150381	0.27922321	4.52E-08	2.23E-07
LOC150527	-0.036020931	0.489128658	0.563293897
LOC150568	0.089568133	0.084916263	0.124453895
LOC150622	0.263911379	2.50E-07	1.09E-06
LOC150776	0.173324825	0.000800518	0.001864164
LOC150786	0.050424176	0.332756264	0.406880536
LOC151009	0.199044172	0.000113567	0.000310227
LOC151162	0.365163242	3.81E-13	4.18E-12
LOC151174	0.095766288	0.065387227	0.098894954
LOC151300	0.082328003	0.113401721	0.16041959
LOC151534	-0.413914467	8.66E-17	1.69E-15
LOC151658	0.068616005	0.18725859	0.247516297
LOC152024	0.144814575	0.005195449	0.010189382

LOC152217	0.028681884	0.581838551	0.650507189
LOC152225	0.113192212	0.029265687	0.048364248
LOC153328	0.151816355	0.003375128	0.006902711
LOC153684	0.06058558	0.244386572	0.312134202
LOC153910	0.085024842	0.102021837	0.146316251
LOC154449	0.123133192	0.017656434	0.030661739
LOC154761	0.247214075	1.43E-06	5.47E-06
LOC154822	0.086567465	0.095931277	0.138518729
LOC157381	-0.185692474	0.000323218	0.000810289
LOC157627	0.041984416	0.420067781	0.496126375
LOC158376	-0.348081406	5.24E-12	4.81E-11
LOC158572	0.163257604	0.001604176	0.00351119
LOC158696	0.095438068	0.066319623	0.100124415
LOC162632	0.026299248	0.613602904	0.67915979
LOC168474	0.094675304	0.068528629	0.103168393
LOC200030	0.011479496	0.825581582	0.861472674
LOC200726	0.152710013	0.003190198	0.006563578
LOC201651	-0.49503586	2.48E-24	1.43E-22
LOC202181	0.20724158	5.77E-05	0.000166469
LOC202781	-0.181214025	0.00045194	0.001102648
LOC219347	-0.188418935	0.000262547	0.000669284
LOC220429	-0.22005561	1.90E-05	5.95E-05
LOC220594	0.078488244	0.131294297	0.182084579
LOC220729	0.185463224	0.000328875	0.000823302
LOC220930	-0.038325812	0.461738837	0.536683846
LOC221122	0.010583888	0.838996026	0.872590908
LOC221442	-0.030135347	0.562845278	0.633038087
LOC221710	-0.026355416	0.612845397	0.678547546
LOC222699	0.003134393	0.952020963	0.962798194
LOC253039	0.103010685	0.047400083	0.074483708
LOC253724	-0.147203451	0.004493503	0.008943013
LOC254559	0.159749589	0.002025581	0.004355962
LOC255025	0.070130088	0.177689684	0.236689882
LOC255167	-0.310223514	1.02E-09	6.58E-09
LOC256880	0.295114426	6.84E-09	3.89E-08
LOC257358	0.205098014	6.91E-05	0.000196417
LOC25845	0.246033159	1.61E-06	6.11E-06
LOC26102	0.115202365	0.02649634	0.044276135
LOC282997	0.029213332	0.574859271	0.644077746
LOC283050	0.183428629	0.000383275	0.000947228
LOC283070	-0.16453813	0.001471554	0.003253763
LOC283174	0.102460098	0.048603443	0.0761734
LOC283267	0.205348373	6.76E-05	0.000192658
LOC283314	-0.209820922	4.64E-05	0.000136098
LOC283332	0.093585577	0.07178868	0.107330137
LOC283392	0.085837331	0.098776847	0.14217953
LOC283404	0.169834537	0.001023106	0.002331539
LOC283663	0.179420377	0.000515754	0.001245739
LOC283731	0.246765409	1.50E-06	5.71E-06
LOC283761	-0.024179077	0.642491423	0.706113209
LOC283856	-0.337359243	2.51E-11	2.07E-10
LOC283867	0.048035764	0.356192414	0.431266882
LOC283914	-0.056562092	0.277188614	0.347654972
LOC283922	-0.068771981	0.186255672	0.246354103
LOC283999	0.003581954	0.945180099	0.957629961
LOC284009	0.133376682	0.010115743	0.018647712
LOC284023	0.362185138	6.09E-13	6.45E-12
LOC284100	0.300776246	3.40E-09	2.02E-08

LOC284232	-0.017570492	0.73587902	0.785858045
LOC284233	0.160367184	0.001944738	0.004194788
LOC284276	0.265287598	2.15E-07	9.49E-07
LOC284379	-0.059982671	0.249120979	0.317204917
LOC284440	-0.304106882	2.23E-09	1.36E-08
LOC284441	0.036818566	0.479552621	0.553834627
LOC284551	0.036894246	0.478649361	0.552951729
LOC284578	0.101637141	0.050449651	0.078661496
LOC284632	0.073273099	0.158991684	0.214917934
LOC284661	0.008811631	0.865675082	0.894454592
LOC284688	-0.004062389	0.937841244	0.95208282
LOC284749	0.39476357	2.76E-15	4.24E-14
LOC284788	-0.248042353	1.32E-06	5.07E-06
LOC284798	0.134423979	0.009535691	0.017684793
LOC284837	0.200876575	9.78E-05	0.000270836
LOC284900	0.002651474	0.959406208	0.968153707
LOC285033	-0.000533914	0.991822463	0.993568014
LOC285045	0.019770068	0.704279113	0.759398597
LOC285074	-0.083268807	0.109323749	0.155331476
LOC285205	0.131597431	0.01117318	0.020372697
LOC285359	0.340673119	1.56E-11	1.33E-10
LOC285370	0.088936398	0.087146366	0.127319588
LOC285375	0.057502575	0.269265939	0.339149321
LOC285401	0.155475153	0.00267475	0.005605552
LOC285419	0.191238277	0.000211113	0.000548703
LOC285456	-0.312649642	7.43E-10	4.89E-09
LOC285501	0.04440447	0.393756333	0.470119481
LOC285548	0.297489126	5.11E-09	2.95E-08
LOC285593	-0.247410571	1.40E-06	5.37E-06
LOC285627	0.049250619	0.344145044	0.418435005
LOC285629	0.277920235	5.25E-08	2.56E-07
LOC285692	0.083847982	0.106871282	0.152317189
LOC285696	0.007801149	0.88095716	0.906015618
LOC285733	-0.140251636	0.006815913	0.013041358
LOC285735	-0.058226798	0.263271524	0.33271348
LOC285740	0.151980098	0.003340533	0.006837816
LOC285768	0.240945621	2.67E-06	9.78E-06
LOC285780	-0.149137363	0.003989227	0.00803167
LOC285796	-0.229367417	8.10E-06	2.72E-05
LOC285830	0.076567219	0.14102799	0.193736009
LOC285847	-0.135711828	0.00886303	0.016540448
LOC285954	-0.266262973	1.93E-07	8.59E-07
LOC286002	0.063858654	0.219782505	0.284836525
LOC286094	0.095219703	0.066945968	0.100984142
LOC286135	0.031498135	0.545312084	0.616520478
LOC286238	-0.085141492	0.10155082	0.145735157
LOC286359	0.161909998	0.001755506	0.003815951
LOC286367	0.014121573	0.786316926	0.828867475
LOC286467	0.130580635	0.011820405	0.021435109
LOC29034	-0.138753681	0.007439013	0.014111599
LOC338588	0.013682349	0.792809856	0.834267798
LOC338651	0.103756002	0.045810958	0.072197287
LOC338758	-0.028345017	0.586282802	0.65426061
LOC338799	0.153352018	0.003063074	0.00632686
LOC339047	0.202239571	8.75E-05	0.000244246
LOC339240	-0.021879993	0.674436825	0.733693574
LOC339290	-0.26098994	3.42E-07	1.46E-06
LOC339524	-0.045573251	0.381413134	0.457631442

LOC339535	0.133242056	0.010192531	0.018777104
LOC339674	0.39414023	3.08E-15	4.70E-14
LOC339788	0.094664732	0.068559664	0.103190562
LOC340017	0.061917048	0.23415492	0.300763012
LOC340074	0.050955505	0.327680621	0.401775808
LOC340094	0.092620013	0.074781998	0.111192168
LOC340357	0.035948669	0.490001243	0.564168338
LOC340508	0.058371289	0.262086635	0.331678944
LOC341056	0.335968357	3.06E-11	2.49E-10
LOC342346	-0.329587738	7.53E-11	5.81E-10
LOC344595	0.193501187	0.00017682	0.000465953
LOC344967	0.185582096	0.00032593	0.000816341
LOC347376	0.060048943	0.248597472	0.316659772
LOC348021	0.08041106	0.122079353	0.171031286
LOC348840	0.063214506	0.224479088	0.290130556
LOC348926	-0.084677253	0.103435629	0.148066724
LOC349114	0.411591897	1.33E-16	2.53E-15
LOC349196	0.121908284	0.018825773	0.032508195
LOC360030	0.159240325	0.002094542	0.004491171
LOC374443	0.354487548	2.00E-12	1.96E-11
LOC374491	0.216448299	2.61E-05	7.99E-05
LOC375190	0.083335664	0.109038407	0.155028771
LOC387646	0.208244805	5.30E-05	0.000153925
LOC387647	0.164712625	0.001454283	0.003219135
LOC388152	0.397347892	1.75E-15	2.76E-14
LOC388242	0.159328323	0.002082475	0.00446722
LOC388387	-0.218324158	2.21E-05	6.86E-05
LOC388428	0.238676412	3.33E-06	1.20E-05
LOC388588	-0.066250776	0.202956631	0.2658493
LOC388692	0.326504921	1.15E-10	8.61E-10
LOC388789	0.025516706	0.624199199	0.689165221
LOC388796	0.275713778	6.75E-08	3.23E-07
LOC388946	0.083502784	0.10832771	0.154161343
LOC388955	0.183183622	0.000390363	0.000963551
LOC389033	-0.015868116	0.760648507	0.806912841
LOC389332	0.272679973	9.51E-08	4.43E-07
LOC389333	0.202707438	8.42E-05	0.000235737
LOC389458	0.004437308	0.932117813	0.947334511
LOC389493	0.056218803	0.280119514	0.350789585
LOC389634	0.276070894	6.48E-08	3.12E-07
LOC389705	0.052585024	0.312428115	0.385282744
LOC389791	0.172687434	0.000837489	0.001942932
LOC390595	-0.143799994	0.005522334	0.010761609
LOC390858	0.003152146	0.95174954	0.962621431
LOC391322	-0.012461724	0.810927961	0.84898349
LOC392196	-0.01269199	0.807502004	0.846184648
LOC399744	-0.144199355	0.005391521	0.010529348
LOC399815	0.310072871	1.04E-09	6.69E-09
LOC399959	-0.413524959	9.31E-17	1.81E-15
LOC400027	0.023322063	0.654326643	0.716590368
LOC400043	0.321177787	2.39E-10	1.70E-09
LOC400657	0.060179042	0.247571994	0.315555296
LOC400696	0.22192778	1.60E-05	5.09E-05
LOC400752	-0.329907426	7.20E-11	5.57E-10
LOC400759	-0.015872617	0.760582687	0.806912841
LOC400794	-0.106122534	0.041058491	0.065500261
LOC400804	0.042384243	0.415651167	0.491663849
LOC400891	0.108446986	0.036802416	0.059323386

LOC400927	0.093448776	0.072206734	0.107874207
LOC400931	0.189682247	0.000238201	0.000612077
LOC400940	0.213348092	3.42E-05	0.000102818
LOC401010	0.024152663	0.642854862	0.706429488
LOC401052	0.206421746	6.18E-05	0.000177474
LOC401093	-0.249399714	1.15E-06	4.47E-06
LOC401127	-0.115511618	0.026090959	0.043660991
LOC401387	0.028852482	0.579593869	0.648397858
LOC401397	0.061643659	0.236230722	0.303097085
LOC401431	-0.035247348	0.498513044	0.572413652
LOC401463	0.08042039	0.122035898	0.170988463
LOC401588	0.080945972	0.119607487	0.167981697
LOC402377	-0.123708453	0.017129504	0.029849517
LOC402644	0.103838637	0.045637553	0.071958158
LOC407835	0.234663565	4.91E-06	1.72E-05
LOC415056	0.096144345	0.064326625	0.097483267
LOC440040	0.001809011	0.972297867	0.978140591
LOC440173	0.139633464	0.007067158	0.013474202
LOC440354	0.063502946	0.222367267	0.287811896
LOC440356	0.325959445	1.24E-10	9.23E-10
LOC440461	0.015967122	0.759200983	0.80566782
LOC440563	0.123231135	0.017565733	0.030520193
LOC440896	0.222129306	1.57E-05	5.01E-05
LOC440905	0.063614586	0.221553708	0.286870724
LOC440925	-0.354538874	1.98E-12	1.95E-11
LOC440944	0.28511182	2.28E-08	1.19E-07
LOC440957	-0.0426876	0.412318496	0.488530626
LOC441046	-0.105783934	0.041711845	0.066404297
LOC441089	0.249642275	1.12E-06	4.37E-06
LOC441177	0.050736485	0.329766771	0.403811765
LOC441204	0.213892843	3.27E-05	9.83E-05
LOC441208	-0.161397637	0.001816393	0.003940561
LOC441294	0.242621263	2.26E-06	8.37E-06
LOC441454	0.164855102	0.001440318	0.003190706
LOC441455	0.054789665	0.292545874	0.364256182
LOC441601	0.068473203	0.18818028	0.248572022
LOC441666	0.274084456	8.12E-08	3.83E-07
LOC441869	0.12869412	0.013109409	0.023498798
LOC442308	0.06125558	0.239199548	0.306393197
LOC442421	-0.032038671	0.538433283	0.610232556
LOC442454	0.162484118	0.001689504	0.003683334
LOC442459	0.188935509	0.000252323	0.000645204
LOC493754	0.23453659	4.97E-06	1.74E-05
LOC494141	0.096736784	0.062693075	0.095261341
LOC541471	0.283752193	2.67E-08	1.38E-07
LOC541473	-0.042820888	0.410859201	0.487065576
LOC550112	-0.044627924	0.391378057	0.467756534
LOC550643	0.258249504	4.58E-07	1.91E-06
LOC554202	0.221769545	1.63E-05	5.16E-05
LOC55908	-0.011371283	0.827199789	0.8626642
LOC572558	-0.227315274	9.80E-06	3.24E-05
LOC595101	0.112105577	0.030863456	0.050676813
LOC606724	0.286620922	1.90E-08	1.00E-07
LOC613037	0.095252334	0.066852063	0.100850129
LOC619207	-0.050291877	0.334027895	0.40792257
LOC641298	0.016902409	0.745568636	0.794289752
LOC641367	0.143688814	0.005559256	0.010827206
LOC642587	0.04616765	0.375227587	0.451324276

LOC642597	-0.427911453	5.97E-18	1.37E-16
LOC642826	0.05267428	0.311606342	0.384432843
LOC642846	0.283997883	2.60E-08	1.34E-07
LOC642852	0.286776607	1.87E-08	9.87E-08
LOC642929	0.083295216	0.109210966	0.1552298
LOC643008	0.220524949	1.82E-05	5.72E-05
LOC643387	0.284435968	2.47E-08	1.28E-07
LOC643486	0.109389386	0.035186757	0.056981837
LOC643677	-0.115729472	0.025808605	0.043246617
LOC643719	-0.144878612	0.005175414	0.010157087
LOC643763	0.088720597	0.087918872	0.128241306
LOC643837	-0.264661772	2.30E-07	1.01E-06
LOC643923	0.08563034	0.09959565	0.143197499
LOC643955	0.019292109	0.711104745	0.765184657
LOC644145	0.009289602	0.858463497	0.888939643
LOC644165	0.337468227	2.47E-11	2.04E-10
LOC644172	-0.104182829	0.0449212	0.070971573
LOC644538	0.178339262	0.000558159	0.001339555
LOC644669	-0.015044818	0.772717501	0.816965348
LOC644936	0.152953589	0.003141414	0.006476586
LOC645166	0.37299139	1.09E-13	1.30E-12
LOC645323	0.131927411	0.01096998	0.020040711
LOC645332	-0.280544968	3.88E-08	1.94E-07
LOC645431	0.113756837	0.028463827	0.047187847
LOC645676	0.159253992	0.002092664	0.004487824
LOC645752	-0.041425156	0.426291385	0.502668066
LOC646214	0.212242959	3.77E-05	0.000112505
LOC646471	-0.130806853	0.011673613	0.021195927
LOC646498	0.078378085	0.131838062	0.182698794
LOC646627	0.148901709	0.004047809	0.008134817
LOC646762	0.105253733	0.042752503	0.067845064
LOC646813	-0.021772242	0.67594902	0.734937312
LOC646851	0.375630545	7.06E-14	8.70E-13
LOC646982	-0.076096408	0.143495813	0.196604717
LOC646999	-0.113464592	0.028876477	0.047800347
LOC647121	0.169066527	0.001079186	0.002448973
LOC647288	-0.170080168	0.001005745	0.002294338
LOC647309	-0.333346281	4.45E-11	3.54E-10
LOC647859	-0.020808005	0.689538618	0.746567782
LOC647946	0.309541046	1.11E-09	7.14E-09
LOC647979	-0.171726999	0.000896187	0.002064743
LOC648691	0.201656754	9.18E-05	0.000255072
LOC648740	0.0068895	0.894782677	0.91706248
LOC649330	0.274826964	7.46E-08	3.55E-07
LOC650368	0.052965325	0.308936602	0.38168279
LOC650623	-0.028229279	0.58781334	0.655520582
LOC651250	0.0778471	0.134483522	0.185938005
LOC652276	0.075749948	0.145332802	0.198731559
LOC653113	-0.089549338	0.084981942	0.124531831
LOC653501	-0.219666249	1.96E-05	6.14E-05
LOC653544	0.045457332	0.382626658	0.458921631
LOC653566	0.144346589	0.005344001	0.010448835
LOC653653	0.033322012	0.522277182	0.594458437
LOC653786	0.094669784	0.068544832	0.103182268
LOC654342	0.198050157	0.000123062	0.000334146
LOC654433	0.054096722	0.298701769	0.370947304
LOC678655	0.109708824	0.034652972	0.05621338
LOC723809	0.378761575	4.22E-14	5.36E-13

LOC723972	0.228568897	8.72E-06	2.91E-05
LOC727677	-0.066845899	0.198919728	0.261196864
LOC727896	0.280401871	3.95E-08	1.97E-07
LOC727924	0.021047996	0.686146735	0.743832794
LOC728024	-0.10960589	0.034824218	0.056463586
LOC728190	0.083263862	0.109344876	0.155331476
LOC728264	0.195335458	0.000152928	0.000407958
LOC728276	-0.078639656	0.130549724	0.181178098
LOC728323	-0.105650029	0.041972632	0.066734139
LOC728392	0.135359237	0.009042864	0.016853955
LOC728554	0.244153665	1.94E-06	7.27E-06
LOC728606	-0.027724094	0.594515396	0.66202429
LOC728613	0.298984125	4.25E-09	2.49E-08
LOC728640	0.057317114	0.270815886	0.340713236
LOC728643	0.249144419	1.18E-06	4.58E-06
LOC728723	0.049542825	0.341286428	0.415542612
LOC728743	-0.163649322	0.001562487	0.003430825
LOC728758	-0.047981732	0.35673431	0.431870498
LOC728819	0.266860023	1.81E-07	8.10E-07
LOC728855	0.110779576	0.032913695	0.053711225
LOC728875	-0.1339134	0.009814682	0.018159942
LOC728989	-0.176041667	0.00065923	0.001562543
LOC729020	0.088773275	0.087729793	0.128039204
LOC729082	0.048620114	0.350364897	0.425037722
LOC729121	0.056494624	0.277762988	0.348309576
LOC729156	-0.014889331	0.775003094	0.818903768
LOC729176	0.08358082	0.107997106	0.153723803
LOC729234	0.158133407	0.002251888	0.004795009
LOC729375	-0.03445342	0.508242483	0.581206977
LOC729467	0.026052159	0.616940179	0.682171408
LOC729603	-0.011863718	0.819841963	0.856717066
LOC729609	0.07873714	0.130072075	0.180641051
LOC729668	-0.028854022	0.579573616	0.648397858
LOC729678	0.108366761	0.036942816	0.059530436
LOC729799	0.066345959	0.202307017	0.265120405
LOC729991-	0.036561594	0.482626538	0.556803677
LOC729991	0.118588423	0.022340204	0.038000473
LOC730101	-0.010241766	0.844132896	0.876652013
LOC730668	0.165477293	0.001380768	0.003069365
LOC731779	0.04758441	0.360735021	0.436316136
LOC731789	0.095462012	0.066251236	0.100034647
LOC732275	0.008967999	0.863314562	0.892524791
LOC80054	-0.044773783	0.389830338	0.466223344
LOC80154	0.343454546	1.04E-11	9.12E-11
LOC81691	0.157787825	0.002303176	0.00489474
LOC84740	0.288499318	1.52E-08	8.16E-08
LOC84856	0.171822723	0.00089017	0.002052257
LOC84931	0.096831789	0.062434323	0.094914526
LOC84989	0.192958661	0.00018453	0.000484796
LOC90110	-0.094896034	0.067883275	0.102289585
LOC90246	0.038468487	0.460071876	0.534926615
LOC90586	-0.291563454	1.05E-08	5.81E-08
LOC90784	0.132750313	0.010477424	0.019223729
LOC90834	0.129073357	0.012840767	0.023067065
LOC91149	0.114109738	0.027972281	0.046442519
LOC91316	0.319467656	3.01E-10	2.10E-09
LOC91450	0.069985925	0.178584855	0.237628071
LOC91948	-0.017957898	0.730279217	0.781511741

LOC92249	-0.159157662	0.002105936	0.004512203
LOC92659	0.281946273	3.30E-08	1.67E-07
LOC92973	-0.064839144	0.212769095	0.276936228
LOC93432	0.28106949	3.65E-08	1.84E-07
LOC93622	0.094541236	0.068923053	0.103613421
LOC96610	0.238157345	3.51E-06	1.26E-05
LOH12CR1	0.043830979	0.399899916	0.476056536
LOH12CR2	-0.309798236	1.08E-09	6.91E-09
LOH3CR2A	0.194024526	0.000169669	0.000448831
LONP1	0.012502323	0.810323664	0.8485292
LONP2	-0.414578569	7.65E-17	1.50E-15
LONRF1	-0.055627132	0.285220087	0.356381998
LONRF2	0.341458797	1.39E-11	1.20E-10
LONRF3	-0.190379237	0.000225689	0.000582783
LOR	0.113912012	0.028246783	0.046862052
LOXHD1	-0.223777094	1.36E-05	4.37E-05
LOXL1	0.2812086	3.59E-08	1.81E-07
LOXL2	0.285349117	2.21E-08	1.16E-07
LOXL3	0.331043736	6.15E-11	4.80E-10
LOXL4	0.230771382	7.10E-06	2.41E-05
LOX	0.111081652	0.03243669	0.053024021
LPAL2	-0.050932185	0.327902335	0.401998174
LPAR1	0.250789191	9.95E-07	3.92E-06
LPAR2	0.500211821	6.95E-25	4.39E-23
LPAR3	0.239632279	3.04E-06	1.10E-05
LPAR4	0.072292747	0.164657504	0.221552532
LPAR5	0.28776608	1.66E-08	8.85E-08
LPAR6	0.220641696	1.80E-05	5.67E-05
LPA	-0.242380501	2.32E-06	8.56E-06
LPCAT1	0.498980376	9.42E-25	5.87E-23
LPCAT2	-0.04312704	0.407518898	0.483748002
LPCAT3	0.074037998	0.154674069	0.20982002
LPCAT4	0.454121188	2.82E-20	8.93E-19
LPGAT1	0.155755579	0.002626956	0.005518582
LPHN1	0.216180052	2.67E-05	8.16E-05
LPHN2	-0.020504921	0.693831122	0.750529599
LPHN3	0.001304068	0.980028348	0.984525478
LPIN1	-0.164624022	0.001463029	0.003236698
LPIN2	-0.137327104	0.00807922	0.015220136
LPIN3	0.043349707	0.405099621	0.481268689
LPL	-0.019875124	0.70278199	0.758295764
LPO	0.163246603	0.001605362	0.003513349
LPPR1	-0.223355927	1.41E-05	4.52E-05
LPPR2	-0.033982948	0.514054495	0.586775548
LPPR3	0.230143464	7.53E-06	2.55E-05
LPPR4	0.091096348	0.079711288	0.117752356
LPPR5	0.03496959	0.501905658	0.575347519
LPP	0.015149566	0.771178854	0.815726878
LPXN	0.144995408	0.005139051	0.010093677
LQK1	0.08575683	0.099094649	0.142559474
LRAT	-0.218959814	2.09E-05	6.51E-05
LRBA	-0.023074617	0.657760155	0.719757102
LRCH1	-0.149435589	0.00391619	0.00789979
LRCH2	-0.01448465	0.780960779	0.824062534
LRCH3	0.098912439	0.056984578	0.087596202
LRCH4	0.118236045	0.022744641	0.038625756
LRDD	0.263226925	2.69E-07	1.17E-06
LRFN1	0.312877648	7.21E-10	4.75E-09

LRFN2	0.065898553	0.205373687	0.268556258
LRFN3	-0.076584776	0.140936594	0.193623807
LRFN4	0.337024102	2.64E-11	2.16E-10
LRFN5	-0.005422899	0.917089431	0.935161777
LRG1	-0.090195228	0.082748233	0.121652299
LRGUK	0.112559336	0.030187408	0.049734828
LRIG1	-0.165910289	0.001340669	0.002985224
LRIG2	0.019225242	0.712061508	0.765965948
LRIG3	0.151233427	0.003500941	0.007135114
LRIT1	0.133558675	0.010012752	0.018478375
LRIT2	-0.07149654	0.169369446	0.227125612
LRIT3	-0.075266297	0.14792713	0.201904924
LRMP	0.029246577	0.574423989	0.643734866
LRP10	0.27778192	5.33E-08	2.60E-07
LRP11	0.353173582	2.44E-12	2.36E-11
LRP12	0.296612198	5.69E-09	3.27E-08
LRP1B	0.096555555	0.063189117	0.095963835
LRP1	-0.391089514	5.23E-15	7.68E-14
LRP2BP	-0.201769553	9.09E-05	0.00025297
LRP2	0.096734688	0.062698794	0.095262765
LRP3	0.091431661	0.078604515	0.116298489
LRP4	0.010870502	0.834697847	0.869027986
LRP5L	-0.104252886	0.044776556	0.0707574
LRP5	-0.327843113	9.60E-11	7.25E-10
LRP6	-0.255653827	6.01E-07	2.45E-06
LRP8	0.390419139	5.87E-15	8.54E-14
LRPAP1	-0.026017957	0.617402743	0.682569225
LRPPRC	0.147467931	0.004421302	0.008814288
LRRC10B	0.064523312	0.215010437	0.279579526
LRRC10	-0.023325485	0.654279207	0.716577809
LRRC14B	0.075479222	0.146780678	0.200504983
LRRC14	0.215564175	2.82E-05	8.58E-05
LRRC15	0.162325528	0.001707505	0.003720135
LRRC16A	0.423098629	1.52E-17	3.31E-16
LRRC16B	-0.024418567	0.639200035	0.703183813
LRRC17	0.043945423	0.398669364	0.474879597
LRRC18	0.197197836	0.000131791	0.000355572
LRRC19	0.224688151	1.25E-05	4.05E-05
LRRC1	0.542592482	8.69E-30	1.19E-27
LRRC20	-0.134688429	0.009393978	0.017444336
LRRC23	0.079752209	0.125178391	0.174783005
LRRC24	0.029831159	0.566795537	0.636467264
LRRC25	0.184776103	0.000346386	0.000863775
LRRC26	0.294263979	7.60E-09	4.29E-08
LRRC27	-0.039542867	0.447627508	0.523383483
LRRC28	-0.03672819	0.48063249	0.554888762
LRRC29	-0.245947282	1.63E-06	6.15E-06
LRRC2	-0.258010027	4.69E-07	1.95E-06
LRRC30	0.033652496	0.51815724	0.590692126
LRRC31	0.04093998	0.431733702	0.507823039
LRRC32	-0.00398896	0.938962576	0.952978371
LRRC33	0.078631357	0.13059045	0.181221993
LRRC34	0.036659801	0.481450516	0.555736554
LRRC36	0.129660777	0.012434226	0.022431825
LRRC37A2	0.147062326	0.004532462	0.009011548
LRRC37A3	0.02758186	0.596408603	0.663519975
LRRC37A4	0.168648809	0.001110863	0.002515128
LRRC37A	0.152545038	0.003223629	0.006623468

LRRC37B2	0.336084583	3.01E-11	2.46E-10
LRRC37B	0.380115875	3.37E-14	4.34E-13
LRRC39	0.006043006	0.907648746	0.927481449
LRRC3B	-0.096692265	0.062814632	0.09542421
LRRC3	-0.27400758	8.19E-08	3.86E-07
LRRC40	-0.114133228	0.027939824	0.046404099
LRRC41	0.105757703	0.041762822	0.066469517
LRRC42	0.190001608	0.000232389	0.000598689
LRRC43	0.147004429	0.004548534	0.009042599
LRRC45	-0.184810412	0.000345491	0.000861867
LRRC46	0.103280338	0.046819894	0.073659158
LRRC47	-0.192098958	0.000197396	0.000515381
LRRC48	0.011706035	0.822196326	0.858741486
LRRC49	0.19059064	0.000222017	0.000574493
LRRC4B	0.063442082	0.222811703	0.288274759
LRRC4C	0.173806997	0.000773559	0.001806875
LRRC4	0.011332858	0.827774566	0.86312807
LRRC50	-0.097773074	0.059917889	0.091534646
LRRC52	-0.172976156	0.000820551	0.001906136
LRRC55	0.053179966	0.306977377	0.379615351
LRRC56	0.183568665	0.000379278	0.000938047
LRRC57	0.171926396	0.000883697	0.002039505
LRRC58	0.033439515	0.52081043	0.59309394
LRRC59	0.354146201	2.10E-12	2.06E-11
LRRC61	0.017733127	0.733526469	0.784018151
LRRC66	0.169820193	0.001024128	0.002333602
LRRC67	0.096497689	0.06334818	0.096190735
LRRC69	0.168654425	0.001110432	0.002514437
LRRC6	0.015670934	0.763533899	0.809489774
LRRC70	-0.09495804	0.067702886	0.102040921
LRRC7	0.090502244	0.081703212	0.120275725
LRRC8A	-0.01110127	0.831240719	0.866017028
LRRC8B	0.373100899	1.07E-13	1.28E-12
LRRC8C	0.131079107	0.011499085	0.02089236
LRRC8D	-0.208415781	5.22E-05	0.000151889
LRRC8E	0.283287033	2.82E-08	1.45E-07
LRRCC1	0.08030184	0.12258892	0.171648614
LRRFIP1	-0.22518632	1.19E-05	3.88E-05
LRRFIP2	-0.006771153	0.896579882	0.918573639
LRRIQ1	0.225806271	1.13E-05	3.68E-05
LRRIQ3	-0.181948182	0.000428002	0.00104762
LRRIQ4	0.146446298	0.004706119	0.009332599
LRRK1	0.286200394	2.00E-08	1.05E-07
LRRK2	-0.173352386	0.000798954	0.001860739
LRRN1	0.281113131	3.63E-08	1.83E-07
LRRN2	0.30168839	3.03E-09	1.82E-08
LRRN3	0.178901439	0.000535721	0.001290368
LRRN4CL	0.140653395	0.006656941	0.012766613
LRRN4	0.075538084	0.146464949	0.200114856
LRRTM1	-0.108754224	0.036268906	0.05852022
LRRTM2	-0.243531143	2.07E-06	7.70E-06
LRRTM3	0.007868969	0.879930029	0.905192917
LRRTM4	-0.13212079	0.010852425	0.019844726
LRSAM1	-0.261087009	3.38E-07	1.44E-06
LRTM1	0.054662488	0.29366927	0.365495045
LRTM2	0.201428484	9.35E-05	0.000259709
LRTOMT	-0.319190904	3.12E-10	2.18E-09
LRWD1	-0.085291668	0.100946972	0.144973009

LSAMP	0.195374131	0.000152458	0.00040676
LSG1	0.128993889	0.012896654	0.023161196
LSM10	0.083620475	0.107829408	0.153522065
LSM11	0.28837437	1.55E-08	8.27E-08
LSM12	0.32146368	2.30E-10	1.64E-09
LSM14A	0.258067488	4.67E-07	1.94E-06
LSM14B	0.167646674	0.001190382	0.002678427
LSM1	0.201208274	9.52E-05	0.000264037
LSM2	0.298329745	4.61E-09	2.68E-08
LSM3	0.118301975	0.022668492	0.038512852
LSM4	0.274895902	7.41E-08	3.52E-07
LSM5	-0.064269397	0.216824621	0.281523632
LSM6	0.019705809	0.705195414	0.760200762
LSM7	0.228846741	8.50E-06	2.85E-05
LSMD1	-0.005231391	0.920007432	0.937487623
LSP1	0.278033905	5.18E-08	2.53E-07
LSR	0.172983931	0.000820099	0.001905309
LSS	-0.090380283	0.082117058	0.12080461
LST-3TM12	-0.262201488	3.00E-07	1.29E-06
LST1	0.241557181	2.52E-06	9.24E-06
LTA4H	0.204230479	7.42E-05	0.000210011
LTA	0.287816732	1.65E-08	8.80E-08
LTB4R2	-0.131997813	0.010927053	0.019967782
LTB4R	-0.013724543	0.792185486	0.833875053
LTBP1	0.127464614	0.014014702	0.024926426
LTBP2	0.155995384	0.002586704	0.005445088
LTBP3	0.05208385	0.317068751	0.390301759
LTBP4	0.113272766	0.029150117	0.048189248
LTBR	0.037025222	0.47708832	0.551596165
LTB	0.389642731	6.72E-15	9.71E-14
LTC4S	0.087574294	0.092114955	0.133696206
LTF	0.088102972	0.090160221	0.131126582
LTK	0.227256293	9.85E-06	3.25E-05
LTV1	0.181573678	0.000440061	0.001075562
LUC7L2	0.057352488	0.270519788	0.340491232
LUC7L3	0.320594383	2.58E-10	1.83E-09
LUC7L	0.303486237	2.41E-09	1.47E-08
LUM	0.195486241	0.000151105	0.000403473
LUZP1	0.15589683	0.002603178	0.005474561
LUZP2	-0.149427538	0.003918145	0.007902936
LUZP4	0.050159029	0.335307953	0.40926388
LUZP6	0.019792877	0.703953972	0.759130185
LXN	0.232752671	5.89E-06	2.03E-05
LY6D	0.096572794	0.063141795	0.09590659
LY6E	0.267277479	1.73E-07	7.75E-07
LY6G5B	0.288567672	1.51E-08	8.10E-08
LY6G5C	0.017440555	0.737760359	0.787402865
LY6G6C	0.129853588	0.012303283	0.022213704
LY6G6D	-0.01256831	0.809341707	0.847812887
LY6G6E	0.066419143	0.201808569	0.264536802
LY6G6F	-0.086994786	0.094296424	0.13645481
LY6H	0.215179075	2.92E-05	8.86E-05
LY6K	-0.022576789	0.664689711	0.725625975
LY75	0.102498044	0.048519695	0.07606218
LY86	0.212259606	3.76E-05	0.000112377
LY96	0.250472741	1.03E-06	4.04E-06
LY9	0.192762227	0.000187399	0.000491489
LYAR	0.228695064	8.62E-06	2.88E-05

LYG1	0.098646551	0.057658244	0.088495187
LYG2	0.154397692	0.002865838	0.005957761
LYL1	0.244120691	1.95E-06	7.29E-06
LYNX1	-0.110719873	0.033008675	0.053852986
LYN	0.194517785	0.000163178	0.00043304
LYPD1	0.343117641	1.09E-11	9.56E-11
LYPD2	-0.107227581	0.038985993	0.06244906
LYPD3	0.135071279	0.009192131	0.017104954
LYPD4	0.104651813	0.043960343	0.069561394
LYPD5	0.262933531	2.78E-07	1.20E-06
LYPD6B	0.357696902	1.22E-12	1.24E-11
LYPD6	0.267503921	1.69E-07	7.57E-07
LYPLA1	-0.007493591	0.885617563	0.909750535
LYPLA2P1	0.030763837	0.55472574	0.625601256
LYPLA2	0.084415486	0.104510549	0.149412211
LYPLAL1	-0.111509236	0.031771588	0.052040666
LYRM1	-0.251555241	9.20E-07	3.64E-06
LYRM2	0.17400622	0.000762667	0.001783944
LYRM4	0.053978163	0.299763579	0.372057194
LYRM5	-0.396680476	1.97E-15	3.09E-14
LYRM7	-0.145002475	0.005136858	0.010090365
LYSMD1	0.231549132	6.60E-06	2.26E-05
LYSMD2	-0.007032997	0.892604257	0.915222929
LYSMD3	-0.135170401	0.009140505	0.017024788
LYSMD4	0.155768757	0.00262473	0.005515227
LYST	-0.138044895	0.007751201	0.014660536
LYVE1	-0.179741287	0.000503755	0.001218825
LYZL1	-0.003022025	0.953739052	0.964046344
LYZL2	-0.099334322	0.055929067	0.086193152
LYZL4	0.038519669	0.459474695	0.534457575
LYZL6	-0.067926551	0.191739346	0.252734634
LYZ	0.115522511	0.026076778	0.043646798
LZIC	-0.040501303	0.436688759	0.512803623
LZTFL1	-0.253291753	7.69E-07	3.08E-06
LZTR1	-0.042113453	0.418639411	0.494640552
LZTS1	0.261253913	3.32E-07	1.42E-06
LZTS2	0.375270842	7.49E-14	9.19E-13
M6PR	0.220935221	1.75E-05	5.53E-05
MAB21L1	0.127154873	0.014251257	0.025304238
MAB21L2	0.122973568	0.017805133	0.030903803
MACC1	0.334991202	3.52E-11	2.84E-10
MACF1	-0.124512961	0.016415651	0.028727038
MACROD1	-0.409765146	1.87E-16	3.45E-15
MACROD2	0.092619107	0.074784854	0.111192168
MAD1L1	0.120252923	0.020512884	0.035147302
MAD2L1BP	0.045979919	0.377174487	0.453392273
MAD2L1	0.508919526	7.76E-26	5.77E-24
MAD2L2	0.042815448	0.410918692	0.487107115
MADCAM1	-0.028639217	0.582400589	0.650952902
MADD	0.120647415	0.020099335	0.034486173
MAEA	0.316456631	4.49E-10	3.06E-09
MAEL	0.227431028	9.69E-06	3.21E-05
MAF1	0.271604429	1.07E-07	4.95E-07
MAFA	0.138488196	0.007554606	0.014314521
MAFB	-0.079939704	0.124290317	0.173750031
MAFF	0.0404761	0.436974432	0.513048363
MAFG	0.189950048	0.000233319	0.000600539
MAFK	-0.044659819	0.391039293	0.467463834

MAF	-0.160261073	0.001958414	0.00422109
MAGEA10	0.162957363	0.001636817	0.003576903
MAGEA11	0.079707554	0.125390626	0.175030273
MAGEA12	0.004063727	0.937820822	0.95208282
MAGEA1	0.023341845	0.654052462	0.716408244
MAGEA2	0.082005512	0.114826723	0.162188474
MAGEA3	0.046482486	0.371976442	0.447792294
MAGEA4	0.187415208	0.000283526	0.000718991
MAGEA5	0.142323224	0.006031196	0.011663122
MAGEA6	0.06379247	0.220261796	0.285401984
MAGEA8	0.043761492	0.40064818	0.476833326
MAGEA9B	0.072557427	0.163113107	0.219727008
MAGEB10	-0.041534192	0.425073824	0.501469813
MAGEB16	-0.023076202	0.657738138	0.719757102
MAGEB18	0.076918191	0.139209555	0.191594664
MAGEB1	-0.033926876	0.514749472	0.587434209
MAGEB2	0.027266466	0.600616396	0.667491074
MAGEB3	0.023500035	0.65186162	0.714597876
MAGEB4	-0.044177728	0.396178497	0.472416833
MAGEB6	-0.042542383	0.413911873	0.490013806
MAGEC1	-0.117623426	0.023462859	0.039723556
MAGEC2	-0.064505739	0.215135639	0.279705814
MAGEC3	0.037497997	0.471476701	0.546249424
MAGED1	0.308457838	1.28E-09	8.12E-09
MAGED2	0.217740094	2.33E-05	7.19E-05
MAGED4B	0.453005578	3.58E-20	1.10E-18
MAGED4	0.435692897	1.28E-18	3.27E-17
MAGEE1	0.264589158	2.32E-07	1.02E-06
MAGEE2	0.109155501	0.035582	0.057556398
MAGEF1	0.160216639	0.001964167	0.004232116
MAGEH1	0.111512291	0.031766878	0.052040108
MAGEL2	0.110335827	0.033625252	0.054728944
MAGI1	-0.309604173	1.10E-09	7.08E-09
MAGI2	-0.118943027	0.021939514	0.037360372
MAGI3	-0.124005455	0.016862871	0.029435041
MAGIX	-0.07989412	0.124505779	0.173977984
MAGOHB	0.239272331	3.15E-06	1.14E-05
MAGOH	0.220374478	1.84E-05	5.79E-05
MAGT1	-0.129867719	0.012293734	0.022200488
MAG	0.079332187	0.12718571	0.177138822
MAK16	0.15014168	0.003748063	0.007592934
MAK	0.133566069	0.010008588	0.0184724
MAL2	0.517360948	8.72E-27	7.45E-25
MALAT1	0.270591324	1.20E-07	5.51E-07
MALL	-0.032333972	0.534693702	0.606511867
MALT1	0.040212964	0.439963379	0.515585319
MAL	0.18520051	0.00033547	0.000838662
MAMDC2	-0.081618097	0.116557116	0.164265058
MAMDC4	-0.117957207	0.02306915	0.039133481
MAML1	0.309157073	1.17E-09	7.47E-09
MAML2	0.052961908	0.308967857	0.381697733
MAML3	-0.210309352	4.45E-05	0.000131235
MAMLD1	0.253164135	7.79E-07	3.12E-06
MAMSTR	0.335880586	3.10E-11	2.52E-10
MAN1A1	-0.091368916	0.078810664	0.116525608
MAN1A2	-0.134178125	0.00966914	0.01791394
MAN1B1	0.067762153	0.192819271	0.25399005
MAN1C1	-0.206667142	6.06E-05	0.000174155

MAN2A1	-0.119636614	0.021173756	0.036176979
MAN2A2	-0.060023467	0.248798626	0.316814716
MAN2B1	0.179251062	0.000522192	0.001260066
MAN2B2	-0.154393562	0.002866594	0.00595871
MAN2C1	-0.277753668	5.35E-08	2.61E-07
MANBAL	0.206891742	5.94E-05	0.000171095
MANBA	0.097924052	0.059522167	0.090970511
MANEAL	-0.087710367	0.091608624	0.133048554
MANEA	-0.160723528	0.001899445	0.004105983
MANF	0.195136051	0.00015537	0.00041403
MANSC1	0.19635195	0.000141027	0.000378643
MAOA	-0.309979563	1.05E-09	6.77E-09
MAOB	-0.411294743	1.41E-16	2.66E-15
MAP1A	0.304342413	2.17E-09	1.33E-08
MAP1B	0.051320573	0.32422239	0.398221852
MAP1D	-0.341543119	1.37E-11	1.18E-10
MAP1LC3A	-0.22244026	1.53E-05	4.88E-05
MAP1LC3B2	0.015318061	0.7687057	0.813673819
MAP1LC3B	0.008322193	0.873071236	0.900182443
MAP1LC3C	0.112900026	0.029688186	0.048977148
MAP1S	0.358145771	1.14E-12	1.16E-11
MAP2K1	0.028603865	0.582866453	0.651363969
MAP2K2	0.26573737	2.05E-07	9.06E-07
MAP2K3	-0.133076709	0.01028755	0.018929396
MAP2K4	-0.048488048	0.351676646	0.426349941
MAP2K5	-0.238067528	3.54E-06	1.27E-05
MAP2K6	0.089109295	0.08653139	0.126504612
MAP2K7	-0.098310757	0.058518434	0.089641933
MAP2	0.059106397	0.256115253	0.325030455
MAP3K10	0.173389771	0.000796837	0.001856459
MAP3K11	-0.080047695	0.123781043	0.173123136
MAP3K12	0.274049325	8.15E-08	3.84E-07
MAP3K13	-0.265110954	2.19E-07	9.66E-07
MAP3K14	0.088376462	0.089162145	0.129807676
MAP3K15	0.285790758	2.10E-08	1.10E-07
MAP3K1	0.247284989	1.42E-06	5.43E-06
MAP3K2	-0.112928743	0.029646431	0.048928523
MAP3K3	0.078753344	0.12999281	0.180556143
MAP3K4	0.190993459	0.000215174	0.000558092
MAP3K5	-0.002881066	0.955894599	0.965490378
MAP3K6	0.236799779	4.00E-06	1.42E-05
MAP3K7	0.140752432	0.006618267	0.012703556
MAP3K8	-0.03170876	0.54262653	0.614111557
MAP3K9	0.032603632	0.531290282	0.60323521
MAP4K1	0.253287773	7.69E-07	3.08E-06
MAP4K2	0.172374593	0.000856207	0.00198203
MAP4K3	0.117750879	0.023311842	0.039504807
MAP4K4	0.170228491	0.000995394	0.002273067
MAP4K5	0.106031587	0.041233127	0.065757794
MAP4	0.139711872	0.007034839	0.013419002
MAP6D1	-0.076794515	0.139848273	0.192340855
MAP6	0.138670089	0.007475238	0.014174918
MAP7D1	0.393901116	3.21E-15	4.88E-14
MAP7D2	0.260614318	3.56E-07	1.51E-06
MAP7D3	0.029532101	0.570692029	0.640164787
MAP7	-0.11247497	0.030312136	0.04991968
MAP9	0.22936986	8.09E-06	2.72E-05
MAPK10	0.156690711	0.002473144	0.005225366

MAPK11	0.130352245	0.011970258	0.021673314
MAPK12	0.261091765	3.38E-07	1.44E-06
MAPK13	0.512969703	2.74E-26	2.25E-24
MAPK14	-0.081738205	0.116018482	0.163575385
MAPK15	0.305079245	1.97E-09	1.22E-08
MAPK1IP1L	0.195671538	0.000148893	0.000398099
MAPK1	0.004067098	0.937769342	0.95208282
MAPK3	0.334969286	3.53E-11	2.85E-10
MAPK4	-0.042224442	0.417413096	0.493396445
MAPK6	0.010190892	0.844897314	0.877354406
MAPK7	0.357595428	1.24E-12	1.25E-11
MAPK8IP1	-0.162036801	0.001740729	0.003786308
MAPK8IP2	0.224635722	1.25E-05	4.06E-05
MAPK8IP3	0.197682269	0.000126761	0.000343208
MAPK8	-0.115170513	0.026538399	0.044331543
MAPK9	0.399187427	1.27E-15	2.04E-14
MAPKAP1	-0.022009377	0.672622752	0.732279931
MAPKAPK2	0.205975486	6.42E-05	0.000183736
MAPKAPK3	0.308559221	1.26E-09	8.02E-09
MAPKAPK5	0.354453729	2.01E-12	1.97E-11
MAPKBP1	0.226466011	1.06E-05	3.49E-05
MAPKSP1	-0.037674176	0.469394856	0.544248884
MAPRE1	0.52071445	3.60E-27	3.30E-25
MAPRE2	-0.086067737	0.097871723	0.140993597
MAPRE3	-0.189619301	0.000239362	0.000614744
MAPT	0.084753627	0.103123661	0.147683817
10-Mar	0.241708397	2.48E-06	9.12E-06
11-Mar	-0.045297192	0.384306985	0.460521128
1-Mar	0.208527243	5.18E-05	0.000150549
2-Mar	-0.294675586	7.22E-09	4.09E-08
3-Mar	0.55036745	9.17E-31	1.46E-28
4-Mar	0.125007107	0.01599021	0.02806048
5-Mar	-0.071740862	0.167912964	0.225384858
6-Mar	0.011274464	0.828648241	0.863948622
7-Mar	0.089707961	0.084428919	0.123785173
8-Mar	-0.286502617	1.93E-08	1.02E-07
9-Mar	0.077743073	0.13500656	0.186505838
MARCKSL1	0.402050045	7.59E-16	1.27E-14
MARCKS	0.438434129	7.35E-19	1.94E-17
MARCO	0.037322307	0.473557821	0.54837357
MARK1	0.243789901	2.02E-06	7.52E-06
MARK2	0.384117123	1.72E-14	2.32E-13
MARK3	0.18935917	0.000244218	0.000626246
MARK4	0.160581609	0.001917367	0.004139334
MARS2	0.154180609	0.002905808	0.006029529
MARS	0.398825672	1.35E-15	2.16E-14
MARVELD1	0.294073557	7.77E-09	4.38E-08
MARVELD2	0.012000252	0.817804672	0.855036645
MARVELD3	0.147451951	0.004425634	0.008822042
MAS1L	0.100361633	0.053426513	0.082752527
MAS1	0.080552628	0.121421317	0.170235483
MASP1	-0.450065826	6.66E-20	2.00E-18
MASP2	-0.426185225	8.36E-18	1.88E-16
MAST1	-0.01147081	0.825711447	0.861517935
MAST2	0.288747076	1.48E-08	7.95E-08
MAST3	-0.199836583	0.000106496	0.000292515
MAST4	-0.049037105	0.346243428	0.420660186
MASTL	0.300689453	3.43E-09	2.04E-08

MAT1A	-0.53191547	1.74E-28	1.92E-26
MAT2A	0.228192992	9.03E-06	3.01E-05
MAT2B	-0.132542568	0.010599887	0.0194299
MATK	0.250453339	1.03E-06	4.05E-06
MATN1	0.225043664	1.21E-05	3.93E-05
MATN2	-0.045993888	0.377029408	0.453245224
MATN3	0.139795025	0.007000708	0.013362854
MATN4	0.160337725	0.001948526	0.004202049
MATR3	0.161273352	0.001831451	0.00396977
MAVS	0.157047123	0.002416714	0.005117535
MAX	0.216509061	2.60E-05	7.95E-05
MAZ	0.267005329	1.78E-07	7.98E-07
MBD1	0.065305974	0.209487035	0.273181954
MBD2	0.154012261	0.002937154	0.00609013
MBD3L1	-0.029308125	0.573618554	0.642940747
MBD3L2	0.032770133	0.529194317	0.601163778
MBD3L5	-0.002822663	0.956787801	0.966049697
MBD3	0.115184951	0.026519326	0.044307113
MBD4	-0.069812289	0.179667468	0.238845275
MBD5	-0.045495005	0.38223201	0.458475893
MBD6	0.183647187	0.000377053	0.000932777
MBIP	-0.219256884	2.04E-05	6.35E-05
MBL1P	-0.121321148	0.019409801	0.033423983
MBL2	-0.305046601	1.98E-09	1.22E-08
MBLAC1	0.012405388	0.811766703	0.84972763
MBLAC2	-0.273086909	9.08E-08	4.25E-07
MBNL1	0.069587271	0.181077664	0.240383103
MBNL2	-0.2157084	2.79E-05	8.48E-05
MBNL3	-0.183914806	0.000369563	0.000915959
MBOAT1	0.203297375	8.02E-05	0.000225229
MBOAT2	0.357783829	1.21E-12	1.22E-11
MBOAT4	0.380173925	3.33E-14	4.30E-13
MBOAT7	0.36454481	4.20E-13	4.58E-12
MBP	0.01933205	0.710533465	0.764735153
MBTD1	-0.024496348	0.63813258	0.702213724
MBTPS1	-0.321582513	2.26E-10	1.61E-09
MBTPS2	-0.14579335	0.00489671	0.00967013
MB	0.129215585	0.012741273	0.022902793
MC1R	0.167906933	0.001169242	0.002636518
MC2R	0.123675326	0.01715947	0.029892529
MC3R	0.026363338	0.612738589	0.678466994
MC4R	0.153892858	0.002959571	0.006132783
MC5R	0.091392434	0.078733344	0.116443602
MCAM	0.191577852	0.000205598	0.000535344
MCART1	0.369914909	1.79E-13	2.07E-12
MCART2	0.065649169	0.207097592	0.27046704
MCART3P	0.082631131	0.112074964	0.158756928
MCART6	0.194197058	0.000167372	0.000443106
MCAT	-0.114119598	0.027958653	0.046431501
MCCC1	-0.177567445	0.000590386	0.001411071
MCCC2	-0.32810484	9.26E-11	7.01E-10
MCCD1	0.24590612	1.63E-06	6.17E-06
MCC	0.060164579	0.247685849	0.315660024
MCEE	-0.520760275	3.55E-27	3.29E-25
MCF2L2	0.335564457	3.25E-11	2.63E-10
MCF2L	0.137490066	0.008003682	0.01508794
MCF2	0.059194802	0.25540352	0.324251142
MCFD2	-0.197800696	0.000125559	0.000340509

MCHR1	0.053603577	0.303134802	0.375717983
MCHR2	0.080831125	0.120134883	0.168650392
MCL1	0.104858944	0.043541506	0.06893697
MCM10	0.557170385	1.22E-31	2.45E-29
MCM2	0.491304368	6.13E-24	3.33E-22
MCM3APAS	0.225820982	1.12E-05	3.68E-05
MCM3AP	0.149049741	0.004010919	0.00807045
MCM3	0.512723516	2.92E-26	2.37E-24
MCM4	0.509559513	6.59E-26	5.01E-24
MCM5	0.376462021	6.16E-14	7.68E-13
MCM6	0.510548349	5.11E-26	4.01E-24
MCM7	0.409266457	2.05E-16	3.74E-15
MCM8	0.349105621	4.50E-12	4.19E-11
MCM9	0.149403838	0.003923908	0.007912155
MCOLN1	-0.113147656	0.029329779	0.048458108
MCOLN2	0.322178877	2.09E-10	1.50E-09
MCOLN3	0.521151588	3.20E-27	3.00E-25
MCPH1	0.121213937	0.019518129	0.033584399
MCRS1	0.498227475	1.13E-24	6.98E-23
MCTP1	0.138251684	0.007658933	0.014501166
MCTP2	0.240291129	2.85E-06	1.04E-05
MCTS1	0.057907793	0.265900536	0.335525113
MDC1	0.222444177	1.53E-05	4.88E-05
MDFIC	-0.136439621	0.008501861	0.01592427
MDFI	0.375017015	7.81E-14	9.56E-13
MDGA1	0.065723204	0.206584726	0.269891856
MDGA2	0.028225055	0.587869226	0.655520582
MDH1B	0.075134206	0.148641763	0.202769187
MDH1	0.060988825	0.241255378	0.308649476
MDH2	-0.188697395	0.000256989	0.000656123
MDK	0.288239717	1.57E-08	8.40E-08
MDM1	0.226896996	1.02E-05	3.36E-05
MDM2	-0.011589241	0.823941217	0.860166479
MDM4	0.210328844	4.44E-05	0.000131055
MDN1	-0.059795395	0.250604498	0.318788252
MDP1	-0.042466825	0.414742361	0.490723799
MDS2	0.147689215	0.0043617	0.008706792
ME1	0.126451299	0.014801667	0.026164935
ME2	0.330206793	6.91E-11	5.36E-10
ME3	0.086278994	0.097047632	0.139988627
MEA1	0.139305873	0.007203632	0.013712092
MEAF6	-0.149536016	0.003891868	0.00785789
MECOM	0.182626081	0.000406951	0.001000353
MECP2	0.099261069	0.056111168	0.086430571
MECR	-0.223092991	1.44E-05	4.63E-05
MED10	0.485958438	2.20E-23	1.10E-21
MED11	-0.076208789	0.142903773	0.195961521
MED12L	0.146420909	0.004713403	0.009342395
MED12	0.177566259	0.000590437	0.001411071
MED13L	0.054955275	0.291087304	0.362802882
MED13	-0.041821633	0.421873776	0.498048555
MED14	0.168049949	0.001157773	0.002612135
MED15	0.40084552	9.42E-16	1.55E-14
MED16	-0.049292673	0.3437327	0.418035703
MED17	0.147160303	0.004505382	0.00896576
MED18	-0.262266578	2.98E-07	1.28E-06
MED19	0.182497837	0.000410858	0.001009261
MED1	0.083961221	0.10639689	0.15178223

MED20	0.176965949	0.000616689	0.001468348
MED21	0.002695301	0.958735808	0.967526203
MED22	0.340161766	1.68E-11	1.42E-10
MED23	0.074832876	0.150281828	0.204782119
MED24	0.189944269	0.000233423	0.00060073
MED25	0.171640472	0.000901657	0.002075662
MED26	0.104015551	0.04526816	0.071443578
MED27	0.269706004	1.32E-07	6.04E-07
MED28	0.239753737	3.00E-06	1.09E-05
MED29	-0.035054776	0.500863881	0.574417722
MED30	0.222678587	1.50E-05	4.79E-05
MED31	-0.066517988	0.201136762	0.263760307
MED4	0.003898604	0.940342571	0.954087328
MED6	0.134603979	0.009439028	0.017520201
MED7	0.388357015	8.37E-15	1.19E-13
MED8	0.185749164	0.000321833	0.0008074
MED9	-0.019926238	0.702053987	0.757737541
MEF2A	-0.043973392	0.398368965	0.47460271
MEF2B	0.175878274	0.000667027	0.001578395
MEF2C	-0.001939785	0.970296163	0.976669369
MEF2D	0.035132088	0.499919386	0.573598687
MEFV	0.079269053	0.127489578	0.177462785
MEG3	0.235609857	4.48E-06	1.58E-05
MEG8	0.13428956	0.009608449	0.017807433
MEGF10	-0.008311176	0.873237848	0.900261043
MEGF11	0.081306483	0.117963692	0.166000753
MEGF6	-0.00488994	0.92521273	0.941487633
MEGF8	0.048854106	0.348048351	0.422561963
MEGF9	-0.141304197	0.006406461	0.012332545
MEI1	0.305660766	1.83E-09	1.14E-08
MEIG1	-0.143869584	0.005499336	0.010719938
MEIS1	0.08321418	0.109557331	0.155599997
MEIS2	0.147546059	0.004400175	0.008776561
MEIS3P1	-0.006122735	0.906435832	0.926479639
MEIS3	0.284666047	2.40E-08	1.25E-07
MELK	0.542618418	8.63E-30	1.19E-27
MEMO1	0.340528028	1.59E-11	1.35E-10
MEN1	-0.000253445	0.996118137	0.996918794
MEOX1	0.198189034	0.000121692	0.000330742
MEOX2	-0.248209943	1.29E-06	4.99E-06
MEP1A	0.354493316	2.00E-12	1.96E-11
MEP1B	-0.171549838	0.00090742	0.002087003
MEPCE	-0.187336963	0.000285225	0.000722841
MEPE	-0.010405919	0.841667345	0.874638613
MERTK	-0.349957721	3.96E-12	3.72E-11
MESDC1	0.344477157	8.94E-12	7.92E-11
MESDC2	0.182030507	0.000425392	0.001041626
MESP1	0.16293826	0.001638915	0.003580879
MESP2	0.23819291	3.49E-06	1.25E-05
MESTIT1	0.08127943	0.118086425	0.16613826
MEST	0.149438835	0.003915401	0.007899
METAP1	-0.018356106	0.72453818	0.776535917
METAP2	0.26183076	3.13E-07	1.34E-06
METRNL	0.351991034	2.92E-12	2.78E-11
METRN	-0.196043481	0.000144543	0.000387198
METT10D	0.066386146	0.202033193	0.264778979
METT11D1	0.214448513	3.11E-05	9.41E-05
METT5D1	-0.145764319	0.004905344	0.009684298

METTL10	-0.008286356	0.873613224	0.900454941
METTL11A	0.017635491	0.734938496	0.785024083
METTL11B	0.262753692	2.83E-07	1.22E-06
METTL12	0.073366846	0.158457682	0.214354175
METTL13	0.0661153	0.203883852	0.266905908
METTL14	-0.308746524	1.23E-09	7.85E-09
METTL1	0.250018389	1.08E-06	4.22E-06
METTL2A	0.147653118	0.004371373	0.008724353
METTL2B	-0.055943673	0.282483577	0.353317291
METTL3	0.141380571	0.006377626	0.012284161
METTL4	0.2423311	2.33E-06	8.60E-06
METTL5	0.209673709	4.70E-05	0.000137675
METTL6	0.281625636	3.42E-08	1.73E-07
METTL7A	-0.543987693	5.83E-30	8.36E-28
METTL7B	-0.195085303	0.000155998	0.000415535
METTL8	0.132548192	0.010596555	0.019426205
METTL9	0.455942545	1.91E-20	6.17E-19
MET	-0.376931751	5.70E-14	7.14E-13
MEX3A	0.329083154	8.08E-11	6.19E-10
MEX3B	0.235892206	4.36E-06	1.54E-05
MEX3C	0.28928062	1.39E-08	7.52E-08
MEX3D	0.089446772	0.085341077	0.124975371
MFAP1	0.110002015	0.034169124	0.055518905
MFAP2	0.409925795	1.81E-16	3.35E-15
MFAP3L	-0.401354947	8.60E-16	1.43E-14
MFAP3	0.110480894	0.033391207	0.054414694
MFAP4	0.11664122	0.024655284	0.041509555
MFAP5	0.136232789	0.008603145	0.016097666
MFF	0.325586415	1.31E-10	9.68E-10
MFGE8	0.227491304	9.64E-06	3.19E-05
MFHAS1	0.012080971	0.816600794	0.853980106
MFI2	0.505121976	2.03E-25	1.43E-23
MFN1	0.049858317	0.338217045	0.412284156
MFN2	-0.244674786	1.85E-06	6.93E-06
MFNG	0.19448075	0.000163657	0.000434254
MFRP	0.115092819	0.02664123	0.044488398
MFSD10	0.554296284	2.88E-31	5.05E-29
MFSD11	-0.124942411	0.016045356	0.028138695
MFSD1	-0.195636653	0.000149307	0.000399046
MFSD2A	-0.24598632	1.62E-06	6.13E-06
MFSD2B	0.401334455	8.63E-16	1.43E-14
MFSD3	0.004929302	0.924612503	0.941098978
MFSD4	0.135112348	0.009170709	0.017073065
MFSD5	0.328379669	8.91E-11	6.78E-10
MFSD6L	0.213361227	3.42E-05	0.000102717
MFSD6	0.382777783	2.16E-14	2.86E-13
MFSD7	0.218964769	2.09E-05	6.51E-05
MFSD8	-0.185511622	0.000327673	0.0008205
MFSD9	-0.203550596	7.85E-05	0.00022109
MGAM	0.236546056	4.10E-06	1.45E-05
MGAT1	-0.076428507	0.141751666	0.194569154
MGAT2	-0.166593348	0.001279582	0.002860716
MGAT3	0.204114626	7.49E-05	0.00021186
MGAT4A	0.367258119	2.73E-13	3.07E-12
MGAT4B	-0.03664935	0.481575591	0.555848721
MGAT4C	0.012185947	0.815035795	0.852477719
MGAT5B	0.12084859	0.019891246	0.034150907
MGAT5	0.071401181	0.169940463	0.22778847

MGA	0.173229416	0.000805955	0.001876166
MGC12916	0.118159657	0.022833145	0.038766143
MGC12982	0.372054221	1.27E-13	1.50E-12
MGC14436	-0.048246465	0.354084185	0.428922847
MGC15885	0.030715597	0.55534693	0.626099264
MGC16025	0.16856545	0.001117286	0.002528521
MGC16121	0.123980601	0.016885044	0.029466
MGC16142	-0.072062728	0.166008538	0.223084121
MGC16275	0.068743036	0.186441488	0.246567135
MGC16384	-0.008405745	0.871807834	0.899065931
MGC16703	0.018278797	0.725651566	0.777352826
MGC21881	-0.091349406	0.078874854	0.116611862
MGC23270	-0.093927919	0.070751163	0.105963403
MGC23284	-0.132985067	0.01034055	0.019012869
MGC26647	0.007273995	0.888947515	0.912396311
MGC27382	-0.106378487	0.040570362	0.064788997
MGC2752	0.205548486	6.65E-05	0.000189818
MGC2889	0.099203454	0.056254737	0.086614643
MGC29506	0.222453281	1.53E-05	4.88E-05
MGC34034	0.078396165	0.131748701	0.182610246
MGC3771	-0.293354701	8.49E-09	4.76E-08
MGC42105	-0.10425623	0.044769662	0.0707574
MGC4473	0.215788548	2.77E-05	8.43E-05
MGC45800	0.152216273	0.003291201	0.006745608
MGC57346	0.426150303	8.42E-18	1.89E-16
MGC70857	-0.196464843	0.00013976	0.000375394
MGC72080	-0.044491855	0.392825237	0.469232784
MGC87042	0.072632518	0.162676943	0.219317186
MGEA5	-0.217231573	2.44E-05	7.48E-05
MGLL	0.148706249	0.004096987	0.008222867
MGMT	-0.152364758	0.003260525	0.006691685
MGP	0.167458079	0.00120592	0.00271006
MGRN1	-0.136488877	0.008477898	0.015882705
MGST1	-0.278128962	5.13E-08	2.51E-07
MGST2	-0.362391081	5.90E-13	6.27E-12
MGST3	-0.105029348	0.043199457	0.068455264
MIA2	-0.229269283	8.17E-06	2.75E-05
MIA3	-0.438018536	8.00E-19	2.10E-17
MIAT	0.205836662	6.49E-05	0.000185564
MIA	0.144949598	0.005153285	0.010117646
MIB1	0.07689203	0.139344475	0.191740615
MIB2	-0.098386074	0.058324581	0.08939393
MICAL1	0.464242114	3.14E-21	1.16E-19
MICAL2	-0.188856327	0.000253866	0.000648816
MICAL3	-0.291688521	1.04E-08	5.72E-08
MICALCL	-0.191244656	0.000211008	0.000548642
MICALL1	0.26818046	1.57E-07	7.07E-07
MICALL2	0.415223568	6.78E-17	1.35E-15
MICA	0.012416434	0.811602238	0.849600115
MICB	0.384439186	1.63E-14	2.20E-13
MID1IP1	0.225366446	1.17E-05	3.82E-05
MID1	-0.026099496	0.616300208	0.68165294
MID2	-0.153943994	0.002949952	0.006114122
MIDN	0.23544737	4.55E-06	1.60E-05
MIER1	-0.045242741	0.384879356	0.461075944
MIER2	0.281627029	3.42E-08	1.73E-07
MIER3	-0.049567256	0.34104811	0.415277822
MIF4GD	-0.035823922	0.491509555	0.565545417

MIF	0.193901139	0.00017133	0.000452624
MIIP	0.086691772	0.095453387	0.13789865
MIMT1	-0.019250635	0.711698118	0.765699082
MINA	0.25392958	7.20E-07	2.90E-06
MINK1	0.17182142	0.000890252	0.002052257
MINPP1	-0.162374854	0.001701888	0.003709113
MIOS	-0.09830933	0.058522113	0.089641933
MIOX	0.348679998	4.80E-12	4.43E-11
MIPEP	-0.111052303	0.032482774	0.053086286
MIPOL1	0.164072004	0.001518619	0.003345194
MIP	-0.261984925	3.07E-07	1.32E-06
MIR155HG	0.295511932	6.52E-09	3.72E-08
MIR17HG	0.1867435	0.000298428	0.000752567
MIS12	0.015447427	0.766808487	0.812053937
MITD1	0.373381464	1.02E-13	1.23E-12
MITF	0.243520113	2.07E-06	7.71E-06
MIXL1	0.301753982	3.00E-09	1.80E-08
MKI67IP	0.256868001	5.29E-07	2.18E-06
MKI67	0.563155058	1.99E-32	4.62E-30
MKKS	0.389050681	7.43E-15	1.07E-13
MKL1	0.232929285	5.79E-06	2.00E-05
MKL2	-0.154890906	0.002776877	0.005789737
MKLN1	-0.376973676	5.67E-14	7.10E-13
MKNK1	0.157768376	0.002306094	0.004898914
MKNK2	-0.192755222	0.000187502	0.000491695
MKRN1	-0.089331848	0.085744936	0.125511434
MKRN2	0.060536836	0.244766987	0.312499866
MKRN3	-0.005634053	0.913873394	0.932693943
MKS1	-0.043142675	0.407348746	0.483623463
MKX	0.09633451	0.063798508	0.09677864
MLANA	-0.154653494	0.002819381	0.005869756
MLC1	0.016584651	0.750191488	0.798062414
MLEC	-0.005086516	0.922215594	0.939157709
MLF1IP	0.388935415	7.58E-15	1.08E-13
MLF1	0.200627349	9.99E-05	0.000275867
MLF2	-0.023434747	0.652765464	0.715391879
MLH1	0.127452637	0.014023785	0.024940354
MLH3	0.018645032	0.720382294	0.773329888
MLKL	0.279240146	4.51E-08	2.23E-07
MLL2	0.067201806	0.196533606	0.258319093
MLL3	-0.101949907	0.049741216	0.07768384
MLL4	0.247312439	1.42E-06	5.42E-06
MLL5	-0.000924597	0.985839173	0.989265501
MLLT10	0.105238477	0.042782768	0.06787595
MLLT11	0.262986328	2.76E-07	1.20E-06
MLLT1	0.002395444	0.963323093	0.971073352
MLLT3	0.242287876	2.34E-06	8.64E-06
MLLT4	0.027386223	0.599017091	0.665938531
MLLT6	0.15368829	0.002998341	0.00620731
MLL	0.104278736	0.044723283	0.070690039
MLNR	-0.02309683	0.657451637	0.719538071
MLN	-0.114081684	0.02801109	0.046503077
MLPH	-0.010576286	0.839110097	0.872663988
MLST8	0.106589563	0.040171513	0.064208671
MLXIPL	-0.399373862	1.22E-15	1.99E-14
MLXIP	-0.100194584	0.053826991	0.083308057
MLX	-0.18905754	0.000249963	0.000639662
MLYCD	-0.542815911	8.16E-30	1.14E-27

MMAA	-0.418221663	3.85E-17	7.88E-16
MMAB	-0.254260426	6.95E-07	2.81E-06
MMACHC	-0.356671558	1.43E-12	1.43E-11
MMADHC	-0.183160647	0.000391034	0.000964968
MMD2	0.102379171	0.048782451	0.076414844
MMD	0.436507453	1.09E-18	2.79E-17
MMEL1	0.259525332	4.00E-07	1.68E-06
MME	-0.264393245	2.37E-07	1.04E-06
MMGT1	0.020300121	0.696737211	0.753067736
MMP10	0.393007291	3.75E-15	5.64E-14
MMP11	0.413907255	8.67E-17	1.69E-15
MMP12	0.403654313	5.69E-16	9.76E-15
MMP13	0.208836501	5.04E-05	0.000146994
MMP14	0.405519403	4.06E-16	7.11E-15
MMP15	0.007634239	0.883485843	0.908019683
MMP16	0.309457379	1.12E-09	7.21E-09
MMP17	0.273855372	8.33E-08	3.92E-07
MMP19	0.128621733	0.013161244	0.023574745
MMP1	0.338754402	2.06E-11	1.72E-10
MMP20	0.097092225	0.061729511	0.093954872
MMP21	-0.011114481	0.831042909	0.865901508
MMP23A	0.128667252	0.013128627	0.023524781
MMP23B	0.177271497	0.000603195	0.001439143
MMP24	-0.144196761	0.005392361	0.010529957
MMP25	0.221729655	1.63E-05	5.17E-05
MMP26	0.112380343	0.030452557	0.050109511
MMP27	0.086685116	0.095478926	0.137925542
MMP28	0.210697103	4.30E-05	0.000127197
MMP2	0.260376786	3.65E-07	1.54E-06
MMP3	0.134162889	0.009677464	0.017922695
MMP7	0.381205162	2.81E-14	3.67E-13
MMP8	0.158660135	0.002175717	0.004645726
MMP9	0.463161804	3.99E-21	1.44E-19
MMRN1	-0.069760565	0.179990902	0.239195434
MMRN2	-0.253773497	7.31E-07	2.94E-06
MMS19	0.192670047	0.000188759	0.000494797
MN1	0.102597312	0.048301182	0.075768201
MNAT1	-0.008491745	0.870507769	0.89809714
MND1	0.356912484	1.38E-12	1.38E-11
MNDA	0.305982576	1.76E-09	1.09E-08
MNS1	0.151682363	0.003403679	0.006956104
MNT	0.158864827	0.002146756	0.004592748
MNX1	0.25402787	7.12E-07	2.87E-06
MOAP1	-0.134809445	0.009329754	0.017338373
MOB2	0.095132766	0.067196679	0.101323965
MOBKL1A	0.020342769	0.696131668	0.752527814
MOBKL1B	0.170125454	0.001002574	0.002288152
MOBKL2A	0.327734249	9.74E-11	7.35E-10
MOBKL2B	0.218193639	2.24E-05	6.93E-05
MOBKL2C	-0.016230323	0.755356934	0.802291486
MOBKL3	0.28518994	2.26E-08	1.18E-07
MOBP	0.215399631	2.86E-05	8.70E-05
MOCOS	-0.224541913	1.26E-05	4.09E-05
MOCS1	-0.416336349	5.50E-17	1.10E-15
MOCS2	-0.254734491	6.62E-07	2.68E-06
MOCS3	-0.089842641	0.083961652	0.123227055
MOGAT1	-0.398582986	1.41E-15	2.25E-14
MOGAT2	-0.384516281	1.61E-14	2.18E-13

MOGAT3	0.010659486	0.837861855	0.87159333
MOGS	0.016780801	0.747336769	0.795847632
MOG	0.094656416	0.068584085	0.103205086
MON1A	-0.146905147	0.004576212	0.009093088
MON1B	-0.163339997	0.001595324	0.003495605
MON2	-0.049428616	0.342401912	0.416671608
MORC1	0.104917557	0.043423597	0.068761219
MORC2	0.087227106	0.093416988	0.135309964
MORC3	-0.222071811	1.58E-05	5.03E-05
MORC4	-0.044718919	0.390412068	0.466826074
MORF4L1	0.253854793	7.25E-07	2.92E-06
MORF4L2	0.154818753	0.002789732	0.005812282
MORF4	0.127887341	0.01369743	0.02444725
MORN1	-0.129146357	0.012789615	0.022979588
MORN2	0.005394362	0.917524184	0.935509329
MORN3	0.287693375	1.68E-08	8.92E-08
MORN4	-0.064032281	0.218528662	0.283488182
MORN5	0.075871635	0.144685564	0.197955209
MOSC1	-0.079917742	0.124394091	0.173853845
MOSC2	-0.410034006	1.78E-16	3.30E-15
MOSPD1	0.261724041	3.16E-07	1.35E-06
MOSPD2	0.047030589	0.366358107	0.442097413
MOSPD3	-0.233251086	5.62E-06	1.95E-05
MOS	-0.043726858	0.401021446	0.477163545
MOV10L1	-0.099299508	0.056015551	0.086306405
MOV10	0.080578784	0.121300044	0.170094987
MOXD1	0.263028331	2.75E-07	1.19E-06
MPDU1	-0.070615133	0.174702256	0.233209485
MPDZ	-0.476557559	1.97E-22	8.66E-21
MPEG1	0.038274935	0.462334075	0.537281657
MPG	-0.142082139	0.006118151	0.011819802
MPHOSPH1	-0.050871388	0.328480823	0.402558744
MPHOSPH6	0.172913187	0.000824218	0.001913985
MPHOSPH8	-0.124744822	0.016214809	0.028400997
MPHOSPH9	-0.005296367	0.919017265	0.936648179
MPI	-0.261337031	3.30E-07	1.41E-06
MPL	-0.317534	3.89E-10	2.68E-09
MPND	-0.299534406	3.96E-09	2.33E-08
MPO	0.038125534	0.464084462	0.53893854
MPP1	0.003339545	0.948884788	0.960504179
MPP2	0.428114749	5.74E-18	1.32E-16
MPP3	0.270606529	1.20E-07	5.50E-07
MPP4	0.291460541	1.07E-08	5.87E-08
MPP5	-0.356147556	1.55E-12	1.54E-11
MPP6	0.256887711	5.28E-07	2.18E-06
MPP7	0.326344653	1.18E-10	8.79E-10
MPPE1	-0.025393682	0.62587219	0.690783699
MPPED1	-0.235423674	4.56E-06	1.61E-05
MPPED2	-0.050401678	0.332972287	0.407061482
MPRIP	0.147750642	0.004345284	0.008682723
MPST	-0.172453307	0.000851461	0.00197242
MPV17L2	0.114481971	0.027461725	0.045705304
MPV17L	-0.04130576	0.427626969	0.503985667
MPV17	0.468745519	1.16E-21	4.48E-20
MPZL1	0.538078805	3.12E-29	3.96E-27
MPZL2	0.019352845	0.710236095	0.764497703
MPZL3	-0.020123466	0.699247495	0.755240368
MPZ	0.043612191	0.402258744	0.478464309

MR1	0.018469019	0.722913079	0.775211237
MRAP2	0.173661021	0.000781631	0.001823805
MRAP	-0.04387913	0.399381894	0.475581955
MRAS	0.024456047	0.638685565	0.702744605
MRC1	-0.096059125	0.064564458	0.097806488
MRC2	0.322066998	2.12E-10	1.52E-09
MRE11A	0.261906516	3.10E-07	1.33E-06
MREG	-0.196474349	0.000139654	0.000375159
MRFAP1L1	0.03559134	0.494328322	0.568263637
MRFAP1	0.077966094	0.133887148	0.185216287
MRGPRD	-0.055693168	0.284647736	0.355733781
MRGPRE	0.092690808	0.074559132	0.110914504
MRGPRF	-0.033477941	0.520331228	0.59261598
MRGPRX1	0.074667434	0.151188112	0.205763736
MRGPRX2	-0.025910859	0.618852172	0.683792179
MRGPRX3	0.068688295	0.186793274	0.246983182
MRGPRX4	0.13475246	0.009359948	0.017390503
MRI1	-0.00719162	0.890197158	0.913464827
MRM1	0.072157676	0.165449853	0.222423374
MRO	-0.295198354	6.77E-09	3.85E-08
MRP63	-0.145830362	0.004885724	0.00965226
MRPL10	0.086258565	0.097127083	0.140082947
MRPL11	0.081371557	0.117668867	0.165644374
MRPL12	-0.010152939	0.8454677	0.877763694
MRPL13	0.243969061	1.98E-06	7.40E-06
MRPL14	0.021049055	0.686131784	0.743832794
MRPL15	0.102504618	0.048505199	0.076046319
MRPL16	-0.22699973	1.01E-05	3.33E-05
MRPL17	0.301325003	3.17E-09	1.90E-08
MRPL18	0.255331206	6.22E-07	2.53E-06
MRPL19	-0.158845731	0.002149443	0.004596522
MRPL1	-0.147883415	0.004309991	0.008619118
MRPL20	-0.028342781	0.586312351	0.65426061
MRPL21	0.077989133	0.133771917	0.185069732
MRPL22	0.205068621	6.92E-05	0.000196842
MRPL23	0.121462432	0.019267842	0.033191003
MRPL24	-0.019715918	0.705051245	0.760107739
MRPL27	0.109516825	0.034972973	0.056672488
MRPL28	-0.008611123	0.868703681	0.89658982
MRPL2	-0.142054443	0.006128212	0.011836944
MRPL30	0.097946153	0.05946442	0.0908962
MRPL32	-0.199126926	0.000112808	0.000308408
MRPL33	0.006685908	0.897874731	0.919569216
MRPL34	-0.119502107	0.021320421	0.036411953
MRPL35	0.003305941	0.949398442	0.960830307
MRPL36	0.128390988	0.013327687	0.023845009
MRPL37	-0.088967976	0.087033786	0.127164437
MRPL38	-0.004754112	0.927284272	0.943240095
MRPL39	-0.110694422	0.033049236	0.053901505
MRPL3	0.152968285	0.003138492	0.006473242
MRPL40	-0.298061426	4.76E-09	2.76E-08
MRPL41	-0.092261933	0.075917561	0.112716474
MRPL42P5	-0.084887558	0.102578388	0.147008607
MRPL42	0.298886536	4.30E-09	2.51E-08
MRPL43	-0.073658943	0.156802497	0.212331386
MRPL44	-0.270845585	1.17E-07	5.37E-07
MRPL45	-0.035176444	0.499377932	0.573136674
MRPL46	-0.391616143	4.78E-15	7.08E-14

MRPL47	0.386480562	1.15E-14	1.59E-13
MRPL48	0.218235897	2.23E-05	6.90E-05
MRPL49	-0.164772579	0.001448391	0.003206808
MRPL4	0.002885061	0.955833504	0.965477618
MRPL50	0.123671634	0.017162813	0.029895738
MRPL51	0.076297614	0.142437157	0.195382151
MRPL52	0.205697908	6.57E-05	0.000187569
MRPL53	0.006306033	0.903648181	0.924246794
MRPL54	-0.177198755	0.000606382	0.001445882
MRPL55	0.011511457	0.825103781	0.86110941
MRPL9	0.390167643	6.13E-15	8.91E-14
MRPS10	0.079156134	0.128034463	0.178159012
MRPS11	-0.05343826	0.304630591	0.377251858
MRPS12	0.204095239	7.51E-05	0.000212141
MRPS14	0.155434247	0.002681788	0.005618527
MRPS15	0.018549269	0.721758829	0.774348613
MRPS16	0.166675528	0.001272408	0.002847205
MRPS17	0.219720663	1.95E-05	6.11E-05
MRPS18A	-0.00424358	0.935074808	0.949806777
MRPS18B	-0.216458797	2.61E-05	7.98E-05
MRPS18C	-0.15862599	0.002180582	0.004654619
MRPS21	0.115553538	0.026036423	0.043584373
MRPS22	-0.203953183	7.60E-05	0.000214413
MRPS23	0.280462383	3.92E-08	1.96E-07
MRPS24	0.01650445	0.751359706	0.79897124
MRPS25	-0.016350755	0.753600022	0.800894141
MRPS26	0.04840849	0.352468353	0.427173289
MRPS27	-0.05159841	0.321606397	0.39537443
MRPS28	-0.011647756	0.823066902	0.859388827
MRPS2	-0.127666469	0.013862406	0.024694564
MRPS30	0.102793083	0.047872656	0.075172937
MRPS31	-0.278237426	5.06E-08	2.48E-07
MRPS33	-0.135145523	0.009153438	0.017044096
MRPS34	0.049424148	0.342445597	0.416699315
MRPS35	0.091476818	0.078456421	0.116105246
MRPS36	0.06676365	0.199474143	0.261838564
MRPS5	0.070219527	0.177135991	0.236015463
MRPS6	0.12780431	0.013759245	0.024535596
MRPS7	0.053494538	0.304120836	0.37672465
MRPS9	-0.028348185	0.586240941	0.65426061
MRRF	0.057652224	0.268019706	0.337792661
MRS2P2	0.081292407	0.118027537	0.166067137
MRS2	0.166551263	0.001283271	0.002867353
MRTO4	0.177018	0.00061437	0.001463528
MRVI1	0.069946217	0.178832005	0.237893376
MS4A10	0.102738519	0.047991771	0.075336227
MS4A12	0.044179123	0.396163571	0.472416833
MS4A13	0.093191472	0.07299841	0.108958891
MS4A14	0.249081161	1.18E-06	4.60E-06
MS4A15	0.183170548	0.000390745	0.000964373
MS4A1	0.129578916	0.01249019	0.02251876
MS4A2	-0.019583495	0.706940734	0.761680675
MS4A3	0.060978714	0.241333543	0.308709827
MS4A4A	0.094286054	0.06967892	0.104591881
MS4A6A	0.153212782	0.003090247	0.006379679
MS4A6E	-0.004359754	0.933301465	0.948247235
MS4A7	0.049122647	0.345401764	0.419809282
MS4A8B	0.373371302	1.02E-13	1.23E-12

MSC	0.293596344	8.24E-09	4.63E-08
MSGN1	0.053618241	0.30300236	0.375585678
MSH2	0.45072002	5.81E-20	1.75E-18
MSH3	-0.126550975	0.014722573	0.026057489
MSH4	0.215829422	2.76E-05	8.40E-05
MSH5	0.156145087	0.002561861	0.005398496
MSH6	0.290021382	1.27E-08	6.92E-08
MSI1	0.420454624	2.52E-17	5.29E-16
MSI2	0.260281439	3.69E-07	1.56E-06
MSL1	0.254193922	7.00E-07	2.83E-06
MSL2	-0.002556829	0.960854026	0.96922197
MSL3L2	0.166610806	0.001278055	0.002858264
MSL3	0.156577256	0.002491358	0.005261059
MSLNL	0.103589293	0.046162471	0.072685349
MSLN	0.208075475	5.38E-05	0.000155896
MSMB	0.125202453	0.015824705	0.027798631
MSMP	0.036993262	0.477468975	0.551940172
MSN	0.074975863	0.14950188	0.203816906
MSR1	0.207876332	5.47E-05	0.000158336
MSRA	-0.266558388	1.87E-07	8.34E-07
MSRB2	-0.190484421	0.000223855	0.000578647
MSRB3	0.180643773	0.000471385	0.001145935
MST1P2	-0.223762107	1.36E-05	4.37E-05
MST1P9	-0.117639138	0.023444196	0.039695332
MST1R	0.328852641	8.34E-11	6.38E-10
MST1	-0.356801448	1.40E-12	1.40E-11
MST4	0.365637814	3.54E-13	3.90E-12
MSTN	-0.078092723	0.133254751	0.18444794
MSTO1	0.316981361	4.19E-10	2.87E-09
MSTO2P	0.267477039	1.69E-07	7.59E-07
MSX1	0.147359941	0.004450655	0.008868368
MSX2P1	0.107046131	0.039320099	0.062938697
MSX2	0.179358259	0.000518107	0.001250988
MT1A	-0.265013884	2.22E-07	9.75E-07
MT1B	0.069927106	0.178951049	0.238019949
MT1DP	-0.073109372	0.159927564	0.216005216
MT1E	-0.096258028	0.06401048	0.097041076
MT1F	0.046167669	0.3752274	0.451324276
MT1G	-0.102813182	0.047828843	0.07511598
MT1H	0.11010702	0.033997241	0.055280201
MT1IP	-0.003394756	0.948040917	0.959796277
MT1L	-0.014317558	0.78342447	0.82644333
MT1M	-0.002566993	0.960698533	0.969114192
MT1X	-0.323375463	1.77E-10	1.29E-09
MT2A	-0.178998546	0.000531931	0.001281704
MT3	0.098088845	0.059092705	0.090411249
MT4	-0.002060641	0.968446421	0.975103083
MTA1	0.007039225	0.892509725	0.915203764
MTA2	0.454057792	2.86E-20	9.03E-19
MTA3	0.439676468	5.72E-19	1.53E-17
MTAP	0.171048396	0.000939924	0.002156036
MTBP	0.518120255	7.14E-27	6.21E-25
MTCH1	0.181087234	0.000456198	0.001111863
MTCH2	-0.038466699	0.460092739	0.534926615
MTCP1NB	-0.100154411	0.053923671	0.083444723
MTCP1	0.145022198	0.005130742	0.010079346
MTDH	0.116363103	0.025002282	0.042008557
MTERFD1	0.200393315	0.000101777	0.000280716

MTERFD2	0.004889117	0.925225268	0.941487633
MTERFD3	0.070130816	0.177685173	0.236689882
MTERF	0.076726262	0.140201724	0.192733835
MTF1	-0.073539795	0.157476072	0.213142082
MTF2	0.389558791	6.81E-15	9.84E-14
MTFMT	-0.21937403	2.01E-05	6.29E-05
MTFR1	-0.095803213	0.065283008	0.098762373
MTG1	0.051923106	0.318566654	0.391855081
MTHFD1L	0.469473775	9.82E-22	3.85E-20
MTHFD1	-0.554279716	2.89E-31	5.05E-29
MTHFD2L	0.03408565	0.512782831	0.585491721
MTHFD2	0.367309135	2.71E-13	3.05E-12
MTHFR	0.092518176	0.075103535	0.111632666
MTHFSD	-0.101631745	0.050461946	0.07867451
MTHFS	-0.15802952	0.002267195	0.004823479
MTIF2	-0.158078084	0.002260028	0.004809771
MTIF3	-0.088824259	0.087547107	0.127801968
MTL5	0.386017242	1.25E-14	1.71E-13
MTM1	-0.273874503	8.31E-08	3.91E-07
MTMR10	-0.35856193	1.07E-12	1.09E-11
MTMR11	0.019796167	0.703907078	0.759130185
MTMR12	-0.133790756	0.009882767	0.018267256
MTMR14	0.055850389	0.283288172	0.35419022
MTMR15	-0.357084469	1.34E-12	1.35E-11
MTMR1	0.108762496	0.036254633	0.058506666
MTMR2	0.478604502	1.23E-22	5.57E-21
MTMR3	-0.068410519	0.188585914	0.249022906
MTMR4	-0.328681015	8.54E-11	6.52E-10
MTMR6	-0.145841759	0.004882345	0.009647498
MTMR7	0.269099444	1.42E-07	6.43E-07
MTMR8	0.151108905	0.003528361	0.007182915
MTMR9L	-0.059555584	0.252513129	0.321049544
MTMR9	0.096035861	0.064629511	0.097890148
MTNR1A	0.20690954	5.93E-05	0.000170864
MTNR1B	0.221741433	1.63E-05	5.17E-05
MTO1	-0.074430221	0.152494782	0.207345474
MTOR	-0.115490572	0.026118377	0.043699531
MTP18	0.235142213	4.69E-06	1.65E-05
MTPAP	0.154535591	0.002840708	0.005911687
MTRF1L	0.098323559	0.058485448	0.089606021
MTRF1	-0.107446264	0.038586523	0.061863891
MTRR	0.034874525	0.503069601	0.576449591
MTR	-0.088464073	0.088844299	0.129458464
MTSS1L	-0.034342844	0.509605402	0.582498067
MTSS1	-0.215414691	2.86E-05	8.69E-05
MTTP	-0.43463925	1.58E-18	3.98E-17
MTUS1	-0.0758161	0.144980676	0.198313771
MTUS2	0.049329259	0.343374227	0.417727244
MTVR2	0.062551115	0.229390193	0.295462044
MTX1	0.144333446	0.005348227	0.010456073
MTX2	0.332625228	4.92E-11	3.89E-10
MTX3	0.136692259	0.008379588	0.015712292
MUC12	0.355837323	1.63E-12	1.61E-11
MUC13	0.337961469	2.30E-11	1.91E-10
MUC15	0.056936613	0.274014878	0.344281307
MUC16	0.018541558	0.721869722	0.774425881
MUC17	0.176503816	0.00063763	0.001515674
MUC1	0.352263721	2.80E-12	2.68E-11

MUC20	0.187899018	0.000273225	0.000694906
MUC21	0.173837724	0.000771869	0.001803352
MUC2	0.102011849	0.049601904	0.077491306
MUC4	0.03423693	0.510912642	0.583657414
MUC5B	0.188309685	0.000264758	0.000674316
MUC6	0.228148761	9.07E-06	3.02E-05
MUC7	-0.104954613	0.043349191	0.06865977
MUCL1	0.033978018	0.514115572	0.586811644
MUDENG	-0.100641325	0.052761538	0.081843445
MUL1	-0.223278639	1.42E-05	4.55E-05
MUM1L1	0.231033498	6.93E-06	2.36E-05
MUM1	-0.136703287	0.008374286	0.01570632
MURC	0.270238825	1.25E-07	5.72E-07
MUS81	0.247312645	1.42E-06	5.42E-06
MUSK	0.025501559	0.624405084	0.68927789
MUSTN1	0.098241768	0.058696469	0.089880942
MUTED	0.062885858	0.226902643	0.292541707
MUTYH	0.139815733	0.006992231	0.013349231
MUT	-0.375864254	6.80E-14	8.41E-13
MVD	-0.059326462	0.254346092	0.323124772
MVK	-0.145786996	0.004898599	0.009671941
MVP	0.310052404	1.04E-09	6.71E-09
MX1	0.131029515	0.011530703	0.020945989
MX2	0.305454556	1.88E-09	1.16E-08
MXD1	0.195432853	0.000151748	0.000405082
MXD3	0.424299137	1.21E-17	2.65E-16
MXD4	0.060198837	0.247416224	0.31538966
MXI1	-0.466405706	1.95E-21	7.32E-20
MXRA5	0.173152766	0.000810347	0.001885289
MXRA7	0.231595061	6.57E-06	2.25E-05
MXRA8	0.24762008	1.37E-06	5.27E-06
MYADML2	0.197308687	0.000130624	0.000352709
MYADML	0.013339433	0.797889072	0.838459759
MYADM	0.208374279	5.24E-05	0.000152379
MYBBP1A	0.168214417	0.001144711	0.002585594
MYBL1	0.396866218	1.91E-15	2.99E-14
MYBL2	0.617032588	2.68E-40	6.50E-37
MYBPC1	0.110434053	0.033466627	0.05451976
MYBPC2	0.218414292	2.19E-05	6.81E-05
MYBPC3	0.26500795	2.22E-07	9.76E-07
MYBPHL	0.229359746	8.10E-06	2.72E-05
MYBPH	-0.015489285	0.766194902	0.811576714
MYB	0.366587484	3.04E-13	3.39E-12
MYCBP2	-0.105208131	0.042843021	0.067955308
MYCBPAP	0.184137814	0.000363427	0.000902429
MYCBP	0.247312733	1.42E-06	5.42E-06
MYCL1	-0.512683916	2.95E-26	2.39E-24
MYCNOS	0.10347574	0.046403192	0.073055508
MYCN	0.139649269	0.007060633	0.013464337
MYCT1	-0.156839748	0.002449403	0.005180504
MYC	0.041242245	0.428338446	0.504693549
MYD88	-0.306824373	1.58E-09	9.89E-09
MYEF2	0.209314981	4.84E-05	0.000141654
MYEOV2	0.218423101	2.19E-05	6.80E-05
MYEOV	0.050489098	0.332133384	0.406166035
MYF6	0.127533627	0.013962468	0.024844614
MYH10	0.031070159	0.5507891	0.621971458
MYH11	0.07686924	0.139462093	0.191889206

MYH13	0.1643314	0.00149226	0.003293321
MYH14	-0.077709477	0.135175808	0.186687865
MYH15	0.275507374	6.91E-08	3.30E-07
MYH16	0.085179072	0.101399447	0.145538889
MYH1	0.122128317	0.018610881	0.032178959
MYH2	0.100245907	0.053703685	0.083123674
MYH3	-0.177301663	0.000601878	0.001436689
MYH4	0.204622993	7.18E-05	0.000203733
MYH6	0.234662962	4.91E-06	1.72E-05
MYH7B	-0.30328112	2.48E-09	1.50E-08
MYH7	0.288591763	1.51E-08	8.09E-08
MYH8	0.153945489	0.002949671	0.006114122
MYH9	0.010565124	0.839277595	0.872792621
MYL10	0.091399314	0.078710738	0.116429709
MYL12A	0.027721514	0.594549715	0.66202429
MYL12B	-0.02815901	0.588743473	0.656348488
MYL1	-0.000739654	0.988671487	0.991430188
MYL2	0.086661082	0.095571195	0.138048822
MYL3	0.054542715	0.294729902	0.366654746
MYL4	0.189571599	0.000240246	0.000616855
MYL5	-0.17480047	0.000720638	0.001693189
MYL6B	0.311803006	8.29E-10	5.42E-09
MYL6	0.182260692	0.000418175	0.001025338
MYL7	0.059897323	0.24979631	0.317942888
MYL9	0.129388404	0.012621296	0.022717877
MYLIP	0.224696965	1.25E-05	4.05E-05
MYLK2	0.452819186	3.72E-20	1.15E-18
MYLK3	0.029620272	0.569541902	0.639162561
MYLK4	0.00532116	0.918639485	0.936454783
MYLK	-0.411390151	1.38E-16	2.61E-15
MYLPF	0.046902497	0.36766637	0.443488098
MYNN	0.018633284	0.720551112	0.773427761
MYO10	0.066394818	0.201974143	0.264719006
MYO15A	-0.086806981	0.095012169	0.137350876
MYO15B	0.034277515	0.510411518	0.583218713
MYO16	-0.47014501	8.45E-22	3.35E-20
MYO18A	-0.229388789	8.08E-06	2.72E-05
MYO18B	0.149558774	0.003886376	0.007849983
MYO19	0.455967978	1.90E-20	6.15E-19
MYO1A	0.178684688	0.000544271	0.001309221
MYO1B	-0.456370592	1.74E-20	5.70E-19
MYO1C	0.118560811	0.022371669	0.038050746
MYO1D	0.151539728	0.003434312	0.00701439
MYO1E	0.178345051	0.000557924	0.001339151
MYO1F	0.202549873	8.53E-05	0.000238617
MYO1G	0.270104511	1.27E-07	5.80E-07
MYO1H	0.105452021	0.042360779	0.067270741
MYO3A	0.216729597	2.55E-05	7.81E-05
MYO3B	-0.148864204	0.004057203	0.008151231
MYO5A	0.197217527	0.000131583	0.000355058
MYO5B	-0.215317768	2.88E-05	8.76E-05
MYO5C	0.195126322	0.000155491	0.000414295
MYO6	0.303178643	2.51E-09	1.52E-08
MYO7A	-0.034994848	0.501596654	0.575059478
MYO7B	0.146198459	0.004777659	0.009459405
MYO9A	-0.18915644	0.000248066	0.000635541
MYO9B	0.145642951	0.004941584	0.009750049
MYOCD	-0.017818215	0.732296648	0.783123829

MYOC	0.081961622	0.11502174	0.162388428
MYOD1	0.148374164	0.004181781	0.008377017
MYOF	0.188941278	0.000252211	0.000645083
MYOG	0.121772893	0.018959071	0.032715688
MYOM1	0.037445517	0.472097827	0.546873643
MYOM2	-0.088148295	0.089994201	0.13090424
MYOM3	0.228584792	8.71E-06	2.91E-05
MYOT	0.018679275	0.719890288	0.772926668
MYOZ1	0.10892142	0.035981344	0.058112715
MYOZ2	-0.034288779	0.510272484	0.583093291
MYOZ3	0.231816697	6.43E-06	2.21E-05
MYPN	0.196197993	0.000142772	0.000382916
MYPOP	0.175949008	0.000663641	0.001572351
MYRIP	-0.461288262	6.01E-21	2.12E-19
MYSM1	0.003814827	0.941622241	0.954996604
MYST1	-0.058265328	0.262955202	0.332461353
MYST2	0.097797741	0.059853086	0.091448208
MYST3	0.037298062	0.473845398	0.5486428
MYST4	-0.052075504	0.317146406	0.390373228
MYT1L	0.041019263	0.430841641	0.507133067
MYT1	0.114960908	0.026816607	0.044743755
MZF1	0.03233398	0.534693606	0.606511867
N4BP1	-0.14863246	0.004115693	0.008256251
N4BP2L1	-0.387631836	9.48E-15	1.33E-13
N4BP2L2	-0.094363374	0.069449181	0.104302041
N4BP2	-0.079914799	0.124407998	0.173853845
N4BP3	0.369597303	1.88E-13	2.17E-12
N6AMT1	0.01326912	0.798931596	0.83916927
N6AMT2	-0.078863544	0.129454737	0.17986183
NAA10	0.168055001	0.001157369	0.002611521
NAA11	0.115898298	0.025591608	0.042911877
NAA15	0.167841624	0.001174514	0.002646609
NAA16	0.105761804	0.04175485	0.066462139
NAA20	0.116968138	0.024252718	0.040894004
NAA25	0.371145766	1.47E-13	1.72E-12
NAA30	-0.192592956	0.000189904	0.000497405
NAA35	0.179673138	0.000506281	0.001224492
NAA38	0.209808127	4.64E-05	0.000136207
NAA40	0.48105094	6.96E-23	3.26E-21
NAA50	0.246735007	1.50E-06	5.72E-06
NAAA	-0.19506111	0.000156298	0.000416279
NAALAD2	0.253199224	7.76E-07	3.11E-06
NAALADL1	0.363813199	4.72E-13	5.10E-12
NAALADL2	-0.267602223	1.67E-07	7.49E-07
NAB1	0.061162039	0.239919041	0.307196294
NAB2	0.128960842	0.012919959	0.023196775
NACA2	0.110706133	0.033030568	0.053875469
NACAD	0.174793596	0.000720992	0.001693622
NACAP1	0.148769951	0.0040809	0.008193882
NACA	0.199616778	0.000108415	0.000297416
NACC1	0.277383364	5.58E-08	2.71E-07
NACC2	0.058009658	0.265059092	0.334632905
NADK	-0.152557528	0.003221087	0.006619169
NADSYN1	0.08220051	0.113963412	0.161061421
NAE1	0.244435103	1.89E-06	7.09E-06
NAF1	-0.059467111	0.253219817	0.321847977
NAGA	-0.242631677	2.26E-06	8.37E-06
NAGK	0.34865059	4.82E-12	4.45E-11

NAGLU	-0.26901219	1.43E-07	6.48E-07
NAGPA	-0.00644339	0.901559996	0.922564697
NAGS	-0.378780313	4.20E-14	5.35E-13
NAIF1	0.012254872	0.814008642	0.851582218
NAIP	0.269168768	1.41E-07	6.38E-07
NALCN	0.273325881	8.84E-08	4.14E-07
NAMPT	-0.070494227	0.175443409	0.234057697
NANOG	0.017874307	0.731486293	0.782509259
NANOS1	0.147244331	0.004482274	0.008922448
NANOS2	0.125921631	0.015228253	0.026861808
NANOS3	0.199709326	0.000107603	0.000295352
NANP	0.132400762	0.010684206	0.019569037
NANS	0.22756179	9.57E-06	3.17E-05
NAP1L1	0.432228296	2.55E-18	6.18E-17
NAP1L2	-0.007921381	0.879136395	0.90465678
NAP1L3	0.190667673	0.000220693	0.000571215
NAP1L4	0.354129399	2.11E-12	2.06E-11
NAP1L5	-0.240641148	2.75E-06	1.01E-05
NAP1L6	0.112920934	0.02965778	0.048939145
NAPA	-0.18691587	0.000294535	0.00074341
NAPB	0.07661251	0.140792313	0.193465613
NAPEPLD	-0.291493036	1.06E-08	5.85E-08
NAPG	0.219984356	1.91E-05	5.98E-05
NAPRT1	-0.084960435	0.102282648	0.146616413
NAPSA	-0.076764923	0.140001432	0.192538212
NAPSB	0.209864842	4.62E-05	0.000135649
NARFL	-0.014191369	0.785286502	0.82805683
NARF	0.120838743	0.019901388	0.034164193
NARG2	-0.065773967	0.206233604	0.269503828
NARS2	-0.281357654	3.53E-08	1.78E-07
NARS	0.255595578	6.05E-07	2.47E-06
NASP	0.281266661	3.57E-08	1.80E-07
NAT10	0.304377852	2.16E-09	1.32E-08
NAT14	0.218428414	2.19E-05	6.80E-05
NAT15	-0.089556853	0.084955677	0.124502501
NAT1	-0.147674141	0.004365737	0.008713978
NAT2	-0.395606493	2.38E-15	3.68E-14
NAT6	-0.312594946	7.48E-10	4.92E-09
NAT8B	0.107784164	0.037976093	0.060995519
NAT8L	0.265178763	2.18E-07	9.60E-07
NAT8	-0.132940394	0.010366474	0.019055259
NAT9	0.204652628	7.17E-05	0.000203318
NAV1	0.176180961	0.000652649	0.001548603
NAV2	0.164275159	0.001497939	0.003304756
NAV3	0.035894554	0.490655246	0.564758136
NBAS	-0.164241739	0.001501323	0.003310755
NBEAL1	-0.11132488	0.032056909	0.052472084
NBEAL2	0.464704819	2.84E-21	1.05E-19
NBEA	0.20072452	9.91E-05	0.000273802
NBL1	0.229081002	8.31E-06	2.79E-05
NBLA00301	0.111162089	0.032310671	0.052844035
NBN	0.180681361	0.00047008	0.00114318
NBPF10	0.059825834	0.250362955	0.318582703
NBPF14	0.128737242	0.013078615	0.02345204
NBPF15	0.25140026	9.34E-07	3.70E-06
NBPF16	0.214557312	3.08E-05	9.33E-05
NBPF1	-0.017795987	0.732617859	0.783299146
NBPF22P	0.125889783	0.015254243	0.026891405

NBPF3	0.009397068	0.856843646	0.88753908
NBPF4	0.179041779	0.000530252	0.001278121
NBPF6	0.119055441	0.021813797	0.03717807
NBPF7	0.045822796	0.378808718	0.454944977
NBPF9	0.0751063	0.148793076	0.202947806
NBR1	-0.301779523	2.99E-09	1.80E-08
NBR2	-0.20808065	5.38E-05	0.000155873
NCALD	0.311001313	9.21E-10	5.99E-09
NCAM1	0.13730317	0.008090368	0.015234055
NCAM2	0.022794447	0.661656453	0.723107129
NCAN	-0.184848204	0.000344508	0.000859523
NCAPD2	0.489515334	9.43E-24	4.95E-22
NCAPD3	0.265129182	2.19E-07	9.64E-07
NCAPG2	0.393785827	3.28E-15	4.97E-14
NCAPG	0.59034118	3.25E-36	2.23E-33
NCAPH2	0.223589306	1.38E-05	4.43E-05
NCAPH	0.574252718	6.24E-34	2.14E-31
NCBP1	0.140179282	0.006844901	0.013090537
NCBP2	0.453326898	3.34E-20	1.04E-18
NCCRP1	0.294533868	7.35E-09	4.16E-08
NCDN	0.36978732	1.82E-13	2.11E-12
NCEH1	0.400443252	1.01E-15	1.66E-14
NCF1B	0.245946246	1.63E-06	6.15E-06
NCF1C	0.219440893	2.00E-05	6.25E-05
NCF1	0.257841887	4.78E-07	1.98E-06
NCF2	0.353131614	2.46E-12	2.38E-11
NCF4	0.282364451	3.14E-08	1.60E-07
NCK1	-0.034725539	0.504896585	0.578177259
NCK2	0.430822257	3.37E-18	8.01E-17
NCKAP1L	0.252852778	8.05E-07	3.21E-06
NCKAP1	0.1860539	0.000314484	0.000790179
NCKAP5L	0.372737571	1.13E-13	1.35E-12
NCKAP5	0.019161085	0.712979935	0.766664126
NCKIPSD	-0.01777978	0.732852081	0.783465477
NCLN	0.086095124	0.097764575	0.14087999
NCL	0.275495765	6.92E-08	3.31E-07
NCOA1	-0.05016639	0.335236942	0.409202276
NCOA2	-0.146826733	0.004598181	0.009133097
NCOA3	0.097559695	0.060480869	0.0923012
NCOA4	-0.356944561	1.37E-12	1.37E-11
NCOA5	0.371047141	1.49E-13	1.75E-12
NCOA6	0.214828033	3.01E-05	9.13E-05
NCOA7	0.118450355	0.022497921	0.038249154
NCOR1	-0.022156363	0.670564201	0.730877558
NCOR2	0.263604887	2.58E-07	1.12E-06
NCR1	-0.098908177	0.056995325	0.087605961
NCR2	0.131588536	0.011178703	0.020379038
NCR3	0.086705942	0.095399032	0.137840115
NCRNA0002	0.009182032	0.860085492	0.890202783
NCRNA0002	0.097489765	0.060666319	0.092513351
NCRNA0003	0.256862929	5.30E-07	2.18E-06
NCRNA0005	-0.05953504	0.252677103	0.321211343
NCRNA0005	0.072908663	0.161080485	0.217341377
NCRNA0008	0.015300672	0.768960836	0.813900637
NCRNA0008	0.317899068	3.71E-10	2.56E-09
NCRNA0008	0.167451974	0.001206426	0.002710853
NCRNA0008	0.015655533	0.763759408	0.809642682
NCRNA0009	0.25317282	7.78E-07	3.11E-06

NCRNA0009	-0.188522761	0.000260461	0.000664223
NCRNA0009	0.083540469	0.108167957	0.153944995
NCRNA0009	0.262593178	2.88E-07	1.24E-06
NCRNA0009	0.053450508	0.304519598	0.377161295
NCRNA0010	0.047319158	0.363421434	0.438971431
NCRNA0010	0.169763539	0.001028175	0.00234202
NCRNA0011	0.088222732	0.089722071	0.130546531
NCRNA0011	0.117684507	0.023390379	0.039624416
NCRNA0011	0.132812783	0.010440845	0.019170924
NCRNA0011	0.233206603	5.64E-06	1.96E-05
NCRNA0011	-0.008506783	0.870280467	0.897909134
NCRNA0011	-0.062655307	0.228613853	0.294557314
NCRNA0011	-0.000685402	0.989502354	0.99202353
NCRNA0012	-0.029108646	0.576230944	0.645313942
NCRNA0015	0.371367303	1.41E-13	1.66E-12
NCRNA0015	-0.082945637	0.110711327	0.15705982
NCRNA0015	0.202534546	8.54E-05	0.000238851
NCRNA0015	0.10907821	0.03571344	0.05774218
NCRNA0016	0.008463671	0.870932121	0.898388456
NCRNA0016	-0.043545597	0.402978366	0.479177212
NCRNA0016	0.059068515	0.256420656	0.325355857
NCRNA0016	0.089516271	0.085097594	0.124646295
NCRNA0016	-0.125076163	0.015931531	0.02796644
NCRNA0016	0.303851732	2.31E-09	1.41E-08
NCRNA0017	-0.037632461	0.469887322	0.544661386
NCRNA0017	0.219201848	2.05E-05	6.38E-05
NCRNA0017	-0.303327008	2.46E-09	1.50E-08
NCRNA0017	0.14341062	0.005652615	0.010988621
NCRNA0017	0.029980598	0.564853232	0.634866352
NCRNA0018	-0.38112775	2.84E-14	3.71E-13
NCRNA0018	-0.151014636	0.003549249	0.007223961
NCRNA0018	0.141756575	0.00623736	0.012033767
NCRNA0018	-0.103460271	0.046436067	0.073101487
NCRNA0018	0.177631717	0.000587638	0.0014059
NCRNA0018	0.136074342	0.00868146	0.016228961
NCRNA0020	0.031396736	0.546607303	0.617774476
NCRNA0020	0.219487298	1.99E-05	6.23E-05
NCRNA0020	0.049303035	0.343631145	0.417963225
NCRNA0020	0.0303054	0.560642716	0.631023965
NCRNA0020	-0.027703119	0.594794416	0.662130887
NCRNA0020	0.016739979	0.747930596	0.796337627
NCRNA0021	0.058105537	0.264268763	0.333825521
NCRNA0023	-0.089479431	0.085226591	0.124816889
NCRNA0023	-0.015247362	0.769743128	0.814578562
NCS1	0.358898018	1.02E-12	1.04E-11
NCSTN	0.054826969	0.2922169	0.363947907
NDC80	0.576858871	2.72E-34	1.10E-31
NDE1	0.290965484	1.13E-08	6.22E-08
NDEL1	-0.249740548	1.11E-06	4.33E-06
NDFIP1	-0.130740828	0.011716288	0.021265661
NDFIP2	-0.226368017	1.07E-05	3.51E-05
NDNL2	-0.046676921	0.369977322	0.445789779
NDN	0.143477796	0.005629943	0.01095096
NDOR1	0.367120999	2.79E-13	3.13E-12
NDP	0.266413021	1.90E-07	8.46E-07
NDRG1	0.325481606	1.33E-10	9.80E-10
NDRG2	-0.456533093	1.68E-20	5.52E-19
NDRG3	0.428699863	5.12E-18	1.19E-16

NDRG4	0.287943855	1.63E-08	8.68E-08
NDST1	0.053847025	0.30094096	0.373355698
NDST2	0.02387055	0.646741916	0.709962114
NDST3	-0.079861817	0.124658635	0.174167146
NDST4	0.146011464	0.004832284	0.009558059
NDUFA10	-0.245543087	1.69E-06	6.38E-06
NDUFA11	-0.022523192	0.665437471	0.726123969
NDUFA12	0.279194622	4.53E-08	2.24E-07
NDUFA13	0.123799362	0.017047501	0.029723466
NDUFA1	0.036963774	0.477820336	0.552218166
NDUFA2	0.058464022	0.261328117	0.330866142
NDUFA3	0.090303113	0.082379791	0.121155337
NDUFA4L2	0.174378619	0.000742686	0.001740477
NDUFA4	-0.064835737	0.212793186	0.276949491
NDUFA5	-0.324282336	1.57E-10	1.14E-09
NDUFA6	0.058136798	0.264011425	0.333542749
NDUFA7	-0.028565557	0.583371468	0.651782087
NDUFA8	0.107659857	0.0381997	0.061312796
NDUFA9	0.100449932	0.053215831	0.082454012
NDUFAB1	-0.069023148	0.184648992	0.244423735
NDUFAF1	-0.316905636	4.23E-10	2.90E-09
NDUFAF2	0.248110182	1.31E-06	5.04E-06
NDUFAF3	-0.02060068	0.692473848	0.74926198
NDUFAF4	0.163495085	0.001578782	0.003462025
NDUFB10	-0.087863233	0.091042471	0.132303457
NDUFB11	0.085270631	0.10103139	0.145083785
NDUFB1	-0.06542934	0.208625839	0.27221928
NDUFB2	0.042071092	0.419108009	0.495135489
NDUFB3	0.172263429	0.00086295	0.001995554
NDUFB4	0.228419036	8.84E-06	2.95E-05
NDUFB5	0.013357479	0.797621572	0.838277601
NDUFB6	-0.109535964	0.034940961	0.05662522
NDUFB7	-0.013464991	0.796028332	0.837035598
NDUFB8	-0.118316283	0.022651997	0.038491392
NDUFB9	0.151212838	0.003505461	0.007142866
NDUFC1	-0.090093255	0.083097707	0.122121017
NDUFC2	-0.088382812	0.089139076	0.129798911
NDUFS1	-0.202136313	8.82E-05	0.000246237
NDUFS2	-0.145142508	0.00509358	0.01001424
NDUFS3	0.107429226	0.03861752	0.06190108
NDUFS4	0.00710808	0.89146476	0.914508802
NDUFS5	0.096812168	0.062487688	0.094970989
NDUFS6	0.243500961	2.08E-06	7.72E-06
NDUFS7	-0.101040944	0.051823402	0.080532435
NDUFS8	0.174398711	0.000741622	0.001738392
NDUFV1	-0.19038363	0.000225612	0.00058266
NDUFV2	-0.090551211	0.081537525	0.120049559
NDUFV3	0.043458393	0.40392186	0.48009852
NEAT1	0.06952007	0.181500399	0.240880084
NEBL	0.266513229	1.88E-07	8.38E-07
NEB	0.229585233	7.93E-06	2.67E-05
NECAB1	-0.024256887	0.641421277	0.70513159
NECAB2	-0.220190879	1.87E-05	5.88E-05
NECAB3	0.093590228	0.071774504	0.107319921
NECAP1	-0.071716338	0.168058734	0.225519742
NECAP2	0.105193704	0.042871693	0.067995372
NEDD1	0.385700042	1.32E-14	1.80E-13
NEDD4L	0.007818123	0.880700065	0.905797971

NEDD4	-0.345121616	8.13E-12	7.27E-11
NEDD8	0.087619664	0.091945885	0.133479991
NEDD9	-0.056422248	0.278380039	0.348973517
NEFH	0.337203385	2.57E-11	2.12E-10
NEFL	0.213532482	3.37E-05	0.000101324
NEFM	0.201797163	9.07E-05	0.000252468
NEGR1	0.038886029	0.455212791	0.530491326
NEIL1	-0.192862839	0.000185924	0.000487944
NEIL2	-0.099385165	0.055802966	0.086025434
NEIL3	0.498774401	9.91E-25	6.15E-23
NEK10	0.161117463	0.001850499	0.004009313
NEK11	0.011686176	0.82249296	0.858924608
NEK1	-0.231507669	6.62E-06	2.26E-05
NEK2	0.550436892	8.98E-31	1.45E-28
NEK3	-0.259029798	4.21E-07	1.77E-06
NEK4	0.062979728	0.226208525	0.291778973
NEK5	-0.128440811	0.013291592	0.023788482
NEK6	0.029106877	0.576254143	0.645313942
NEK7	-0.031977489	0.539209693	0.610943026
NEK8	0.098757593	0.057376105	0.088123257
NEK9	-0.241950933	2.42E-06	8.91E-06
NELF	0.029030267	0.577258924	0.646330146
NELL1	0.266215987	1.94E-07	8.63E-07
NELL2	0.115953768	0.025520654	0.042803711
NENF	0.064380316	0.216030777	0.280668133
NEO1	0.163503663	0.001577872	0.003460624
NES	0.185106733	0.000337854	0.000844084
NET1	-0.116759453	0.024509031	0.041284707
NETO1	-0.112451667	0.030346664	0.049968282
NETO2	0.1897047	0.000237788	0.000611174
NEU1	0.198484785	0.000118822	0.000323693
NEU2	-0.082696411	0.111790842	0.158433318
NEU3	-0.173488107	0.000791293	0.001844406
NEU4	-0.11436415	0.027622455	0.045926769
NEURL1B	0.179956758	0.000495844	0.001201242
NEURL2	-0.068285265	0.189398363	0.249963179
NEURL3	0.488470448	1.21E-23	6.29E-22
NEURL4	0.118522023	0.022415935	0.038119526
NEURL	0.455967922	1.90E-20	6.15E-19
NEUROD1	0.117880829	0.023158732	0.039275412
NEUROD2	0.18463703	0.000350034	0.000871999
NEUROD4	0.208639858	5.13E-05	0.000149249
NEUROD6	0.037231134	0.474639773	0.549402914
NEUROG1	0.143691982	0.0055582	0.010826209
NEUROG2	0.06398918	0.218839439	0.283780466
NEUROG3	0.281214491	3.59E-08	1.81E-07
NEXN	0.080011261	0.123952681	0.173326687
NF1P1	-0.021850903	0.674844942	0.734057381
NF1	0.014690383	0.77793037	0.821517563
NF2	0.212040026	3.83E-05	0.000114285
NFAM1	0.235201314	4.66E-06	1.64E-05
NFASC	-0.015178532	0.770753529	0.81541148
NFAT5	0.017059271	0.743289909	0.792243409
NFATC1	0.206069872	6.37E-05	0.00018242
NFATC2IP	0.131541456	0.011207977	0.02042306
NFATC2	0.117470115	0.023645633	0.039992215
NFATC3	-0.302758205	2.65E-09	1.60E-08
NFATC4	0.316580833	4.42E-10	3.01E-09

NFE2L1	-0.280673058	3.82E-08	1.92E-07
NFE2L2	-0.31363688	6.52E-10	4.33E-09
NFE2L3	0.463818124	3.45E-21	1.26E-19
NFE2	0.037021047	0.477138037	0.551621633
NFIA	-0.545265196	4.04E-30	5.96E-28
NFIB	-0.178889518	0.000536188	0.001291336
NFIC	-0.286449256	1.94E-08	1.02E-07
NFIL3	-0.091098322	0.079704733	0.117751405
NFIX	-0.405464156	4.10E-16	7.18E-15
NFKB1	-0.011354597	0.827449378	0.862879319
NFKB2	0.335679408	3.19E-11	2.59E-10
NFKBIA	-0.126361882	0.014872937	0.02627693
NFKBIB	0.076134752	0.143293603	0.196414969
NFKBID	0.351712466	3.04E-12	2.90E-11
NFKBIE	0.416175258	5.67E-17	1.13E-15
NFKBIL1	0.083442818	0.108582302	0.154490546
NFKBIL2	0.479033842	1.11E-22	5.09E-21
NFKBIZ	0.182656759	0.000406021	0.000998735
NFRKB	0.013739231	0.791968167	0.833690348
NFS1	-0.24652702	1.53E-06	5.83E-06
NFU1	0.151579551	0.003425734	0.006998306
NFX1	-0.160372245	0.001944088	0.00419384
NFXL1	0.056114053	0.281018002	0.351793429
NFYA	0.278980591	4.65E-08	2.29E-07
NFYB	-0.152334977	0.003266657	0.006701569
NFYC	0.198853131	0.000115337	0.000314715
NGB	0.142306276	0.006037272	0.011672606
NGDN	0.047468739	0.361904979	0.43751872
NGEF	-0.223065484	1.45E-05	4.63E-05
NGFRAP1	0.224742581	1.24E-05	4.03E-05
NGFR	0.038020788	0.465313865	0.540241874
NGF	0.011635665	0.823247541	0.859532388
NGLY1	-0.056035919	0.281689451	0.352501083
NGRN	0.082273364	0.113642176	0.160713294
NHEDC1	-0.049906334	0.337751448	0.411792188
NHEDC2	-0.400884459	9.36E-16	1.54E-14
NHEG1	0.086294862	0.096985959	0.13993006
NHEJ1	-0.070423157	0.175880156	0.234546119
NHLH1	0.257180337	5.12E-07	2.12E-06
NHLH2	0.191037954	0.000214431	0.000556308
NHLRC1	-0.189393083	0.00024358	0.000624771
NHLRC2	-0.048261264	0.353936404	0.428801849
NHLRC3	-0.115569911	0.026015149	0.04355608
NHLRC4	0.211812737	3.91E-05	0.000116353
NHP2L1	0.2063471	6.22E-05	0.000178512
NHP2	0.168070491	0.001156134	0.00260932
NHSL1	0.008030796	0.87747998	0.90355901
NHSL2	-0.102309891	0.048936134	0.076625455
NHS	0.303313667	2.47E-09	1.50E-08
NICN1	-0.129159649	0.012780321	0.022966764
NID1	0.079187022	0.127885235	0.177963792
NID2	0.045531187	0.381853222	0.458104293
NIF3L1	0.268074755	1.59E-07	7.14E-07
NINJ1	-0.076071668	0.143626395	0.196735771
NINJ2	0.043987957	0.398212592	0.474501541
NINL	0.15802059	0.002268515	0.004825258
NIN	0.248559951	1.25E-06	4.83E-06
NIP7	0.001584704	0.975731638	0.980901534

NIPA1	0.168864537	0.001094398	0.002480387
NIPA2	0.325585415	1.31E-10	9.68E-10
NIPAL1	0.021805625	0.675480378	0.734588137
NIPAL2	0.033426596	0.52097159	0.593243556
NIPAL3	0.104110002	0.04507198	0.071162147
NIPAL4	0.278950412	4.66E-08	2.30E-07
NIPBL	0.123198841	0.017595593	0.030569409
NIPSNAP1	-0.262445835	2.93E-07	1.26E-06
NIPSNAP3A	-0.277853209	5.29E-08	2.58E-07
NIPSNAP3B	-0.077317967	0.137160225	0.189075418
NISCH	0.014302754	0.783642851	0.826629932
NIT1	-0.29139266	1.08E-08	5.91E-08
NIT2	-0.176607951	0.000632855	0.001505041
NKAIN1	0.364497076	4.23E-13	4.61E-12
NKAIN2	0.001997673	0.969410142	0.975922622
NKAIN3	-0.051696008	0.320690729	0.394297395
NKAIN4	0.248918303	1.20E-06	4.67E-06
NKAPL	-0.117779447	0.023278107	0.039450996
NKAP	0.053334635	0.305570666	0.378110485
NKD1	-0.28747739	1.72E-08	9.13E-08
NKD2	0.153230993	0.00308668	0.006372976
NKG7	-0.015983806	0.758957138	0.805580697
NKIRAS1	-0.387184731	1.02E-14	1.42E-13
NKIRAS2	0.203009657	8.21E-05	0.000230257
NKPD1	0.185935383	0.000317324	0.000796775
NKRF	-0.008785374	0.866071577	0.894817859
NKTR	0.123750828	0.017091237	0.029791901
NKX1-2	0.001319662	0.979789575	0.984384887
NKX2-1	0.01551233	0.765857161	0.811391532
NKX2-2	0.062517188	0.229643386	0.295749922
NKX2-3	-0.020141272	0.698994322	0.755089734
NKX2-4	0.067480779	0.194677922	0.256201187
NKX2-5	0.248836212	1.21E-06	4.71E-06
NKX2-6	0.109585255	0.034858635	0.05651019
NKX2-8	0.282450914	3.11E-08	1.59E-07
NKX3-1	-0.056681329	0.27617549	0.346621166
NKX3-2	0.296389622	5.85E-09	3.36E-08
NKX6-1	0.11701835	0.024191393	0.040804413
NKX6-2	0.086716315	0.095359257	0.13780686
NKX6-3	0.090683098	0.081092625	0.119473988
NLE1	0.167732105	0.001183404	0.002664833
NLGN1	0.046184613	0.37505198	0.451194779
NLGN2	0.075425661	0.14706843	0.20087051
NLGN3	0.282341179	3.15E-08	1.61E-07
NLGN4X	-0.089816357	0.08405268	0.123306149
NLGN4Y	0.00555765	0.915036911	0.933546646
NLK	0.106575964	0.040197108	0.064244428
NLN	-0.034511572	0.507526494	0.580495249
NLRC3	0.102085959	0.049435654	0.077280062
NLRC4	0.235498351	4.53E-06	1.60E-05
NLRC5	0.151590555	0.003423368	0.006994188
NLRP10	0.087065745	0.094027112	0.136114528
NLRP11	-0.202826918	8.34E-05	0.000233525
NLRP12	0.131239433	0.011397391	0.020730286
NLRP13	0.09639243	0.063638364	0.096565121
NLRP14	-0.314106402	6.13E-10	4.09E-09
NLRP1	-0.012358269	0.812468369	0.850328072
NLRP2	0.227259808	9.85E-06	3.25E-05

NLRP3	0.205824665	6.50E-05	0.000185723
NLRP4	0.069895617	0.179147317	0.238249188
NLRP5	0.026251027	0.614253579	0.679728923
NLRP6	-0.392651091	3.99E-15	5.98E-14
NLRP7	0.160323314	0.001950381	0.004205596
NLRP8	0.05867337	0.259621296	0.328956017
NLRP9	-0.040122216	0.440996885	0.516705284
NLRX1	-0.104709727	0.043842896	0.069386573
NMBR	0.068863693	0.185667815	0.245658113
NMB	0.262978236	2.76E-07	1.20E-06
NMD3	-0.291851188	1.02E-08	5.62E-08
NME1-NME	0.067585091	0.193987359	0.255376738
NME1	0.294020566	7.82E-09	4.41E-08
NME2P1	0.18991326	0.000233984	0.000602018
NME2	0.237817331	3.62E-06	1.30E-05
NME3	0.058236188	0.263194409	0.332666359
NME4	0.076368818	0.142063946	0.194957494
NME5	0.161898537	0.001756847	0.00381845
NME6	0.34547756	7.71E-12	6.92E-11
NME7	0.109644422	0.034760031	0.056373277
NMI	0.242558679	2.28E-06	8.42E-06
NMNAT1	-0.256135279	5.72E-07	2.34E-06
NMNAT2	0.217788381	2.32E-05	7.16E-05
NMNAT3	-0.089821	0.084036594	0.123295238
NMRAL1	-0.061222533	0.239453563	0.30667913
NMT1	-0.047458416	0.362009508	0.437551202
NMT2	-0.321813319	2.19E-10	1.57E-09
NMUR1	0.143971022	0.005465967	0.010661151
NMUR2	0.070620276	0.174670778	0.233183089
NMU	0.246272309	1.57E-06	5.97E-06
NNAT	-0.005350486	0.918192657	0.936142995
NNMT	0.019491491	0.708254573	0.762642354
NNT	-0.275936924	6.58E-08	3.16E-07
NOB1	-0.038755626	0.456727237	0.531882856
NOBOX	0.109044323	0.035771198	0.057822646
NOC2L	0.034870188	0.503122738	0.576459098
NOC3L	-0.114294208	0.027718252	0.046074512
NOC4L	0.203739299	7.73E-05	0.000217908
NOD1	0.292367495	9.57E-09	5.31E-08
NOD2	0.259964192	3.82E-07	1.61E-06
NODAL	-0.05738539	0.270244579	0.340187824
NOG	0.160046211	0.001986378	0.004277196
NOL10	0.253617431	7.43E-07	2.99E-06
NOL11	0.147320958	0.004461294	0.008884237
NOL12	0.36924828	1.99E-13	2.28E-12
NOL3	-0.010996932	0.832803407	0.867236918
NOL4	-0.14574308	0.004911668	0.009695824
NOL6	0.145477757	0.0049913	0.00983645
NOL7	0.328857948	8.34E-11	6.37E-10
NOL8	0.15723505	0.002387435	0.005060914
NOL9	-0.017534555	0.736399192	0.786255856
NOLC1	0.153243878	0.003084159	0.006368431
NOM1	-0.09108552	0.079747235	0.117796724
NOMO1	0.088706521	0.087969452	0.128296297
NOMO2	0.119088615	0.021776816	0.037122929
NOMO3	0.090889869	0.080399068	0.118610059
NONO	0.278904368	4.69E-08	2.31E-07
NOP10	0.133404068	0.010100185	0.01862248

NOP14	-0.044194407	0.396000019	0.472317091
NOP16	0.161755773	0.001773634	0.003852413
NOP2	0.27264426	9.54E-08	4.45E-07
NOP56	0.433282872	2.07E-18	5.10E-17
NOP58	0.442383048	3.29E-19	9.02E-18
NOS1AP	-0.18061443	0.000472407	0.001148138
NOS1	-0.193175278	0.000181415	0.000477366
NOS2	-0.042941912	0.409536818	0.485758067
NOS3	0.033590853	0.518924435	0.591385652
NOSIP	0.195208241	0.000154482	0.000411883
NOSTRIN	-0.283826133	2.65E-08	1.37E-07
NOTCH1	0.292640845	9.25E-09	5.15E-08
NOTCH2NL	0.163455616	0.001582977	0.003470078
NOTCH2	0.107325834	0.038806083	0.062185873
NOTCH3	0.156143451	0.002562131	0.005398496
NOTCH4	-0.148630806	0.004116113	0.008256263
NOTO	0.114283514	0.027732924	0.046091208
NOTUM	-0.405229187	4.28E-16	7.42E-15
NOVA1	0.248724173	1.23E-06	4.76E-06
NOVA2	-0.005963055	0.908865237	0.928391183
NOV	0.14882081	0.004068098	0.008170647
NOX1	0.105544569	0.042178986	0.067035481
NOX3	0.105505813	0.042255035	0.067145633
NOX4	0.204630315	7.18E-05	0.000203638
NOX5	0.083695405	0.107513101	0.153111445
NOXA1	-0.013936158	0.789056081	0.831151866
NOXO1	0.030118664	0.563061585	0.633209872
NPAS1	0.307157337	1.51E-09	9.49E-09
NPAS2	0.283138515	2.87E-08	1.47E-07
NPAS3	0.036906982	0.478497441	0.552808282
NPAS4	0.066120997	0.203844806	0.266889867
NPAT	0.003177515	0.951361686	0.962278001
NPBWR1	0.136817496	0.008319558	0.015619851
NPB	0.01865306	0.720266933	0.773251897
NPC1L1	0.035340919	0.497372889	0.571236175
NPC1	0.300939161	3.33E-09	1.98E-08
NPC2	0.220563925	1.81E-05	5.71E-05
NPDC1	0.105094758	0.043068763	0.068264452
NPEPL1	0.332957318	4.70E-11	3.72E-10
NPEPPS	0.209962075	4.58E-05	0.000134685
NPFFR1	0.172804424	0.000830587	0.001927877
NPFFR2	0.121580251	0.019150153	0.033008585
NPFF	0.101085699	0.051719207	0.08040815
NPHP1	0.214089059	3.21E-05	9.68E-05
NPHP3	0.066671296	0.200098009	0.26258827
NPHP4	0.214138056	3.20E-05	9.64E-05
NPHS1	0.221819159	1.62E-05	5.14E-05
NPHS2	0.165303926	0.001397129	0.003103659
NPIPL3	0.141282678	0.006414606	0.012345839
NPIP	0.209214419	4.88E-05	0.000142704
NPLOC4	0.144399136	0.005327133	0.010418922
NPL	0.155796604	0.00262003	0.005506513
NPM1	0.398958187	1.32E-15	2.12E-14
NPM2	-0.025746538	0.621078902	0.685986244
NPM3	0.150930172	0.003568059	0.007260021
NPNT	0.098419538	0.05823862	0.089282785
NPPA	0.005742483	0.912222449	0.931295256
NPPB	0.170271672	0.000992399	0.002266747

NPPC	0.097729938	0.060031348	0.091692441
NPR1	-0.258622343	4.40E-07	1.84E-06
NPR2	-0.311194734	8.98E-10	5.85E-09
NPR3	-0.163244475	0.001605591	0.003513465
NPRL2	0.045361999	0.383626429	0.459899248
NPRL3	-0.00954829	0.854565296	0.88559352
NPSR1	0.143534012	0.005611033	0.010918441
NPTN	0.044904219	0.388449408	0.464814049
NPTX1	0.32766996	9.83E-11	7.41E-10
NPTX2	0.267037736	1.78E-07	7.95E-07
NPTXR	0.155403065	0.002687164	0.005628015
NPVF	-0.080117203	0.123454107	0.172702248
NPW	0.149811285	0.003825902	0.007732538
NPY1R	-0.1752589	0.000697366	0.001643743
NPY2R	-0.002871148	0.956046279	0.965545675
NPY5R	0.045390122	0.383331336	0.459600799
NPY6R	0.003672351	0.943798864	0.956608235
NPY	0.068684973	0.186814639	0.246995038
NQO1	0.178364065	0.000557151	0.001337458
NQO2	-0.176069669	0.000657902	0.001559581
NR0B1	0.360102706	8.42E-13	8.71E-12
NR0B2	-0.048433517	0.352219178	0.426923246
NR1D1	-0.134838146	0.00931458	0.017316634
NR1D2	-0.253567405	7.47E-07	3.00E-06
NR1H2	-0.022140148	0.670791171	0.730997259
NR1H3	-0.190570077	0.000222372	0.000575186
NR1H4	-0.175761363	0.000672658	0.001590965
NR1I2	-0.368332038	2.30E-13	2.63E-12
NR1I3	-0.437292083	9.26E-19	2.41E-17
NR2C1	0.216577823	2.58E-05	7.90E-05
NR2C2AP	0.372857751	1.11E-13	1.33E-12
NR2C2	0.052653594	0.311796669	0.384620015
NR2E1	0.148361916	0.004184938	0.008381656
NR2E3	-0.002711888	0.958482088	0.967417173
NR2F1	-0.088443324	0.088919492	0.129539606
NR2F2	0.031831789	0.541060912	0.612652921
NR2F6	-0.002484038	0.961967627	0.97005057
NR3C1	-0.28003786	4.11E-08	2.05E-07
NR3C2	-0.358986217	1.00E-12	1.03E-11
NR4A1	-0.094316128	0.069589487	0.104473381
NR4A2	-0.10012221	0.054001269	0.083532583
NR4A3	-0.077044665	0.1385587	0.190814458
NR5A1	0.155920739	0.002599173	0.005467869
NR5A2	-0.048796938	0.348613429	0.423196438
NR6A1	0.018462267	0.723010222	0.775270812
NRADDP	0.154515245	0.002844403	0.005918758
NRAP	0.111757215	0.03139122	0.051471266
NRARP	0.20431945	7.37E-05	0.000208554
NRAS	0.231969895	6.34E-06	2.18E-05
NRBF2	-0.15193287	0.003350478	0.006856066
NRBP1	0.113735723	0.028493469	0.047233058
NRBP2	0.110367032	0.033574791	0.05466021
NRCAM	0.284795341	2.36E-08	1.23E-07
NRD1	0.001848959	0.971686378	0.977772301
NRF1	0.250843332	9.89E-07	3.90E-06
NRG1	-0.087039569	0.094126388	0.13623844
NRG2	0.110872991	0.032765549	0.053508917
NRG3	0.296608739	5.70E-09	3.27E-08

NRG4	0.09894032	0.056914316	0.087501699
NRGN	0.343876581	9.76E-12	8.61E-11
NRIP1	-0.27684992	5.93E-08	2.87E-07
NRIP2	0.092745301	0.074387958	0.110717736
NRIP3	0.304433495	2.14E-09	1.31E-08
NRK	0.164342846	0.001491107	0.003291505
NRL	-0.117570636	0.023525655	0.03981972
NRM	0.52811533	4.92E-28	5.19E-26
NRN1L	-0.07680008	0.139819487	0.192314542
NRN1	-0.206521537	6.13E-05	0.000176146
NRP1	-0.023765154	0.648196578	0.711127938
NRP2	0.03502904	0.501178502	0.574679298
NRSN1	0.135016795	0.009220619	0.017153216
NRSN2	0.467197592	1.63E-21	6.18E-20
NRTN	0.056782851	0.275314869	0.345631283
NRXN1	0.08723899	0.093372178	0.135264728
NRXN2	-0.089922738	0.083684749	0.122884024
NRXN3	0.102745161	0.047977258	0.07531938
NSA2	0.047165005	0.364988369	0.440631335
NSD1	0.305762356	1.81E-09	1.12E-08
NSDHL	-0.155471946	0.002675301	0.005606117
NSFL1C	0.042840711	0.410642431	0.486837569
NSF	0.183144068	0.000391519	0.000966044
NSL1	0.012882786	0.804666074	0.843846793
NSMAF	0.368300963	2.31E-13	2.64E-12
NSMCE1	-0.078804325	0.129743673	0.180235231
NSMCE2	0.237721674	3.66E-06	1.31E-05
NSMCE4A	-0.197230957	0.000131441	0.000354724
NSUN2	0.165173288	0.001409576	0.003129912
NSUN3	-0.102317349	0.04891957	0.07660554
NSUN4	0.015673145	0.763501533	0.809489774
NSUN5P1	0.107607107	0.038294926	0.061446518
NSUN5P2	0.091396176	0.078721048	0.116436314
NSUN5	0.181805791	0.00043255	0.001058113
NSUN6	-0.32107758	2.42E-10	1.72E-09
NSUN7	0.271168913	1.13E-07	5.19E-07
NT5C1A	0.080016216	0.123929328	0.173306196
NT5C1B	-0.067305513	0.195842266	0.257614369
NT5C2	0.199675504	0.000107899	0.000296083
NT5C3L	0.051458399	0.322922963	0.396810653
NT5C3	0.410690447	1.58E-16	2.94E-15
NT5C	0.236200654	4.24E-06	1.50E-05
NT5DC1	-0.360170881	8.33E-13	8.63E-12
NT5DC2	0.330027138	7.08E-11	5.49E-10
NT5DC3	0.133796623	0.009879501	0.018262913
NT5E	-0.035836956	0.491351846	0.565396608
NT5M	-0.118483006	0.022460537	0.038192115
NTAN1	0.053051095	0.30815271	0.380808776
NTF3	-0.027985442	0.591043861	0.658618178
NTF4	0.141853874	0.006201516	0.011973891
NTHL1	-0.19906558	0.00011337	0.000309732
NTM	0.287423458	1.73E-08	9.19E-08
NTN1	-0.099319336	0.05596628	0.08624383
NTN3	-0.166563811	0.00128217	0.002865536
NTN4	-0.144521771	0.005287952	0.010349403
NTN5	-0.196095335	0.000143946	0.000385858
NTNG1	0.135853248	0.0087918	0.016416743
NTNG2	0.372103281	1.26E-13	1.49E-12

NTRK1	0.281705904	3.39E-08	1.72E-07
NTRK2	0.168004463	0.001161409	0.002620042
NTRK3	0.084172295	0.105517088	0.150678189
NTSR1	0.116931138	0.024297993	0.040966877
NTSR2	0.145499957	0.004984593	0.009826675
NTS	0.230113419	7.55E-06	2.55E-05
NUAK1	0.272993332	9.18E-08	4.29E-07
NUAK2	0.041468317	0.425809185	0.502218376
NUB1	-0.116518809	0.024807497	0.04174818
NUBP1	-0.022148258	0.670677646	0.730940886
NUBP2	-0.065607354	0.207387662	0.270798781
NUBPL	-0.4924021	4.71E-24	2.60E-22
NUCB1	-0.140742655	0.006622076	0.012708313
NUCB2	0.119431476	0.021397788	0.036528426
NUCKS1	-0.05831927	0.262512787	0.332091678
NUDCD1	0.263617045	2.58E-07	1.12E-06
NUDCD2	-0.174256599	0.000749179	0.001754455
NUDCD3	-0.222369304	1.54E-05	4.91E-05
NUDC	0.137922376	0.007806335	0.01475361
NUDT10	0.038823161	0.45594256	0.531186414
NUDT11	0.30929954	1.15E-09	7.34E-09
NUDT12	-0.10155312	0.05064139	0.078923396
NUDT13	-0.265710298	2.05E-07	9.09E-07
NUDT14	0.248675732	1.23E-06	4.78E-06
NUDT15	0.222500368	1.52E-05	4.86E-05
NUDT16L1	0.016366736	0.753366984	0.800713671
NUDT16P1	-0.238388215	3.43E-06	1.23E-05
NUDT16	-0.362908129	5.44E-13	5.81E-12
NUDT17	0.220138815	1.88E-05	5.91E-05
NUDT18	-0.021889757	0.674299852	0.733624687
NUDT19	0.094668635	0.068548204	0.103182268
NUDT1	0.453071963	3.53E-20	1.09E-18
NUDT21	0.030882754	0.553195868	0.624300011
NUDT22	0.134274474	0.009616646	0.01782
NUDT2	0.030800856	0.554249267	0.625240877
NUDT3	0.202108584	8.84E-05	0.000246678
NUDT4	-0.143227658	0.005714781	0.011099723
NUDT5	-0.006239177	0.904664812	0.925178839
NUDT6	-0.526385398	7.87E-28	8.08E-26
NUDT7	-0.390981275	5.33E-15	7.81E-14
NUDT8	-0.208290478	5.28E-05	0.000153364
NUDT9P1	0.013088374	0.801613147	0.841204969
NUDT9	-0.098070091	0.059141448	0.09046961
NUF2	0.593013638	1.32E-36	1.05E-33
NUFIP1	-0.034972995	0.501863998	0.575332867
NUFIP2	0.165269285	0.00140042	0.003110275
NUMA1	-0.070873651	0.17312536	0.231368043
NUMBL	0.295326763	6.67E-09	3.80E-08
NUMB	-0.173263588	0.000804003	0.001871843
NUP107	0.410004279	1.79E-16	3.31E-15
NUP133	0.082253614	0.113729187	0.160802901
NUP153	-0.003931974	0.939832891	0.953715915
NUP155	0.258374625	4.52E-07	1.88E-06
NUP160	0.068640232	0.187102548	0.247326453
NUP188	0.318505124	3.42E-10	2.37E-09
NUP205	0.337452639	2.48E-11	2.05E-10
NUP210L	-0.129406297	0.012608931	0.022699723
NUP210	0.446065051	1.54E-19	4.41E-18

NUP214	0.121580056	0.019150348	0.033008585
NUP35	0.189309809	0.000245149	0.000628554
NUP37	0.378668073	4.28E-14	5.44E-13
NUP43	0.42936211	4.49E-18	1.05E-16
NUP50	0.238616938	3.35E-06	1.21E-05
NUP54	-0.037900826	0.46672406	0.541593472
NUP62CL	0.188785272	0.000255258	0.000651954
NUP62	0.429483881	4.39E-18	1.03E-16
NUP85	0.352923289	2.53E-12	2.45E-11
NUP88	-0.114433789	0.027527357	0.045799231
NUP93	0.317312633	4.01E-10	2.75E-09
NUP98	0.088898989	0.08727989	0.127467925
NUPL1	0.168944221	0.001088373	0.002467013
NUPL2	0.169660385	0.001035582	0.002355928
NUPR1	0.121010547	0.019725087	0.033896591
NUS1	0.004993457	0.923634301	0.940295495
NUSAP1	0.579247015	1.26E-34	5.83E-32
NUTF2	0.222621535	1.50E-05	4.81E-05
NVL	0.314697503	5.67E-10	3.79E-09
NWD1	0.009875084	0.849645853	0.881343137
NXF1	0.05169646	0.320686492	0.394297395
NXF2B	0.057346942	0.270566194	0.340506614
NXF2	0.020100959	0.699567556	0.755504139
NXF3	0.098913409	0.056982133	0.087596202
NXF4	0.152960353	0.003140069	0.006475315
NXF5	-0.154311634	0.002881623	0.005986828
NXNL1	-0.025785804	0.620546488	0.685436191
NXNL2	0.191301612	0.000210074	0.000546427
NXN	0.099735763	0.054939809	0.084819503
NXPH1	0.053705546	0.30221462	0.374737858
NXPH2	0.284162179	2.55E-08	1.32E-07
NXPH3	0.015718612	0.762835918	0.80887892
NXPH4	0.405343157	4.19E-16	7.28E-15
NXT1	0.339221229	1.92E-11	1.62E-10
NXT2	0.009598579	0.853807878	0.884983382
NYNRIN	0.098621171	0.05772289	0.088567115
NYX	0.137337287	0.008074481	0.015212769
OAF	-0.434441617	1.64E-18	4.11E-17
OAS1	0.062840265	0.227240325	0.292958115
OAS2	0.0490733	0.345887136	0.420346968
OAS3	0.24628744	1.57E-06	5.97E-06
OASL	-0.166605039	0.001278559	0.00285875
OAT	-0.098635271	0.057686968	0.088532453
OAZ1	0.266675452	1.85E-07	8.25E-07
OAZ2	-0.17247298	0.000850279	0.00197014
OAZ3	0.061069322	0.240633699	0.30800999
OBFC1	-0.100180143	0.053861727	0.083355342
OBFC2A	0.271992111	1.03E-07	4.75E-07
OBFC2B	0.433041034	2.17E-18	5.33E-17
OBP2A	0.182640954	0.0004065	0.000999665
OBP2B	0.134447227	0.009523158	0.017663192
OBSCN	0.428154737	5.69E-18	1.32E-16
OBSL1	0.117646665	0.023435261	0.039683575
OC90	0.06976789	0.179945077	0.239150489
OCA2	0.321589756	2.26E-10	1.61E-09
OCEL1	-0.367675239	2.56E-13	2.89E-12
OCIAD1	-0.155944915	0.002595128	0.005459937
OCIAD2	-0.138912966	0.007370419	0.014004148

OCLM	-0.023979272	0.645242768	0.708545986
OCLN	0.112454525	0.030342428	0.049965436
OCM2	0.026311641	0.613435733	0.679012484
OCM	-0.02508945	0.630017643	0.694549913
OCRL	0.134247003	0.009631587	0.017846026
ODAM	-0.124188193	0.016700628	0.029172285
ODC1	0.312440436	7.63E-10	5.01E-09
ODF1	0.119047256	0.021822929	0.037190453
ODF2L	0.278859199	4.71E-08	2.32E-07
ODF2	0.240604385	2.76E-06	1.01E-05
ODF3B	-0.182918311	0.000398176	0.000980649
ODF3L1	-0.034905501	0.502690187	0.576047967
ODF3L2	-0.058232927	0.26322119	0.332670973
ODF3	0.021892885	0.674255974	0.733617013
ODF4	-0.025572894	0.623435742	0.688398562
ODZ1	0.074727515	0.150858511	0.205399347
ODZ2	-0.254631836	6.69E-07	2.71E-06
ODZ3	0.079292981	0.127374347	0.177364348
ODZ4	0.274729475	7.55E-08	3.58E-07
OFD1	0.39626352	2.12E-15	3.30E-14
OGDHL	-0.303020825	2.56E-09	1.55E-08
OGDH	-0.166034034	0.001329407	0.002962798
OGFOD1	-0.011087171	0.831451846	0.86619169
OGFOD2	0.024700091	0.635339997	0.699643106
OGFRL1	0.414849124	7.27E-17	1.44E-15
OGFR	0.075018641	0.149269136	0.203527461
OGG1	0.080358846	0.122322753	0.171324092
OGN	-0.032205065	0.536324526	0.608119363
OGT	0.161616556	0.001790145	0.003887428
OIP5	0.497062623	1.51E-24	8.99E-23
OIT3	-0.272508181	9.69E-08	4.51E-07
OLA1	0.544105982	5.64E-30	8.16E-28
OLAH	0.01813319	0.727750134	0.77918193
OLFM1	0.086963786	0.094414269	0.136615418
OLFM2	-0.127957858	0.013645124	0.024360441
OLFM3	0.088644299	0.08819331	0.128603947
OLFM4	0.363085101	5.29E-13	5.67E-12
OLFML1	0.067230472	0.196342333	0.258127982
OLFML2A	0.15348339	0.003037635	0.006279434
OLFML2B	0.352379623	2.75E-12	2.64E-11
OLFML3	0.254001445	7.14E-07	2.88E-06
OLIG1	0.175356308	0.000692511	0.001633074
OLIG2	0.084144311	0.105633398	0.150801043
OLIG3	0.106530517	0.040282751	0.06437614
OLR1	0.205019711	6.95E-05	0.000197477
OMA1	-0.259774243	3.89E-07	1.64E-06
OMD	-0.00017589	0.997305995	0.997756744
OMG	0.2180204	2.27E-05	7.03E-05
OMP	0.127108733	0.014286794	0.025356037
ONECUT1	-0.004459075	0.931785632	0.947141862
ONECUT2	-0.299453784	4.00E-09	2.35E-08
ONECUT3	-0.113828184	0.028363857	0.047041688
OOEP	0.014087817	0.786815414	0.829156506
OPA1	-0.17495074	0.000712931	0.001677456
OPA3	0.18862853	0.000258353	0.000659268
OPALIN	0.069919503	0.178998424	0.238067067
OPCML	0.078584292	0.130821602	0.181504837
OPHN1	0.166412576	0.001295495	0.002893044

OPLAH	-0.150148888	0.003746381	0.007590298
OPN1LW	0.08610684	0.097718767	0.140834355
OPN1MW	0.113411833	0.028951517	0.047908641
OPN1SW	0.201276868	9.47E-05	0.000262624
OPN3	0.124289221	0.016611518	0.029036997
OPN4	-0.128841089	0.013004718	0.023338436
OPN5	-0.043624209	0.402128966	0.478338504
OPRD1	0.092792595	0.074239653	0.110521773
OPRK1	0.047457409	0.3620197	0.437551202
OPRL1	0.274572984	7.68E-08	3.64E-07
OPRM1	0.054462919	0.295437941	0.367397919
OPTC	-0.004149163	0.93651628	0.951076108
OPTN	0.200324484	0.00010235	0.000282061
OR10A2	0.113472393	0.028865396	0.047789946
OR10A3	0.069695823	0.180396348	0.239638331
OR10A4	0.025154064	0.629136245	0.693847003
OR10A5	-0.074766819	0.150643187	0.205162262
OR10A6	0.083428329	0.108643887	0.15456713
OR10A7	0.200360387	0.000102051	0.000281314
OR10AD1	0.110101083	0.03400694	0.055291459
OR10C1	0.084451291	0.104363	0.149233396
OR10G2	0.125911612	0.015236425	0.026869086
OR10G3	0.022854818	0.660816103	0.722347238
OR10G4	0.029910715	0.565761131	0.635528235
OR10H1	0.069055039	0.184445726	0.24423581
OR10H2	-0.032092787	0.537747004	0.609558795
OR10H3	-0.002053347	0.968558056	0.975166199
OR10H4	-0.018267491	0.725814433	0.777443687
OR10H5	0.070177209	0.177397814	0.236332704
OR10J3	-0.029431944	0.571999819	0.6414873
OR10J5	-0.127167324	0.014241681	0.025291743
OR10P1	0.001756385	0.973103431	0.978555679
OR10Q1	0.158391936	0.002214202	0.00472183
OR10V1	-0.091986042	0.076802003	0.113885337
OR10W1	0.069640164	0.180745449	0.239990058
OR11A1	0.2409255	2.68E-06	9.80E-06
OR11G2	0.018558232	0.721629958	0.774252047
OR11H12	0.030766192	0.554695421	0.625601256
OR11H1	-0.024972537	0.63161379	0.696149633
OR11H4	0.044648909	0.391155151	0.467574278
OR11H6	0.091105102	0.079682232	0.117726893
OR11L1	0.116861267	0.02438369	0.041090499
OR12D2	0.268638842	1.49E-07	6.75E-07
OR12D3	0.09819997	0.058804553	0.090032609
OR13A1	0.419273588	3.16E-17	6.57E-16
OR13C2	-0.046551188	0.371269307	0.447074902
OR13C3	-0.063577629	0.221822788	0.287175865
OR13C4	-0.000670346	0.989732943	0.99202353
OR13C5	-0.04649666	0.371830481	0.447670686
OR13D1	0.010111672	0.846087967	0.878270346
OR13F1	-0.019544828	0.707492809	0.761956522
OR13G1	-0.039790777	0.444783303	0.520472842
OR13H1	0.048511946	0.351439048	0.426133215
OR13J1	-0.070612268	0.174719793	0.233209993
OR14I1	0.085303511	0.100899475	0.144915243
OR14J1	0.193960336	0.000170531	0.000450813
OR1B1	-0.032824509	0.528510723	0.600489941
OR1C1	0.111625322	0.03159304	0.051768099

OR1D4	0.054378132	0.296191503	0.368197124
OR1E1	-0.024127677	0.643198745	0.706695643
OR1E2	-0.050064623	0.336219498	0.410175434
OR1F1	0.095483931	0.066188684	0.099955349
OR1F2P	0.085801156	0.098919557	0.1423487
OR1G1	-0.032412596	0.533700253	0.605591869
OR1I1	0.025393608	0.625873194	0.690783699
OR1J1	0.163317958	0.001597687	0.003499998
OR1J2	0.158137781	0.002251245	0.004794153
OR1J4	0.075998377	0.144013783	0.197171506
OR1K1	-0.009096335	0.861378101	0.891109824
OR1L3	0.059813783	0.250458568	0.31866366
OR1L4	0.065471956	0.208328938	0.271903113
OR1L6	0.04089915	0.43219352	0.508303873
OR1L8	-0.021138457	0.684869832	0.742771887
OR1N1	0.054302612	0.296863777	0.368894721
OR1N2	0.007291999	0.888674435	0.912257025
OR1Q1	0.069479441	0.181756336	0.241113808
OR1S2	0.077599534	0.135730824	0.187376445
OR2A12	-0.02391287	0.646158202	0.709360429
OR2A14	0.01666434	0.7490313	0.797211324
OR2A1	0.040968179	0.431416293	0.507624327
OR2A25	0.142321012	0.006031988	0.011663523
OR2A2	0.063706934	0.220882341	0.286113004
OR2A4	0.08709378	0.093920881	0.135970627
OR2A5	0.029403591	0.572370299	0.641794399
OR2A7	0.18506736	0.00033886	0.000846278
OR2A9P	0.196162083	0.000143182	0.000383912
OR2AE1	0.120476255	0.020277866	0.034780512
OR2AG1	0.02925889	0.574262812	0.643590445
OR2AG2	0.099160861	0.056361069	0.08676495
OR2AK2	0.095561772	0.065966936	0.099659115
OR2AT4	0.050557605	0.331476924	0.405486019
OR2B11	0.106789426	0.039796908	0.063640552
OR2B2	0.058279862	0.26283595	0.332394959
OR2B3	0.047421853	0.36237988	0.437853709
OR2B6	0.093935212	0.070729194	0.105944887
OR2C1	-0.111175436	0.032289801	0.052818575
OR2C3	0.108142276	0.037338097	0.060070222
OR2D2	0.134757897	0.009357063	0.017387503
OR2F1	0.033252387	0.523147295	0.59521076
OR2F2	0.039475119	0.448406553	0.524215443
OR2G6	0.020805857	0.689569007	0.746567782
OR2H1	0.274853755	7.44E-08	3.54E-07
OR2H2	0.314243337	6.02E-10	4.02E-09
OR2J2	-0.007190258	0.89021783	0.913464827
OR2J3	0.094214376	0.069892452	0.104877584
OR2K2	0.137066525	0.008201328	0.01542073
OR2L13	-0.069496223	0.18165059	0.24102085
OR2L1P	0.037932801	0.466347953	0.541251612
OR2L2	0.005988425	0.908479201	0.928139623
OR2L3	0.061984021	0.233648377	0.30022852
OR2S2	0.008640348	0.868262127	0.896430249
OR2T10	0.092751743	0.07436774	0.110704185
OR2T2	-0.019940983	0.701844029	0.757634069
OR2T33	-0.018930263	0.71628761	0.769639274
OR2T34	-0.033651661	0.518167634	0.590692126
OR2T3	0.014633501	0.778767898	0.822227678

OR2T4	-0.000874939	0.986599645	0.989879035
OR2T5	-0.127370162	0.014086469	0.02504289
OR2T8	-0.016713662	0.748313501	0.796594335
OR2V2	0.03907851	0.452982555	0.528510598
OR2W3	0.049341619	0.343253169	0.417605473
OR2W5	0.058268328	0.262930583	0.332461353
OR2Z1	0.061143657	0.240060611	0.307318307
OR3A1	0.056230944	0.280015502	0.350736801
OR3A2	-0.01947361	0.708510013	0.762804608
OR3A3	0.01941587	0.709335095	0.763610384
OR3A4	0.073622987	0.157005534	0.212577426
OR4A16	-0.005862685	0.910392717	0.929617822
OR4A47	-0.070045731	0.178213093	0.23727603
OR4C6	0.18911166	0.000248923	0.000637574
OR4D10	0.023466412	0.652327027	0.714990044
OR4D11	-0.009225286	0.859433221	0.889666335
OR4D1	-0.077492753	0.136271555	0.188018694
OR4D6	-0.083445923	0.108569107	0.154482804
OR4D9	0.007973362	0.87834939	0.904284973
OR4E2	0.027712815	0.594665429	0.66202429
OR4F17	-0.071127857	0.171585122	0.229633131
OR4F21	-0.001697452	0.97400559	0.979265171
OR4F29	-0.060839759	0.242409578	0.309887296
OR4F4	-0.007907502	0.879346539	0.904779556
OR4F5	-0.045519088	0.381979858	0.458201031
OR4K14	0.074918151	0.149816309	0.204203648
OR4K1	0.202410579	8.63E-05	0.000241166
OR4K2	0.040885792	0.432344013	0.508390829
OR4M1	0.04542402	0.38297582	0.459229823
OR4M2	0.062629182	0.22880834	0.294731654
OR4N2	0.060622455	0.244099064	0.311826967
OR4N3P	0.032970665	0.526675527	0.598677956
OR4N4	0.027032435	0.603747291	0.670112738
OR4Q3	0.004504752	0.931088597	0.946529929
OR51B2	0.011990152	0.817955341	0.855127325
OR51B4	-0.01642452	0.752524541	0.800117089
OR51B5	-0.004450764	0.931912464	0.947222454
OR51B6	-0.051547444	0.322085245	0.395889823
OR51E1	0.149989058	0.00378384	0.007651407
OR51E2	0.086270136	0.097082073	0.140028169
OR51G2	0.084423956	0.104475628	0.149383726
OR51I1	-0.040289819	0.439089181	0.51477601
OR51I2	-0.020237884	0.697621225	0.753769934
OR51M1	0.023343236	0.654033189	0.716408244
OR51Q1	0.090811893	0.080660052	0.118933429
OR52A4	0.033743684	0.517023407	0.589559237
OR52B2	0.06865584	0.187002074	0.247226446
OR52B4	0.027405235	0.598763384	0.665693629
OR52B6	0.042277566	0.416826877	0.49276199
OR52D1	0.056218454	0.280122505	0.350789585
OR52E2	0.057050357	0.27305592	0.343227966
OR52E4	-0.002808774	0.957000224	0.966125579
OR52E6	0.05111393	0.326176969	0.400301705
OR52E8	0.011529948	0.824827379	0.860956253
OR52H1	-0.049873384	0.338070901	0.412131226
OR52I1	-0.006752168	0.896868236	0.918774567
OR52I2	-0.006864722	0.895158909	0.917353693
OR52K1	0.021301927	0.68256467	0.74070609

OR52K2	0.057923964	0.26576684	0.335398897
OR52L1	0.073290791	0.1588908	0.21483497
OR52M1	-0.00561441	0.914172505	0.932855845
OR52N1	0.046683851	0.369906195	0.445732592
OR52N2	0.148370655	0.004182685	0.008377986
OR52N4	-0.001152312	0.982352066	0.986412191
OR52N5	0.049036472	0.34624966	0.420660186
OR52R1	-0.083956836	0.10641523	0.151797523
OR52W1	0.045886244	0.378148282	0.454288734
OR56A1	0.023936346	0.645834491	0.709044128
OR56A3	0.09563973	0.065745464	0.099368874
OR56A4	0.057471575	0.269524587	0.339410808
OR56A5	0.071211552	0.171080252	0.229065178
OR56B1	0.113648302	0.028616484	0.047421195
OR56B4	-0.003005613	0.953990007	0.964163576
OR5A1	0.123820332	0.017028635	0.02970097
OR5AK2	-0.019009725	0.71514832	0.76866387
OR5AN1	0.113286335	0.029130687	0.048161125
OR5AP2	-0.077665918	0.135395494	0.18693943
OR5AU1	0.039998221	0.442411262	0.518088241
OR5B12	0.083755239	0.107261045	0.152807104
OR5B21	-0.055470442	0.286581258	0.357948076
OR5B2	-0.009467605	0.85578077	0.88671475
OR5C1	0.078572779	0.130878191	0.181570705
OR5E1P	0.007902121	0.879428014	0.904816655
OR5H2	-0.074820012	0.150352147	0.204835907
OR5H6	-0.078294773	0.132250463	0.183168362
OR5K1	0.04755183	0.361064308	0.436634888
OR5K2	0.038300923	0.462029971	0.536969449
OR5K3	-0.005098415	0.922034198	0.939050421
OR5K4	-0.025187988	0.628673692	0.693375259
OR5M10	0.043364936	0.404934477	0.481129878
OR5M11	0.122044917	0.018692079	0.03229973
OR5M8	-0.027722692	0.594534045	0.66202429
OR5P3	0.017714225	0.733799773	0.784184051
OR5V1	0.16773526	0.001183147	0.002664556
OR6A2	0.058691022	0.259477728	0.328836851
OR6B1	0.109043431	0.035772719	0.057822646
OR6B2	0.089499988	0.08515459	0.12472061
OR6B3	0.093146507	0.073137483	0.109117422
OR6C2	0.019815634	0.703629628	0.759026935
OR6C3	0.039001772	0.45387096	0.52941569
OR6C68	-0.000459146	0.992967593	0.994515127
OR6C70	0.03847624	0.459981382	0.534859575
OR6F1	-0.033526598	0.519724762	0.592106036
OR6K3	0.100988397	0.051945961	0.080704003
OR6M1	0.065264384	0.20977794	0.273543403
OR6S1	-0.041055066	0.430439147	0.506809024
OR6T1	0.046549104	0.371290746	0.447074902
OR6W1P	0.022386991	0.667339197	0.727760646
OR7A5	0.098270084	0.058623344	0.089782769
OR7C1	0.169534428	0.001044692	0.002375568
OR7D2	0.042530067	0.414047179	0.490046874
OR7E156P	0.088012045	0.090494025	0.131544836
OR7E24	0.086227492	0.097248024	0.14022692
OR7E37P	0.018816364	0.717921733	0.771145563
OR7E5P	0.100636536	0.052772866	0.081853665
OR7E91P	0.097716526	0.060066662	0.091739347

OR8A1	0.133552736	0.010016098	0.018482837
OR8B12	0.075717234	0.145507182	0.1989427
OR8B2	0.013719949	0.792253449	0.833902531
OR8B3	-0.001348266	0.979351603	0.984044113
OR8B8	0.082132068	0.114265841	0.161447098
OR8D1	0.12488328	0.016095904	0.02821244
OR8D2	0.095505583	0.066126941	0.09988079
OR8G2	0.001038094	0.984101108	0.98786971
OR8G5	0.042752704	0.411605328	0.487833978
OR8I2	0.027693235	0.594925911	0.662240264
OR8K3	0.028293647	0.586961903	0.654875394
OR8S1	0.042984089	0.409076565	0.485327735
OR9A2	-0.078255202	0.132446687	0.183427382
OR9A4	0.15449697	0.002847726	0.005925054
OR9I1	0.154619247	0.00282556	0.005881812
OR9Q1	0.020998461	0.686846324	0.744307684
ORAI1	0.034146927	0.512024864	0.584693302
ORAI2	0.363591319	4.88E-13	5.26E-12
ORAI3	-0.330506179	6.63E-11	5.16E-10
ORAOV1	0.237721951	3.66E-06	1.31E-05
ORC1L	0.533912186	1.00E-28	1.17E-26
ORC2L	0.147742047	0.004347578	0.008686435
ORC3L	0.101384633	0.05102772	0.079457109
ORC4L	0.228881732	8.47E-06	2.84E-05
ORC5L	-0.082243411	0.11377416	0.160843657
ORC6L	0.584296533	2.42E-35	1.38E-32
ORM1	-0.076829516	0.139667284	0.192131724
ORM2	-0.19228107	0.000194602	0.000508908
ORMDL1	0.164975391	0.001428625	0.003167267
ORMDL2	0.239485267	3.08E-06	1.12E-05
ORMDL3	-0.379135136	3.96E-14	5.06E-13
OS9	-0.32903911	8.13E-11	6.22E-10
OSBP2	0.371164104	1.46E-13	1.72E-12
OSBPL10	0.348927769	4.62E-12	4.28E-11
OSBPL11	-0.145301257	0.005044915	0.009927379
OSBPL1A	0.037263744	0.474252634	0.549060604
OSBPL2	0.130273654	0.01202221	0.021757338
OSBPL3	0.45141206	5.02E-20	1.52E-18
OSBPL5	0.204637341	7.18E-05	0.000203548
OSBPL6	0.155805525	0.002618526	0.005503933
OSBPL7	0.397267476	1.78E-15	2.80E-14
OSBPL8	0.054927375	0.291332681	0.363085997
OSBPL9	0.289202199	1.40E-08	7.58E-08
OSBP	-0.276821283	5.95E-08	2.88E-07
OSCAR	0.278399241	4.97E-08	2.44E-07
OSCP1	-0.089957641	0.083564318	0.122743371
OSGEPL1	-0.12614429	0.015047631	0.026566723
OSGEP	0.032668612	0.530471789	0.60240888
OSGIN1	-0.282271258	3.18E-08	1.62E-07
OSGIN2	0.328310659	9.00E-11	6.83E-10
OSMR	0.173783841	0.000774834	0.001809005
OSM	0.25930692	4.09E-07	1.72E-06
OSR1	-0.002808236	0.957008442	0.966125579
OSR2	0.030661925	0.556038463	0.626726917
OST4	0.071270783	0.170723624	0.22871917
OSTBETA	0.163319648	0.001597506	0.003499998
OSTCL	-0.026668084	0.608636105	0.674524282
OSTC	0.028880337	0.579227735	0.648235812

OSTF1	0.137065333	0.00820189	0.01542073
OSTM1	0.129797682	0.012341124	0.02227597
OSTN	0.058359941	0.262179558	0.331775466
OSTalpha	-0.216280501	2.65E-05	8.10E-05
OTC	-0.434532581	1.61E-18	4.05E-17
OTOA	0.027137949	0.602334808	0.668954958
OTOF	0.284443407	2.46E-08	1.28E-07
OTOL1	-0.008703196	0.867312711	0.895728555
OTOP1	0.141690967	0.006261634	0.01207826
OTOP2	0.043441369	0.40410621	0.480239972
OTOP3	0.205448742	6.71E-05	0.000191269
OTOR	-0.041379581	0.426800913	0.503090219
OTOS	0.061167063	0.23988036	0.307166508
OTP	0.309679912	1.09E-09	7.01E-09
OTUB1	0.256621019	5.43E-07	2.23E-06
OTUB2	0.006006764	0.908200153	0.92794971
OTUD1	0.167500525	0.001202407	0.002703041
OTUD3	0.289249399	1.39E-08	7.54E-08
OTUD4	-0.1366146	0.008417006	0.015776046
OTUD5	-0.13887476	0.007386821	0.014026021
OTUD6A	0.016913575	0.745406351	0.794178066
OTUD6B	0.109202633	0.035502048	0.057441063
OTUD7A	-0.008031423	0.877470503	0.90355901
OTUD7B	-0.137823685	0.007851	0.01482913
OTX1	0.289626995	1.33E-08	7.23E-08
OTX2	0.148978571	0.004028616	0.008101621
OVCA2	0.088510528	0.088676132	0.129232326
OVCH1	-0.063165021	0.224842821	0.290449921
OVCH2	0.049746628	0.339301631	0.413454467
OVGP1	-0.004961496	0.924121603	0.940743514
OVOL1	0.234081786	5.19E-06	1.81E-05
OVOL2	0.475389379	2.57E-22	1.11E-20
OXA1L	0.009567797	0.854271476	0.885342448
OXCT1	0.217575583	2.36E-05	7.28E-05
OXCT2	0.199083909	0.000113202	0.0003094
OXER1	-0.035308101	0.497772611	0.571662303
OXGR1	0.02021645	0.697925774	0.754058098
OXNAD1	-0.200206833	0.000103336	0.000284423
OXR1	-0.165344749	0.001393261	0.00309541
OXSM	-0.266423123	1.90E-07	8.46E-07
OXSR1	0.003698152	0.943404674	0.956317504
OXTR	0.366283533	3.19E-13	3.55E-12
OXT	0.146155367	0.004790198	0.009480865
P2RX1	0.178034084	0.000570701	0.001367843
P2RX2	-0.017797676	0.732593444	0.783299146
P2RX3	-0.212261306	3.76E-05	0.000112377
P2RX4	0.448276674	9.70E-20	2.86E-18
P2RX5	0.382654315	2.20E-14	2.92E-13
P2RX6	-0.051046893	0.326812687	0.400859645
P2RX7	0.026534006	0.610439562	0.67629724
P2RY10	0.124415018	0.016501142	0.028859254
P2RY11	0.204295902	7.38E-05	0.000208933
P2RY12	0.094598867	0.068753278	0.10341282
P2RY13	0.07216178	0.165425737	0.222420971
P2RY14	-0.10008511	0.054090789	0.083638366
P2RY1	0.000193655	0.99703389	0.99758471
P2RY2	-0.064295357	0.216638641	0.281366126
P2RY4	-0.012512624	0.81017035	0.848457852

P2RY6	0.336089968	3.01E-11	2.45E-10
P2RY8	-0.118860771	0.022031901	0.037498465
P4HA1	-0.015546124	0.76536196	0.810910013
P4HA2	0.251875234	8.90E-07	3.53E-06
P4HA3	0.115853515	0.025649015	0.042993662
P4HB	-0.086807983	0.09500834	0.137350876
P4HTM	0.113172109	0.029294589	0.048407996
P704P	0.156067433	0.00257472	0.005424447
PA2G4P4	0.427795879	6.11E-18	1.40E-16
PA2G4	0.47905713	1.11E-22	5.08E-21
PAAF1	-0.150878681	0.00357957	0.007281956
PABPC1L2A	0.043440725	0.404113186	0.480239972
PABPC1L2E	0.015113116	0.771714163	0.81616444
PABPC1L	0.296342404	5.89E-09	3.37E-08
PABPC1P2	0.164218723	0.001503658	0.003315537
PABPC1	0.216766278	2.54E-05	7.78E-05
PABPC3	0.178147985	0.00056599	0.001357368
PABPC4L	-0.035346334	0.497306945	0.571226301
PABPC4	0.163316389	0.001597856	0.003499998
PABPC5	0.069443885	0.181980533	0.241340446
PABPN1L	-0.069415215	0.182161458	0.241564306
PABPN1	0.328752962	8.46E-11	6.46E-10
PACRGL	0.163654452	0.001561948	0.003430019
PACRG	-0.422549099	1.69E-17	3.65E-16
PACS1	0.386821285	1.09E-14	1.51E-13
PACS2	0.294450393	7.42E-09	4.20E-08
PACSIN1	0.433679487	1.91E-18	4.75E-17
PACSIN2	-0.044455364	0.393213894	0.469612564
PACSIN3	-0.049282683	0.343830628	0.418129274
PADI1	-0.012639485	0.80828288	0.846881852
PADI2	0.277489916	5.51E-08	2.68E-07
PADI3	0.108903929	0.036011335	0.058151724
PADI4	-0.11702432	0.024184111	0.040795584
PADI6	-0.033031831	0.525908478	0.597942512
PAEP	0.285669273	2.13E-08	1.12E-07
PAF1	0.09873867	0.057424104	0.088183381
PAFAH1B1	-0.054044073	0.299172981	0.371416721
PAFAH1B2	-0.022052255	0.672021988	0.731806698
PAFAH1B3	0.497747845	1.28E-24	7.73E-23
PAFAH2	-0.376178188	6.46E-14	8.04E-13
PAG1	0.316225319	4.64E-10	3.15E-09
PAGE1	0.050392228	0.333063061	0.407122488
PAGE2B	0.083552363	0.108117574	0.153884283
PAGE2	0.034619099	0.506203961	0.579274818
PAGE3	0.037007741	0.477296503	0.551772816
PAGE4	-0.382379386	2.31E-14	3.05E-13
PAGE5	-0.130026944	0.012186593	0.022022977
PAH	-0.396038699	2.21E-15	3.42E-14
PAICS	-0.022059545	0.671919867	0.731754638
PAIP1	0.154943844	0.002767479	0.005771955
PAIP2B	-0.096339992	0.063783335	0.096762991
PAIP2	0.06160006	0.236562958	0.303503816
PAK1IP1	0.270616351	1.20E-07	5.50E-07
PAK1	0.348697395	4.78E-12	4.42E-11
PAK2	0.191050177	0.000214227	0.000555852
PAK3	0.21617989	2.67E-05	8.16E-05
PAK4	0.352068314	2.88E-12	2.75E-11
PAK6	0.476512893	1.99E-22	8.73E-21

PAK7	0.311724138	8.38E-10	5.48E-09
PALB2	0.196258103	0.000142088	0.000381236
PALLD	0.068306763	0.189258734	0.249811999
PALM2-AK/	-0.204825574	7.06E-05	0.000200555
PALM2	-0.254358155	6.88E-07	2.78E-06
PALM3	0.023699945	0.649097253	0.712015442
PALMD	-0.343668766	1.01E-11	8.85E-11
PALM	0.280480595	3.91E-08	1.96E-07
PAMR1	-0.001288842	0.980261476	0.98471002
PAM	0.171822682	0.000890173	0.002052257
PAN2	-0.304132397	2.22E-09	1.36E-08
PAN3	0.217704374	2.34E-05	7.21E-05
PANK1	-0.503608941	2.98E-25	2.06E-23
PANK2	0.06325751	0.22416333	0.289797655
PANK3	-0.011998843	0.817825684	0.855036645
PANK4	0.083233548	0.109474471	0.155504486
PANX1	-0.299833005	3.82E-09	2.25E-08
PANX2	-0.175989407	0.000661714	0.001568059
PANX3	0.143669782	0.005565598	0.010838499
PAOX	-0.343545119	1.02E-11	9.01E-11
PAPD4	0.124972006	0.016020109	0.028100608
PAPD5	-0.305464814	1.88E-09	1.16E-08
PAPD7	-0.009138926	0.86073564	0.890690599
PAPLN	0.502974654	3.49E-25	2.39E-23
PAPL	0.239672698	3.03E-06	1.10E-05
PAPOLA	0.212090433	3.82E-05	0.000113841
PAPOLB	0.080419911	0.122038129	0.170988463
PAPOLG	0.156730885	0.002466724	0.00521348
PAPPA2	-0.334976016	3.53E-11	2.85E-10
PAPPA	0.158577594	0.002187495	0.004668875
PAPSS1	0.259721205	3.92E-07	1.65E-06
PAPSS2	-0.247618817	1.37E-06	5.27E-06
PAQR3	-0.064044736	0.218438918	0.283408669
PAQR4	0.456326648	1.76E-20	5.74E-19
PAQR5	0.413048251	1.02E-16	1.97E-15
PAQR6	0.243830427	2.01E-06	7.49E-06
PAQR7	0.081911453	0.115244975	0.162669008
PAQR8	0.279878986	4.19E-08	2.08E-07
PAQR9	0.109782365	0.034531064	0.056033873
PAR-SN	0.200948943	9.73E-05	0.000269389
PAR1	0.141247741	0.00642785	0.012367744
PAR4	0.077186843	0.137829825	0.189879384
PAR5	0.052766811	0.310755923	0.383597465
PARD3B	-0.148735054	0.004089706	0.008209907
PARD3	0.022776637	0.661904444	0.723338471
PARD6A	-0.126478676	0.014779906	0.026133424
PARD6B	0.306189997	1.71E-09	1.07E-08
PARD6G	0.166899382	0.001253052	0.002806443
PARG	0.064972458	0.21182808	0.275873636
PARK2	-0.307689397	1.41E-09	8.92E-09
PARK7	-0.11467912	0.027194572	0.045306094
PARL	0.152769975	0.003178126	0.006546853
PARM1	0.178381807	0.000556431	0.001336051
PARN	0.001038688	0.984092005	0.98786971
PARP10	-0.035852588	0.491162742	0.565244304
PARP11	-0.050325139	0.333707894	0.407758971
PARP12	0.164779472	0.001447715	0.003205667
PARP14	0.00152619	0.976627475	0.98165351

PARP15	0.133631024	0.009972069	0.018415235
PARP16	-0.231410602	6.68E-06	2.28E-05
PARP1	0.383890373	1.79E-14	2.40E-13
PARP2	0.15228316	0.003277351	0.006719985
PARP3	-0.166575899	0.00128111	0.002863489
PARP4	-0.02048318	0.694139411	0.750659284
PARP6	-0.086286861	0.09701705	0.139954646
PARP8	0.378257869	4.58E-14	5.79E-13
PARP9	-0.06106756	0.240647299	0.30800999
PARS2	-0.028875879	0.579286325	0.648235812
PART1	0.012556529	0.809516987	0.847951912
PARVA	0.028566148	0.583363677	0.651782087
PARVB	-0.033254927	0.523115543	0.595208627
PARVG	0.280460099	3.92E-08	1.96E-07
PASD1	0.064744467	0.213439213	0.277681447
PASK	0.379259824	3.88E-14	4.97E-13
PATE1	-0.023377679	0.653555917	0.715982457
PATE2	0.05055728	0.331480037	0.405486019
PATE3	-0.047504149	0.361546574	0.437191554
PATE4	0.008973204	0.863236005	0.89248989
PATL1	0.118153347	0.02284047	0.038775274
PATL2	-0.109909854	0.034320593	0.055742284
PATZ1	-0.060731506	0.243250191	0.310862165
PAWR	-0.041248132	0.428272472	0.504645661
PAX1	0.028660956	0.582114191	0.650742322
PAX2	0.022218499	0.669694713	0.730089639
PAX3	0.084981299	0.102198101	0.146516305
PAX4	-0.04099543	0.431109684	0.507377568
PAX5	-0.016508479	0.751301006	0.79897124
PAX6	-0.043147604	0.407295103	0.483588596
PAX7	0.07227434	0.164765311	0.221637713
PAX8	0.172268727	0.000862628	0.00199504
PAX9	0.107892408	0.037782283	0.060711296
PAXIP1	0.102028866	0.049563688	0.077449823
PBK	0.510589828	5.06E-26	3.98E-24
PBLD	-0.339521908	1.84E-11	1.55E-10
PBOV1	0.190994085	0.000215164	0.000558092
PBRM1	0.08831924	0.089370234	0.130082107
PBX1	0.110999057	0.032566526	0.053201338
PBX2	0.133709924	0.009927869	0.018338978
PBX3	0.234209771	5.13E-06	1.79E-05
PBX4	0.28047147	3.91E-08	1.96E-07
PBXIP1	-0.2162244	2.66E-05	8.14E-05
PCA3	-0.002476309	0.962085877	0.970071609
PCBD1	-0.2728923	9.28E-08	4.34E-07
PCBD2	-0.024373912	0.639813212	0.703669467
PCBP1	0.028904663	0.578908089	0.647921738
PCBP2	-0.011267647	0.828750246	0.863964543
PCBP3	0.191810823	0.000201892	0.000526383
PCBP4	0.302243607	2.82E-09	1.70E-08
PCCA	-0.270956125	1.15E-07	5.31E-07
PCCB	-0.341005818	1.48E-11	1.27E-10
PCDH10	0.130232724	0.012049346	0.02180066
PCDH11X	0.155327712	0.002700195	0.005648776
PCDH11Y	0.009987483	0.847955173	0.879795998
PCDH12	-0.107046645	0.03931915	0.062938697
PCDH15	0.139435688	0.007149272	0.013621633
PCDH17	0.063392035	0.223177624	0.288691943

PCDH18	0.123881611	0.016973607	0.029615362
PCDH19	0.025318108	0.626900864	0.691764651
PCDH1	0.008640613	0.868258122	0.896430249
PCDH20	-0.16508599	0.00141795	0.003147103
PCDH7	0.145268467	0.005054932	0.009945127
PCDH8	0.081085269	0.118970238	0.167181003
PCDH9	0.15030656	0.003709757	0.007519908
PCDHA10	0.037361669	0.473091125	0.54792869
PCDHA11	0.131299001	0.01135981	0.020671368
PCDHA12	0.10444394	0.044384081	0.070198449
PCDHA13	0.081473585	0.117207777	0.165088613
PCDHA1	0.151113227	0.003527406	0.007181705
PCDHA2	0.217157852	2.45E-05	7.53E-05
PCDHA3	0.152958325	0.003140472	0.006475315
PCDHA4	0.176563198	0.000634903	0.001509552
PCDHA5	0.150657912	0.003629308	0.007374854
PCDHA6	0.129609747	0.012469087	0.022486571
PCDHA7	0.146177401	0.004783783	0.009470588
PCDHA8	0.081136199	0.118737908	0.166901616
PCDHA9	0.051036837	0.326908117	0.40095201
PCDHAC1	0.110540125	0.033296046	0.054268496
PCDHAC2	0.097598902	0.060377096	0.092178138
PCDHB10	0.085483154	0.100181157	0.143997764
PCDHB11	-0.033068743	0.52544585	0.597518821
PCDHB12	-0.024633705	0.636249337	0.70045089
PCDHB13	-0.045015506	0.387273563	0.463546258
PCDHB14	-0.057745162	0.267247736	0.336998884
PCDHB15	0.100118439	0.054010363	0.083532583
PCDHB16	0.04961966	0.340537276	0.414782564
PCDHB17	-0.043478525	0.403703929	0.479896753
PCDHB18	0.083419868	0.10867986	0.154600328
PCDHB19P	-0.029200392	0.575028743	0.644195165
PCDHB1	0.050290078	0.334045217	0.40792257
PCDHB2	0.122742154	0.018022647	0.031251386
PCDHB3	0.053042754	0.308228887	0.380879289
PCDHB4	-0.054508721	0.295031394	0.366961069
PCDHB5	0.050786339	0.329291158	0.403378125
PCDHB6	0.01638158	0.75315054	0.800611764
PCDHB7	-0.001907594	0.970788875	0.977017224
PCDHB8	-0.0057576	0.911992319	0.931134354
PCDHB9	0.065825325	0.205878804	0.269128447
PCDHGA10	0.032300243	0.535120176	0.606891959
PCDHGA11	0.099593474	0.055288773	0.085303312
PCDHGA12	0.09105377	0.079852724	0.117926313
PCDHGA1	0.024117514	0.643338625	0.706801969
PCDHGA2	-0.051968589	0.318142345	0.391429829
PCDHGA3	-0.0325592	0.531850311	0.603767844
PCDHGA4	-0.069086286	0.184246723	0.244004735
PCDHGA5	0.102383899	0.048771979	0.076405851
PCDHGA6	0.107467958	0.038547084	0.061805634
PCDHGA7	0.129291478	0.012688462	0.022822277
PCDHGA8	0.188085186	0.000269356	0.000685589
PCDHGA9	0.010386379	0.841960749	0.874897874
PCDHGB1	0.060200326	0.247404506	0.31538966
PCDHGB2	-0.054487305	0.295221438	0.367174522
PCDHGB3	0.019574514	0.707068955	0.761747119
PCDHGB4	0.123396924	0.017413128	0.030294676
PCDHGB5	0.031253543	0.548438945	0.619703968

PCDHGB6	0.0355022	0.495410928	0.569245387
PCDHGB7	0.005244138	0.919813167	0.937363441
PCDHGB8P	0.16942823	0.001052431	0.002391528
PCDHGC3	0.052535858	0.31288139	0.385743118
PCDHGC4	0.093948001	0.070690681	0.105895162
PCDHGC5	0.062648892	0.228661601	0.294580731
PCDP1	0.12461921	0.016323348	0.0285759
PCF11	-0.042532157	0.414024218	0.490046874
PCGEM1	-0.030390069	0.559547623	0.62989816
PCGF1	0.080162103	0.123243274	0.17245575
PCGF2	0.24367781	2.04E-06	7.59E-06
PCGF3	0.317059127	4.15E-10	2.84E-09
PCGF5	-0.088517471	0.088651021	0.129205182
PCGF6	0.057841628	0.26644807	0.336130855
PCID2	0.206682194	6.05E-05	0.00017396
PCIF1	0.128628211	0.013156599	0.023570662
PCK1	-0.352412053	2.74E-12	2.63E-11
PCK2	-0.478466648	1.27E-22	5.72E-21
PCLO	0.284642746	2.41E-08	1.25E-07
PCM1	0.123826262	0.017023303	0.029694269
PCMT1	0.128349864	0.013357545	0.023885558
PCMTD1	-0.089794328	0.084129033	0.12340907
PCMTD2	-0.115626649	0.025941541	0.043445802
PCNAP1	-0.014892727	0.774953153	0.818894431
PCNA	0.417683377	4.27E-17	8.67E-16
PCNP	0.188246895	0.000266036	0.000677486
PCNT	0.240976959	2.66E-06	9.75E-06
PCNXL2	0.425492168	9.57E-18	2.13E-16
PCNXL3	0.25971461	3.92E-07	1.65E-06
PCNX	-0.261853994	3.12E-07	1.34E-06
PCOLCE2	-0.189972927	0.000232906	0.000599864
PCOLCE	0.039441113	0.448797875	0.524580572
PCOTH	0.079577512	0.126010274	0.175759762
PCP2	0.169345982	0.00105846	0.002403858
PCP4L1	-0.051372523	0.323732207	0.397668826
PCP4	0.016290973	0.754471983	0.80160287
PCSK1N	0.362969389	5.38E-13	5.76E-12
PCSK1	0.013000829	0.802912801	0.842274173
PCSK2	0.047983512	0.356716451	0.431870498
PCSK4	-0.213418025	3.40E-05	0.000102262
PCSK5	0.116478839	0.024857373	0.041817986
PCSK6	-0.354681186	1.94E-12	1.91E-11
PCSK7	0.062837328	0.227262093	0.29296722
PCSK9	0.190164791	0.000229471	0.00059163
PCTP	-0.433465414	2.00E-18	4.93E-17
PCYOX1L	0.406202822	3.59E-16	6.31E-15
PCYOX1	-0.427623878	6.32E-18	1.44E-16
PCYT1A	-0.042685615	0.412340251	0.488530626
PCYT1B	0.210550829	4.36E-05	0.000128666
PCYT2	-0.332341528	5.12E-11	4.03E-10
PC	-0.453836265	3.00E-20	9.45E-19
PDAP1	0.079369625	0.127005788	0.176974841
PDCD10	0.234338301	5.06E-06	1.77E-05
PDCD11	0.062547337	0.229418376	0.29547924
PDCD1LG2	0.026683231	0.608432524	0.674336174
PDCD1	0.289935399	1.28E-08	6.99E-08
PDCD2L	0.186925079	0.000294329	0.000742982
PDCD2	0.236461204	4.13E-06	1.46E-05

PDCD4	0.127543728	0.013954838	0.024833254
PDCD5	0.338373711	2.17E-11	1.81E-10
PDCD6IP	-0.024702207	0.635311016	0.699643106
PDCD6	0.273008805	9.16E-08	4.28E-07
PDCD7	0.234593874	4.94E-06	1.73E-05
PDCL2	0.166325555	0.00130322	0.002908013
PDCL3	0.471845713	5.76E-22	2.32E-20
PDCL	0.253965887	7.17E-07	2.89E-06
PDC	0.05125976	0.324796813	0.398829026
PDDC1	0.121961442	0.018773659	0.032423827
PDE10A	0.064842673	0.212744148	0.27692185
PDE11A	-0.370369426	1.66E-13	1.93E-12
PDE12	-0.056533513	0.27743182	0.3479381
PDE1A	0.208902891	5.01E-05	0.000146275
PDE1B	0.061508293	0.237263314	0.304226025
PDE1C	0.058486418	0.261145151	0.33067652
PDE2A	-0.367866645	2.48E-13	2.81E-12
PDE3A	0.066819741	0.199095931	0.261411003
PDE3B	-0.208581559	5.15E-05	0.000149901
PDE4A	0.361526537	6.75E-13	7.10E-12
PDE4B	0.117810804	0.02324113	0.03939503
PDE4C	0.160574883	0.00191822	0.004140728
PDE4DIP	-0.20997534	4.58E-05	0.000134594
PDE4D	0.209462187	4.78E-05	0.000140055
PDE5A	0.150304953	0.003710128	0.007519908
PDE6A	0.075305446	0.147715828	0.20164415
PDE6B	0.334743905	3.65E-11	2.94E-10
PDE6C	0.024371669	0.639844011	0.703669467
PDE6D	0.130161136	0.012096937	0.021882846
PDE6G	0.124599756	0.016340215	0.028600401
PDE6H	0.081845583	0.115538591	0.162991065
PDE7A	0.572654972	1.04E-33	3.44E-31
PDE7B	-0.199972764	0.000105323	0.000289453
PDE8A	-0.109301678	0.035334535	0.057197904
PDE8B	-0.077106982	0.138238868	0.19041289
PDE9A	0.279504736	4.38E-08	2.17E-07
PDF	-0.171171857	0.000931823	0.002138684
PDGFA	0.253011783	7.91E-07	3.16E-06
PDGFB	0.098413434	0.058254292	0.089299939
PDGFC	-0.249328877	1.15E-06	4.50E-06
PDGFD	-0.092359905	0.075605483	0.112278228
PDGFRA	0.160966822	0.001869078	0.004045608
PDGFRB	0.155230035	0.002717173	0.005679522
PDGFRL	0.413868408	8.74E-17	1.70E-15
PDHA1	0.163835292	0.001543045	0.003392996
PDHA2	0.089825565	0.084020782	0.123291357
PDHB	-0.076905547	0.139274752	0.191667952
PDHX	-0.074264648	0.153411889	0.208391631
PDIA2	0.092741281	0.074400572	0.110722075
PDIA3P	0.200280305	0.000102719	0.000282882
PDIA3	0.232763494	5.88E-06	2.03E-05
PDIA4	-0.039631701	0.446607163	0.522345423
PDIA5	-0.149300105	0.003949219	0.007959165
PDIA6	0.200192498	0.000103456	0.000284676
PDIK1L	-0.126732033	0.01457985	0.025827829
PDILT	-0.137613965	0.007946673	0.014993239
PDK1	-0.114364569	0.027621882	0.045926769
PDK2	-0.426457804	7.93E-18	1.78E-16

PDK3	0.291438617	1.07E-08	5.88E-08
PDK4	-0.484890556	2.83E-23	1.39E-21
PDLIM1	-0.23275865	5.88E-06	2.03E-05
PDLIM2	-0.276423165	6.23E-08	3.00E-07
PDLIM3	0.173586944	0.000785757	0.001832788
PDLIM4	0.248722402	1.23E-06	4.76E-06
PDLIM5	-0.213283879	3.44E-05	0.000103326
PDLIM7	0.495913193	2.00E-24	1.17E-22
PDP1	0.355116401	1.82E-12	1.79E-11
PDP2	-0.292072169	9.91E-09	5.48E-08
PDPK1	-0.165995446	0.001332909	0.002969939
PDPN	0.268450282	1.52E-07	6.88E-07
PDPR	-0.011269762	0.828718589	0.863964543
PDRG1	0.3213228	2.34E-10	1.66E-09
PDS5A	0.189939334	0.000233512	0.000600882
PDS5B	-0.078442664	0.131519079	0.182345542
PDSS1	0.516079186	1.22E-26	1.03E-24
PDSS2	-0.226591852	1.05E-05	3.45E-05
PDX1	0.342336803	1.22E-11	1.06E-10
PDXDC1	-0.183909796	0.000369702	0.000916095
PDXDC2	-0.080998978	0.119364684	0.16768798
PDXK	0.154182977	0.00290537	0.006029247
PDXP	-0.431573778	2.90E-18	6.96E-17
PDYN	0.174635115	0.000729207	0.001711506
PDZD11	0.277315953	5.63E-08	2.73E-07
PDZD2	-0.040893631	0.432255695	0.50831698
PDZD3	0.143863916	0.005501206	0.010722534
PDZD4	0.07000145	0.178488295	0.237547185
PDZD7	0.343516662	1.03E-11	9.04E-11
PDZD8	-0.011210424	0.829606611	0.864721545
PDZD9	0.02856917	0.583323827	0.651782087
PDZK1IP1	0.036099995	0.488174902	0.562293039
PDZK1P1	-0.102929505	0.047575925	0.074742337
PDZK1	-0.178443024	0.000553953	0.001330262
PDZRN3	0.155035469	0.002751282	0.005742131
PDZRN4	0.010302939	0.843213908	0.875894535
PEA15	0.38306518	2.06E-14	2.73E-13
PEAR1	-0.025607066	0.622971637	0.687924226
PEBP1	-0.461042916	6.34E-21	2.22E-19
PEBP4	-0.199354855	0.000110744	0.000303322
PECAM1	-0.032280865	0.53536527	0.607135362
PECI	-0.469561747	9.63E-22	3.79E-20
PECR	-0.406516637	3.39E-16	5.99E-15
PEF1	-0.134871962	0.009296729	0.017285062
PEG10	0.139399402	0.00716443	0.013645293
PEG3AS	-0.073192897	0.159449607	0.215435715
PEG3	0.052863574	0.309868242	0.382667686
PELI1	0.365723235	3.49E-13	3.86E-12
PELI2	0.097882704	0.059630327	0.091128825
PELI3	-0.104000369	0.045299761	0.071482125
PELO	0.225743878	1.13E-05	3.70E-05
PELP1	0.198271631	0.000120884	0.00032886
PEMT	-0.351310268	3.23E-12	3.07E-11
PENK	0.09803692	0.059227747	0.090576125
PEPD	-0.283661467	2.70E-08	1.39E-07
PER1	-0.216210014	2.67E-05	8.14E-05
PER2	-0.318009282	3.66E-10	2.52E-09
PER3	-0.269832385	1.31E-07	5.96E-07

PER4	-0.035917833	0.490373857	0.564499479
PERP	0.210995073	4.19E-05	0.000124229
PES1	0.193210366	0.000180915	0.000476176
PET112L	-0.380001806	3.43E-14	4.41E-13
PEX10	-0.094398629	0.069344633	0.104171226
PEX11A	-0.417939265	4.07E-17	8.28E-16
PEX11B	-0.094446692	0.069202314	0.103986159
PEX11G	-0.50940174	6.86E-26	5.20E-24
PEX12	-0.388768965	7.80E-15	1.11E-13
PEX13	-0.231366815	6.71E-06	2.29E-05
PEX14	-0.297837121	4.90E-09	2.83E-08
PEX16	-0.09373371	0.071338228	0.106728761
PEX19	-0.348593938	4.86E-12	4.48E-11
PEX1	-0.319288118	3.08E-10	2.15E-09
PEX26	-0.093842749	0.071008132	0.106282795
PEX2	-0.035413261	0.496492354	0.570422184
PEX3	-0.090634678	0.081255734	0.119696594
PEX5L	0.132251498	0.0107736	0.019719925
PEX5	-0.345352189	7.86E-12	7.04E-11
PEX6	-0.18036529	0.000481161	0.001168275
PEX7	-0.192637549	0.000189241	0.000495895
PF4V1	0.159253324	0.002092756	0.004487824
PF4	0.069798072	0.179756329	0.23894746
PFAS	0.184853032	0.000344383	0.000859318
PFDN1	0.44688651	1.30E-19	3.76E-18
PFDN2	0.257393527	5.01E-07	2.07E-06
PFDN4	0.459522583	8.83E-21	3.01E-19
PFDN5	0.087229818	0.093406758	0.135304984
PFDN6	0.099882631	0.054581538	0.084344818
PFKFB1	-0.380850896	2.98E-14	3.87E-13
PFKFB2	0.244150272	1.95E-06	7.27E-06
PFKFB3	0.295563505	6.48E-09	3.70E-08
PFKFB4	0.282144627	3.22E-08	1.64E-07
PFKL	-0.053894507	0.300514303	0.3729425
PFKM	0.142933081	0.005816161	0.011282334
PFKP	0.337942216	2.31E-11	1.92E-10
PFN1	0.300922	3.33E-09	1.99E-08
PFN2	0.197662142	0.000126966	0.000343671
PFN3	-0.115656936	0.025902323	0.04338541
PFN4	0.032879388	0.527821261	0.599816797
PGA3	0.072500289	0.16344558	0.220144875
PGA4	0.060558861	0.244595044	0.312369353
PGA5	0.061537063	0.237043585	0.303983412
PGAM1	0.043680003	0.40152675	0.477650679
PGAM2	0.115100822	0.026630622	0.044478139
PGAM4	0.007963234	0.878502729	0.904284973
PGAM5	0.154836053	0.002786645	0.005807044
PGAP1	-0.021897867	0.674186097	0.733581049
PGAP2	-0.023768799	0.648146245	0.711111878
PGAP3	-0.252818425	8.07E-07	3.22E-06
PGBD1	0.227761578	9.40E-06	3.12E-05
PGBD2	-0.017825718	0.732188235	0.783049926
PGBD3	0.010615413	0.838523029	0.872235578
PGBD4	-0.221579923	1.65E-05	5.24E-05
PGBD5	0.153948468	0.002949111	0.006113652
PGCP	-0.141457751	0.006348606	0.012235362
PGC	0.208890237	5.02E-05	0.00014641
PGD	0.254572783	6.73E-07	2.73E-06

PGF	0.337948816	2.31E-11	1.92E-10
PGGT1B	0.036120609	0.487926396	0.562039298
PGK1	0.201985973	8.93E-05	0.000248862
PGK2	0.075340014	0.147529449	0.201403529
PGLS	0.141603231	0.006294227	0.012138779
PGLYRP1	-0.013227715	0.799545685	0.839626226
PGLYRP2	-0.092903566	0.073892617	0.110079168
PGLYRP3	0.13725429	0.008113177	0.015267237
PGLYRP4	0.249822098	1.10E-06	4.29E-06
PGM1	-0.518068969	7.24E-27	6.27E-25
PGM2L1	0.272025998	1.02E-07	4.74E-07
PGM2	-0.04818736	0.35467478	0.42955991
PGM3	0.128367929	0.013344422	0.023870663
PGM5P2	-0.150549276	0.003654012	0.007422025
PGM5	-0.289063987	1.42E-08	7.68E-08
PGPEP1L	0.112092114	0.030883711	0.050705892
PGPEP1	-0.152441512	0.00324477	0.006662095
PGP	0.298896288	4.29E-09	2.51E-08
PGRMC1	-0.437889901	8.21E-19	2.15E-17
PGRMC2	-0.425243003	1.00E-17	2.23E-16
PGR	0.075346781	0.147492981	0.201381343
PGS1	0.340888359	1.51E-11	1.29E-10
PHACTR1	0.077821124	0.134613981	0.186066727
PHACTR2	0.062761882	0.227821691	0.293669604
PHACTR3	0.079409073	0.126816412	0.176760411
PHACTR4	-0.124958126	0.016031945	0.028117652
PHAX	0.148453383	0.00416141	0.008338726
PHB2	-0.099053695	0.056629345	0.08711736
PHB	0.062675407	0.228464305	0.294383668
PHC1	0.314023964	6.20E-10	4.12E-09
PHC2	0.190645383	0.000221075	0.00057213
PHC3	0.023487503	0.652035064	0.714730449
PHEX	0.318815814	3.28E-10	2.28E-09
PHF10	0.137765021	0.007877658	0.014872887
PHF11	0.002581915	0.960470261	0.968932982
PHF12	0.171022713	0.000941618	0.002158926
PHF13	0.047780773	0.358754309	0.434157656
PHF14	-0.045623073	0.380892286	0.457089098
PHF15	0.354718924	1.93E-12	1.90E-11
PHF16	0.05411904	0.298502167	0.370731703
PHF17	-0.128618348	0.013163673	0.023576975
PHF19	0.524295215	1.38E-27	1.36E-25
PHF1	0.09236484	0.075589791	0.112263293
PHF20L1	0.080630028	0.121062722	0.169810009
PHF20	0.019882765	0.702673145	0.758278283
PHF21A	0.387290291	1.00E-14	1.40E-13
PHF21B	0.171441824	0.000914333	0.002101204
PHF23	0.036891635	0.47868051	0.552955649
PHF2	0.006986584	0.893308756	0.91569447
PHF3	-0.05803445	0.264854575	0.334438286
PHF5A	0.235929364	4.35E-06	1.54E-05
PHF6	0.161355464	0.001821489	0.003950328
PHF7	-0.231088863	6.89E-06	2.35E-05
PHF8	-0.300322234	3.59E-09	2.13E-08
PHGDH	0.119476104	0.021348875	0.036454297
PHGR1	-0.143481619	0.005628655	0.010949524
PHIP	0.321640046	2.24E-10	1.60E-09
PHKA1	0.03820573	0.463144421	0.538097811

PHKA2	0.157398961	0.002362162	0.005011606
PHKB	-0.351072395	3.35E-12	3.17E-11
PHKG1	-0.014729811	0.777349987	0.821035227
PHKG2	0.104013319	0.045272806	0.07144525
PHLDA1	-0.197078174	0.000133062	0.000358807
PHLDA2	0.428578151	5.24E-18	1.21E-16
PHLDA3	0.07486118	0.150127194	0.204593931
PHLDB1	0.334488266	3.78E-11	3.04E-10
PHLDB2	-0.155753699	0.002627274	0.005518582
PHLDB3	-0.103624856	0.046087294	0.072581113
PHLPP1	-0.480479512	7.95E-23	3.70E-21
PHLPP2	-0.058679632	0.259570363	0.328912401
PHOSPHO1	0.002950565	0.954831781	0.964808214
PHOSPHO2	0.049695103	0.339802724	0.414014425
PHOX2A	0.115007089	0.026755095	0.044656083
PHOX2B	0.072940885	0.160894975	0.217141627
PHPT1	0.275215685	7.14E-08	3.40E-07
PHRF1	-0.003698857	0.943393903	0.956317504
PHTF1	0.107802748	0.037942758	0.060952147
PHTF2	0.340413075	1.62E-11	1.37E-10
PHYHD1	-0.260649853	3.55E-07	1.50E-06
PHYHIPL	-0.099773116	0.054848505	0.084695027
PHYHIP	0.002905742	0.955517224	0.965304976
PHYH	-0.200319925	0.000102388	0.000282126
PI15	0.056816656	0.27502871	0.34535909
PI16	0.149465482	0.003908936	0.007888353
PI3	0.359391261	9.41E-13	9.65E-12
PI4K2A	0.021255391	0.683220595	0.741306067
PI4K2B	-0.350608898	3.59E-12	3.39E-11
PI4KAP1	0.223757211	1.36E-05	4.37E-05
PI4KAP2	0.244991167	1.79E-06	6.72E-06
PI4KA	0.15867297	0.00217389	0.004642324
PI4KB	0.288448798	1.53E-08	8.20E-08
PIAS1	-0.033860352	0.515574625	0.588241091
PIAS2	0.044763103	0.38994353	0.466321808
PIAS3	0.282119684	3.23E-08	1.64E-07
PIAS4	0.110631762	0.033149276	0.05405139
PIBF1	-0.040515871	0.436523691	0.512650945
PICALM	0.121550357	0.019179955	0.033056757
PICK1	0.002477812	0.962062888	0.970071609
PID1	-0.260015376	3.79E-07	1.60E-06
PIF1	0.556836454	1.35E-31	2.69E-29
PIGA	0.31700637	4.18E-10	2.86E-09
PIGB	0.042532982	0.414015154	0.490046874
PIGC	0.301808827	2.98E-09	1.79E-08
PIGF	-0.019717799	0.705024417	0.760107739
PIGG	0.055347844	0.287649317	0.359124503
PIGH	0.003294779	0.949569069	0.960903895
PIGK	-0.187205012	0.000288112	0.000729138
PIGL	0.03503844	0.501063578	0.574613666
PIGM	0.010741254	0.83663551	0.870635846
PIGN	-0.175473298	0.000686722	0.001621149
PIGO	-0.050423474	0.332763004	0.406880536
PIGP	-0.299427535	4.02E-09	2.36E-08
PIGQ	-0.13887467	0.007386859	0.014026021
PIGR	0.059588366	0.252251626	0.320760637
PIGS	0.434052958	1.77E-18	4.42E-17
PIGT	0.14172511	0.006248991	0.012055039

PIGU	0.418333757	3.77E-17	7.73E-16
PIGV	-0.511073219	4.47E-26	3.55E-24
PIGW	0.063192052	0.224644081	0.290257568
PIGX	0.290301348	1.23E-08	6.71E-08
PIGY	-0.420081573	2.71E-17	5.67E-16
PIGZ	0.230276152	7.44E-06	2.52E-05
PIH1D1	0.167655868	0.001189629	0.002677035
PIH1D2	0.100137104	0.053965367	0.083496275
PIK3AP1	0.123185322	0.017608108	0.030588483
PIK3C2A	-0.031462489	0.545767237	0.616930032
PIK3C2B	0.140232626	0.006823519	0.013054656
PIK3C2G	-0.043479863	0.403689455	0.479896753
PIK3C3	-0.043240433	0.406285807	0.482562801
PIK3CA	-0.104626538	0.044011683	0.069631571
PIK3CB	0.122549412	0.018205578	0.03153291
PIK3CD	0.271694166	1.06E-07	4.91E-07
PIK3CG	0.089439477	0.085366669	0.125003659
PIK3IP1	0.006236944	0.904698774	0.925178839
PIK3R1	-0.363145102	5.24E-13	5.62E-12
PIK3R2	0.308704644	1.24E-09	7.89E-09
PIK3R3	-0.099965003	0.054381448	0.084050023
PIK3R4	-0.211211178	4.12E-05	0.000122055
PIK3R5	0.203212905	8.08E-05	0.000226743
PIK3R6	0.400955146	9.24E-16	1.53E-14
PIKFYVE	-0.052775914	0.310672347	0.383518063
PILRA	0.234223491	5.12E-06	1.79E-05
PILRB	-0.051749919	0.320185661	0.393773612
PIM1	-0.18151526	0.000441971	0.001079964
PIM2	0.240111506	2.90E-06	1.05E-05
PIM3	0.119379431	0.021454952	0.036622873
PIN1L	0.100635066	0.052776344	0.081853665
PIN1	0.039080867	0.45295528	0.528509728
PIN4	-0.096412438	0.063583121	0.096488645
PINK1	-0.438971332	6.60E-19	1.75E-17
PINX1	0.015321173	0.768660045	0.813668726
PION	-0.24678396	1.49E-06	5.70E-06
PIP4K2A	0.45814628	1.19E-20	3.98E-19
PIP4K2B	0.100582928	0.052899806	0.082025991
PIP4K2C	0.220690086	1.79E-05	5.65E-05
PIP5K1A	0.203469453	7.91E-05	0.00022236
PIP5K1B	0.081761366	0.11591484	0.163452396
PIP5K1C	0.362209343	6.07E-13	6.43E-12
PIP5K1P1	0.127725721	0.013817979	0.024627104
PIP5KL1	0.269984733	1.28E-07	5.87E-07
PIPOX	-0.447185491	1.22E-19	3.55E-18
PIPSL	0.050220551	0.334714768	0.408640006
PIP	0.253992825	7.15E-07	2.88E-06
PIRT	-0.068354737	0.188947424	0.249483734
PIR	-0.1016759	0.050361403	0.078542341
PISD	0.100133026	0.053975194	0.083504995
PITPNA	-0.021083837	0.685640719	0.743386774
PITPNB	-0.019071321	0.714265608	0.767880818
PITPNC1	-0.205396982	6.74E-05	0.000192015
PITPNM1	0.204033046	7.55E-05	0.000213148
PITPNM2	-0.264823082	2.26E-07	9.94E-07
PITPNM3	0.08830895	0.089407693	0.130127123
PITRM1	0.116913605	0.024319473	0.040996408
PITX1	0.489690523	9.04E-24	4.76E-22

PITX2	0.279984743	4.14E-08	2.06E-07
PITX3	0.142930611	0.005817018	0.011282897
PIWIL1	0.096478686	0.063400489	0.09625549
PIWIL2	-0.152576936	0.003217141	0.006612864
PIWIL3	0.140624058	0.006668436	0.012784966
PIWIL4	0.406052055	3.69E-16	6.48E-15
PJA1	0.323148004	1.83E-10	1.32E-09
PJA2	-0.148307215	0.004199066	0.008405727
PKD1L1	0.060197568	0.247426212	0.31538966
PKD1L2	0.090124476	0.082990584	0.121981586
PKD1L3	-0.119754521	0.021045914	0.035986327
PKD1	0.091820816	0.077335673	0.114599917
PKD2L1	-0.141500204	0.006332694	0.012205876
PKD2L2	0.086064387	0.097884834	0.141002289
PKD2	-0.053295258	0.305928401	0.378506123
PKDCC	0.370057685	1.75E-13	2.03E-12
PKDREJ	-0.186408534	0.000306129	0.000771207
PKHD1L1	0.189958228	0.000233171	0.000600314
PKHD1	0.006986502	0.89331001	0.91569447
PKIA	0.367686069	2.55E-13	2.88E-12
PKIB	0.340390652	1.62E-11	1.38E-10
PKIG	-0.174545743	0.000733878	0.001722063
PKLR	-0.30857653	1.26E-09	8.00E-09
PKM2	0.564779308	1.21E-32	3.13E-30
PKMYT1	0.500303499	6.79E-25	4.32E-23
PKN1	0.302073569	2.89E-09	1.74E-08
PKN2	-0.121892419	0.01884135	0.032532274
PKN3	0.237489849	3.74E-06	1.33E-05
PKNOX1	0.347891866	5.39E-12	4.93E-11
PKNOX2	0.157055494	0.002415403	0.005115303
PKP1	0.133477701	0.01005846	0.018554134
PKP2	-0.232030981	6.30E-06	2.17E-05
PKP3	0.357067705	1.35E-12	1.35E-11
PKP4	-0.001381079	0.9788492	0.983588911
PL-5283	-0.072188813	0.165266945	0.222237601
PLA1A	0.04227213	0.416886845	0.492803639
PLA2G10	0.309417952	1.13E-09	7.24E-09
PLA2G12A	-0.325449078	1.33E-10	9.84E-10
PLA2G12B	-0.194403375	0.000164662	0.000436514
PLA2G15	-0.01066876	0.837722749	0.871536341
PLA2G16	-0.106924916	0.039544641	0.063272696
PLA2G1B	0.245215214	1.75E-06	6.58E-06
PLA2G2A	0.084031238	0.106104406	0.151419196
PLA2G2C	-0.01141638	0.826525332	0.862141351
PLA2G2D	0.159346581	0.002079979	0.004462827
PLA2G2E	0.033316889	0.522341189	0.594497325
PLA2G2F	0.110320027	0.033650829	0.054766098
PLA2G3	0.031836174	0.541005149	0.6126246
PLA2G4A	0.238610838	3.36E-06	1.21E-05
PLA2G4C	0.097167361	0.061527397	0.093668715
PLA2G4D	0.344920799	8.37E-12	7.47E-11
PLA2G4E	0.23162707	6.55E-06	2.24E-05
PLA2G4F	0.369704486	1.85E-13	2.13E-12
PLA2G5	-0.005309997	0.918809583	0.936543262
PLA2G6	0.057655749	0.267990399	0.337777093
PLA2G7	0.333908313	4.11E-11	3.28E-10
PLA2R1	0.209240001	4.87E-05	0.000142477
PLAA	-0.00851066	0.87022187	0.897895178

PLAC1	0.297567495	5.06E-09	2.93E-08
PLAC2	0.142990956	0.005796117	0.011244663
PLAC4	0.131286158	0.011367904	0.020684206
PLAC8L1	0.121751397	0.01898031	0.032746665
PLAC8	0.14244744	0.005986828	0.011587446
PLAC9	0.025191584	0.628624661	0.693375259
PLAG1	0.088079788	0.090245237	0.131221488
PLAGL1	0.310899948	9.33E-10	6.06E-09
PLAGL2	0.309280663	1.15E-09	7.35E-09
PLAT	0.036977635	0.477655164	0.552091331
PLAUR	0.452059424	4.37E-20	1.34E-18
PLAU	0.294699673	7.20E-09	4.08E-08
PLB1	0.222141462	1.57E-05	5.00E-05
PLBD1	0.326621225	1.14E-10	8.48E-10
PLBD2	0.100665653	0.052704025	0.081760597
PLCB1	0.214818713	3.01E-05	9.13E-05
PLCB2	0.239114178	3.20E-06	1.15E-05
PLCB3	0.416748676	5.09E-17	1.03E-15
PLCB4	0.092149445	0.076277167	0.113182907
PLCD1	-0.098949938	0.056890095	0.08747121
PLCD3	0.274995861	7.32E-08	3.49E-07
PLCD4	0.379157836	3.95E-14	5.05E-13
PLCE1	0.30723889	1.50E-09	9.41E-09
PLCG1	0.113620999	0.028654996	0.047477116
PLCG2	-0.266715397	1.84E-07	8.22E-07
PLCH1	0.14611162	0.004802957	0.00950477
PLCH2	0.002716215	0.958415905	0.967399385
PLCL1	0.143623477	0.005581057	0.010866479
PLCL2	-0.12865149	0.013139913	0.023542887
PLCXD1	0.275904948	6.61E-08	3.17E-07
PLCXD2	0.189668723	0.00023845	0.00061248
PLCXD3	0.011533359	0.824776391	0.860948141
PLCZ1	0.042522563	0.414129627	0.490046874
PLD1	0.196340693	0.000141154	0.000378933
PLD2	0.166868471	0.001255709	0.002811445
PLD3	0.055410357	0.287104382	0.358556513
PLD4	0.301368904	3.15E-09	1.89E-08
PLD5	0.050760485	0.329537757	0.403580943
PLD6	0.117667345	0.023410725	0.039655511
PLDN	-0.054324317	0.296670459	0.368677492
PLEC	0.203648238	7.79E-05	0.000219434
PLEK2	0.263780164	2.53E-07	1.10E-06
PLEKHA1	0.020298593	0.696758902	0.753067736
PLEKHA2	0.346333303	6.80E-12	6.15E-11
PLEKHA3	-0.170682593	0.00096431	0.002207652
PLEKHA4	0.200203179	0.000103366	0.000284468
PLEKHA5	-0.143356076	0.005671084	0.011021298
PLEKHA6	0.113365035	0.029018218	0.047995097
PLEKHA7	-0.076475376	0.14150683	0.194300026
PLEKHA8	0.042358371	0.41593613	0.491884101
PLEKHA9	0.346110367	7.03E-12	6.33E-11
PLEKHB1	0.467628825	1.48E-21	5.65E-20
PLEKHB2	0.422831511	1.60E-17	3.47E-16
PLEKHF1	-0.023312547	0.654458552	0.716656037
PLEKHF2	0.242690028	2.25E-06	8.32E-06
PLEKHG1	-0.060360214	0.246148865	0.314042759
PLEKHG2	0.483963379	3.52E-23	1.70E-21
PLEKHG3	0.012742726	0.806747632	0.845540106

PLEKHG4B	0.168757139	0.001102567	0.002497764
PLEKHG4	0.368243815	2.34E-13	2.66E-12
PLEKHG5	0.258723581	4.35E-07	1.82E-06
PLEKHG6	0.255464875	6.13E-07	2.50E-06
PLEKHG7	-0.00470503	0.928032962	0.943905278
PLEKHH1	0.301781549	2.99E-09	1.80E-08
PLEKHH2	0.333210391	4.53E-11	3.60E-10
PLEKHH3	0.103666686	0.045999004	0.072470713
PLEKHJ1	0.223294123	1.42E-05	4.55E-05
PLEKHM1P	0.15412129	0.002916818	0.006050484
PLEKHM1	0.001190379	0.981769167	0.98592627
PLEKHM2	0.140630813	0.006665788	0.012781119
PLEKHM3	-0.317473606	3.93E-10	2.70E-09
PLEKHN1	0.249095856	1.18E-06	4.59E-06
PLEKHO1	0.298128165	4.72E-09	2.74E-08
PLEKHO2	0.248953734	1.20E-06	4.66E-06
PLEK	0.253895006	7.22E-07	2.91E-06
PLGLA	-0.341873796	1.31E-11	1.13E-10
PLGLB2	-0.057791691	0.266861821	0.336610181
PLG	-0.482288642	5.22E-23	2.46E-21
PLIN1	-0.298488651	4.52E-09	2.64E-08
PLIN2	-0.040053981	0.441774903	0.517495126
PLIN3	0.411871509	1.27E-16	2.41E-15
PLIN4	-0.238418558	3.42E-06	1.23E-05
PLIN5	-0.305018283	1.99E-09	1.23E-08
PLK1S1	-0.196592928	0.000138336	0.00037197
PLK1	0.614301332	7.31E-40	1.12E-36
PLK2	-0.019498895	0.708148801	0.762621785
PLK3	0.197167872	0.000132108	0.000356331
PLK4	0.496214635	1.86E-24	1.09E-22
PLK5P	0.054193988	0.297832526	0.37000621
PLLP	0.009617613	0.853521246	0.884833755
PLN	-0.015642303	0.763953141	0.809718795
PLOD1	-0.067042134	0.197601511	0.25960283
PLOD2	0.227820273	9.35E-06	3.11E-05
PLOD3	0.179136639	0.000526584	0.001270049
PLP1	0.027805126	0.593438039	0.660990306
PLP2	0.549612319	1.14E-30	1.78E-28
PLRG1	-0.05439024	0.29608381	0.368086219
PLS1	-0.113209205	0.029241273	0.048331921
PLS3	-0.106870651	0.039645514	0.063423909
PLSCR1	0.277624556	5.43E-08	2.64E-07
PLSCR2	0.168561928	0.001117558	0.00252885
PLSCR3	0.088622157	0.088273082	0.128708553
PLSCR4	-0.206025893	6.39E-05	0.000183041
PLSCR5	0.059553824	0.252527173	0.321049544
PLTP	0.292173588	9.79E-09	5.42E-08
PLUNC	0.243385895	2.10E-06	7.79E-06
PLVAP	-0.230490664	7.29E-06	2.47E-05
PLXDC1	0.380004626	3.43E-14	4.41E-13
PLXDC2	0.158238658	0.002236475	0.004765757
PLXNA1	0.515083443	1.58E-26	1.33E-24
PLXNA2	0.036057449	0.488688015	0.562818976
PLXNA3	0.389306264	7.12E-15	1.03E-13
PLXNA4	0.104477095	0.044316268	0.07009676
PLXNB1	0.085244771	0.101135236	0.145201511
PLXNB2	-0.092339354	0.07567086	0.11236694
PLXNB3	0.238196964	3.49E-06	1.25E-05

PLXNC1	0.18560365	0.000325399	0.000815113
PLXND1	0.262238041	2.99E-07	1.29E-06
PM20D1	0.148894586	0.004049591	0.008136758
PM20D2	0.361318981	6.97E-13	7.31E-12
PMAIP1	0.416638682	5.20E-17	1.05E-15
PMCHL1	0.079360615	0.127049069	0.177007278
PMCHL2	0.221577323	1.65E-05	5.24E-05
PMCH	0.301662485	3.04E-09	1.82E-08
PMEPA1	0.312293797	7.78E-10	5.10E-09
PMF1	0.026878749	0.605807297	0.671949945
PMFBP1	0.152751904	0.00318176	0.006550952
PML	0.268405364	1.53E-07	6.91E-07
PMM1	-0.19322342	0.000180729	0.000475876
PMM2	0.150499313	0.003665424	0.007440671
PMP22	0.194657703	0.00016138	0.00042861
PMP2	0.101369505	0.051062528	0.079505095
PMPCA	-0.157774901	0.002305115	0.004897356
PMPCB	-0.269793321	1.31E-07	5.99E-07
PMS1	0.140321237	0.006788132	0.012994442
PMS2CL	0.119211643	0.021640146	0.036913691
PMS2L11	0.11487971	0.026925052	0.044905892
PMS2L1	-0.141558741	0.006310812	0.012167232
PMS2L2	-0.349217735	4.43E-12	4.12E-11
PMS2L3	0.255736122	5.96E-07	2.43E-06
PMS2L4	0.068929267	0.185248337	0.24513566
PMS2L5	-0.249488352	1.14E-06	4.43E-06
PMS2	0.062213292	0.231920158	0.298296429
PMVK	-0.12757109	0.013934185	0.024805365
PNCK	0.35960433	9.10E-13	9.36E-12
PNKD	0.071930177	0.16679085	0.224074942
PNKP	0.315136676	5.35E-10	3.59E-09
PNLDC1	0.249387051	1.15E-06	4.47E-06
PNLIPRP1	0.055124363	0.289603127	0.361155747
PNLIPRP2	0.162506785	0.001686946	0.003678158
PNLIPRP3	0.004615635	0.929396734	0.94505113
PNLIP	0.050719231	0.329931475	0.403952782
PNMA1	0.426325023	8.14E-18	1.83E-16
PNMA2	0.274046667	8.15E-08	3.84E-07
PNMA3	0.11715823	0.024021259	0.040551777
PNMA5	0.186178061	0.000311535	0.000783736
PNMA6A	-0.015882053	0.760444691	0.806858709
PNMAL1	0.27741653	5.56E-08	2.70E-07
PNMAL2	0.010915386	0.834025183	0.868373037
PNMT	-0.201111136	9.60E-05	0.000265951
PNN	0.164134081	0.001512273	0.003332687
PNO1	0.267812615	1.63E-07	7.33E-07
PNOC	0.210011031	4.56E-05	0.000134244
PNPLA1	0.264008959	2.47E-07	1.08E-06
PNPLA2	-0.338951942	2.00E-11	1.68E-10
PNPLA3	-0.165002113	0.001426039	0.003162238
PNPLA4	-0.293100652	8.75E-09	4.90E-08
PNPLA5	-0.132778111	0.010461133	0.019197375
PNPLA6	0.076760902	0.140022256	0.192553558
PNPLA7	-0.370579805	1.61E-13	1.87E-12
PNPLA8	-0.190939622	0.000216077	0.000559939
PNPO	-0.495007454	2.50E-24	1.43E-22
PNPT1	0.207480257	5.65E-05	0.000163459
PNP	0.376405643	6.22E-14	7.75E-13

PNRC1	-0.001834389	0.971909401	0.977897934
PNRC2	0.118784418	0.02211796	0.037632078
POC1A	0.195990042	0.000145161	0.000388747
POC1B	0.19320805	0.000180948	0.0004762
POC5	0.490972171	6.64E-24	3.58E-22
PODNL1	0.23499214	4.76E-06	1.67E-05
PODN	0.136766619	0.008343898	0.015658171
PODXL2	0.232711689	5.91E-06	2.04E-05
PODXL	0.129245533	0.01272041	0.02286962
POF1B	0.380640074	3.09E-14	3.99E-13
POFUT1	-0.234383393	5.04E-06	1.76E-05
POFUT2	0.238470389	3.40E-06	1.22E-05
POGK	0.451979188	4.45E-20	1.36E-18
POGZ	0.150634519	0.003634615	0.007384885
POLA1	0.449674047	7.24E-20	2.17E-18
POLA2	0.392725238	3.94E-15	5.91E-14
POLB	-0.164403477	0.001485011	0.003279866
POLD1	0.431587786	2.90E-18	6.95E-17
POLD2	-0.156457741	0.002510676	0.005298484
POLD3	0.47436603	3.25E-22	1.37E-20
POLD4	-0.089301897	0.085850443	0.12564077
POLDIP2	-0.260457538	3.62E-07	1.53E-06
POLDIP3	0.103739899	0.045844812	0.072244926
POLE2	0.323482875	1.75E-10	1.27E-09
POLE3	0.211256428	4.10E-05	0.000121655
POLE4	0.132120834	0.010852399	0.019844726
POLE	0.100697019	0.05262995	0.081671122
POLG2	0.100522299	0.053043679	0.082219827
POLG	-0.112545926	0.030207204	0.049763328
POLH	-0.035760939	0.49227203	0.566390031
POLI	-0.014006421	0.788017775	0.830189852
POLK	-0.040454419	0.437220263	0.513162734
POLL	-0.080486675	0.121727537	0.170610384
POLM	0.043305643	0.405577701	0.481807929
POLN	-0.194431048	0.000164302	0.00043574
POLQ	0.520718088	3.59E-27	3.30E-25
POLR1A	0.064474542	0.21535804	0.279940159
POLR1B	-0.034733886	0.504794142	0.578093177
POLR1C	0.028570494	0.583306377	0.651782087
POLR1D	-0.023865969	0.646805115	0.709992368
POLR1E	-0.06158795	0.236655294	0.303596895
POLR2A	0.003320276	0.949179327	0.960704698
POLR2B	0.149066735	0.004006703	0.008062782
POLR2C	-0.166672385	0.001272682	0.002847205
POLR2D	0.233679645	5.39E-06	1.88E-05
POLR2E	-0.090977096	0.080107932	0.118276901
POLR2F	-0.058068995	0.264569788	0.334142216
POLR2G	0.229840024	7.75E-06	2.61E-05
POLR2H	0.299556243	3.95E-09	2.33E-08
POLR2I	0.123364625	0.017442768	0.030335645
POLR2J2	0.038507874	0.459612286	0.534555197
POLR2J3	0.050943526	0.327794504	0.401890707
POLR2J4	-0.030898497	0.55299349	0.624142332
POLR2J	0.000282937	0.995666427	0.996566849
POLR2K	0.246457812	1.54E-06	5.87E-06
POLR2L	0.041550921	0.424887195	0.501279326
POLR3A	-0.016551209	0.750678548	0.798410017
POLR3B	0.151141805	0.003521098	0.00717106

POLR3C	0.304678007	2.08E-09	1.28E-08
POLR3D	0.184699013	0.000348404	0.000868481
POLR3E	0.025126374	0.629513893	0.694148214
POLR3F	0.204610681	7.19E-05	0.000203884
POLR3GL	-0.29700886	5.42E-09	3.13E-08
POLR3G	0.060557965	0.244602043	0.312369353
POLR3H	-0.087103262	0.093884973	0.135928519
POLR3K	0.262567712	2.89E-07	1.25E-06
POLRMT	-0.216270132	2.65E-05	8.11E-05
POM121C	0.051162667	0.325715296	0.399809003
POM121L10	0.252641723	8.22E-07	3.28E-06
POM121L1P	0.221349	1.69E-05	5.34E-05
POM121L2	0.256469506	5.52E-07	2.27E-06
POM121L4P	0.008828394	0.865421973	0.894285832
POM121L8P	0.148409693	0.004172634	0.008359534
POM121L9P	0.151488129	0.003445455	0.007033544
POM121	0.010475122	0.840628396	0.873695685
POMC	0.097468246	0.06072348	0.092586343
POMGNT1	-0.053396192	0.305011996	0.377606822
POMP	0.099431089	0.055689266	0.085863443
POMT1	-0.11898899	0.021888036	0.037291837
POMT2	0.207148509	5.82E-05	0.000167729
POMZP3	0.074074653	0.154469411	0.20961376
PON1	-0.4379792	8.06E-19	2.12E-17
PON2	0.101135518	0.051603427	0.080246934
PON3	-0.484875314	2.84E-23	1.39E-21
POP1	0.411439079	1.37E-16	2.59E-15
POP4	0.1530526	0.003121778	0.006442254
POP5	0.105368882	0.042524654	0.067509456
POP7	0.224991756	1.21E-05	3.95E-05
POPDC2	-0.006535039	0.900167051	0.921632644
POPDC3	0.228884653	8.47E-06	2.84E-05
PORCN	0.223875741	1.34E-05	4.33E-05
POR	-0.497024083	1.53E-24	9.05E-23
POSTN	0.337799308	2.36E-11	1.95E-10
POT1	-0.250556165	1.02E-06	4.01E-06
POTEA	0.050506034	0.331971019	0.406036754
POTEB	0.046387817	0.372952207	0.448831332
POTEC	0.011009987	0.832607839	0.867169248
POTED	0.045823308	0.378803386	0.454944977
POTEE	0.143450181	0.005639253	0.010966927
POTEF	0.172507852	0.000848187	0.00196575
POTEG	0.120011298	0.020769814	0.035556951
POTEH	0.124208268	0.016682889	0.029148966
POU1F1	0.082893586	0.110936104	0.157356288
POU2AF1	0.337423265	2.49E-11	2.05E-10
POU2F1	0.201487004	9.31E-05	0.000258503
POU2F2	0.199036887	0.000113634	0.000310325
POU2F3	-0.135411661	0.009015921	0.016806886
POU3F1	0.113369657	0.029011625	0.04799215
POU3F2	0.173791341	0.000774421	0.001808662
POU3F3	-0.007964864	0.878478046	0.904284973
POU3F4	0.080336123	0.122428796	0.17146056
POU4F1	-0.003459858	0.947045921	0.959081376
POU4F2	0.060087826	0.248290677	0.316329659
POU4F3	0.049259495	0.344057988	0.41835469
POU5F1B	0.151227814	0.003502173	0.007136895
POU5F1	0.209298001	4.85E-05	0.000141817

POU5F2	0.105011865	0.043234446	0.06850526
POU6F1	-0.11606465	0.025379329	0.042584604
POU6F2	0.021465294	0.680263894	0.738782016
PP14571	0.11472506	0.027132642	0.045221826
PPA1	0.225412049	1.17E-05	3.81E-05
PPA2	-0.164443534	0.001480996	0.003272086
PPAN-P2RY	-0.034079994	0.512852821	0.585538076
PPAN	0.190468805	0.000224126	0.000579065
PPAP2A	-0.196786577	0.000136208	0.000366694
PPAP2B	-0.355384159	1.74E-12	1.72E-11
PPAP2C	0.352358632	2.76E-12	2.65E-11
PPAPDC1A	0.296794526	5.57E-09	3.21E-08
PPAPDC1B	0.202290601	8.71E-05	0.000243423
PPAPDC2	-0.397778877	1.62E-15	2.57E-14
PPAPDC3	0.053641313	0.302794051	0.375397541
PPARA	-0.436833955	1.02E-18	2.62E-17
PPARD	0.290358168	1.22E-08	6.66E-08
PPARGC1A	-0.141189292	0.006450062	0.012407231
PPARGC1B	0.052021847	0.31764598	0.390915691
PPARG	0.029299574	0.573730418	0.643029951
PPAT	0.229206784	8.22E-06	2.76E-05
PPBPL2	0.094677903	0.068521001	0.103164705
PPBP	-0.147343092	0.004455251	0.008875416
PPCDC	0.308937133	1.20E-09	7.67E-09
PPCS	-0.092276486	0.075871136	0.112655942
PPDPF	0.080551647	0.121425871	0.170235483
PPEF1	0.027665967	0.595288773	0.662533125
PPEF2	0.023235394	0.655528422	0.717709234
PPFIA1	-0.029099884	0.576345825	0.645380334
PPFIA2	-0.066935927	0.198314171	0.260453221
PPFIA3	0.024159712	0.64275787	0.706367072
PPFIA4	0.339680937	1.80E-11	1.52E-10
PPFIBP1	0.007912474	0.879271247	0.904748813
PPFIBP2	-0.174218627	0.00075121	0.001758591
PPHLN1	0.446503008	1.40E-19	4.05E-18
PPIAL4C	0.289722718	1.32E-08	7.15E-08
PPIAL4D	0.163723929	0.001554661	0.003415831
PPIAL4E	0.111378503	0.031973694	0.052353081
PPIAL4G	0.270363142	1.23E-07	5.64E-07
PPIA	0.328148628	9.20E-11	6.98E-10
PPIB	0.145523103	0.004977608	0.009817237
PPIC	0.354632839	1.95E-12	1.92E-11
PPID	-0.199690391	0.000107768	0.000295766
PPIEL	0.129861461	0.012297962	0.022206109
PPIE	0.169777521	0.001027175	0.002340009
PPIF	-0.007011027	0.892937725	0.91549885
PPIG	-0.005755906	0.912018103	0.931134354
PPIH	0.33616693	2.98E-11	2.43E-10
PPIL1	0.183736828	0.000374529	0.000927222
PPIL2	0.011899323	0.819310568	0.856409315
PPIL3	-0.042742242	0.411719882	0.487911694
PPIL4	0.061276112	0.239041818	0.306214209
PPIL5	0.373279027	1.04E-13	1.24E-12
PPIL6	-0.087189726	0.093558044	0.135494574
PPIP5K1	0.129351517	0.01264682	0.022755596
PPIP5K2	0.026858426	0.606079933	0.672214922
PPL	-0.266649332	1.85E-07	8.27E-07
PPM1A	-0.39103382	5.28E-15	7.75E-14

PPM1B	-0.063146815	0.224976749	0.290585243
PPM1D	0.070601759	0.174784125	0.233256252
PPM1E	0.179125824	0.000527001	0.001270901
PPM1F	0.012925319	0.804034224	0.843228565
PPM1G	0.343020157	1.11E-11	9.68E-11
PPM1H	0.247388858	1.41E-06	5.38E-06
PPM1J	0.027524759	0.597169418	0.664255117
PPM1K	-0.26502778	2.21E-07	9.74E-07
PPM1L	-0.133852904	0.009848214	0.018215218
PPM1M	0.384782825	1.54E-14	2.09E-13
PPM1N	0.121854574	0.018878555	0.032588587
PPME1	0.352651162	2.64E-12	2.54E-11
PPOX	0.081461335	0.117263063	0.165154807
PPP1CA	0.269611636	1.34E-07	6.10E-07
PPP1CB	0.131692856	0.011114078	0.020272355
PPP1CC	0.411216043	1.43E-16	2.69E-15
PPP1R10	-0.216266955	2.65E-05	8.11E-05
PPP1R11	0.108623711	0.03649473	0.058860753
PPP1R12A	0.221954534	1.60E-05	5.08E-05
PPP1R12B	-0.022868291	0.660628617	0.72218192
PPP1R12C	0.090891637	0.080393159	0.118610059
PPP1R13B	0.154358538	0.00287301	0.005970179
PPP1R13L	0.175577115	0.000681622	0.001610064
PPP1R14A	-0.015974289	0.759096224	0.80561598
PPP1R14B	0.375305012	7.45E-14	9.15E-13
PPP1R14C	0.373919923	9.34E-14	1.13E-12
PPP1R14D	0.328489959	8.77E-11	6.68E-10
PPP1R15A	0.141356828	0.006386578	0.01229853
PPP1R15B	-0.059911484	0.249684173	0.317820465
PPP1R16A	-0.038903916	0.455005276	0.530342565
PPP1R16B	-0.041921396	0.420766415	0.496859019
PPP1R1A	0.023688899	0.649249876	0.712126632
PPP1R1B	0.24300081	2.18E-06	8.08E-06
PPP1R1C	-0.080962633	0.119531125	0.167898123
PPP1R2P1	0.028534587	0.583779894	0.652070048
PPP1R2P3	-0.058731455	0.259149089	0.328483053
PPP1R2P9	0.042797521	0.411114811	0.487281607
PPP1R2	-0.053376925	0.305186781	0.377729315
PPP1R3A	0.044436655	0.393413248	0.469738012
PPP1R3B	-0.247724184	1.36E-06	5.22E-06
PPP1R3C	-0.009814776	0.850553275	0.882078206
PPP1R3D	0.10417193	0.044943739	0.070987803
PPP1R3E	-0.170686833	0.000964024	0.002207504
PPP1R3F	-0.166125194	0.001321167	0.002945751
PPP1R3G	-0.252855481	8.04E-07	3.21E-06
PPP1R7	0.127832062	0.013738557	0.024510318
PPP1R8	0.000479864	0.99265027	0.994247294
PPP1R9A	0.291464836	1.07E-08	5.87E-08
PPP1R9B	0.324734785	1.47E-10	1.08E-09
PPP2CA	0.510229199	5.55E-26	4.32E-24
PPP2CB	-0.073272851	0.158993099	0.214917934
PPP2R1A	0.328167759	9.18E-11	6.96E-10
PPP2R1B	-0.123866689	0.016986993	0.029636122
PPP2R2A	0.068808247	0.186023046	0.246064521
PPP2R2B	0.335579973	3.24E-11	2.63E-10
PPP2R2C	0.340180398	1.67E-11	1.42E-10
PPP2R2D	-0.061857382	0.234606859	0.301285234
PPP2R3A	0.310174692	1.03E-09	6.62E-09

PPP2R3B	0.227834897	9.34E-06	3.10E-05
PPP2R3C	0.093907239	0.070813487	0.106047229
PPP2R4	0.038938583	0.454603249	0.529966992
PPP2R5A	-0.081151599	0.118667727	0.166833974
PPP2R5B	0.056278195	0.279610949	0.350296147
PPP2R5C	-0.256470151	5.52E-07	2.27E-06
PPP2R5D	0.127472832	0.014008474	0.024917573
PPP2R5E	0.073499388	0.157704996	0.213420215
PPP3CA	-0.087141219	0.09374134	0.135740295
PPP3CB	0.063731128	0.220706691	0.285922662
PPP3CC	0.04445573	0.393209999	0.469612564
PPP3R1	-0.219991015	1.91E-05	5.98E-05
PPP3R2	0.168166044	0.001148539	0.002592769
PPP4C	0.134311713	0.009596425	0.017789148
PPP4R1L	0.181402012	0.000445694	0.001088261
PPP4R1	0.356700013	1.42E-12	1.42E-11
PPP4R2	-0.249118702	1.18E-06	4.59E-06
PPP4R4	-0.29043298	1.21E-08	6.61E-08
PPP5C	0.340778595	1.53E-11	1.31E-10
PPP6C	0.003092095	0.952667675	0.963256632
PPPDE1	0.233716747	5.37E-06	1.87E-05
PPPDE2	-0.109568165	0.03488716	0.056551831
PPRC1	0.256294608	5.62E-07	2.30E-06
PPT1	0.447611999	1.11E-19	3.26E-18
PPT2	0.169192485	0.001069798	0.002428501
PPTC7	-0.154260508	0.002891038	0.006002008
PPWD1	0.142554399	0.005948858	0.011519552
PPY2	0.237010431	3.92E-06	1.39E-05
PPYR1	0.165446729	0.001383639	0.003075406
PPY	0.126776068	0.014545322	0.025771248
PQBP1	0.260644596	3.55E-07	1.51E-06
PQLC1	-0.374942646	7.91E-14	9.66E-13
PQLC2	0.205952845	6.43E-05	0.000183952
PQLC3	0.155453734	0.002678433	0.005612089
PRAC	0.099857165	0.05464352	0.084427502
PRAF2	0.357305089	1.30E-12	1.31E-11
PRAM1	0.230536999	7.26E-06	2.46E-05
PRAMEF10	-0.121217987	0.019514028	0.033583143
PRAMEF11	-0.056835546	0.274868889	0.345201917
PRAMEF12	0.076443212	0.141674816	0.194477068
PRAMEF13	0.093342554	0.072532706	0.108320583
PRAMEF14	-0.015604488	0.76450696	0.810262683
PRAMEF16	-0.185676312	0.000323614	0.00081105
PRAMEF17	-0.094626533	0.068671896	0.103318282
PRAMEF18	-0.176363221	0.000644131	0.001530031
PRAMEF1	-0.108970297	0.035897645	0.058001045
PRAMEF20	0.029868067	0.566315534	0.636079273
PRAMEF22	-0.090957674	0.080172683	0.118337422
PRAMEF2	-0.097060343	0.061815439	0.094071284
PRAMEF4	-0.041148145	0.429393773	0.505757524
PRAMEF5	-0.06535446	0.209148258	0.272829465
PRAMEF6	-0.03054169	0.557589158	0.628119379
PRAMEF8	0.036157378	0.487483296	0.561595812
PRAMEF9	-0.084844473	0.102753553	0.147227868
PRAME	0.371293607	1.43E-13	1.68E-12
PRAP1	-0.241216317	2.60E-06	9.54E-06
PRB1	0.01358159	0.794301379	0.835528384
PRB2	0.02617907	0.615225084	0.680577163

PRB3	0.124914282	0.016069384	0.028173393
PRB4	-0.025925549	0.618653275	0.683648244
PRC1	0.555230835	2.18E-31	4.09E-29
PRCC	0.306934025	1.56E-09	9.76E-09
PRCD	-0.035185919	0.499262308	0.573108828
PRCP	-0.348078745	5.25E-12	4.81E-11
PRDM10	0.135933821	0.008751445	0.016350585
PRDM11	0.088837805	0.08749862	0.127740548
PRDM12	-0.175442003	0.000688266	0.00162441
PRDM13	0.213537765	3.37E-05	0.000101293
PRDM14	0.044923424	0.388246337	0.464598962
PRDM15	0.329084955	8.08E-11	6.19E-10
PRDM16	0.231301181	6.75E-06	2.30E-05
PRDM1	0.13758524	0.007959857	0.015015271
PRDM2	0.060757595	0.243047422	0.310622962
PRDM4	0.45131147	5.12E-20	1.55E-18
PRDM5	0.138411887	0.007588127	0.014371202
PRDM6	0.115957183	0.025516291	0.042799995
PRDM7	-0.022952077	0.659463184	0.721237248
PRDM8	0.193233269	0.000180589	0.000475571
PRDM9	-0.097436694	0.060807373	0.092700067
PRDX1	0.126561535	0.014714215	0.026045011
PRDX2	0.033491919	0.520156962	0.592451375
PRDX3	-0.252636023	8.23E-07	3.28E-06
PRDX4	0.032572025	0.531688634	0.603618701
PRDX5	0.131220348	0.011409455	0.020750334
PRDX6	-0.115304565	0.026361777	0.044074034
PRDXDD1P	0.02462854	0.636320118	0.700490103
PREB	-0.280211573	4.03E-08	2.01E-07
PRELID1	0.293078731	8.77E-09	4.91E-08
PRELID2	0.272858342	9.32E-08	4.35E-07
PRELP	0.104030678	0.045236693	0.071399572
PREPL	-0.086254805	0.097141712	0.140093903
PREP	0.092506207	0.075141398	0.111673411
PREX1	0.239310006	3.13E-06	1.14E-05
PREX2	-0.152704636	0.003191283	0.006564454
PRF1	-0.03763727	0.469830539	0.544627254
PRG1	0.227677796	9.47E-06	3.14E-05
PRG2	-0.05953384	0.252686687	0.321211343
PRG3	0.018402615	0.723868653	0.776027084
PRG4	0.034869124	0.503135769	0.576459098
PRH1	0.041313454	0.427540833	0.50393258
PRH2	0.056907749	0.274258598	0.344565793
PRHOXNB	0.078394722	0.131755831	0.182610246
PRIC285	-0.101619025	0.05049094	0.078713554
PRICKLE1	0.096513258	0.063305353	0.096133032
PRICKLE2	0.17488398	0.000716346	0.001684097
PRICKLE3	0.089691864	0.084484908	0.123849032
PRICKLE4	-0.022315028	0.668344839	0.728777552
PRIM1	0.269405747	1.37E-07	6.23E-07
PRIM2	0.401631727	8.19E-16	1.36E-14
PRIMA1	0.126981621	0.014385098	0.02552141
PRINS	0.086223751	0.097262592	0.140237776
PRKAA1	0.076296683	0.142442042	0.195382151
PRKAA2	0.006512012	0.900517001	0.92184881
PRKAB1	0.088771732	0.087735327	0.128039204
PRKAB2	-0.219836243	1.93E-05	6.05E-05
PRKACA	-0.04630856	0.373770327	0.449707239

PRKACB	-0.079344814	0.127125006	0.177091417
PRKACG	0.041159922	0.429261611	0.50566165
PRKAG1	0.193047346	0.000183249	0.00048181
PRKAG2	-0.351456201	3.16E-12	3.00E-11
PRKAG3	0.084463845	0.104311306	0.149180892
PRKAR1A	-0.175992835	0.000661551	0.001567858
PRKAR1B	0.122487211	0.018264958	0.031633008
PRKAR2A	-0.11281206	0.029816402	0.049168311
PRKAR2B	0.257310014	5.05E-07	2.09E-06
PRKCA	-0.016780564	0.747340215	0.795847632
PRKCB	0.136309785	0.008565316	0.0160314
PRKCDBP	0.182743708	0.000403398	0.000992649
PRKCD	0.554997045	2.34E-31	4.31E-29
PRKCE	-0.035935074	0.490165501	0.564324845
PRKCG	0.110746072	0.032966966	0.053789345
PRKCH	-0.082477331	0.112746594	0.159583492
PRKCI	0.377357052	5.32E-14	6.68E-13
PRKCQ	0.185551454	0.000326687	0.000818134
PRKCSH	0.031816516	0.541255148	0.612805636
PRKCZ	-0.046440273	0.372411335	0.44823457
PRKD1	-0.022231561	0.669511997	0.729930389
PRKD2	0.261449171	3.26E-07	1.39E-06
PRKD3	0.070817125	0.173469242	0.231780968
PRKDC	0.365177849	3.80E-13	4.17E-12
PRKG1	0.011662948	0.822839937	0.859196877
PRKG2	0.057213562	0.271683946	0.341632745
PRKRA	0.318032603	3.64E-10	2.51E-09
PRKRIP1	0.172743069	0.000834201	0.001935586
PRKRIR	0.089534319	0.085034454	0.124599617
PRKX	0.376834609	5.80E-14	7.25E-13
PRKY	0.083757655	0.10725088	0.15280355
PRLHR	0.079482409	0.12646494	0.176319864
PRLH	0.085120946	0.10163366	0.145843533
PRLR	-0.017648763	0.7347465	0.784943145
PRL	0.103454478	0.046448383	0.073109323
PRM1	0.078863117	0.12945682	0.17986183
PRM2	0.201868293	9.02E-05	0.000251139
PRMT10	-0.227695909	9.46E-06	3.14E-05
PRMT1	0.388349002	8.38E-15	1.19E-13
PRMT2	0.431711982	2.83E-18	6.79E-17
PRMT3	0.392108064	4.39E-15	6.54E-14
PRMT5	0.02141436	0.680980895	0.739399466
PRMT6	0.112626412	0.030088555	0.049584266
PRMT7	-0.112172078	0.030763575	0.050537803
PRMT8	0.090628369	0.081277007	0.119719079
PRND	0.155281997	0.002708129	0.0056624
PRNP	0.217755591	2.33E-05	7.18E-05
PRO0611	0.043540449	0.403034033	0.479214802
PRO0628	0.12490256	0.016079407	0.028188485
PRO1768	0.091498683	0.078384796	0.116016486
PROCA1	0.154261918	0.002890778	0.006002008
PROCR	0.176913555	0.00061903	0.001473572
PROC	-0.465513014	2.37E-21	8.84E-20
PRODH2	-0.356610252	1.44E-12	1.44E-11
PRODH	-0.015590891	0.764706119	0.810430647
PROK1	-0.269854478	1.30E-07	5.95E-07
PROK2	0.109481253	0.035032535	0.056751782
PROKR1	0.117140301	0.024043007	0.040578176

PROKR2	0.072897969	0.161142089	0.217403843
PROL1	-0.2558053	5.92E-07	2.42E-06
PROM1	0.333225461	4.52E-11	3.60E-10
PROM2	0.309096427	1.18E-09	7.52E-09
PROP1	0.073192506	0.159451843	0.215435715
PROS1	-0.397668851	1.66E-15	2.62E-14
PROSC	-0.122605811	0.018151883	0.03144911
PROX1	-0.22805659	9.15E-06	3.04E-05
PROX2	0.007369882	0.887493255	0.911420209
PROZ	-0.371193235	1.45E-13	1.71E-12
PRPF18	0.064658685	0.214047676	0.278418503
PRPF19	0.162684266	0.001667035	0.003636736
PRPF31	0.195188948	0.000154719	0.000412405
PRPF38A	0.257183204	5.12E-07	2.12E-06
PRPF38B	0.31124514	8.92E-10	5.81E-09
PRPF39	0.351282034	3.25E-12	3.08E-11
PRPF3	0.320053913	2.78E-10	1.95E-09
PRPF40A	0.174209462	0.000751701	0.001759327
PRPF40B	0.310171756	1.03E-09	6.62E-09
PRPF4B	0.146457469	0.004702917	0.009328107
PRPF4	0.220172707	1.88E-05	5.89E-05
PRPF6	0.243556684	2.06E-06	7.68E-06
PRPF8	-0.088601962	0.088345886	0.128798151
PRPH2	0.268076579	1.59E-07	7.14E-07
PRPH	0.299734074	3.87E-09	2.28E-08
PRPS1L1	0.009308865	0.858173097	0.888731322
PRPS1	-0.105793052	0.041694137	0.066381411
PRPS2	0.08386381	0.106804871	0.152255216
PRPSAP1	-0.147870608	0.004313384	0.008625037
PRPSAP2	-0.02893815	0.578468193	0.647547249
PRR11	0.392022267	4.45E-15	6.63E-14
PRR12	0.228210617	9.02E-06	3.00E-05
PRR13	0.299405075	4.03E-09	2.37E-08
PRR14	0.240347885	2.83E-06	1.03E-05
PRR15L	0.288819588	1.47E-08	7.89E-08
PRR15	0.158150653	0.002249356	0.004790642
PRR16	0.203805659	7.69E-05	0.000216775
PRR18	-0.236969726	3.93E-06	1.40E-05
PRR19	0.392941083	3.80E-15	5.70E-14
PRR22	0.014381711	0.782478304	0.825576357
PRR23A	0.079724823	0.125308521	0.17495244
PRR23B	0.016912365	0.745423936	0.794178066
PRR23C	0.064459769	0.215463414	0.280040588
PRR24	0.183952875	0.000368508	0.000913795
PRR25	0.066626249	0.200402826	0.262936322
PRR3	0.182129008	0.00042229	0.00103441
PRR4	0.072354003	0.164299105	0.221130034
PRR5-ARHC	0.064309037	0.21654068	0.281275571
PRR5L	-0.137454355	0.008020181	0.015114752
PRR5	-0.111896857	0.031178746	0.051146159
PRR7	0.393091292	3.70E-15	5.57E-14
PRRC1	0.222764726	1.49E-05	4.76E-05
PRRG1	-0.264435909	2.36E-07	1.03E-06
PRRG2	0.222449203	1.53E-05	4.88E-05
PRRG3	0.025852972	0.619636203	0.684544581
PRRG4	-0.04502359	0.387188237	0.463471974
PRRT1	0.076114908	0.143398224	0.196517811
PRRT2	0.277264035	5.66E-08	2.75E-07

PRRT3	0.047654969	0.36002251	0.435533668
PRRT4	-0.021472842	0.68015766	0.738747186
PRRX1	0.259942894	3.82E-07	1.61E-06
PRRX2	0.403411911	5.95E-16	1.02E-14
PRSS12	0.280640562	3.84E-08	1.92E-07
PRSS16	0.358417125	1.09E-12	1.12E-11
PRSS1	0.191208187	0.000211608	0.000549744
PRSS21	0.337665495	2.40E-11	1.99E-10
PRSS22	0.447135796	1.23E-19	3.58E-18
PRSS23	0.163543974	0.0015736	0.003452942
PRSS27	0.183909413	0.000369712	0.000916095
PRSS30P	0.218680023	2.14E-05	6.66E-05
PRSS33	0.152049711	0.003325923	0.006811869
PRSS35	0.279154009	4.56E-08	2.25E-07
PRSS36	-0.213030044	3.52E-05	0.000105532
PRSS37	0.071401063	0.169941172	0.22778847
PRSS38	0.062414095	0.230413974	0.296625229
PRSS3	0.334313803	3.88E-11	3.11E-10
PRSS41	0.17969453	0.000505487	0.001222719
PRSS42	-0.006124134	0.906414559	0.926479639
PRSS45	-0.005033105	0.923029826	0.939776163
PRSS48	-0.046376084	0.373073253	0.448949885
PRSS50	-0.019700771	0.705267275	0.76021722
PRSS53	-0.126447774	0.014804471	0.026167569
PRSS54	0.020827123	0.68926819	0.746363784
PRSS55	0.058273667	0.262886777	0.332438135
PRSS8	0.15354196	0.003026355	0.006258803
PRSSL1	-0.042020662	0.419666275	0.495736231
PRTFDC1	0.233392541	5.54E-06	1.93E-05
PRTG	0.145618445	0.004948931	0.009763578
PRTN3	0.095437677	0.06632074	0.100124415
PRUNE2	0.287058285	1.81E-08	9.58E-08
PRUNE	0.018621181	0.720725057	0.773531116
PRX	-0.007871232	0.879895758	0.905192917
PRY2	0.021940209	0.673592306	0.733015017
PSAPL1	0.376848077	5.78E-14	7.24E-13
PSAP	0.037212164	0.47486506	0.549631752
PSAT1	0.001994657	0.969456314	0.975922622
PSCA	0.250114423	1.07E-06	4.18E-06
PSD2	-0.063458614	0.222690918	0.288174632
PSD3	-0.299461874	4.00E-09	2.35E-08
PSD4	-0.28094557	3.70E-08	1.86E-07
PSD	-0.065792168	0.206107817	0.269374791
PSEN1	-0.008998044	0.862861135	0.89219492
PSEN2	0.186850167	0.000296013	0.000746923
PSENEN	0.199478024	0.000109643	0.000300702
PSG10	0.089748098	0.084289449	0.12361708
PSG11	-0.093338877	0.072544013	0.108329347
PSG1	0.126246652	0.014965227	0.026430608
PSG2	0.109205084	0.035497895	0.057439008
PSG3	0.002290796	0.96492432	0.972490631
PSG4	-0.041679037	0.42345951	0.499772533
PSG5	4.59E-05	0.999296877	0.999397208
PSG6	0.010772382	0.836168743	0.870284901
PSG7	-0.083095873	0.110064552	0.156253546
PSG8	-0.098578701	0.057831203	0.088704022
PSG9	-0.021687636	0.677137301	0.735867844
PSIMCT-1	-0.04669603	0.369781206	0.445688256

PSIP1	0.253187399	7.77E-07	3.11E-06
PSKH1	-0.185933945	0.000317358	0.000796775
PSKH2	0.07074113	0.173932372	0.232337449
PSMA1	0.31904797	3.18E-10	2.21E-09
PSMA2	-0.152760801	0.00317997	0.006549106
PSMA3	0.155232276	0.002716783	0.005679522
PSMA4	0.254871082	6.52E-07	2.65E-06
PSMA5	0.127567543	0.013936861	0.024807912
PSMA6	0.166377924	0.001298566	0.002899252
PSMA7	0.136729506	0.008361694	0.015685657
PSMA8	0.16677845	0.001263475	0.002828514
PSMB10	0.145690286	0.004927421	0.009723068
PSMB11	-0.08129715	0.118006023	0.166048593
PSMB1	0.07203969	0.166144311	0.223236458
PSMB2	0.114415701	0.02755203	0.045834966
PSMB3	0.193564989	0.000175933	0.000463801
PSMB4	0.19618086	0.000142967	0.000383388
PSMB5	0.099344499	0.055903808	0.086167557
PSMB6	0.029803994	0.567148946	0.636799938
PSMB7	0.002175957	0.966681647	0.97390886
PSMB8	0.175238162	0.000698403	0.001645994
PSMB9	0.264047004	2.46E-07	1.07E-06
PSMC1	0.030426032	0.559082793	0.629552758
PSMC2	-0.098862643	0.057110246	0.08775552
PSMC3IP	0.49122773	6.25E-24	3.38E-22
PSMC3	0.089653458	0.084618612	0.12402678
PSMC4	0.292119842	9.86E-09	5.45E-08
PSMC5	0.053380333	0.305155864	0.377714516
PSMC6	0.094529763	0.068956891	0.103648648
PSMD10	0.226780934	1.03E-05	3.39E-05
PSMD11	0.169102012	0.001076533	0.002443511
PSMD12	0.103633774	0.04606846	0.072557187
PSMD13	0.209964975	4.58E-05	0.000134674
PSMD14	0.388852787	7.69E-15	1.10E-13
PSMD1	0.121051664	0.019683095	0.033841945
PSMD2	0.243686929	2.04E-06	7.59E-06
PSMD3	0.097635751	0.060279703	0.092050609
PSMD4	0.02207517	0.671701013	0.731636281
PSMD5	0.0190168	0.715046911	0.76859634
PSMD6	0.120686361	0.020058904	0.034422731
PSMD7	-0.015483266	0.766283121	0.811583856
PSMD8	0.106335791	0.040651446	0.064902878
PSMD9	0.128986244	0.012902042	0.023168783
PSME1	-0.006519529	0.900402769	0.921789235
PSME2	0.02252014	0.665480066	0.726130676
PSME3	0.36912841	2.03E-13	2.32E-12
PSME4	-0.00151209	0.976843346	0.981820955
PSMF1	-0.129367402	0.012635823	0.022739915
PSMG1	0.341772496	1.33E-11	1.14E-10
PSMG2	0.154155708	0.002910425	0.006037856
PSMG3	0.332478296	5.02E-11	3.96E-10
PSMG4	0.010531282	0.839785451	0.873184016
PSORS1C1	0.332431001	5.06E-11	3.98E-10
PSORS1C2	0.31676822	4.31E-10	2.94E-09
PSORS1C3	0.067514515	0.194454392	0.25595774
PSPC1	0.386037827	1.24E-14	1.71E-13
PSPH	0.39644468	2.06E-15	3.20E-14
PSPN	0.01774667	0.733330689	0.783850949

PSRC1	0.390457515	5.84E-15	8.49E-14
PSTK	-0.131162452	0.01144612	0.020809418
PSTPIP1	0.264481146	2.35E-07	1.03E-06
PSTPIP2	-0.01470401	0.77772976	0.821392795
PTAFR	0.182578846	0.000408386	0.001003436
PTAR1	-0.163257395	0.001604199	0.00351119
PTBP1	0.354877554	1.88E-12	1.86E-11
PTBP2	0.285318097	2.22E-08	1.16E-07
PTCD1	-0.023169555	0.656441961	0.718590953
PTCD2	-0.134885231	0.009289733	0.017273666
PTCD3	-0.077139044	0.138074537	0.190203355
PTCH1	-0.040708218	0.434347503	0.510445432
PTCH2	0.132385577	0.01069327	0.019583685
PTCHD1	0.250463913	1.03E-06	4.04E-06
PTCHD2	0.212988489	3.53E-05	0.000105881
PTCHD3	0.184612839	0.000350672	0.000873262
PTCRA	0.089924707	0.083677953	0.122883103
PTDSS1	0.138571398	0.007518208	0.014249619
PTDSS2	0.37417035	8.97E-14	1.09E-12
PTENP1	-0.123327282	0.01747709	0.030390032
PTEN	-0.254284059	6.94E-07	2.80E-06
PTER	0.163288072	0.001600897	0.003505504
PTF1A	0.133221449	0.010204331	0.018793629
PTGDR	-0.05944002	0.253436479	0.322082262
PTGDS	0.289345396	1.38E-08	7.46E-08
PTGER1	-0.039749529	0.445255821	0.520898847
PTGER2	0.112297181	0.030576425	0.050284261
PTGER3	-0.159349882	0.002079528	0.00446234
PTGER4	0.272359059	9.85E-08	4.58E-07
PTGES2	0.187097277	0.00029049	0.000734596
PTGES3	0.326680423	1.13E-10	8.42E-10
PTGES	0.394910194	2.69E-15	4.14E-14
PTGFRN	0.30607775	1.74E-09	1.08E-08
PTGFR	0.191119303	0.000213077	0.000553085
PTGIR	0.175933243	0.000664394	0.001573661
PTGIS	0.194428964	0.000164329	0.000435747
PTGR1	-0.183095018	0.000392957	0.000969111
PTGR2	-0.258543971	4.44E-07	1.85E-06
PTGS1	0.28862523	1.50E-08	8.06E-08
PTGS2	0.174280054	0.000747927	0.00175214
PTH1R	-0.045430843	0.382904293	0.459171693
PTH2R	-0.052161779	0.316344225	0.389578393
PTH2	0.016009086	0.758587702	0.805231469
PTHLH	0.38647241	1.16E-14	1.59E-13
PTH	0.098896335	0.057025193	0.087645108
PTK2B	-0.194013128	0.000169822	0.000449175
PTK2	0.179080699	0.000528744	0.001274641
PTK6	0.094544434	0.068913623	0.103607063
PTK7	0.335264521	3.39E-11	2.74E-10
PTMA	0.428725528	5.09E-18	1.18E-16
PTMS	-0.256945607	5.25E-07	2.16E-06
PTN	0.027900475	0.59217148	0.659764008
PTOV1	0.145257774	0.005058203	0.009949597
PTP4A1	-0.122649044	0.018110817	0.031387881
PTP4A2	0.235521277	4.52E-06	1.59E-05
PTP4A3	0.378728781	4.24E-14	5.39E-13
PTPDC1	0.367733049	2.53E-13	2.87E-12
PTPLAD1	-0.149864098	0.003813362	0.007708758

PTPLAD2	0.281136002	3.62E-08	1.83E-07
PTPLA	0.277958123	5.23E-08	2.55E-07
PTPLB	-0.028631004	0.582508803	0.651037328
PTPMT1	0.204768482	7.10E-05	0.000201396
PTPN11	-0.024416681	0.639225924	0.703183813
PTPN12	0.220270792	1.86E-05	5.84E-05
PTPN13	0.2705984	1.20E-07	5.51E-07
PTPN14	0.246027401	1.61E-06	6.11E-06
PTPN18	0.006949457	0.893872375	0.916223783
PTPN1	0.192221756	0.000195508	0.000511076
PTPN20B	0.142111909	0.006107353	0.011800085
PTPN21	-0.272889763	9.29E-08	4.34E-07
PTPN22	0.294769149	7.14E-09	4.05E-08
PTPN23	0.263168411	2.71E-07	1.17E-06
PTPN2	0.369969539	1.77E-13	2.05E-12
PTPN3	-0.235282154	4.63E-06	1.63E-05
PTPN4	-0.217306928	2.42E-05	7.44E-05
PTPN5	0.070611245	0.17472605	0.233209993
PTPN6	0.30047302	3.53E-09	2.09E-08
PTPN7	0.317067883	4.14E-10	2.84E-09
PTPN9	-0.031692788	0.542829954	0.614271987
PTPRA	0.103806163	0.045705633	0.072048395
PTPRB	-0.18161853	0.000438601	0.001072255
PTPRCAP	0.216292486	2.65E-05	8.09E-05
PTPRC	0.15575275	0.002627435	0.005518582
PTPRD	-0.090755643	0.080848746	0.119141114
PTPRE	0.348664475	4.81E-12	4.44E-11
PTPRF	-0.080381983	0.122214848	0.171197033
PTPRG	-0.177226709	0.000605155	0.001443475
PTPRH	-0.007585241	0.884228384	0.908535658
PTPRJ	-0.02748293	0.597727033	0.664726914
PTPRK	0.000489734	0.992499105	0.994195855
PTPRM	0.012038086	0.817240351	0.854514369
PTPRN2	-0.055983515	0.282140402	0.352932381
PTPRN	0.132086788	0.010873014	0.019878148
PTPRO	0.319205709	3.11E-10	2.17E-09
PTPRQ	0.054874599	0.291797225	0.363483044
PTPRR	0.04683723	0.368334086	0.444212825
PTPRS	0.180670082	0.000470471	0.001143853
PTPRT	0.111745962	0.031408397	0.051495193
PTPRU	-0.015674315	0.76348441	0.809489774
PTPRVP	0.01706623	0.743188869	0.792220301
PTPRZ1	0.205091623	6.91E-05	0.000196493
PTRF	0.028349477	0.58622386	0.65426061
PTRH1	-0.03023063	0.561610646	0.632006286
PTRH2	0.194730351	0.000160453	0.00042632
PTS	-0.069112678	0.18407876	0.243798501
PTTG1IP	0.225487236	1.16E-05	3.79E-05
PTTG1	0.596954977	3.43E-37	2.97E-34
PTTG2	0.326163823	1.21E-10	8.99E-10
PTTG3P	0.501179833	5.46E-25	3.57E-23
PTX3	0.089297693	0.085865258	0.125650629
PTX4	0.023032875	0.658340071	0.720233437
PUF60	0.210066	4.54E-05	0.000133734
PUM1	-0.083127706	0.109927893	0.156070659
PUM2	-0.01605901	0.757858284	0.804500065
PURA	-0.040515048	0.43653301	0.512650945
PURB	0.144950423	0.005153029	0.010117646

PURG	0.145353953	0.005028853	0.009898707
PUS10	-0.337951206	2.31E-11	1.92E-10
PUS1	0.301013596	3.30E-09	1.97E-08
PUS3	-0.260938578	3.44E-07	1.46E-06
PUS7L	0.114412985	0.027555737	0.045834966
PUS7	0.189501567	0.000241548	0.00062012
PUSL1	0.25670732	5.38E-07	2.21E-06
PVALB	0.15137172	0.003470715	0.007080755
PVRIG	0.130008099	0.01219923	0.022043815
PVRL1	0.401505769	8.37E-16	1.39E-14
PVRL2	0.234720317	4.88E-06	1.71E-05
PVRL3	-0.093066288	0.07338613	0.109422834
PVRL4	0.168641655	0.001111413	0.002516087
PVR	-0.019187408	0.712603066	0.766382977
PVT1	0.257112955	5.16E-07	2.13E-06
PWP1	0.273062705	9.11E-08	4.26E-07
PWP2	0.276055229	6.49E-08	3.12E-07
PWRN1	-0.00346733	0.946931726	0.959050018
PWRN2	0.004797384	0.926624272	0.942713142
PWWP2A	0.298329205	4.61E-09	2.68E-08
PWWP2B	0.385370037	1.39E-14	1.90E-13
PXDNL	-0.093468297	0.072146958	0.107801072
PXDN	0.170539597	0.000974	0.002228556
PXK	0.032712788	0.529915718	0.601914644
PXMP2	-0.395775031	2.31E-15	3.58E-14
PXMP4	0.017926532	0.730732077	0.781870371
PXN	0.225383862	1.17E-05	3.82E-05
PXT1	0.009166057	0.860326427	0.890359641
PYCARD	0.477988546	1.42E-22	6.30E-21
PYCR1	0.255803271	5.92E-07	2.42E-06
PYCR2	0.262217934	3.00E-07	1.29E-06
PYCRL	0.050373895	0.333239185	0.407312783
PYDC1	0.20502181	6.95E-05	0.000197471
PYDC2	-0.009337065	0.857748013	0.888337279
PYGB	0.320534816	2.60E-10	1.84E-09
PYGL	-0.201879109	9.01E-05	0.000250951
PYGM	0.006518886	0.900412534	0.921789235
PYGO1	0.157914415	0.002284266	0.004857205
PYGO2	0.284499434	2.45E-08	1.27E-07
PYHIN1	0.094125449	0.070158111	0.105216041
PYROXD1	-0.056240602	0.27993278	0.350655234
PYROXD2	-0.036284039	0.48595862	0.560064076
PYY2	0.199416254	0.000110193	0.000302047
PYY	-0.156280273	0.002539614	0.005355583
PZP	0.063092268	0.225378353	0.290953056
ProSAPiP1	0.008179197	0.875234246	0.901712605
QARS	0.0604514	0.245434752	0.313231975
QDPR	-0.445022107	1.91E-19	5.40E-18
QKI	0.073233493	0.159217695	0.215192329
QPCTL	-0.086330494	0.096847577	0.139740525
QPCT	0.352775476	2.59E-12	2.50E-11
QPRT	-0.213814503	3.29E-05	9.90E-05
QRFPR	-0.125196982	0.015829321	0.02780389
QRFP	0.152927186	0.003146669	0.006486749
QRICH1	0.074802126	0.150449959	0.204927122
QRICH2	0.090916787	0.080309133	0.118494931
QRSL1	0.18602522	0.000315169	0.000791778
QSER1	0.274827753	7.46E-08	3.55E-07

QSOX1	0.299924484	3.78E-09	2.23E-08
QSOX2	0.100976566	0.051973587	0.080734332
QTRT1	-0.131592231	0.011176408	0.020376719
QTRTD1	0.237099222	3.88E-06	1.38E-05
R3HCC1	-0.025012051	0.63107414	0.695637637
R3HDM1	0.25368483	7.38E-07	2.97E-06
R3HDM2	-0.216723064	2.55E-05	7.81E-05
R3HDML	-0.173882501	0.000769414	0.001798037
RAB10	0.38348108	1.92E-14	2.56E-13
RAB11A	0.337543682	2.45E-11	2.02E-10
RAB11B	-0.115744507	0.025789216	0.043217762
RAB11FIP1	0.285861394	2.08E-08	1.09E-07
RAB11FIP2	-0.182975319	0.000396485	0.000976968
RAB11FIP3	-0.146453205	0.004704139	0.009329602
RAB11FIP4	0.309903424	1.06E-09	6.83E-09
RAB11FIP5	0.259467347	4.02E-07	1.69E-06
RAB12	0.010585451	0.838972577	0.872590908
RAB13	0.030626903	0.556489936	0.62707377
RAB14	-0.112998178	0.02954568	0.048775085
RAB15	0.149158871	0.003983918	0.008022604
RAB17	-0.252829528	8.06E-07	3.22E-06
RAB18	-0.024921113	0.632316405	0.696686161
RAB19	0.267191585	1.75E-07	7.82E-07
RAB1A	0.298644136	4.43E-09	2.59E-08
RAB1B	-0.024011905	0.644793065	0.70813492
RAB20	0.195296202	0.000153406	0.000409178
RAB21	0.022485362	0.665965469	0.726461374
RAB22A	0.208195248	5.32E-05	0.000154527
RAB23	0.135174075	0.009138596	0.017022824
RAB24	0.289552193	1.34E-08	7.28E-08
RAB25	0.227764222	9.40E-06	3.12E-05
RAB26	-0.102076806	0.049456162	0.077299989
RAB27A	0.205286756	6.80E-05	0.000193541
RAB27B	0.246078052	1.60E-06	6.08E-06
RAB28	0.202008698	8.92E-05	0.000248468
RAB2A	0.035179673	0.499338526	0.573130271
RAB2B	-0.116503894	0.024826099	0.0417689
RAB30	-0.212376857	3.72E-05	0.000111346
RAB31	0.315496692	5.10E-10	3.44E-09
RAB32	0.152958633	0.003140411	0.006475315
RAB33A	0.274266305	7.95E-08	3.76E-07
RAB33B	-0.206633784	6.07E-05	0.000174594
RAB34	0.352527574	2.69E-12	2.59E-11
RAB35	0.42376565	1.34E-17	2.92E-16
RAB36	0.337746173	2.38E-11	1.97E-10
RAB37	-0.270587729	1.20E-07	5.51E-07
RAB38	0.271480389	1.09E-07	5.02E-07
RAB39B	0.239230347	3.16E-06	1.14E-05
RAB39	0.248406445	1.27E-06	4.90E-06
RAB3A	0.060945236	0.241592479	0.308961701
RAB3B	0.251339843	9.40E-07	3.72E-06
RAB3C	0.15153443	0.003435455	0.007015286
RAB3D	0.29408727	7.76E-09	4.37E-08
RAB3GAP1	0.027427589	0.598465116	0.665436292
RAB3GAP2	0.086897522	0.094666568	0.13691088
RAB3IL1	0.362478201	5.82E-13	6.19E-12
RAB3IP	0.27190568	1.04E-07	4.80E-07
RAB40AL	0.199039254	0.000113612	0.000310308

RAB40A	0.208812587	5.05E-05	0.000147271
RAB40B	0.05581902	0.283559082	0.354462197
RAB40C	0.014962396	0.773928824	0.818028971
RAB41	0.085868836	0.09865269	0.142026224
RAB42	0.405637541	3.98E-16	6.97E-15
RAB43	-0.166328222	0.001302982	0.002908013
RAB4A	-0.232839751	5.84E-06	2.02E-05
RAB4B	0.124511536	0.016416892	0.028727038
RAB5A	-0.007701448	0.882467474	0.907406273
RAB5B	-0.178099372	0.000567996	0.001361852
RAB5C	0.076686078	0.14041014	0.192993709
RAB6A	0.114772904	0.027068273	0.045118318
RAB6B	0.404990543	4.47E-16	7.72E-15
RAB6C	0.160664182	0.001906921	0.004120355
RAB7A	0.130272509	0.012022968	0.021757338
RAB7L1	0.063971428	0.218967526	0.283909603
RAB8A	-0.078708103	0.130214212	0.180750245
RAB8B	0.112715483	0.029957717	0.049380906
RAB9A	0.224781538	1.24E-05	4.02E-05
RAB9BP1	0.02999198	0.564705427	0.634736037
RAB9B	0.246534923	1.53E-06	5.83E-06
RABAC1	0.075879847	0.144641964	0.197909149
RABEP1	-0.090283822	0.082445575	0.121243135
RABEP2	0.342610261	1.17E-11	1.02E-10
RABEPK	-0.325260591	1.37E-10	1.01E-09
RABGAP1L	0.097009337	0.061953115	0.094259199
RABGAP1	-0.005193381	0.92058671	0.937934667
RABGEF1	-0.080004117	0.123986357	0.173361609
RABGGTA	-0.050352273	0.333447	0.407516786
RABGGTB	0.040225774	0.439817604	0.515475132
RABIF	0.335408595	3.32E-11	2.69E-10
RABL2A	-0.005925398	0.909438286	0.928833668
RABL2B	-0.003745034	0.942688433	0.955785992
RABL3	-0.17201681	0.000878087	0.002027262
RABL5	0.036694465	0.481035787	0.555290008
RAC1	0.287699979	1.68E-08	8.92E-08
RAC2	0.340762888	1.54E-11	1.31E-10
RAC3	-0.210947746	4.21E-05	0.000124679
RACGAP1P	0.279319112	4.47E-08	2.21E-07
RACGAP1	0.491569004	5.75E-24	3.14E-22
RAD17	-0.062889167	0.226878154	0.292529065
RAD18	0.28878904	1.47E-08	7.92E-08
RAD1	0.374843213	8.04E-14	9.80E-13
RAD21L1	0.102190197	0.049202606	0.076970107
RAD21	0.416623892	5.21E-17	1.05E-15
RAD23A	0.039276387	0.450696208	0.52645968
RAD23B	-0.172059012	0.000875479	0.002021945
RAD50	0.014687562	0.777971897	0.82151787
RAD51AP1	0.477341481	1.64E-22	7.26E-21
RAD51AP2	-0.16426872	0.001498591	0.003305827
RAD51C	0.281062129	3.65E-08	1.84E-07
RAD51L1	0.064779572	0.213190562	0.277394186
RAD51L3	0.297001993	5.43E-09	3.13E-08
RAD51	0.548859356	1.42E-30	2.20E-28
RAD52	0.073969848	0.155055113	0.210279644
RAD54B	0.410747057	1.56E-16	2.91E-15
RAD54L2	-0.319953297	2.82E-10	1.98E-09
RAD54L	0.520851096	3.47E-27	3.23E-25

RAD9A	0.335244645	3.40E-11	2.75E-10
RAD9B	0.152687563	0.003194729	0.006570186
RADIL	0.277482244	5.52E-08	2.68E-07
RAE1	0.358091486	1.15E-12	1.17E-11
RAET1E	0.070464045	0.175628786	0.234242262
RAET1G	0.045111847	0.386257391	0.462524478
RAET1K	0.282560935	3.07E-08	1.57E-07
RAET1L	0.16020896	0.001965163	0.004233346
RAF1	0.040239403	0.439662526	0.515390787
RAG1AP1	0.218901424	2.10E-05	6.54E-05
RAG1	-0.29617429	6.01E-09	3.44E-08
RAG2	-0.104148763	0.044991677	0.071052254
RAGE	0.13212027	0.01085274	0.019844726
RAI14	0.167041566	0.0012409	0.002781726
RAI1	0.336553638	2.82E-11	2.31E-10
RAI2	0.074428319	0.152505292	0.207345474
RALA	0.290443472	1.21E-08	6.60E-08
RALBP1	-0.107199301	0.039037908	0.06252719
RALB	0.224732137	1.24E-05	4.03E-05
RALGAPA1	-0.260437446	3.63E-07	1.53E-06
RALGAPA2	-0.070712195	0.174108953	0.232510964
RALGAPB	0.293013703	8.84E-09	4.94E-08
RALGDS	0.305964554	1.76E-09	1.10E-08
RALGPS1	0.101398845	0.05099504	0.079412428
RALGPS2	-0.239650869	3.03E-06	1.10E-05
RALYL	0.186154662	0.000312089	0.000784832
RALY	0.363778419	4.74E-13	5.12E-12
RAMP1	-0.286753259	1.88E-08	9.89E-08
RAMP2	-0.007998366	0.877970882	0.903971053
RAMP3	-0.27312186	9.05E-08	4.23E-07
RANBP10	-0.399588802	1.18E-15	1.92E-14
RANBP17	0.250310773	1.04E-06	4.10E-06
RANBP1	0.173422349	0.000794996	0.001852604
RANBP2	0.036908726	0.478476637	0.552808282
RANBP3L	-0.478566499	1.24E-22	5.61E-21
RANBP3	0.224760989	1.24E-05	4.02E-05
RANBP6	-0.202024325	8.91E-05	0.000248218
RANBP9	-0.044094424	0.39707064	0.473338996
RANGAP1	0.331924564	5.43E-11	4.26E-10
RANGRF	0.306613092	1.62E-09	1.01E-08
RAN	0.575420855	4.30E-34	1.59E-31
RAP1A	0.063031784	0.225824255	0.291415391
RAP1B	0.332038068	5.35E-11	4.20E-10
RAP1GAP2	0.256995647	5.22E-07	2.15E-06
RAP1GAP	0.348533776	4.90E-12	4.51E-11
RAP1GDS1	-0.161535218	0.001799856	0.003906813
RAP2A	0.276745448	6.00E-08	2.90E-07
RAP2B	0.353183141	2.44E-12	2.36E-11
RAP2C	-0.208772377	5.07E-05	0.000147688
RAPGEF1	0.200826168	9.82E-05	0.000271899
RAPGEF2	-0.403427129	5.93E-16	1.01E-14
RAPGEF3	-0.164581614	0.001467233	0.003245277
RAPGEF4	-0.266361695	1.91E-07	8.51E-07
RAPGEF5	0.099967946	0.054374309	0.084050023
RAPGEF6	-0.041416265	0.426390763	0.502725737
RAPGEFL1	0.072896838	0.161148607	0.217403843
RAPH1	-0.203439795	7.93E-05	0.000222811
RAPSN	-0.020366407	0.695796135	0.752246736

RARA	0.079516131	0.126303578	0.176131869
RARB	-0.002164207	0.96686146	0.973950245
RARG	0.147317416	0.004462262	0.008885276
RARRES1	0.158319262	0.002224737	0.004742773
RARRES2	-0.341288127	1.42E-11	1.22E-10
RARRES3	0.03626219	0.486221437	0.560334555
RARS2	0.040075251	0.441532301	0.517257422
RARS	0.23558201	4.50E-06	1.58E-05
RASA1	0.046733903	0.36939272	0.445246961
RASA2	-0.002455491	0.962404379	0.970343643
RASA3	0.399251816	1.25E-15	2.02E-14
RASA4P	0.189261826	0.000246058	0.000630723
RASA4	0.168496199	0.001122648	0.002539791
RASAL1	0.456380338	1.74E-20	5.69E-19
RASAL2	0.084455881	0.104344098	0.149217078
RASAL3	0.223733803	1.36E-05	4.38E-05
RASD1	0.029180723	0.575286377	0.644411313
RASD2	0.21573169	2.78E-05	8.47E-05
RASEF	0.200292675	0.000102616	0.000282665
RASGEF1A	0.456103616	1.85E-20	6.00E-19
RASGEF1B	-0.115348986	0.026303473	0.043987055
RASGEF1C	0.307947054	1.37E-09	8.65E-09
RASGRF1	0.114383836	0.027595545	0.045889686
RASGRF2	-0.114538121	0.027385408	0.045589713
RASGRP1	0.234658422	4.91E-06	1.72E-05
RASGRP2	-0.014931487	0.774383213	0.818378992
RASGRP3	0.19796718	0.000123887	0.00033625
RASGRP4	0.072354347	0.164297094	0.221130034
RASIP1	-0.191411826	0.000208277	0.000542037
RASL10A	0.086914574	0.094601591	0.13684671
RASL10B	-0.187026778	0.000292056	0.000738087
RASL11A	-0.007546465	0.884816082	0.909065724
RASL11B	0.174273091	0.000748298	0.001752805
RASL12	0.234065234	5.20E-06	1.81E-05
RASSF10	0.155952134	0.002593922	0.005458552
RASSF1	0.251477222	9.27E-07	3.67E-06
RASSF2	0.206442108	6.17E-05	0.000177196
RASSF3	0.276398914	6.25E-08	3.01E-07
RASSF4	0.09415412	0.07007237	0.105111192
RASSF5	0.079269497	0.127487438	0.177462785
RASSF6	0.112117017	0.030846254	0.050661094
RASSF7	0.287764687	1.66E-08	8.85E-08
RASSF8	0.17537914	0.000691378	0.00163098
RASSF9	0.142866853	0.005839175	0.011319261
RAVER1	0.41025037	1.71E-16	3.19E-15
RAVER2	0.388927471	7.59E-15	1.08E-13
RAX2	0.059805665	0.250522987	0.318704913
RAX	0.243091599	2.16E-06	8.01E-06
RB1CC1	-0.05665455	0.27640281	0.346844091
RB1	-0.050668341	0.330417576	0.404434686
RBAK	-0.012094016	0.816406284	0.853821513
RBBP4	0.240505742	2.79E-06	1.02E-05
RBBP5	-0.095699557	0.065575917	0.099157755
RBBP6	0.072666336	0.162480796	0.219097226
RBBP7	0.460297631	7.46E-21	2.58E-19
RBBP8	0.405810166	3.85E-16	6.76E-15
RBBP9	-0.367615593	2.58E-13	2.91E-12
RBCK1	0.401666506	8.14E-16	1.36E-14

RBKS	-0.384066621	1.74E-14	2.33E-13
RBL1	0.375302052	7.46E-14	9.15E-13
RBL2	-0.402997155	6.41E-16	1.09E-14
RBM10	0.173999727	0.00076302	0.00178435
RBM11	0.236658678	4.05E-06	1.44E-05
RBM12B	0.293734926	8.10E-09	4.55E-08
RBM12	0.292978487	8.88E-09	4.96E-08
RBM14	0.363985025	4.59E-13	4.97E-12
RBM15B	0.26648803	1.89E-07	8.40E-07
RBM15	0.014053656	0.787319965	0.829630189
RBM16	0.121606279	0.019124238	0.032972139
RBM17	0.490604542	7.26E-24	3.87E-22
RBM18	-0.057563633	0.268756985	0.338636181
RBM19	0.30281475	2.63E-09	1.59E-08
RBM20	0.00404901	0.938045542	0.952193176
RBM22	0.340043036	1.71E-11	1.44E-10
RBM23	-0.138714057	0.007456164	0.014141441
RBM24	-0.109377465	0.035206812	0.057005047
RBM25	0.204144693	7.48E-05	0.000211422
RBM26	0.105829365	0.041623676	0.066279823
RBM27	0.085071481	0.10183331	0.146098459
RBM28	0.206808399	5.98E-05	0.000172229
RBM33	0.127686224	0.013847579	0.024675445
RBM34	0.275367104	7.02E-08	3.35E-07
RBM38	0.487082436	1.68E-23	8.58E-22
RBM39	0.289113471	1.42E-08	7.65E-08
RBM3	0.328460861	8.81E-11	6.70E-10
RBM41	0.016227853	0.755392971	0.802291486
RBM42	0.169384968	0.001055598	0.002397905
RBM43	-0.161420657	0.001813616	0.003934966
RBM44	0.02197083	0.673163022	0.732707956
RBM45	0.318871482	3.26E-10	2.26E-09
RBM46	-0.186501603	0.000303971	0.00076606
RBM47	0.142990781	0.005796177	0.011244663
RBM4B	0.135722249	0.008857764	0.016532169
RBM4	0.303415973	2.44E-09	1.48E-08
RBM5	0.138097206	0.007727767	0.014620378
RBM6	0.231772525	6.46E-06	2.21E-05
RBM7	-0.175206085	0.000700011	0.001649588
RBM8A	0.233675767	5.39E-06	1.88E-05
RBM9	-0.090844403	0.08055116	0.118790452
RBMS1	-0.016095312	0.757328019	0.804108554
RBMS2	0.193852849	0.000171984	0.000454292
RBMS3	-0.067434639	0.194983956	0.256553093
RBMX2	0.124193328	0.016696089	0.029166914
RBMXL1	0.006811982	0.895959793	0.917985548
RBMXL2	-0.181147477	0.00045417	0.001107598
RBMXL3	-0.012473344	0.810754995	0.848847016
RBMX	0.413572364	9.23E-17	1.80E-15
RBMY1A1	-0.017697542	0.734041	0.784357691
RBMY1A3P	0.121040264	0.01969473	0.033859027
RBMY1B	-0.015493104	0.76613893	0.811560576
RBMY1E	0.004819513	0.926286761	0.94246603
RBMY1F	-0.049502521	0.341679806	0.415970735
RBMY1J	-0.013906882	0.789488817	0.831563721
RBMY2EP	0.088838721	0.087495344	0.127740548
RBMY2FP	-0.043734839	0.400935412	0.477118169
RBMY3AP	0.063950518	0.219118475	0.28404986

RBP1	-0.039704775	0.445768813	0.521468367
RBP2	0.159578985	0.002048449	0.004400388
RBP3	0.141125358	0.006474437	0.012452571
RBP4	-0.448257457	9.74E-20	2.87E-18
RBP5	-0.344420287	9.01E-12	7.98E-11
RBP7	-0.329583032	7.54E-11	5.81E-10
RBPJL	0.133025444	0.010317169	0.018975133
RBPJ	0.237095772	3.89E-06	1.38E-05
RBPMS2	-0.026089515	0.616435123	0.681726464
RBPMS	0.152340237	0.003265573	0.006700664
RBX1	0.265817606	2.03E-07	8.99E-07
RC3H1	-0.061367882	0.238337744	0.305466067
RC3H2	-0.132788375	0.010455124	0.019189882
RCAN1	-0.371444595	1.40E-13	1.65E-12
RCAN2	0.137041918	0.008212943	0.015437141
RCAN3	0.418836733	3.43E-17	7.09E-16
RCBTB1	-0.100690792	0.05264465	0.08168757
RCBTB2	-0.024607734	0.636605242	0.700726539
RCC1	0.373800875	9.53E-14	1.15E-12
RCC2	0.571959799	1.29E-33	4.21E-31
RCCD1	0.146740715	0.004622389	0.009177519
RCE1	0.325251738	1.37E-10	1.01E-09
RCHY1	-0.090817517	0.080641207	0.118914443
RCL1	-0.376049622	6.60E-14	8.19E-13
RCN1	0.268712756	1.48E-07	6.70E-07
RCN2	0.340459997	1.61E-11	1.37E-10
RCN3	0.232190792	6.21E-06	2.13E-05
RCOR1	0.024098383	0.643602002	0.70702173
RCOR2	0.422470153	1.71E-17	3.70E-16
RCOR3	0.05068727	0.33023671	0.404238141
RCSD1	0.186332938	0.000307893	0.000775355
RCVRN	0.112996414	0.029548236	0.048775085
RD3	-0.007110474	0.891428421	0.914508802
RDBP	0.112207543	0.030710421	0.050471293
RDH10	0.09821556	0.058764219	0.089977771
RDH11	-0.142358613	0.006018525	0.01164088
RDH12	0.232469084	6.05E-06	2.08E-05
RDH13	0.250014614	1.08E-06	4.22E-06
RDH14	-0.094780158	0.068221441	0.102768056
RDH16	-0.398088086	1.54E-15	2.45E-14
RDH5	-0.508424944	8.80E-26	6.48E-24
RDH8	0.222636039	1.50E-05	4.81E-05
RDM1	0.364757574	4.06E-13	4.44E-12
RDX	-0.205873463	6.47E-05	0.000185046
REC8	0.259081849	4.19E-07	1.76E-06
RECK	0.204590929	7.20E-05	0.000204161
RECQL4	0.547330881	2.22E-30	3.35E-28
RECQL5	-0.069740603	0.180115844	0.239345507
RECQL	0.162244978	0.001716715	0.003738157
REEP1	0.226210754	1.08E-05	3.56E-05
REEP2	0.435128797	1.43E-18	3.64E-17
REEP3	0.030366467	0.559852779	0.630170465
REEP4	0.399217553	1.26E-15	2.03E-14
REEP5	-0.096827578	0.062445773	0.094914526
REEP6	-0.183257091	0.000388225	0.000958748
REG1A	0.053072733	0.307955162	0.380588259
REG1B	0.121130765	0.01960253	0.033712156
REG1P	-0.156328189	0.002531771	0.005340739

REG3A	-0.051493143	0.322595929	0.396444149
REG3G	-0.03312915	0.524689205	0.596760581
REG4	0.050639494	0.33069333	0.404707985
RELA	-0.034565863	0.506858522	0.579890627
RELB	0.377057761	5.59E-14	7.01E-13
RELL1	0.297084225	5.37E-09	3.10E-08
RELL2	0.374842346	8.04E-14	9.80E-13
RELN	0.003129579	0.95209456	0.962823748
RELT	0.556041556	1.71E-31	3.31E-29
REL	-0.047102722	0.365622652	0.441290104
REM1	0.063913471	0.219386092	0.284344233
REM2	0.278641049	4.83E-08	2.38E-07
RENBP	0.374947037	7.90E-14	9.66E-13
REN	-0.343075617	1.10E-11	9.61E-11
REP15	0.207241475	5.77E-05	0.000166469
REPIN1	-0.038178471	0.463463834	0.538374723
REPS1	-0.051285444	0.324554129	0.39858016
REPS2	-0.276365324	6.27E-08	3.02E-07
RER1	-0.207694351	5.55E-05	0.000160701
RERE	0.107606872	0.03829535	0.061446518
RERGL	-0.025218138	0.62826272	0.69307547
RERG	0.080475973	0.121777282	0.170668098
RESP18	0.05785198	0.266362358	0.336044005
REST	-0.071734424	0.167951219	0.225421019
RETNLB	0.073124197	0.159842651	0.215905166
RETN	0.098626594	0.057709074	0.088552736
RETSAT	-0.3365958	2.80E-11	2.29E-10
RET	0.131924243	0.010971916	0.020041052
REV1	-0.011871475	0.819726185	0.856663959
REV3L	-0.106022984	0.041249678	0.065778923
REXO1L1	0.073573319	0.157286329	0.212899732
REXO1	0.035547102	0.494865438	0.568782626
REXO2	0.027902396	0.592145968	0.659764008
REXO4	0.173784674	0.000774788	0.001809005
RFC1	0.105435751	0.042392808	0.067316237
RFC2	0.438353903	7.48E-19	1.97E-17
RFC3	0.238350467	3.44E-06	1.24E-05
RFC4	0.54531657	3.98E-30	5.92E-28
RFC5	0.381087728	2.86E-14	3.73E-13
RFESD	-0.003540116	0.945819418	0.958180242
RFFL	-0.131885413	0.010995659	0.020074737
RFK	0.122053098	0.0186841	0.032294347
RFNG	-0.278204964	5.08E-08	2.49E-07
RFPL1S	0.121344132	0.019386645	0.033386994
RFPL1	0.104432408	0.044407688	0.07023021
RFPL2	0.083653216	0.107691109	0.153343026
RFPL3S	0.195215494	0.000154393	0.000411701
RFPL3	0.064794847	0.213082438	0.277289721
RFPL4A	-0.130757649	0.011705403	0.021249776
RFPL4B	0.040161625	0.440547889	0.51623993
RFT1	0.214162311	3.19E-05	9.62E-05
RFTN1	0.080229935	0.122925294	0.172071228
RFTN2	0.165726159	0.001357588	0.003020535
RFWD2	0.288533707	1.52E-08	8.13E-08
RFWD3	0.370728271	1.57E-13	1.83E-12
RFX1	0.019796404	0.703903695	0.759130185
RFX2	0.220076541	1.89E-05	5.94E-05
RFX3	0.194959284	0.000157566	0.00041937

RFX4	0.243209131	2.14E-06	7.93E-06
RFX5	0.259380137	4.06E-07	1.70E-06
RFX6	-0.023327778	0.654247431	0.716577809
RFX7	0.061074029	0.240597381	0.307985673
RFX8	-0.05609263	0.281201992	0.352001639
RFXANK	0.316818588	4.28E-10	2.93E-09
RFXAP	0.19057654	0.00022226	0.000574973
RG9MTD1	0.076819434	0.139719401	0.192190147
RG9MTD2	-0.216097946	2.69E-05	8.21E-05
RG9MTD3	0.22867526	8.63E-06	2.89E-05
RGAG1	0.025460155	0.624967995	0.689861059
RGAG4	-0.013202614	0.799918014	0.839747144
RGL1	-0.060672761	0.24370722	0.311366333
RGL2	0.088730373	0.087883759	0.128214892
RGL3	0.159171146	0.002104074	0.004508697
RGL4	0.286333153	1.97E-08	1.04E-07
RGMA	0.044180916	0.396144385	0.472416833
RGMB	-0.237705943	3.66E-06	1.31E-05
RGNEF	-0.167750533	0.001181904	0.002662358
RGN	-0.433835509	1.85E-18	4.61E-17
RGP1	0.001406811	0.978455211	0.983242608
RGPD1	-0.035063234	0.500760509	0.574398368
RGPD3	-0.010700598	0.837245207	0.871179308
RGPD4	0.137882042	0.007824562	0.014783851
RGPD5	-0.00928241	0.85857192	0.888959502
RGPD6	0.087759252	0.09142727	0.132814209
RGPD8	-0.052755956	0.310855607	0.38367297
RGR	0.126451512	0.014801497	0.026164935
RGS10	0.399543306	1.19E-15	1.93E-14
RGS11	0.173310035	0.000801359	0.001865903
RGS12	-0.007672073	0.882912552	0.907604947
RGS13	0.176602138	0.00063312	0.001505493
RGS14	0.087615076	0.091962969	0.133495065
RGS16	-0.04437335	0.394088246	0.470416678
RGS17	0.313402599	6.73E-10	4.45E-09
RGS18	0.137287974	0.008097452	0.015243073
RGS19	0.491208372	6.28E-24	3.39E-22
RGS1	0.341322749	1.42E-11	1.22E-10
RGS20	0.336069408	3.02E-11	2.46E-10
RGS21	0.08081082	0.120228317	0.16874655
RGS22	0.125584956	0.015504976	0.027289525
RGS2	0.453037274	3.56E-20	1.10E-18
RGS3	0.158912198	0.002140104	0.004580485
RGS4	0.226708032	1.04E-05	3.41E-05
RGS5	-0.079270912	0.127480624	0.177462785
RGS6	-0.160743992	0.001896874	0.004101315
RGS7BP	0.068270753	0.189492658	0.250071061
RGS7	0.099303416	0.056005837	0.086298111
RGS8	0.067084508	0.197317696	0.259281257
RGS9BP	0.155137806	0.002733293	0.005710221
RGS9	-0.002907979	0.955483023	0.965304976
RGSL1	-0.408116802	2.53E-16	4.56E-15
RHAG	0.040874071	0.432476089	0.50848611
RHBDD1	-0.178170821	0.00056505	0.001355276
RHBDD2	-0.069050055	0.184477479	0.244237585
RHBDD3	0.010836781	0.835203295	0.869463346
RHBDF1	0.260701849	3.53E-07	1.50E-06
RHBDF2	0.316459142	4.49E-10	3.06E-09

RHBDL1	0.066689285	0.199976379	0.262445943
RHBDL2	0.290104334	1.26E-08	6.86E-08
RHBDL3	-0.035740275	0.492522323	0.566547128
RHBG	-0.196694306	0.000137218	0.000369064
RHCE	-0.342531822	1.19E-11	1.03E-10
RHCG	-0.086798701	0.095043825	0.13738667
RHD	-0.209971014	4.58E-05	0.000134624
RHEBL1	0.292600982	9.30E-09	5.17E-08
RHEB	0.132812611	0.010440946	0.019170924
RHOA	0.223527658	1.39E-05	4.46E-05
RHOBTB1	-0.039815696	0.44449798	0.520226105
RHOBTB2	0.265102392	2.20E-07	9.67E-07
RHOBTB3	-0.075692853	0.145637246	0.199106864
RHOB	-0.373591743	9.86E-14	1.19E-12
RHOC	0.117380786	0.023752696	0.04015284
RHOD	0.005682115	0.913141561	0.932042533
RHOF	0.427365549	6.64E-18	1.51E-16
RHOG	0.404719849	4.69E-16	8.10E-15
RHOH	0.275198311	7.16E-08	3.41E-07
RHOJ	-0.023431577	0.652809358	0.715400629
RHOQ	0.291877743	1.02E-08	5.60E-08
RHOT1	0.069300575	0.182886237	0.242364107
RHOT2	0.158786455	0.002157802	0.004612418
RHOU	-0.267962191	1.61E-07	7.22E-07
RHOV	0.422011322	1.87E-17	4.02E-16
RHOXF1	-0.139611282	0.007076326	0.013490389
RHOXF2B	0.053440249	0.304612558	0.377251858
RHO	0.209382126	4.81E-05	0.000140888
RHPN1	0.247347983	1.41E-06	5.40E-06
RHPN2	0.188468827	0.000261543	0.00066681
RIBC1	0.080627678	0.121073598	0.169813307
RIBC2	0.470506425	7.79E-22	3.11E-20
RIC3	0.213003598	3.53E-05	0.000105758
RIC8A	0.257482462	4.96E-07	2.05E-06
RIC8B	0.325965444	1.24E-10	9.22E-10
RICH2	0.191956292	0.00019961	0.000520774
RICTOR	0.101526589	0.050702061	0.07900559
RIF1	0.081949586	0.115075264	0.16245248
RILPL1	0.236367267	4.17E-06	1.48E-05
RILPL2	0.040454362	0.437220902	0.513162734
RILP	-0.236842666	3.98E-06	1.41E-05
RIMBP2	0.037516021	0.471263491	0.546034156
RIMBP3C	0.33312396	4.59E-11	3.64E-10
RIMBP3	0.364071696	4.53E-13	4.90E-12
RIMKLA	0.127729491	0.013815155	0.024624275
RIMKLB	0.117368062	0.023767981	0.04017492
RIMS1	-0.044149834	0.396477089	0.472716295
RIMS2	0.069648768	0.180691453	0.239935605
RIMS3	0.360650724	7.74E-13	8.07E-12
RIMS4	0.285548455	2.16E-08	1.13E-07
RIN1	0.37078216	1.55E-13	1.82E-12
RIN2	-0.004067215	0.937767548	0.95208282
RIN3	0.065132656	0.210701265	0.274531759
RING1	-0.168092258	0.0011544	0.002605705
RINL	0.22020545	1.87E-05	5.88E-05
RINT1	-0.023083489	0.657636935	0.719701331
RIOK1	0.221193065	1.71E-05	5.42E-05
RIOK2	0.047238567	0.364240096	0.439861259

RIOK3	-0.062143384	0.232446156	0.298915073
RIPK1	-0.129834107	0.012316458	0.022235476
RIPK2	0.405404043	4.15E-16	7.24E-15
RIPK3	0.250447376	1.03E-06	4.05E-06
RIPK4	-0.125949705	0.015205375	0.026826203
RIPPLY1	-0.365678734	3.51E-13	3.88E-12
RIPPLY2	0.181416424	0.000445219	0.001087233
RIT1	0.439054848	6.49E-19	1.72E-17
RIT2	0.031808199	0.54136093	0.612888131
RLBP1	0.014116751	0.786388121	0.828867475
RLF	0.016712416	0.748331643	0.796594335
RLIM	0.011423334	0.826421328	0.862141351
RLN1	0.027649836	0.595503467	0.66266101
RLN2	0.216525903	2.59E-05	7.94E-05
RLN3	0.23903903	3.22E-06	1.16E-05
RLTPR	0.287371772	1.74E-08	9.24E-08
RMI1	0.105048581	0.043160994	0.068399755
RMND1	-0.124083011	0.016793846	0.029322261
RMND5A	-0.333261013	4.50E-11	3.58E-10
RMND5B	0.143032442	0.005781786	0.011218929
RMRP	0.120223402	0.020544126	0.035197805
RMST	0.194430861	0.000164305	0.00043574
RNASE10	0.112852692	0.02975712	0.049078678
RNASE11	-0.06298954	0.226136065	0.291723285
RNASE12	-0.069658068	0.180633099	0.239888847
RNASE13	-0.133121441	0.010261767	0.018888933
RNASE1	0.055282867	0.288216468	0.35969733
RNASE2	0.370714582	1.57E-13	1.84E-12
RNASE3	0.195144681	0.000155264	0.000413802
RNASE4	-0.396547585	2.02E-15	3.15E-14
RNASE6	0.240518963	2.79E-06	1.02E-05
RNASE7	0.04966854	0.340061237	0.414278723
RNASE8	0.012802781	0.805854936	0.844915637
RNASE9	-0.051334496	0.324090967	0.398084977
RNASEH1	0.308261534	1.31E-09	8.32E-09
RNASEH2A	0.468994906	1.09E-21	4.26E-20
RNASEH2B	0.201362444	9.40E-05	0.000260936
RNASEH2C	0.172997962	0.000819285	0.001903638
RNASEK	0.112339383	0.030513513	0.050197374
RNASEL	-0.197712008	0.000126458	0.000342575
RNASEN	0.409814543	1.85E-16	3.42E-15
RNASET2	0.288073776	1.60E-08	8.55E-08
RND1	-0.011030299	0.832303583	0.866905046
RND2	0.041227792	0.428500432	0.504854551
RND3	-0.033960159	0.514336888	0.587030621
RNF103	-0.160772817	0.001893257	0.004094268
RNF10	0.099030325	0.056687991	0.087200846
RNF111	-0.127831577	0.013738918	0.024510318
RNF112	0.088712834	0.087946764	0.128272599
RNF113A	0.063591665	0.221720568	0.287068119
RNF113B	0.123253876	0.017544731	0.030486361
RNF114	0.245347582	1.73E-06	6.50E-06
RNF115	0.218543223	2.17E-05	6.74E-05
RNF11	-0.329584658	7.54E-11	5.81E-10
RNF121	0.095861756	0.065118055	0.098540213
RNF122	0.284225713	2.53E-08	1.31E-07
RNF123	-0.300971367	3.31E-09	1.97E-08
RNF125	-0.40005137	1.09E-15	1.77E-14

RNF126P1	0.062278747	0.231428433	0.29776009
RNF126	0.130696421	0.011745069	0.021314015
RNF128	-0.225839522	1.12E-05	3.67E-05
RNF130	-0.134815242	0.009326688	0.017335909
RNF133	0.150824405	0.003591741	0.007304477
RNF135	0.109834417	0.034444999	0.055924592
RNF138P1	0.116421008	0.02492969	0.041907796
RNF138	0.21724451	2.43E-05	7.47E-05
RNF139	0.03007754	0.563594961	0.63373815
RNF13	-0.110299032	0.033684838	0.054816969
RNF141	-0.167354122	0.001214565	0.002726987
RNF144A	0.322184967	2.08E-10	1.50E-09
RNF144B	-0.362694003	5.62E-13	5.99E-12
RNF145	0.427529249	6.43E-18	1.47E-16
RNF146	-0.073196359	0.15942982	0.215435181
RNF148	0.203048931	8.19E-05	0.000229544
RNF149	0.21177429	3.92E-05	0.000116722
RNF14	0.039220338	0.451343159	0.527140219
RNF150	0.112718728	0.029952959	0.049377149
RNF151	0.0875641	0.092152977	0.133731906
RNF152	-0.160280432	0.001955913	0.004216154
RNF157	0.091978382	0.07682668	0.113904973
RNF160	-0.069839764	0.179495844	0.238633047
RNF165	-0.212254522	3.76E-05	0.000112409
RNF166	0.089977845	0.083494666	0.122650107
RNF167	-0.158513299	0.002196711	0.004687221
RNF168	-0.071849695	0.167267199	0.224639149
RNF169	0.017277467	0.7401239	0.789713906
RNF170	-0.164465271	0.001478821	0.003268007
RNF175	0.194880081	0.000158559	0.000421738
RNF17	0.122309846	0.018435206	0.03190567
RNF180	-0.007430847	0.886568815	0.910517781
RNF181	0.025111984	0.629710201	0.694287821
RNF182	0.017013319	0.743957214	0.792827411
RNF183	0.246553083	1.53E-06	5.82E-06
RNF185	-0.329210032	7.94E-11	6.09E-10
RNF186	0.26752561	1.68E-07	7.55E-07
RNF187	0.116314956	0.025062779	0.042099552
RNF19A	0.19194212	0.000199831	0.000521215
RNF19B	0.208470968	5.20E-05	0.000151247
RNF207	0.091599588	0.078054935	0.115571194
RNF208	-0.041134031	0.4295522	0.505884306
RNF20	-0.051089456	0.326408966	0.400463075
RNF212	0.186412194	0.000306044	0.00077109
RNF213	0.166274074	0.001307809	0.002916948
RNF214	0.086190196	0.097393349	0.140416146
RNF215	-0.139864629	0.006972252	0.013316193
RNF216L	0.239069853	3.21E-06	1.16E-05
RNF216	0.13468716	0.009394653	0.017444336
RNF217	-0.317387554	3.97E-10	2.73E-09
RNF219	0.298069686	4.76E-09	2.76E-08
RNF220	0.137434514	0.008029361	0.015130622
RNF222	0.015427399	0.767102114	0.812278533
RNF24	0.515043849	1.60E-26	1.34E-24
RNF25	0.062652271	0.228636452	0.29456738
RNF26	0.149501732	0.003900156	0.007873027
RNF2	0.12829218	0.013399525	0.023956325
RNF31	0.102289496	0.048981454	0.076664804

RNF32	-0.094608594	0.068724657	0.103377576
RNF34	0.550071751	1.00E-30	1.57E-28
RNF38	0.034129471	0.512240732	0.58490628
RNF39	0.305330986	1.91E-09	1.18E-08
RNF40	6.83E-05	0.998954395	0.999155008
RNF41	0.113694357	0.028551621	0.04732158
RNF43	-0.280948592	3.70E-08	1.86E-07
RNF44	0.374568501	8.41E-14	1.02E-12
RNF4	0.209412463	4.80E-05	0.000140546
RNF5P1	-0.095212536	0.066966606	0.100999979
RNF5	-0.167150017	0.001231703	0.002762976
RNF6	-0.002877071	0.955955697	0.965503138
RNF7	0.137492197	0.008002698	0.015087513
RNF8	0.27005904	1.27E-07	5.83E-07
RNFT1	-0.041813065	0.421968954	0.498131399
RNFT2	0.544944772	4.43E-30	6.49E-28
RNGTT	0.063494964	0.22242552	0.28786859
RNH1	-0.013336682	0.797929855	0.838459759
RNLS	-0.362103338	6.17E-13	6.52E-12
RNMTL1	-0.172440216	0.000852249	0.001974015
RNMT	0.06882364	0.18592438	0.245964904
RNPC3	0.050296693	0.333981556	0.407916999
RNPEPL1	0.063324598	0.223671368	0.289311842
RNPEP	-0.056151189	0.280699247	0.351416477
RNPS1	0.321773308	2.20E-10	1.58E-09
RNU11	0.097507871	0.060618256	0.092454211
RNU4ATAC	0.030514615	0.557938634	0.628466077
RNU6ATAC	0.077833662	0.134550999	0.186005482
ROBLD3	-0.090872031	0.080458712	0.118671686
ROBO1	0.130629667	0.01178845	0.021383002
ROBO2	0.046939058	0.367292659	0.443117807
ROBO3	0.038980396	0.454118606	0.529572791
ROBO4	-0.301904453	2.95E-09	1.77E-08
ROCK1	-0.005595636	0.914458403	0.93307755
ROCK2	-0.153581845	0.003018696	0.006246854
ROD1	0.420421002	2.54E-17	5.32E-16
ROGDI	-0.195947978	0.000145648	0.000389896
ROM1	0.044440912	0.393367885	0.469712
ROMO1	0.208090625	5.37E-05	0.000155765
ROPN1B	-0.166556382	0.001282822	0.002866671
ROPN1L	0.003614082	0.944689179	0.957278627
ROPN1	-0.164435719	0.001481778	0.003273452
ROR1	0.139450562	0.007143067	0.013611113
ROR2	0.223874114	1.34E-05	4.33E-05
RORA	-0.49049257	7.46E-24	3.96E-22
RORB	0.109931513	0.034284944	0.055688924
RORC	-0.470242596	8.27E-22	3.28E-20
ROS1	-0.12536933	0.015684512	0.027573847
RP1-177G6.2	0.120259273	0.020506169	0.035138818
RP1L1	-0.014663973	0.77831919	0.821797482
RP1	0.098164784	0.058895665	0.090144395
RP2	0.107580917	0.038342278	0.06149705
RP9P	0.284249078	2.52E-08	1.30E-07
RP9	0.062080993	0.232916312	0.299442357
RPA1	0.244203029	1.94E-06	7.24E-06
RPA2	0.085601972	0.099708287	0.143349101
RPA3	-0.055148805	0.289389011	0.361002372
RPA4	0.132881195	0.010400917	0.019109754

RPAIN	-0.033635156	0.518372992	0.590824804
RPAP1	0.063738428	0.220653713	0.285878214
RPAP2	0.071400628	0.169943782	0.22778847
RPAP3	0.371615312	1.36E-13	1.60E-12
RPE65	0.057502522	0.269266379	0.339149321
RPE	0.248307858	1.28E-06	4.95E-06
RPF1	0.035721231	0.49275306	0.566747097
RPF2	0.199844561	0.000106427	0.000292365
RPGRIP1L	0.142650035	0.005915092	0.011458621
RPGRIP1	-0.046780014	0.368920055	0.444721215
RPGR	0.155881871	0.002605687	0.005479258
RPH3AL	-0.124065247	0.016809634	0.029347255
RPH3A	0.126905962	0.014443891	0.025616507
RPIA	0.397725791	1.64E-15	2.59E-14
RPL10A	0.022240137	0.669392037	0.729839545
RPL10L	0.079460169	0.126571452	0.176443669
RPL10	0.021490849	0.679904247	0.738555329
RPL11	-0.015736003	0.762581374	0.808695099
RPL12	0.009873644	0.849667518	0.881343137
RPL13AP17	0.059639664	0.251842798	0.320281662
RPL13AP20	0.11659141	0.024717123	0.04160312
RPL13AP3	0.074612575	0.151489542	0.206061359
RPL13AP6	-0.00674066	0.89704303	0.918906378
RPL13A	0.097417157	0.060859367	0.092759876
RPL13P5	0.253557361	7.48E-07	3.00E-06
RPL13	-0.051097795	0.326329902	0.400390731
RPL14	0.097477552	0.060698756	0.09255573
RPL15	-0.01017538	0.845130428	0.877504997
RPL17	0.143775319	0.005530509	0.010775433
RPL18A	0.155297569	0.002705424	0.005657338
RPL18	0.135276753	0.009085399	0.016926913
RPL19P12	0.094117695	0.070181313	0.105242914
RPL19	0.122044998	0.018692	0.03229973
RPL21P44	-0.022996457	0.6588462	0.720707994
RPL21	0.040144505	0.440742912	0.516438084
RPL22L1	0.217275282	2.43E-05	7.46E-05
RPL22	-0.015152975	0.771128791	0.815718567
RPL23AP32	0.069478665	0.181761227	0.241113808
RPL23AP53	0.094409744	0.0693117	0.104142672
RPL23AP64	-0.075506322	0.146635251	0.200320062
RPL23AP7	0.100978435	0.051969224	0.080733849
RPL23AP82	0.077720748	0.13511901	0.186635299
RPL23A	0.139007083	0.007330156	0.013931632
RPL23P8	0.13292653	0.010374532	0.01906831
RPL23	0.104850175	0.04355917	0.068959456
RPL24	0.102748487	0.047969994	0.075313911
RPL26L1	0.10186589	0.049930699	0.077913322
RPL26	0.037832555	0.467527666	0.542399614
RPL27A	0.157412934	0.002360018	0.005008126
RPL27	0.139427971	0.007152493	0.013626467
RPL28	0.139422331	0.007154848	0.013628348
RPL29P2	-0.030180917	0.562254643	0.63251663
RPL29	0.053388434	0.305082368	0.377670474
RPL30	0.118288076	0.022684528	0.038530238
RPL31P11	0.183393891	0.000384273	0.000949576
RPL31	0.052897058	0.309561455	0.38235993
RPL32P3	0.111835529	0.031271908	0.051292521
RPL32	0.103277301	0.046826394	0.073663568

RPL34	-0.060432942	0.245579186	0.313376156
RPL35A	0.168860424	0.00109471	0.002480811
RPL35	0.138047589	0.007749993	0.014659642
RPL36AL	-0.066148905	0.20365357	0.266674539
RPL36A	0.183019733	0.000395173	0.000973853
RPL36	0.109401898	0.035165718	0.056952397
RPL37A	0.090275389	0.082474347	0.121276494
RPL37	0.139839198	0.006982637	0.013334747
RPL38	0.122916595	0.017858469	0.03098558
RPL39L	0.263708107	2.55E-07	1.11E-06
RPL39	0.11084467	0.032810403	0.053560214
RPL3L	0.039447335	0.448726266	0.524527646
RPL3	-0.103662814	0.04600717	0.072477846
RPL41	0.010005242	0.847688106	0.87967586
RPL4	0.075951345	0.14426279	0.197471712
RPL5	0.07396678	0.155072284	0.210288615
RPL6	0.182381929	0.000414419	0.001017131
RPL7A	0.093963991	0.070642558	0.105831031
RPL7L1	-0.023422412	0.65293628	0.715461008
RPL7	0.12842034	0.013306412	0.023809084
RPL8	0.251015834	9.72E-07	3.84E-06
RPL9	0.144924774	0.005161014	0.010130823
RPLP0P2	0.363464917	4.98E-13	5.36E-12
RPLP0	0.188803133	0.000254907	0.000651226
RPLP1	0.112366277	0.030473478	0.050139795
RPLP2	0.179283181	0.000520965	0.00125741
RPN1	0.125076815	0.015930978	0.02796644
RPN2	0.294761947	7.15E-09	4.06E-08
RPP14	-0.304882246	2.02E-09	1.25E-08
RPP21	0.262663488	2.86E-07	1.23E-06
RPP25	0.373626823	9.80E-14	1.18E-12
RPP30	0.306016095	1.75E-09	1.09E-08
RPP38	0.194714535	0.000160654	0.000426741
RPP40	-0.034163549	0.51181936	0.584525641
RPPH1	0.084986759	0.102175986	0.146495141
RPRD1A	0.238174245	3.50E-06	1.25E-05
RPRD1B	-0.067645078	0.193591045	0.254922387
RPRD2	0.084644818	0.103568341	0.148224748
RPRML	0.068224962	0.189790433	0.250414266
RPRM	0.131312094	0.011351565	0.020660138
RPS10P7	0.031251111	0.548470089	0.619704011
RPS10	0.099021953	0.05670901	0.087226444
RPS11	0.098084332	0.059104432	0.090422247
RPS12	0.072408866	0.163978606	0.220773251
RPS13	0.068861122	0.185684277	0.245663578
RPS14	0.11244771	0.030352531	0.049973812
RPS15AP10	-0.028229607	0.587808993	0.655520582
RPS15A	0.075039275	0.149156971	0.203388444
RPS15	0.158850953	0.002148708	0.004595937
RPS16	0.122051005	0.018686142	0.032295074
RPS17	0.090955097	0.080181277	0.11834134
RPS18	0.115071904	0.026668971	0.044530989
RPS19BP1	0.10243	0.048669953	0.076262609
RPS19	0.194882747	0.000158526	0.000421706
RPS20	0.116999821	0.024214007	0.040835642
RPS21	0.1828213	0.00040107	0.000987408
RPS23	0.161803223	0.001768038	0.003841097
RPS24	0.170111207	0.001003571	0.002290164

RPS25	-0.012322316	0.813003879	0.850799143
RPS26P11	0.024287491	0.641000576	0.704746881
RPS26	0.024188504	0.642361721	0.706009611
RPS27A	0.178869768	0.000536962	0.001293045
RPS27L	-0.100006116	0.054281809	0.083914193
RPS27	-0.004432988	0.932183751	0.947353195
RPS28	0.030501701	0.558105366	0.628558714
RPS29	-0.012990178	0.803070963	0.842395731
RPS2P32	0.260628695	3.55E-07	1.51E-06
RPS2	0.209846054	4.63E-05	0.000135847
RPS3A	0.013760379	0.791655298	0.833493122
RPS3	0.192870359	0.000185814	0.000487784
RPS4X	0.107713795	0.038102537	0.06118149
RPS4Y1	-0.082169453	0.114100567	0.161225015
RPS4Y2	0.008621861	0.868541437	0.896534038
RPS5	0.131429002	0.011278175	0.020535947
RPS6KA1	0.192418191	0.000192523	0.00050367
RPS6KA2	-0.164535809	0.001471785	0.003253902
RPS6KA3	0.067602473	0.193872462	0.255242347
RPS6KA4	0.319960046	2.81E-10	1.98E-09
RPS6KA5	0.014086909	0.786828819	0.829156506
RPS6KA6	0.153677123	0.00300047	0.006210428
RPS6KB1	0.106012793	0.041269292	0.065804934
RPS6KB2	0.1856349	0.00032463	0.000813392
RPS6KC1	0.185210014	0.000335229	0.000838368
RPS6KL1	-0.035379068	0.496908432	0.570834378
RPS6	0.082391905	0.113121007	0.160090695
RPS7	0.224685659	1.25E-05	4.05E-05
RPS8	0.119705849	0.021098606	0.036070233
RPS9	0.092005294	0.076740015	0.11380189
RPSAP52	0.219907399	1.92E-05	6.02E-05
RPSAP58	0.275245813	7.12E-08	3.39E-07
RPSAP9	0.181983839	0.00042687	0.001044987
RPSA	0.263721768	2.55E-07	1.11E-06
RPTN	0.086420648	0.09649816	0.139279566
RPTOR	-0.009059732	0.861930324	0.891557697
RPUSD1	0.181790805	0.000433032	0.00105903
RPUSD2	-0.163835517	0.001543022	0.003392996
RPUSD3	-0.051163598	0.325706481	0.399809003
RPUSD4	-0.185179336	0.000336007	0.000839785
RQCD1	0.229937417	7.68E-06	2.59E-05
RRAD	0.277178734	5.71E-08	2.77E-07
RRAGA	-0.155184056	0.002725199	0.005695102
RRAGB	0.078065028	0.133392864	0.184596599
RRAGC	0.122611569	0.018146409	0.031444095
RRAGD	0.236185704	4.24E-06	1.50E-05
RRAS2	0.077910165	0.1341672	0.185552166
RRAS	0.165939673	0.001337987	0.002980251
RRBP1	0.096292119	0.063915922	0.096932543
RREB1	-0.206639177	6.07E-05	0.00017454
RRH	-0.065458511	0.208422573	0.271989683
RRM1	0.381050694	2.88E-14	3.75E-13
RRM2B	0.037717408	0.468884762	0.543752385
RRM2	0.537791403	3.39E-29	4.24E-27
RRN3P1	0.045655375	0.380554834	0.456739166
RRN3P2	0.117366555	0.023769791	0.04017492
RRN3P3	0.258290204	4.56E-07	1.90E-06
RRN3	-0.286824614	1.86E-08	9.82E-08

RRP12	0.250827954	9.91E-07	3.91E-06
RRP15	0.107820751	0.03791049	0.060907483
RRP1B	0.07232475	0.164470185	0.221345338
RRP1	0.294776939	7.13E-09	4.05E-08
RRP7A	0.087782511	0.091341081	0.132718038
RRP7B	0.269519016	1.35E-07	6.16E-07
RRP8	-0.007085754	0.891803557	0.914762149
RRP9	0.203943759	7.60E-05	0.000214498
RRS1	0.203267542	8.04E-05	0.000225753
RS1	0.047042151	0.366240166	0.441981862
RSAD1	-0.32631988	1.18E-10	8.81E-10
RSAD2	-0.039162404	0.452012412	0.527703284
RSBN1L	-0.057599007	0.268462426	0.33828643
RSBN1	-0.129492326	0.01254963	0.02261749
RSC1A1	-0.390720852	5.58E-15	8.15E-14
RSF1	-0.039768819	0.445034806	0.520732022
RSL1D1	0.051223596	0.325138722	0.399199656
RSL24D1	0.066511275	0.201182336	0.263802706
RSPH10B2	0.035623649	0.493936247	0.567878457
RSPH1	0.384514489	1.61E-14	2.18E-13
RSPH3	0.197326354	0.000130439	0.000352257
RSPH4A	0.112307082	0.030561655	0.050264121
RSPH6A	0.12101158	0.019724031	0.033896591
RSPH9	0.187264413	0.000286809	0.000726209
RSPO1	0.134158194	0.00968003	0.017925782
RSPO2	-0.272337454	9.88E-08	4.59E-07
RSPO3	0.090762925	0.080824298	0.119120619
RSPO4	-0.007928228	0.879032725	0.904643556
RSPRY1	-0.205450209	6.71E-05	0.000191269
RSRC1	0.145517093	0.004979421	0.00981984
RSRC2	0.225374019	1.17E-05	3.82E-05
RSU1	0.143772482	0.00553145	0.010776211
RTBDN	0.065453331	0.208458655	0.272018951
RTCD1	0.228968876	8.40E-06	2.82E-05
RTDR1	0.281863446	3.33E-08	1.69E-07
RTEL1	0.133430967	0.010084925	0.018597785
RTF1	-0.034922011	0.502488025	0.575882553
RTKN2	0.492176052	4.97E-24	2.74E-22
RTKN	0.318257359	3.54E-10	2.44E-09
RTL1	0.215886338	2.74E-05	8.36E-05
RTN1	0.163154927	0.001615271	0.003533483
RTN2	0.131912752	0.010978938	0.020049965
RTN3	0.28633582	1.97E-08	1.04E-07
RTN4IP1	0.052725748	0.311133133	0.383944145
RTN4RL1	-0.014231668	0.784691714	0.827473445
RTN4RL2	0.056088784	0.281235034	0.352020882
RTN4R	0.187768183	0.000275976	0.000701364
RTN4	-0.153773513	0.002982134	0.006175683
RTP1	-0.185705667	0.000322895	0.000809759
RTP2	-0.145712718	0.004920723	0.009711773
RTP3	-0.415377592	6.59E-17	1.31E-15
RTP4	-0.068459981	0.188265782	0.248663676
RTTN	-0.035342291	0.497356174	0.571236175
RUFY1	0.400229898	1.05E-15	1.72E-14
RUFY2	0.097108964	0.061684436	0.09389344
RUFY3	-0.000821903	0.98741186	0.990544291
RUFY4	0.299808176	3.83E-09	2.26E-08
RUNDC1	0.264944638	2.23E-07	9.82E-07

RUNDC2A	0.043900184	0.399155517	0.475426056
RUNDC2C	-0.054921381	0.29138542	0.363106295
RUNDC3A	0.48699404	1.72E-23	8.72E-22
RUNDC3B	-0.541246066	1.28E-29	1.68E-27
RUNX1T1	-0.117357896	0.023780198	0.04018569
RUNX1	0.322760456	1.93E-10	1.39E-09
RUNX2	0.317599992	3.86E-10	2.65E-09
RUNX3	0.223649452	1.37E-05	4.41E-05
RUSC1	0.507359261	1.15E-25	8.36E-24
RUSC2	0.155482747	0.002673446	0.005603997
RUVBL1	0.245800775	1.65E-06	6.23E-06
RUVBL2	0.131498812	0.011234551	0.020465868
RWDD1	0.132153318	0.010832761	0.019817288
RWDD2A	0.129824229	0.012323143	0.022245528
RWDD2B	-0.323386019	1.77E-10	1.28E-09
RWDD3	-0.153440042	0.003046008	0.006294219
RWDD4A	-0.040003932	0.442346071	0.518072806
RXFP1	0.045341833	0.383838116	0.460082182
RXFP2	0.045303258	0.384243256	0.46050016
RXFP3	0.01510259	0.771868772	0.81628462
RXFP4	0.242227016	2.35E-06	8.68E-06
RXRA	-0.267813469	1.63E-07	7.33E-07
RXRB	-0.238278321	3.47E-06	1.24E-05
RXRG	-0.143519997	0.005615742	0.01092547
RYBP	-0.063191832	0.224645699	0.290257568
RYK	0.136949135	0.008256873	0.015514999
RYR1	0.280716648	3.80E-08	1.91E-07
RYR2	0.167343259	0.001215471	0.002728715
RYR3	0.108294366	0.037069902	0.059706248
S100A10	0.190937591	0.000216111	0.000559939
S100A11	0.434481659	1.63E-18	4.09E-17
S100A12	-0.026004019	0.617591289	0.682701901
S100A13	0.223593627	1.38E-05	4.43E-05
S100A14	0.399056509	1.30E-15	2.09E-14
S100A16	0.329591257	7.53E-11	5.81E-10
S100A1	-0.07383389	0.155817423	0.211198442
S100A2	0.386333843	1.18E-14	1.63E-13
S100A3	0.278267354	5.04E-08	2.47E-07
S100A4	0.349552019	4.21E-12	3.94E-11
S100A5	0.247426172	1.40E-06	5.37E-06
S100A6	0.417175431	4.70E-17	9.50E-16
S100A7A	0.013605083	0.793953549	0.835294814
S100A7	0.114602315	0.027298383	0.045471436
S100A8	0.096008936	0.064704863	0.097981931
S100A9	0.301672628	3.03E-09	1.82E-08
S100B	0.300970804	3.31E-09	1.97E-08
S100G	0.046621359	0.370547918	0.446423296
S100PBP	0.286251976	1.99E-08	1.05E-07
S100P	0.405909386	3.78E-16	6.64E-15
S100Z	0.164332455	0.001492154	0.003293321
S1PR1	-0.309985298	1.05E-09	6.76E-09
S1PR2	0.379207588	3.92E-14	5.01E-13
S1PR3	0.14338472	0.005661378	0.011004583
S1PR4	0.079605896	0.125874826	0.175607723
S1PR5	0.124747929	0.016212133	0.028400997
SAA1	-0.0833983	0.108771609	0.154704647
SAA2	-0.076544912	0.141144177	0.193868884
SAA3P	0.081273445	0.118113591	0.166164746

SAA4	-0.185980453	0.000316241	0.000794271
SAAL1	0.37840237	4.47E-14	5.67E-13
SAC3D1	0.307566593	1.44E-09	9.05E-09
SACM1L	-0.087376682	0.092854253	0.134592727
SACS	0.192072146	0.00019781	0.000516281
SAE1	0.510511809	5.16E-26	4.03E-24
SAFB2	0.243488798	2.08E-06	7.73E-06
SAFB	0.125039839	0.015962373	0.028017831
SAGE1	0.289686909	1.32E-08	7.18E-08
SAG	0.103898539	0.045512197	0.071783231
SALL1	-0.533757503	1.04E-28	1.20E-26
SALL2	0.406455421	3.43E-16	6.05E-15
SALL3	0.127561659	0.013941301	0.024813598
SALL4	0.355573101	1.69E-12	1.68E-11
SAMD10	0.307929348	1.37E-09	8.66E-09
SAMD11	0.139759743	0.007015172	0.013385332
SAMD12	0.270574721	1.20E-07	5.52E-07
SAMD13	0.163037034	0.001628097	0.00355897
SAMD14	0.282105719	3.24E-08	1.65E-07
SAMD1	0.288572859	1.51E-08	8.10E-08
SAMD3	0.13455228	0.009466702	0.017566658
SAMD4A	-0.346185919	6.95E-12	6.26E-11
SAMD4B	0.102138727	0.049317562	0.077119149
SAMD5	0.208077749	5.38E-05	0.000155889
SAMD7	0.106606323	0.040139984	0.064163424
SAMD8	0.002886824	0.95580655	0.965477618
SAMD9L	0.203128177	8.13E-05	0.000228175
SAMD9	0.151331738	0.003479429	0.007095627
SAMHD1	-0.048161746	0.354930923	0.429844003
SAMM50	-0.269579286	1.34E-07	6.12E-07
SAMSN1	0.221378086	1.68E-05	5.33E-05
SAP130	0.183710401	0.000375271	0.00092883
SAP18	-0.087109837	0.093860081	0.135902357
SAP30BP	0.154443616	0.002857446	0.005942176
SAP30L	-0.139870823	0.006969725	0.013312642
SAP30	0.240001175	2.93E-06	1.07E-05
SAPS1	0.421908393	1.91E-17	4.09E-16
SAPS2	-0.243476183	2.08E-06	7.73E-06
SAPS3	0.197957274	0.000123986	0.000336427
SAR1A	0.050594799	0.331120869	0.40514617
SAR1B	-0.094139763	0.070115294	0.105167661
SARDH	-0.418263091	3.82E-17	7.83E-16
SARM1	0.135107069	0.009173461	0.017076591
SARNP	0.367521066	2.62E-13	2.96E-12
SARS2	0.134887354	0.009288614	0.017273198
SARS	0.126326538	0.014901192	0.026322181
SART1	0.117261753	0.023896011	0.040357437
SART3	0.315337456	5.21E-10	3.51E-09
SASH1	-0.038725425	0.457078389	0.532105163
SASH3	0.235500335	4.53E-06	1.60E-05
SASS6	0.315104383	5.38E-10	3.61E-09
SAT1	-0.193541724	0.000176256	0.00046459
SAT2	-0.179831868	0.000500415	0.001211459
SATB1	-0.099276808	0.056072001	0.086386699
SATB2	-0.114805092	0.027025042	0.045053798
SATL1	-0.237793874	3.63E-06	1.30E-05
SAV1	-0.29004359	1.27E-08	6.90E-08
SBDSP1	-0.023005055	0.658726685	0.720616826

SBDS	-0.257514346	4.95E-07	2.05E-06
SBF1P1	0.169666955	0.001035109	0.002355658
SBF1	0.184657547	0.000349494	0.000870762
SBF2	-0.209219329	4.88E-05	0.000142665
SBK1	0.346029528	7.11E-12	6.40E-11
SBK2	0.176830962	0.000622739	0.001482045
SBNO1	-0.030203269	0.561965048	0.632331702
SBNO2	0.26036404	3.66E-07	1.55E-06
SBSN	0.14955155	0.003888118	0.007852706
SC4MOL	-0.45611741	1.84E-20	5.99E-19
SC5DL	-0.399650724	1.17E-15	1.90E-14
SC65	0.35481163	1.90E-12	1.87E-11
SCAF1	0.149770117	0.003835703	0.00775156
SCAI	-0.112025185	0.030984571	0.050854722
SCAMP1	0.037848781	0.467336605	0.542241109
SCAMP2	0.094871583	0.067954517	0.102389191
SCAMP3	0.256658061	5.41E-07	2.22E-06
SCAMP4	0.274770969	7.51E-08	3.57E-07
SCAMP5	0.316767909	4.31E-10	2.94E-09
SCAND1	-0.045227098	0.385043888	0.461237709
SCAND2	-0.010769571	0.836210901	0.870284901
SCAND3	0.156865031	0.002445396	0.005172773
SCAPER	-0.234377297	5.05E-06	1.76E-05
SCAP	-0.005166415	0.920997709	0.938282606
SCARA3	0.123375068	0.01743318	0.030321618
SCARA5	0.145961698	0.004846917	0.009583195
SCARB1	-0.037126097	0.475887938	0.550495848
SCARB2	-0.186936657	0.000294069	0.000742515
SCARF1	-0.078300852	0.132220335	0.183152101
SCARF2	0.113015457	0.029520653	0.048745168
SCARNA10	0.012770087	0.80634089	0.845304249
SCARNA11	0.05687941	0.274498018	0.344844843
SCARNA12	0.179448675	0.000514686	0.00124361
SCARNA14	0.099910885	0.054512836	0.084245188
SCARNA15	0.090808946	0.080669928	0.118939188
SCARNA16	0.09581549	0.065248387	0.09872244
SCARNA17	-0.207320904	5.73E-05	0.00016543
SCARNA18	0.040325171	0.438687395	0.514513733
SCARNA1	0.130652201	0.011773791	0.0213603
SCARNA21	0.047554564	0.361036672	0.436627972
SCARNA22	0.093572911	0.071827304	0.107379825
SCARNA2	-0.054078735	0.298862696	0.371077759
SCARNA3	-0.026712546	0.608038581	0.673937054
SCARNA4	0.009598311	0.853811919	0.884983382
SCARNA5	0.021010869	0.686671048	0.744198706
SCARNA6	-0.022932571	0.659734421	0.721481535
SCARNA7	-0.126539971	0.014731286	0.026063648
SCARNA8	0.048536982	0.351190245	0.425909299
SCARNA9L	0.104370434	0.044534738	0.070403194
SCARNA9	0.024888615	0.632760595	0.697072361
SCCPDH	-0.03000421	0.564546633	0.634600111
SCD5	0.267101835	1.76E-07	7.90E-07
SCD	-0.08793267	0.090786232	0.131950341
SCEL	0.14317767	0.005731872	0.011128579
SCFD1	0.002776258	0.957497535	0.966570352
SCFD2	0.019922303	0.702110022	0.757756968
SCG2	0.343037437	1.10E-11	9.66E-11
SCG3	0.263173986	2.70E-07	1.17E-06

SCG5	0.200504586	0.000100858	0.000278373
SCGB1A1	-0.038150307	0.463793964	0.538726164
SCGB1C1	0.02239156	0.667275364	0.727760646
SCGB1D2	0.213192318	3.47E-05	0.000104122
SCGB2A1	0.342855055	1.13E-11	9.91E-11
SCGB2A2	0.096286367	0.063931868	0.096944031
SCGB3A1	-0.02322119	0.655725459	0.717885508
SCGB3A2	-0.041491466	0.425550686	0.501972927
SCGBL	-0.097062586	0.06180939	0.094069264
SCGN	0.209814809	4.64E-05	0.000136149
SCHIP1	-0.10440893	0.044455782	0.07030069
SCIN	0.264201734	2.42E-07	1.06E-06
SCLT1	0.258331901	4.54E-07	1.89E-06
SCLY	0.017325759	0.739423777	0.789009132
SCMH1	0.39351135	3.44E-15	5.19E-14
SCML1	-0.050330297	0.333658287	0.407731887
SCML2	0.232474095	6.05E-06	2.08E-05
SCML4	0.106119234	0.041064817	0.065505107
SCN10A	0.034449109	0.50829559	0.581234345
SCN11A	-0.133342646	0.010135108	0.018678225
SCN1A	-0.002771258	0.957574004	0.966598567
SCN1B	0.142263918	0.006052483	0.011698609
SCN2A	-0.249262787	1.16E-06	4.52E-06
SCN2B	0.009188808	0.859983314	0.89017229
SCN3A	0.043996311	0.398122922	0.474423067
SCN3B	0.300381788	3.57E-09	2.11E-08
SCN4A	-0.315397876	5.17E-10	3.49E-09
SCN4B	-0.380441816	3.19E-14	4.12E-13
SCN5A	0.193602056	0.00017542	0.00046257
SCN7A	-0.016715251	0.748290391	0.796594335
SCN8A	-0.123273211	0.017526893	0.030463336
SCN9A	0.008747655	0.866641203	0.895174265
SCNM1	0.36733005	2.70E-13	3.04E-12
SCNN1A	-0.036753445	0.480330586	0.554604494
SCNN1B	0.062158108	0.232335299	0.298791804
SCNN1D	-0.196545212	0.000138865	0.000373291
SCNN1G	0.032054748	0.538229358	0.610036142
SCO1	-0.210672224	4.31E-05	0.000127449
SCO2	0.088439569	0.088933104	0.129549964
SCOC	-0.032958368	0.526829817	0.598819171
SCP2	-0.437874013	8.24E-19	2.16E-17
SCPEP1	0.176455642	0.000639851	0.001520408
SCRG1	0.149406475	0.003923266	0.007911662
SCRIB	0.33890032	2.01E-11	1.69E-10
SCRN1	0.242561442	2.28E-06	8.42E-06
SCRN2	-0.245224445	1.75E-06	6.58E-06
SCRN3	-0.144704255	0.005230129	0.010249232
SCRT1	0.04291359	0.409846055	0.486038044
SCRT2	0.086889045	0.094698884	0.136937735
SCTR	0.202775809	8.37E-05	0.000234478
SCT	0.089517674	0.085092684	0.124646295
SCUBE1	-0.227393005	9.73E-06	3.22E-05
SCUBE2	0.022076234	0.671686111	0.731636281
SCUBE3	0.208453218	5.21E-05	0.000151452
SCXB	0.259824108	3.87E-07	1.63E-06
SCYL1	-0.113583459	0.028708023	0.047553108
SCYL2	-0.050852582	0.328659892	0.402728649
SCYL3	-0.033513776	0.51988454	0.592208806

SDAD1	-0.120135631	0.020637259	0.035342171
SDC1	-0.34731038	5.88E-12	5.36E-11
SDC2	-0.196702695	0.000137126	0.000368866
SDC3	0.311301033	8.85E-10	5.78E-09
SDC4P	-0.01248093	0.810642078	0.8487734
SDC4	0.006104171	0.906718236	0.9266732
SDCBP2	0.286948273	1.83E-08	9.69E-08
SDCBP	-0.09572295	0.065509718	0.099065174
SDCCAG1	0.003890497	0.940466399	0.954164371
SDCCAG3	-0.037530007	0.471098075	0.545905995
SDCCAG8	0.269535764	1.35E-07	6.15E-07
SDF2L1	0.065409361	0.208765133	0.272383193
SDF2	0.055987053	0.282109941	0.352916436
SDF4	-0.130325948	0.011987619	0.021702776
SDHAF1	-0.057733654	0.267343243	0.337061369
SDHAF2	0.180451021	0.000478132	0.001161061
SDHAP1	0.188667272	0.000257585	0.00065756
SDHAP2	0.129161779	0.012778832	0.02296616
SDHAP3	0.185015661	0.000340184	0.000849267
SDHA	-0.22550903	1.16E-05	3.78E-05
SDHB	-0.30576383	1.81E-09	1.12E-08
SDHC	-0.158311879	0.00222581	0.004744553
SDHD	-0.339238481	1.92E-11	1.61E-10
SDK1	0.079983104	0.124085455	0.173487995
SDK2	0.202881136	8.30E-05	0.000232515
SDPR	-0.407836758	2.66E-16	4.78E-15
SDR16C5	0.218037213	2.27E-05	7.02E-05
SDR39U1	0.038746118	0.456837772	0.531918299
SDR42E1	0.009407296	0.856689516	0.887471715
SDR9C7	-0.063684148	0.221047862	0.286290177
SDSL	0.078714781	0.130181508	0.180717442
SDS	0.016226323	0.755415305	0.802291486
SEBOX	-0.009186949	0.860011347	0.89017229
SEC11A	0.026891272	0.605639321	0.67183844
SEC11C	0.03097064	0.552066552	0.623343337
SEC13	0.062759778	0.227837311	0.293670736
SEC14L1	0.021397087	0.681224125	0.739582944
SEC14L2	-0.563867133	1.60E-32	3.98E-30
SEC14L3	-0.32432596	1.56E-10	1.14E-09
SEC14L4	0.000562114	0.991390563	0.993235242
SEC14L5	-0.118920972	0.021964252	0.037396105
SEC16A	-0.013202029	0.799926683	0.839747144
SEC16B	-0.307239278	1.50E-09	9.41E-09
SEC1	-0.028368445	0.585973218	0.654175313
SEC22A	0.09877443	0.057333425	0.088066962
SEC22B	-0.061154402	0.239977853	0.307251851
SEC22C	0.091733061	0.077620339	0.114970438
SEC23A	-0.249861396	1.09E-06	4.28E-06
SEC23B	0.167432112	0.001208074	0.002713332
SEC23IP	0.071253293	0.170828875	0.22880549
SEC24A	-0.088474476	0.088806615	0.129413019
SEC24B	-0.261466177	3.25E-07	1.39E-06
SEC24C	0.083852775	0.106851166	0.152299415
SEC24D	-0.163117944	0.001619285	0.003541096
SEC31A	-0.098159749	0.058908714	0.090155915
SEC31B	-0.234731776	4.88E-06	1.71E-05
SEC61A1	0.265903599	2.01E-07	8.91E-07
SEC61A2	0.019278584	0.711298223	0.76535151

SEC61B	0.052631737	0.311997861	0.384844367
SEC61G	0.257926281	4.74E-07	1.97E-06
SEC62	-0.310614246	9.68E-10	6.27E-09
SEC63	-0.030558221	0.557375825	0.627950075
SECISBP2L	-0.231155968	6.85E-06	2.33E-05
SECISBP2	-0.25433711	6.90E-07	2.79E-06
SECTM1	0.320856057	2.49E-10	1.77E-09
SEH1L	0.192725556	0.000187939	0.000492711
SEL1L2	0.017079596	0.7429948	0.792098379
SEL1L3	0.500284758	6.82E-25	4.33E-23
SEL1L	-0.286644354	1.90E-08	1.00E-07
SELENBP1	-0.497920774	1.22E-24	7.43E-23
SELE	-0.165415884	0.001386543	0.003081516
SELK	0.017831124	0.732110126	0.783008425
SELL	0.070235268	0.177038677	0.235933141
SELM	0.440104529	5.24E-19	1.41E-17
SELO	-0.412390044	1.15E-16	2.21E-15
SELPLG	0.227557117	9.58E-06	3.18E-05
SELP	0.006494156	0.900788378	0.9219738
SELS	0.168299173	0.001138033	0.002571677
SELT	-0.055401074	0.287185255	0.358635031
SELV	0.059881501	0.249921642	0.31808209
SEMA3A	0.223432072	1.40E-05	4.49E-05
SEMA3B	-0.036356537	0.48508707	0.559189018
SEMA3C	0.324565459	1.51E-10	1.10E-09
SEMA3D	-0.01308847	0.801611726	0.841204969
SEMA3E	0.308155943	1.33E-09	8.43E-09
SEMA3F	0.043164177	0.407114809	0.483403339
SEMA3G	-0.121035104	0.019699998	0.033865162
SEMA4A	0.437739686	8.46E-19	2.21E-17
SEMA4B	0.057047102	0.273083323	0.343240754
SEMA4C	0.251885354	8.89E-07	3.52E-06
SEMA4D	0.461542267	5.69E-21	2.02E-19
SEMA4F	0.210784411	4.27E-05	0.000126343
SEMA4G	-0.099868084	0.054616935	0.084392972
SEMA5A	0.122920106	0.017855179	0.030982569
SEMA5B	0.249216431	1.17E-06	4.54E-06
SEMA6A	0.368214227	2.35E-13	2.67E-12
SEMA6B	0.067959903	0.1915208	0.252513845
SEMA6C	-0.075915499	0.144452797	0.197695314
SEMA6D	0.02832445	0.586554651	0.654486066
SEMA7A	-0.11363737	0.028631899	0.047442792
SEMG1	0.222388744	1.54E-05	4.90E-05
SEMG2	0.0457454	0.379615304	0.455709831
SENP1	0.277215041	5.69E-08	2.76E-07
SENP2	-0.180491195	0.000476718	0.001157911
SENP3	0.151304396	0.0034854	0.007107076
SENP5	0.080692062	0.120775909	0.169443497
SENP6	0.144626227	0.005254783	0.010292576
SENP7	0.026443183	0.611662546	0.677463792
SENP8	-0.412649619	1.10E-16	2.11E-15
15-Sep	0.009809491	0.850632798	0.882114757
SEPHS1	0.244019422	1.97E-06	7.36E-06
SEPHS2	-0.19226509	0.000194846	0.000509412
SEPN1	0.344776997	8.55E-12	7.62E-11
SEPP1	-0.470095778	8.54E-22	3.38E-20
SEPSECS	-0.488771978	1.13E-23	5.90E-22
10-Sep	-0.198182484	0.000121756	0.000330827

11-Sep	0.071674521	0.168307512	0.225792745
12-Sep	0.030802618	0.554226592	0.625240877
14-Sep	0.182801796	0.000401654	0.000988724
1-Sep	0.222099266	1.58E-05	5.02E-05
2-Sep	0.345007274	8.27E-12	7.39E-11
3-Sep	0.291930591	1.01E-08	5.57E-08
4-Sep	-0.456074848	1.86E-20	6.03E-19
5-Sep	0.362746856	5.58E-13	5.94E-12
6-Sep	0.208999125	4.97E-05	0.000145216
SEPT7L	-0.045925588	0.377739096	0.453934025
SEPT7P2	0.009834281	0.850259775	0.881819733
7-Sep	0.167164009	0.001230521	0.002761154
8-Sep	0.443804142	2.46E-19	6.86E-18
9-Sep	0.359549602	9.18E-13	9.43E-12
SEPW1	0.307015172	1.54E-09	9.66E-09
SEPX1	-0.249698175	1.11E-06	4.34E-06
SERAC1	0.270878373	1.16E-07	5.35E-07
SERBP1	-0.028022366	0.59055415	0.658146105
SERF1A	0.338773891	2.05E-11	1.71E-10
SERF2	0.085257236	0.10108517	0.14514009
SERGEF	0.206606148	6.09E-05	0.000174974
SERHL2	0.247143769	1.44E-06	5.51E-06
SERHL	0.192156874	0.000196503	0.000513408
SERINC1	-0.253061677	7.87E-07	3.15E-06
SERINC2	-0.165603353	0.001368981	0.003044524
SERINC3	-0.283194426	2.85E-08	1.46E-07
SERINC4	0.24849012	1.26E-06	4.86E-06
SERINC5	-0.176270934	0.000648431	0.001539695
SERP1	-0.127859902	0.013717831	0.024477083
SERP2	-0.0532687	0.306169826	0.378710746
SERPINA10	-0.39358366	3.39E-15	5.13E-14
SERPINA11	-0.311121532	9.06E-10	5.90E-09
SERPINA12	0.085782149	0.098994607	0.142436123
SERPINA13	0.142139883	0.006097222	0.011781654
SERPINA1	-0.096307148	0.063874275	0.096878821
SERPINA3	-0.040556875	0.436059248	0.512245553
SERPINA4	-0.356056238	1.57E-12	1.56E-11
SERPINA5	-0.195429316	0.000151791	0.000405141
SERPINA6	-0.228718903	8.60E-06	2.88E-05
SERPINA7	-0.069986367	0.178582109	0.237628071
SERPINA9	-0.004761406	0.927173021	0.943223251
SERPINB10	0.103588368	0.046164427	0.072685349
SERPINB11	0.204525518	7.24E-05	0.000205129
SERPINB12	0.028974088	0.577996277	0.647082992
SERPINB13	0.013568492	0.794495322	0.835644148
SERPINB1	0.241218447	2.60E-06	9.54E-06
SERPINB2	0.135016735	0.00922065	0.017153216
SERPINB3	0.1124345	0.030372123	0.049993674
SERPINB4	0.126080753	0.01509898	0.026647934
SERPINB5	0.050316975	0.333786419	0.407781508
SERPINB6	0.154780602	0.002796551	0.005825271
SERPINB7	0.265165057	2.18E-07	9.61E-07
SERPINB8	0.146020113	0.004829745	0.009555883
SERPINB9	0.220738894	1.78E-05	5.63E-05
SERPINC1	-0.475949973	2.26E-22	9.82E-21
SERPIND1	-0.48722702	1.63E-23	8.33E-22
SERPINE1	0.083970494	0.106358119	0.151748653
SERPINE2	0.344076584	9.48E-12	8.37E-11

SERPINE3	0.114396048	0.027578861	0.045865771
SERPINF1	-0.322865538	1.90E-10	1.37E-09
SERPINF2	-0.271212673	1.12E-07	5.16E-07
SERPING1	-0.453320473	3.35E-20	1.04E-18
SERPINH1	0.457236584	1.45E-20	4.80E-19
SERPINI1	0.104979609	0.043299063	0.068591279
SERPINI2	0.098889352	0.057042812	0.087665425
SERTAD1	0.036868485	0.478956715	0.553210556
SERTAD2	-0.174161384	0.000754282	0.001764953
SERTAD3	0.191186134	0.000211972	0.00055043
SERTAD4	-0.029237539	0.574542307	0.643758821
SESN1	-0.172478344	0.000849957	0.001969622
SESN2	-0.102547047	0.048411725	0.075929648
SESN3	-0.102269997	0.049024814	0.076716075
SESTD1	0.194282776	0.000166241	0.000440465
SETBP1	-0.093281788	0.072719722	0.108567319
SETD1A	0.080404426	0.122110258	0.171062552
SETD1B	0.073502918	0.15768499	0.213410352
SETD2	-0.007435548	0.886497541	0.91049152
SETD3	-0.198321861	0.000120395	0.000327665
SETD4	0.313641055	6.52E-10	4.33E-09
SETD5	0.143546641	0.005606793	0.010911256
SETD6	-0.152809762	0.003170138	0.006531749
SETD7	-0.37816575	4.65E-14	5.88E-13
SETD8	0.395711394	2.34E-15	3.62E-14
SETDB1	0.225729896	1.13E-05	3.71E-05
SETDB2	-0.161379777	0.00181855	0.003944811
SETMAR	0.06629167	0.202677351	0.265500933
SETX	0.008725096	0.866981925	0.895479776
SET	0.352828627	2.57E-12	2.48E-11
SEZ6L2	0.217738487	2.33E-05	7.19E-05
SEZ6L	0.192543668	0.000190639	0.0004992
SEZ6	0.155129765	0.002734703	0.005712567
SF1	0.153158525	0.003100895	0.006400334
SF3A1	-0.08736155	0.092911058	0.134655471
SF3A2	0.407301154	2.94E-16	5.24E-15
SF3A3	0.185110867	0.000337749	0.000843927
SF3B14	0.171724826	0.000896324	0.00206482
SF3B1	0.071091493	0.17180482	0.229865388
SF3B2	0.171652761	0.000900878	0.002074113
SF3B3	0.026798144	0.60688896	0.67277513
SF3B4	0.48296793	4.45E-23	2.11E-21
SF3B5	0.126453069	0.014800259	0.026164935
SF4	0.156795271	0.002456466	0.005193433
SFI1	0.401795778	7.95E-16	1.33E-14
SFMBT1	-0.070066427	0.178084575	0.237120766
SFMBT2	0.128096641	0.013542695	0.024203603
SFN	0.277740795	5.36E-08	2.61E-07
SFPQ	0.464003094	3.31E-21	1.22E-19
SFRP1	0.021446361	0.680530382	0.738990857
SFRP2	0.163935006	0.001532712	0.003372136
SFRP4	0.128705415	0.013101337	0.023486442
SFRP5	0.273924448	8.27E-08	3.89E-07
SFRS11	0.162335952	0.001706317	0.003717952
SFRS12IP1	0.253711445	7.36E-07	2.96E-06
SFRS12	0.14093688	0.006546775	0.012580771
SFRS13A	0.22706186	1.00E-05	3.31E-05
SFRS13B	0.521410308	2.99E-27	2.82E-25

SFRS14	0.256332207	5.60E-07	2.29E-06
SFRS15	0.105292162	0.04267635	0.067739482
SFRS16	0.159196065	0.002100636	0.004503268
SFRS17A	0.238182207	3.50E-06	1.25E-05
SFRS18	0.21316565	3.48E-05	0.000104347
SFRS1	0.436458415	1.10E-18	2.81E-17
SFRS2B	-0.123724053	0.017115407	0.029828812
SFRS2IP	0.042616866	0.413094167	0.489278358
SFRS2	0.398338428	1.47E-15	2.35E-14
SFRS3	0.414502807	7.76E-17	1.52E-15
SFRS4	0.0603567	0.246176416	0.314057796
SFRS5	-0.097753226	0.059970071	0.091605869
SFRS6	0.19026626	0.000227674	0.00058737
SFRS7	0.40290882	6.51E-16	1.10E-14
SFRS8	0.355589609	1.69E-12	1.67E-11
SFRS9	0.432495799	2.42E-18	5.89E-17
SFT2D1	0.406894561	3.16E-16	5.62E-15
SFT2D2	0.237976293	3.57E-06	1.28E-05
SFT2D3	-0.080235937	0.12289719	0.172043976
SFTA1P	-0.028360856	0.586073486	0.654216893
SFTA2	0.138915162	0.007369478	0.014003695
SFTA3	-0.044791799	0.389639418	0.466070038
SFTPA1	0.02466117	0.635873056	0.700114017
SFTPA2	0.169884189	0.001019575	0.002324169
SFTPB	0.028276731	0.587185596	0.655014919
SFTPC	-0.044184597	0.396104994	0.472414015
SFTPD	-0.108406618	0.036873005	0.059427556
SFXN1	-0.340800333	1.53E-11	1.31E-10
SFXN2	-0.372128459	1.25E-13	1.49E-12
SFXN3	0.453281084	3.38E-20	1.05E-18
SFXN4	0.137729662	0.007893765	0.014901884
SFXN5	-0.311344933	8.80E-10	5.75E-09
SGCA	0.13901088	0.007328536	0.013929881
SGCB	-0.01243053	0.811392364	0.849425048
SGCD	0.042580838	0.413489583	0.489619991
SGCE	0.122318395	0.018426967	0.031896954
SGCG	0.121236748	0.019495036	0.033556256
SGCZ	-0.030154128	0.56260182	0.632835721
SGEF	-0.213138366	3.49E-05	0.000104578
SGIP1	0.281672253	3.40E-08	1.72E-07
SGK196	0.041635998	0.423938816	0.500278942
SGK1	0.019704517	0.705213849	0.760200762
SGK223	-0.016658251	0.749119935	0.79724235
SGK269	0.075283605	0.147833685	0.201791207
SGK2	0.009619143	0.85349821	0.884833755
SGK3	-0.107314542	0.038826724	0.062213947
SGK494	0.309072962	1.18E-09	7.54E-09
SGMS1	-0.094581099	0.068805583	0.10345405
SGMS2	-0.065318773	0.209397563	0.273083154
SGOL1	0.521181329	3.18E-27	2.99E-25
SGOL2	0.509924814	6.00E-26	4.63E-24
SGPL1	-0.264176824	2.43E-07	1.06E-06
SGPP1	-0.053397129	0.305003498	0.377606822
SGPP2	0.427665892	6.26E-18	1.43E-16
SGSH	0.272621294	9.57E-08	4.46E-07
SGSM1	0.338715475	2.07E-11	1.73E-10
SGSM2	0.111013274	0.032544145	0.053169137
SGSM3	0.105737599	0.04180193	0.066515818

SGTA	0.167516015	0.001201127	0.002701079
SGTB	0.279601637	4.33E-08	2.15E-07
SH2B1	0.097509523	0.060613874	0.092454211
SH2B2	0.231065784	6.90E-06	2.35E-05
SH2B3	0.054903337	0.291544208	0.363218408
SH2D1A	0.126512046	0.01475342	0.026098173
SH2D1B	-0.076100835	0.143472452	0.196592489
SH2D2A	0.338288994	2.20E-11	1.83E-10
SH2D3A	0.327861071	9.57E-11	7.24E-10
SH2D3C	0.036609166	0.482056676	0.556242867
SH2D4A	-0.232809436	5.86E-06	2.02E-05
SH2D4B	-0.037984235	0.465743309	0.540644343
SH2D5	0.281865009	3.33E-08	1.69E-07
SH2D6	0.172759817	0.000833213	0.00193352
SH2D7	0.158372362	0.002217035	0.004727366
SH3BGRL2	-0.37412758	9.03E-14	1.09E-12
SH3BGRL3	0.41463048	7.58E-17	1.49E-15
SH3BGRL	0.076834582	0.139641103	0.192108974
SH3BGR	-0.183745736	0.000374279	0.000926834
SH3BP1	0.513250024	2.55E-26	2.10E-24
SH3BP2	0.083614003	0.107856764	0.153545981
SH3BP4	-0.384295395	1.67E-14	2.25E-13
SH3BP5L	0.144241898	0.005377751	0.010504516
SH3BP5	-0.303116116	2.53E-09	1.53E-08
SH3D19	-0.536443929	4.94E-29	6.04E-27
SH3D20	0.128264705	0.01341956	0.023989993
SH3GL1	0.237858249	3.61E-06	1.29E-05
SH3GL2	0.200141948	0.000103883	0.000285732
SH3GL3	-0.171448419	0.000913909	0.002100473
SH3GLB1	0.178473978	0.000552704	0.001327743
SH3GLB2	0.280429112	3.93E-08	1.97E-07
SH3KBP1	0.188054197	0.000269996	0.000687132
SH3PXD2A	0.186402379	0.000306272	0.00077147
SH3PXD2B	0.362849337	5.49E-13	5.86E-12
SH3RF1	0.130319698	0.011991749	0.02170828
SH3RF2	-0.134357185	0.009571784	0.017746774
SH3RF3	0.043460036	0.403904078	0.48009852
SH3TC1	0.242806925	2.22E-06	8.23E-06
SH3TC2	-0.06828798	0.189380728	0.249956464
SH3YL1	0.037158115	0.475507273	0.55018329
SHANK1	0.115771056	0.025755009	0.04316407
SHANK2	-0.098510135	0.058006424	0.088961046
SHANK3	-0.230823578	7.06E-06	2.40E-05
SHARPIN	0.230305839	7.41E-06	2.51E-05
SHBG	-0.026547164	0.610262469	0.676138641
SHB	-0.305587817	1.85E-09	1.15E-08
SHC1	0.126085768	0.015094921	0.026643131
SHC2	-0.197854616	0.000125015	0.000339127
SHC3	0.089705943	0.084435939	0.123786355
SHC4	0.052166586	0.316299571	0.389547481
SHCBP1	0.535350197	6.71E-29	8.03E-27
SHD	-0.1586563	0.002176263	0.004646394
SHE	-0.022798257	0.661603409	0.723088825
SHFM1	0.03245864	0.533118884	0.605001106
SHF	-0.33009654	7.02E-11	5.44E-10
SHH	-0.384322691	1.66E-14	2.24E-13
SHISA2	0.305302581	1.92E-09	1.18E-08
SHISA3	0.007322452	0.888212545	0.911923846

SHISA4	-0.212868459	3.57E-05	0.000106954
SHISA5	0.019113502	0.713661353	0.767355434
SHISA6	-0.176790788	0.00062455	0.001486178
SHISA7	0.029239188	0.574520729	0.643758821
SHISA9	0.238407922	3.42E-06	1.23E-05
SHKBP1	0.357220255	1.32E-12	1.32E-11
SHMT1	-0.293993372	7.85E-09	4.42E-08
SHMT2	-0.112524548	0.030238786	0.049807118
SHOC2	-0.028842632	0.579723355	0.648469887
SHOX2	0.265423694	2.12E-07	9.36E-07
SHOX	-0.017635354	0.734940476	0.785024083
SHPK	-0.321941763	2.15E-10	1.54E-09
SHPRH	-0.00959623	0.853843268	0.884983382
SHQ1	0.031608488	0.543904219	0.615312882
SHROOM1	-0.128020633	0.013598709	0.024291218
SHROOM2	0.023557179	0.651070943	0.713848945
SHROOM3	-0.055861901	0.283188799	0.354110422
SHROOM4	-0.131409983	0.011290086	0.020555756
SIAE	-0.341366263	1.41E-11	1.21E-10
SIAH1	-0.00067832	0.989610824	0.99202353
SIAH2	-0.320593133	2.58E-10	1.83E-09
SIAH3	0.193992387	0.0001701	0.000449852
SIDT1	0.136769079	0.00834272	0.015657435
SIDT2	-0.39430275	2.99E-15	4.58E-14
SIGIRR	0.0594543	0.253322259	0.321957642
SIGLEC10	0.378370382	4.50E-14	5.69E-13
SIGLEC11	-0.011345433	0.827586462	0.8629771
SIGLEC12	0.172861902	0.000827216	0.001920498
SIGLEC14	0.076488975	0.141435847	0.194229333
SIGLEC15	-0.034917591	0.502542142	0.575911445
SIGLEC16	-0.021026507	0.686450187	0.744080778
SIGLEC1	0.106465954	0.04040468	0.064534758
SIGLEC5	0.214795189	3.02E-05	9.15E-05
SIGLEC6	0.059042486	0.256630641	0.325601555
SIGLEC7	0.168508516	0.001121693	0.002537917
SIGLEC8	0.22763761	9.51E-06	3.15E-05
SIGLEC9	0.260958639	3.43E-07	1.46E-06
SIGLECP3	-0.012657318	0.80801763	0.846648463
SIGMAR1	-0.235843341	4.38E-06	1.55E-05
SIK1	-0.143831467	0.005511922	0.010742371
SIK2	-0.412462679	1.14E-16	2.18E-15
SIK3	-0.200676393	9.95E-05	0.000274803
SIKE1	-0.049497467	0.341729159	0.416005396
SIL1	-0.109320706	0.035302429	0.057150577
SILV	-0.406670349	3.29E-16	5.84E-15
SIM1	0.01548451	0.766264896	0.811583856
SIM2	0.080332982	0.122443459	0.171469042
SIN3A	-0.001794733	0.972516423	0.97826166
SIN3B	0.221425921	1.68E-05	5.31E-05
SIP1	0.249937505	1.09E-06	4.25E-06
SIPA1L1	-0.133370016	0.010119533	0.018652973
SIPA1L2	0.148569933	0.004131604	0.008284832
SIPA1L3	0.313013884	7.08E-10	4.67E-09
SIPA1	0.120504812	0.020247984	0.034732248
SIRPA	0.207946714	5.44E-05	0.000157488
SIRPB1	0.114413338	0.027555256	0.045834966
SIRPB2	0.157087819	0.002410346	0.005105136
SIRPD	0.076231141	0.142786245	0.195813834

SIRPG	0.280915237	3.72E-08	1.87E-07
SIRT1	-0.22046584	1.83E-05	5.75E-05
SIRT2	0.046309273	0.373762961	0.449707239
SIRT3	-0.316705712	4.35E-10	2.97E-09
SIRT4	-0.003259764	0.950104313	0.961250222
SIRT5	-0.421351239	2.12E-17	4.52E-16
SIRT6	0.313618378	6.54E-10	4.34E-09
SIRT7	0.077687622	0.135285996	0.186814141
SIT1	0.22950053	8.00E-06	2.69E-05
SIVA1	0.020354403	0.695966522	0.752390116
SIX1	0.166130969	0.001320646	0.00294492
SIX2	0.272748003	9.43E-08	4.40E-07
SIX3	0.231594747	6.57E-06	2.25E-05
SIX4	0.212836561	3.58E-05	0.000107218
SIX5	0.213932199	3.25E-05	9.80E-05
SIX6	0.019546613	0.70746732	0.761956522
SI	0.12013015	0.020643087	0.035349114
SKA1	0.551029267	7.55E-31	1.24E-28
SKA2	0.231151531	6.85E-06	2.33E-05
SKA3	0.570780751	1.87E-33	5.82E-31
SKAP1	0.032354652	0.534432311	0.606318936
SKAP2	0.053092814	0.307771905	0.380456186
SKIL	0.167917528	0.001168389	0.002635474
SKINTL	0.016377038	0.753216766	0.80063441
SKIV2L2	0.179348254	0.000518487	0.001251734
SKIV2L	-0.044715749	0.390445695	0.466838262
SKI	-0.111339698	0.032033895	0.052441503
SKP1	0.23400381	5.23E-06	1.82E-05
SKP2	0.128675657	0.013122612	0.023516118
SLA2	0.198315211	0.000120459	0.000327796
SLAIN1	0.081853751	0.11550215	0.162962736
SLAIN2	-0.148837874	0.004063811	0.008162859
SLAMF1	0.208299166	5.28E-05	0.000153285
SLAMF6	0.098378883	0.058343065	0.089415381
SLAMF7	0.244229555	1.93E-06	7.22E-06
SLAMF8	0.34118459	1.45E-11	1.24E-10
SLAMF9	-0.020829504	0.689234509	0.746363784
SLA	0.197677757	0.000126807	0.000343286
SLBP	0.457789298	1.28E-20	4.29E-19
SLC10A1	-0.446741713	1.34E-19	3.86E-18
SLC10A2	0.172664291	0.000838861	0.001945494
SLC10A3	0.310172631	1.03E-09	6.62E-09
SLC10A4	0.13011547	0.012127381	0.021931889
SLC10A5	-0.31348527	6.65E-10	4.40E-09
SLC10A6	-0.175911836	0.000665418	0.001575337
SLC10A7	-0.101884343	0.04988903	0.077866598
SLC11A1	0.083087732	0.110099524	0.156292056
SLC11A2	-0.096369216	0.06370251	0.096653876
SLC12A1	-0.299181653	4.14E-09	2.43E-08
SLC12A2	0.246042338	1.61E-06	6.10E-06
SLC12A3	0.172098351	0.000873055	0.002016814
SLC12A4	-0.236421565	4.15E-06	1.47E-05
SLC12A5	0.194283785	0.000166228	0.000440465
SLC12A6	-0.045603443	0.381097452	0.457307761
SLC12A7	0.176696994	0.000628797	0.001495776
SLC12A8	0.319555898	2.97E-10	2.08E-09
SLC12A9	0.300680875	3.44E-09	2.04E-08
SLC13A1	0.06203786	0.233241734	0.299783344

SLC13A2	0.252328844	8.49E-07	3.37E-06
SLC13A3	-0.314874636	5.54E-10	3.71E-09
SLC13A4	0.094747112	0.068318133	0.102885155
SLC13A5	-0.41809777	3.95E-17	8.05E-16
SLC14A1	-0.066298007	0.202634097	0.265468941
SLC14A2	-0.271315431	1.11E-07	5.11E-07
SLC15A1	0.156179716	0.002556145	0.005387023
SLC15A2	0.19138164	0.000208768	0.000543101
SLC15A3	0.124468627	0.016454301	0.02878744
SLC15A4	0.253522418	7.51E-07	3.01E-06
SLC16A10	-0.052561946	0.312640827	0.385494248
SLC16A11	-0.364544468	4.20E-13	4.58E-12
SLC16A12	-0.35152304	3.13E-12	2.98E-11
SLC16A13	-0.254670129	6.66E-07	2.70E-06
SLC16A14	0.080937977	0.119644142	0.16802133
SLC16A1	-0.297673615	5.00E-09	2.89E-08
SLC16A2	-0.434545461	1.61E-18	4.05E-17
SLC16A3	0.449166961	8.05E-20	2.40E-18
SLC16A4	-0.209899083	4.61E-05	0.000135313
SLC16A5	0.195887796	0.000146349	0.000391614
SLC16A6	0.131585815	0.011180393	0.020380253
SLC16A7	-0.091982566	0.076813201	0.113893465
SLC16A8	0.110676758	0.033077412	0.053943041
SLC16A9	0.359566124	9.16E-13	9.41E-12
SLC17A1	-0.17981002	0.000501219	0.001213131
SLC17A2	-0.401832886	7.90E-16	1.32E-14
SLC17A3	-0.232493343	6.03E-06	2.08E-05
SLC17A4	-0.280080395	4.09E-08	2.04E-07
SLC17A5	0.07646293	0.141571815	0.194362462
SLC17A6	0.076283879	0.14250923	0.195460853
SLC17A7	-0.124426756	0.016490876	0.028843831
SLC17A8	0.155706833	0.002635208	0.005533159
SLC17A9	0.212048096	3.83E-05	0.000114223
SLC18A1	0.132229324	0.010786937	0.019737083
SLC18A2	0.008976435	0.863187235	0.892485785
SLC18A3	0.096558454	0.063181157	0.095959061
SLC19A1	-0.074425874	0.152518809	0.207345474
SLC19A2	-0.154937074	0.002768679	0.005773854
SLC19A3	-0.141839504	0.006206798	0.011981767
SLC1A1	-0.2484703	1.26E-06	4.87E-06
SLC1A2	-0.462299572	4.82E-21	1.72E-19
SLC1A3	0.127860687	0.013717247	0.024477083
SLC1A4	0.231221519	6.80E-06	2.32E-05
SLC1A5	0.473430948	4.02E-22	1.67E-20
SLC1A6	0.108124176	0.037370123	0.060104165
SLC1A7	0.255072344	6.39E-07	2.60E-06
SLC20A1	0.258559921	4.43E-07	1.85E-06
SLC20A2	-0.381698133	2.59E-14	3.40E-13
SLC22A10	-0.111194006	0.032260783	0.052775441
SLC22A11	-0.111941943	0.031110408	0.051040233
SLC22A12	-0.179493873	0.000512983	0.001239797
SLC22A13	0.123386309	0.017422864	0.030308967
SLC22A14	0.065925226	0.205189924	0.268368806
SLC22A15	0.414416375	7.89E-17	1.54E-15
SLC22A16	0.076315414	0.142343787	0.195287716
SLC22A17	0.161344381	0.001822831	0.003952807
SLC22A18A	-0.031781496	0.541700653	0.613237891
SLC22A18	-0.085685044	0.099378735	0.14294752

SLC22A1	-0.293364872	8.47E-09	4.75E-08
SLC22A20	0.194788439	0.000159716	0.000424475
SLC22A23	0.123399191	0.017411049	0.030293705
SLC22A24	-0.038886647	0.455205621	0.530491326
SLC22A25	-0.257074468	5.18E-07	2.14E-06
SLC22A2	-0.180295729	0.000483633	0.001173274
SLC22A3	-0.446964476	1.28E-19	3.70E-18
SLC22A4	0.00145193	0.977764405	0.982597986
SLC22A5	0.385722673	1.31E-14	1.80E-13
SLC22A6	0.067162885	0.196793527	0.258643663
SLC22A7	-0.139097447	0.007291683	0.013867772
SLC22A8	0.113851189	0.028331687	0.046992245
SLC22A9	-0.093185858	0.073015764	0.108976629
SLC23A1	0.040451954	0.437248218	0.513162734
SLC23A2	-0.366851116	2.92E-13	3.26E-12
SLC23A3	-0.187348463	0.000284975	0.000722298
SLC24A1	-0.205928922	6.44E-05	0.000184268
SLC24A2	0.172214659	0.000865924	0.002001967
SLC24A3	0.257525979	4.94E-07	2.05E-06
SLC24A4	0.066780378	0.199361294	0.261724922
SLC24A5	0.187037204	0.000291824	0.000737659
SLC24A6	0.205183592	6.86E-05	0.000195159
SLC25A10	-0.316094407	4.72E-10	3.20E-09
SLC25A11	-0.055366288	0.287488464	0.358968673
SLC25A12	-0.000848105	0.987010586	0.990191605
SLC25A13	-0.487848823	1.40E-23	7.26E-22
SLC25A14	0.058640975	0.259884912	0.329206283
SLC25A15	-0.315843801	4.88E-10	3.30E-09
SLC25A16	0.067050386	0.197546215	0.259547299
SLC25A17	-0.10553834	0.042191202	0.067049548
SLC25A18	-0.033743486	0.517025872	0.589559237
SLC25A19	0.493749603	3.39E-24	1.91E-22
SLC25A1	-0.241273215	2.59E-06	9.49E-06
SLC25A20	-0.293380737	8.46E-09	4.75E-08
SLC25A21	0.078184197	0.132799355	0.18387745
SLC25A22	0.126584065	0.014696398	0.026018096
SLC25A23	-0.192184732	0.000196075	0.000512424
SLC25A24	0.254693174	6.65E-07	2.69E-06
SLC25A25	-0.25670146	5.39E-07	2.21E-06
SLC25A26	-0.219069604	2.07E-05	6.45E-05
SLC25A27	-0.139280997	0.007214091	0.013730086
SLC25A28	-0.169697984	0.001032876	0.002351559
SLC25A29	0.13212873	0.010847623	0.019842653
SLC25A2	0.035084099	0.500505549	0.574172034
SLC25A30	-0.35076531	3.51E-12	3.31E-11
SLC25A31	0.023951528	0.645625194	0.708853409
SLC25A32	0.042859508	0.41043694	0.48665187
SLC25A33	-0.133994373	0.009769959	0.01808391
SLC25A34	-0.385671254	1.32E-14	1.81E-13
SLC25A35	0.310627622	9.67E-10	6.26E-09
SLC25A36	0.259756996	3.90E-07	1.64E-06
SLC25A37	-0.128353976	0.013354556	0.023883572
SLC25A38	-0.190091069	0.000230785	0.000594864
SLC25A39	0.274894812	7.41E-08	3.52E-07
SLC25A3	0.199073429	0.000113298	0.000309577
SLC25A40	0.036381334	0.484789171	0.559007245
SLC25A41	0.008660795	0.867953221	0.896204212
SLC25A42	-0.432934882	2.22E-18	5.43E-17

SLC25A43	-0.043402112	0.404531494	0.480708406
SLC25A44	-0.175728377	0.000674255	0.001593985
SLC25A45	0.229598628	7.92E-06	2.67E-05
SLC25A46	0.000481392	0.992626875	0.994247294
SLC25A4	-0.280596298	3.86E-08	1.93E-07
SLC25A5	0.114525217	0.027402931	0.045615073
SLC25A6	0.366596271	3.04E-13	3.39E-12
SLC26A10	0.187097775	0.000290479	0.000734596
SLC26A11	0.296308499	5.91E-09	3.39E-08
SLC26A1	-0.277936499	5.24E-08	2.56E-07
SLC26A2	0.367543524	2.61E-13	2.95E-12
SLC26A3	-0.049294704	0.343712795	0.418035703
SLC26A4	-0.025609126	0.622943656	0.687924226
SLC26A5	-0.128244737	0.013434139	0.0240139
SLC26A6	0.301083878	3.27E-09	1.95E-08
SLC26A7	0.021299897	0.682593269	0.74070609
SLC26A8	-0.258362542	4.52E-07	1.88E-06
SLC26A9	0.412941596	1.04E-16	2.00E-15
SLC27A1	-0.110398417	0.033524102	0.054586609
SLC27A2	-0.281440408	3.50E-08	1.77E-07
SLC27A3	0.143443704	0.005641439	0.010970107
SLC27A4	-0.079593323	0.125934809	0.175666802
SLC27A5	-0.563591011	1.74E-32	4.28E-30
SLC27A6	0.161280926	0.00183053	0.003968206
SLC28A1	-0.362019326	6.25E-13	6.60E-12
SLC28A2	-0.101879563	0.049899822	0.07787734
SLC28A3	0.270236719	1.25E-07	5.72E-07
SLC29A1	-0.022514851	0.665553873	0.726171436
SLC29A2	0.320297196	2.69E-10	1.89E-09
SLC29A3	0.072626969	0.162709139	0.219345748
SLC29A4	0.262327161	2.96E-07	1.28E-06
SLC2A10	-0.167445496	0.001206963	0.002711615
SLC2A11	-0.119031981	0.021839982	0.03721633
SLC2A12	-0.282072198	3.25E-08	1.65E-07
SLC2A13	0.137985918	0.007777697	0.014700881
SLC2A14	0.01619893	0.755815111	0.802630524
SLC2A1	0.320079119	2.77E-10	1.95E-09
SLC2A2	-0.524176251	1.43E-27	1.40E-25
SLC2A3	0.057319886	0.270792681	0.340705557
SLC2A4RG	-0.157409254	0.002360583	0.00500879
SLC2A4	-0.307358268	1.47E-09	9.28E-09
SLC2A5	0.178234922	0.000562418	0.001349614
SLC2A6	0.481994422	5.59E-23	2.63E-21
SLC2A7	0.175948115	0.000663684	0.001572351
SLC2A8	-0.08921694	0.086150276	0.126039935
SLC2A9	-0.031580804	0.544257236	0.615677284
SLC30A10	-0.310399837	9.96E-10	6.44E-09
SLC30A1	-0.374196104	8.93E-14	1.08E-12
SLC30A2	0.053958898	0.299936348	0.372248438
SLC30A3	0.053737118	0.301930087	0.374466238
SLC30A4	-0.039212772	0.45143053	0.527188188
SLC30A5	0.122026454	0.018710096	0.03232806
SLC30A6	0.056365379	0.278865538	0.349516153
SLC30A7	0.032156443	0.536940293	0.608748266
SLC30A8	0.19112294	0.000213017	0.000553001
SLC30A9	0.047004742	0.36662186	0.442388898
SLC31A1	-0.399482395	1.20E-15	1.95E-14
SLC31A2	-0.215667275	2.80E-05	8.51E-05

SLC32A1	0.088620801	0.088277968	0.128708553
SLC33A1	-0.144755602	0.005213962	0.010220659
SLC34A1	-0.239395511	3.11E-06	1.13E-05
SLC34A2	0.32536484	1.35E-10	9.95E-10
SLC34A3	0.24460134	1.86E-06	6.97E-06
SLC35A1	-0.047497064	0.36161826	0.437251698
SLC35A2	0.220897974	1.76E-05	5.55E-05
SLC35A3	-0.274320597	7.90E-08	3.74E-07
SLC35A4	0.243321099	2.11E-06	7.84E-06
SLC35A5	-0.323761347	1.68E-10	1.23E-09
SLC35B1	0.110096922	0.034013739	0.055298001
SLC35B2	0.181118969	0.000455128	0.001109393
SLC35B3	-0.067687205	0.193313078	0.254590013
SLC35B4	-0.182678234	0.000405372	0.000997261
SLC35C1	0.052096064	0.316955126	0.390185998
SLC35C2	-0.009055502	0.861994136	0.891576095
SLC35D1	-0.383420943	1.94E-14	2.58E-13
SLC35D2	-0.011703687	0.822231395	0.858741486
SLC35D3	0.116913482	0.024319624	0.040996408
SLC35E1	0.039873985	0.443830987	0.519506518
SLC35E2	-0.015973231	0.759111694	0.80561598
SLC35E3	0.096862693	0.062350343	0.09481288
SLC35E4	0.483834657	3.63E-23	1.74E-21
SLC35F1	0.053702716	0.302240128	0.374737858
SLC35F2	0.431936639	2.70E-18	6.50E-17
SLC35F3	0.291811381	1.02E-08	5.64E-08
SLC35F4	-0.020888339	0.688402534	0.745710146
SLC35F5	-0.131769787	0.011066633	0.020195059
SLC36A1	0.472252527	5.25E-22	2.14E-20
SLC36A2	0.127555206	0.013946171	0.024820049
SLC36A3	0.150723493	0.003614468	0.007348447
SLC36A4	0.211445211	4.03E-05	0.000119894
SLC37A1	0.496276439	1.83E-24	1.08E-22
SLC37A2	0.247436121	1.40E-06	5.36E-06
SLC37A3	0.337060311	2.62E-11	2.16E-10
SLC37A4	-0.253187194	7.77E-07	3.11E-06
SLC38A10	-0.255979181	5.81E-07	2.37E-06
SLC38A11	-0.259519167	4.00E-07	1.68E-06
SLC38A1	0.399174408	1.27E-15	2.05E-14
SLC38A2	-0.278471251	4.93E-08	2.42E-07
SLC38A3	-0.202615073	8.48E-05	0.000237405
SLC38A4	-0.278172201	5.10E-08	2.50E-07
SLC38A5	0.203088616	8.16E-05	0.000228866
SLC38A6	0.127530656	0.013964714	0.02484639
SLC38A7	-0.150868888	0.003581764	0.00728493
SLC38A8	0.128748542	0.013070556	0.023443919
SLC38A9	0.07380935	0.155955317	0.211356587
SLC39A10	0.363649502	4.84E-13	5.21E-12
SLC39A11	-0.11177255	0.031367827	0.051437143
SLC39A12	0.011231929	0.829284762	0.864476533
SLC39A13	0.299699509	3.88E-09	2.29E-08
SLC39A14	-0.268454345	1.52E-07	6.88E-07
SLC39A1	0.393962639	3.18E-15	4.83E-14
SLC39A2	0.178586843	0.000548172	0.001317809
SLC39A3	0.049461633	0.342079193	0.416355186
SLC39A4	0.288572238	1.51E-08	8.10E-08
SLC39A5	-0.069239467	0.18327344	0.242844927
SLC39A6	0.194551647	0.000162741	0.000432053

SLC39A7	0.102177866	0.049230125	0.07700711
SLC39A8	-0.335930993	3.08E-11	2.51E-10
SLC39A9	-0.277336114	5.61E-08	2.72E-07
SLC3A1	0.207058744	5.86E-05	0.000168903
SLC3A2	0.055269352	0.288334534	0.359799598
SLC40A1	-0.229554815	7.95E-06	2.68E-05
SLC41A1	0.35384264	2.20E-12	2.15E-11
SLC41A2	-0.187072121	0.000291048	0.000735913
SLC41A3	0.200481815	0.000101046	0.000278852
SLC43A1	-0.037323671	0.473541646	0.54837357
SLC43A2	0.308990631	1.19E-09	7.62E-09
SLC43A3	0.056177146	0.28047659	0.351181863
SLC44A1	0.155096637	0.002740517	0.005723512
SLC44A2	0.212438229	3.70E-05	0.000110807
SLC44A3	0.245851976	1.64E-06	6.20E-06
SLC44A4	0.1529063	0.003150832	0.006494658
SLC44A5	0.041459505	0.425907605	0.502304719
SLC45A1	-0.079401805	0.126851287	0.176796651
SLC45A2	-0.248911405	1.20E-06	4.67E-06
SLC45A3	0.109788351	0.034521157	0.056026925
SLC45A4	0.399774473	1.14E-15	1.86E-14
SLC46A1	-0.099469529	0.055594244	0.085736843
SLC46A2	0.180236456	0.000485748	0.001178118
SLC46A3	-0.326660921	1.13E-10	8.44E-10
SLC47A1	-0.469311094	1.02E-21	3.98E-20
SLC47A2	-0.11042596	0.033479672	0.054527635
SLC48A1	0.082510353	0.112602124	0.159401657
SLC4A10	0.060028543	0.24875854	0.31680417
SLC4A11	0.484952494	2.79E-23	1.38E-21
SLC4A1AP	0.034375934	0.509197348	0.582098443
SLC4A1	-0.061244888	0.239281707	0.30647873
SLC4A2	0.184961901	0.000341567	0.000852505
SLC4A3	0.398081029	1.54E-15	2.45E-14
SLC4A4	-0.295462574	6.56E-09	3.74E-08
SLC4A5	0.389890561	6.43E-15	9.32E-14
SLC4A7	0.325789805	1.27E-10	9.42E-10
SLC4A8	0.258354722	4.53E-07	1.89E-06
SLC4A9	0.061546159	0.236974151	0.303933499
SLC5A10	0.239180438	3.17E-06	1.15E-05
SLC5A11	0.142618758	0.005926116	0.011476629
SLC5A12	0.099694209	0.055041531	0.084956798
SLC5A1	0.219083615	2.07E-05	6.44E-05
SLC5A2	0.141091416	0.006487411	0.012475115
SLC5A3	0.059577328	0.252339654	0.320852093
SLC5A4	-0.094625523	0.068674866	0.103318282
SLC5A5	0.323219843	1.81E-10	1.31E-09
SLC5A6	-0.353382398	2.36E-12	2.30E-11
SLC5A7	0.015576765	0.764913062	0.810606338
SLC5A8	0.04611804	0.37574148	0.451833268
SLC5A9	0.053013381	0.30849723	0.381187237
SLC6A10P	0.218586478	2.16E-05	6.71E-05
SLC6A11	0.167544966	0.001198739	0.002696316
SLC6A12	-0.507927572	9.99E-26	7.29E-24
SLC6A13	-0.514388234	1.90E-26	1.57E-24
SLC6A14	0.384846481	1.52E-14	2.07E-13
SLC6A15	0.237542663	3.72E-06	1.33E-05
SLC6A16	-0.283088108	2.89E-08	1.48E-07
SLC6A17	0.299281371	4.09E-09	2.40E-08

SLC6A18	0.031311426	0.547698172	0.618902041
SLC6A19	0.337747928	2.38E-11	1.97E-10
SLC6A1	-0.325108313	1.40E-10	1.03E-09
SLC6A20	0.08300106	0.110472379	0.156765491
SLC6A2	-0.180695245	0.000469599	0.001142289
SLC6A3	0.292906382	8.96E-09	5.00E-08
SLC6A4	0.037593178	0.470351345	0.545135813
SLC6A5	0.039348196	0.449868112	0.525646482
SLC6A6	0.397205789	1.80E-15	2.83E-14
SLC6A7	-0.027892388	0.59227886	0.659846742
SLC6A8	0.281997251	3.28E-08	1.66E-07
SLC6A9	0.226225243	1.08E-05	3.56E-05
SLC7A10	0.357973381	1.17E-12	1.19E-11
SLC7A11	0.108157477	0.037311218	0.060037678
SLC7A13	0.0471442	0.365200165	0.44086031
SLC7A14	-0.105696663	0.041881654	0.066619553
SLC7A1	0.442703298	3.08E-19	8.51E-18
SLC7A2	-0.109199272	0.035507745	0.057445615
SLC7A3	0.055178518	0.289128857	0.360700425
SLC7A4	0.08965497	0.084613344	0.12402678
SLC7A5P1	0.095358219	0.066548097	0.100437211
SLC7A5P2	0.176114824	0.000655766	0.001555258
SLC7A5	0.175763222	0.000672568	0.001590941
SLC7A6OS	0.052757717	0.310839438	0.38367297
SLC7A6	0.11500119	0.026762946	0.044665444
SLC7A7	0.424723492	1.11E-17	2.45E-16
SLC7A8	0.228860737	8.49E-06	2.84E-05
SLC7A9	-0.161479249	0.001806567	0.003920525
SLC8A1	0.11509483	0.026638565	0.044487676
SLC8A2	0.288889546	1.45E-08	7.84E-08
SLC8A3	-0.255405277	6.17E-07	2.51E-06
SLC9A10	0.140478649	0.006725674	0.012883545
SLC9A11	0.001276044	0.980457435	0.984857209
SLC9A1	0.312625514	7.45E-10	4.90E-09
SLC9A2	0.295150399	6.81E-09	3.88E-08
SLC9A3R1	0.037916686	0.466537483	0.541440041
SLC9A3R2	-0.394227011	3.03E-15	4.64E-14
SLC9A3	0.16937288	0.001056485	0.002399646
SLC9A4	0.174845831	0.000718304	0.001688302
SLC9A5	0.248318455	1.28E-06	4.94E-06
SLC9A6	0.03794383	0.466218254	0.541164136
SLC9A7	0.127159485	0.01424771	0.025300194
SLC9A8	0.037462323	0.471898865	0.546706745
SLC9A9	0.203894316	7.63E-05	0.00021531
SLCO1A2	-0.195805027	0.000147318	0.000394047
SLCO1B1	-0.387255818	1.01E-14	1.41E-13
SLCO1B3	-0.216123276	2.69E-05	8.20E-05
SLCO1C1	0.020286891	0.696925091	0.753181169
SLCO2A1	-0.189583067	0.000240033	0.000616388
SLCO2B1	-0.284501683	2.45E-08	1.27E-07
SLCO3A1	0.32694791	1.09E-10	8.14E-10
SLCO4A1	0.305706965	1.82E-09	1.13E-08
SLCO4C1	0.357281815	1.30E-12	1.31E-11
SLCO5A1	0.319355145	3.05E-10	2.13E-09
SLCO6A1	0.128914694	0.012952563	0.023246934
SLED1	-0.045875709	0.378257893	0.454393015
SLFN11	0.188088848	0.00026928	0.000685484
SLFN12L	0.069787011	0.179825482	0.239023115

SLFN12	0.13078301	0.011689008	0.021221946
SLFN13	0.340729871	1.54E-11	1.32E-10
SLFN14	0.088847357	0.087464443	0.12770937
SLFN5	-0.118116645	0.02288311	0.038834425
SLFNL1	0.039013448	0.453735725	0.529327347
SLIT1	0.185060804	0.000339027	0.000846591
SLIT2	0.146253908	0.004761569	0.009430359
SLIT3	0.014291592	0.783807525	0.82671609
SLITRK1	0.150448344	0.0036771	0.007459828
SLITRK2	0.232357011	6.11E-06	2.10E-05
SLITRK3	-0.046598723	0.370780528	0.44661294
SLITRK4	0.22071417	1.79E-05	5.64E-05
SLITRK5	0.202986514	8.23E-05	0.000230632
SLITRK6	0.084861842	0.10268291	0.147137232
SLK	-0.034708741	0.505102796	0.578380153
SLMAP	0.075980358	0.144109141	0.197274947
SLMO1	0.349410919	4.30E-12	4.01E-11
SLMO2	0.418857571	3.42E-17	7.07E-16
SLN	0.214593337	3.07E-05	9.30E-05
SLPI	0.161040174	0.00186001	0.00402773
SLTM	-0.0385311	0.459341388	0.534364913
SLU7	-0.030445309	0.558833712	0.629307852
SLURP1	-0.012829298	0.805460846	0.844546893
SMAD1	-0.212656856	3.63E-05	0.000108848
SMAD2	0.203381552	7.96E-05	0.000223793
SMAD3	-0.06363431	0.221410197	0.286703538
SMAD4	-0.119845064	0.020948196	0.035828465
SMAD5OS	0.229697955	7.85E-06	2.65E-05
SMAD5	0.361897448	6.37E-13	6.72E-12
SMAD6	-0.135323165	0.009061443	0.016885424
SMAD7	0.069511355	0.181555277	0.240936865
SMAD9	0.029240217	0.574507253	0.643758821
SMAGP	0.303455434	2.42E-09	1.47E-08
SMAP1	0.418525922	3.64E-17	7.49E-16
SMAP2	-0.161559442	0.001796959	0.00390095
SMARCA1	0.090250687	0.082558666	0.121391523
SMARCA2	-0.342248873	1.24E-11	1.07E-10
SMARCA4	0.459336664	9.19E-21	3.11E-19
SMARCA5	0.149538374	0.003891299	0.007857536
SMARCAD1	-0.084152416	0.105599699	0.150763739
SMARCAL1	0.121157427	0.01957544	0.033668472
SMARCB1	0.287796663	1.66E-08	8.82E-08
SMARCC1	0.307605657	1.43E-09	9.01E-09
SMARCC2	0.231732236	6.48E-06	2.22E-05
SMARCD1	0.535337648	6.73E-29	8.03E-27
SMARCD2	-0.210079926	4.54E-05	0.000133615
SMARCD3	0.356706371	1.42E-12	1.42E-11
SMARCE1	0.383911568	1.78E-14	2.39E-13
SMC1A	0.137295371	0.008094003	0.01523946
SMC1B	0.260570711	3.58E-07	1.52E-06
SMC2	0.245269109	1.74E-06	6.55E-06
SMC3	0.166962372	0.001247655	0.002795612
SMC4	0.474763618	2.97E-22	1.26E-20
SMC5	0.1311538	0.011451608	0.020815596
SMC6	0.017075017	0.743061278	0.792126861
SMCHD1	0.229445477	8.04E-06	2.70E-05
SMCP	0.05743141	0.269859966	0.339768075
SMCR5	0.133011338	0.010325332	0.018986641

SMCR7L	-0.030591242	0.556949822	0.627505619
SMCR7	0.002118735	0.96755734	0.974428422
SMCR8	0.127344538	0.014105994	0.025075365
SMEK1	-0.024224459	0.641867179	0.70550499
SMEK2	-0.075775694	0.145195681	0.198571316
SMEK3P	-0.014595449	0.779328311	0.822601389
SMG1	-0.013811609	0.790897528	0.832791631
SMG5	0.304544917	2.11E-09	1.30E-08
SMG6	-0.086288062	0.097012384	0.139954646
SMG7	0.096130564	0.064365036	0.097534056
SMN1	0.129445121	0.012582139	0.022667325
SMN2	0.34062146	1.57E-11	1.34E-10
SMNDC1	0.219159373	2.05E-05	6.40E-05
SMOC1	-0.45304943	3.55E-20	1.10E-18
SMOC2	0.082110501	0.114361272	0.161559018
SMOX	0.402536254	6.96E-16	1.18E-14
SMO	-0.262321286	2.97E-07	1.28E-06
SMPD1	-0.247815378	1.35E-06	5.18E-06
SMPD2	0.31781523	3.75E-10	2.58E-09
SMPD3	0.084610749	0.103707889	0.148371171
SMPD4	0.369467576	1.92E-13	2.21E-12
SMPDL3A	-0.174002271	0.000762881	0.001784236
SMPDL3B	0.435685255	1.28E-18	3.27E-17
SMPX	-0.113470445	0.028868163	0.047790555
SMR3A	-0.2963576	5.88E-09	3.37E-08
SMR3B	-0.272551979	9.64E-08	4.49E-07
SMS	0.478911023	1.14E-22	5.23E-21
SMTNL1	0.090600941	0.08136954	0.11981994
SMTNL2	0.209262812	4.86E-05	0.000142222
SMTN	0.174955022	0.000712713	0.00167714
SMU1	0.080748814	0.120513984	0.169111755
SMUG1	0.139673089	0.007050808	0.013448176
SMURF1	0.026386429	0.612427307	0.678273116
SMURF2	0.168948558	0.001088046	0.002466726
SMYD1	-0.086755947	0.095207415	0.137603172
SMYD2	-0.10902461	0.035804834	0.05786986
SMYD3	0.2821478	3.22E-08	1.64E-07
SMYD4	0.059353255	0.254131273	0.322882859
SMYD5	0.394089819	3.11E-15	4.74E-14
SNAI1	0.164492531	0.001476099	0.003262352
SNAI2	-0.048969781	0.346906755	0.421304339
SNAI3	0.210819438	4.26E-05	0.000126001
SNAP23	0.034948323	0.502165931	0.575612755
SNAP25	0.311581959	8.54E-10	5.58E-09
SNAP29	-0.05349491	0.304117474	0.37672465
SNAP47	-0.010253517	0.843956347	0.876514354
SNAP91	0.126352562	0.014880384	0.026287754
SNAPC1	0.059317535	0.254417689	0.323184926
SNAPC2	0.138908088	0.007372512	0.014006788
SNAPC3	0.080096525	0.123551298	0.172813941
SNAPC4	0.020018592	0.700739311	0.756687547
SNAPC5	0.044395098	0.39385628	0.470182455
SNAPIN	0.208634211	5.13E-05	0.000149298
SNAR-B2	0.083222423	0.109522061	0.155560994
SNAR-C4	0.090974559	0.080116386	0.118280617
SNAR-G1	0.084894015	0.102552158	0.146981589
SNCAIP	0.018988715	0.715449481	0.768904599
SNCA	0.191097663	0.000213437	0.000553946

SNCB	0.298908972	4.29E-09	2.51E-08
SNCG	0.172642117	0.000840177	0.001948092
SND1	-0.058265577	0.262953162	0.332461353
SNED1	-0.22917331	8.24E-06	2.77E-05
SNF8	0.164890528	0.001436865	0.003183765
SNHG10	0.11738335	0.023749617	0.040151042
SNHG11	0.083296184	0.109206833	0.1552298
SNHG12	0.393969548	3.17E-15	4.83E-14
SNHG1	0.472196625	5.32E-22	2.16E-20
SNHG3-RC(	0.124379451	0.016532284	0.028908643
SNHG3	0.318984403	3.21E-10	2.23E-09
SNHG4	0.355083278	1.82E-12	1.80E-11
SNHG5	0.001748497	0.973224187	0.978627713
SNHG6	0.294513137	7.37E-09	4.17E-08
SNHG7	0.268601799	1.50E-07	6.77E-07
SNHG8	-0.113945354	0.028200333	0.046789958
SNHG9	-0.014007716	0.787998642	0.830189852
SNIP1	-0.219969186	1.91E-05	5.99E-05
SNN	0.263186153	2.70E-07	1.17E-06
SNORA10	0.14272748	0.005887874	0.011410334
SNORA11B	0.108655522	0.036439579	0.058781319
SNORA11	0.012830648	0.805440789	0.844546893
SNORA12	0.1057464	0.041784806	0.066493881
SNORA13	0.064659196	0.214044052	0.278418503
SNORA14A	-0.008424627	0.871522371	0.898911141
SNORA14B	0.031500739	0.545278843	0.616520478
SNORA15	0.07771047	0.135170803	0.186687865
SNORA16A	0.071753542	0.167837629	0.225298919
SNORA16B	-0.094157378	0.070062632	0.1051045
SNORA18	-0.032620277	0.531080561	0.603031458
SNORA1	0.087629709	0.091908486	0.133441477
SNORA20	0.047649318	0.360079538	0.435576207
SNORA21	0.14601848	0.004830225	0.009555883
SNORA22	0.009778973	0.851092074	0.882545091
SNORA23	0.040583601	0.435756688	0.511950507
SNORA24	0.025197301	0.628546736	0.693350392
SNORA25	-0.00546532	0.916443226	0.934646357
SNORA26	0.08269432	0.111799935	0.158434934
SNORA27	0.057274456	0.271173257	0.341098222
SNORA28	0.048730287	0.349272957	0.423919572
SNORA29	0.076068009	0.143645721	0.196746992
SNORA2A	0.040994001	0.431125764	0.507377568
SNORA2B	0.022388717	0.667315077	0.727760646
SNORA31	0.026894965	0.60558979	0.671820904
SNORA32	0.123516634	0.017303659	0.030130539
SNORA34	0.138200135	0.007681842	0.014540395
SNORA36A	0.05036925	0.333283826	0.407342355
SNORA37	0.053309691	0.305797246	0.378367352
SNORA38B	-0.005483144	0.916171718	0.934465132
SNORA38	0.05748063	0.269449024	0.339357912
SNORA39	0.028225465	0.587863802	0.655520582
SNORA3	0.039994276	0.442456314	0.518110544
SNORA40	0.019489519	0.708282744	0.762642354
SNORA41	0.090883642	0.080419885	0.118631985
SNORA42	0.048260779	0.353941251	0.428801849
SNORA44	0.040230601	0.439762675	0.515441079
SNORA45	-0.02538267	0.626022042	0.690909707
SNORA46	0.174305645	0.000746563	0.001749357

SNORA47	-0.003004945	0.954000221	0.964163576
SNORA48	0.046603772	0.370728635	0.446587004
SNORA49	0.0874328	0.092643825	0.134346359
SNORA4	-0.003419614	0.947660988	0.959509159
SNORA50	0.080155177	0.12327578	0.172489119
SNORA51	0.003422424	0.947618038	0.959509159
SNORA52	-0.003903439	0.940268717	0.954060985
SNORA53	0.003567892	0.94539497	0.957798952
SNORA54	0.080030787	0.123860673	0.173222347
SNORA55	0.066208102	0.203248372	0.266178941
SNORA56	-0.015021365	0.773062118	0.817286325
SNORA57	0.147127895	0.004514323	0.008980862
SNORA58	0.112862753	0.029742455	0.049062615
SNORA59B	0.057067873	0.272908446	0.343085888
SNORA5A	0.042985617	0.409059894	0.485327735
SNORA5B	0.097345855	0.061049438	0.093012153
SNORA5C	-0.039342641	0.449932142	0.525690465
SNORA61	0.00939256	0.856911589	0.887563308
SNORA62	0.085010936	0.102078105	0.146375874
SNORA63	0.101758598	0.050173549	0.078281596
SNORA64	0.07109846	0.171762709	0.22982448
SNORA65	0.037786353	0.468071929	0.542936184
SNORA66	-0.003050386	0.953305394	0.963734912
SNORA67	0.134548975	0.009468473	0.017568308
SNORA68	0.070940705	0.172718078	0.230932185
SNORA6	0.057980442	0.26530024	0.334894905
SNORA70	0.02274125	0.662397286	0.723757952
SNORA71A	0.076952124	0.139034703	0.191367235
SNORA71B	0.018222764	0.726458897	0.778050328
SNORA71C	0.100140196	0.053957915	0.08349123
SNORA71D	0.129268646	0.01270433	0.022846693
SNORA72	0.052017304	0.317688307	0.390943632
SNORA74A	0.129935884	0.012247765	0.022123491
SNORA74B	0.109212484	0.035485357	0.057423385
SNORA75	0.043886643	0.399301106	0.475514175
SNORA76	0.103251932	0.04688073	0.073743222
SNORA77	-0.025959142	0.618198533	0.683221523
SNORA79	0.097965345	0.059414314	0.090833547
SNORA7B	-0.079326997	0.127210672	0.177161202
SNORA80	-0.041304911	0.427636468	0.503985667
SNORA81	0.008609268	0.868731719	0.89658982
SNORA84	0.041479844	0.425680456	0.502096272
SNORA8	0.216644276	2.57E-05	7.86E-05
SNORA9	0.037128513	0.475859208	0.550494579
SNORD10	0.040213826	0.439953565	0.515585319
SNORD115-	0.104784217	0.043692223	0.069153608
SNORD115-	0.021997202	0.672793385	0.732425673
SNORD115-	-0.02971127	0.568356071	0.638083384
SNORD116-	0.191288763	0.000210284	0.000546903
SNORD116-	0.183733437	0.000374624	0.000927343
SNORD116-	0.138810501	0.007414479	0.014069078
SNORD15A	0.038406528	0.460795372	0.535649749
SNORD15B	0.095215157	0.066959058	0.100996241
SNORD17	0.119883159	0.0209072	0.035770666
SNORD1C	0.125609111	0.015484977	0.027259142
SNORD22	0.050253895	0.334393551	0.408297887
SNORD89	-0.030606053	0.55675879	0.627325865
SNORD94	-0.007339971	0.887946849	0.911745033

SNORD97	-0.017457482	0.737515175	0.787183354
SNPH	0.416507804	5.33E-17	1.07E-15
SNRK	-0.303986735	2.27E-09	1.38E-08
SNRNP200	0.084651648	0.103540384	0.148195383
SNRNP25	-0.022627412	0.66398374	0.725053939
SNRNP27	0.153560506	0.003022792	0.006253356
SNRNP35	0.187818517	0.000274915	0.000698934
SNRNP40	0.231999111	6.32E-06	2.17E-05
SNRNP48	0.034545766	0.507105727	0.580140134
SNRNP70	0.184955523	0.000341731	0.000852808
SNRPA1	0.450651609	5.89E-20	1.78E-18
SNRPA	0.465100152	2.60E-21	9.67E-20
SNRPB2	0.296505191	5.77E-09	3.31E-08
SNRPB	0.4781954	1.35E-22	6.03E-21
SNRPC	0.194088422	0.000168815	0.00044669
SNRPD1	0.500861353	5.91E-25	3.82E-23
SNRPD2	0.327538537	1.00E-10	7.54E-10
SNRPD3	0.152869252	0.003158228	0.006508557
SNRPE	0.314046289	6.18E-10	4.11E-09
SNRPF	0.421933822	1.90E-17	4.07E-16
SNRPG	0.401560946	8.29E-16	1.38E-14
SNRPN	0.250959391	9.78E-07	3.86E-06
SNTA1	-0.069052292	0.184463228	0.244237585
SNTB1	-0.114110185	0.027971664	0.046442519
SNTB2	0.038906147	0.454979407	0.530342565
SNTG1	-0.362064408	6.20E-13	6.56E-12
SNTG2	0.066049803	0.204333229	0.267423899
SNTN	0.131493243	0.011238026	0.020468454
SNUPN	0.047742986	0.359134935	0.434591883
SNURF	0.085753681	0.0991071	0.142567091
SNW1	0.164302692	0.001495157	0.003299348
SNX10	-0.010539039	0.83966904	0.873130548
SNX11	0.048566294	0.350899094	0.425608022
SNX12	-0.039391061	0.449374205	0.525192592
SNX13	-0.091585007	0.078102529	0.11563307
SNX14	0.144800512	0.005199859	0.010196022
SNX15	0.094762892	0.068271949	0.102828591
SNX16	0.18182145	0.000432048	0.001057013
SNX17	-0.021030496	0.686393855	0.744060201
SNX18	0.057325574	0.270745052	0.340667146
SNX19	-0.200704569	9.92E-05	0.00027421
SNX1	-0.039171689	0.451905118	0.527624319
SNX20	0.159569729	0.002049696	0.004402593
SNX21	-0.048998923	0.346619521	0.421058177
SNX22	0.294106305	7.74E-09	4.37E-08
SNX24	0.327649298	9.86E-11	7.43E-10
SNX25	-0.101690353	0.050328531	0.078503366
SNX27	0.221150987	1.72E-05	5.43E-05
SNX29	0.095090567	0.06731865	0.1015002
SNX2	0.192910085	0.000185236	0.000486521
SNX30	0.052133437	0.316607598	0.389806351
SNX31	-0.141992908	0.006150619	0.011877733
SNX32	0.22232688	1.55E-05	4.93E-05
SNX33	-0.246524567	1.53E-06	5.83E-06
SNX3	0.155101365	0.002739687	0.005722378
SNX4	-0.194224107	0.000167014	0.000442395
SNX5	0.247554545	1.38E-06	5.30E-06
SNX6	0.147884716	0.004309647	0.008619118

SNX7	0.231613992	6.56E-06	2.24E-05
SNX8	0.240113725	2.90E-06	1.05E-05
SNX9	-0.005615494	0.914155998	0.932855845
SOAT1	0.398846681	1.34E-15	2.15E-14
SOAT2	0.210591666	4.34E-05	0.000128236
SOBP	0.098506839	0.058014858	0.088967131
SOCS1	0.353309334	2.39E-12	2.32E-11
SOCS2	-0.228159388	9.06E-06	3.02E-05
SOCS3	0.160681337	0.001904757	0.004116126
SOCS4	0.195123737	0.000155522	0.000414325
SOCS5	0.120337273	0.020423844	0.035006781
SOCS6	-0.275540836	6.88E-08	3.29E-07
SOCS7	0.237885688	3.60E-06	1.29E-05
SOD1	-0.2989287	4.28E-09	2.50E-08
SOD2	0.066396819	0.201960517	0.264718561
SOD3	0.226123607	1.09E-05	3.59E-05
SOHLH1	0.101540825	0.050669501	0.078961029
SOHLH2	0.124223431	0.0166695	0.029128127
SOLH	0.225906365	1.12E-05	3.65E-05
SON	0.002852514	0.956331259	0.965686621
SORBS1	-0.214816204	3.01E-05	9.13E-05
SORBS2	-0.459503015	8.87E-21	3.01E-19
SORBS3	-0.123707589	0.017130285	0.029849517
SORCS1	0.272123573	1.01E-07	4.69E-07
SORCS2	0.24125047	2.59E-06	9.51E-06
SORCS3	0.199372068	0.000110589	0.000303048
SORD	-0.501166497	5.48E-25	3.57E-23
SORL1	-0.501422556	5.14E-25	3.38E-23
SORT1	0.089069479	0.086672702	0.126701905
SOS1	-0.074292622	0.153256653	0.208223353
SOS2	-0.149134432	0.003989951	0.008032316
SOSTDC1	0.036612778	0.48201342	0.556242867
SOST	-0.001741698	0.973328267	0.978682973
SOX10	-0.029386263	0.572596779	0.641981988
SOX11	0.375218754	7.56E-14	9.27E-13
SOX12	0.247539285	1.38E-06	5.31E-06
SOX13	0.112035773	0.030968596	0.05083688
SOX14	-0.038778316	0.456463526	0.531668987
SOX15	0.074761372	0.150673016	0.20518886
SOX17	-0.0891664	0.086329044	0.126255118
SOX18	-0.045542346	0.381736437	0.457991767
SOX1	0.08253114	0.112511256	0.159295661
SOX21	0.192630965	0.000189339	0.000496055
SOX2OT	-0.259468802	4.02E-07	1.69E-06
SOX2	0.072490059	0.163505161	0.220210236
SOX30	0.077423913	0.136621029	0.188422578
SOX3	0.110945235	0.032651368	0.053326821
SOX4	0.443158344	2.80E-19	7.79E-18
SOX5	-0.253467986	7.55E-07	3.03E-06
SOX6	0.072844199	0.16145211	0.217798546
SOX7	-0.167915628	0.001168542	0.002635474
SOX8	0.157904851	0.00228569	0.004859195
SOX9	0.302970535	2.58E-09	1.56E-08
SP100	-0.092673349	0.074614045	0.110979618
SP110	0.136691789	0.008379814	0.015712292
SP140L	0.212100723	3.81E-05	0.000113757
SP140	0.25870687	4.36E-07	1.82E-06
SP1	0.150692616	0.003621448	0.007361135

SP2	-0.02457636	0.637035286	0.701044963
SP3	0.127488944	0.013996268	0.024898085
SP4	0.105886692	0.041512645	0.066113592
SP5	-0.197425041	0.000129409	0.000349665
SP6	0.117550537	0.023549603	0.039853482
SP7	0.03157338	0.544351925	0.615749435
SP8	0.303313397	2.47E-09	1.50E-08
SP9	0.087399556	0.092768436	0.134487904
SPA17	0.203410903	7.94E-05	0.000223313
SPACA1	-0.217769684	2.32E-05	7.17E-05
SPACA3	0.043714806	0.401151391	0.477289656
SPACA4	0.204116351	7.49E-05	0.00021186
SPACA5	-0.073322406	0.158710651	0.214638082
SPAG11A	0.01432777	0.783273839	0.826328184
SPAG11B	-0.004108622	0.937135283	0.951608599
SPAG16	0.05750383	0.269255471	0.339149321
SPAG17	0.21615778	2.68E-05	8.17E-05
SPAG1	0.387849633	9.13E-15	1.28E-13
SPAG4	-0.016349578	0.753617185	0.800894141
SPAG5	0.480131984	8.62E-23	4.00E-21
SPAG6	0.14263556	0.005920191	0.01146627
SPAG7	-0.073185302	0.159493028	0.215476746
SPAG8	0.26335217	2.65E-07	1.15E-06
SPAG9	-0.074052328	0.154594033	0.209748913
SPAM1	0.013539597	0.79492323	0.835963556
SPANXA2	0.09556157	0.065967511	0.099659115
SPANXB2	0.109107882	0.035662931	0.057677943
SPANXC	0.119873412	0.020917683	0.035785494
SPANXE	0.122223355	0.018518726	0.032036303
SPANXN1	0.031609024	0.543897384	0.615312882
SPANXN2	0.085028015	0.102009004	0.146308379
SPANXN3	0.256171609	5.70E-07	2.33E-06
SPANXN4	-0.021113261	0.685225394	0.743036159
SPANXN5	0.105919828	0.041448582	0.066037959
SPARCL1	-0.385001137	1.48E-14	2.01E-13
SPARC	0.159218254	0.002097579	0.004497199
SPAST	0.075365854	0.147390241	0.201268653
SPATA12	0.353301969	2.39E-12	2.32E-11
SPATA13	-0.037415082	0.472458233	0.547227495
SPATA16	0.134174889	0.009670907	0.017915095
SPATA17	0.17202622	0.000877505	0.002026153
SPATA18	-0.217567253	2.37E-05	7.29E-05
SPATA19	0.069679607	0.180498009	0.239743449
SPATA1	-0.283248069	2.83E-08	1.45E-07
SPATA20	0.001128692	0.982713763	0.98667592
SPATA21	-0.035370679	0.497010547	0.570918764
SPATA22	-0.069453618	0.181919139	0.241291151
SPATA24	0.1466337	0.004652666	0.009233953
SPATA2L	-0.08391058	0.106608836	0.152030153
SPATA2	0.164963132	0.001429812	0.003169547
SPATA3	-0.0337987	0.516339959	0.588945646
SPATA4	0.020801599	0.689629253	0.746592434
SPATA5L1	0.07215896	0.165442308	0.222423374
SPATA5	-0.137222647	0.008127973	0.015291857
SPATA6	0.186312171	0.000308379	0.000776481
SPATA7	-0.105375845	0.042510908	0.067498391
SPATA8	0.126186313	0.015013753	0.026509261
SPATA9	-0.033218513	0.523570882	0.595624664

SPATC1	0.065194658	0.210266309	0.274036726
SPATS1	0.064540396	0.214888765	0.279439555
SPATS2L	0.365951652	3.36E-13	3.73E-12
SPATS2	0.509967526	5.93E-26	4.60E-24
SPC24	0.330212136	6.90E-11	5.36E-10
SPC25	0.575628744	4.03E-34	1.57E-31
SPCS1	-0.098141319	0.058956493	0.090216703
SPCS2	0.167727777	0.001183757	0.002665326
SPCS3	-0.13127323	0.011376055	0.020696616
SPDEF	0.273809999	8.37E-08	3.93E-07
SPDYA	0.268141809	1.57E-07	7.09E-07
SPDYC	-0.511514157	3.99E-26	3.20E-24
SPDYE1	0.019021825	0.714974883	0.768560385
SPDYE2	0.077928242	0.134076635	0.185439789
SPDYE3	-0.189101165	0.000249125	0.000638007
SPDYE4	0.035661327	0.493479212	0.567484003
SPDYE5	0.056422724	0.278375979	0.348973517
SPDYE6	0.050705841	0.330059331	0.404070669
SPDYE7P	-0.007677503	0.882830268	0.907567191
SPDYE8P	-0.24377626	2.02E-06	7.52E-06
SPEF1	0.030191236	0.562120939	0.632437643
SPEF2	0.207523273	5.63E-05	0.00016289
SPEG	0.220664515	1.80E-05	5.66E-05
SPEM1	0.110710177	0.033024123	0.053869367
SPEN	-0.050089311	0.335980972	0.409984866
SPERT	0.22732207	9.79E-06	3.24E-05
SPESP1	0.051379219	0.323669055	0.397615768
SPG11	-0.190298891	0.000227099	0.000586045
SPG20	0.046142762	0.375485346	0.451574073
SPG21	-0.061275771	0.239044443	0.306214209
SPG7	-0.364909739	3.97E-13	4.34E-12
SPHAR	-0.228644096	8.66E-06	2.90E-05
SPHK1	0.483144413	4.27E-23	2.03E-21
SPHK2	-0.056592625	0.27692895	0.347394908
SPHKAP	0.093927577	0.070752193	0.105963403
SPI1	0.342735434	1.15E-11	1.01E-10
SPIB	0.377601297	5.11E-14	6.42E-13
SPIC	-0.049823699	0.338552978	0.412643158
SPIN1	0.333634169	4.27E-11	3.41E-10
SPIN2A	0.12873069	0.013083289	0.023456199
SPIN2B	0.013882814	0.789844628	0.831894511
SPIN3	0.098773796	0.057335031	0.088066962
SPIN4	0.206955366	5.91E-05	0.000170231
SPINK13	0.308071171	1.35E-09	8.52E-09
SPINK14	-0.006841315	0.895514346	0.917623537
SPINK1	0.291550718	1.06E-08	5.81E-08
SPINK2	0.190477742	0.000223971	0.000578872
SPINK4	0.227235242	9.87E-06	3.26E-05
SPINK5	-0.064432098	0.21566089	0.280278964
SPINK6	-0.06212643	0.232573851	0.29905998
SPINK7	0.01331665	0.79822683	0.838639115
SPINK8	-0.108163107	0.037301267	0.060035211
SPINK9	-0.014001288	0.78809361	0.830225842
SPINLW1	0.05509665	0.289846033	0.361414074
SPINT1	0.497354137	1.41E-24	8.44E-23
SPINT2	0.396482844	2.04E-15	3.19E-14
SPINT3	-0.182455646	0.000412151	0.001012012
SPIRE1	0.283084247	2.89E-08	1.48E-07

SPIRE2	0.08721839	0.093449861	0.135347738
SPNS1	0.276720858	6.02E-08	2.91E-07
SPNS2	-0.178526202	0.000550603	0.001323333
SPNS3	0.242867931	2.21E-06	8.18E-06
SPN	0.177810636	0.00058005	0.001389081
SPO11	0.036765249	0.480189528	0.5545059
SPOCD1	0.427770826	6.14E-18	1.41E-16
SPOCK1	0.170903257	0.000949531	0.002175817
SPOCK2	0.203520188	7.87E-05	0.000221491
SPOCK3	0.011809984	0.820644081	0.857398331
SPON1	0.167161091	0.001230767	0.002761188
SPON2	0.132903022	0.010388206	0.01908816
SPOPL	0.126542229	0.014729498	0.0260628
SPOP	-0.123273938	0.017526223	0.030463336
SPP1	0.314159422	6.09E-10	4.06E-09
SPP2	-0.315817346	4.89E-10	3.31E-09
SPPL2A	-0.314026333	6.20E-10	4.12E-09
SPPL2B	0.106507574	0.040326044	0.064425791
SPPL3	0.313337982	6.78E-10	4.48E-09
SPRED1	0.274541024	7.71E-08	3.65E-07
SPRED2	0.024804431	0.63391186	0.698186205
SPRED3	0.163093634	0.001621928	0.003546487
SPRN	0.053747745	0.301834352	0.374370811
SPRR1A	0.150102637	0.003757186	0.007608321
SPRR1B	0.092974669	0.073670951	0.109806426
SPRR2A	-0.003305847	0.949399889	0.960830307
SPRR2B	-0.029416115	0.572206639	0.641647003
SPRR2C	0.010761132	0.836337436	0.870371122
SPRR2D	0.035508626	0.495332837	0.569188486
SPRR2E	-0.023574762	0.650827734	0.713621559
SPRR2F	0.155628458	0.002648525	0.005557606
SPRR2G	0.031915128	0.540001642	0.611731645
SPRR3	0.150707486	0.003618085	0.00735505
SPRR4	-0.014697267	0.777829025	0.821454084
SPRY1	-0.026977328	0.604485579	0.670820059
SPRY2	-0.090619526	0.08130683	0.119736448
SPRY3	-0.111270485	0.032141511	0.052593281
SPRY4	0.284870797	2.34E-08	1.22E-07
SPRYD3	0.122980052	0.017799071	0.030895974
SPRYD4	-0.31265573	7.42E-10	4.89E-09
SPRYD5	-0.006487495	0.900889622	0.921993376
SPR	-0.115029037	0.026725904	0.044614837
SPSB1	0.038349436	0.461462592	0.536394058
SPSB2	0.161093296	0.001853468	0.00401531
SPSB3	-0.331494323	5.77E-11	4.51E-10
SPSB4	0.133759063	0.009900429	0.01829481
SPTA1	0.065987447	0.204761717	0.267861509
SPTAN1	0.148849095	0.004060994	0.008158023
SPTBN1	-0.173050415	0.000816246	0.001897241
SPTBN2	-0.171033081	0.000940934	0.002157606
SPTBN4	-0.107109148	0.039203793	0.062772702
SPTBN5	0.177053485	0.000612794	0.001460123
SPTB	0.321670596	2.23E-10	1.60E-09
SPTLC1	0.218237034	2.23E-05	6.90E-05
SPTLC2	0.062050917	0.233143191	0.299695357
SPTLC3	0.039754832	0.445195053	0.520858342
SPTY2D1	-0.015756564	0.762280456	0.808462055
SPZ1	0.051977823	0.318056254	0.391348076

SQLE	0.142700542	0.005897328	0.011426432
SQRDL	0.074629951	0.151394015	0.205972634
SQSTM1	0.165122666	0.001414426	0.003139632
SR140	0.285511819	2.17E-08	1.14E-07
SRA1	0.104178621	0.044929901	0.070971573
SRBD1	-0.175181998	0.00070122	0.001652047
SRCAP	0.170456497	0.000979673	0.002240248
SRCIN1	-0.024087096	0.643757406	0.707075479
SRCRB4D	0.092850221	0.074059274	0.110286205
SRC	0.412256068	1.18E-16	2.25E-15
SRD5A1	-0.328127342	9.23E-11	6.99E-10
SRD5A2	-0.457508087	1.37E-20	4.54E-19
SRD5A3	0.11729193	0.023859608	0.040302791
SREBF1	0.054076385	0.298883726	0.371080743
SREBF2	0.006918908	0.894336165	0.916652008
SRFBP1	0.106797455	0.039781921	0.063621693
SRF	0.139152311	0.007268413	0.013826331
SRGAP1	0.182454661	0.000412181	0.001012012
SRGAP2	0.0972058	0.061424207	0.093533062
SRGAP3	-0.155582964	0.002656283	0.005573298
SRGN	0.098627505	0.057706752	0.088552736
SRI	0.184135714	0.000363484	0.000902459
SRL	-0.249593279	1.12E-06	4.39E-06
SRMS	0.069408375	0.182204638	0.241605485
SRM	0.202982726	8.23E-05	0.000230672
SRP14	0.265489918	2.10E-07	9.30E-07
SRP19	0.370152954	1.72E-13	2.00E-12
SRP54	-0.106007839	0.041278829	0.065810347
SRP68	0.095580783	0.065912872	0.099591675
SRP72	0.20435491	7.35E-05	0.00020797
SRP9	0.085982213	0.09820692	0.141435572
SRPK1	0.262379055	2.95E-07	1.27E-06
SRPK2	0.105471811	0.042321852	0.067224998
SRPK3	0.279669195	4.29E-08	2.13E-07
SRPRB	0.119361329	0.021474865	0.036653724
SRPR	-0.305202404	1.94E-09	1.20E-08
SRPX2	0.298152	4.71E-09	2.74E-08
SRPX	0.101607064	0.050518217	0.078743754
SRRD	0.211839493	3.90E-05	0.00011612
SRRM1	-0.042373891	0.415765177	0.49176951
SRRM2	-0.008356619	0.872550643	0.899738815
SRRM3	0.219998974	1.91E-05	5.97E-05
SRRM4	0.116759237	0.024509298	0.041284707
SRRM5	0.274822182	7.47E-08	3.55E-07
SRRT	0.486067012	2.14E-23	1.08E-21
SRR	-0.24283746	2.22E-06	8.20E-06
SRXN1	0.131435128	0.011274341	0.020530843
SRY	0.062457349	0.230090439	0.296249062
SS18L1	-0.372089493	1.26E-13	1.49E-12
SS18L2	0.070683777	0.174282506	0.232711534
SS18	0.081840273	0.115562285	0.163012946
SSBP1	0.191250898	0.000210905	0.000548447
SSBP2	0.408250188	2.47E-16	4.46E-15
SSBP3	-0.210489137	4.38E-05	0.000129328
SSBP4	0.245800672	1.65E-06	6.23E-06
SSB	0.346947586	6.21E-12	5.64E-11
SSC5D	0.232152564	6.23E-06	2.14E-05
SSFA2	-0.222088861	1.58E-05	5.02E-05

SSH1	0.06593917	0.205093906	0.26827845
SSH2	0.361852681	6.41E-13	6.76E-12
SSH3	0.186778162	0.000297641	0.000750773
SSNA1	0.255507572	6.11E-07	2.49E-06
SSPN	0.250862073	9.87E-07	3.89E-06
SSPO	-0.073064988	0.160181977	0.216290183
SSR1	0.236149777	4.26E-06	1.51E-05
SSR2	0.326352719	1.18E-10	8.78E-10
SSR3	0.205518487	6.67E-05	0.000190267
SSR4	0.073497852	0.157713703	0.213420215
SSRP1	0.360606255	7.79E-13	8.11E-12
SSSCA1	0.077781739	0.134811968	0.186301611
SSTR1	-0.288162471	1.59E-08	8.47E-08
SSTR2	0.255200885	6.30E-07	2.56E-06
SSTR3	0.3685143	2.24E-13	2.56E-12
SSTR4	0.060277497	0.246797913	0.314790193
SSTR5	0.30563284	1.84E-09	1.14E-08
SST	0.054660289	0.293688727	0.365496428
SSU72	-0.080660126	0.120923497	0.16963861
SSX1	-0.006734449	0.897137369	0.918940742
SSX2IP	-0.030394779	0.559486731	0.629865204
SSX2	-0.017170258	0.741678945	0.791076557
SSX3	0.022180125	0.670231645	0.730635011
SSX4	0.127060815	0.014323782	0.025414891
SSX5	0.014145353	0.785965803	0.828698947
SSX6	0.051164681	0.325696223	0.399809003
SSX7	0.036495687	0.483416638	0.557553769
SSX8	-0.050415578	0.332838814	0.406948254
ST13	-0.288318257	1.56E-08	8.32E-08
ST14	0.160249186	0.001959952	0.00422349
ST18	0.293248611	8.60E-09	4.82E-08
ST20	0.075799524	0.14506885	0.198411481
ST3GAL1	-0.274662518	7.60E-08	3.60E-07
ST3GAL2	-0.142428323	0.005993637	0.011599498
ST3GAL3	-0.419248106	3.17E-17	6.60E-16
ST3GAL4	0.164361295	0.00148925	0.003287769
ST3GAL5	-0.017640656	0.734863781	0.785024083
ST3GAL6	-0.517933345	7.50E-27	6.47E-25
ST5	0.1409551	0.00653975	0.012568484
ST6GAL1	-0.284819802	2.36E-08	1.23E-07
ST6GAL2	0.149491073	0.003902736	0.007877437
ST6GALNA	0.136910501	0.008275226	0.015543943
ST6GALNA	0.103889098	0.045531934	0.071808676
ST6GALNA	-0.250466146	1.03E-06	4.04E-06
ST6GALNA	0.461064689	6.31E-21	2.21E-19
ST6GALNA	0.362798028	5.53E-13	5.90E-12
ST6GALNA	-0.388106955	8.74E-15	1.23E-13
ST7L	0.018832327	0.717692637	0.77094105
ST7OT1	-0.121536118	0.019194164	0.033073484
ST7OT2	0.070487401	0.175485319	0.234097932
ST7OT3	0.021688527	0.677124776	0.735867844
ST7OT4	-0.067891189	0.191971262	0.25299011
ST7	-0.319769548	2.89E-10	2.02E-09
ST8SIA1	0.247408423	1.40E-06	5.37E-06
ST8SIA2	0.143569407	0.005599156	0.010898524
ST8SIA3	0.006133259	0.906275755	0.92645862
ST8SIA4	0.221963125	1.60E-05	5.08E-05
ST8SIA5	0.091918836	0.077018715	0.114164199

ST8SIA6	-0.043819853	0.400019671	0.476170643
STAB1	0.132793639	0.010452043	0.019185995
STAB2	0.052024368	0.317622496	0.390910938
STAC2	0.268974227	1.44E-07	6.51E-07
STAC3	0.304721753	2.06E-09	1.27E-08
STAC	0.187687446	0.000277686	0.00070535
STAG1	0.004150051	0.936502715	0.951076108
STAG2	-0.057867132	0.266236927	0.335907034
STAG3L1	-0.006081024	0.907070358	0.926937967
STAG3L2	0.001910528	0.970743973	0.977017224
STAG3L3	-0.047128222	0.365362879	0.441003288
STAG3L4	0.281748154	3.38E-08	1.71E-07
STAG3	-0.166607193	0.001278371	0.002858649
STAM2	-0.146821941	0.004599526	0.009134858
STAMBPL1	0.23997236	2.94E-06	1.07E-05
STAMBP	0.222287773	1.55E-05	4.94E-05
STAM	0.195246557	0.000154012	0.000410741
STAP1	0.169033535	0.001081657	0.002453464
STAP2	-0.033639266	0.518321844	0.590803472
STARD10	-0.292136223	9.84E-09	5.44E-08
STARD13	-0.351784237	3.01E-12	2.87E-11
STARD3NL	0.310921902	9.30E-10	6.04E-09
STARD3	0.109011692	0.035826891	0.057896116
STARD4	0.068064716	0.190835179	0.251709377
STARD5	-0.453527244	3.20E-20	1.01E-18
STARD6	0.135934146	0.008751282	0.016350585
STARD7	-0.076316088	0.142340254	0.195287716
STARD8	0.016559197	0.750562202	0.798328894
STAR	0.171175784	0.000931566	0.002138342
STAT1	0.229768988	7.80E-06	2.63E-05
STAT2	-0.092850702	0.07405777	0.110286205
STAT3	-0.016075222	0.757621463	0.804334388
STAT4	0.273367069	8.80E-08	4.13E-07
STAT5A	0.022637166	0.663847752	0.72494518
STAT5B	-0.359710286	8.95E-13	9.22E-12
STAT6	-0.177145646	0.000608719	0.00145076
STAU1	-0.145354669	0.005028635	0.009898707
STAU2	0.082533138	0.112502525	0.15929462
STBD1	-0.42806715	5.79E-18	1.34E-16
STC1	-0.05316565	0.307107795	0.37970592
STC2	0.25478165	6.59E-07	2.67E-06
STEAP1	0.002954503	0.954771556	0.964796294
STEAP2	0.054178285	0.297972748	0.370112896
STEAP3	-0.245237605	1.75E-06	6.57E-06
STEAP4	-0.300338584	3.59E-09	2.13E-08
STH	0.026039846	0.61710668	0.682317642
STIL	0.529309679	3.55E-28	3.87E-26
STIM1	-0.117359401	0.023778389	0.04018569
STIM2	0.190713728	0.000219905	0.000569249
STIP1	0.567649212	4.98E-33	1.40E-30
STK10	0.125525784	0.015554064	0.027370418
STK11IP	0.411176304	1.44E-16	2.71E-15
STK11	-0.024393183	0.639548562	0.703422208
STK16	-0.28855745	1.51E-08	8.11E-08
STK17A	0.293311831	8.53E-09	4.78E-08
STK17B	0.388363148	8.36E-15	1.19E-13
STK19	0.210080149	4.54E-05	0.000133615
STK24	0.420008817	2.74E-17	5.74E-16

STK25	0.164398199	0.001485541	0.003280672
STK31	0.195662939	0.000148995	0.000398319
STK32A	0.18224291	0.000418728	0.001026443
STK32B	0.085015121	0.102061167	0.14636212
STK32C	0.341900019	1.30E-11	1.13E-10
STK33	0.207446617	5.67E-05	0.000163899
STK35	0.173977596	0.000764223	0.001786954
STK36	-0.045300897	0.384268053	0.460502175
STK38L	0.125006301	0.015990896	0.02806048
STK38	0.132235403	0.010783279	0.019732203
STK39	0.394732444	2.78E-15	4.26E-14
STK3	0.219153132	2.06E-05	6.40E-05
STK40	-0.116783776	0.024479038	0.04124071
STK4	0.268383087	1.53E-07	6.92E-07
STL	-0.263495514	2.61E-07	1.14E-06
STMN1	0.490877819	6.80E-24	3.65E-22
STMN2	0.308724778	1.24E-09	7.87E-09
STMN3	0.289393788	1.37E-08	7.42E-08
STMN4	0.040309366	0.438866998	0.51469408
STOML1	-0.053130289	0.30743009	0.380080815
STOML2	0.079199007	0.127827369	0.177895693
STOML3	0.106291031	0.040736596	0.065028403
STOM	0.030709552	0.55542479	0.626106075
STON1-GTF	-0.026274798	0.613932787	0.679411676
STON1	0.127608433	0.013906044	0.024761906
STON2	-0.107229126	0.038983158	0.06244906
STOX1	0.23861668	3.35E-06	1.21E-05
STOX2	0.011687934	0.822466696	0.858924608
STRA13	-0.124971176	0.016020816	0.028100608
STRA6	0.428129416	5.72E-18	1.32E-16
STRA8	0.198168061	0.000121898	0.000331121
STRADA	0.224661037	1.25E-05	4.06E-05
STRADB	-0.054086255	0.298795409	0.371034854
STRAP	0.190826482	0.000217986	0.00056465
STRBP	0.334896849	3.57E-11	2.88E-10
STRC	0.098721741	0.057467073	0.088235764
STRN3	-0.097584134	0.060416166	0.092216585
STRN4	0.401515075	8.36E-16	1.39E-14
STRN	0.013218242	0.799686191	0.839722732
STS	0.046162499	0.375280933	0.451361189
STT3A	0.2545938	6.72E-07	2.72E-06
STT3B	-0.084129122	0.105696572	0.150880418
STUB1	-0.144986352	0.005141862	0.010098203
STX10	0.257928221	4.73E-07	1.97E-06
STX11	0.210037917	4.55E-05	0.000134016
STX12	-0.092412443	0.075438559	0.112080466
STX16	0.25868453	4.37E-07	1.83E-06
STX17	-0.12548679	0.015586487	0.027420401
STX18	0.330460766	6.67E-11	5.19E-10
STX19	-0.063175528	0.224765561	0.290368945
STX1A	0.33865604	2.08E-11	1.74E-10
STX1B	-0.25967391	3.94E-07	1.65E-06
STX2	0.252024457	8.76E-07	3.48E-06
STX3	0.445349066	1.78E-19	5.06E-18
STX4	0.197351029	0.00013018	0.000351607
STX5	-0.143589075	0.005592566	0.010887824
STX6	0.48092219	7.18E-23	3.35E-21
STX7	0.39201322	4.46E-15	6.63E-14

STX8	-0.008352506	0.87261283	0.899756369
STXBP1	0.046497725	0.371819519	0.447670686
STXBP2	0.368106295	2.39E-13	2.71E-12
STXBP3	0.079974625	0.124125457	0.173531744
STXBP4	0.300480252	3.52E-09	2.09E-08
STXBP5L	0.283760489	2.67E-08	1.38E-07
STXBP5	0.307197331	1.50E-09	9.46E-09
STXBP6	-0.034439902	0.508409004	0.581330665
STYK1	0.251174491	9.56E-07	3.78E-06
STYXL1	-0.102150953	0.049290236	0.077089031
STYX	-0.278144318	5.12E-08	2.51E-07
SUB1	0.421977228	1.88E-17	4.04E-16
SUCLA2	-0.046081433	0.376120955	0.452234997
SUCLG1	0.097574799	0.060440874	0.09224723
SUCLG2	-0.319999314	2.80E-10	1.97E-09
SUCNR1	-0.265813423	2.03E-07	9.00E-07
SUDS3	0.308517934	1.27E-09	8.06E-09
SUFU	-0.043852954	0.399663456	0.475831911
SUGT1L1	-0.194650206	0.000161475	0.000428807
SUGT1P1	-0.067255266	0.196177002	0.257986549
SUGT1	-0.058002027	0.265122061	0.334691192
SULF1	0.334381754	3.84E-11	3.08E-10
SULF2	0.269369088	1.37E-07	6.25E-07
SULT1A1	-0.101287697	0.051251101	0.079742615
SULT1A2	-0.084616159	0.10368572	0.148350109
SULT1A3	0.233716028	5.37E-06	1.87E-05
SULT1B1	-0.462419758	4.69E-21	1.68E-19
SULT1C2	0.42087893	2.33E-17	4.91E-16
SULT1C3	0.166874403	0.001255199	0.002810618
SULT1C4	0.118123836	0.022874751	0.038826854
SULT1E1	-0.150012699	0.003778278	0.007642487
SULT2A1	-0.358081894	1.15E-12	1.17E-11
SULT2B1	0.071780052	0.167680212	0.225102776
SULT4A1	-0.179358041	0.000518116	0.001250988
SULT6B1	-0.047699727	0.359570995	0.435040287
SUMF1	-0.210215402	4.48E-05	0.000132214
SUMF2	-0.113383463	0.028991938	0.04796756
SUMO1P1	0.249837666	1.10E-06	4.29E-06
SUMO1P3	-0.063932246	0.219250434	0.28420243
SUMO1	-0.060896955	0.241966259	0.309399988
SUMO2	0.268980615	1.43E-07	6.51E-07
SUMO3	0.170182274	0.000998609	0.002279624
SUMO4	-0.162997546	0.001632414	0.003567849
SUN1	0.112444903	0.030356693	0.04997446
SUN2	-0.445850498	1.61E-19	4.60E-18
SUN3	0.162520519	0.001685397	0.003675587
SUOX	-0.498214521	1.14E-24	6.98E-23
SUPT16H	0.224566712	1.26E-05	4.08E-05
SUPT3H	0.311821335	8.27E-10	5.41E-09
SUPT4H1	0.066010954	0.204600109	0.267702835
SUPT5H	0.303712141	2.35E-09	1.43E-08
SUPT6H	0.027261575	0.600681748	0.667491074
SUPT7L	0.223890455	1.34E-05	4.33E-05
SUPV3L1	-0.018233907	0.726298318	0.777920166
SURF1	-0.274624833	7.64E-08	3.62E-07
SURF2	0.176196834	0.000651903	0.001547018
SURF4	0.040954033	0.431575508	0.507756881
SURF6	0.084764109	0.103080902	0.147654424

SUSD1	0.333330885	4.46E-11	3.55E-10
SUSD2	0.156695667	0.002472351	0.005224715
SUSD3	-0.006691546	0.897789077	0.919528764
SUSD4	0.363865935	4.68E-13	5.06E-12
SUSD5	0.277585341	5.45E-08	2.65E-07
SUV39H1	0.050836948	0.328808801	0.402886336
SUV39H2	0.408756748	2.25E-16	4.09E-15
SUV420H1	-0.031231955	0.548715358	0.619910817
SUV420H2	0.388643242	7.97E-15	1.13E-13
SUZ12P	0.474945203	2.85E-22	1.22E-20
SUZ12	0.21610168	2.69E-05	8.21E-05
SV2A	0.326830274	1.10E-10	8.26E-10
SV2B	0.117600459	0.023490162	0.0397664
SV2C	0.006390734	0.90236042	0.923199468
SVEP1	0.057784009	0.266925512	0.336669202
SVIL	-0.053186333	0.306919385	0.379590761
SVIP	0.257383483	5.01E-07	2.07E-06
SVOPL	0.177481279	0.000594089	0.001419458
SVOP	0.140106253	0.006874272	0.013142922
SWAP70	0.238093909	3.53E-06	1.26E-05
SYAP1	-0.107597732	0.03831187	0.061463124
SYBU	-0.291937868	1.01E-08	5.57E-08
SYCE1L	0.246774	1.50E-06	5.70E-06
SYCE1	0.019492371	0.708242	0.762642354
SYCE2	0.084925058	0.102426127	0.146811519
SYCN	0.073148914	0.159701159	0.215728675
SYCP1	0.142019332	0.006140988	0.011860471
SYCP2L	0.058249734	0.263083195	0.332571085
SYCP2	0.197793693	0.000125629	0.000340655
SYCP3	0.030221732	0.56172589	0.632100271
SYDE1	0.207973844	5.42E-05	0.000157173
SYDE2	-0.029566664	0.570241043	0.639802999
SYF2	-0.074200746	0.153766956	0.208788529
SYK	0.36533643	3.71E-13	4.08E-12
SYMPK	0.100116935	0.054013991	0.083532583
SYN1	0.322162383	2.09E-10	1.50E-09
SYN2	-0.107127416	0.039170129	0.062723842
SYN3	0.01539929	0.76751428	0.812671718
SYNCRIP	0.28589661	2.08E-08	1.09E-07
SYNC	0.23211069	6.26E-06	2.15E-05
SYNE1	-0.042976649	0.409157731	0.485395124
SYNE2	-0.172302254	0.00086059	0.001991482
SYNGAP1	0.264381553	2.37E-07	1.04E-06
SYNGR1	0.061315709	0.238737845	0.305880464
SYNGR2	0.330184997	6.93E-11	5.38E-10
SYNGR3	0.488608204	1.17E-23	6.12E-22
SYNGR4	0.104386861	0.044501032	0.070355492
SYNJ1	-0.001765887	0.972957989	0.978469632
SYNJ2BP	-0.209428242	4.79E-05	0.000140398
SYNJ2	0.207104224	5.84E-05	0.000168307
SYNM	0.092407967	0.075452768	0.112093217
SYNPO2L	0.168166317	0.001148517	0.002592769
SYNPO2	-0.145801792	0.004894202	0.009666135
SYNPO	-0.085944956	0.09835323	0.141615571
SYNPR	0.089790226	0.084143255	0.123420846
SYNRG	0.062749901	0.227910649	0.293727258
SYPL1	-0.062274028	0.231463857	0.297786435
SYPL2	-0.171046731	0.000940034	0.002156039

SYP	0.13715648	0.00815899	0.015347314
SYS1-DBNL	0.166947284	0.001248946	0.002797875
SYS1	0.119087806	0.021777717	0.037122929
SYT10	-0.04085495	0.432691615	0.508709493
SYT11	0.204566477	7.22E-05	0.000204548
SYT12	0.137820934	0.007852248	0.01482913
SYT13	0.378642222	4.30E-14	5.46E-13
SYT14L	-0.098293052	0.058564084	0.089698906
SYT14	0.053725802	0.302032044	0.374546055
SYT15	-0.060238952	0.247100759	0.315115947
SYT16	0.173058086	0.000815803	0.001896432
SYT17	0.071158455	0.171400418	0.229463015
SYT1	0.201371208	9.40E-05	0.000260857
SYT2	0.212002314	3.85E-05	0.00011464
SYT3	0.197524966	0.000128374	0.000347236
SYT4	0.083319122	0.109108952	0.155106932
SYT5	0.283803587	2.66E-08	1.37E-07
SYT6	0.297353292	5.20E-09	3.00E-08
SYT7	0.012935774	0.803878928	0.843146678
SYT8	0.34032265	1.64E-11	1.39E-10
SYT9	-0.231658471	6.53E-06	2.24E-05
SYTL1	0.385431652	1.38E-14	1.88E-13
SYTL2	0.08390235	0.106643311	0.15206843
SYTL3	0.187130669	0.000289751	0.000733006
SYTL4	-0.323827851	1.67E-10	1.22E-09
SYTL5	-0.272247019	9.98E-08	4.63E-07
SYVN1	-0.283642735	2.71E-08	1.39E-07
TAAR1	0.038423221	0.460600385	0.535454331
TAAR5	0.028934032	0.578522282	0.647562698
TAAR6	0.176321675	0.000646063	0.001534439
TAAR8	0.127991291	0.013620387	0.024320637
TAAR9	0.098176248	0.058865967	0.090105863
TAB1	-0.033639043	0.51832462	0.590803472
TAB2	-0.252279797	8.54E-07	3.39E-06
TAB3	-0.082934709	0.110758485	0.157115532
TAC1	0.076973751	0.138923345	0.191241643
TAC3	0.171495372	0.0009109	0.002093798
TAC4	-0.062103813	0.232744269	0.2992598
TACC1	0.003438362	0.947374456	0.959316556
TACC2	-0.108653673	0.036442782	0.058781726
TACC3	0.577362504	2.31E-34	1.00E-31
TACO1	-0.322218336	2.07E-10	1.49E-09
TACR1	-0.132840167	0.010424846	0.019148422
TACR2	0.134292324	0.009606948	0.017806998
TACR3	0.100449344	0.053217231	0.082454012
TACSTD2	0.245356068	1.72E-06	6.50E-06
TADA1	-0.08326398	0.109344374	0.155331476
TADA2A	0.043053021	0.408325009	0.484551515
TADA2B	-0.144781451	0.00520584	0.010206746
TADA3	-0.102288192	0.048984353	0.076664804
TAF10	0.086603776	0.095791486	0.138346961
TAF11	0.181181329	0.000453034	0.001104964
TAF12	0.043574135	0.402669895	0.478838993
TAF13	-0.111041598	0.032499598	0.053109424
TAF15	0.173935496	0.000766517	0.001791898
TAF1A	0.143747933	0.005539595	0.010791025
TAF1B	0.3074778	1.45E-09	9.15E-09
TAF1C	-0.034697933	0.505235494	0.578498851

TAF1D	0.259348571	4.07E-07	1.71E-06
TAF1L	-0.063026086	0.225866297	0.291441436
TAF1	-0.048284999	0.353699475	0.428587127
TAF2	0.18263562	0.000406661	0.000999816
TAF3	0.130131268	0.012116841	0.021914817
TAF4B	0.232771168	5.88E-06	2.03E-05
TAF4	0.275962421	6.56E-08	3.15E-07
TAF5L	0.062403416	0.230493898	0.296691829
TAF5	0.156730826	0.002466733	0.00521348
TAF6L	-0.202742817	8.39E-05	0.000235084
TAF6	0.263371857	2.65E-07	1.15E-06
TAF7L	-0.133117645	0.010263953	0.018891211
TAF7	0.154108601	0.002919178	0.00605475
TAF8	-0.116059024	0.025386484	0.042593021
TAF9B	-0.100119631	0.054007488	0.083532583
TAF9	0.27603166	6.51E-08	3.13E-07
TAGAP	0.189088152	0.000249374	0.000638483
TAGLN2	0.431016885	3.24E-18	7.74E-17
TAGLN3	0.175550249	0.000682939	0.001612791
TAGLN	0.077105638	0.13824576	0.19041289
TAL1	-0.135692345	0.008872883	0.016557285
TAL2	0.074252219	0.153480903	0.208460722
TALDO1	0.062910044	0.226723655	0.292367704
TANC1	0.22686078	1.02E-05	3.37E-05
TANC2	0.231272306	6.77E-06	2.31E-05
TANK	-0.057633071	0.26817898	0.337972017
TAOK1	-0.034945851	0.502196182	0.575614191
TAOK2	-0.042705638	0.412120822	0.488328735
TAOK3	-0.060638917	0.243970785	0.311683082
TAP1	0.345300097	7.92E-12	7.09E-11
TAP2	0.217432692	2.39E-05	7.36E-05
TAPBPL	-0.100807127	0.052370602	0.081306666
TAPBP	0.100327398	0.053508383	0.082853568
TAPT1	-0.494498625	2.83E-24	1.61E-22
TARBP1	0.117486521	0.023626016	0.039965822
TARBP2	0.139032428	0.007319347	0.013913743
TARDBP	0.197776831	0.0001258	0.000341056
TARM1	-0.029443709	0.571846132	0.641351046
TARP	0.070254626	0.176919054	0.235805272
TARS2	-0.179167424	0.000525399	0.001267498
TARSL2	-0.053730129	0.301993051	0.374521013
TARS	0.38391982	1.78E-14	2.39E-13
TAS1R1	-0.228316012	8.93E-06	2.98E-05
TAS1R2	0.039110166	0.452616344	0.528145188
TAS1R3	0.191945217	0.000199783	0.000521157
TAS2R10	0.015175728	0.7707947	0.81541148
TAS2R13	-0.003953874	0.93949842	0.953425069
TAS2R14	0.133963824	0.00978681	0.018111736
TAS2R19	0.20027392	0.000102772	0.000282951
TAS2R1	0.133012044	0.010324923	0.018986641
TAS2R20	0.138858346	0.007393877	0.01403667
TAS2R30	0.117831209	0.023217094	0.039364335
TAS2R31	0.153407456	0.003052315	0.006305945
TAS2R38	0.168767854	0.001101749	0.002496196
TAS2R3	0.003852155	0.941052054	0.954564105
TAS2R40	0.008470649	0.870826638	0.898333071
TAS2R42	0.01920505	0.712350516	0.766194093
TAS2R43	0.203458135	7.91E-05	0.000222536

TAS2R46	0.186729149	0.000298754	0.000753294
TAS2R4	0.094020522	0.070472628	0.105603946
TAS2R50	0.002627948	0.959766073	0.968467797
TAS2R5	0.108165296	0.037297397	0.060033833
TAS2R60	-0.022909121	0.660060585	0.721725692
TAS2R8	-0.001455515	0.977709523	0.982592398
TAS2R9	-0.048456337	0.351992082	0.426699906
TASP1	0.145321039	0.00503888	0.009917463
TATDN1	0.180532875	0.000475256	0.001154499
TATDN2	0.169431645	0.001052181	0.002391233
TATDN3	-0.149475665	0.003906468	0.007884171
TAT	-0.425224179	1.01E-17	2.23E-16
TAX1BP1	-0.163307596	0.0015988	0.003501296
TAX1BP3	0.360865864	7.48E-13	7.81E-12
TAZ	0.090635025	0.081254564	0.119696594
TBC1D10A	-0.205420234	6.72E-05	0.000191698
TBC1D10B	0.42511231	1.03E-17	2.28E-16
TBC1D10C	0.235402838	4.57E-06	1.61E-05
TBC1D12	0.182966966	0.000396733	0.000977456
TBC1D13	0.135152463	0.009149828	0.017038967
TBC1D14	0.107670692	0.038180165	0.061291317
TBC1D15	-0.110827998	0.032836831	0.053594573
TBC1D16	-0.030020662	0.564333058	0.634513034
TBC1D17	-0.277837229	5.30E-08	2.58E-07
TBC1D19	-0.00793621	0.878911872	0.90456591
TBC1D1	0.247573947	1.38E-06	5.29E-06
TBC1D20	0.069493625	0.181666955	0.24102085
TBC1D21	0.013332276	0.79799517	0.838484166
TBC1D22A	0.04657881	0.370985238	0.446788049
TBC1D22B	0.390647791	5.65E-15	8.24E-14
TBC1D23	-0.277083945	5.78E-08	2.80E-07
TBC1D24	-0.072607796	0.162820442	0.219421555
TBC1D25	0.141441679	0.00635464	0.012245805
TBC1D26	0.047731665	0.359249026	0.43470354
TBC1D28	0.045144539	0.385912926	0.462167557
TBC1D29	-0.103072806	0.047265891	0.074296282
TBC1D2B	-0.138148293	0.007704942	0.014581349
TBC1D2	-0.163849353	0.001541584	0.003390906
TBC1D3B	0.353046289	2.49E-12	2.41E-11
TBC1D3C	0.0993791	0.055817995	0.086041945
TBC1D3G	0.284314872	2.50E-08	1.29E-07
TBC1D3H	0.190192571	0.000228978	0.000590435
TBC1D3P2	0.101962253	0.049713423	0.077647261
TBC1D3	0.318445004	3.45E-10	2.39E-09
TBC1D4	-0.169039727	0.001081193	0.00245269
TBC1D5	-0.128020193	0.013599034	0.024291218
TBC1D7	0.15683869	0.002449571	0.005180504
TBC1D8B	-0.193511974	0.00017667	0.000465619
TBC1D8	0.061449897	0.237709755	0.304759234
TBC1D9B	-0.064224524	0.217146371	0.281896911
TBC1D9	-0.100466728	0.053175834	0.082415509
TBCA	0.050971843	0.327525349	0.401610143
TBCB	0.304463437	2.13E-09	1.31E-08
TBCCD1	0.029884956	0.566095953	0.635868492
TBCC	0.127579095	0.013928148	0.024796834
TBCD	0.079359231	0.127055721	0.177007278
TBCEL	-0.077375076	0.136869375	0.188725893
TBCE	0.114146609	0.027921348	0.046381147

TBCK	-0.275646937	6.80E-08	3.25E-07
TBK1	0.126876314	0.014466988	0.025648423
TBKBP1	0.26267509	2.85E-07	1.23E-06
TBL1XR1	0.138601477	0.007505088	0.014230167
TBL1X	-0.236685661	4.04E-06	1.43E-05
TBL1Y	0.026069274	0.616708757	0.681991222
TBL2	0.029924078	0.565587468	0.635368983
TBL3	-0.061021618	0.241001984	0.308384708
TBPL1	0.0662972	0.202639604	0.265468941
TBPL2	0.131135023	0.011463527	0.02083536
TBP	0.314807835	5.59E-10	3.74E-09
TBR1	0.169779756	0.001027015	0.002339913
TBRG1	-0.147125105	0.004515094	0.008981498
TBRG4	0.066090849	0.204051528	0.267090312
TBX10	-0.131517022	0.011223196	0.020448922
TBX15	-0.007937383	0.878894104	0.90456591
TBX18	0.097414955	0.060865229	0.092759876
TBX19	0.391368293	4.99E-15	7.36E-14
TBX1	0.284095396	2.57E-08	1.33E-07
TBX20	0.116600972	0.024705242	0.041586635
TBX21	0.016954581	0.744810483	0.793609373
TBX22	0.050786991	0.329284941	0.403378125
TBX2	0.139067728	0.007304316	0.013889146
TBX3	-0.349986335	3.94E-12	3.70E-11
TBX4	0.02920092	0.575021826	0.644195165
TBX5	0.133197104	0.010218286	0.018814186
TBX6	-0.088271301	0.089544866	0.13030773
TBXA2R	-0.004275422	0.934588723	0.949409849
TBXAS1	0.268069436	1.59E-07	7.14E-07
TC2N	0.353843538	2.20E-12	2.15E-11
TCAM1P	0.165972909	0.001334959	0.00297384
TCAP	-0.177580999	0.000589806	0.001410239
TCEA1	0.121645399	0.019085346	0.032910781
TCEA2	-0.079717641	0.12534266	0.174975578
TCEA3	-0.294669224	7.23E-09	4.09E-08
TCEAL1	-0.238944233	3.25E-06	1.17E-05
TCEAL2	0.099571783	0.055342131	0.085367861
TCEAL3	0.319323894	3.07E-10	2.14E-09
TCEAL4	-0.041608845	0.424241361	0.500606313
TCEAL5	0.283740784	2.67E-08	1.38E-07
TCEAL6	0.258139981	4.63E-07	1.93E-06
TCEAL7	0.133576822	0.010002534	0.018464648
TCEAL8	0.308106029	1.34E-09	8.48E-09
TCEANC	-0.220805215	1.77E-05	5.60E-05
TCEB1	0.3044182	2.15E-09	1.31E-08
TCEB2	0.069189174	0.183592575	0.243235438
TCEB3B	0.053828279	0.301109511	0.373541545
TCEB3C	0.009238658	0.859231584	0.889503826
TCEB3	-0.189825125	0.000235584	0.000605823
TCERG1L	0.058740838	0.25907287	0.328407338
TCERG1	0.533683417	1.07E-28	1.22E-26
TCF12	0.045284789	0.38443731	0.460649591
TCF15	-0.196548536	0.000138828	0.000373242
TCF19	0.455417353	2.14E-20	6.85E-19
TCF20	0.022298886	0.668570495	0.728983712
TCF21	-0.018101976	0.72820027	0.779621978
TCF23	-0.090383399	0.082106465	0.120797947
TCF25	-0.233403012	5.54E-06	1.92E-05

TCF3	0.397674844	1.66E-15	2.61E-14
TCF4	-0.052408727	0.314055432	0.386927169
TCF7L1	0.229994377	7.63E-06	2.58E-05
TCF7L2	-0.137312315	0.008086106	0.015227471
TCF7	-0.033000307	0.526303732	0.598323611
TCFL5	-0.05419014	0.297866888	0.370025825
TCHHL1	0.101709041	0.05028605	0.078449388
TCHH	0.009439165	0.856209279	0.887020346
TCHP	0.279306699	4.48E-08	2.21E-07
TCIRG1	0.430313631	3.73E-18	8.78E-17
TCL1A	0.144147007	0.005408507	0.010556313
TCL1B	0.170039973	0.001008567	0.002300249
TCL6	0.175658994	0.000677625	0.001601762
TCN1	0.343707662	1.00E-11	8.81E-11
TCN2	0.140773322	0.006610135	0.012690287
TCOF1	0.477804891	1.48E-22	6.54E-21
TCP10L2	0.281739344	3.38E-08	1.71E-07
TCP10L	-0.08327221	0.109309212	0.155331476
TCP10	0.257882557	4.76E-07	1.98E-06
TCP11L1	0.014953463	0.774060135	0.818080956
TCP11L2	-0.000590078	0.990962274	0.992856086
TCP11	-0.24914822	1.18E-06	4.58E-06
TCP1	0.305379845	1.90E-09	1.17E-08
TCTA	-0.217773228	2.32E-05	7.17E-05
TCTE1	-0.249617989	1.12E-06	4.38E-06
TCTE3	0.239384763	3.11E-06	1.13E-05
TCTEX1D1	0.109730909	0.034616324	0.056158502
TCTEX1D2	0.141384088	0.006376302	0.012282796
TCTEX1D4	-0.277122191	5.75E-08	2.79E-07
TCTN1	-0.189683385	0.00023818	0.000612077
TCTN2	0.266518	1.88E-07	8.37E-07
TCTN3	-0.138004969	0.007769129	0.014687473
TDGF1	-0.158970815	0.002131899	0.004564395
TDGF3	-0.152756418	0.003180851	0.006549759
TDG	0.531942972	1.72E-28	1.92E-26
TDH	-0.062222384	0.231851814	0.298227779
TDO2	-0.041022177	0.43080887	0.507124457
TDP1	0.209322505	4.84E-05	0.000141584
TDP2	-0.018417911	0.723648492	0.775832809
TDRD10	0.075914912	0.144455912	0.197695314
TDRD12	0.11490047	0.02689729	0.044874618
TDRD1	0.0782227	0.132608029	0.183625297
TDRD3	-0.214186151	3.18E-05	9.60E-05
TDRD5	0.268336218	1.54E-07	6.96E-07
TDRD6	-0.182185462	0.000420521	0.001030457
TDRD7	0.175927036	0.000664691	0.001574176
TDRD9	0.164640979	0.001461352	0.003233346
TDRG1	0.101341058	0.051128036	0.079594649
TDRKH	0.137669369	0.007921297	0.01494961
TEAD1	-0.116405181	0.024949513	0.041928947
TEAD2	0.406145812	3.62E-16	6.37E-15
TEAD3	0.248617776	1.24E-06	4.80E-06
TEAD4	0.32147598	2.29E-10	1.63E-09
TECPR1	-0.049805904	0.338725738	0.412803215
TECPR2	-0.23280992	5.86E-06	2.02E-05
TECRL	-0.013811076	0.790905414	0.832791631
TECR	-0.010482289	0.840520806	0.873629441
TECTA	-0.027416187	0.598617237	0.665568289

TECTB	-0.185150291	0.000336745	0.000841524
TEC	-0.314121562	6.12E-10	4.08E-09
TEDDM1	-0.225880482	1.12E-05	3.66E-05
TEF	-0.307135879	1.52E-09	9.52E-09
TEKT1	-0.023407733	0.653139582	0.715605057
TEKT2	0.230801767	7.08E-06	2.40E-05
TEKT3	0.073819901	0.155896015	0.211290592
TEKT4	0.000112689	0.998274006	0.998624894
TEKT5	-0.08337207	0.108883272	0.154841356
TEK	-0.137075758	0.008196973	0.015414395
TELO2	0.200569904	0.000100322	0.000277086
TENC1	-0.124255845	0.016640911	0.029080722
TEP1	0.265369568	2.13E-07	9.41E-07
TEPP	0.039181918	0.451786929	0.527517243
TERC	0.07475645	0.150699973	0.205211542
TERF1	0.047059961	0.366058526	0.441789419
TERF2IP	-0.176155561	0.000653844	0.00155107
TERF2	-0.163970739	0.001529025	0.003365138
TERT	0.134320733	0.009591532	0.017781733
TESC	0.3521909	2.83E-12	2.71E-11
TESK1	0.222094749	1.58E-05	5.02E-05
TESK2	-0.280666697	3.83E-08	1.92E-07
TES	0.348856351	4.67E-12	4.33E-11
TET1	0.371756619	1.33E-13	1.57E-12
TET2	-0.110264455	0.033740913	0.054890288
TET3	0.254149243	7.03E-07	2.84E-06
TEX101	0.004844087	0.92591198	0.942132819
TEX10	0.388246824	8.53E-15	1.21E-13
TEX11	0.074658346	0.151238013	0.205803531
TEX12	0.094730416	0.068367028	0.102948445
TEX13A	-0.096763492	0.062620247	0.095157937
TEX13B	0.069128615	0.183977393	0.243680448
TEX14	0.003766783	0.942356163	0.955546367
TEX15	0.292637781	9.26E-09	5.15E-08
TEX19	0.363014856	5.35E-13	5.73E-12
TEX261	0.057738246	0.267305125	0.337041311
TEX264	-0.088364947	0.089203987	0.129859104
TEX2	-0.020131158	0.699138119	0.755163175
TEX9	0.056302393	0.279403924	0.350058799
TFAMP1	0.101465046	0.050843034	0.079188094
TFAM	0.026614967	0.60935029	0.675203096
TFAP2A	0.248819166	1.22E-06	4.72E-06
TFAP2B	0.159425965	0.00206916	0.004442483
TFAP2C	0.276503827	6.17E-08	2.98E-07
TFAP2D	0.024001676	0.64493402	0.708250678
TFAP2E	0.393589109	3.39E-15	5.13E-14
TFAP4	0.214119949	3.20E-05	9.66E-05
TFB1M	-0.172295849	0.000860979	0.001991919
TFB2M	-0.158022461	0.002268239	0.004825184
TFCP2L1	0.343014444	1.11E-11	9.69E-11
TFCP2	0.144574754	0.005271104	0.010320485
TFDP1	0.382971746	2.09E-14	2.77E-13
TFDP2	0.069024457	0.184640647	0.244423735
TFDP3	-0.105252098	0.042755747	0.067845064
TFE3	0.309495013	1.12E-09	7.17E-09
TFEB	0.083821276	0.106983402	0.152455174
TFEC	0.237786312	3.64E-06	1.30E-05
TFF1	0.275457246	6.95E-08	3.32E-07

TFF2	0.246260779	1.58E-06	5.98E-06
TFF3	0.18996999	0.000232959	0.000599923
TFG	0.085462986	0.100261596	0.144092592
TFIP11	0.084712983	0.10328959	0.147900181
TFPI2	0.018591111	0.721157268	0.773953299
TFPI	-0.197775669	0.000125812	0.000341056
TFPT	0.228721541	8.60E-06	2.88E-05
TFR2	-0.442653269	3.11E-19	8.56E-18
TFRC	0.27919595	4.53E-08	2.24E-07
TF	-0.244486774	1.88E-06	7.05E-06
TGDS	-0.037907401	0.466646702	0.541535247
TGFA	0.359666107	9.01E-13	9.28E-12
TGFB1I1	0.117783102	0.023273796	0.039447044
TGFB1	0.29898453	4.25E-09	2.49E-08
TGFB2	0.127712286	0.013828041	0.024642833
TGFB3	0.214353994	3.14E-05	9.48E-05
TGFBI	-0.019633667	0.706224631	0.76103606
TGFBR1	0.137562085	0.0079705	0.0150325
TGFBR2	-0.262642394	2.86E-07	1.24E-06
TGFBR3	-0.373153025	1.06E-13	1.27E-12
TGFBRAP1	-0.035106496	0.500231931	0.573924241
TGIF1	-0.034624255	0.506140591	0.579268849
TGIF2LX	-0.039063654	0.453154465	0.528680209
TGIF2LY	-0.033814577	0.516142804	0.58878819
TGIF2	0.362372025	5.91E-13	6.28E-12
TGM1	0.285936511	2.07E-08	1.08E-07
TGM2	0.10298037	0.047465683	0.074580909
TGM3	0.044632912	0.391325063	0.467721257
TGM4	0.011460588	0.825864279	0.861632268
TGM5	0.144829274	0.005190844	0.010184859
TGM6	-0.033290207	0.522674568	0.594841129
TGM7	0.049443689	0.342254562	0.416543184
TGOLN2	-0.218639063	2.15E-05	6.68E-05
TGS1	0.226870938	1.02E-05	3.37E-05
TG	-0.126128959	0.015060007	0.026586217
TH1L	0.118832012	0.022064282	0.037547162
THADA	-0.017119314	0.742418243	0.791695549
THAP10	-0.094230371	0.069844756	0.104817116
THAP11	-0.034999548	0.501539165	0.575048024
THAP1	0.054654482	0.293740094	0.36553752
THAP2	-0.032014382	0.538741449	0.610481059
THAP3	0.038728724	0.457040023	0.532091593
THAP4	0.176221978	0.000650723	0.001544401
THAP5	-0.365547222	3.59E-13	3.96E-12
THAP6	-0.209618509	4.72E-05	0.000138284
THAP7	-0.093474278	0.072128649	0.1077818
THAP8	0.261172666	3.35E-07	1.43E-06
THAP9	-0.258758171	4.34E-07	1.81E-06
THBD	0.010298988	0.843273252	0.875894535
THBS1	0.16750733	0.001201845	0.002702082
THBS2	0.197589081	0.000127714	0.000345649
THBS3	-0.11035318	0.033597183	0.054687727
THBS4	-0.264514633	2.34E-07	1.03E-06
THEG	0.142071599	0.006121978	0.011826049
THEM4	0.188364404	0.000263648	0.000671834
THEM5	-0.078390053	0.131778902	0.182629516
THEMIS	0.125867508	0.015272444	0.026918307
THG1L	-0.049475151	0.341947119	0.416219863

THNSL1	-0.515456034	1.44E-26	1.21E-24
THNSL2	-0.325162909	1.39E-10	1.02E-09
THOC1	0.330107174	7.01E-11	5.43E-10
THOC2	0.147099094	0.004522283	0.0089949
THOC3	0.306564113	1.63E-09	1.02E-08
THOC4	0.351236403	3.27E-12	3.10E-11
THOC5	0.266731675	1.84E-07	8.20E-07
THOC6	0.154791023	0.002794687	0.005821997
THOC7	0.278966028	4.66E-08	2.29E-07
THOP1	-0.11164695	0.031559869	0.051717998
THPO	-0.102518001	0.0484757	0.07601447
THRAP3	-0.034176833	0.511655164	0.584405124
THRA	-0.112965624	0.029592879	0.048844187
THRB	-0.31662505	4.40E-10	3.00E-09
THRSP	-0.322781963	1.92E-10	1.39E-09
THSD1P1	-0.311245005	8.92E-10	5.81E-09
THSD1	-0.332483701	5.02E-11	3.96E-10
THSD4	-0.049576782	0.340955213	0.415190083
THSD7A	0.068558798	0.187627423	0.247956398
THSD7B	0.032156664	0.5369375	0.608748266
THTPA	-0.254094988	7.07E-07	2.85E-06
THUMPD1	-0.062986492	0.226158571	0.291733427
THUMPD2	0.346439915	6.69E-12	6.07E-11
THUMPD3	0.152460836	0.003240815	0.00665466
THY1	0.269196482	1.40E-07	6.36E-07
THYN1	-0.13313921	0.010251541	0.018871854
TH	0.114026617	0.028087398	0.046621992
TIA1	0.216750521	2.54E-05	7.79E-05
TIAF1	-0.071897513	0.166984059	0.224289133
TIAL1	0.156811078	0.002453954	0.005188672
TIAM1	-0.025815267	0.620147127	0.685033051
TIAM2	0.347922901	5.37E-12	4.92E-11
TICAM1	0.140608622	0.006674491	0.012794113
TICAM2	0.081969537	0.114986552	0.162354219
TIE1	-0.157121039	0.002405159	0.005095233
TIFAB	0.283498043	2.75E-08	1.42E-07
TIFA	0.052426117	0.313894668	0.386800865
TIGD1	0.329686313	7.43E-11	5.74E-10
TIGD2	-0.218208313	2.24E-05	6.92E-05
TIGD3	0.144506009	0.005292973	0.010358213
TIGD4	0.041031006	0.4307096	0.507037562
TIGD5	0.348192269	5.16E-12	4.74E-11
TIGD6	0.006348217	0.903006796	0.923685633
TIGD7	0.145084472	0.005111476	0.010047031
TIGIT	0.219317186	2.03E-05	6.32E-05
TIMD4	0.111982011	0.031049782	0.050944964
TIMELESS	0.328964989	8.21E-11	6.28E-10
TIMM10	0.007057791	0.892227952	0.915056126
TIMM13	0.112395906	0.030429424	0.050075581
TIMM16	0.038130651	0.464024451	0.538900263
TIMM17A	-0.124342661	0.01656455	0.028962522
TIMM17B	0.287449388	1.73E-08	9.16E-08
TIMM22	0.15959603	0.002046154	0.004396405
TIMM44	0.191408078	0.000208338	0.000542125
TIMM50	0.256496895	5.50E-07	2.26E-06
TIMM8A	-0.160000451	0.00199238	0.00428873
TIMM8B	0.053755735	0.301762388	0.374304856
TIMM9	0.167457873	0.001205937	0.00271006

TIMP1	0.318398574	3.47E-10	2.40E-09
TIMP2	0.331398307	5.85E-11	4.57E-10
TIMP3	-0.361158553	7.15E-13	7.49E-12
TIMP4	-0.123180334	0.017612726	0.030593838
TINAGL1	-0.013236572	0.799414303	0.839533075
TINAG	0.329382179	7.75E-11	5.96E-10
TINF2	-0.079857587	0.124678664	0.174180217
TIPARP	-0.196834623	0.000135685	0.000365335
TIPIN	0.413434838	9.47E-17	1.84E-15
TIPRL	0.342846044	1.14E-11	9.92E-11
TIRAP	-0.078662156	0.130439361	0.181037547
TJAP1	0.185227158	0.000334795	0.000837493
TJP1	-0.138089921	0.007731027	0.014625156
TJP2	0.031222733	0.548833451	0.62000879
TJP3	0.374960961	7.88E-14	9.64E-13
TK1	0.33913516	1.95E-11	1.63E-10
TK2	-0.468521531	1.22E-21	4.69E-20
TKTL1	0.101512236	0.050734911	0.079044413
TKTL2	0.144546824	0.005279979	0.010335662
TKT	0.198172344	0.000121856	0.000331052
TLCD1	0.335204518	3.42E-11	2.76E-10
TLE1	-0.095504761	0.066129285	0.09988079
TLE2	-0.130501358	0.011872232	0.021513425
TLE3	0.24592108	1.63E-06	6.16E-06
TLE4	-0.025516703	0.624199239	0.689165221
TLE6	0.012229255	0.814390359	0.851936816
TLK1	-0.005325037	0.918580409	0.936442478
TLK2	0.239892681	2.96E-06	1.08E-05
TLL1	-0.137130443	0.008171225	0.015368876
TLL2	0.303949859	2.28E-09	1.39E-08
TLN1	0.183219866	0.000389307	0.000961063
TLN2	-0.072614389	0.162782165	0.219399654
TLR10	0.312456635	7.61E-10	5.00E-09
TLR1	0.134724157	0.009374976	0.017412668
TLR2	0.239070323	3.21E-06	1.16E-05
TLR3	-0.127335586	0.014112821	0.025083025
TLR4	0.00059654	0.990863299	0.992856086
TLR5	0.295579085	6.47E-09	3.69E-08
TLR6	0.108233493	0.037177047	0.059859463
TLR7	0.273499148	8.67E-08	4.07E-07
TLR8	0.149525872	0.003894319	0.007862041
TLR9	0.34915282	4.47E-12	4.16E-11
TLX1NB	-0.070465688	0.175618691	0.234242262
TLX1	-0.135437925	0.009002451	0.016784916
TLX2	0.134136153	0.009692087	0.01794644
TLX3	0.187547098	0.000280682	0.000712144
TM2D1	0.01353972	0.794921407	0.835963556
TM2D2	0.004912382	0.924870506	0.941313488
TM2D3	-0.237448635	3.76E-06	1.34E-05
TM4SF18	-0.252949377	7.97E-07	3.18E-06
TM4SF19	0.245885617	1.64E-06	6.18E-06
TM4SF1	0.196923346	0.000134724	0.000362895
TM4SF20	0.214741026	3.03E-05	9.19E-05
TM4SF4	-0.08217044	0.114096208	0.161225015
TM4SF5	-0.008921435	0.86401735	0.893158657
TM6SF1	0.251805733	8.96E-07	3.55E-06
TM6SF2	-0.502745267	3.69E-25	2.51E-23
TM7SF2	-0.360673594	7.71E-13	8.04E-12

TM7SF3	-0.155005233	0.002756618	0.005750506
TM7SF4	0.067485443	0.194647011	0.256177429
TM9SF1	-0.184574956	0.000351674	0.000875537
TM9SF2	-0.082385213	0.113150381	0.160120891
TM9SF3	-0.019644946	0.706063687	0.760911002
TM9SF4	0.144434672	0.005315752	0.010398706
TMBIM1	0.085640757	0.099554317	0.143149158
TMBIM4	-0.07555979	0.146348649	0.199983386
TMBIM6	-0.409996078	1.79E-16	3.31E-15
TMC1	-0.031562374	0.544492313	0.615838302
TMC2	0.202280311	8.72E-05	0.000243594
TMC3	-0.158456162	0.00220493	0.004703567
TMC4	0.301622574	3.05E-09	1.83E-08
TMC5	0.407233762	2.97E-16	5.30E-15
TMC6	0.44093386	4.42E-19	1.20E-17
TMC7	0.390916089	5.39E-15	7.89E-14
TMC8	0.223609855	1.38E-05	4.43E-05
TMCC1	-0.208438093	5.21E-05	0.000151624
TMCC2	0.318013747	3.65E-10	2.52E-09
TMCC3	0.206985859	5.90E-05	0.000169844
TMCO1	0.153281528	0.003076803	0.0063539
TMCO2	0.067764058	0.192806735	0.25399005
TMCO3	0.402231933	7.35E-16	1.24E-14
TMCO4	0.136175281	0.008631496	0.016146166
TMCO5A	0.175738429	0.000673768	0.001593211
TMCO6	-0.171586275	0.000905099	0.002081906
TMCO7	-0.442241646	3.39E-19	9.26E-18
TMED10P1	-0.022358696	0.667734535	0.728151919
TMED10	-0.190302908	0.000227028	0.000585938
TMED1	-0.090153164	0.082892249	0.121855032
TMED2	0.071657119	0.168411125	0.225916533
TMED3	0.391154729	5.17E-15	7.61E-14
TMED4	-0.158695991	0.002170618	0.004635833
TMED5	-0.105324808	0.042611745	0.067642325
TMED6	-0.112201648	0.030719251	0.050476156
TMED7-TIC	0.046101889	0.375908871	0.452007274
TMED7	-0.033957222	0.514373295	0.587038541
TMED8	0.166076569	0.001325556	0.002955207
TMED9	0.310501311	9.83E-10	6.36E-09
TMEFF1	0.317280692	4.03E-10	2.76E-09
TMEFF2	0.0182992	0.725357665	0.777163354
TMEM100	-0.321666231	2.24E-10	1.60E-09
TMEM101	0.050974663	0.327498553	0.401610143
TMEM102	0.143559449	0.005602495	0.010903958
TMEM104	0.125474944	0.015596348	0.027433379
TMEM105	0.045818212	0.378856461	0.454974888
TMEM106A	0.155654042	0.002644171	0.005550809
TMEM106B	-0.243233534	2.13E-06	7.91E-06
TMEM106C	0.412183409	1.20E-16	2.28E-15
TMEM107	0.31619759	4.65E-10	3.16E-09
TMEM108	0.294178587	7.67E-09	4.33E-08
TMEM109	0.017844163	0.73192174	0.782848969
TMEM110	-0.220834067	1.77E-05	5.58E-05
TMEM111	-0.104812544	0.043635038	0.069068586
TMEM114	0.185056339	0.000339142	0.00084677
TMEM115	0.106527666	0.040288128	0.064379569
TMEM116	0.060235908	0.24712469	0.315120195
TMEM117	0.043581887	0.402586121	0.478796532

TMEM119	0.258619743	4.40E-07	1.84E-06
TMEM11	0.208289599	5.28E-05	0.000153364
TMEM120A	-0.200275464	0.00010276	0.000282951
TMEM120B	0.366981624	2.86E-13	3.20E-12
TMEM121	0.13186792	0.011006371	0.020092378
TMEM123	0.016505736	0.751340974	0.79897124
TMEM125	0.055954205	0.282392835	0.353225973
TMEM126A	0.078416701	0.131647249	0.182485144
TMEM126B	0.159489008	0.002060604	0.004425545
TMEM127	-0.115701848	0.025844261	0.043299081
TMEM128	0.205962316	6.42E-05	0.000183886
TMEM129	-0.188118116	0.000268677	0.000684036
TMEM130	0.250885528	9.85E-07	3.89E-06
TMEM131	0.09316138	0.07309146	0.109065094
TMEM132A	0.363793652	4.73E-13	5.11E-12
TMEM132B	-0.049612999	0.340602183	0.414836259
TMEM132C	0.083706837	0.107464909	0.153053754
TMEM132D	0.065839645	0.205779961	0.269016889
TMEM132E	0.110863514	0.032780553	0.053520258
TMEM133	-0.189961765	0.000233107	0.000600227
TMEM134	-0.173670828	0.000781086	0.001822747
TMEM135	-0.183819216	0.000372222	0.000921971
TMEM136	0.354140105	2.11E-12	2.06E-11
TMEM138	0.18939555	0.000243533	0.000624732
TMEM139	0.044601182	0.391662215	0.468011915
TMEM140	-0.336613054	2.79E-11	2.29E-10
TMEM141	0.09312394	0.073207363	0.109200183
TMEM143	-0.375715574	6.97E-14	8.59E-13
TMEM144	0.130448389	0.011906972	0.021574415
TMEM145	0.173504469	0.000790374	0.001842911
TMEM146	0.144121535	0.00541679	0.010570408
TMEM147	0.41602766	5.83E-17	1.16E-15
TMEM149	-0.004888765	0.925230644	0.941487633
TMEM14A	-0.129959337	0.012231984	0.022096988
TMEM14B	0.060543541	0.244714638	0.312453055
TMEM14C	0.020904299	0.688176913	0.745546819
TMEM14E	0.122605204	0.018152461	0.03144911
TMEM150A	-0.076654335	0.140574941	0.193193569
TMEM150B	0.035156642	0.499619626	0.573320788
TMEM150C	-0.445489046	1.73E-19	4.92E-18
TMEM151A	0.264150764	2.43E-07	1.06E-06
TMEM151B	0.128753043	0.013067347	0.023440274
TMEM154	0.181212999	0.000451974	0.001102648
TMEM155	0.331736108	5.58E-11	4.37E-10
TMEM156	0.322664342	1.95E-10	1.41E-09
TMEM158	0.310244496	1.02E-09	6.56E-09
TMEM159	0.275357639	7.03E-08	3.35E-07
TMEM160	0.04808432	0.355705877	0.430703974
TMEM161A	-0.06493467	0.212094502	0.276148401
TMEM161B	0.084193076	0.105430779	0.150565733
TMEM163	0.268117885	1.58E-07	7.11E-07
TMEM164	0.357732251	1.22E-12	1.23E-11
TMEM165	0.454383483	2.67E-20	8.48E-19
TMEM167A	0.083357411	0.108945718	0.1549191
TMEM167B	-0.089848281	0.083942131	0.12320748
TMEM168	0.082237155	0.113801744	0.160871237
TMEM169	-0.189711056	0.000237671	0.000610953
TMEM170A	-0.128925035	0.012945251	0.023235903

TMEM170B	-0.223496364	1.39E-05	4.47E-05
TMEM171	0.178048135	0.000570118	0.00136661
TMEM173	0.133612244	0.009982615	0.018433002
TMEM174	0.028194746	0.588270355	0.655931158
TMEM175	-0.181222004	0.000451673	0.001102183
TMEM176A	-0.21445341	3.11E-05	9.40E-05
TMEM176B	-0.378375133	4.50E-14	5.69E-13
TMEM177	-0.213306346	3.44E-05	0.000103144
TMEM178	-0.053272463	0.30613561	0.378691936
TMEM179B	-0.058124803	0.264110146	0.333646311
TMEM179	0.155328429	0.002700071	0.005648776
TMEM17	0.007633415	0.88349832	0.908019683
TMEM180	0.041505329	0.425395929	0.501820091
TMEM181	0.16416706	0.001508911	0.003326751
TMEM182	0.052532453	0.312912792	0.38575796
TMEM183A	0.16823168	0.001143348	0.002583101
TMEM184A	0.13725109	0.008114672	0.015268274
TMEM184B	0.348928571	4.62E-12	4.28E-11
TMEM184C	-0.175288324	0.000695896	0.001640667
TMEM185A	0.19377074	0.000173102	0.000457003
TMEM185B	0.319598371	2.95E-10	2.07E-09
TMEM186	-0.103350027	0.046670922	0.073430588
TMEM187	-0.275655632	6.80E-08	3.25E-07
TMEM188	0.028474509	0.584572575	0.652831147
TMEM189-U	0.074633087	0.151376783	0.205964231
TMEM189	0.416310523	5.53E-17	1.11E-15
TMEM18	0.023045479	0.658164949	0.720120942
TMEM190	0.248294245	1.28E-06	4.95E-06
TMEM191A	0.146323847	0.004741343	0.009394972
TMEM192	-0.519054047	5.58E-27	4.94E-25
TMEM194A	0.368104462	2.39E-13	2.71E-12
TMEM194B	0.187640014	0.000278695	0.000707643
TMEM195	-0.519062742	5.57E-27	4.94E-25
TMEM196	0.058769088	0.258843468	0.328164728
TMEM198	0.137458099	0.00801845	0.015112919
TMEM199	0.107423939	0.038627145	0.061909089
TMEM19	-0.097286164	0.061208933	0.093233761
TMEM200A	0.307476308	1.45E-09	9.15E-09
TMEM200B	0.061515692	0.237206793	0.304173129
TMEM200C	0.258928296	4.26E-07	1.78E-06
TMEM201	0.469697513	9.34E-22	3.69E-20
TMEM202	-0.22337559	1.41E-05	4.52E-05
TMEM203	-0.078436789	0.131548074	0.182360359
TMEM204	-0.068983559	0.184901556	0.244701923
TMEM205	-0.231639945	6.54E-06	2.24E-05
TMEM206	0.380157044	3.34E-14	4.31E-13
TMEM207	0.155156211	0.00273007	0.005704085
TMEM208	-0.146615135	0.004657937	0.009243492
TMEM209	0.009252077	0.859029247	0.889340573
TMEM20	0.141828711	0.006210768	0.011988269
TMEM211	0.047934179	0.357211664	0.432422116
TMEM212	0.101452947	0.050870786	0.079225124
TMEM213	0.175655527	0.000677794	0.001601971
TMEM214	0.052148251	0.316469916	0.389690264
TMEM215	0.044796723	0.38958725	0.466035619
TMEM216	0.235055789	4.73E-06	1.66E-05
TMEM217	0.131477897	0.011247605	0.020484029
TMEM218	0.009404119	0.856737387	0.887475157

TMEM219	-0.201756076	9.10E-05	0.000253215
TMEM220	-0.370657472	1.59E-13	1.85E-12
TMEM222	-0.129420159	0.012599359	0.02268659
TMEM223	0.175554576	0.000682727	0.001612481
TMEM229A	0.098973693	0.05683031	0.087392777
TMEM229B	0.22519606	1.19E-05	3.88E-05
TMEM22	0.132109319	0.010859367	0.019855021
TMEM231	-0.033022044	0.526031166	0.598047874
TMEM232	-0.309719655	1.09E-09	6.98E-09
TMEM233	-0.157251714	0.002384854	0.005057058
TMEM25	-0.278304622	5.02E-08	2.46E-07
TMEM26	0.132197419	0.010806152	0.019770425
TMEM27	-0.143118477	0.00575217	0.011165811
TMEM2	-0.146044444	0.00482261	0.009542712
TMEM30A	-0.093383324	0.072407451	0.108149741
TMEM30B	-0.07089608	0.172989045	0.23124792
TMEM30C	0.080179419	0.123162045	0.172366298
TMEM31	0.050880471	0.328394352	0.402502294
TMEM33	0.039323269	0.450155471	0.525889714
TMEM35	0.272868009	9.31E-08	4.34E-07
TMEM37	-0.090096535	0.083086449	0.122113482
TMEM38A	-0.068495227	0.188037911	0.248447491
TMEM38B	-0.004085544	0.937487666	0.951917904
TMEM39A	0.059977297	0.249163466	0.31723874
TMEM39B	0.063060689	0.225611083	0.291215757
TMEM40	-0.014816949	0.776067732	0.819811303
TMEM41A	0.064765237	0.21329207	0.277508139
TMEM41B	0.086466185	0.09632205	0.139042669
TMEM42	-0.136956059	0.008253587	0.015510609
TMEM43	0.29045695	1.20E-08	6.59E-08
TMEM44	0.438923341	6.66E-19	1.77E-17
TMEM45A	0.134959248	0.009250794	0.017204473
TMEM45B	0.103945386	0.045414362	0.071634593
TMEM47	-0.265252743	2.16E-07	9.53E-07
TMEM48	0.23256193	6.00E-06	2.07E-05
TMEM49	0.279327278	4.47E-08	2.21E-07
TMEM50A	0.109814474	0.034477952	0.055965921
TMEM50B	-0.022908705	0.660066364	0.721725692
TMEM51	0.469224705	1.04E-21	4.05E-20
TMEM52	-0.022572423	0.664750613	0.725637943
TMEM53	-0.303542554	2.40E-09	1.46E-08
TMEM54	0.22377024	1.36E-05	4.37E-05
TMEM55A	0.380221293	3.31E-14	4.27E-13
TMEM55B	0.111508681	0.031772443	0.052040666
TMEM56	-0.49805152	1.18E-24	7.24E-23
TMEM57	-0.289121595	1.41E-08	7.65E-08
TMEM59L	0.183932117	0.000369083	0.000915105
TMEM59	-0.189951789	0.000233287	0.000600536
TMEM5	0.149991684	0.003783222	0.007650933
TMEM60	0.119846495	0.020946655	0.035828465
TMEM61	0.44583236	1.61E-19	4.61E-18
TMEM62	0.145485486	0.004988964	0.009833163
TMEM63A	0.077776221	0.134839724	0.186327043
TMEM63B	-0.113334851	0.029061309	0.048054399
TMEM63C	0.241230249	2.60E-06	9.53E-06
TMEM64	-0.086078541	0.09782944	0.140946765
TMEM65	0.526028737	8.66E-28	8.76E-26
TMEM66	-0.135911395	0.008762661	0.016366934

TMEM67	0.129429889	0.012592645	0.022676549
TMEM68	0.137905776	0.007813832	0.014766378
TMEM69	0.162925462	0.001640321	0.003583559
TMEM70	0.009458874	0.855912313	0.886758807
TMEM71	0.187862898	0.000273982	0.000696741
TMEM72	0.290908834	1.14E-08	6.26E-08
TMEM74	0.049233202	0.344315916	0.418591669
TMEM79	0.06133217	0.238611562	0.305757961
TMEM80	0.079814112	0.124884643	0.174409524
TMEM81	0.13250518	0.010622061	0.019465799
TMEM82	-0.175494979	0.000685654	0.00161882
TMEM84	0.122594822	0.018162334	0.03146348
TMEM85	-0.033921618	0.514814671	0.587474961
TMEM86A	0.185909014	0.000317959	0.000798182
TMEM86B	-0.147780939	0.004337208	0.008667454
TMEM87A	0.284596269	2.42E-08	1.26E-07
TMEM87B	0.365994187	3.34E-13	3.71E-12
TMEM88B	0.159394882	0.00207339	0.004450127
TMEM88	-0.358601334	1.06E-12	1.09E-11
TMEM89	0.095973613	0.064803833	0.098101973
TMEM8A	0.072559286	0.163102298	0.219727008
TMEM8B	-0.032586303	0.531508666	0.603448775
TMEM8C	0.063224057	0.22440893	0.290067993
TMEM90A	0.110150693	0.033925969	0.055173319
TMEM90B	0.125769006	0.015353159	0.027048605
TMEM91	0.249075932	1.19E-06	4.60E-06
TMEM92	0.16161992	0.001789744	0.003886982
TMEM93	0.015014796	0.773158658	0.817345014
TMEM95	0.000763272	0.98830977	0.991145594
TMEM97	0.071133314	0.171552166	0.229620972
TMEM98	0.126949728	0.014409856	0.025563058
TMEM99	0.078157382	0.132932725	0.184049326
TMEM9B	0.088235356	0.089675983	0.130489002
TMEM9	0.257499863	4.95E-07	2.05E-06
TMF1	0.003517321	0.94616776	0.958435659
TMIE	0.126668951	0.014629437	0.02590876
TMIGD1	0.157221984	0.00238946	0.005064668
TMIGD2	0.059008312	0.256906523	0.32591007
TMLHE	-0.205043088	6.94E-05	0.000197177
TMOD1	-0.364081609	4.52E-13	4.90E-12
TMOD2	0.099844984	0.054673188	0.084457007
TMOD3	0.038553996	0.459074425	0.534121476
TMOD4	0.190327298	0.000226599	0.000584907
TMPO	0.263498817	2.61E-07	1.14E-06
TMPPE	0.154428134	0.002860273	0.005947433
TMPRSS11A	-0.080581803	0.121286054	0.170087341
TMPRSS11E	0.069659548	0.180623812	0.239888847
TMPRSS11E	0.04023892	0.439668027	0.515390787
TMPRSS11D	0.053602247	0.303146815	0.375717983
TMPRSS11F	0.082678583	0.111868379	0.158498105
TMPRSS12	0.045803116	0.379013716	0.455136302
TMPRSS13	0.287001923	1.82E-08	9.64E-08
TMPRSS15	0.044997232	0.387466498	0.463749328
TMPRSS2	-0.031902519	0.540161839	0.611808753
TMPRSS3	0.263134391	2.72E-07	1.18E-06
TMPRSS4	0.303878052	2.30E-09	1.40E-08
TMPRSS5	-0.008278605	0.873730458	0.9004893
TMPRSS6	-0.273784098	8.40E-08	3.95E-07

TMPRSS7	0.342365471	1.22E-11	1.06E-10
TMPRSS9	-0.017797432	0.732596969	0.783299146
TMSB10	0.473650968	3.82E-22	1.59E-20
TMSB15A	0.18745143	0.000282742	0.000717096
TMSB15B	0.23914241	3.19E-06	1.15E-05
TMSB4Y	-0.099772226	0.054850679	0.084695027
TMSL3	0.341285836	1.43E-11	1.22E-10
TMTC1	-0.089526891	0.085060438	0.124628524
TMTC2	0.13558709	0.008926283	0.016652254
TMTC3	0.037523418	0.471175996	0.545964532
TMTC4	-0.023594897	0.650549263	0.713394749
TMUB1	-0.083415088	0.10870019	0.154614108
TMUB2	0.079470321	0.126522824	0.176388223
TMX1	0.069994278	0.178532896	0.237590671
TMX2	0.050103005	0.335848705	0.409848569
TMX3	0.149043831	0.004012386	0.008072586
TMX4	0.006441627	0.901586792	0.922564697
TNC	0.215170517	2.92E-05	8.86E-05
TNFAIP1	-0.217081846	2.47E-05	7.58E-05
TNFAIP2	0.278261281	5.05E-08	2.48E-07
TNFAIP3	0.108314337	0.037034806	0.059659368
TNFAIP6	0.436776774	1.03E-18	2.64E-17
TNFAIP8L1	-0.187594531	0.000279666	0.000710018
TNFAIP8L2	0.292696474	9.19E-09	5.12E-08
TNFAIP8L3	0.341788513	1.32E-11	1.14E-10
TNFAIP8	0.368585118	2.21E-13	2.53E-12
TNFRSF10A	0.081567887	0.116782862	0.164548283
TNFRSF10B	0.016652846	0.74919861	0.797261616
TNFRSF10C	0.162607139	0.001675661	0.003654753
TNFRSF10D	-0.112860953	0.029745079	0.049062881
TNFRSF11A	0.330957883	6.22E-11	4.85E-10
TNFRSF11B	0.113791799	0.028414801	0.047118334
TNFRSF12A	0.117143653	0.02403894	0.040574748
TNFRSF13B	0.245879625	1.64E-06	6.19E-06
TNFRSF13C	0.24652576	1.53E-06	5.83E-06
TNFRSF14	0.13672197	0.008365312	0.015690965
TNFRSF17	0.165929148	0.001338947	0.002982057
TNFRSF18	0.412765197	1.07E-16	2.07E-15
TNFRSF19	-0.073574453	0.157279917	0.212899732
TNFRSF1A	-0.134810048	0.009329435	0.017338373
TNFRSF1B	0.070379439	0.176149216	0.234889202
TNFRSF21	0.360643749	7.74E-13	8.07E-12
TNFRSF25	0.267586148	1.67E-07	7.51E-07
TNFRSF4	0.290830074	1.15E-08	6.31E-08
TNFRSF6B	0.165757423	0.001354701	0.003014786
TNFRSF8	0.297869645	4.88E-09	2.83E-08
TNFRSF9	0.180795634	0.000466132	0.001134272
TNFSF10	-0.011955486	0.818472517	0.855578208
TNFSF11	-0.035749421	0.49241153	0.566485101
TNFSF12-TN	0.270389172	1.23E-07	5.63E-07
TNFSF12	0.184458387	0.000354773	0.000882591
TNFSF13B	0.253320437	7.67E-07	3.07E-06
TNFSF13	0.183970746	0.000368015	0.000912797
TNFSF14	0.13719792	0.008139552	0.015312196
TNFSF15	0.384481015	1.62E-14	2.19E-13
TNFSF18	0.15918591	0.002102036	0.004505509
TNFSF4	0.329140552	8.02E-11	6.15E-10
TNFSF8	0.125735593	0.015380623	0.027094595

TNFSF9	0.313994777	6.22E-10	4.14E-09
TNF	0.211905675	3.88E-05	0.000115512
TNIK	0.294082135	7.77E-09	4.38E-08
TNIP1	0.037452806	0.472011528	0.54680547
TNIP2	0.210008555	4.56E-05	0.000134253
TNIP3	0.229212661	8.21E-06	2.76E-05
TNK1	0.127282829	0.014153113	0.025149121
TNK2	0.184502588	0.000353595	0.00087988
TNKS1BP1	-0.115519816	0.026080287	0.043646798
TNKS2	-0.115894995	0.025595837	0.042913021
TNKS	-0.014509086	0.780600674	0.823813457
TNMD	-0.273071467	9.10E-08	4.26E-07
TNNC1	0.061339083	0.238558543	0.305709719
TNNC2	-0.214496179	3.10E-05	9.37E-05
TNNI1	0.135487412	0.008977118	0.016740817
TNNI2	0.284827555	2.35E-08	1.23E-07
TNNI3K	-0.102277195	0.049008806	0.076697049
TNNI3	0.263761521	2.54E-07	1.11E-06
TNNT1	0.381419961	2.71E-14	3.54E-13
TNNT2	0.315051787	5.41E-10	3.63E-09
TNNT3	-0.072940056	0.160899744	0.217141627
TNN	0.048390211	0.352650408	0.427367931
TNP1	0.083963298	0.106388207	0.151780711
TNPO1	0.168947541	0.001088123	0.002466726
TNPO2	0.121969094	0.018766169	0.03241651
TNPO3	-0.102012187	0.049601144	0.077491306
TNRC18	0.139812896	0.006993392	0.013350167
TNRC6A	-0.241646362	2.49E-06	9.17E-06
TNRC6B	-0.170817394	0.000955257	0.002188434
TNRC6C	-0.143462865	0.005634975	0.010959677
TNR	0.131100813	0.01148527	0.020871071
TNS1	0.03802067	0.465315246	0.540241874
TNS3	-0.267012465	1.78E-07	7.97E-07
TNS4	0.222572393	1.51E-05	4.83E-05
TNXB	0.063160786	0.224873969	0.290471322
TOB1	-0.238666701	3.34E-06	1.20E-05
TOB2	-0.163362106	0.001592956	0.003490801
TOE1	0.158728077	0.002166065	0.004628093
TOLLIP	-0.365172936	3.81E-13	4.17E-12
TOM1L1	-0.315535621	5.08E-10	3.43E-09
TOM1L2	0.004945399	0.924367045	0.940897214
TOM1	-0.145431832	0.005005201	0.009857994
TOMM20L	-0.135225411	0.009111964	0.016974804
TOMM20	0.04033657	0.438557889	0.514392127
TOMM22	0.020660669	0.691624071	0.748467312
TOMM34	0.288129436	1.59E-08	8.50E-08
TOMM40L	0.197006793	0.000133826	0.00036072
TOMM40	0.384167575	1.71E-14	2.30E-13
TOMM5	0.325562287	1.31E-10	9.71E-10
TOMM6	0.063737833	0.220658031	0.285878214
TOMM70A	-0.256917365	5.27E-07	2.17E-06
TOMM7	-0.130607718	0.011802745	0.021405034
TOP1MT	0.122314906	0.018430329	0.031900001
TOP1P1	-0.169997498	0.001011558	0.002306541
TOP1P2	0.134086946	0.009719051	0.017994696
TOP1	0.18076534	0.000467176	0.001136672
TOP2A	0.57184638	1.34E-33	4.23E-31
TOP2B	0.180327423	0.000482505	0.001171253

TOP3A	0.046394796	0.372880217	0.448771805
TOP3B	0.163280924	0.001601666	0.003506802
TOPBP1	0.348562998	4.88E-12	4.49E-11
TOPORS	-0.195009494	0.00015694	0.000417765
TOR1AIP1	-0.245739221	1.66E-06	6.26E-06
TOR1AIP2	-0.233341429	5.57E-06	1.94E-05
TOR1A	-0.035750249	0.492401509	0.566485101
TOR1B	0.032307418	0.535029445	0.606858153
TOR2A	0.22743796	9.68E-06	3.21E-05
TOR3A	0.261685956	3.17E-07	1.36E-06
TOX2	0.062333361	0.231018707	0.297328941
TOX3	-0.127522686	0.013970738	0.024854889
TOX4	-0.005969401	0.908768672	0.928387748
TOX	0.329691362	7.42E-11	5.74E-10
TP53AIP1	0.003279019	0.949809982	0.961001242
TP53BP1	0.195563875	0.000150174	0.000401203
TP53BP2	0.066117188	0.203870911	0.266905908
TP53I11	0.244879469	1.81E-06	6.80E-06
TP53I13	0.036595529	0.482219987	0.556366861
TP53I3	0.031906044	0.540117052	0.611805888
TP53INP1	0.129417559	0.012601154	0.022687772
TP53INP2	-0.107690994	0.038143585	0.061237529
TP53RK	0.10152139	0.050713959	0.079017949
TP53TG1	-0.09274083	0.074401988	0.110722075
TP53TG3B	0.102960597	0.047508513	0.074642318
TP53TG5	0.112997164	0.029547149	0.048775085
TP53	0.200882227	9.78E-05	0.000270748
TP63	-0.151503928	0.00344204	0.007028012
TP73	0.110866414	0.032775961	0.053517146
TPBG	0.282796073	2.99E-08	1.53E-07
TPCN1	0.253087805	7.85E-07	3.14E-06
TPCN2	-0.038750111	0.456791355	0.53189534
TPD52L1	-0.181189789	0.000452751	0.001104408
TPD52L2	0.427233363	6.82E-18	1.55E-16
TPD52L3	-0.008299751	0.873410636	0.900392584
TPD52	0.327346465	1.03E-10	7.73E-10
TPH1	0.107260931	0.038924848	0.062361143
TPH2	0.036321799	0.485504574	0.559605538
TPI1P2	-0.312760069	7.32E-10	4.82E-09
TPI1P3	0.07831207	0.132164759	0.183087847
TPI1	0.180299619	0.000483494	0.001173081
TPK1	-0.120341496	0.020419395	0.035002167
TPM1	0.171275853	0.000925049	0.002123871
TPM2	0.300194043	3.65E-09	2.16E-08
TPM3	0.541908768	1.06E-29	1.41E-27
TPM4	0.507178138	1.21E-25	8.69E-24
TPMT	-0.379386348	3.80E-14	4.87E-13
TPO	-0.047844088	0.358117108	0.433465522
TPP1	-0.083072134	0.110166552	0.156376063
TPP2	0.030510515	0.557991566	0.628466077
TPPP2	-0.405349571	4.19E-16	7.28E-15
TPPP3	0.229358461	8.10E-06	2.72E-05
TPPP	-0.099765255	0.054867709	0.084714756
TPRA1	-0.062386023	0.230624121	0.296840272
TPRG1L	-0.430749903	3.42E-18	8.12E-17
TPRG1	-0.543890382	6.00E-30	8.53E-28
TPRKB	0.268149382	1.57E-07	7.09E-07
TPRN	0.33764142	2.41E-11	1.99E-10

TPRX1	-0.007871177	0.879896588	0.905192917
TPRXL	0.176844113	0.000622147	0.001480814
TPR	0.224900114	1.22E-05	3.98E-05
TPSAB1	-0.078858742	0.129478145	0.179878913
TPSB2	-0.049655679	0.340186452	0.414405925
TPSD1	-0.022435478	0.666661939	0.727141481
TPSG1	0.095363451	0.066533106	0.100422193
TPST1	0.101264179	0.051305418	0.07981466
TPST2	-0.050489514	0.332129395	0.406166035
TPT1	-0.246044688	1.61E-06	6.10E-06
TPTE2P1	0.260226376	3.71E-07	1.57E-06
TPTE2P3	0.04668622	0.369881881	0.445732592
TPTE2	0.127281934	0.014153797	0.025149121
TPTE	-0.00508532	0.922233825	0.939157709
TPX2	0.556634186	1.43E-31	2.83E-29
TRA2A	0.212118243	3.81E-05	0.000113602
TRA2B	0.363359013	5.06E-13	5.44E-12
TRABD	0.238524279	3.38E-06	1.22E-05
TRADD	-0.08153628	0.116925146	0.164737112
TRAF1	0.198482893	0.00011884	0.000323699
TRAF2	0.463139796	4.01E-21	1.44E-19
TRAF3IP1	-0.01926625	0.711474687	0.765500039
TRAF3IP2	-0.089243607	0.086056071	0.125911358
TRAF3IP3	0.10234072	0.048867698	0.076536343
TRAF3	0.346376956	6.75E-12	6.12E-11
TRAF4	0.160994128	0.001865698	0.004039608
TRAF5	0.495137114	2.42E-24	1.40E-22
TRAF6	-0.095430155	0.066342235	0.100149277
TRAF7	0.064558209	0.214761956	0.279292884
TRAFD1	0.45741107	1.39E-20	4.63E-19
TRAIP	0.462795498	4.32E-21	1.55E-19
TRAK1	0.197936313	0.000124195	0.000336949
TRAK2	0.184303171	0.000358939	0.000892397
TRAM1L1	0.274449367	7.79E-08	3.69E-07
TRAM1	0.133197035	0.010218326	0.018814186
TRAM2	-0.042641538	0.412823513	0.488986862
TRANK1	-0.121215817	0.019516225	0.033584023
TRAP1	-0.071473112	0.169509604	0.227298265
TRAPPC10	0.089520669	0.085082206	0.124646295
TRAPPC1	0.156532823	0.002498524	0.005274515
TRAPPC2L	-0.024314459	0.640629961	0.704378281
TRAPPC2P1	0.093014976	0.073545535	0.109635899
TRAPPC2	0.084412886	0.104521269	0.149416814
TRAPPC3	0.013697287	0.79258879	0.834143444
TRAPPC4	0.360846594	7.50E-13	7.83E-12
TRAPPC5	0.033985419	0.514023874	0.586774215
TRAPPC6A	-0.157151607	0.002400395	0.005086764
TRAPPC6B	-0.206993602	5.89E-05	0.000169758
TRAPPC9	-0.036001824	0.489359305	0.563510008
TRAT1	0.139816091	0.006992085	0.013349231
TRDMT1	0.330249935	6.87E-11	5.34E-10
TRDN	0.157110688	0.002406774	0.005098113
TREH	-0.247974937	1.33E-06	5.10E-06
TREM1	0.400406862	1.02E-15	1.67E-14
TREM2	0.453297235	3.36E-20	1.05E-18
TREML1	0.274122687	8.08E-08	3.81E-07
TREML2P1	0.015098309	0.771931661	0.816307795
TREML2	0.157786383	0.002303393	0.00489474

TREML3	0.26292319	2.78E-07	1.20E-06
TREML4	0.208731036	5.09E-05	0.00014812
TRERF1	0.286972125	1.83E-08	9.67E-08
TREX1	-0.037674987	0.469385279	0.544248884
TREX2	0.069606082	0.180959465	0.2402422
TRHDE	0.149273277	0.00395579	0.0079716
TRHR	0.021389738	0.681327609	0.739654984
TRH	0.171669525	0.000899817	0.002071908
TRIAP1	-0.082980377	0.110561502	0.156880786
TRIB1	-0.051396947	0.323501914	0.397483973
TRIB2	-0.100745305	0.052516086	0.081504621
TRIB3	0.314313301	5.97E-10	3.98E-09
TRIL	-0.191526259	0.000206427	0.000537432
TRIM10	0.008521714	0.870054802	0.897815796
TRIM11	0.359447026	9.33E-13	9.58E-12
TRIM13	-0.030479853	0.558387489	0.628840902
TRIM14	0.021082423	0.685660676	0.743386774
TRIM15	0.150452203	0.003676215	0.007459519
TRIM16L	-0.005265936	0.919480994	0.937072865
TRIM16	0.14734501	0.004454727	0.008875416
TRIM17	0.387390193	9.88E-15	1.38E-13
TRIM21	0.189064696	0.000249826	0.000639392
TRIM22	-0.044880941	0.388695633	0.465038688
TRIM23	-0.171697341	0.000898058	0.002068098
TRIM24	-0.032372749	0.534203624	0.606094002
TRIM25	0.174863096	0.000717417	0.001686417
TRIM26	-0.10929423	0.035347109	0.057213609
TRIM27	-0.034253434	0.51070883	0.583491501
TRIM28	0.396919797	1.89E-15	2.97E-14
TRIM29	-0.108397598	0.036888795	0.059448195
TRIM2	-0.278984688	4.65E-08	2.29E-07
TRIM31	0.444340079	2.20E-19	6.17E-18
TRIM32	0.093497319	0.072058163	0.107692627
TRIM33	0.097541729	0.060528468	0.092359693
TRIM34	0.151639946	0.003412763	0.006973953
TRIM35	-0.235792346	4.41E-06	1.56E-05
TRIM36	0.230231489	7.47E-06	2.53E-05
TRIM37	0.097589763	0.060401273	0.092200917
TRIM38	-0.051457227	0.322933998	0.396810653
TRIM39	-0.000678437	0.989609024	0.99202353
TRIM3	-0.107755409	0.038027719	0.061071199
TRIM40	0.290212007	1.24E-08	6.77E-08
TRIM41	-0.105151516	0.042955625	0.068112223
TRIM42	0.004946622	0.924348402	0.940897214
TRIM43	0.005619913	0.914088713	0.932855845
TRIM44	-0.050081209	0.336059236	0.410049633
TRIM45	0.327313369	1.03E-10	7.76E-10
TRIM46	0.488049212	1.34E-23	6.94E-22
TRIM47	0.393382454	3.52E-15	5.30E-14
TRIM48	0.066666728	0.200128904	0.262611515
TRIM49L	0.088184613	0.089861345	0.130739628
TRIM49	0.129443914	0.012582971	0.022667325
TRIM4	-0.369713061	1.85E-13	2.13E-12
TRIM50	0.218880711	2.11E-05	6.55E-05
TRIM52	0.163722582	0.001554802	0.003415831
TRIM53	0.156011995	0.002583936	0.005440987
TRIM54	0.176949814	0.000617409	0.001469888
TRIM55	-0.117011413	0.024199857	0.040815235

TRIM56	-0.061745519	0.235455802	0.302161202
TRIM58	-0.025086659	0.630055724	0.69455346
TRIM59	0.558066494	9.32E-32	1.93E-29
TRIM5	0.03919097	0.451682347	0.527426043
TRIM6-TRIM	0.112108024	0.030859775	0.050674946
TRIM60	0.026369805	0.612651407	0.678408166
TRIM61	0.033056229	0.525602669	0.597663035
TRIM62	0.111442529	0.031874576	0.052203659
TRIM63	-0.059808035	0.250504179	0.318701338
TRIM65	0.290642313	1.18E-08	6.46E-08
TRIM66	-0.154388238	0.002867568	0.005960114
TRIM67	0.128188316	0.013475405	0.024085503
TRIM68	0.168624517	0.001112731	0.002518786
TRIM69	0.02817171	0.588575318	0.656215137
TRIM6	0.394659007	2.81E-15	4.31E-14
TRIM71	0.262342569	2.96E-07	1.27E-06
TRIM72	0.118034662	0.022978609	0.038989852
TRIM74	-0.011474666	0.825653795	0.861502902
TRIM77	-0.042096959	0.418821825	0.494826735
TRIM78P	0.051266331	0.324734713	0.39877735
TRIM7	0.109043991	0.035771764	0.057822646
TRIM8	-0.166686122	0.001271486	0.002845488
TRIM9	0.208998744	4.97E-05	0.000145216
TRIML1	0.031816347	0.541257302	0.612805636
TRIML2	0.098350618	0.058415772	0.089513038
TRIOBP	-0.001186031	0.981835741	0.985943426
TRIO	0.143268287	0.005700923	0.011076148
TRIP10	0.328107954	9.25E-11	7.01E-10
TRIP11	-0.180608064	0.000472628	0.001148537
TRIP12	0.021648539	0.677686685	0.736424706
TRIP13	0.622928279	2.97E-41	9.85E-38
TRIP4	0.253756035	7.33E-07	2.95E-06
TRIP6	0.129532237	0.012522202	0.022570099
TRIT1	0.054212684	0.297665646	0.369821952
TRMT112	0.113445522	0.028903582	0.04784124
TRMT11	0.102599211	0.048297012	0.075767625
TRMT12	0.12182963	0.018903111	0.032627602
TRMT1	0.273892982	8.29E-08	3.90E-07
TRMT2A	0.073723689	0.156437384	0.211937815
TRMT2B	0.102080677	0.049447487	0.077292494
TRMT5	-0.182463516	0.000411909	0.001011595
TRMT61A	0.082349733	0.113306203	0.16031862
TRMT61B	0.004420416	0.932375612	0.947499844
TRMT6	0.315126138	5.36E-10	3.60E-09
TRMU	0.153799588	0.002977191	0.006166222
TRNAU1AP	0.203936133	7.61E-05	0.000214595
TRNP1	0.412202723	1.19E-16	2.27E-15
TRNT1	0.256456828	5.53E-07	2.27E-06
TROAP	0.563144432	2.00E-32	4.62E-30
TROVE2	0.054579991	0.29439954	0.366289511
TRO	-0.277110249	5.76E-08	2.79E-07
TRPA1	0.225434373	1.16E-05	3.80E-05
TRPC1	0.374939356	7.91E-14	9.66E-13
TRPC2	0.118881772	0.022008281	0.037461464
TRPC3	0.250623511	1.01E-06	3.98E-06
TRPC4AP	0.200369851	0.000101972	0.000281136
TRPC4	0.111577329	0.031666752	0.051884615
TRPC5	-0.175909332	0.000665538	0.001575434

TRPC6	0.018886378	0.716917073	0.770274077
TRPC7	0.205305621	6.79E-05	0.00019332
TRPM1	-0.021091638	0.685530602	0.743326657
TRPM2	0.423270294	1.47E-17	3.20E-16
TRPM3	-0.275846287	6.65E-08	3.19E-07
TRPM4	0.106683815	0.039994485	0.063941107
TRPM5	0.155328121	0.002700124	0.005648776
TRPM6	-0.055122354	0.289620732	0.361155747
TRPM7	-0.276961613	5.86E-08	2.83E-07
TRPM8	-0.157443428	0.002355348	0.004999279
TRPS1	0.072585354	0.162950792	0.219567513
TRPT1	0.043188852	0.406846455	0.483171093
TRPV1	-0.265542424	2.09E-07	9.25E-07
TRPV2	0.264188754	2.42E-07	1.06E-06
TRPV3	-0.001066371	0.983668078	0.987534542
TRPV4	0.260595099	3.57E-07	1.51E-06
TRPV5	0.266658848	1.85E-07	8.26E-07
TRPV6	0.362084382	6.18E-13	6.54E-12
TRRAP	0.175370328	0.000691815	0.001631818
TRUB1	-0.117311324	0.023836238	0.040273559
TRUB2	-0.257756363	4.82E-07	2.00E-06
TRY6	0.133835498	0.00985788	0.01822802
TRYX3	0.143914973	0.005484382	0.010692882
TSC1	0.16904808	0.001080567	0.002451828
TSC22D1	-0.317827667	3.75E-10	2.58E-09
TSC22D2	-0.218090122	2.26E-05	6.99E-05
TSC22D3	-0.202239312	8.75E-05	0.000244246
TSC22D4	0.243711679	2.03E-06	7.57E-06
TSC2	-0.161194012	0.001841123	0.003990301
TSEN15	0.293715757	8.12E-09	4.56E-08
TSEN2	-0.018021586	0.729359994	0.780611894
TSEN34	0.239289016	3.14E-06	1.14E-05
TSEN54	0.372324277	1.21E-13	1.45E-12
TSFM	-0.093859679	0.070956989	0.106222207
TSG101	0.088097796	0.090179193	0.1311446
TSG1	-0.031235195	0.548673868	0.619899098
TSGA10IP	0.190359098	0.000226041	0.000583542
TSGA10	0.151294913	0.003487473	0.007110576
TSGA13	-0.00377853	0.94217672	0.955413041
TSGA14	0.211949397	3.86E-05	0.000115112
TSHB	-0.037088225	0.476338404	0.550857008
TSHR	-0.058844668	0.258230426	0.327464452
TSHZ1	-0.201820077	9.06E-05	0.000252029
TSHZ2	-0.227830523	9.34E-06	3.10E-05
TSHZ3	0.139932871	0.006944454	0.013272007
TSIX	0.122113164	0.01862561	0.032198838
TSKS	0.130110431	0.012130745	0.021935122
TSKU	-0.37120398	1.45E-13	1.71E-12
TSLP	-0.206325978	6.23E-05	0.000178802
TSNARE1	0.138353807	0.00761373	0.01441832
TSNAX-DIS	0.077075908	0.138398283	0.190609789
TSNAXIP1	-0.183096075	0.000392926	0.000969111
TSNAX	0.003502634	0.946392197	0.958565527
TSN	0.22470664	1.24E-05	4.04E-05
TSPAN10	0.108184625	0.037263253	0.059983721
TSPAN11	0.090307646	0.082364341	0.121141557
TSPAN12	0.007280215	0.888853179	0.912393499
TSPAN13	0.088156387	0.089964586	0.130870718

TSPAN14	0.184142711	0.000363293	0.00090221
TSPAN15	0.394518173	2.88E-15	4.41E-14
TSPAN16	0.08654242	0.096027789	0.138648037
TSPAN17	0.253020023	7.91E-07	3.16E-06
TSPAN18	-0.03574378	0.492479865	0.566531001
TSPAN19	0.108480867	0.036743258	0.05923282
TSPAN1	-0.008601721	0.868845749	0.896661055
TSPAN2	0.171778677	0.000892934	0.002057963
TSPAN31	-0.265097707	2.20E-07	9.67E-07
TSPAN32	0.105218162	0.042823097	0.067929113
TSPAN33	-0.21720122	2.44E-05	7.50E-05
TSPAN3	0.504549576	2.35E-25	1.65E-23
TSPAN4	0.077588801	0.135785098	0.187425395
TSPAN5	-0.050915176	0.328064111	0.402147011
TSPAN6	-0.127127526	0.014272311	0.025332589
TSPAN7	0.067229422	0.196349334	0.258127982
TSPAN8	0.190989676	0.000215238	0.000558183
TSPAN9	-0.364948727	3.94E-13	4.31E-12
TSPO2	0.294741321	7.16E-09	4.06E-08
TSPO	0.125626113	0.015470913	0.027244014
TSPY1	-0.017556957	0.736074911	0.786025106
TSPY2	0.016395839	0.752942642	0.800518909
TSPY3	0.026179796	0.615215275	0.680577163
TSPY4	0.026964425	0.604658504	0.670937213
TSPYL1	-0.281637184	3.42E-08	1.73E-07
TSPYL2	0.048811401	0.348470409	0.423048597
TSPYL3	0.123778305	0.017066465	0.029751323
TSPYL4	-0.063093005	0.225372923	0.290953056
TSPYL5	0.174710082	0.000725311	0.001703163
TSPYL6	-0.044313042	0.394731932	0.471058437
TSR1	0.081268506	0.118136016	0.166184559
TSR2	-0.092246888	0.075965578	0.11275919
TSSC1	0.447798352	1.07E-19	3.14E-18
TSSC4	0.211876728	3.89E-05	0.000115783
TSSK1B	-0.044372276	0.394099706	0.470416678
TSSK3	-0.125381136	0.015674635	0.027558916
TSSK4	0.120657461	0.0200889	0.034471237
TSSK6	-0.030003747	0.564552643	0.634600111
TSTA3	0.169702429	0.001032557	0.002351194
TSTD1	-0.119695488	0.021109837	0.036083241
TSTD2	-0.176696749	0.000628809	0.001495776
TST	-0.475241809	2.66E-22	1.14E-20
TTBK1	-0.235834411	4.39E-06	1.55E-05
TTBK2	-0.147440445	0.004428756	0.008827382
TTC12	-0.125612036	0.015482557	0.027258707
TTC13	0.456642266	1.65E-20	5.40E-19
TTC14	0.018014771	0.729458337	0.780675207
TTC15	-0.022091154	0.67147716	0.73147244
TTC16	0.070720425	0.174058717	0.232475044
TTC17	-0.054371319	0.296252104	0.36824948
TTC18	-0.350888648	3.45E-12	3.26E-11
TTC19	-0.103427693	0.046505365	0.073181665
TTC1	0.167492173	0.001203097	0.002704288
TTC21A	0.190219087	0.000228508	0.000589299
TTC21B	-0.043686463	0.401457061	0.477596296
TTC22	0.222851078	1.47E-05	4.72E-05
TTC23L	-0.017788765	0.732722231	0.783368696
TTC23	-0.008912153	0.864157464	0.893210802

TTC24	0.173523036	0.000789332	0.001840697
TTC25	0.056950686	0.273896111	0.344175497
TTC26	0.198130984	0.000122263	0.000332067
TTC27	0.171038036	0.000940607	0.002157105
TTC28	-0.164947623	0.001431316	0.003172528
TTC29	0.030185539	0.562194758	0.632484976
TTC30A	-0.237700171	3.67E-06	1.31E-05
TTC30B	-0.074251719	0.15348368	0.208460722
TTC31	-0.239183046	3.17E-06	1.15E-05
TTC32	-0.045116764	0.386205571	0.462490225
TTC33	-0.091555173	0.078199988	0.115768756
TTC35	0.107631683	0.038250536	0.061384499
TTC36	-0.554347972	2.83E-31	5.04E-29
TTC37	-0.044204854	0.395888253	0.472249458
TTC38	-0.568933119	3.34E-33	9.77E-31
TTC39A	0.375865693	6.80E-14	8.41E-13
TTC39B	-0.048752288	0.349055165	0.423681046
TTC39C	-0.127997705	0.013615645	0.024318376
TTC3	0.085676936	0.099410859	0.142983405
TTC4	0.069004448	0.184768263	0.244549119
TTC5	-0.036646302	0.481612078	0.555858631
TTC7A	0.081643679	0.116442228	0.164126367
TTC7B	-0.11471495	0.02714626	0.045240739
TTC8	-0.141350852	0.006388833	0.012300988
TTC9B	0.144330806	0.005349077	0.010456114
TTC9C	0.241712439	2.48E-06	9.11E-06
TTC9	-0.224622196	1.25E-05	4.07E-05
TTF1	0.22013243	1.88E-05	5.91E-05
TTF2	0.391537938	4.84E-15	7.16E-14
TTK	0.58320079	3.48E-35	1.82E-32
TTLL10	-0.133352588	0.010129448	0.018669521
TTLL11	-0.221553642	1.66E-05	5.25E-05
TTLL12	-0.009567325	0.85427858	0.885342448
TTLL13	-0.017725367	0.733638659	0.784095996
TTLL1	0.212181092	3.79E-05	0.000113022
TTLL2	0.001827912	0.972008544	0.977948296
TTLL3	0.300016597	3.73E-09	2.21E-08
TTLL4	0.408570866	2.33E-16	4.21E-15
TTLL5	0.185670032	0.000323768	0.000811334
TTLL6	0.205386606	6.74E-05	0.000192154
TTLL7	0.077482405	0.136324045	0.18807809
TTLL8	0.160860168	0.001882335	0.00407209
TTLL9	0.242637984	2.26E-06	8.36E-06
TTL	0.006535978	0.900152778	0.921632644
TTN	0.107132759	0.03916029	0.062713127
TTPAL	-0.222267342	1.55E-05	4.95E-05
TTPA	-0.389403758	7.00E-15	1.01E-13
TTR	-0.252650313	8.22E-07	3.27E-06
TTTY10	-0.12679827	0.014527941	0.025747321
TTTY14	-0.105713846	0.041848174	0.066577839
TTTY15	0.06834464	0.189012912	0.249537126
TTTY1B	0.085836382	0.098780588	0.14217953
TTTY20	0.092634974	0.074734855	0.111134862
TTTY2	0.148308926	0.004198623	0.008405686
TTTY4C	0.066563218	0.200829898	0.263453146
TTTY5	0.056396762	0.278597543	0.349224202
TTTY6B	0.0590931	0.256222427	0.325145754
TTTY6	-0.005037623	0.922960939	0.939754055

TTTY8	0.097463795	0.060735309	0.092597293
TTTY9B	-0.021712279	0.6767911	0.735611997
TTYH1	0.261504364	3.24E-07	1.38E-06
TTYH2	0.244865472	1.81E-06	6.80E-06
TTYH3	0.296410705	5.84E-09	3.35E-08
TUBA1A	0.346259493	6.87E-12	6.21E-11
TUBA1B	0.586417549	1.20E-35	7.49E-33
TUBA1C	0.575455149	4.26E-34	1.59E-31
TUBA3C	0.107014927	0.039377799	0.063015866
TUBA3D	-0.000672426	0.989701077	0.99202353
TUBA3E	0.079137438	0.128124853	0.178272337
TUBA4A	0.265148405	2.18E-07	9.63E-07
TUBA4B	0.055887789	0.282965402	0.353853288
TUBA8	0.113502952	0.028822022	0.047737972
TUBAL3	0.295351481	6.65E-09	3.79E-08
TUBB1	0.099273406	0.056080464	0.086393056
TUBB2A	0.178642792	0.000545938	0.001313073
TUBB2B	-0.008685056	0.86758672	0.895965095
TUBB2C	0.258792848	4.32E-07	1.81E-06
TUBB3	0.414672735	7.52E-17	1.48E-15
TUBB4Q	0.254236287	6.97E-07	2.82E-06
TUBB4	0.203948224	7.60E-05	0.000214471
TUBB6	0.246984531	1.46E-06	5.59E-06
TUBB8	0.252923706	7.99E-07	3.19E-06
TUBBP5	0.273018156	9.15E-08	4.28E-07
TUBB	0.295261434	6.72E-09	3.83E-08
TUBD1	0.163704125	0.001556735	0.003418947
TUBE1	-0.255028882	6.42E-07	2.61E-06
TUBG1	0.453165284	3.46E-20	1.07E-18
TUBG2	0.252873355	8.03E-07	3.20E-06
TUBGCP2	0.021246692	0.683343229	0.741398748
TUBGCP3	0.067763097	0.192813058	0.25399005
TUBGCP4	0.015867486	0.760657722	0.806912841
TUBGCP5	-0.223244167	1.42E-05	4.57E-05
TUBGCP6	-0.037602027	0.470246801	0.545046356
TUB	0.209462581	4.78E-05	0.000140055
TUFM	-0.137558122	0.007972322	0.015034514
TUFT1	0.310496539	9.83E-10	6.36E-09
TUG1	0.177612073	0.000588477	0.001407399
TULP1	0.176694947	0.00062889	0.001495792
TULP2	0.071332155	0.170354696	0.228277814
TULP3	0.240669846	2.74E-06	1.00E-05
TULP4	0.169516077	0.001046026	0.002378329
TUSC1	0.00294193	0.954963822	0.964892694
TUSC2	0.055558748	0.285813608	0.357034029
TUSC3	0.234231702	5.12E-06	1.79E-05
TUSC5	0.02592299	0.618687917	0.683648604
TUT1	-0.089725821	0.084366835	0.123712358
TWF1	0.187359674	0.000284731	0.000721772
TWF2	0.301582054	3.07E-09	1.84E-08
TWIST1	0.304608529	2.09E-09	1.29E-08
TWIST2	0.056622396	0.276675917	0.347099346
TWISTNB	-0.056679577	0.276190362	0.346621166
TWSG1	0.15319269	0.003094186	0.006387149
TXK	-0.153496161	0.003035172	0.006275083
TXLNA	0.105488155	0.042289722	0.067190035
TXLNB	0.244938374	1.80E-06	6.76E-06
TXLNG	0.027518765	0.597249308	0.664273311

TXN2	-0.200750393	9.89E-05	0.000273338
TXNDC11	-0.184260508	0.000360092	0.00089504
TXNDC12	0.140618601	0.006670576	0.012787839
TXNDC15	-0.00193626	0.970350116	0.976674329
TXNDC16	-0.090465827	0.081826605	0.120430676
TXNDC17	0.146931206	0.004568933	0.009080435
TXNDC2	0.052538347	0.312858431	0.385738685
TXNDC3	0.21833317	2.21E-05	6.85E-05
TXNDC5	-0.024409749	0.639321101	0.703249681
TXNDC6	0.046688787	0.369855539	0.445732592
TXNDC9	0.350587329	3.60E-12	3.40E-11
TXNIP	-0.154844034	0.002785222	0.005805314
TXNL1	0.003465034	0.946966815	0.959050018
TXNL4A	0.203560478	7.85E-05	0.00022094
TXNL4B	-0.173375544	0.000797642	0.001857901
TXNRD1	0.044127901	0.396711979	0.472968045
TXNRD2	-0.229631528	7.90E-06	2.66E-05
TXNRD3IT1	-0.429372685	4.48E-18	1.05E-16
TXN	0.095784167	0.065336747	0.098833612
TYK2	0.016835319	0.74654393	0.795243727
TYMP	0.137853498	0.007837484	0.014806861
TYMS	0.444933043	1.94E-19	5.50E-18
TYRO3	0.492115051	5.04E-24	2.78E-22
TYROBP	0.281896689	3.32E-08	1.68E-07
TYRP1	0.088333092	0.089319825	0.130018234
TYR	0.004146093	0.936563151	0.951076108
TYSND1	0.117332349	0.023810924	0.040234201
TYW1B	-0.163640676	0.001563397	0.003432443
TYW1	-0.276530895	6.15E-08	2.97E-07
TYW3	0.027232773	0.601066684	0.667769936
T	0.154842214	0.002785546	0.005805382
U2AF1L4	0.189680272	0.000238237	0.000612092
U2AF1	0.401526636	8.34E-16	1.39E-14
U2AF2	0.415817196	6.07E-17	1.21E-15
UACA	0.100990067	0.051942062	0.080704003
UAP1L1	0.471858789	5.74E-22	2.32E-20
UAP1	-0.183064618	0.00039385	0.000970954
UBA1	0.237975803	3.57E-06	1.28E-05
UBA2	0.393942547	3.19E-15	4.85E-14
UBA3	0.139670092	0.007052044	0.013449246
UBA52	0.125355507	0.015696083	0.027589322
UBA5	-0.047450879	0.362085834	0.437578039
UBA6	0.010666055	0.837763324	0.871536341
UBA7	-0.016232668	0.755322709	0.802291486
UBAC1	0.167775922	0.00117984	0.002658009
UBAC2	0.133694783	0.009936338	0.018352653
UBAP1	-0.025817037	0.620123138	0.685033051
UBAP2L	0.304816909	2.04E-09	1.26E-08
UBAP2	0.358100278	1.15E-12	1.17E-11
UBASH3A	0.217422288	2.40E-05	7.37E-05
UBASH3B	0.365305506	3.73E-13	4.09E-12
UBB	-0.226217176	1.08E-05	3.56E-05
UBC	-0.113032742	0.029495635	0.048715971
UBD	0.40282892	6.60E-16	1.12E-14
UBE2A	0.228878389	8.47E-06	2.84E-05
UBE2B	0.149459903	0.003910289	0.007890284
UBE2CBP	0.216674394	2.56E-05	7.84E-05
UBE2C	0.615254364	5.15E-40	8.56E-37

UBE2D1	0.409393898	2.00E-16	3.67E-15
UBE2D2	0.430000481	3.96E-18	9.33E-17
UBE2D3	-0.275522303	6.90E-08	3.30E-07
UBE2D4	-0.304268172	2.19E-09	1.34E-08
UBE2DNL	0.00250083	0.961710731	0.969889702
UBE2E1	0.434221568	1.72E-18	4.28E-17
UBE2E2	0.138005869	0.007768725	0.014687473
UBE2E3	-0.000309256	0.995263308	0.996442872
UBE2F	0.08772224	0.091564553	0.132994242
UBE2G1	-0.030546584	0.557526002	0.628083749
UBE2G2	-0.056648329	0.276455633	0.346885579
UBE2H	-0.224611752	1.26E-05	4.07E-05
UBE2I	0.455467317	2.12E-20	6.80E-19
UBE2J1	0.21727305	2.43E-05	7.46E-05
UBE2J2	0.19288863	0.000185548	0.000487213
UBE2K	0.193963544	0.000170488	0.000450813
UBE2L3	0.314545225	5.79E-10	3.87E-09
UBE2L6	0.101202857	0.051447275	0.080016599
UBE2MP1	0.346290493	6.84E-12	6.18E-11
UBE2M	0.32785586	9.58E-11	7.24E-10
UBE2NL	0.151501427	0.00344258	0.007028396
UBE2N	0.434821082	1.52E-18	3.86E-17
UBE2O	0.114032283	0.028079537	0.046612828
UBE2Q1	0.090626357	0.081283792	0.11972022
UBE2Q2P1	-0.079848353	0.124722391	0.174207353
UBE2Q2	0.313375119	6.75E-10	4.46E-09
UBE2QL1	-0.268444493	1.52E-07	6.88E-07
UBE2R2	0.113320614	0.029081654	0.048084049
UBE2S	0.50856985	8.48E-26	6.28E-24
UBE2T	0.561230596	3.58E-32	7.92E-30
UBE2U	0.182145238	0.00042178	0.00103329
UBE2V1	0.227624614	9.52E-06	3.16E-05
UBE2V2	0.16497921	0.001428255	0.003166799
UBE2W	0.038789235	0.456336644	0.531552279
UBE2Z	0.365742334	3.48E-13	3.85E-12
UBE3A	-0.196085592	0.000144058	0.000386107
UBE3B	-0.004699359	0.928119472	0.943945075
UBE3C	-0.151779552	0.003382949	0.006917995
UBE4A	-0.207523496	5.63E-05	0.00016289
UBE4B	-0.132251853	0.010773387	0.019719925
UBFD1	-0.013212721	0.799768084	0.839726983
UBIAD1	-0.144181084	0.005397444	0.010537817
UBL3	-0.168474001	0.001124372	0.002542249
UBL4A	0.13957403	0.007091745	0.013518491
UBL4B	0.027637122	0.595672719	0.662812328
UBL5	0.097144007	0.061590159	0.093757099
UBL7	0.070605867	0.174758973	0.23323831
UBLCP1	0.251605399	9.15E-07	3.62E-06
UBN1	0.03826448	0.462456448	0.537329836
UBN2	-0.064960755	0.211910564	0.275963019
UBOX5	0.021135535	0.684911071	0.742776176
UBP1	0.006023215	0.907949856	0.927741552
UBQLN1	-0.083721989	0.107401056	0.152979335
UBQLN2	-0.002083368	0.968098601	0.974851412
UBQLN3	0.070640877	0.174544746	0.233030454
UBQLN4	0.045031003	0.38711	0.463406166
UBQLNL	-0.043373288	0.404843913	0.481050962
UBR1	-0.130634867	0.011785066	0.021378809

UBR2	-0.098460316	0.058134014	0.089136137
UBR3	-0.29872558	4.38E-09	2.56E-08
UBR4	-0.039541574	0.447642363	0.523383483
UBR5	0.288815142	1.47E-08	7.90E-08
UBR7	0.117810876	0.023241044	0.03939503
UBTD1	0.021155626	0.684627593	0.742549592
UBTD2	0.325821685	1.27E-10	9.39E-10
UBTFL1	0.102701995	0.048071644	0.075449719
UBTF	0.044164808	0.396316786	0.472553448
UBXN10	-0.38689418	1.08E-14	1.49E-13
UBXN11	0.195972488	0.000145364	0.000389187
UBXN1	0.132286773	0.010752415	0.019690192
UBXN2A	0.266962238	1.79E-07	8.01E-07
UBXN2B	-0.07583104	0.14490124	0.198223065
UBXN4	0.002086541	0.968050047	0.974851412
UBXN6	-0.341501054	1.38E-11	1.19E-10
UBXN7	-0.002702821	0.958620786	0.967508146
UBXN8	-0.159646202	0.002039411	0.004384283
UCA1	0.302326078	2.80E-09	1.69E-08
UCHL1	0.368134689	2.38E-13	2.71E-12
UCHL3	0.059416814	0.253622176	0.32227714
UCHL5	0.125226508	0.01580443	0.027767514
UCK1	0.021604706	0.678302828	0.736973655
UCK2	0.427513479	6.45E-18	1.47E-16
UCKL1AS	0.200024839	0.000104878	0.000288349
UCKL1	0.159870136	0.002009565	0.004322918
UCMA	0.032671836	0.530431201	0.602397126
UCN2	0.090872896	0.080455819	0.118671686
UCN3	0.1203588	0.020401173	0.034973942
UCN	-0.06222809	0.231808927	0.298211123
UCP1	0.193762188	0.000173219	0.000457195
UCP2	0.287209858	1.78E-08	9.41E-08
UCP3	0.182647359	0.000406306	0.000999312
UEVLD	0.039982669	0.442588848	0.518235278
UFC1	0.215411376	2.86E-05	8.69E-05
UFD1L	0.120406376	0.020351148	0.034897192
UFM1	-0.176246035	0.000649596	0.001542093
UFSP1	-0.251986138	8.80E-07	3.49E-06
UFSP2	-0.330556331	6.58E-11	5.12E-10
UGCG	0.08647018	0.096306612	0.139030458
UGDH	0.114248121	0.02778153	0.046164288
UGGT1	-0.073688918	0.156633383	0.212145643
UGGT2	0.20340712	7.95E-05	0.000223351
UGP2	-0.316268559	4.61E-10	3.13E-09
UGT1A10	0.241229809	2.60E-06	9.53E-06
UGT1A1	-0.371852299	1.31E-13	1.55E-12
UGT1A3	-0.436785101	1.03E-18	2.64E-17
UGT1A4	-0.478779235	1.18E-22	5.38E-21
UGT1A5	-0.404729152	4.69E-16	8.09E-15
UGT1A6	-0.146014347	0.004831438	0.009557333
UGT1A7	0.129347801	0.012649394	0.022758172
UGT1A8	-0.003105348	0.952465029	0.963100615
UGT1A9	-0.256134927	5.72E-07	2.34E-06
UGT2A1	0.103782133	0.045756064	0.07211648
UGT2A3	0.084197678	0.105411672	0.150560033
UGT2B10	-0.480968898	7.10E-23	3.32E-21
UGT2B11	-0.038265781	0.462441214	0.537329836
UGT2B15	-0.342677978	1.16E-11	1.01E-10

UGT2B28	-0.23548914	4.54E-06	1.60E-05
UGT2B4	-0.196478344	0.000139609	0.00037509
UGT2B7	-0.378781958	4.20E-14	5.35E-13
UGT3A1	-0.043523345	0.403218996	0.479406113
UGT3A2	0.126435805	0.014813995	0.02618208
UGT8	0.237650823	3.68E-06	1.31E-05
UHMK1	-0.116577545	0.02473436	0.041628616
UHRF1BP1L	-0.061422402	0.237920154	0.305009351
UHRF1BP1	0.383273211	1.98E-14	2.64E-13
UHRF1	0.603399928	3.65E-38	3.82E-35
UHRF2	0.226102484	1.10E-05	3.59E-05
UIMC1	0.398159126	1.52E-15	2.42E-14
ULBP1	0.312930223	7.16E-10	4.72E-09
ULBP2	0.213567797	3.36E-05	0.000101044
ULBP3	0.222599337	1.51E-05	4.82E-05
ULK1	0.088563001	0.088486486	0.128984252
ULK2	0.090793766	0.080720821	0.118987806
ULK3	0.122115725	0.018623121	0.032197328
ULK4	-0.112133388	0.030821651	0.050624861
UMODL1	0.361309876	6.98E-13	7.32E-12
UMOD	-0.033287933	0.522702982	0.594841129
UMPS	0.115243811	0.026441698	0.044195948
UNC119B	0.262418637	2.93E-07	1.26E-06
UNC119	0.45466322	2.51E-20	8.01E-19
UNC13A	0.369817255	1.81E-13	2.10E-12
UNC13B	-0.125844316	0.015291415	0.026944593
UNC13C	0.092458358	0.075292924	0.111880781
UNC13D	0.320024696	2.79E-10	1.96E-09
UNC45A	0.235729996	4.43E-06	1.56E-05
UNC45B	-0.0076073	0.883894074	0.908285849
UNC50	-0.00621985	0.90495874	0.92534969
UNC5A	0.192092016	0.000197503	0.000515546
UNC5B	0.346361227	6.77E-12	6.13E-11
UNC5CL	0.231983159	6.33E-06	2.17E-05
UNC5C	0.081330351	0.117855485	0.165871916
UNC5D	0.054692964	0.29339981	0.365228132
UNC80	0.020588093	0.692652194	0.749376398
UNC93A	0.078908349	0.129236462	0.179618305
UNC93B1	0.261970936	3.08E-07	1.32E-06
UNG	0.217050063	2.48E-05	7.60E-05
UNKL	0.00452593	0.930765439	0.946249698
UNK	0.187022335	0.000292155	0.000738244
UOX	0.024872657	0.632978763	0.697197043
UPB1	-0.472317829	5.18E-22	2.11E-20
UPF0639	0.163753771	0.00155154	0.003409794
UPF1	0.018976263	0.715628006	0.769013492
UPF2	-0.007217355	0.889806728	0.913137055
UPF3A	0.100780796	0.052432526	0.081396462
UPF3B	0.332068343	5.32E-11	4.19E-10
UPK1A	0.164035683	0.001522344	0.003352286
UPK1B	0.204200529	7.44E-05	0.000210475
UPK2	0.014610877	0.779101088	0.8224487
UPK3A	0.392558828	4.06E-15	6.07E-14
UPK3BL	0.007849216	0.880229159	0.905360386
UPK3B	-0.035207459	0.498999513	0.572873181
UPP1	0.326216443	1.20E-10	8.93E-10
UPP2	-0.13212166	0.010851899	0.019844726
UPRT	-0.029541737	0.570566274	0.640059762

UQCC	-0.081676494	0.116294984	0.163942023
UQCR10	-0.010782849	0.836011817	0.870181477
UQCR11	-0.07192784	0.16680467	0.224078397
UQCRB	0.154469656	0.002852698	0.00593478
UQCRC1	-0.156935753	0.00243422	0.005152414
UQCRC2	-0.178204736	0.000563656	0.001352421
UQCRFS1	0.043221404	0.406492589	0.482779621
UQCRHL	0.321312911	2.35E-10	1.67E-09
UQCRH	0.328561283	8.69E-11	6.62E-10
UQCRQ	-0.112322326	0.030538927	0.050230887
URB1	-0.038755628	0.45672721	0.531882856
URB2	0.193042484	0.000183319	0.000481931
URGCP	-0.119684934	0.021121283	0.036099709
URM1	0.175921427	0.000664959	0.001574508
UROC1	-0.200440117	0.00010139	0.000279724
UROD	-0.071233314	0.17094916	0.228920425
UROS	-0.10690577	0.039580206	0.063324516
USE1	-0.034761731	0.504452456	0.577769766
USF1	0.192947287	0.000184695	0.000485165
USF2	0.045381726	0.383419423	0.459678747
USH1C	0.167078568	0.001237755	0.002775613
USH1G	0.260376434	3.65E-07	1.54E-06
USH2A	-0.244079025	1.96E-06	7.32E-06
USHBP1	-0.193114616	0.000182282	0.000479459
USMG5	0.06602306	0.204516916	0.267629138
USO1	-0.155513098	0.002668237	0.005596023
USP10	-0.091015801	0.07997902	0.118095319
USP11	0.204487673	7.27E-05	0.000205746
USP12	-0.163789801	0.00154778	0.003402657
USP13	0.006231539	0.904780976	0.925215409
USP14	0.237919038	3.59E-06	1.28E-05
USP15	-0.017259489	0.740384584	0.789949748
USP16	-0.004045655	0.93809678	0.95219667
USP17L2	0.019797373	0.703889885	0.759130185
USP17	0.026093025	0.616387674	0.681711832
USP18	-0.052129433	0.31664482	0.389828086
USP19	-0.105571808	0.042125606	0.066961327
USP1	0.300183333	3.66E-09	2.16E-08
USP20	-0.163090811	0.001622235	0.00354677
USP21	0.27951618	4.37E-08	2.17E-07
USP22	0.133709666	0.009928013	0.018338978
USP24	0.147329979	0.00445883	0.008880217
USP25	-0.131907597	0.010982089	0.020053636
USP26	0.100546921	0.052985211	0.082145633
USP27X	0.198652197	0.000117226	0.000319651
USP28	0.005191912	0.9206091	0.937934667
USP29	0.034676521	0.505498438	0.57863364
USP2	-0.143579533	0.005595763	0.010892983
USP30	-0.266618915	1.86E-07	8.29E-07
USP31	-0.097592477	0.060394093	0.092197021
USP32	0.027050295	0.603508102	0.669959234
USP33	0.127812198	0.013753362	0.024529497
USP34	0.087654806	0.091815099	0.133319271
USP35	-0.016140553	0.756667352	0.803449898
USP36	0.155086614	0.002742278	0.00572659
USP37	0.279222068	4.52E-08	2.23E-07
USP38	-0.313252975	6.86E-10	4.53E-09
USP39	0.396945365	1.88E-15	2.96E-14

USP3	0.187302217	0.000285983	0.000724393
USP40	-0.139521617	0.007113491	0.013558646
USP42	0.064297593	0.216622625	0.281363668
USP43	0.309939632	1.06E-09	6.80E-09
USP44	0.111316654	0.032069692	0.052488695
USP45	0.040530683	0.436355889	0.512563799
USP46	0.041790378	0.422221047	0.498369931
USP47	-0.250124563	1.06E-06	4.18E-06
USP48	0.163477372	0.001580663	0.003465387
USP49	0.02076116	0.690201427	0.747130669
USP4	0.077673057	0.13535947	0.186902645
USP50	-0.011416687	0.826520739	0.862141351
USP51	0.158711061	0.002168478	0.00463176
USP53	-0.237438018	3.76E-06	1.34E-05
USP54	-0.020952236	0.687499401	0.744934356
USP5	0.01334244	0.797844493	0.838458511
USP6NL	-0.026629653	0.609152796	0.675021804
USP6	0.067395092	0.195246537	0.256880142
USP7	-0.152714967	0.003189199	0.0065622
USP8	-0.175184445	0.000701097	0.001651953
USP9X	-0.022579941	0.664645739	0.725617734
USP9Y	0.023698823	0.649112763	0.712015442
USPL1	-0.013268415	0.798942051	0.83916927
UST	-0.149687498	0.003855441	0.007789077
UTF1	0.187794884	0.000275412	0.000700111
UTP11L	0.22576072	1.13E-05	3.70E-05
UTP14A	0.136761834	0.008346191	0.015659523
UTP14C	-0.243273953	2.12E-06	7.88E-06
UTP15	0.140302907	0.006795438	0.013003431
UTP18	0.323003538	1.86E-10	1.35E-09
UTP20	0.231149574	6.85E-06	2.33E-05
UTP23	0.248685557	1.23E-06	4.78E-06
UTP3	-0.077041371	0.138575624	0.190814458
UTP6	0.342787449	1.14E-11	9.99E-11
UTRN	0.273297483	8.87E-08	4.15E-07
UTS2D	-0.047244882	0.364175907	0.439810404
UTS2R	0.146429172	0.004711031	0.009338623
UTS2	0.289105123	1.42E-08	7.65E-08
UTY	-0.082004489	0.114831263	0.162188474
UVRAG	0.037182792	0.475214004	0.549939784
UXS1	0.367831064	2.49E-13	2.83E-12
UXT	0.069521934	0.181488665	0.240880084
VAC14	-0.03039772	0.559448713	0.629857997
VAMP1	0.128527514	0.013228984	0.023682552
VAMP2	-0.225421005	1.17E-05	3.81E-05
VAMP3	-0.162838503	0.001649907	0.003603315
VAMP4	-0.049754087	0.339229126	0.413391402
VAMP5	-0.19145388	0.000207595	0.000540404
VAMP7	-0.207277243	5.75E-05	0.000166016
VAMP8	0.265081728	2.20E-07	9.69E-07
VANGL1	0.316244725	4.62E-10	3.14E-09
VANGL2	0.346184003	6.95E-12	6.26E-11
VAPA	0.182273371	0.00041778	0.001024498
VAPB	0.156694275	0.002472574	0.005224715
VARS2	-0.073407487	0.158226598	0.214085187
VARS	0.135618321	0.008910409	0.016624196
VASH1	0.304717875	2.07E-09	1.27E-08
VASH2	0.180683177	0.000470017	0.001143167

VASN	-0.037110883	0.476068873	0.550641204
VASP	0.474033011	3.51E-22	1.46E-20
VAT1L	0.121655287	0.019075526	0.032896696
VAT1	0.282021371	3.27E-08	1.66E-07
VAV1	0.244226735	1.93E-06	7.22E-06
VAV2	-0.092956585	0.073727278	0.109873941
VAV3	0.197372554	0.000129956	0.000351095
VAX1	0.044204704	0.39588986	0.472249458
VAX2	0.286862342	1.85E-08	9.78E-08
VBP1	0.223750951	1.36E-05	4.38E-05
VCAM1	0.255115331	6.36E-07	2.59E-06
VCAN	0.315176137	5.33E-10	3.58E-09
VCL	0.250766488	9.97E-07	3.93E-06
VCPIP1	-0.040617389	0.435374346	0.511531477
VCP	0.057611811	0.268355862	0.338173539
VCX2	0.131077464	0.011500131	0.02089236
VCX3A	0.080867543	0.119967452	0.168439748
VCX3B	-0.009059648	0.861931596	0.891557697
VCX	0.092201542	0.076110451	0.112952354
VCY	0.018782562	0.718406953	0.771583553
VDAC1	0.575416256	4.31E-34	1.59E-31
VDAC2	0.284472527	2.45E-08	1.27E-07
VDAC3	0.089120719	0.086490881	0.126454669
VDR	0.326653185	1.13E-10	8.45E-10
VEGFA	0.116148695	0.025272659	0.042423484
VEGFB	0.390562347	5.73E-15	8.35E-14
VEGFC	0.060693205	0.243548096	0.311182989
VENTXP1	0.099730521	0.054952633	0.084832727
VENTXP7	0.089935641	0.083640212	0.122836734
VENTX	0.13006835	0.012158866	0.021978852
VEPH1	0.305534011	1.86E-09	1.15E-08
VEZF1	0.163773332	0.001549498	0.003406057
VEZT	0.328795692	8.41E-11	6.42E-10
VGF	0.229978423	7.65E-06	2.58E-05
VGLL1	0.117538467	0.023563994	0.039871062
VGLL2	0.025308313	0.627034247	0.691860291
VGLL3	0.059957426	0.249320606	0.317418527
VGLL4	0.248672809	1.23E-06	4.78E-06
VHLL	0.055834144	0.283428442	0.354343359
VHL	0.206053483	6.38E-05	0.000182645
VIL1	0.189843595	0.000235248	0.000605036
VILL	0.245951306	1.62E-06	6.15E-06
VIM	0.227412986	9.71E-06	3.21E-05
VIPAR	-0.112252715	0.030642833	0.05037268
VIPR1	-0.376020461	6.63E-14	8.22E-13
VIPR2	0.165248675	0.001402381	0.003114283
VIP	0.031439523	0.546060583	0.617226606
VIT	0.163710364	0.001556081	0.003417888
VKORC1L1	-0.127964211	0.013640421	0.024354227
VKORC1	-0.252944428	7.97E-07	3.18E-06
VLDLR	0.065054197	0.211252593	0.275214109
VMA21	0.19317091	0.000181477	0.000477467
VMAC	-0.030771601	0.554625794	0.625559365
VMO1	0.065461421	0.208402307	0.271981052
VN1R1	0.156308039	0.002535066	0.005346559
VN1R2	0.002450562	0.962479801	0.970370577
VN1R5	0.176372348	0.000643707	0.001529207
VNN1	0.175412137	0.000689743	0.001627702

VNN2	0.361406197	6.88E-13	7.23E-12
VNN3	0.161923754	0.001753897	0.003813703
VOPP1	0.319820072	2.87E-10	2.01E-09
VPRBP	-0.094406983	0.06931988	0.104147108
VPREB1	0.014330661	0.783231188	0.826326947
VPREB3	0.054528238	0.294858274	0.36676864
VPS11	0.149847287	0.00381735	0.007716036
VPS13A	0.022531062	0.665327655	0.726113644
VPS13B	0.062903119	0.226774893	0.292414849
VPS13C	0.03505489	0.500862495	0.574417722
VPS13D	-0.192866918	0.000185865	0.000487852
VPS16	0.12266177	0.018098744	0.031369686
VPS18	0.090040258	0.083279804	0.122343504
VPS24	0.403154863	6.23E-16	1.06E-14
VPS25	0.130707341	0.011737986	0.021303102
VPS26A	0.163596017	0.001568101	0.003442014
VPS26B	-0.230940593	6.99E-06	2.38E-05
VPS28	0.069494788	0.18165963	0.24102085
VPS29	0.357575047	1.25E-12	1.26E-11
VPS33A	0.087036058	0.094139709	0.136247822
VPS33B	0.134507137	0.009490926	0.017606688
VPS35	0.07960849	0.125862452	0.175602757
VPS36	-0.290765854	1.16E-08	6.36E-08
VPS37A	0.11316797	0.029300543	0.048413819
VPS37B	0.134362958	0.00956866	0.017742632
VPS37C	0.500428576	6.58E-25	4.20E-23
VPS37D	-0.291389167	1.08E-08	5.91E-08
VPS39	-0.00475705	0.927239463	0.943240095
VPS41	-0.145817789	0.004889453	0.00965867
VPS45	0.322753895	1.93E-10	1.39E-09
VPS4A	-0.040756052	0.433807289	0.509900809
VPS4B	-0.151999509	0.003336454	0.006832031
VPS52	-0.109806224	0.034491593	0.055983502
VPS53	0.042168672	0.41802903	0.494036563
VPS54	0.06937848	0.18239347	0.241775907
VPS72	0.323285576	1.79E-10	1.30E-09
VPS8	0.226163492	1.09E-05	3.57E-05
VRK1	0.502119499	4.32E-25	2.88E-23
VRK2	0.31479021	5.60E-10	3.75E-09
VRK3	-0.248719427	1.23E-06	4.76E-06
VSIG10L	0.211564203	3.99E-05	0.000118744
VSIG10	0.239375204	3.11E-06	1.13E-05
VSIG1	0.378495388	4.41E-14	5.59E-13
VSIG2	-0.150752069	0.003608019	0.007336833
VSIG4	0.135652497	0.008893066	0.016593394
VSIG8	0.191757453	0.000202735	0.000528513
VSNL1	-0.136635192	0.00840707	0.015760388
VSTM1	0.115625724	0.02594274	0.043445802
VSTM2A	0.057550114	0.268869613	0.338756668
VSTM2B	0.105173964	0.042910947	0.068052212
VSTM2L	-0.071133179	0.171552984	0.229620972
VSX1	0.232256095	6.17E-06	2.12E-05
VSX2	0.134740277	0.009366414	0.017400009
VTA1	0.177639482	0.000587307	0.001405276
VTCN1	0.304435521	2.14E-09	1.31E-08
VTI1A	0.071912435	0.166895774	0.224185665
VTI1B	-0.152229111	0.003288539	0.006740845
VTN	-0.167162912	0.001230614	0.002761154

VWA1	-0.014121805	0.786313505	0.828867475
VWA2	0.15550053	0.002670393	0.005598187
VWA3A	0.025503288	0.624381575	0.68927789
VWA3B	0.184804119	0.000345655	0.000862168
VWA5A	0.068599335	0.187366013	0.247641857
VWA5B1	-0.027625188	0.595831602	0.66295209
VWA5B2	0.387675326	9.41E-15	1.32E-13
VWC2L	0.02381831	0.647462764	0.710596804
VWC2	0.03467109	0.505565156	0.578676762
VWCE	-0.065560703	0.207711627	0.271168481
VWDE	0.284957938	2.32E-08	1.21E-07
VWF	-0.372232092	1.23E-13	1.47E-12
WAC	0.144614738	0.005258422	0.010298692
WAPAL	0.044586845	0.391814618	0.468154286
WARS2	0.130029624	0.012184796	0.022021728
WARS	0.216349615	2.63E-05	8.06E-05
WASF1	0.349677452	4.13E-12	3.87E-11
WASF2	0.322619848	1.96E-10	1.41E-09
WASF3	0.000341803	0.994764811	0.996064765
WASH2P	0.155082203	0.002743054	0.005727609
WASH3P	0.232874033	5.82E-06	2.01E-05
WASH5P	0.156052516	0.002577197	0.005429092
WASH7P	0.220470017	1.83E-05	5.75E-05
WASL	-0.184714042	0.000348009	0.000867606
WAS	0.192881757	0.000185648	0.000487412
WBP11P1	-0.039763192	0.445099275	0.52077687
WBP11	0.075343999	0.147507971	0.201388007
WBP1	-0.150605045	0.003641311	0.007397736
WBP2NL	-0.096592081	0.063088883	0.095833528
WBP2	-0.219890148	1.92E-05	6.03E-05
WBP4	-0.131912532	0.010979072	0.020049965
WBP5	0.395309009	2.51E-15	3.87E-14
WBSCR16	-0.079283855	0.127418284	0.177413129
WBSCR17	0.157210246	0.002391281	0.005067988
WBSCR22	-0.00023206	0.996445672	0.997146408
WBSCR26	0.203730814	7.74E-05	0.000218031
WBSCR27	0.011966058	0.818314775	0.855458202
WBSCR28	0.207030886	5.87E-05	0.000169275
WDFY1	0.049303362	0.343627939	0.417963225
WDFY2	0.165667057	0.00136306	0.003032371
WDFY3	-0.035708539	0.492906865	0.566891269
WDFY4	0.264413568	2.37E-07	1.04E-06
WDHD1	0.474574736	3.10E-22	1.31E-20
WDR11	-0.120847922	0.019891935	0.034150907
WDR12	0.152706246	0.003190958	0.006564454
WDR13	0.151624897	0.003415991	0.006979833
WDR16	0.171752702	0.000894568	0.002061489
WDR17	0.036157236	0.48748501	0.561595812
WDR18	0.050133261	0.335556598	0.409542274
WDR19	-0.165737781	0.001356514	0.003018483
WDR1	0.284508347	2.44E-08	1.27E-07
WDR20	-0.091787468	0.07744375	0.114751534
WDR24	-0.309355157	1.14E-09	7.29E-09
WDR25	-0.175580896	0.000681437	0.001609982
WDR26	0.161759571	0.001773185	0.003851859
WDR27	0.207693962	5.55E-05	0.000160701
WDR31	-0.153433478	0.003047277	0.006296189
WDR33	0.078890018	0.129325728	0.17971596

WDR34	0.323773214	1.68E-10	1.22E-09
WDR35	-0.08575989	0.099082554	0.142552368
WDR36	0.038553646	0.459078508	0.534121476
WDR37	-0.043777055	0.400480521	0.476662262
WDR38	0.196054147	0.00014442	0.00038692
WDR3	-0.015010449	0.773222535	0.817369168
WDR41	0.259943191	3.82E-07	1.61E-06
WDR43	0.112258675	0.030633925	0.050362194
WDR44	-0.094774972	0.068236609	0.102783134
WDR45L	0.214792301	3.02E-05	9.15E-05
WDR45	-0.139424916	0.007153769	0.013627594
WDR46	0.087411461	0.092723794	0.134442753
WDR47	0.127338193	0.014110832	0.025081728
WDR48	-0.091919066	0.077017969	0.114164199
WDR49	0.154303716	0.00288308	0.005989229
WDR4	0.424064912	1.26E-17	2.76E-16
WDR52	0.090045295	0.083262485	0.122327081
WDR53	0.260566184	3.58E-07	1.52E-06
WDR54	0.311500447	8.63E-10	5.64E-09
WDR55	0.226782304	1.03E-05	3.39E-05
WDR59	-0.408573053	2.33E-16	4.21E-15
WDR5B	-0.162142245	0.001728527	0.003760946
WDR5	0.081768345	0.115883625	0.163419947
WDR60	-0.025506606	0.62433648	0.68927789
WDR61	0.013539485	0.794924887	0.835963556
WDR62	0.58430934	2.41E-35	1.38E-32
WDR63	0.032431658	0.533459527	0.605353195
WDR64	-0.012281227	0.81361598	0.85121613
WDR65	-0.443349666	2.70E-19	7.50E-18
WDR66	0.151825523	0.003373183	0.006900149
WDR67	0.434794466	1.53E-18	3.87E-17
WDR69	0.259050907	4.20E-07	1.76E-06
WDR6	0.243319219	2.11E-06	7.84E-06
WDR70	0.069186366	0.183610404	0.243242882
WDR72	0.104599785	0.044066079	0.069712096
WDR73	0.038750221	0.456790068	0.53189534
WDR74	0.008287883	0.873590129	0.900454941
WDR75	0.250539935	1.02E-06	4.01E-06
WDR76	0.424745229	1.11E-17	2.44E-16
WDR77	0.266176564	1.95E-07	8.67E-07
WDR78	-0.20874004	5.08E-05	0.000148051
WDR7	-0.338525539	2.12E-11	1.77E-10
WDR81	-0.248368003	1.27E-06	4.92E-06
WDR82	0.107981581	0.03762325	0.060485022
WDR83	0.055279222	0.288248312	0.359714538
WDR85	0.291794889	1.03E-08	5.65E-08
WDR86	0.125709281	0.015402281	0.027130349
WDR87	0.117403503	0.02372543	0.040113554
WDR88	0.129395309	0.012616523	0.022711337
WDR89	-0.056323448	0.27922387	0.349877221
WDR8	0.1373256	0.00807992	0.015220136
WDR90	0.103235923	0.046915045	0.073785547
WDR91	0.096852605	0.062377746	0.09484731
WDR92	-0.141845161	0.006204719	0.011978913
WDR93	0.044239256	0.395520344	0.471914493
WDSUB1	0.2297613	7.80E-06	2.63E-05
WDTC1	-0.204817244	7.07E-05	0.000200665
WDYHV1	0.382733649	2.17E-14	2.88E-13

WEE1	0.090747936	0.080874625	0.119170435
WEE2	-0.035998356	0.489401173	0.563510008
WFDC10A	0.171299982	0.000923484	0.002120522
WFDC10B	0.223719621	1.36E-05	4.39E-05
WFDC12	-0.022812535	0.661404619	0.72291122
WFDC13	0.152416199	0.003249959	0.006670686
WFDC1	0.11595879	0.025514238	0.042799995
WFDC2	0.313888772	6.31E-10	4.19E-09
WFDC3	0.197499679	0.000128635	0.000347812
WFDC5	0.000768095	0.988235917	0.991121423
WFDC6	0.095640547	0.065743147	0.099368874
WFDC8	0.154221367	0.002898265	0.006016384
WFIKKN1	-0.10067563	0.052680455	0.081736762
WFIKKN2	-0.117910514	0.023123879	0.039219642
WFS1	0.152206524	0.003293225	0.006748366
WHAMML1	0.037255648	0.474348738	0.549129848
WHAMML2	0.048096056	0.355588346	0.430587833
WHAMM	0.0208244	0.689306712	0.746364928
WHSC1L1	0.233239246	5.62E-06	1.95E-05
WHSC1	0.522577923	2.19E-27	2.09E-25
WHSC2	0.260493672	3.61E-07	1.53E-06
WIBG	0.137312408	0.008086063	0.015227471
WIF1	0.084193503	0.105429006	0.150565733
WIPF1	0.302435934	2.76E-09	1.67E-08
WIPF2	0.128084177	0.013551866	0.024217822
WIPF3	0.244608487	1.86E-06	6.97E-06
WIPI1	0.024283559	0.641054619	0.704767403
WIPI2	-0.230429587	7.33E-06	2.48E-05
WISP1	0.151879118	0.00336183	0.006878338
WISP2	0.07007249	0.178046936	0.237102344
WISP3	0.082891137	0.110946688	0.157360097
WIT1	0.243894709	2.00E-06	7.45E-06
WIZ	0.068318181	0.189184609	0.249730704
WLS	-0.037108291	0.476099697	0.550644886
WNK1	0.160966887	0.00186907	0.004045608
WNK2	0.463968316	3.34E-21	1.22E-19
WNK3	-0.25619379	5.68E-07	2.33E-06
WNK4	0.277147719	5.73E-08	2.78E-07
WNT10A	0.378731078	4.24E-14	5.39E-13
WNT10B	0.340567593	1.58E-11	1.35E-10
WNT11	0.007144385	0.89091384	0.91408485
WNT16	0.152539839	0.003224688	0.00662496
WNT1	0.1833079	0.000386753	0.000955349
WNT2B	0.148801375	0.004072986	0.00817964
WNT2	0.211396718	4.05E-05	0.000120341
WNT3A	0.272885303	9.29E-08	4.34E-07
WNT3	-0.105135066	0.04298839	0.068158581
WNT4	0.274232576	7.98E-08	3.77E-07
WNT5A	0.085469899	0.10023402	0.144063354
WNT5B	-0.053480197	0.304250679	0.376851656
WNT6	0.083481062	0.108419876	0.154281484
WNT7A	0.119261019	0.021585505	0.03682679
WNT7B	0.388859614	7.68E-15	1.10E-13
WNT8A	0.055576696	0.285657752	0.356861713
WNT8B	-0.101046251	0.051811038	0.080519502
WNT9A	0.285953913	2.06E-08	1.08E-07
WNT9B	-0.124924006	0.016061074	0.028163782
WRAP53	0.24242833	2.31E-06	8.53E-06

WRB	-0.045357524	0.383673404	0.459927887
WRNIP1	0.147522442	0.004406552	0.0087884
WRN	0.164662199	0.001459255	0.003229424
WSB1	0.31335434	6.77E-10	4.47E-09
WSB2	0.321971843	2.14E-10	1.54E-09
WSCD1	0.246126702	1.60E-06	6.05E-06
WSCD2	-0.069616643	0.180893127	0.240170135
WT1	0.285488328	2.18E-08	1.14E-07
WTAP	0.26197584	3.08E-07	1.32E-06
WTIP	0.182639166	0.000406554	0.000999675
WWC1	0.221807909	1.62E-05	5.14E-05
WWC2	-0.294354562	7.51E-09	4.24E-08
WWC3	0.114820464	0.027004417	0.045023182
WWOX	-0.135564758	0.00893765	0.016671898
WWP1	-0.126741006	0.014572808	0.02581765
WWP2	-0.165804452	0.00135037	0.003005818
WWTR1	0.026221815	0.614647887	0.680127484
XAB2	-0.058473892	0.261247475	0.330785063
XAF1	-0.126493613	0.014768045	0.026114769
XAGE1D	0.035900734	0.490580539	0.564704772
XAGE2	0.161910226	0.001755479	0.003815951
XAGE3	-0.013687798	0.792729216	0.834227006
XAGE5	0.008131725	0.875952527	0.902265976
XBP1	-0.08985178	0.083930022	0.123198785
XCL1	0.205178019	6.86E-05	0.000195222
XCL2	0.150458652	0.003674736	0.007457278
XCR1	0.024350766	0.640131133	0.703907514
XDH	-0.231149845	6.85E-06	2.33E-05
XG	0.038997733	0.453917749	0.52941569
XIAP	-0.126605817	0.014679213	0.025992293
XIRP1	0.152279953	0.003278013	0.006720653
XIRP2	0.100425899	0.053273106	0.082534164
XIST	0.101924106	0.04979934	0.077757069
XKR3	0.05039806	0.333007039	0.407078986
XKR4	0.18703605	0.00029185	0.000737659
XKR5	0.149743666	0.003842012	0.007762735
XKR6	0.225729633	1.13E-05	3.71E-05
XKR7	0.019217711	0.712169304	0.766040542
XKR8	0.091781118	0.077464344	0.114773513
XKR9	-0.081213129	0.118387649	0.166503264
XKRX	-0.094320702	0.069575896	0.104460849
XKRY2	-0.168283353	0.001139277	0.002574195
XK	0.007323928	0.888190157	0.911923846
XPA	-0.001234323	0.981096275	0.985349868
XPC	-0.392957025	3.79E-15	5.69E-14
XPNPEP1	0.243847521	2.00E-06	7.48E-06
XPNPEP2	0.010782025	0.836024168	0.870181477
XPNPEP3	-0.203623458	7.81E-05	0.000219854
XPO1	0.233020319	5.74E-06	1.99E-05
XPO4	0.13972348	0.007030065	0.013411181
XPO5	0.254723871	6.63E-07	2.69E-06
XPO6	0.294955602	6.98E-09	3.97E-08
XPO7	0.205352579	6.76E-05	0.000192644
XPOT	0.203132678	8.13E-05	0.000228132
XPR1	0.238724642	3.32E-06	1.20E-05
XRCC1	0.382133796	2.40E-14	3.16E-13
XRCC2	0.463837992	3.44E-21	1.25E-19
XRCC3	0.440427089	4.91E-19	1.32E-17

XRCC4	0.234853415	4.82E-06	1.69E-05
XRCC5	0.255790084	5.93E-07	2.42E-06
XRCC6BP1	-0.131714899	0.011100465	0.020249379
XRCC6	0.140540104	0.00670143	0.012842043
XRN1	-0.104478719	0.044312948	0.07009676
XRN2	-0.035461557	0.49590496	0.569780185
XRRA1	0.102372104	0.04879811	0.076433363
XYLB	-0.274256592	7.96E-08	3.76E-07
XYLT1	0.229190086	8.23E-06	2.76E-05
XYLT2	0.054084678	0.298809517	0.371034854
YAF2	-0.031220322	0.548864322	0.62000879
YAP1	-0.073667289	0.156755393	0.212282029
YARS2	0.34904537	4.54E-12	4.21E-11
YARS	0.235236474	4.65E-06	1.63E-05
YBX1	0.279957524	4.15E-08	2.07E-07
YBX2	-0.013215836	0.799721885	0.839722732
YDJC	0.27407385	8.13E-08	3.83E-07
YEATS2	0.501255297	5.36E-25	3.51E-23
YEATS4	0.088381706	0.089143093	0.129798911
YES1	0.082709161	0.111735415	0.158377299
YIF1A	-0.106358069	0.04060912	0.064845695
YIF1B	0.196522053	0.000139122	0.000373924
YIPF1	-0.060777107	0.242895845	0.310449157
YIPF2	0.1453626	0.005026222	0.009895484
YIPF3	-0.116272055	0.025116791	0.042183165
YIPF4	-0.159099373	0.002114004	0.004528029
YIPF5	0.139890487	0.006961707	0.013298603
YIPF6	-0.112615864	0.030104082	0.049601712
YIPF7	0.077867744	0.134379911	0.185820544
YJEFN3	0.310136101	1.03E-09	6.64E-09
YKT6	0.354150507	2.10E-12	2.06E-11
YLPM1	-0.022530248	0.665339021	0.726113644
YME1L1	0.029345162	0.573134128	0.642433922
YOD1	0.191962929	0.000199507	0.000520572
YPEL1	-0.472903145	4.53E-22	1.87E-20
YPEL2	-0.08872743	0.087894327	0.128214892
YPEL3	-0.036613163	0.482008811	0.556242867
YPEL4	0.343884353	9.75E-12	8.60E-11
YPEL5	-0.075168221	0.148457483	0.202545541
YRDC	0.020040924	0.700421548	0.756385411
YSK4	0.05402332	0.299358856	0.371601167
YTHDC1	0.060476321	0.245239843	0.313023331
YTHDC2	0.072382681	0.164131515	0.220949256
YTHDF1	0.243505422	2.07E-06	7.72E-06
YTHDF2	0.104180383	0.044926256	0.070971573
YTHDF3	-0.131542991	0.011207021	0.02042306
YWHAB	0.412267987	1.18E-16	2.25E-15
YWHAE	0.030933861	0.552539019	0.6237354
YWHAG	0.052981536	0.308788337	0.381523273
YWHAH	0.385935509	1.27E-14	1.73E-13
YWHAQ	0.365979991	3.35E-13	3.71E-12
YWHAZ	0.560064474	5.10E-32	1.11E-29
YY1AP1	0.151993214	0.003337776	0.006834037
YY1	-0.084038678	0.106073364	0.151385741
YY2	-0.024923619	0.632282157	0.696686161
ZACN	0.145051636	0.005121627	0.010064416
ZADH2	-0.29255054	9.36E-09	5.20E-08
ZAK	0.008914739	0.864118437	0.893210802

ZAN	0.12991999	0.01225847	0.02214082
ZAP70	0.173433598	0.000794362	0.001851342
ZAR1L	0.088814235	0.087583001	0.127844999
ZAR1	0.044293323	0.394942544	0.471281543
ZBBX	0.124726321	0.016230756	0.028426296
ZBED1	-0.325159444	1.39E-10	1.02E-09
ZBED2	0.308974812	1.20E-09	7.63E-09
ZBED3	-0.074196025	0.153793217	0.208809955
ZBED4	0.287885979	1.64E-08	8.73E-08
ZBED5	0.330291861	6.83E-11	5.31E-10
ZBP1	0.23525611	4.64E-06	1.63E-05
ZBTB10	-0.151385705	0.003467671	0.007075996
ZBTB11	0.021729018	0.676555993	0.735436705
ZBTB12	0.326768118	1.11E-10	8.33E-10
ZBTB16	-0.378823838	4.17E-14	5.32E-13
ZBTB17	0.088460249	0.088858152	0.129469181
ZBTB1	0.150585875	0.003645672	0.007405841
ZBTB20	-0.204071127	7.52E-05	0.000212506
ZBTB22	-0.153799245	0.002977256	0.006166222
ZBTB24	0.246627261	1.52E-06	5.78E-06
ZBTB25	0.108870817	0.036068171	0.058229344
ZBTB26	0.104926617	0.043405395	0.068737861
ZBTB2	0.27151647	1.08E-07	5.00E-07
ZBTB32	0.225956358	1.11E-05	3.64E-05
ZBTB33	-0.225752939	1.13E-05	3.70E-05
ZBTB34	0.326515553	1.15E-10	8.60E-10
ZBTB37	-0.226137186	1.09E-05	3.58E-05
ZBTB38	-0.212902898	3.56E-05	0.000106652
ZBTB39	5.87E-06	0.999910017	0.999910017
ZBTB3	-0.118309115	0.022660259	0.038502148
ZBTB40	0.243975655	1.98E-06	7.39E-06
ZBTB41	0.117478303	0.023635841	0.039979048
ZBTB42	-0.072144801	0.165525527	0.222510091
ZBTB43	0.036358187	0.485067253	0.559189018
ZBTB44	-0.123286157	0.017514957	0.030447903
ZBTB45	0.288836778	1.46E-08	7.88E-08
ZBTB46	0.17160014	0.000904217	0.002080118
ZBTB47	0.17307938	0.000814573	0.001894014
ZBTB48	-0.173058445	0.000815782	0.001896432
ZBTB49	0.147837112	0.00432227	0.008639336
ZBTB4	0.034690533	0.505326366	0.578569647
ZBTB5	0.106007598	0.041279294	0.065810347
ZBTB6	-0.033820921	0.516064041	0.588765767
ZBTB7A	-0.122468983	0.018282391	0.031660447
ZBTB7B	-0.02126255	0.683119677	0.74123694
ZBTB7C	-0.320844224	2.50E-10	1.77E-09
ZBTB8A	0.080975709	0.119471227	0.16782582
ZBTB8B	0.194162399	0.000167831	0.000444204
ZBTB8OS	0.174693086	0.000726192	0.001705033
ZBTB9	0.279356232	4.45E-08	2.20E-07
ZC3H10	0.069648618	0.180692394	0.239935605
ZC3H11A	0.063208495	0.224523252	0.290168811
ZC3H12A	0.227381104	9.74E-06	3.22E-05
ZC3H12B	0.175385706	0.000691052	0.001630405
ZC3H12C	-0.089947893	0.083597936	0.122783699
ZC3H12D	0.224475814	1.27E-05	4.11E-05
ZC3H13	-0.213309444	3.43E-05	0.000103132
ZC3H14	0.002888117	0.955786774	0.965477618

ZC3H15	0.167215895	0.001226147	0.002751752
ZC3H18	-0.024955063	0.63185251	0.696341523
ZC3H3	0.218612861	2.16E-05	6.70E-05
ZC3H4	0.140732935	0.006625864	0.012711909
ZC3H6	-0.339526235	1.84E-11	1.55E-10
ZC3H7A	0.05841423	0.261735207	0.331276289
ZC3H7B	-0.136422247	0.008510328	0.015937465
ZC3H8	-0.028049746	0.590191126	0.657851934
ZC3HAV1L	0.257000729	5.22E-07	2.15E-06
ZC3HAV1	0.057065681	0.272926899	0.343087435
ZC3HC1	-0.123029188	0.017753196	0.030821714
ZC4H2	0.222683013	1.50E-05	4.79E-05
ZCCHC10	0.364737625	4.08E-13	4.45E-12
ZCCHC11	0.088091405	0.090202628	0.131169107
ZCCHC12	0.166326683	0.001303119	0.002908013
ZCCHC13	0.001786662	0.972639973	0.978336541
ZCCHC14	-0.224341871	1.29E-05	4.16E-05
ZCCHC16	-0.167259347	0.001222495	0.002744175
ZCCHC17	0.256773707	5.35E-07	2.20E-06
ZCCHC18	0.205350972	6.76E-05	0.000192644
ZCCHC24	-0.472616448	4.84E-22	1.99E-20
ZCCHC2	-0.107801827	0.037944409	0.060952147
ZCCHC3	0.242469145	2.30E-06	8.49E-06
ZCCHC4	0.069855752	0.179396028	0.238542001
ZCCHC5	0.175898307	0.000666066	0.001576309
ZCCHC6	-0.347000833	6.16E-12	5.60E-11
ZCCHC7	0.106763813	0.039844749	0.063711942
ZCCHC8	0.218358619	2.21E-05	6.84E-05
ZCCHC9	0.189206377	0.000247113	0.000633262
ZCRB1	0.110587713	0.033219758	0.054157448
ZCWPW1	-0.23582253	4.39E-06	1.55E-05
ZCWPW2	-0.187558694	0.000280434	0.000711629
ZDBF2	0.272316957	9.90E-08	4.60E-07
ZDHHC11	0.113603544	0.028679641	0.047510046
ZDHHC12	0.065859623	0.205642109	0.268871962
ZDHHC13	0.600476527	1.01E-37	9.62E-35
ZDHHC14	0.130685236	0.011752328	0.021323304
ZDHHC15	-0.026447597	0.611603077	0.677435589
ZDHHC16	0.097395443	0.060917199	0.092824876
ZDHHC17	0.121230255	0.019501607	0.033564667
ZDHHC18	0.133579697	0.010000916	0.018463373
ZDHHC19	-0.103249156	0.046886679	0.073746756
ZDHHC1	0.143242454	0.00570973	0.011092077
ZDHHC20	0.06739332	0.195258307	0.256880142
ZDHHC21	0.154713493	0.002808583	0.005849111
ZDHHC22	0.137651254	0.007929586	0.014963836
ZDHHC23	0.372260153	1.22E-13	1.46E-12
ZDHHC24	0.190504502	0.000223506	0.000577896
ZDHHC2	0.203297974	8.02E-05	0.000225229
ZDHHC3	0.020771183	0.690059602	0.747017736
ZDHHC4	-0.087539524	0.09224469	0.133855249
ZDHHC5	0.102923296	0.047589397	0.074757606
ZDHHC6	-0.029547852	0.57048648	0.64004233
ZDHHC7	0.289819368	1.30E-08	7.08E-08
ZDHHC8P1	0.363649378	4.84E-13	5.21E-12
ZDHHC8	-0.200751827	9.88E-05	0.000273338
ZDHHC9	-0.017892805	0.731219128	0.782349489
ZEB1	-0.181845944	0.000431263	0.001055223

ZEB2	0.125010641	0.015987202	0.028058941
ZER1	0.091422282	0.078635301	0.116335397
ZFAND1	-0.011127707	0.830844871	0.865740442
ZFAND2A	-0.047462864	0.361964464	0.437537532
ZFAND2B	-0.028281452	0.587123166	0.654981952
ZFAND3	0.042151809	0.41821538	0.494198161
ZFAND5	-0.260797462	3.49E-07	1.48E-06
ZFAND6	-0.012225146	0.814451589	0.851956133
ZFATAS	0.029502113	0.571083443	0.640531716
ZFAT	0.292408249	9.52E-09	5.29E-08
ZFC3H1	0.328493643	8.77E-11	6.68E-10
ZFHX3	-0.172942377	0.000822516	0.001910255
ZFHX4	-0.241035487	2.65E-06	9.70E-06
ZFP106	-0.064828103	0.212847165	0.277001647
ZFP112	0.183233143	0.000388921	0.000960228
ZFP14	0.097517166	0.060593597	0.092437831
ZFP161	-0.217484207	2.38E-05	7.34E-05
ZFP1	-0.272881474	9.29E-08	4.34E-07
ZFP28	0.098615537	0.057737249	0.088582324
ZFP2	-0.021163735	0.684513195	0.742465937
ZFP30	0.086376758	0.096668142	0.139491722
ZFP36L1	-0.146339274	0.004736893	0.00938802
ZFP36L2	-0.00253233	0.961228822	0.969452772
ZFP36	-0.004636437	0.929079364	0.944776637
ZFP37	0.005857217	0.910475946	0.929655158
ZFP3	0.236780364	4.01E-06	1.42E-05
ZFP41	0.104092272	0.045108752	0.071208918
ZFP42	0.209167172	4.90E-05	0.000143236
ZFP57	0.105240588	0.04277858	0.067874711
ZFP62	0.285418542	2.20E-08	1.15E-07
ZFP64	0.253150212	7.80E-07	3.12E-06
ZFP82	0.230974343	6.96E-06	2.37E-05
ZFP90	-0.135014002	0.009222081	0.017154277
ZFP91-CNTl	-0.014382691	0.782463862	0.825576357
ZFP91	-0.005546169	0.915211764	0.933677221
ZFP92	0.16631132	0.001304487	0.002910516
ZFPL1	0.150911043	0.003572331	0.007267973
ZFPM1	-0.075358447	0.147430133	0.201309329
ZFPM2	-0.010502028	0.840224518	0.873458195
ZFR2	0.221852791	1.61E-05	5.12E-05
ZFR	0.052584753	0.312430616	0.385282744
ZFX	0.130087991	0.012145734	0.021957106
ZFYVE16	0.257122018	5.15E-07	2.13E-06
ZFYVE19	0.148624461	0.004117725	0.008258665
ZFYVE1	-0.319637282	2.94E-10	2.06E-09
ZFYVE20	-0.099648409	0.055153828	0.085110345
ZFYVE21	-0.198524652	0.00011844	0.000322741
ZFYVE26	0.04293153	0.409650167	0.485863584
ZFYVE27	0.015449191	0.766782622	0.812053937
ZFYVE28	-0.093370052	0.072448207	0.108202502
ZFYVE9	-0.278223212	5.07E-08	2.49E-07
ZFY	-0.092461722	0.075282263	0.111873283
ZG16B	0.35760387	1.24E-12	1.25E-11
ZG16	-0.154661532	0.002817932	0.005867353
ZGLP1	0.131673305	0.011126165	0.020292543
ZGPAT	-0.205160692	6.87E-05	0.000195476
ZHX1	-0.028979446	0.577925938	0.647040605
ZHX2	-0.081858828	0.115479503	0.162942323

ZHX3	-0.232994822	5.75E-06	1.99E-05
ZIC1	-0.133205286	0.010213594	0.01880895
ZIC2	0.226654664	1.04E-05	3.43E-05
ZIC3	0.052521384	0.313014907	0.385859971
ZIC4	-0.074628201	0.151403637	0.205972634
ZIC5	0.323495672	1.74E-10	1.27E-09
ZIK1	0.144978371	0.005144341	0.010102075
ZIM2	0.095353104	0.066562756	0.100451725
ZIM3	-0.031878948	0.540461384	0.612078431
ZKSCAN1	-0.281941791	3.30E-08	1.67E-07
ZKSCAN2	-0.210036074	4.55E-05	0.000134017
ZKSCAN3	0.171825864	0.000889974	0.002052257
ZKSCAN4	0.119734747	0.021067308	0.036019816
ZKSCAN5	0.00965404	0.852972741	0.884357166
ZMAT1	-0.360308173	8.16E-13	8.48E-12
ZMAT2	-0.060714711	0.243380792	0.311009117
ZMAT3	-0.144178784	0.00539819	0.010538241
ZMAT4	0.190295503	0.000227159	0.000586123
ZMAT5	0.074185345	0.153852624	0.208862145
ZMIZ1	0.260950798	3.43E-07	1.46E-06
ZMIZ2	0.195376135	0.000152434	0.00040675
ZMPSTE24	0.009835927	0.850235006	0.881819733
ZMYM1	0.126900731	0.014447964	0.025617686
ZMYM2	0.146795596	0.00460693	0.009148651
ZMYM3	0.203538389	7.86E-05	0.00022122
ZMYM4	0.109632084	0.034780572	0.056401999
ZMYM5	0.104103898	0.045084636	0.071176489
ZMYM6	0.027713697	0.594653694	0.66202429
ZMYND10	0.200954183	9.72E-05	0.000269311
ZMYND11	-0.123660971	0.01717247	0.029909945
ZMYND12	-0.47966119	9.62E-23	4.44E-21
ZMYND15	0.183338966	0.000385855	0.000953249
ZMYND17	0.056794131	0.275219361	0.345557291
ZMYND19	0.320577116	2.59E-10	1.83E-09
ZMYND8	0.07119699	0.171168012	0.229167281
ZNF100	0.259985398	3.81E-07	1.61E-06
ZNF101	0.373866968	9.42E-14	1.14E-12
ZNF107	0.40090434	9.32E-16	1.54E-14
ZNF10	0.015359419	0.768099014	0.813118049
ZNF114	0.161191526	0.001841426	0.003990525
ZNF117	0.145954893	0.00484892	0.009585254
ZNF121	-0.041585933	0.424496755	0.500848347
ZNF124	0.319121035	3.15E-10	2.19E-09
ZNF12	0.079382907	0.126942002	0.176898333
ZNF131	0.162253595	0.001715728	0.003736415
ZNF132	-0.074051153	0.154600594	0.209748913
ZNF133	0.223077234	1.44E-05	4.63E-05
ZNF134	0.017764497	0.733072982	0.783659581
ZNF135	0.144261654	0.005371367	0.010493075
ZNF136	0.060619188	0.244124524	0.311839495
ZNF137	0.198496464	0.00011871	0.000323433
ZNF138	0.108588585	0.036555711	0.058954333
ZNF140	0.095134411	0.067191928	0.101323965
ZNF141	0.233137113	5.68E-06	1.97E-05
ZNF142	0.338030655	2.28E-11	1.90E-10
ZNF143	0.275615293	6.83E-08	3.26E-07
ZNF146	0.130880314	0.011626291	0.021111929
ZNF148	-0.022508036	0.665648982	0.726235434

ZNF14	0.323870719	1.66E-10	1.21E-09
ZNF154	0.101096168	0.051694858	0.080376568
ZNF155	0.067695308	0.193259643	0.254536467
ZNF157	0.112328367	0.030529924	0.050220226
ZNF160	0.145178345	0.005082557	0.009996515
ZNF165	0.352191646	2.83E-12	2.71E-11
ZNF167	0.100338813	0.053481073	0.082817717
ZNF169	0.29391218	7.93E-09	4.46E-08
ZNF16	0.271694097	1.06E-07	4.91E-07
ZNF174	0.10217282	0.049241392	0.077018686
ZNF175	-0.208627031	5.13E-05	0.000149368
ZNF177	0.180487915	0.000476834	0.00115805
ZNF17	-0.084354617	0.104761764	0.149706898
ZNF180	0.158958229	0.002133658	0.004567671
ZNF181	-0.163270025	0.001602839	0.003508984
ZNF182	0.177212951	0.000605759	0.001444569
ZNF184	0.108157124	0.037311841	0.060037678
ZNF185	0.287256539	1.77E-08	9.36E-08
ZNF187	0.105563936	0.042141027	0.066980497
ZNF189	0.005917114	0.909564359	0.928914808
ZNF18	-0.260543108	3.59E-07	1.52E-06
ZNF192	-0.065235349	0.209981203	0.273718873
ZNF193	0.138879026	0.007384988	0.014025141
ZNF195	0.391250889	5.09E-15	7.50E-14
ZNF197	-0.112032718	0.030973204	0.050840255
ZNF19	-0.131667688	0.011129639	0.020297022
ZNF200	0.018692038	0.719706936	0.772813108
ZNF202	0.232522477	6.02E-06	2.07E-05
ZNF204P	0.288820645	1.47E-08	7.89E-08
ZNF205	0.139233612	0.007234051	0.013764734
ZNF207	0.490352747	7.71E-24	4.09E-22
ZNF208	0.156933223	0.002434619	0.005152469
ZNF20	0.179271496	0.000521411	0.001258334
ZNF211	0.226642024	1.04E-05	3.43E-05
ZNF212	-0.202544033	8.53E-05	0.000238698
ZNF213	0.365259458	3.75E-13	4.12E-12
ZNF214	-0.226018169	1.10E-05	3.62E-05
ZNF215	0.228815461	8.52E-06	2.85E-05
ZNF217	0.057929645	0.265719881	0.335360878
ZNF219	0.156946102	0.002432588	0.005149508
ZNF221	0.05417813	0.297974136	0.370112896
ZNF222	0.158777291	0.002159098	0.004614692
ZNF223	0.069339925	0.18263722	0.242055398
ZNF224	0.038300197	0.462038465	0.536969449
ZNF225	-0.023702876	0.649056765	0.712015442
ZNF226	0.132400621	0.01068429	0.019569037
ZNF227	0.057471514	0.269525097	0.339410808
ZNF229	0.102137917	0.049319374	0.077119149
ZNF22	-0.08065153	0.120963248	0.169682426
ZNF230	0.189845005	0.000235222	0.000605036
ZNF232	0.235728351	4.43E-06	1.56E-05
ZNF233	0.271279764	1.11E-07	5.13E-07
ZNF234	0.144588292	0.005266807	0.0103141
ZNF235	0.031499853	0.545290148	0.616520478
ZNF236	0.033507084	0.519967935	0.592269937
ZNF238	-0.29306186	8.79E-09	4.92E-08
ZNF239	0.38773755	9.31E-15	1.31E-13
ZNF23	-0.134449277	0.009522053	0.017662788

ZNF248	0.201884279	9.01E-05	0.000250879
ZNF24	-0.074659452	0.15123194	0.205803531
ZNF250	0.262828558	2.81E-07	1.21E-06
ZNF251	0.272257353	9.97E-08	4.63E-07
ZNF252	-0.077442403	0.136527096	0.188319104
ZNF253	0.133881801	0.009832184	0.018187258
ZNF254	-0.054427118	0.295755965	0.367728473
ZNF256	0.210362343	4.43E-05	0.000130699
ZNF257	0.229051441	8.34E-06	2.80E-05
ZNF259	0.087525777	0.092296026	0.133919987
ZNF25	-0.15516739	0.002728113	0.005700595
ZNF260	0.17238691	0.000855463	0.001980538
ZNF263	0.043816179	0.400059215	0.476189262
ZNF264	0.039458503	0.448597736	0.524408174
ZNF266	0.128928514	0.012942791	0.023233581
ZNF267	0.262229668	2.99E-07	1.29E-06
ZNF268	-0.155299458	0.002705096	0.005657246
ZNF26	0.33507668	3.48E-11	2.81E-10
ZNF271	0.169651945	0.00103619	0.002357042
ZNF273	0.377799669	4.94E-14	6.23E-13
ZNF274	-0.036468751	0.483739753	0.557861844
ZNF275	-0.179313827	0.000519797	0.001254742
ZNF276	-0.092093921	0.076455179	0.113421704
ZNF277	-0.267375638	1.71E-07	7.67E-07
ZNF280A	0.244356991	1.91E-06	7.14E-06
ZNF280B	0.224119453	1.31E-05	4.25E-05
ZNF280C	0.118890865	0.021998062	0.037447269
ZNF280D	-0.197684843	0.000126734	0.000343208
ZNF281	-0.103976758	0.045348943	0.071554067
ZNF282	0.005444887	0.916754478	0.934868074
ZNF283	0.272305479	9.91E-08	4.60E-07
ZNF284	0.050888181	0.328320966	0.402437106
ZNF285	0.117497117	0.023613354	0.039951176
ZNF286A	0.203307633	8.01E-05	0.000225101
ZNF286B	0.143968339	0.005466847	0.010661824
ZNF287	0.223151193	1.43E-05	4.60E-05
ZNF28	0.269112957	1.41E-07	6.42E-07
ZNF292	0.291984256	1.00E-08	5.54E-08
ZNF295	-0.163240749	0.001605993	0.003513958
ZNF296	0.500940136	5.80E-25	3.76E-23
ZNF2	-0.004384227	0.932927937	0.947916069
ZNF300	0.279554739	4.35E-08	2.16E-07
ZNF302	0.027518529	0.597252462	0.664273311
ZNF304	0.094104049	0.070222164	0.105286127
ZNF30	0.005160657	0.921085467	0.938324027
ZNF311	0.065475187	0.208306441	0.271891562
ZNF317	0.020747377	0.690396493	0.747301218
ZNF318	0.033193365	0.523885472	0.59591449
ZNF319	0.059262076	0.254862829	0.323709103
ZNF320	0.300354546	3.58E-09	2.12E-08
ZNF321	0.342643734	1.17E-11	1.02E-10
ZNF322A	0.144294554	0.005360752	0.010476447
ZNF322B	0.141639757	0.006280639	0.012113748
ZNF323	-0.041390413	0.426679782	0.503006959
ZNF324B	-0.018281729	0.725609323	0.777349372
ZNF324	0.035737208	0.492559474	0.566557151
ZNF326	0.10569554	0.041883842	0.066619553
ZNF329	0.045032525	0.387093935	0.463406166

ZNF32	0.174794595	0.000720941	0.001693622
ZNF330	-0.319029264	3.19E-10	2.22E-09
ZNF331	0.139494576	0.007124734	0.013578776
ZNF333	0.166971429	0.001246881	0.002794191
ZNF334	0.019165998	0.712909588	0.766629861
ZNF335	0.370724959	1.57E-13	1.83E-12
ZNF337	0.283146219	2.87E-08	1.47E-07
ZNF33A	-0.091126314	0.079611868	0.117631657
ZNF33B	-0.017573798	0.735831169	0.785853569
ZNF341	0.359021253	9.96E-13	1.02E-11
ZNF343	-0.022748434	0.662297217	0.723688304
ZNF345	0.12548546	0.015587594	0.027420401
ZNF346	0.374041145	9.16E-14	1.11E-12
ZNF347	0.150241387	0.003724856	0.007548223
ZNF34	0.043099555	0.407818111	0.484007649
ZNF350	0.077824052	0.13459927	0.186059301
ZNF354A	0.384457096	1.63E-14	2.20E-13
ZNF354B	0.182525278	0.000410019	0.001007325
ZNF354C	0.051108175	0.326231509	0.400343976
ZNF358	-0.215528455	2.83E-05	8.61E-05
ZNF35	0.307196238	1.51E-09	9.46E-09
ZNF362	-0.045435423	0.382856283	0.459141757
ZNF365	0.288707745	1.49E-08	7.99E-08
ZNF366	-0.122276963	0.01846692	0.031955006
ZNF367	0.109901731	0.03433397	0.055759465
ZNF37A	0.107273411	0.038901987	0.06232953
ZNF37B	0.326919631	1.09E-10	8.17E-10
ZNF382	0.183828318	0.000371968	0.000921456
ZNF383	0.035924862	0.490288901	0.564434296
ZNF384	0.346331387	6.80E-12	6.15E-11
ZNF385A	0.299081314	4.19E-09	2.46E-08
ZNF385B	-0.483943101	3.54E-23	1.70E-21
ZNF385D	-0.157837946	0.002295672	0.004879896
ZNF389	0.058352425	0.262241115	0.331832285
ZNF391	0.198533679	0.000118354	0.00032255
ZNF394	-0.023831307	0.647283387	0.710439074
ZNF395	-0.140340222	0.006780571	0.012981217
ZNF396	-0.154443923	0.00285739	0.005942176
ZNF397OS	0.147840067	0.004321485	0.008638634
ZNF397	-0.116456632	0.024885122	0.041861133
ZNF398	-0.208881768	5.02E-05	0.000146494
ZNF3	-0.185792712	0.000320774	0.000804944
ZNF404	0.189732525	0.000237277	0.000610018
ZNF407	-0.140374657	0.006766878	0.012956246
ZNF408	0.102294883	0.04896948	0.076658821
ZNF410	0.239632201	3.04E-06	1.10E-05
ZNF414	0.13669153	0.008379939	0.015712292
ZNF415	0.136161627	0.00863824	0.016155746
ZNF416	0.100455207	0.053203266	0.082451611
ZNF417	-0.022133212	0.670888272	0.731030803
ZNF418	0.166327145	0.001303078	0.002908013
ZNF419	0.143101699	0.005757934	0.011174824
ZNF41	-0.038805561	0.456146984	0.531393498
ZNF420	-0.06971592	0.18027042	0.239518961
ZNF423	-0.044066689	0.397367932	0.473636708
ZNF425	-0.325514427	1.32E-10	9.77E-10
ZNF426	0.001900777	0.970893215	0.977072875
ZNF428	0.177987737	0.000572629	0.001372133

ZNF429	-0.298268611	4.64E-09	2.70E-08
ZNF430	0.23084623	7.05E-06	2.40E-05
ZNF431	0.322190235	2.08E-10	1.50E-09
ZNF432	0.146983141	0.004554456	0.009053469
ZNF433	0.002431281	0.962774784	0.970618858
ZNF434	0.073604902	0.157107733	0.212701343
ZNF436	0.041379838	0.426798038	0.503090219
ZNF438	0.255494647	6.11E-07	2.49E-06
ZNF439	0.298542953	4.49E-09	2.62E-08
ZNF43	0.287362355	1.74E-08	9.25E-08
ZNF440	0.046595139	0.370817363	0.44661294
ZNF441	-0.156462568	0.002509893	0.005297393
ZNF442	-0.115494059	0.026113833	0.043695598
ZNF443	0.029183019	0.575256295	0.644411313
ZNF444	0.002609174	0.960053263	0.968659464
ZNF445	0.066433244	0.20171263	0.264428441
ZNF446	0.196768035	0.00013641	0.000367189
ZNF449	0.06433437	0.216359357	0.281076695
ZNF44	0.079512028	0.126323203	0.176146907
ZNF451	-0.097046237	0.061853489	0.094121998
ZNF454	0.107428391	0.038619041	0.06190108
ZNF45	0.107440271	0.038597423	0.061876387
ZNF460	-0.084868198	0.102657067	0.147110782
ZNF461	0.23038741	7.36E-06	2.49E-05
ZNF462	-0.016701042	0.74849715	0.796685379
ZNF467	0.065400086	0.20882982	0.272449749
ZNF468	0.265057461	2.21E-07	9.71E-07
ZNF469	0.343076476	1.10E-11	9.61E-11
ZNF470	-0.034031003	0.51345927	0.586196882
ZNF471	0.04199268	0.419976216	0.496072938
ZNF473	0.231224262	6.80E-06	2.32E-05
ZNF474	0.029628502	0.569434603	0.639078146
ZNF479	0.019229735	0.711997212	0.765965948
ZNF480	0.183125867	0.000392052	0.00096712
ZNF483	0.137352597	0.008067361	0.015200792
ZNF484	0.029522239	0.570820729	0.640273102
ZNF485	0.198829655	0.000115556	0.00031527
ZNF486	0.255111082	6.36E-07	2.59E-06
ZNF487	-0.024680468	0.635608725	0.69986166
ZNF488	0.352490223	2.71E-12	2.60E-11
ZNF48	0.215816419	2.76E-05	8.41E-05
ZNF490	0.090348099	0.082226549	0.120947823
ZNF491	0.03899956	0.453896584	0.52941569
ZNF492	0.170588599	0.00097067	0.00222119
ZNF493	0.054350983	0.296433067	0.368428446
ZNF496	0.28012444	4.07E-08	2.03E-07
ZNF497	-0.148934088	0.004039714	0.008121007
ZNF498	0.130557538	0.011835484	0.021452834
ZNF500	-0.058977031	0.257159228	0.326199665
ZNF501	0.032211539	0.536242566	0.608061039
ZNF502	0.063188413	0.224670827	0.290265384
ZNF503	-0.244105124	1.95E-06	7.30E-06
ZNF506	0.206009725	6.40E-05	0.000183263
ZNF507	-0.02339656	0.653294358	0.715735272
ZNF510	-0.040497186	0.436735419	0.512818969
ZNF511	-0.085380722	0.10059024	0.144533628
ZNF512B	0.072088343	0.165857678	0.222911466
ZNF512	0.132401679	0.010683659	0.019569037

ZNF513	0.085342555	0.100743007	0.144721818
ZNF514	0.196499293	0.000139376	0.000374513
ZNF516	-0.087408983	0.092733084	0.134446437
ZNF517	0.022167173	0.670412904	0.730779501
ZNF518A	0.074220482	0.153657231	0.208667986
ZNF518B	0.198873576	0.000115146	0.000314238
ZNF519	0.35169229	3.05E-12	2.91E-11
ZNF521	-0.060401574	0.245824783	0.313669463
ZNF524	-0.013574146	0.794411605	0.835600211
ZNF525	0.31749791	3.91E-10	2.69E-09
ZNF526	0.147066808	0.00453122	0.009009978
ZNF527	0.020005316	0.700928245	0.756850542
ZNF528	0.038736117	0.456954052	0.532022595
ZNF529	0.185628074	0.000324798	0.00081371
ZNF530	0.314090369	6.15E-10	4.09E-09
ZNF532	0.386455982	1.16E-14	1.60E-13
ZNF534	-0.015257671	0.769591837	0.814481969
ZNF536	0.101312343	0.051194229	0.079679018
ZNF540	-0.160591577	0.001916103	0.004137503
ZNF541	-0.191201549	0.000211717	0.000549913
ZNF542	0.01318687	0.800151574	0.839908302
ZNF543	0.108561221	0.036603278	0.059021489
ZNF544	0.177013107	0.000614588	0.001463871
ZNF546	-0.205888102	6.46E-05	0.000184846
ZNF547	0.15808168	0.002259498	0.004809157
ZNF548	0.088866548	0.087395809	0.127618508
ZNF549	0.156932309	0.002434763	0.005152469
ZNF550	0.16221954	0.001719633	0.003743692
ZNF551	0.099074957	0.056576036	0.087055518
ZNF552	0.219678821	1.96E-05	6.13E-05
ZNF554	-0.081109887	0.118857892	0.167058482
ZNF555	0.098706129	0.057506725	0.088289841
ZNF556	0.099854081	0.054651031	0.084432558
ZNF557	0.018052492	0.72891407	0.780302316
ZNF558	-0.021560294	0.678927323	0.737571716
ZNF559	0.09622749	0.064095279	0.09716224
ZNF560	0.116642618	0.024653551	0.041509555
ZNF561	-0.070884683	0.173058302	0.231324979
ZNF562	0.038142401	0.463886666	0.538771653
ZNF563	-0.2214507	1.67E-05	5.30E-05
ZNF564	-0.029683674	0.568715562	0.638415024
ZNF565	0.115951298	0.02552381	0.0428054
ZNF566	-0.021178419	0.684306038	0.742281655
ZNF567	0.249753062	1.11E-06	4.32E-06
ZNF568	0.012570941	0.809302562	0.847812887
ZNF569	0.027354739	0.599437368	0.666331394
ZNF570	-0.036498832	0.483378913	0.557542537
ZNF571	0.015765143	0.762154922	0.808371951
ZNF572	0.085346653	0.100726598	0.144719117
ZNF573	-0.130619255	0.011795229	0.02139335
ZNF574	0.125511215	0.015566171	0.027387553
ZNF575	-0.262387101	2.94E-07	1.27E-06
ZNF576	0.052839839	0.310085825	0.382888918
ZNF577	0.017091936	0.742815656	0.792034548
ZNF578	0.197146801	0.000132332	0.000356886
ZNF579	0.209222972	4.88E-05	0.000142642
ZNF57	0.187581875	0.000279937	0.000710524
ZNF580	0.270374892	1.23E-07	5.64E-07

ZNF581	0.194171164	0.000167714	0.000443955
ZNF582	-0.035081094	0.500542262	0.574181087
ZNF583	-0.027172028	0.601878914	0.668597731
ZNF584	-0.110870409	0.032769637	0.053511205
ZNF585A	0.144933394	0.005158329	0.010126551
ZNF585B	-0.004591376	0.929766845	0.945379222
ZNF586	0.248521794	1.25E-06	4.85E-06
ZNF587	0.173153129	0.000810326	0.001885289
ZNF589	0.319843553	2.86E-10	2.00E-09
ZNF592	-0.136164017	0.008637059	0.016155055
ZNF593	-0.020530066	0.69347463	0.750184708
ZNF594	0.209950498	4.59E-05	0.000134761
ZNF595	0.008753881	0.866547168	0.895139889
ZNF596	-0.107654595	0.03820919	0.061323087
ZNF597	0.001267928	0.98058172	0.984882734
ZNF598	0.032498357	0.532617673	0.604501184
ZNF599	-0.054223503	0.297569096	0.369725054
ZNF600	0.199365402	0.000110649	0.000303128
ZNF605	0.321518275	2.28E-10	1.63E-09
ZNF606	0.127995465	0.013617301	0.024318376
ZNF607	0.278431857	4.95E-08	2.43E-07
ZNF608	0.242036547	2.40E-06	8.85E-06
ZNF609	0.108053386	0.037495606	0.060299278
ZNF610	0.211449825	4.03E-05	0.000119864
ZNF611	0.256317622	5.61E-07	2.30E-06
ZNF613	0.112642412	0.030065017	0.049549575
ZNF614	0.112357132	0.030487087	0.050158043
ZNF615	-0.024346769	0.64018603	0.703929026
ZNF616	-0.191393368	0.000208577	0.000542676
ZNF618	0.330299284	6.82E-11	5.30E-10
ZNF619	-0.174631634	0.000729389	0.00171173
ZNF620	0.241171109	2.61E-06	9.58E-06
ZNF621	0.095912823	0.064974447	0.098337835
ZNF622	0.082656057	0.111966411	0.158614438
ZNF623	0.152694875	0.003193253	0.006567828
ZNF624	0.293611218	8.22E-09	4.62E-08
ZNF625	0.13922694	0.007236865	0.013768774
ZNF626	0.108123532	0.037371263	0.060104165
ZNF627	-0.041587399	0.424480408	0.500848347
ZNF628	0.142496951	0.005969225	0.011555621
ZNF629	0.179721996	0.000504469	0.001220405
ZNF630	0.241406106	2.55E-06	9.37E-06
ZNF638	0.041274566	0.427976302	0.504356338
ZNF639	0.143380885	0.005662677	0.011006034
ZNF641	-0.012726744	0.806985254	0.84570017
ZNF642	0.229914008	7.69E-06	2.60E-05
ZNF643	0.194532719	0.000162985	0.000432586
ZNF644	0.019841169	0.703265746	0.75875766
ZNF645	0.041924132	0.420736078	0.496852646
ZNF646	0.097020634	0.0619226	0.094219968
ZNF648	-0.407107254	3.04E-16	5.41E-15
ZNF649	0.023317363	0.654391792	0.716622321
ZNF652	-0.042599123	0.413288864	0.489450763
ZNF653	0.094618838	0.068694524	0.103340051
ZNF654	-0.122875464	0.017897063	0.031047134
ZNF655	-0.044733368	0.390258805	0.466670823
ZNF658	-0.092918259	0.073846768	0.110026809
ZNF660	0.144330119	0.005349298	0.010456114

ZNF662	0.00745423	0.88621429	0.910294461
ZNF664	0.339832731	1.76E-11	1.49E-10
ZNF665	0.178690495	0.00054404	0.001308824
ZNF667	0.102657656	0.048168754	0.075590835
ZNF668	0.370703875	1.57E-13	1.84E-12
ZNF669	0.246697143	1.51E-06	5.74E-06
ZNF670	0.15933962	0.002080931	0.004464387
ZNF671	0.028608442	0.582806122	0.651333084
ZNF672	0.139903286	0.006956493	0.013289917
ZNF673	0.067466543	0.194772313	0.256308476
ZNF674	0.002265139	0.965316928	0.972837102
ZNF675	0.274355157	7.87E-08	3.72E-07
ZNF676	0.081997388	0.114862801	0.162210018
ZNF677	0.060054239	0.248555668	0.316626767
ZNF678	0.14342211	0.005648731	0.010983215
ZNF679	0.003209513	0.950872505	0.961929723
ZNF680	-0.344928783	8.36E-12	7.47E-11
ZNF681	0.292870807	9.00E-09	5.02E-08
ZNF682	0.16371704	0.001555382	0.003416729
ZNF683	0.225134673	1.20E-05	3.90E-05
ZNF684	-0.078231162	0.132566008	0.183579871
ZNF687	0.110450462	0.03344019	0.054485602
ZNF688	-0.18911413	0.000248876	0.000637534
ZNF689	-0.127430665	0.014040461	0.024965553
ZNF691	-0.115916387	0.02556845	0.042876656
ZNF692	0.2692368	1.39E-07	6.34E-07
ZNF695	0.366817789	2.93E-13	3.28E-12
ZNF696	0.170924093	0.000948146	0.002173144
ZNF697	0.007045982	0.892407169	0.915148474
ZNF699	-0.118825306	0.022071838	0.037556812
ZNF69	-0.001661666	0.974553424	0.979766518
ZNF700	0.22398726	1.33E-05	4.29E-05
ZNF701	0.227353401	9.76E-06	3.23E-05
ZNF702P	0.254423395	6.84E-07	2.77E-06
ZNF703	0.101667329	0.050380907	0.07856046
ZNF704	0.021950979	0.673441305	0.732916849
ZNF705A	0.052606362	0.31223153	0.385084909
ZNF705D	0.016614093	0.749762785	0.797734149
ZNF706	0.247933713	1.33E-06	5.12E-06
ZNF707	0.249971286	1.08E-06	4.24E-06
ZNF708	0.275140603	7.20E-08	3.43E-07
ZNF709	0.118225263	0.022757116	0.038643646
ZNF70	0.258223875	4.59E-07	1.91E-06
ZNF710	0.201349284	9.41E-05	0.000261181
ZNF711	0.39169329	4.71E-15	6.99E-14
ZNF713	0.248261417	1.29E-06	4.97E-06
ZNF714	0.322640744	1.96E-10	1.41E-09
ZNF716	-0.071246731	0.170868374	0.228827625
ZNF717	-0.187238293	0.000287382	0.000727566
ZNF718	0.153763498	0.002984034	0.006178976
ZNF71	0.352751492	2.60E-12	2.50E-11
ZNF720	-0.136262895	0.008588336	0.016071466
ZNF721	0.025346689	0.626511757	0.691411878
ZNF727	0.080546323	0.121450567	0.170258124
ZNF732	0.072617264	0.162765473	0.219391999
ZNF735	-0.024910973	0.632454989	0.696774224
ZNF737	0.249903211	1.09E-06	4.26E-06
ZNF738	0.377759015	4.98E-14	6.27E-13

ZNF740	0.04246541	0.414757923	0.490723799
ZNF746	0.039599278	0.446979417	0.52273108
ZNF747	-0.175174672	0.000701589	0.001652719
ZNF749	0.058332306	0.262405952	0.33199869
ZNF74	0.122633534	0.01812554	0.031410665
ZNF750	0.08463324	0.103615748	0.148271293
ZNF75A	0.235548654	4.51E-06	1.59E-05
ZNF75D	-0.142141392	0.006096676	0.011781654
ZNF761	0.321011997	2.44E-10	1.73E-09
ZNF763	0.067606703	0.193844509	0.255222411
ZNF764	0.157352765	0.00236926	0.005025595
ZNF765	0.32811939	9.24E-11	7.00E-10
ZNF766	0.137833507	0.007846545	0.014822574
ZNF767	0.111707658	0.031466921	0.051582655
ZNF768	0.099206091	0.05624816	0.08661121
ZNF76	0.022762537	0.6621008	0.723513364
ZNF770	-0.323746641	1.69E-10	1.23E-09
ZNF771	-0.077202109	0.137751737	0.189784931
ZNF772	0.12781346	0.013752421	0.024529497
ZNF773	0.183268713	0.000387888	0.000958034
ZNF774	0.025697077	0.621749839	0.686689228
ZNF775	0.159906922	0.0020047	0.004313851
ZNF776	-0.070942736	0.172705758	0.23093121
ZNF777	-0.042366009	0.415851997	0.491813802
ZNF778	-0.178786376	0.000540244	0.001300319
ZNF77	0.177415274	0.00059694	0.001425587
ZNF780A	0.073695041	0.156598856	0.212113299
ZNF780B	0.100590689	0.052881413	0.082010238
ZNF781	0.088406743	0.089052184	0.129694979
ZNF782	0.121999989	0.018735949	0.03236827
ZNF784	-0.1181445	0.022850742	0.038789408
ZNF785	0.171629397	0.000902359	0.002076563
ZNF786	0.015189635	0.770590519	0.815365643
ZNF787	0.187184931	0.000288554	0.000730101
ZNF788	0.13294887	0.010361551	0.019047967
ZNF789	0.166673912	0.001272549	0.002847205
ZNF790	-0.215874241	2.75E-05	8.37E-05
ZNF791	-0.119253949	0.021593321	0.03683697
ZNF792	0.341840901	1.31E-11	1.14E-10
ZNF793	0.154300431	0.002883684	0.00598986
ZNF799	-0.061782777	0.235172805	0.301939377
ZNF79	0.011753857	0.821482136	0.858138908
ZNF7	0.196911948	0.000134847	0.000363177
ZNF800	-0.150437121	0.003679676	0.00746426
ZNF804A	0.154834475	0.002786926	0.005807044
ZNF804B	0.026830118	0.606459785	0.672486465
ZNF805	-0.009048921	0.862093437	0.891632512
ZNF808	0.136223322	0.008607806	0.016104876
ZNF80	0.133786079	0.009885371	0.018270375
ZNF813	0.146648249	0.004648539	0.009226682
ZNF814	0.143785634	0.00552709	0.010769825
ZNF815	0.028343367	0.586304609	0.65426061
ZNF816A	0.313768714	6.41E-10	4.26E-09
ZNF81	0.043120107	0.407594356	0.483770912
ZNF821	0.030850323	0.553612893	0.624735248
ZNF823	-0.042193048	0.417759751	0.493747613
ZNF826	0.256406093	5.56E-07	2.28E-06
ZNF827	0.139760694	0.007014781	0.013385332

ZNF828	0.037988241	0.465696236	0.5406212
ZNF829	0.028535083	0.58377336	0.652070048
ZNF830	-0.104927705	0.043403209	0.068737861
ZNF831	0.065784082	0.206163692	0.269430141
ZNF833	-0.005994192	0.908391454	0.928097572
ZNF835	0.075248736	0.148021986	0.202020553
ZNF836	-0.004638846	0.929042604	0.944776637
ZNF837	-0.264123393	2.44E-07	1.07E-06
ZNF839	-0.17628296	0.000647869	0.001538544
ZNF83	0.284117396	2.56E-08	1.32E-07
ZNF841	0.159403666	0.002072194	0.004448038
ZNF843	-0.114577403	0.027332127	0.045520616
ZNF844	-0.020848482	0.688966107	0.746117786
ZNF845	0.211766934	3.92E-05	0.000116778
ZNF846	-0.044444439	0.393330295	0.469695267
ZNF84	0.241985609	2.41E-06	8.89E-06
ZNF853	0.194844537	0.000159007	0.000422816
ZNF85	0.345698105	7.47E-12	6.71E-11
ZNF860	0.350444851	3.68E-12	3.46E-11
ZNF862	-0.145500887	0.004984312	0.009826675
ZNF876P	0.192459051	0.000191908	0.000502324
ZNF878	0.286763557	1.87E-08	9.88E-08
ZNF879	0.254752276	6.61E-07	2.68E-06
ZNF880	0.198350225	0.00012012	0.00032696
ZNF883	0.218920357	2.10E-05	6.53E-05
ZNF8	0.384221791	1.69E-14	2.28E-13
ZNF90	0.228775655	8.55E-06	2.86E-05
ZNF91	0.054914268	0.29144801	0.363138861
ZNF92	0.220500933	1.82E-05	5.73E-05
ZNF93	0.435691962	1.28E-18	3.27E-17
ZNF98	0.11431124	0.027694897	0.046039532
ZNF99	0.02793544	0.59170733	0.659320623
ZNFX1	-0.063264898	0.224109119	0.289746374
ZNHIT1	-0.266167266	1.95E-07	8.67E-07
ZNHIT2	-0.001765184	0.972968749	0.978469632
ZNHIT3	0.155917429	0.002599727	0.005468457
ZNHIT6	0.042579389	0.413505489	0.489619991
ZNRD1	0.173022629	0.000817855	0.001900758
ZNRF1	-0.162319512	0.001708191	0.003721224
ZNRF2	-0.198225101	0.000121338	0.000329962
ZNRF3	-0.238815144	3.29E-06	1.19E-05
ZNRF4	0.033080828	0.525294427	0.597380727
ZP1	0.186626818	0.00030109	0.000758991
ZP2	-0.144685914	0.005235915	0.010258645
ZP3	0.17723314	0.000604874	0.001442976
ZPBP2	-0.003506809	0.946328407	0.95854965
ZPBP	0.039365789	0.449665364	0.5254404
ZPLD1	0.346194385	6.94E-12	6.26E-11
ZRANB1	-0.332724025	4.85E-11	3.84E-10
ZRANB2	0.115174065	0.026533706	0.044327421
ZRANB3	-0.136438344	0.008502483	0.01592427
ZRSR2	0.241260995	2.59E-06	9.50E-06
ZSCAN10	0.171016537	0.000942025	0.002159612
ZSCAN12P1	0.291990593	1.00E-08	5.53E-08
ZSCAN12	0.181035385	0.000457949	0.001115723
ZSCAN16	0.263810024	2.53E-07	1.10E-06
ZSCAN18	0.098192296	0.058824415	0.090049178
ZSCAN1	0.01747411	0.73727436	0.787010652

ZSCAN20	0.253207316	7.76E-07	3.11E-06
ZSCAN21	-0.32050029	2.62E-10	1.85E-09
ZSCAN22	-0.034998025	0.501557798	0.575048024
ZSCAN23	0.115824787	0.025685899	0.043051866
ZSCAN29	0.070097102	0.177894215	0.236930642
ZSCAN2	0.02061478	0.692274082	0.749130052
ZSCAN4	0.027812465	0.593340518	0.660918636
ZSCAN5A	0.210722656	4.29E-05	0.000126937
ZSCAN5B	0.139322772	0.007196534	0.0137012
ZSWIM1	0.192971503	0.000184344	0.000484435
ZSWIM2	0.033818332	0.516096191	0.588768729
ZSWIM3	0.077572013	0.135870031	0.187529635
ZSWIM4	0.298148645	4.71E-09	2.74E-08
ZSWIM5	0.108007418	0.037577278	0.06041599
ZSWIM6	0.24564719	1.68E-06	6.32E-06
ZSWIM7	-0.050728506	0.329842926	0.403880195
ZUFSP	0.316324408	4.57E-10	3.11E-09
ZW10	0.015874775	0.760551129	0.806912841
ZWILCH	0.375504069	7.21E-14	8.87E-13
ZWINT	0.512402242	3.17E-26	2.56E-24
ZXDA	-0.061330173	0.238626884	0.305757961
ZXDB	-0.000738244	0.98869308	0.991430188
ZXDC	0.093804433	0.071123984	0.106424215
ZYG11A	0.069153513	0.183819109	0.243502734
ZYG11B	-0.340024312	1.71E-11	1.45E-10
ZYX	0.341805708	1.32E-11	1.14E-10
ZZEF1	0.083271495	0.109312266	0.155331476
ZZZ3	-0.065795469	0.206085006	0.269362649
psiTPTE22	-0.002180057	0.966618894	0.973902878
tAKR	-0.43466179	1.57E-18	3.97E-17
