# Supplementary material for: Exploring the Prognostic Value, Immune Implication and Biological Function of H2AFY Gene in Hepatocellular Carcinoma
Source: Front Immunol. 2021 Nov 24;12:723293. doi: 10.3389/fimmu.2021.723293 (PMC8651705; doi:10.3389/fimmu.2021.723293)
Supplement: Supplementary file 3 [file Table_2.pdf]

**Supplementary Table 2.**

**Biological process terms of H2AFY co-expressed genes.**

| Gene Set | Description | ES | NES | pValue | FDR | Size | LeadingEdgeNum |
|---|---|---|---|---|---|---|---|
| GO:0007059 | chromosome segregation | 0.755762 | 2.270581 | 0 | 0 | 263 | 86 |
| GO:1902850 | microtubule cytoskeleton organization involved in mitosis | 0.765582 | 2.194404 | 0 | 0 | 119 | 48 |
| GO:0050000 | chromosome localization | 0.791856 | 2.146589 | 0 | 0 | 68 | 25 |
| GO:0006260 | DNA replication | 0.707545 | 2.134672 | 0 | 0 | 233 | 82 |
| GO:0007051 | spindle organization | 0.723982 | 2.115171 | 0 | 0 | 152 | 59 |
| GO:0044839 | cell cycle G2/M phase transition | 0.703056 | 2.086181 | 0 | 0 | 192 | 73 |
| GO:0071103 | DNA conformation change | 0.685882 | 2.045259 | 0 | 0 | 220 | 54 |
| GO:0033044 | regulation of chromosome organization | 0.671491 | 2.040344 | 0 | 0 | 305 | 91 |
| GO:0048285 | organelle fission | 0.661249 | 2.021647 | 0 | 0 | 404 | 104 |
| GO:0000075 | cell cycle checkpoint | 0.674409 | 2.019197 | 0 | 0 | 196 | 78 |
| GO:0032200 | telomere organization | 0.692237 | 2.016116 | 0 | 0 | 136 | 61 |
| GO:0051383 | kinetochore organization | 0.933487 | 2.008502 | 0 | 0 | 17 | 10 |
| GO:0044772 | mitotic cell cycle phase transition | 0.652829 | 2.007339 | 0 | 0 | 453 | 144 |

| | | | | | | | |
|---|---|---|---|---|---|---|---|
| GO:0000910 | cytokinesis | 0.679992 | 1.987148 | 0 | 0 | 150 | 42 |
| GO:1901987 | regulation of cell cycle phase transition | 0.646706 | 1.975158 | 0 | 0 | 357 | 125 |
| GO:0034502 | protein localization to chromosome | 0.729937 | 1.967792 | 0 | 0 | 68 | 30 |
| GO:0006333 | chromatin assembly or disassembly | 0.667655 | 1.92881 | 0 | 0 | 138 | 47 |
| GO:0051321 | meiotic cell cycle | 0.646489 | 1.921538 | 0 | 0 | 217 | 56 |
| GO:0006081 | cellular aldehyde metabolic process | -0.63386 | -2.16236 | 0 | 0 | 69 | 27 |
| GO:0033865 | nucleoside bisphosphate metabolic process | -0.60557 | -2.18984 | 0 | 0 | 128 | 45 |
| GO:0055088 | lipid homeostasis | -0.60158 | -2.19643 | 0 | 0 | 121 | 37 |
| GO:0006732 | coenzyme metabolic process | -0.53293 | -2.19717 | 0 | 0 | 331 | 114 |
| GO:0009410 | response to xenobiotic stimulus | -0.54148 | -2.20481 | 0 | 0 | 270 | 87 |
| GO:0042737 | drug catabolic process | -0.62751 | -2.28274 | 0 | 0 | 130 | 49 |
| GO:0008202 | steroid metabolic process | -0.56488 | -2.29951 | 0 | 0 | 291 | 95 |
| GO:0016042 | lipid catabolic process | -0.57321 | -2.30227 | 0 | 0 | 302 | 108 |
| GO:0042537 | benzene-containing compound metabolic process | -0.86725 | -2.32499 | 0 | 0 | 22 | 9 |
| GO:0006631 | fatty acid metabolic process | -0.65476 | -2.4735 | 0 | 0 | 339 | 142 |
| GO:0007031 | peroxisome organization | -0.75491 | -2.56694 | 0 | 0 | 79 | 40 |

| | | | | | | | |
|---|---|---|---|---|---|---|---|
| GO:0044282 | small molecule catabolic process | -0.62726 | -2.58221 | 0 | 0 | 404 | 175 |
| GO:0072376 | protein activation cascade | -0.74746 | -2.58921 | 0 | 0 | 87 | 43 |
| GO:0043574 | peroxisomal transport | -0.78152 | -2.62063 | 0 | 0 | 68 | 35 |
| GO:0061641 | CENP-A containing chromatin organization | 0.817969 | 1.856183 | 0 | 3.94E-05 | 24 | 12 |
| GO:0045930 | negative regulation of mitotic cell cycle | 0.615025 | 1.857686 | 0 | 4.13E-05 | 237 | 78 |
| GO:0010948 | negative regulation of cell cycle process | 0.626301 | 1.88104 | 0 | 4.33E-05 | 257 | 73 |
| GO:0051653 | spindle localization | 0.74987 | 1.886287 | 0 | 4.56E-05 | 40 | 14 |
| GO:0006302 | double-strand break repair | 0.625706 | 1.846904 | 0 | 7.22E-05 | 180 | 65 |
| GO:0045787 | positive regulation of cell cycle | 0.608336 | 1.853364 | 0 | 7.53E-05 | 353 | 117 |
| GO:0051052 | regulation of DNA metabolic process | 0.600601 | 1.827903 | 0 | 9.63E-05 | 367 | 125 |
| GO:0042180 | cellular ketone metabolic process | -0.54611 | -2.08794 | 0 | 1.26E-04 | 176 | 56 |
| GO:1901615 | organic hydroxy compound metabolic process | -0.50406 | -2.1028 | 0 | 1.34E-04 | 481 | 123 |
| GO:0006520 | cellular amino acid metabolic process | -0.55238 | -2.10644 | 0 | 1.43E-04 | 301 | 98 |
| GO:0016999 | antibiotic metabolic process | -0.56676 | -2.07876 | 0 | 2.38E-04 | 141 | 49 |
| GO:0043648 | dicarboxylic acid metabolic process | -0.58468 | -2.04314 | 0 | 6.76E-04 | 102 | 42 |
| GO:0006638 | neutral lipid metabolic process | -0.56902 | -2.01424 | 0 | 0.00107 | 111 | 36 |
| GO:0030258 | lipid modification | -0.51964 | -1.98338 | 0 | 0.001302 | 270 | 81 |

| | | | | | | | |
|---|---|---|---|---|---|---|---|
| GO:0006790 | sulfur compound metabolic process | -0.48908 | -1.99751 | 0 | 0.001325 | 335 | 90 |
| GO:0016053 | organic acid biosynthetic process | -0.49264 | -1.97635 | 0 | 0.001337 | 383 | 122 |
| GO:0002526 | acute inflammatory response | -0.52762 | -1.98808 | 0 | 0.001362 | 151 | 56 |
| GO:0006091 | generation of precursor metabolites and energy | -0.46567 | -1.96607 | 0 | 0.001455 | 418 | 118 |

**Cell component terms of H2AFY co-expressed genes.**

| Gene Set | Description | ES | NES | pValue | FDR | Size | LeadingEdgeNum |
|---|---|---|---|---|---|---|---|
| GO:0000793 | condensed chromosome | 0.757599 | 2.279948 | 0 | 0 | 193 | 74 |
| GO:0098687 | chromosomal region | 0.727267 | 2.229685 | 0 | 0 | 289 | 105 |
| GO:0005819 | spindle | 0.70536 | 2.154457 | 0 | 0 | 292 | 95 |
| GO:0005657 | replication fork | 0.787613 | 2.093952 | 0 | 0 | 62 | 24 |
| GO:0045171 | intercellular bridge | 0.764231 | 2.049313 | 0 | 0 | 55 | 19 |
| GO:0030496 | midbody | 0.689715 | 2.046275 | 0 | 0 | 158 | 50 |
| GO:0032994 | protein-lipid complex | -0.7298 | -2.14205 | 0 | 0 | 36 | 17 |
| GO:0005759 | mitochondrial matrix | -0.56756 | -2.33663 | 0 | 0 | 415 | 146 |
| GO:0042579 | microbody | -0.71381 | -2.71706 | 0 | 0 | 125 | 63 |
| GO:0044450 | microtubule organizing center part | 0.594149 | 1.766649 | 0 | 4.32E-04 | 150 | 50 |

| | | | | | | | |
|---|---|---|---|---|---|---|---|
| GO:0005874 | microtubule | 0.592283 | 1.815278 | 0 | 4.86E-04 | 370 | 99 |
| GO:0005875 | microtubule associated complex | 0.631237 | 1.845532 | 0 | 5.56E-04 | 143 | 41 |
| GO:0005681 | spliceosomal complex | 0.583839 | 1.754274 | 0 | 9.73E-04 | 155 | 65 |
| GO:0034399 | nuclear periphery | 0.592632 | 1.737198 | 0 | 0.001135 | 127 | 39 |
| GO:0072562 | blood microparticle | -0.54552 | -1.96423 | 0 | 0.001207 | 103 | 40 |
| GO:0000792 | heterochromatin | 0.636662 | 1.7385 | 0 | 0.001238 | 72 | 32 |
| GO:0043073 | germ cell nucleus | 0.785675 | 1.721277 | 0.002747 | 0.001668 | 18 | 6 |
| GO:0000803 | sex chromosome | 0.708712 | 1.716538 | 0 | 0.001686 | 29 | 19 |
| GO:0120114 | Sm-like protein family complex | 0.621832 | 1.722763 | 0 | 0.001796 | 69 | 41 |
| GO:0044322 | endoplasmic reticulum quality control compartment | -0.67044 | -1.87145 | 0 | 0.002414 | 23 | 10 |
| GO:0044815 | DNA packaging complex | 0.606358 | 1.655072 | 0 | 0.005108 | 66 | 11 |
| GO:0090734 | site of DNA damage | 0.606753 | 1.634864 | 0 | 0.007097 | 59 | 20 |
| GO:0032153 | cell division site | 0.583892 | 1.626896 | 0 | 0.007567 | 67 | 22 |
| GO:0042827 | platelet dense granule | -0.62877 | -1.71272 | 0 | 0.008853 | 20 | 9 |
| GO:0034708 | methyltransferase complex | 0.564617 | 1.60952 | 0 | 0.00891 | 105 | 41 |
| GO:0000790 | nuclear chromatin | 0.505515 | 1.558153 | 0 | 0.020723 | 318 | 109 |

| | | | | | | | |
|---|---|---|---|---|---|---|---|
| GO:0005697 | telomerase holoenzyme complex | 0.682423 | 1.53891 | 0.011019 | 0.026964 | 20 | 12 |
| GO:0005743 | mitochondrial inner membrane | -0.38516 | -1.59402 | 0 | 0.030354 | 369 | 97 |
| GO:0048770 | pigment granule | 0.524632 | 1.504592 | 0.002247 | 0.036647 | 103 | 35 |
| GO:0000791 | euchromatin | 0.613755 | 1.508056 | 0.014963 | 0.036886 | 32 | 8 |
| GO:1904949 | ATPase complex | 0.517964 | 1.509489 | 0 | 0.038209 | 100 | 37 |
| GO:0101031 | chaperone complex | 0.664395 | 1.487058 | 0.029412 | 0.043042 | 21 | 12 |
| GO:0070971 | endoplasmic reticulum exit site | -0.55235 | -1.51278 | 0.049296 | 0.04668 | 24 | 8 |
| GO:0005801 | cis-Golgi network | -0.48243 | -1.51628 | 0.020202 | 0.051006 | 49 | 22 |
| GO:1990204 | oxidoreductase complex | -0.38638 | -1.3823 | 0 | 0.081489 | 95 | 25 |
| GO:0045178 | basal part of cell | -0.42639 | -1.40411 | 0.033708 | 0.087404 | 49 | 21 |
| GO:0000407 | phagophore assembly site | -0.48366 | -1.38338 | 0.092308 | 0.08758 | 25 | 6 |
| GO:0005788 | endoplasmic reticulum lumen | -0.33569 | -1.3291 | 0 | 0.109952 | 291 | 58 |
| GO:0098798 | mitochondrial protein complex | -0.28994 | -1.17965 | 0 | 0.276286 | 213 | 45 |
| GO:0035327 | transcriptionally active chromatin | -0.43826 | -1.14488 | 0.225806 | 0.298642 | 20 | 4 |
| GO:0031970 | organelle envelope lumen | -0.32888 | -1.1536 | 0.192308 | 0.305513 | 73 | 6 |
| GO:0031091 | platelet alpha granule | -0.30878 | -1.11857 | 0.231884 | 0.305835 | 88 | 22 |
| GO:0005811 | lipid droplet | -0.31462 | -1.09842 | 0.192982 | 0.308571 | 72 | 14 |

| | | | | | | | |
|---|---|---|---|---|---|---|---|
| GO:0044455 | mitochondrial membrane part | -0.28759 | -1.12415 | 0.060606 | 0.31573 | 167 | 38 |
| GO:0005790 | smooth endoplasmic reticulum | -0.38467 | -1.10055 | 0.333333 | 0.32011 | 34 | 4 |
| GO:0009295 | nucleoid | -0.3472 | -1.0532 | 0.387931 | 0.381719 | 36 | 15 |
| GO:0070469 | respiratory chain | -0.29968 | -1.03965 | 0.378788 | 0.39411 | 84 | 32 |
| GO:0030964 | NADH dehydrogenase complex | -0.314 | -0.96634 | 0.481013 | 0.550398 | 43 | 15 |
| GO:0097386 | glial cell projection | -0.31561 | -0.86466 | 0.757812 | 0.769517 | 22 | 4 |

**Molecular function terms of H2AFY co-expressed genes.**

| **Gene Set** | **Description** | **ES** | **NES** | **pValue** | **FDR** | **Size** | **LeadingEdgeNum** |
|---|---|---|---|---|---|---|---|
| GO:0140097 | catalytic activity, acting on DNA | 0.629617 | 1.866057 | 0 | 0.003209 | 170 | 53 |
| GO:0031491 | nucleosome binding | 0.672972 | 1.820134 | 0 | 0.001605 | 66 | 24 |
| GO:0097472 | cyclin-dependent protein kinase activity | 0.722269 | 1.775187 | 0 | 0.005349 | 36 | 10 |
| GO:0004386 | helicase activity | 0.594905 | 1.748667 | 0 | 0.006017 | 142 | 35 |
| GO:0015631 | tubulin binding | 0.563254 | 1.738632 | 0 | 0.005456 | 292 | 91 |
| GO:0003684 | damaged DNA binding | 0.625338 | 1.710807 | 0 | 0.008825 | 67 | 18 |
| GO:0003697 | single-stranded DNA binding | 0.59318 | 1.688286 | 0 | 0.010545 | 93 | 36 |
| GO:0017124 | SH3 domain binding | 0.558209 | 1.637249 | 0 | 0.023668 | 123 | 65 |

| | | | | | | | |
|---|---|---|---|---|---|---|---|
| GO:0008327 | methyl-CpG binding | 0.695624 | 1.63434 | 0.005362 | 0.022643 | 21 | 8 |
| GO:0036002 | pre-mRNA binding | 0.662144 | 1.625472 | 0.002494 | 0.025192 | 31 | 15 |
| GO:0000217 | DNA secondary structure binding | 0.707903 | 1.60787 | 0.00545 | 0.030634 | 22 | 12 |
| GO:0003774 | motor activity | 0.543825 | 1.587567 | 0 | 0.041586 | 134 | 37 |
| GO:0017048 | Rho GTPase binding | 0.529548 | 1.584644 | 0 | 0.039498 | 162 | 54 |
| GO:0031490 | chromatin DNA binding | 0.552444 | 1.581847 | 0 | 0.038282 | 106 | 30 |
| GO:0042393 | histone binding | 0.525545 | 1.564577 | 0 | 0.045785 | 174 | 52 |
| GO:0030215 | semaphorin receptor binding | 0.670459 | 1.539021 | 0.013228 | 0.061477 | 23 | 10 |
| GO:0016887 | ATPase activity | 0.491971 | 1.526949 | 0 | 0.068621 | 406 | 108 |
| GO:0140103 | catalytic activity, acting on a glycoprotein | 0.671069 | 1.518003 | 0.018817 | 0.072386 | 22 | 6 |
| GO:0017056 | structural constituent of nuclear pore | 0.652978 | 1.517407 | 0.012987 | 0.068998 | 22 | 13 |
| GO:0001047 | core promoter binding | 0.570934 | 1.505662 | 0.009926 | 0.076941 | 43 | 13 |
| GO:0003727 | single-stranded RNA binding | 0.530045 | 1.500429 | 0.009195 | 0.078473 | 82 | 39 |
| GO:0004674 | protein serine/threonine kinase activity | 0.476886 | 1.492653 | 0 | 0.083367 | 427 | 106 |
| GO:0004712 | protein serine/threonine/tyrosine kinase activity | 0.583651 | 1.487887 | 0.018135 | 0.084068 | 43 | 9 |
| GO:0005112 | Notch binding | 0.668244 | 1.486696 | 0.019126 | 0.082103 | 19 | 9 |

| | | | | | | | |
|---|---|---|---|---|---|---|---|
| GO:0050839 | cell adhesion molecule binding | 0.475668 | 1.476138 | 0 | 0.09095 | 449 | 154 |
| GO:1901338 | catecholamine binding | -0.6172 | -1.60647 | 0.036765 | 0.033368 | 19 | 5 |
| GO:0005319 | lipid transporter activity | -0.44217 | -1.63412 | 0 | 0.028812 | 139 | 46 |
| GO:0005342 | organic acid transmembrane transporter activity | -0.44178 | -1.6414 | 0 | 0.028758 | 149 | 40 |
| GO:0016790 | thiolester hydrolase activity | -0.57611 | -1.68422 | 0.008197 | 0.02167 | 31 | 12 |
| GO:0016769 | transferase activity, transferring nitrogenous groups | -0.69112 | -1.71689 | 0.021429 | 0.017589 | 19 | 10 |
| GO:0001221 | transcription cofactor binding | -0.5591 | -1.72785 | 0.010753 | 0.01675 | 41 | 10 |
| GO:1901681 | sulfur compound binding | -0.44535 | -1.73436 | 0 | 0.016954 | 216 | 50 |
| GO:0043177 | organic acid binding | -0.44028 | -1.75498 | 0 | 0.015271 | 198 | 63 |
| GO:0019840 | isoprenoid binding | -0.55185 | -1.75829 | 0 | 0.015411 | 42 | 16 |
| GO:0052689 | carboxylic ester hydrolase activity | -0.47604 | -1.75885 | 0 | 0.016106 | 126 | 31 |
| GO:0016829 | lyase activity | -0.47484 | -1.78464 | 0 | 0.012312 | 157 | 52 |
| GO:0009055 | electron transfer activity | -0.49228 | -1.80859 | 0 | 0.010431 | 104 | 30 |
| GO:0019825 | oxygen binding | -0.60931 | -1.84274 | 0 | 0.007268 | 31 | 13 |

| | | | | | | | |
|---|---|---|---|---|---|---|---|
| GO:0016645 | oxidoreductase activity, acting on the CH-NH group of donors | -0.69696 | -2.0188 | 0 | 3.58E-04 | 24 | 12 |
| GO:0016705 | oxidoreductase activity, acting on paired donors, with incorporation or reduction of molecular oxygen | -0.54154 | -2.07188 | 0 | 0 | 142 | 52 |
| GO:0005506 | iron ion binding | -0.55193 | -2.07571 | 0 | 0 | 134 | 49 |
| GO:0046906 | tetrapyrrole binding | -0.54678 | -2.10772 | 0 | 0 | 128 | 45 |
| GO:0098531 | transcription factor activity, direct ligand regulated sequence-specific DNA binding | -0.6721 | -2.11333 | 0 | 0 | 47 | 14 |
| GO:0019842 | vitamin binding | -0.58364 | -2.17525 | 0 | 0 | 120 | 44 |
| GO:0016903 | oxidoreductase activity, acting on the aldehyde or oxo group of donors | -0.72523 | -2.21345 | 0 | 0 | 43 | 20 |
| GO:0003707 | steroid hormone receptor activity | -0.68119 | -2.21893 | 0 | 0 | 52 | 16 |
| GO:0016229 | steroid dehydrogenase activity | -0.75641 | -2.22912 | 0 | 0 | 31 | 11 |
| GO:0004497 | monooxygenase activity | -0.6792 | -2.4025 | 0 | 0 | 90 | 45 |
| GO:0016627 | oxidoreductase activity, acting on the CH-CH group of donors | -0.7278 | -2.42108 | 0 | 0 | 56 | 32 |

| | | | | | | | |
|---|---|---|---|---|---|---|---|
| GO:0016614 | oxidoreductase activity, acting on CH-OH group of donors | -0.67439 | -2.50691 | 0 | 0 | 129 | 49 |

ES: Enrichment score; NES: Normalized enrichment score; FDR: false discovery rate.
