# Supplementary material for: Exploring the Prognostic Value, Immune Implication and Biological Function of H2AFY Gene in Hepatocellular Carcinoma
Source: Front Immunol. 2021 Nov 24;12:723293. doi: 10.3389/fimmu.2021.723293 (PMC8651705; doi:10.3389/fimmu.2021.723293)
Supplement: Supplementary file 4 [file Table_3.pdf]

Supplementary Table 3. KEGG annotation of H2AFY co-expressed genes.

Gene set	Description	ES	NES	pValue	FDR	Size	LeadingEdgeNum
hsa04110	Cell cycle	0.737074	2.096602	0	0	118	48
hsa03030	DNA replication	0.804531	1.98063	0	0	36	19
hsa03440	Homologous recombination	0.749357	1.84992	0	0	34	13
hsa03040	Spliceosome	0.631871	1.801283	0	0.001042	115	54
hsa03430	Mismatch repair	0.791621	1.794321	0	0.001167	23	9
hsa04114	Oocyte meiosis	0.600679	1.719537	0	0.005975	118	41
hsa03460	Fanconi anemia pathway	0.665981	1.694938	0.001235	0.008218	44	17
hsa03410	Base excision repair	0.691031	1.676685	0.001285	0.009588	33	9
hsa05130	Pathogenic Escherichia coli infection	0.641596	1.671678	0.001198	0.00982	53	24
hsa05131	Shigellosis	0.623039	1.658258	0	0.011339	63	25
hsa05206	MicroRNAs in cancer	0.571406	1.641496	0	0.013479	149	39
hsa03015	mRNA surveillance pathway	0.596755	1.643912	0	0.014022	87	32
hsa04914	Progesterone-mediated oocyte maturation	0.572255	1.605205	0	0.0245	94	16
hsa04666	Fc gamma R-mediated phagocytosis	0.574647	1.59716	0	0.026204	86	40
hsa05219	Bladder cancer	0.634002	1.577918	0.001252	0.035796	40	16

hsa05132	Salmonella infection	0.570449	1.574586	0.001133	0.035852	83	33
hsa05161	Hepatitis B	0.536619	1.553984	0.001072	0.045808	135	33
hsa05323	Rheumatoid arthritis	0.557384	1.536578	0.001117	0.056325	85	50
hsa04115	p53 signaling pathway	0.560123	1.518498	0.008111	0.062889	69	19
hsa05166	Human T-cell leukemia virus 1 infection	0.509768	1.523642	0	0.064594	253	98
hsa04672	Intestinal immune network for IgA production	0.594371	1.519996	0.007186	0.064825	45	29
hsa04260	Cardiac muscle contraction	0.557108	1.511752	0.001163	0.066587	74	16
hsa03013	RNA transport	0.505275	1.479223	0	0.079466	158	47
hsa04912	GnRH signaling pathway	0.533234	1.481266	0.00452	0.08016	91	30
hsa04657	IL-17 signaling pathway	0.530137	1.475028	0.004551	0.08118	89	24
hsa00620	Pyruvate metabolism	-0.68821	-2.02927	0	0	38	19
hsa00630	Glyoxylate and dicarboxylate metabolism	-0.73921	-2.06378	0	0	28	19
hsa00860	Porphyrin and chlorophyll metabolism	-0.67437	-2.0679	0	0	40	16
hsa00040	Pentose and glucuronate interconversions	-0.73078	-2.13023	0	0	30	16
hsa00650	Butanoate metabolism	-0.79712	-2.20865	0	0	27	14
hsa00053	Ascorbate and aldarate metabolism	-0.80458	-2.22189	0	0	25	15
hsa00120	Primary bile acid biosynthesis	-0.89156	-2.23822	0	0	17	13

hsa00350	Tyrosine metabolism	-0.75354	-2.28136	0	0	36	17
hsa00640	Propanoate metabolism	-0.80829	-2.32856	0	0	31	19
hsa01212	Fatty acid metabolism	-0.75082	-2.33169	0	0	44	21
hsa03320	PPAR signaling pathway	-0.70957	-2.34422	0	0	74	32
hsa00260	Glycine, serine and threonine metabolism	-0.80841	-2.41769	0	0	39	22
hsa00980	Metabolism of xenobiotics by cytochrome P450	-0.73279	-2.43211	0	0	70	37
hsa00140	Steroid hormone biosynthesis	-0.75012	-2.43738	0	0	57	27
hsa04610	Complement and coagulation cascades	-0.71067	-2.45904	0	0	78	36
hsa00380	Tryptophan metabolism	-0.83318	-2.47575	0	0	37	21
hsa00280	Valine, leucine and isoleucine degradation	-0.81052	-2.52923	0	0	47	34
hsa05204	Chemical carcinogenesis	-0.75688	-2.53488	0	0	75	38
hsa00982	Drug metabolism	-0.78533	-2.60987	0	0	66	38
hsa00071	Fatty acid degradation	-0.88482	-2.69205	0	0	42	32
hsa04146	Peroxisome	-0.80109	-2.73636	0	0	82	40
hsa00830	Retinol metabolism	-0.81332	-2.74173	0	0	63	34
hsa00220	Arginine biosynthesis	-0.74422	-1.98297	0	3.06E-04	20	13

hsa00310	Lysine degradation	-0.63934	-1.9928	0	3.19E-04	44	15
hsa00020	Citrate cycle (TCA cycle)	-0.68381	-1.9977	0	3.32E-04	30	15

ES: Enrichment score; NES: Normalized enrichment score; FDR: false discovery rate.
