# Supplementary material for: Exploring the Prognostic Value, Immune Implication and Biological Function of H2AFY Gene in Hepatocellular Carcinoma
Source: Front Immunol. 2021 Nov 24;12:723293. doi: 10.3389/fimmu.2021.723293 (PMC8651705; doi:10.3389/fimmu.2021.723293)
Supplement: Supplementary file 5 [file Table_4.pdf]

Supplementary Table 4. Kinases enrichment of H2AFY co-expressed genes.

GeneSet	Description	ES	NES	pValue	FDR	Size	LeadingEdgeNum
Kinase_PLK1	polo like kinase 1	0.734122	2.034636	0	0	91	32
Kinase_CDK1	cyclin dependent kinase 1	0.6558	1.984308	0	0	258	77
Kinase_CHEK1	checkpoint kinase 1	0.674386	1.955393	0	0	130	34
Kinase_AURKB	aurora kinase B	0.674169	1.868953	0	2.65E-04	87	24
Kinase_CDK2	cyclin dependent kinase 2	0.60153	1.818745	0	5.31E-04	278	90
Kinase_CHEK2	checkpoint kinase 2	0.767176	1.820614	0	6.07E-04	27	12
Kinase_AURKA	aurora kinase A	0.710876	1.855246	0	6.37E-04	46	17
Kinase_ATR	ATR serine/threonine kinase	0.668697	1.824901	0	7.08E-04	66	24
Kinase_PRKCI	protein kinase C iota	0.794764	1.777468	0	0.00118	21	9
Kinase_TTK	TTK protein kinase	0.877967	1.738764	0	0.002414	12	5
Kinase_ATM	ATM serine/threonine kinase	0.609283	1.749292	0	0.002549	123	40
Kinase_BRSK1	BR serine/threonine kinase 1	0.958893	1.68839	0	0.006195	6	4
Kinase_MAPKAPK2	mitogen-activated protein kinase-activated protein kinase 2	0.672983	1.672693	0.002503	0.007597	36	14
Kinase_PRKD3	protein kinase D3	0.882943	1.605061	0.002899	0.025753	8	3

Kinase_CAMK4	calcium/calmodulin dependent protein kinase IV	0.729562	1.59375	0.005602	0.026661	18	5
Kinase_LATS1	large tumor suppressor kinase 1	0.954126	1.605279	0	0.027328	5	1
Kinase_BRAF	B-Raf proto-oncogene, serine/threonine kinase	0.748092	1.59413	0.002766	0.027848	16	6
Kinase_PRKD1	protein kinase D1	0.636836	1.607102	0.004902	0.028143	45	21
Kinase_PLK3	polo like kinase 3	0.718384	1.582522	0.005472	0.028623	20	12
Kinase_PRKDC	protein kinase, DNA-activated, catalytic polypeptide	0.619807	1.595096	0	0.029048	46	24
Kinase_PKN2	protein kinase N2	0.890499	1.584606	0.001456	0.029258	7	3
Kinase_PLK4	polo like kinase 4	0.936106	1.566137	0.001524	0.037459	5	3
Kinase_PRKD2	protein kinase D2	0.802499	1.557152	0.007143	0.042526	11	7
Kinase_PRKCH	protein kinase C eta	0.762357	1.553207	0.005517	0.043143	13	8
Kinase_CAMK2A	calcium/calmodulin dependent protein kinase II alpha	0.598567	1.549382	0.007084	0.044561	53	20
Kinase_PIM1	Pim-1 proto-oncogene, serine/threonine kinase	-0.7412	-1.87362	0	0.050433	19	4

Kinase_NTRK2	neurotrophic receptor tyrosine kinase 2	-0.53213	-1.21524	0.215488	0.760556	11	1
Kinase_DYRK1A	dual specificity tyrosine phosphorylation regulated kinase 1A	-0.50847	-1.21845	0.195205	0.796147	14	3
Kinase_RPS6KB1	ribosomal protein S6 kinase B1	-0.49764	-1.31675	0.083333	0.811282	23	3
Kinase_RPS6KB2	ribosomal protein S6 kinase B2	-0.69823	-1.33397	0.178451	0.819787	6	1
Kinase_NTRK3	neurotrophic receptor tyrosine kinase 3	-0.56809	-1.22455	0.208609	0.826416	9	1
Kinase_PIK3CA	phosphatidylinositol-4,5-bisphosphate 3-kinase catalytic subunit alpha	-0.5673	-1.29406	0.171233	0.827169	10	4
Kinase_JAK1	Janus kinase 1	-0.62366	-1.16999	0.304795	0.84568	6	1
Kinase_TNK2	tyrosine kinase non receptor 2	-0.69377	-1.24874	0.216359	0.857779	5	1
Kinase_MAPK9	mitogen-activated protein kinase 9	-0.40209	-1.22636	0.117347	0.875403	41	9
Kinase_CDK16	cyclin dependent kinase 16	-0.84798	-1.33896	0.152231	0.88902	3	3
Kinase_SGK3	serum/glucocorticoid regulated kinase family member 3	-0.71598	-1.13951	0.371585	0.891779	3	1
Kinase_CAMKK2	calcium/calmodulin dependent protein kinase kinase 2	-0.59567	-1.25448	0.186544	0.904236	8	2
Kinase_CDK3	cyclin dependent kinase 3	-0.52402	-1.04833	0.391176	0.911671	7	1

Kinase_DYRK1B	dual specificity tyrosine phosphorylation regulated kinase 1B	-0.52546	-1.05275	0.39441	0.935395	7	2
Kinase_SGK1	serum/glucocorticoid regulated kinase 1	-0.37549	-0.99974	0.41791	0.94266	21	2
Kinase_PIK3CB	phosphatidylinositol-4,5-bisphosphate 3-kinase catalytic subunit beta	-0.47915	-1.01179	0.448718	0.944386	8	3
Kinase_STK39	serine/threonine kinase 39	-0.49701	-1.01926	0.439759	0.958754	8	3
Kinase_FER	FER tyrosine kinase	-0.46315	-0.96821	0.496753	0.965817	8	1
Kinase_MTOR	mechanistic target of rapamycin	-0.33972	-1.0559	0.307692	0.965825	46	3
Kinase_MAP3K2	mitogen-activated protein kinase kinase kinase 2	-0.67488	-1.34599	0.16358	0.968416	7	5
Kinase_MAP3K11	mitogen-activated protein kinase kinase kinase 11	-0.4856	-0.97423	0.469208	0.980555	7	7
Kinase_NTRK1	neurotrophic receptor tyrosine kinase 1	-0.46341	-1.06356	0.344828	0.98472	12	1
Kinase_FGFR2	fibroblast growth factor receptor 2	-0.38352	-0.63066	0.885638	0.990706	3	2
Kinase_DAPK3	death associated protein kinase 3	-0.2357	-0.60011	0.972881	0.995179	17	2

ES: Enrichment score; NES: Normalized enrichment score; FDR: false discovery rate.
