# Supplementary material for: Exploring the Prognostic Value, Immune Implication and Biological Function of H2AFY Gene in Hepatocellular Carcinoma
Source: Front Immunol. 2021 Nov 24;12:723293. doi: 10.3389/fimmu.2021.723293 (PMC8651705; doi:10.3389/fimmu.2021.723293)
Supplement: Supplementary file 6 [file Table_5.pdf]

Supplementary Table 5. miRNA enrichment of H2AFY co-expressed genes.

Gene set	ES	NES	pValue	FDR	Size	LeadingEdgeNum
GAGCCAG,MIR-149	0.54543	1.582008	0	0.256383	135	43
TAGGTCA,MIR-192,MIR-215	-0.49741	-1.51601	0.020408	0.379246	42	7
GCAAGAC,MIR-431	0.54421	1.374646	0.059611	0.434887	43	15
ACACTCC,MIR-122A	0.513054	1.37558	0.03298	0.472815	65	22
GGGGCCC,MIR-296	0.506384	1.35156	0.044153	0.475381	67	12
GTGGTGA,MIR-197	0.514972	1.38212	0.027251	0.488283	67	24
CACTGCC,MIR-34A,MIR-34C,MIR-449	0.451176	1.356489	0.006192	0.488397	263	78
CCAGGTT,MIR-490	0.53605	1.44728	0.016687	0.49243	60	20
TCCCCAC,MIR-491	0.561224	1.46328	0.017094	0.524778	55	17
GGTGAAG,MIR-412	0.501643	1.328035	0.056166	0.531505	55	13
AAACCAC,MIR-140	0.470553	1.333922	0.033105	0.536448	100	41
GTGTGAG,MIR-342	0.524431	1.382919	0.044864	0.5452	59	21
CTACCTC,LET-7A,LET-7B,LET-7C,LET-7D,LET-7E,LET-7F,MIR-98,LET-7G,LET-7I	0.42068	1.287281	0.00804	0.549786	364	109
GTGACTT,MIR-224	0.439037	1.277271	0.045359	0.554209	150	34

AACATTC,MIR-409-3P	0.448823	1.291339	0.049676	0.554344	132	29
CTCAGGG,MIR-125B,MIR-125A	0.42059	1.280851	0.018256	0.559503	304	82
CAGGGTC,MIR-504	0.454412	1.26214	0.101382	0.570121	79	18
GCGCTTT,MIR-518B,MIR-518C,MIR-518D	0.591423	1.309246	0.134986	0.573273	18	10
CCAGGGG,MIR-331	0.459531	1.269434	0.087311	0.573762	83	18
AGGTGCA,MIR-500	0.461074	1.292711	0.059887	0.575214	93	17
AGCGCTT,MIR-518F,MIR-518E,MIR-518A	0.595523	1.263209	0.175	0.586148	16	7
CAGCAGG,MIR-370	0.511699	1.486216	0.003219	0.589475	138	32
TTGGAGA,MIR-515-5P,MIR-519E	0.451443	1.295156	0.037199	0.589664	130	30
GGCCAGT,MIR-193A,MIR-193B	0.475151	1.310694	0.040724	0.600028	83	16
CTCTGGA,MIR-520A,MIR-525	0.42841	1.248633	0.079399	0.601777	145	33
AACGGTT,MIR-451	0.722489	1.384224	0.057803	0.613524	10	3
GGCGGCA,MIR-371	-0.32115	-0.57851	0.934718	0.98891	5	1
CTCAAGA,MIR-526B	-0.18947	-0.6506	1	1	69	12
GGATCCG,MIR-127	-0.31094	-0.67807	0.864865	1	10	3
CTAGGAA,MIR-384	-0.22098	-0.72503	0.994152	1	60	5
GTATGAT,MIR-154,MIR-487	-0.22717	-0.76101	0.974522	1	67	11

CGTCTTA,MIR-208	-0.40365	-0.84383	0.682493	1	8	8
TCTATGA,MIR-376A,MIR-376B	-0.25217	-0.85798	0.816176	1	76	14
CTACTAG,MIR-325	-0.35985	-0.87279	0.668966	1	16	3
CAATGCA,MIR-33	-0.26165	-0.9118	0.708333	1	90	13
TGCAAAC,MIR-452	-0.27154	-0.94885	0.612613	1	101	11
ACATATC,MIR-190	-0.29393	-0.96626	0.494253	1	58	5
GCTTGAA,MIR-498	-0.27074	-0.9734	0.504587	1	103	18
GACAATC,MIR-219	-0.26785	-0.98301	0.539474	1	134	20
GTAAGAT,MIR-200A	-0.321	-1.00757	0.403846	1	45	5
GAGCCTG,MIR-484	-0.28889	-1.02523	0.36	1	98	12
GTACAGG,MIR-486	-0.32322	-1.02987	0.372549	1	54	12
GTGTCAA,MIR-514	-0.33579	-1.09498	0.263473	1	59	19
CGGTGTG,MIR-220	-0.62305	-1.10745	0.369048	1	5	1
ATAAGCT,MIR-21	-0.32667	-1.19012	0.085106	1	105	16

ES: Enrichment score; NES: Normalized enrichment score; FDR: false discovery rate.
