# Supplementary material for: Exploring the Prognostic Value, Immune Implication and Biological Function of H2AFY Gene in Hepatocellular Carcinoma
Source: Front Immunol. 2021 Nov 24;12:723293. doi: 10.3389/fimmu.2021.723293 (PMC8651705; doi:10.3389/fimmu.2021.723293)
Supplement: Supplementary file 7 [file Table_6.pdf]

Supplementary Table 6. Transcription factor enrichment of H2AFY co-expressed genes.

Gene set	ES	NES	pValue	FDR	Size	LeadingEdgeNum
V$E2F_Q4	0.65041	1.946087	0	0	212	75
V$E2F_Q6	0.649358	1.945892	0	0	211	75
V$E2F_02	0.645457	1.93066	0	0	218	82
V$E2F1DP1_01	0.643671	1.925811	0	0	218	82
V$E2F1DP2_01	0.643671	1.925811	0	0	218	82
V$E2F4DP2_01	0.643671	1.925811	0	0	218	82
V$E2F4DP1_01	0.642795	1.923499	0	0	220	71
V$E2F1_Q6	0.644003	1.921749	0	0	213	74
SGCGSSAAA_V$E2F1DP2_01	0.651521	1.909875	0	0	155	63
V$E2F1_Q6_01	0.619077	1.858374	0	0	218	84
V$E2F1DP1RB_01	0.621487	1.857974	0	0	210	65
V$E2F1_Q3	0.611411	1.829281	0	0	225	79
V$E2F_Q4_01	0.601261	1.800357	0	0	215	62
V$E2F_Q3_01	0.597288	1.787333	0	0	215	63
V$E2F_Q3	0.592382	1.763757	0	2.31E-04	204	61

V$E2F1_Q4_01	0.592	1.767124	0	2.47E-04	210	60
V$E2F_Q6_01	0.586737	1.760953	0	2.72E-04	219	89
V$E2F_01	0.629552	1.698743	0	7.71E-04	64	24
V$E2F_03	0.564469	1.697794	0	7.79E-04	224	62
V$E2F1_Q4	0.557861	1.673114	0	0.001387	222	63
KTGGYRSGAA_UNKNOWN	0.576242	1.54525	0.001202	0.016997	68	27
GCGSCMNTTT_UNKNOWN	0.571318	1.542074	0.00241	0.017359	65	37
KCCGNSWTTT_UNKNOWN	0.533712	1.493974	0.004469	0.036718	95	32
V$E47_01	0.500694	1.494111	0	0.038314	234	87
RYTGCNWTGGNR_UNKNOWN	0.523664	1.475547	0.004505	0.046234	103	35
V$STAT3_01	-0.44326	-1.14146	0.284615	0.45809	19	4
V$CEBP_Q3	-0.2903	-1.14342	0.125	0.471207	238	36
V$HNF4_DR1_Q3	-0.2899	-1.13128	0.086957	0.476395	237	40
V$FOX_Q2	-0.29529	-1.15411	0.055556	0.476734	201	35
RGTTAMWNATT_V$HNF1_01	-0.34375	-1.1459	0.171598	0.483311	69	12
V$COUP_01	-0.2911	-1.15757	0.096774	0.488025	238	34
V$IPF1_Q4	-0.28352	-1.11959	0.064516	0.488698	230	37

V$FOXO1_02	-0.29646	-1.16271	0.066667	0.495547	226	41
V$FREAC2_01	-0.28186	-1.11985	0.083333	0.507432	234	40
V$GFI1_01	-0.30147	-1.16587	0.035714	0.512729	249	48
RYTAAWNNNTGAY_UNKNOWN	-0.35651	-1.16784	0.141975	0.536722	61	7
YNTTTNNNANGCARM_UNKNOWN	-0.34951	-1.17349	0.132353	0.541309	66	10
V$HNF1_C	-0.31004	-1.17528	0	0.570932	222	39
GATAAGR_V$GATA_C	-0.3135	-1.18136	0.045455	0.582855	278	40
AAAYWAACM_V$HFH4_01	-0.30098	-1.18395	0.027778	0.61824	232	34
V$HNF4_01_B	-0.27625	-1.07955	0.142857	0.658301	228	38
V$CEBP_Q2	-0.27767	-1.0757	0.181818	0.658848	213	34
V$HNF3B_01	-0.30985	-1.18436	0.052632	0.670951	196	32
V$PAX2_02	-0.27318	-1.06723	0.225	0.682976	241	30
V$CEBPA_01	-0.30293	-1.18656	0.047619	0.722222	228	23
V$HNF3_Q6	-0.3119	-1.19585	0.055556	0.738925	176	32
V$STAT5B_01	-0.25211	-1.00935	0.346154	0.767916	226	31
RTTTNNNYTGGM_UNKNOWN	-0.27197	-1.01179	0.363636	0.771925	146	18
V$MEF2_03	-0.25689	-1.00433	0.483871	0.778971	222	36

V$FOXO1_01	-0.2595	-1.01323	0.439024	0.781786	220	25

ES: Enrichment score; NES: Normalized enrichment score; FDR: false discovery rate.
